# The mitochondrial genome sequence of *Abies alba* Mill. reveals a high structural and combinatorial variation

**DOI:** 10.1186/s12864-022-08993-9

**Published:** 2022-11-28

**Authors:** Birgit Kersten, Christian Rellstab, Hilke Schroeder, Sabine Brodbeck, Matthias Fladung, Konstantin V. Krutovsky, Felix Gugerli

**Affiliations:** 1 Thünen Institute of Forest Genetics, Sieker Landstrasse 2, 22927 Grosshansdorf, Germany; 2 Swiss Federal Research Institute WSL, Zürcherstrasse 111, 8903 Birmensdorf, Switzerland; 3 Department of Forest Genetics and Forest Tree Breeding, Georg-August University of Göttingen, Büsgenweg 2, 37077 Göttingen, Germany

**Keywords:** Silver fir, Mitochondrial genome, Genome assembly, Long-read sequencing, Repetitive elements, mtDNA

## Abstract

**Background:**

Plant mitogenomes vary widely in size and genomic architecture. Although hundreds of plant mitogenomes of angiosperm species have already been sequence-characterized, only a few mitogenomes are available from gymnosperms. Silver fir (*Abies alba)* is an economically important gymnosperm species that is widely distributed in Europe and occupies a large range of environmental conditions. Reference sequences of the nuclear and chloroplast genome of *A. alba* are available, however, the mitogenome has not yet been assembled and studied.

**Results:**

Here, we used paired-end Illumina short reads generated from a single haploid megagametophyte in combination with PacBio long reads from high molecular weight DNA of needles to assemble the first mitogenome sequence of *A. alba*. Assembly and scaffolding resulted in 11 mitogenome scaffolds, with the largest scaffold being 0.25 Mbp long. Two of the scaffolds displayed a potential circular structure supported by PCR. The total size of the *A. alba* mitogenome was estimated at 1.43 Mbp, similar to the size (1.33 Mbp) of a draft assembly of the *Abies firma* mitogenome. In total, 53 distinct genes of known function were annotated in the *A. alba* mitogenome, comprising 41 protein-coding genes, nine tRNA, and three rRNA genes. The proportion of highly repetitive elements (REs) was 0.168. The mitogenome seems to have a complex and dynamic structure featured by high combinatorial variation, which was specifically confirmed by PCR for the contig with the highest mapping coverage. Comparative analysis of all sequenced mitogenomes of gymnosperms revealed a moderate, but significant positive correlation between mitogenome size and proportion of REs.

**Conclusions:**

The *A. alba* mitogenome provides a basis for new comparative studies and will allow to answer important structural, phylogenetic and other evolutionary questions. Future long-read sequencing with higher coverage of the *A. alba* mitogenome will be the key to further resolve its physical structure. The observed positive correlation between mitogenome size and proportion of REs will be further validated once available mitogenomes of gymnosperms would become more numerous. To test whether a higher proportion of REs in a mitogenome leads to an increased recombination and higher structural complexity and variability is a prospective avenue for future research.

**Supplementary Information:**

The online version contains supplementary material available at 10.1186/s12864-022-08993-9.

## Background

The mitochondrial genomes (mitogenomes) of eukaryotes exhibit an amazing diversity of genomic architectures. In contrast to the relatively compact and small mitogenomes of most animal species (about 15–20 Kbp) [1], mitogenome sizes in land plants vary extremely, ranging from about 0.22 Mbp in *Brassica napus* [2] to 11.66 Mbp in *Larix sibirica* [3, 4]. Thus far, all angiosperms and most gymnosperms have been found to show maternal inheritance of mitochondrial DNA (mtDNA) [5], but paternal inheritance has been also reported, e.g. in *Sequoia sempervirens* [6].

The variability of plant mitogenome sizes arise primarily from a high abundance of interspersed repetitive elements (REs) (including non-tandem repeats of 50 bp and longer), intron expansion and incorporation of plastid and nuclear DNA by intracellular gene transfer [7–21]. Moreover, foreign mtDNA from other plant species can be obtained by horizontal gene transfer [22, 23]. Plant mitogenomes consist of between 0.56% (*Marchantia polymorpha*) and 10.86% (*Phoenix dactylifera*) plastid-derived DNA sequences (MTPTs) [24]. The integration of nuclear sequences is usually more complex, as it involves retrotransposons and other REs [19]. Other observed gene transfer involving the mitogenomes are transfers mostly from the mitogenome to the nucleus and rarely from the mitogenome to plastids [19, 25–28]. A large number of protein-coding genes have been lost by gene transfer in some lineages of angiosperms [14, 29] and gymnosperms [30]. However, core mitochondrial gene sets are relatively conserved in the oldest angiosperm groups [13, 31–33].

In mitogenomes of land plants, DNA double strand breaks are rampant [34, 35]. The mitogenome sequences of angiosperms generally have one or more pairs of large non-tandem repeats that can act as sites for inter- and intramolecular recombination, leading to multiple alternative arrangements within a species (isoforms, including subgenomic forms) [16]. Although plant mitogenomes are often assembled and displayed as circular maps (e.g., [36]), plant mtDNA most likely does not exist as one large circular DNA molecule, but mostly as a complex and dynamic collection of linear DNA with combinations of smaller circular and branched DNA molecules [3, 11, 15–17, 37–43].

All these special features of mitogenomes of land plants make their complete and high-quality assembly and annotation a challenging task. Thus, it is not surprising that only 309, mostly angiosperm mitogenomes are available for land plants to date [4]. Considering extant gymnosperms, comprising 12 families and 83 genera with more than 1,000 known species, only a handful of mitogenomes were fully assembled and annotated in this plant group: *Ginkgo biloba* and *Welwitschia mirabilis* [44], *Cycas taitungensis* [45], *Cycas debaoensis* [46], and specifically in the conifers *L. sibirica* [3], *Taxus cuspidata* [30], *Picea glauca* [47], *Picea abies* [48], *Picea sitchensis* [49], and *Pinus taeda* [50]. Their mitogenome sizes range from 0.35 Mbp in *G. biloba* [44] to 11.66 Mbp in *L. sibirica* [3, 4].

Silver fir (*Abies alba* Mill.) is a frequent and widespread conifer with a core distribution in Central and Eastern Europe, with marginal populations in Southern Europe (Pyrenees, Italy, and Balkan Peninsula). It occupies a broad range of environmental conditions and possesses a deep root system that allows to access deep water under summer drought. It is therefore considered to better cope with future drought events under climate change than *P. abies* that often occupies similar habitats [51, 52]. In many countries of Europe, *A. alba* is an important tree species for wood production. In mountainous regions it also plays an essential role in protective forests.

The nuclear reference genome sequence of *A. alba* with an estimated size of 18.16 Gb was generated from DNA representing the adult tree AA_WSL01 (Birmensdorf, Switzerland) in a community-based effort of the Alpine Forest Genomics Network [53–55]. In the same study, the chloroplast genome of this genotype was assembled (120,908 bp) [54]. However, a complete mitogenome for *A. alba* has not been available so far.

In this study, the mitogenome of the *A. alba* reference individual AA_WSL01 was de novo assembled, annotated and compared to known mitogenome sequences of other gymnosperms and angiosperms. To do so, we used already available paired-end Illumina short reads [54] generated from a single megagametophyte, i.e. the haploid endosperm in conifer seeds [51], in combination with PacBio long reads from high molecular weight (HMW) DNA of needles, i.e. diploid tissue.

## Results

### Long-read sequencing by PacBio

In total, PacBio long-read sequencing produced 31.71 Gb of sequences. In the first run (three SMRT cells with sample Aa06), read length (mean ≈ 10 Kbp) was low despite a large number of total bases sequenced (Additional file 1: Table S1), pointing towards the presence of polymerase inhibitors in the DNA extraction that interfered with the sequencing. This problem was mostly solved in the second run (samples Aa18 and Aa20), for which a sorbitol washing step and other smaller changes were introduced to the HWM DNA extraction procedure (Additional file 2). Mean read length was around 14 and 17 Kbp, respectively (Additional file 1: Table S1).

### Short-read assembly and scaffolding by long reads

The cellular copy number of the mitogenome in the sequenced sample from a haploid megagametophyte was roughly estimated to be about 160 copies per cell. The initial short-read assembly resulted in a total contig length of 29 Mbp and represented mainly mitochondrial contigs and nuclear contigs of repeat regions. Based on (partial) CDS and tRNA/rRNA genes of other conifer species, 26 mitochondrial contigs were selected in a first step. Most of them were among the largest contigs in the initial assembly (Table 1). The mapping coverage of these 26 mitochondrial contigs was in a range of 32-139X. Additional mitochondrial contigs were selected based on mapping coverage in that range. In total, 47 mitochondrial contigs were selected (Table 1). Note that the total amount of PacBio data was too low to make a direct long-read assembly, which is not surprising given the nuclei isolation prior to HMW DNA extraction. Scaffolding using PacBio long reads resulted in 11 mitochondrial scaffolds with a total scaffold length of 1.43 Mbp and a GC content of 45.98% (Table 1).

**Table 1:** Summary of assembly statistics for mitochondrial contigs and scaffolds of *Abies alba*

Feature	Value
Number of contigs	47
Contig N50, bp	42,892
Length of the largest contig, bp	114,581
Number of scaffolds	11
Scaffold N50, bp	195,078
Length of the largest scaffold, bp	248,038
Total scaffold length, Mbp	1.43
GC content in scaffolds, %	45.98
Undetermined DNA bases (gaps) in scaffolds	None

### Structural diversity of the *A. alba* mitogenome

**Fig. 1:**
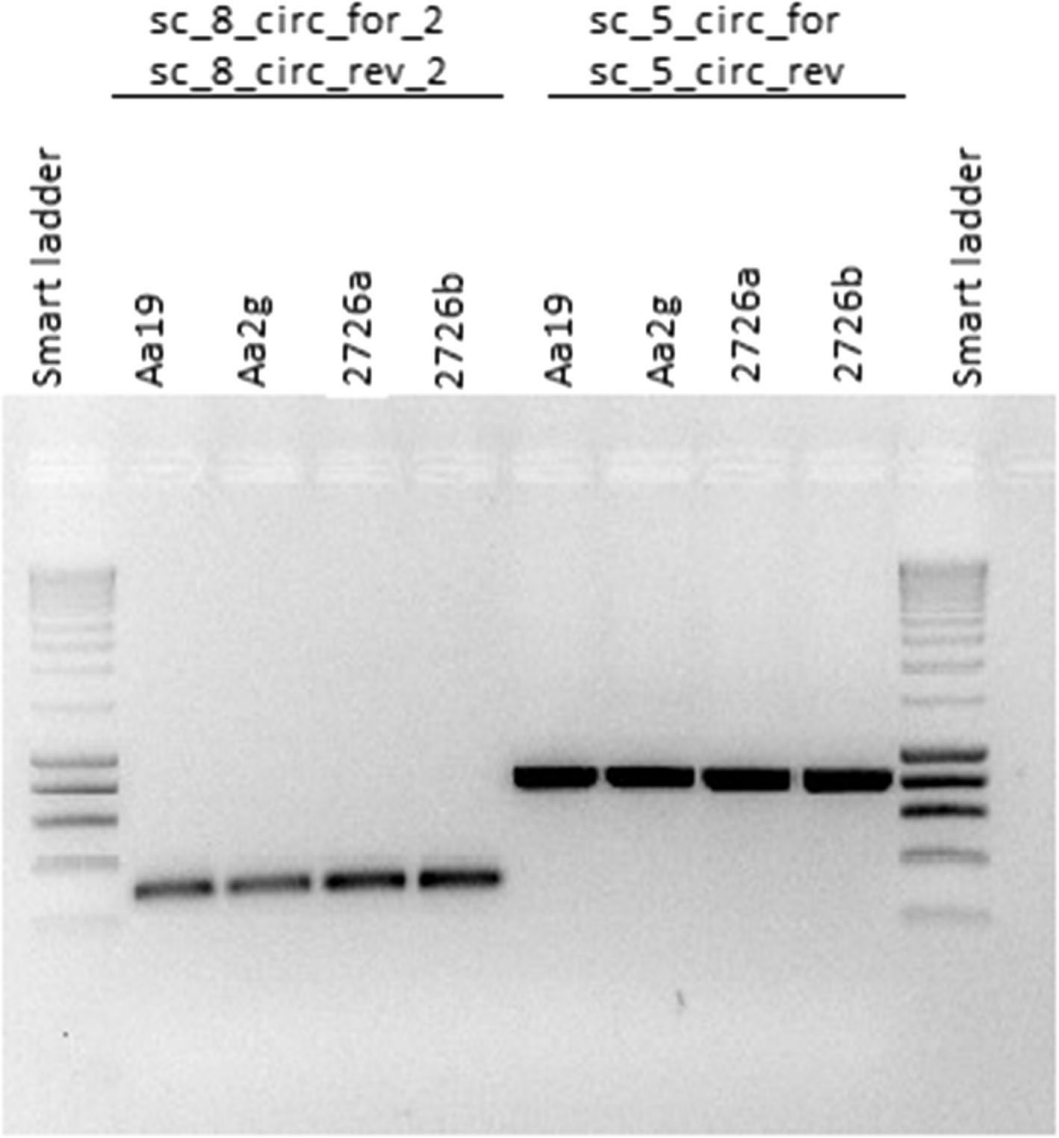
PCR fragments verifying circle closure prediction for two scaffolds sc_5_circ and sc_8_circ of *Abies alba*. Presence of a PCR amplification product of expected size indicates circular connection of two opposing ends of a given scaffold. Primer sequences are presented in Additional file 1: Table S2. Aa19 – HMW-DNA of the *Abies alba* reference tree AA_WSL01; Aa2g – total DNA of AA_WSL01; total DNA of two other *Abies alba* individuals, 2726a and 2726b

The 11 scaffold sequences of the *A. alba* mitogenome (GenBank ON378818—ON378828) most likely do not represent the physical structure of the mitogenome in vivo, which is expected to be highly complex, consisting of various alternative structures and substructures including subcircles. Two of the 11 scaffolds (Table 1) are expected to be of circular structure as also indicated by PCR results (Fig. 1).

**Fig. 2:**
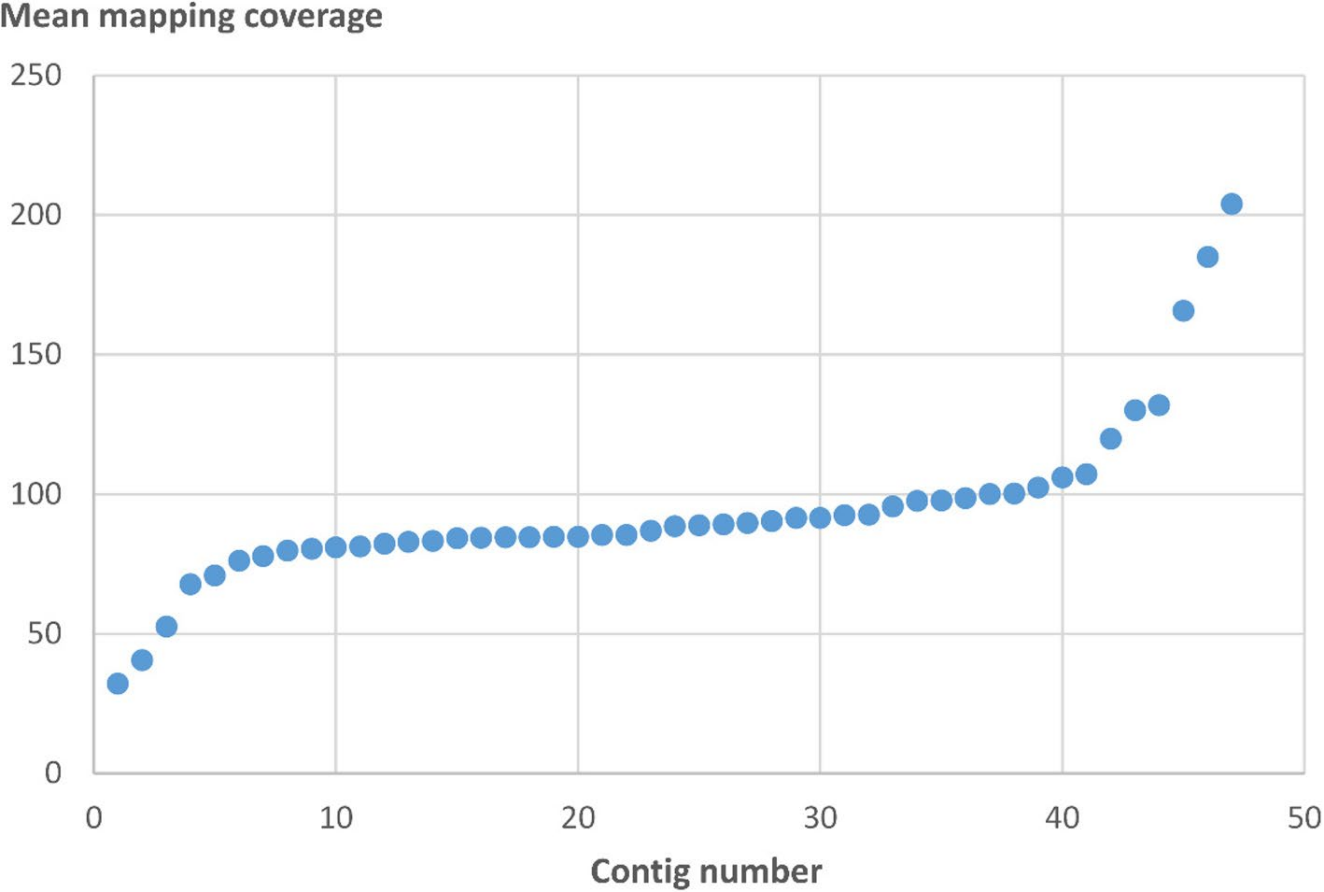
Mean mapping coverage of 47 original Illumina contigs included in the 11 scaffolds of the mitogenome of *Abies alba*. Contigs are sorted in ascending order of mean mapping coverage

We found a median mapping coverage of 89X (mean = 94X) in the 47 Illumina contigs included in the 11 scaffolds of the mitogenome of *A. alba* (Fig. 2). A majority of the 36 contigs had a coverage close to the median value (79-107X). Some contigs showed a lower coverage (32-71X) and may represent components of different low-copy subgenomic structures probably not present in each genome-containing mitochondrion. Other contigs show coverage values in a higher range (120-204X) corresponding to the 1.4- to 2.3-fold of the median coverage value including contigs with double coverage as well as contigs with intermediate coverage values (between single and double coverage, or double and triple coverage). These contigs are components of different types of repeat regions which may be present in different copy number values. The presence of contigs with intermediate coverage between double and triple coverage, e.g., may point to two-copy repeats in the entire mitogenome which may also exist in a single-copy version in some alternative substructures not present in each genome-containing mitochondrion.

**Fig. 3:**
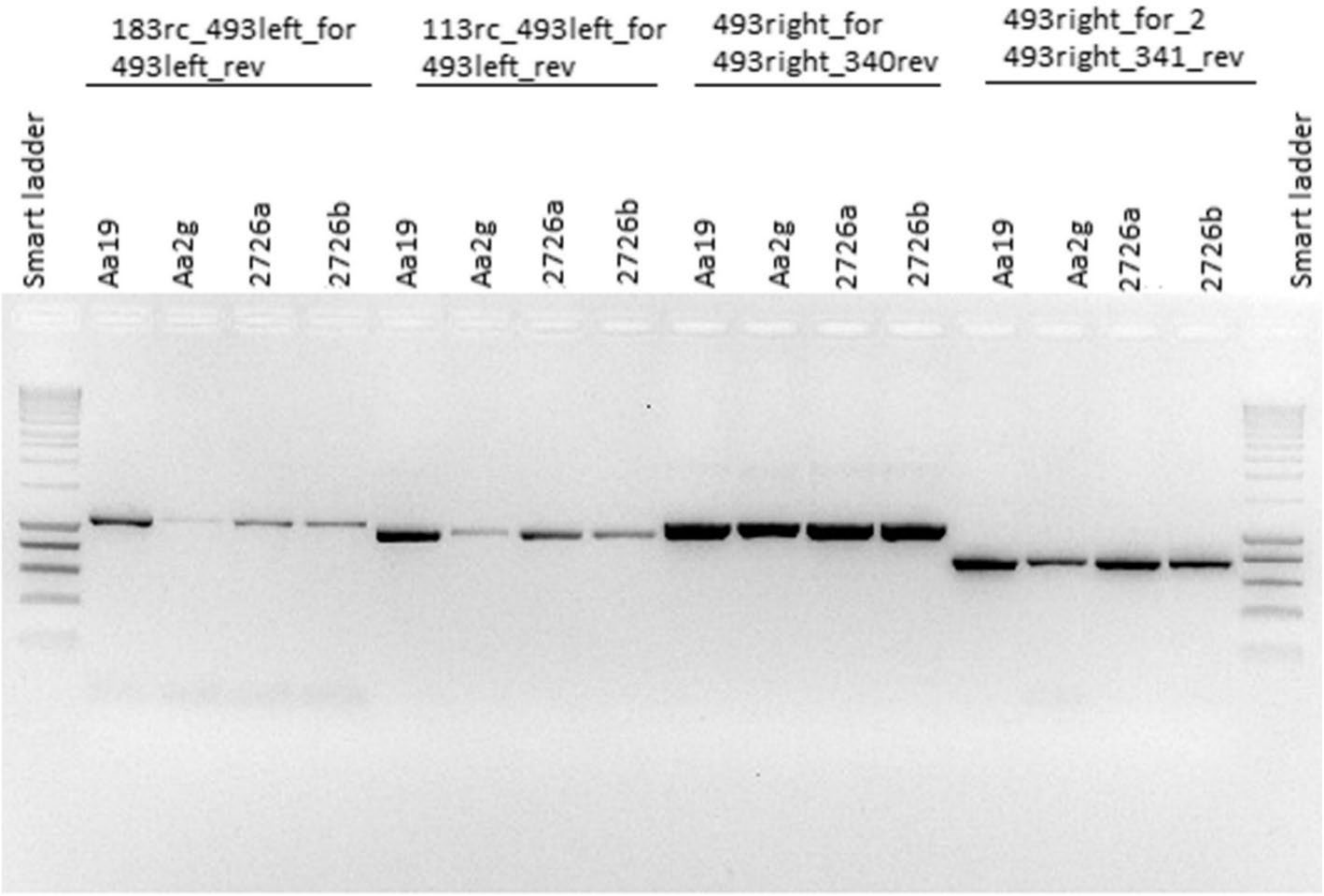
PCR fragments confirming different alternative connections of contig 493 to other four contigs 183, 113, 340, and 341 in different scaffolds of the *Abies alba* mitogenome. Primer sequences are presented in Additional file 1: Table S2. Aa19 – HMW-DNA of the *Abies alba* reference tree AA_WSL01; Aa2g – total DNA of AA_WSL01; total DNA of two other *Abies alba* individuals, 2726a and 2726b

Contig 493 with the highest mean mapping coverage of 204X (Fig. 2) was assigned to four different scaffolds (2, 3, 4, and 9) based on potential bilateral connections to other different contigs supported by long reads. These different alternative connections of contig 493 to contigs 183, 113, 340, and 341 were confirmed by PCR in the reference individual as well as in the two other *A. alba* individuals 2726a and 2726b (Fig. 3).

The data presented in Figs. 1,2, and 3 suggest a highly dynamic and variable structure of the *A. alba* mitogenome probably mediated by repeats described in the next section.

### Genome annotation

**Fig. 4:**
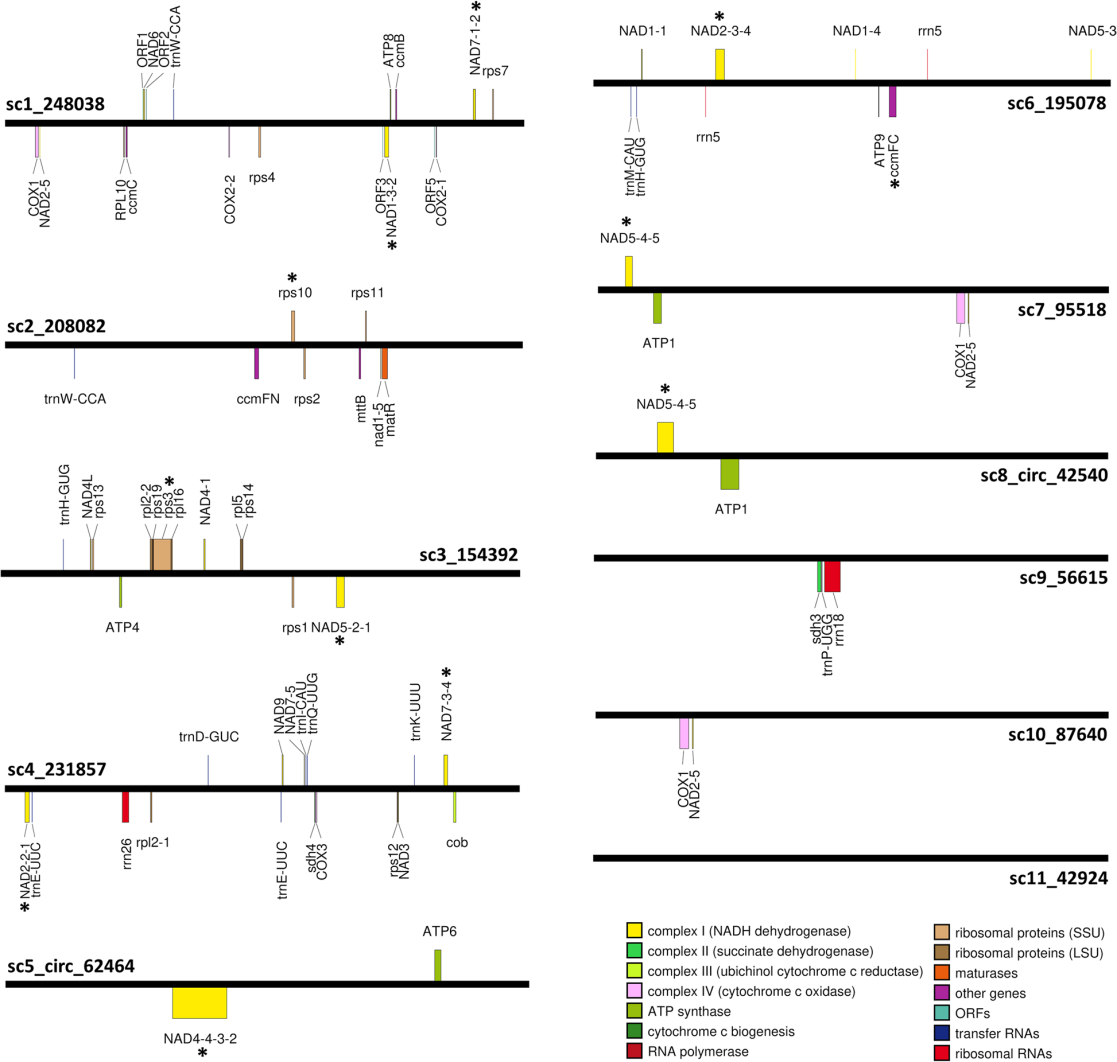
Linear graphical display of the genetic maps of 11 scaffolds in the assembly of the *Abies alba* mitogenome. The display most likely does not represent the physical structure of the mitogenome in vivo. Scaffold names are indicated, e.g., as “sc1_248038”, where “sc1” represents “scaffold 1” and “248,038” corresponds to the length of the scaffold in bp; “circ” indicates scaffolds with potential circular structure. The length of the black bars is not proportional to the length of the scaffolds. Numbers after gene names refer to one or more exons. Potential CDS regions of unknown function are indicated as “ORFs”. The map was created with OrganellarGenomeDraw [56, 57] using a linear map stretch factor of 1/4. * genes/gene fragments with introns

In the 11 scaffolds of the mitogenome assembly of *A. alba,* 53 distinct genes with predicted functions were found and annotated, including 41 protein-coding genes, nine tRNA and three rRNA genes (Fig. 4). Some genes (gene fragments) are located in repeat regions. The 41 protein-coding genes comprise the following genes: *atp1, atp4, atp6, atp8, atp9, ccmB, ccmC, ccmFc, ccmFn, cob, cox1, cox2, cox3, matR, mttB, nad1, nad2, nad3, nad4, nad4L, nad5, nad6, nad7, nad9, rpl2, rpl5, rpl10, rpl16, rps1, rps2, rps3, rps4, rps7, rps10, rps11, rps12, rps13, rps14, rps19, sdh3,* and *sdh4*. These genes include all known genes coding for subunits of proteins of the respiratory chain and 11 genes coding for small and four genes for large subunits of ribosomal proteins, beside other genes.

The *A. alba* mtDNA encodes 5S, 18S, and 26S rRNAs. The nine distinct tRNA genes with eight different anticodons comprise the following: *trnD-GUC*, *trnE-UUC*, *trnH-GUG*, *trnI-CAU*, *trnK-UUU*, *trnM-CAU*, *trnP-UGG*, *trnQ-UUG*, and *trnW-CCA* (Fig. 4). The nucleotide sequence of the gene *trnI-CAU*, predicted on *A. alba* scaffold 4, shows 100% nucleotide identity to potato *trnI-CAU* [58]. The C-residue in the anticodon of *trnI-CAU* is inferred to be post-transcriptionally modified to a lysidine-like nucleotide which pairs with A, not G [58]. Because the tRNA gene *trnY-GUA* was not annotated in the *A. alba* mitogenome but in four other gymnosperm species (see summary of tRNA genes in gymnosperm mitogenomes [49]), we performed additional BlastN analyses to check the Illumina contigs as well as the raw data (Illumina and PacBio reads) for a potential presence of this tRNA gene; however, no relevant hits were identified (Additional file 3).

Exons of the following genes were annotated on at least two different scaffolds: *nad1*, *nad2*, *nad4*, *nad5*, *nad7*, and *rpl2*. The two exons of *cox2* were annotated on scaffold 1 far apart from each other (98,582 bp in-between). The putative start codons of *atp6*, *cox1*, *mttB*, *nad1*, *nad6*, *nad9*, *rpl16*, *rps3*, and *rps19*, as well as the putative stop codons of *nad4*, *nad4L*, *rpl2,* and *sdh3* are potentially created by RNA editing. In addition, four potential protein-encoding genes of unknown function were annotated on scaffold 1 based on ORF prediction (Fig. 4). In BlastP analyses versus nonredundant proteins at NCBI GenBank [59], the predicted translated ORF1 showed 100% identity and 41% query coverage to two hypothetical proteins predicted to be expressed by two ORFs in the mitogenomes of *P. sitchensis* (QHR91286.1) and *P. glauca* (KUM50578.1), whereas the other three ORFs did not provide results. BlastN analysis of ORF sequences resulted in top hits to mitochondrial scaffolds from *A. firma*/*A. sibirica* (ORF1: MW354087.1/MN965103.1; ORF2: MW354087.1; ORF3: MW354090.1/ MN965092.1; ORF5: MW354090.1/MN965098.1).

MTPTs with more than 90% similarity to the *A. alba* chloroplast genome sequence (NC_042410) [60] accounted for only 0.12% of the mitogenome and are distributed among seven distinct regions in the genome (Additional file 1: Table S3). Chloroplast genes included in these regions comprise *trnW-CCA* as well as fragments of *rpoB*, *rrn16* and *rrn23*.

All MTPTs except hit 1 were also confirmed in contigs of an alternative short-read assembly including chloroplast DNA (cpDNA) reads (Additional file 1: Table S3). The cpDNA-query region of hit_1 (rrn23_partial) was only present in a cpDNA contig of this assembly. No additional MTPTs were identified in the contigs of this alternative assembly compared to the scaffolds of the *A. alba* mitogenome. Although one putative mtDNA-derived contig of the alternative short-read assembly (contig_885; coverage value of 72X) provided a short hit to the *A. alba* cpDNA sequence (hit length of 55 bp; identity of 94%), it turned out to be of nuclear DNA origin because BlastN analysis of the most similar PacBio read (read m54273_190620_131850_58982525_0_36806; 36 806 bp length; ENA project PRJEB52007) provided no hit to mtDNA-derived sequences at NCBI but resulted in several BlastN hits to *A. alba* scaffolds of the genome assembly [54] (top-3 hits: aalba5_s00427688, aalba5_s00660298, aalba5_s00225537). Hence, we conclude that our assembly strategy, a priori excluding Illumina short reads representing plastome, successfully retrieved the complete mitogenome of *A. alba,* including MTPTs.

The proportion of repetitive elements (REs) in the mitogenome of *A. alba* was 0.168 according to the analysis using the ROUSFinde1_1 tool [16]. In total, 1,201 different REs ≥ 24 bp were identified (Additional file 4). These REs included 183 different interspersed REs ≥ 50 bp with an average size of 1,862 bp and an average copy number of 2.7 per RE (Additional file 1: Table S4). Most of these REs were in the size range between 50 and 200 bp, with nine REs even above 10,000 bp (Additional file 5).

### Size and proportion of repetitive elements (REs) in the *A. alba* mitogenome

**Fig. 5:**
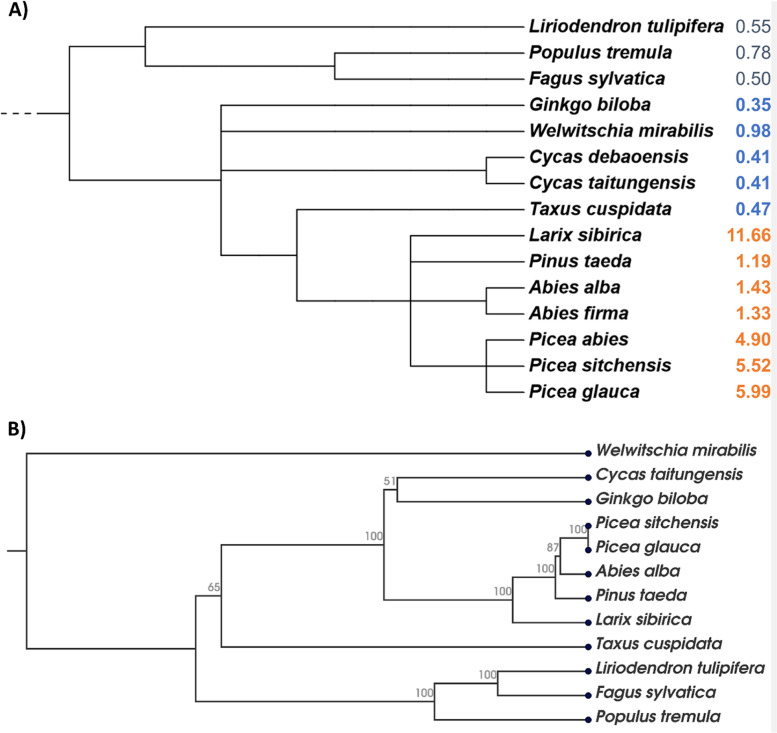
Phylogenetic trees of **A** 12 gymnosperm species with fully assembled mitogenomes and three angiosperm species (outgroups) based on NCBI taxonomy and **B** 9 gymnosperm species with annotated mitogenomes (same outgroups) based on CDS of conserved mitochondrial genes. The mitogenome assembly of *Abies firma* represents a draft assembly [61]. Mitogenome sizes are presented in Mbp (sizes of member species of the order Pinales highlighted in orange, and of other gymnosperms in blue). For the taxonomy-based tree generated using phyloT v2 [65], bootstrap scores cannot be provided. The CDS-based tree was created using the UPGMA-method based on an alignment of CDS of 17 genes. See Table 2 for accession numbers of related mitogenomes, number of scaffolds, and respective references

The *A. alba* mitogenome size of 1.43 Mbp estimated based on the current genome assembly is very close to the total scaffold length of 1.33 Mbp of a mitogenome draft assembly of *A. firma* [61] (Table 2). The GC content of both mitogenomes is also very similar: 45.98% in *A. alba* (Table 1) *vs* 45.78% in *A. firma* [61]. The estimated mitogenome sizes of the two *Abies* species were compared with other gymnosperm species and three angiosperm species (outgroups) in the context of a phylogenetic tree constructed simply based on NCBI taxonomy (Fig. 5A). For comparison, a phylogenetic tree was created based on an alignment of the coding sequences of 17 conserved mitochondrial genes of the same species (except species without available mitogenome annotation) placing *Welwitschia mirabilis* as outgroup (Fig. 5B), largely confirming the taxonomy-based tree presented in Fig. 5A. Mitogenomes of all Pinales species analysed so far including *A. alba* and *A. firma* show sizes above 1 Mbp, whereas the other gymnosperm species show mitogenome sizes below 1 Mbp (Fig. 5A). Mitogenome sizes of two species of the genus *Cycas* and three species of the genus *Picea* are each in the same range.

**Table 2:** Mitogenomes of species used for annotation, size comparisons and/or analyses of repetitive elements in *Abies alba*, with related accession numbers at NCBI GenBank or CNSA of China National GenBank DataBase (*) and references

Species	NCBI GenBank accession number	Number of scaffolds	Total size, Mbp	Reference
Gymnosperms				
* Abies alba*	ON378818- > ON378828	11	1.43	This study
* Abies firma*		n.d	1.33	[61]
* Cycas debaoensis*	CNA0019277*	1	0.41	[46]
* Cycas taitungensis*	NC_010303.1	1	0.41	[45]
* Ginkgo biloba*	NC_027976.1	1	0.35	[44]
* Larix sibirica*	MT797187.1- > MT797195.1	9	11.66	[3]
* Picea abies*	MN642623- > MN642626	4	4.90	[48]
* Picea glauca*	LKAM01000001- > LKAM01000036	36	5.99	[47]
* Picea sitchensis*	MK697696.1- > MK697708.1	13	5.52	[49]
* Pinus taeda*	NC_039746.1	1	1.19	[50]
* Taxus cuspidata*	MN593023.1	1	0.47	[30]
* Welwitschia mirabilis*	NC_029130.1	1	0.98	[44]
Angiosperms				
* Arabidopsis thaliana*	NC_037304.1	1	0.37	[62]
* Fagus sylvatica*	NC_050960.1	1	0.50	[63]
* Liriodendron tulipifera*	NC_021152.1	1	0.55	[13]
* Populus tremula*	NC_028096	1	0.78	[64]

The mitogenome size of *Abies firma* was estimated based on a draft assembly [61]. n.d., no data available

**Fig. 6:**
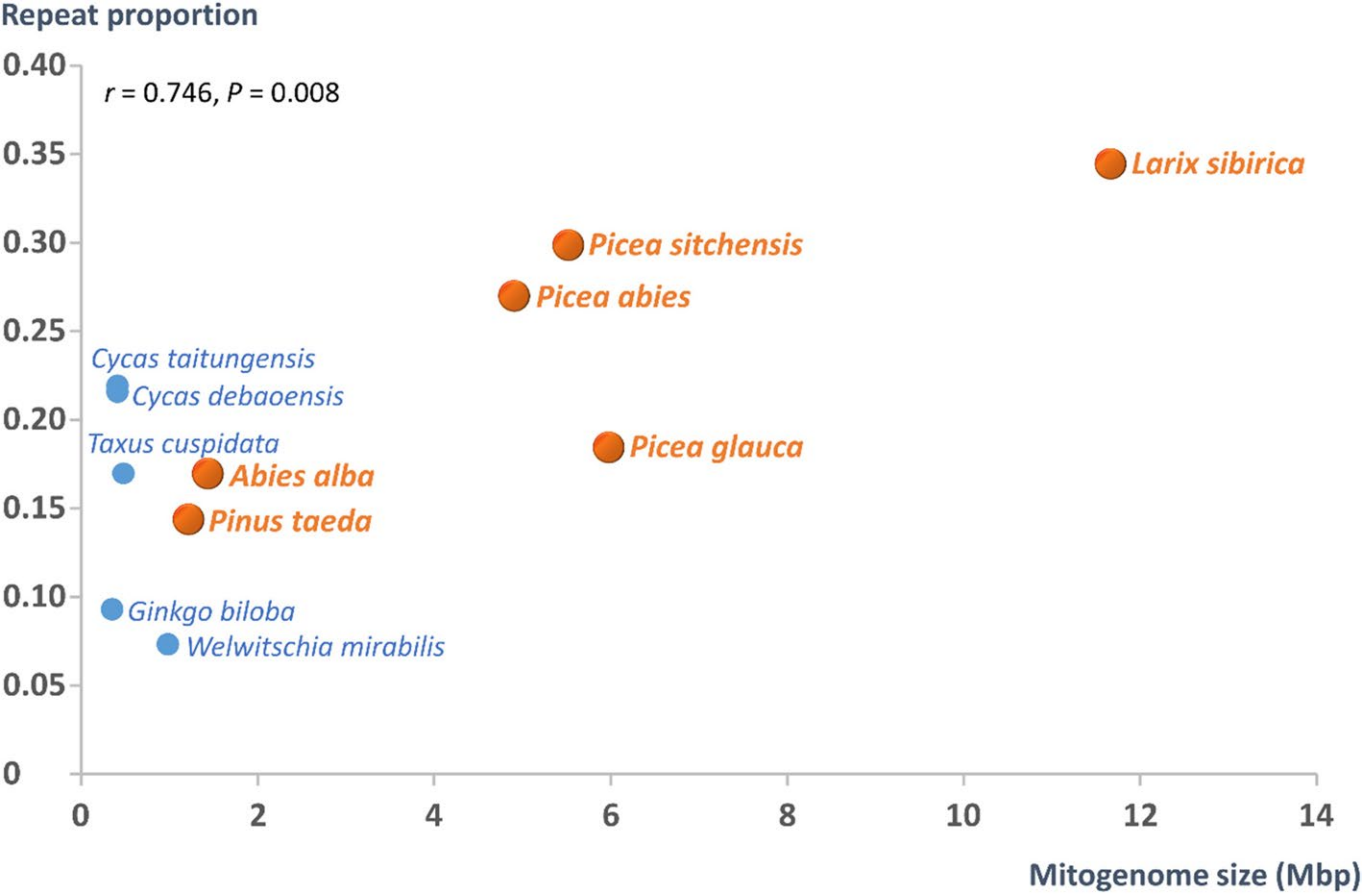
Repeat proportion vs mitogenome size in 11 gymnosperm species (values are presented in Additional file 1: Table S5). Member species of the order Pinales are highlighted in orange, and other gymnosperms in blue. *r*—Pearson correlation coefficient with its respective *P* value

The proportion of REs in assembled mitogenome sequences of gymnosperm species positively and significantly correlated with mitogenome sizes (Pearson’s *r* = 0.746; *P* = 0.008; Fig. 6). This relationship demonstrates a trend for gymnosperm mitogenomes with large genome sizes to also contain high proportions of REs (and *vice versa*).

## Discussion

In this study, we used short and newly generated long reads from PacBio sequencing to assemble the mito-genome sequence of *A. alba*, an economically important gymnosperm species of the Pinaceae family in the Pinales order (clade Conifers I). The *A. alba* mitogenome sequence consisting of 11 scaffolds (Fig. 4) extends the still small database of fully sequenced Pinales mito-genomes to six assemblies, which all show sizes above 1 Mbp (Fig. 5). The estimated size of the mitogenome of *A. alba* and its GC content (Fig. 5, Table 1) – based on the presented assembly – are very similar compared to those of another member of the genus, *A. firma* [61]. The proportion of REs estimated at 0.17 in the *A. alba* mitogenome is within the range of other sequenced Pinales mitogenomes (0.14–0.34; Fig. 6).

The set of 41 distinct protein coding genes of known function in the mitogenome of *A. alba* (Fig. 4) is consistent with related sets in *P. sitchensis* [49] and *P. taeda* (MF991879.1) – other member species in the Pinales – as well as in *G. biloba* [44] and *C. taitungensis* (AP009381.1; [45, 65]). In contrast, the mitogenomes of *T. cuspidata* and *W. mirabilis* have individually lost/transferred some of these genes, comprising genes coding for small or large subunits of ribosomal proteins and *sdh3* [30, 44, 61, 66]. Interestingly, a broader requirement for mitochondrial trans-splicing – the joining of exons from distinct transcripts – was discovered in gymnosperms compared to angiosperms [66]. These authors reported that trans-splicing affected 13 Pinaceae introns within the genes *cox2*, *nad1*, *nad2*, *nad4*, *nad5*, *nad7*, and *rpl2.* Exons of these genes were annotated in *A. alba* on different scaffolds or – as in case of *cox2* – far apart from each other on the same scaffold (Fig. 4).

The discovery of three distinct rRNA genes in the mitogenome of *A. alba* (Fig. 4) is consistent with the rRNA genes in other gymnosperms (recently summarised in [49]). The tRNA gene content of mitochondrial origin in mitogenomes of streptophytic green algae and land plants shows a high dynamic range [67]. Gymnosperm mitogenomes also show a high variation in the number of predicted tRNA genes. Whereas, only six tRNA genes were annotated in *T. cuspidata* [30] and only eight in *W. mirabilis*, more genes were predicted in *G. biloba*, *C. taitungensis*, and *P. sitchensis* (see summary of tRNA genes in gymnosperm mitogenomes in [49]). The common tRNA gene content in the mitogenomes of the above mentioned five gymnosperm species comprises only the following five genes: *trnD-GUC*, *trnE-UUC*, *trnM-CAU*, *trnW-CCA* and *trnY-GUA*. All of these genes except *trnY-GUA* were also annotated in the *A. alba* mitogenome, beside five other tRNA genes previously predicted also in other gymnosperm mitogenomes [49]. A deeper sequence analysis considering both the *A. alba* Illumina and PacBio reads (Additional file 3) also did not provide any indication of the presence of *trnY-GUA* in the mitogenome of *A. alba*. Thus, we assume a loss of this gene in the mitogenome of *A. alba*. In general, plant mitogenomes feature high evolutionary dynamics in their tRNA gene content due to ongoing tRNA gene losses/gains, transfers and functional replacements [67]. The gene *trnP-AGG*—predicted in *G. biloba*, *C. taitungensis, P. sitchensis* [49]—was not identified in *A. alba, T. cuspidata* [30] and *W. mirabilis* [44]. This is not unexpected because this gene has not been described as part of the tRNA gene content of plant mitogenomes in general [67].

Recent publications provided evidence that plant mitogenomes exist as a complex and dynamic collection of linear DNA with combinations of smaller circular, concatenated and branched DNA molecules [11, 21]. For example, the assembly graph of the mitogenome of *P. sitchensis* shows a multipartite genome structure, composed of one smaller circular segment of DNA and a larger component with a branching structure [49]. Two of the *A. alba* mitogenome scaffolds assembled in our study also display a potential circular structure (Fig. 4) as indicated by PCR results (Fig. 1). To further confirm these subgenomic circles, ultra-long reads similar to the size of these circles are needed in future studies.

The mapping coverage analysis of mitochondrial contigs from the initial short-read assembly in our study reveals that, beside many contigs showing a coverage close to the median value, there are some contigs with a lower or intermediate coverage between one- and three-fold of the median coverage (Fig. 2). These results suggest a highly dynamic and variable structure of the *A. alba* mitogenome featured by combinatorial variation, which was specifically confirmed by PCR for the mitochondrial contig with the highest mapping coverage (Fig. 3). The results are in line with some reports on other species in the Pinales that show mitogenomes of complex and dynamic physical structure [3, 49] as well as with recent findings that different mtDNA-containing mitochondria of a plant individual may contain different portions of the mitogenome (different subgenomic structures or the entire mitogenome) [11]. The coexistence of genetically distinct mitochondrial genomes within an individual is known as heteroplasmy, which can occur via the coexistence of mitochondrial genomes of either different nucleotide lengths (length heteroplasmy) or different nucleotide compositions (site heteroplasmy) [68]. Reports of heteroplasmy caused by paternal leakage, e.g. in [69], are rare in plants [68]. One source of heteroplasmy that is known only from higher plants is the presence of mitochondrial genes on subgenomic molecules occurring at very low (‘substoichiometric’) levels in cells [68] due to frequent inter- and intramolecular recombination in areas of repeated sequences [70]. Especially large non-tandem repeats may act as recombination sites [16]. For example, a pair of direct repeats had mediated mitogenome recombination in the angiosperm *Scutellaria tsinyunensis*, resulting in the formation of two conformations confirmed by Oxford Nanopore sequencing [71].

So far, complete mitogenomes of several gymnosperm species were assembled based on whole-genome sequencing data obtained from total DNA [30, 47–50], enriched mtDNA [44, 45] or a combination of both as in the case of *L. sibirica* [3]. However, as reported in the *L. sibirica* study, enrichment of mtDNA did not allow to obtain HMW mtDNA needed for long-read sequencing. In the present study, we used published short Illumina reads [54] from total DNA prepared from a single haploid megagametophyte in combination with newly generated long reads from HMW DNA obtained after nuclei isolation from diploid needle tissue of the same individual. The comparably high cellular copy number of the mitogenome in the Illumina input data from the megagametophyte – roughly estimated to be about 160 copies per cell – supported the separation of mitochondrial contigs from nuclear contigs based on depth of coverage (after the initial assembly). In other tissues and species, cellular copy numbers of the mitogenome were estimated to be only 38–40 or 32–34 copies per cell when analysed from whole-genome sequencing data of leaf samples of deciduous trees or needle samples of conifers, respectively [72]. In general, cellular copy numbers of the mitogenome seem to show high variation among organs or during development, as elucidated by analysing several individual mitochondrial genes using real-time quantitative PCR in various samples of *Arabidopsis thaliana*, *Nicotiana tabacum* and *Hordeum vulgare* [73, 74] or by droplet digital PCR (ddPCR) in *Cucumis melo* [75]. There are also reports that not each mitochondrion contains mtDNA copies when comparing the copy number of individual mitochondrial genes with the mean number of mitochondria per cell in plant tissues [73, 75]. It is important to note that the DNA extraction method may play an important role for the level of observed variation in mtDNA copy numbers, as has been proven in humans [76].

## Conclusions

The mitogenome sequence of *A. alba* opens new possibilities for comparative studies and will allow to answer important structural, phylogenetic and other evolutionary questions. Future long-read sequencing in higher coverage of the *A. alba* mitogenome will be the key to provide a refined size estimation and to further resolve the physical structure of the mitogenome including alternative structures and substructures. The database of gymnosperm mitogenomes has to be extended to validate the observed positive correlation between mitogenome size and proportion of REs in the future. Whether higher RE proportions in mitogenomes are correlated with higher recombination tendencies, resulting in higher structural complexity and dynamics and increased heteroplasmy, is an interesting open question for future research.

## Methods

### High-molecular weight DNA extraction and PacBio long-read sequencing

Fresh needle tissue for HMW DNA extraction was sampled from the reference tree AA_WSL01 [54] (collection source of specimen sample: adult silver fir tree AA_WSL01 in a public forest next to the WSL institute in Birmensdorf, Switzerland; tree location: 47.3624°N, 8.4536°E; collected by Gabor Reiss, WSL; permission for collection was obtained from the local forest service; formal species identification by the WSL team as described in [54], appendix 2; voucher specimen: WSL-accession AA_WSL01, WSL Birmensdorf, Switzerland) in spring 2017 and immediately stored at –80 °C until further use. HMW DNA extraction was performed following a modified protocol of Workman et al. [77] using the Nanobind Plant Nuclei Big DNA Kit (Circulomics, Baltimore, MD, USA); see Additional file 2 for the detailed protocols and DNA quantity/quality parameters. We finally selected three DNA extracts (Aa06, Aa18, Aa20) for library preparation, which was performed by the Genomics Facility of ETH Basel (BSSE) using SMRTbell Template Preparation Kit 1 (Pacific Biosciences, Menlo Park, CA, USA) and a BluePippin size selection instrument (Sage Science, Beverly, MA, USA). The samples were run on five SMRT cells on a PacBio Sequel system (Pacific Biosciences, Menlo Park, CA, USA); the first run with three cells all containing Aa06 (but different loading concentrations), and the second run with one cell each for Aa18 and Aa20. Note that the HMW DNA was extracted supposedly from nuclei. However, it can be expected that the resulting libraries still contain a considerable amount of mtDNA, and that their reads can be used for the assembly of the mitogenome, which was the case for the data presented here.

### Assembly of short reads

For the initial short-read assembly, we used the following data sets of paired-end Illumina reads from the *A. alba* reference tree AA_WSL01, generated by HiSeq 4000 sequencer (Illumina Inc., San Diego, CA, USA) in the genome sequencing study [54], available at NCBI BioProject accession number PRJEB35555 and SRA accession numbers ERR3686456 from library PE300-2.6 and ERR3686466 from library PE300-3.7. These short reads (2 × 150 bp read length) originated from total DNA isolated from a single haploid megagametophyte of the reference tree [54]. Reads were reduced to about 25% of the original data amount using the “sample reads” tool of CLC Genomics Workbench (CLC-GWB) v21 (QIAGEN, Hilden, Germany; sample percentage = 25, sample type = random) for efficiency reasons and then trimmed using the “trim reads” tool of CLC-GWB with the following parameters: adapter trimming = yes, quality limit = 0.01, maximum number of ambiguities = 0, number of 3’/5’-terminal nucleotides to be removed = 1, minimum length = 149 bp. Potential chloroplast-derived reads were removed from the trimmed reads by mapping them to the *A. alba* chloroplast genome NC_042410 [60] using CLC-GWB “map reads to reference” tool with default parameters, but with a length fraction of 0.9 and a similarity fraction of 0.95, followed by collection of unmapped reads. The unmapped reads were considered as chloroplast-free trimmed short reads for further analysis. These reads were subjected to an assembly using CLC-GWB “de novo assembly” tool with default parameters, but with minimum contig length = 500 bp, and auto-detect paired distances = yes.

### Selection of mitochondrial contigs

All contigs fulfilling one of the following two criteria were selected as mitochondrial contigs: (1) contigs including complete or partial mitochondrial CDS, rRNA genes and/or tRNA genes identified based on BlastN analyses using related sequences from other mitogenomes as queries (*P. taeda*, NC_039746; *L. sibirica*, MT797187-MT797195), (2) contigs ≥ 2,000 bp with a mapping coverage in the range of all mitochondrial contigs selected based on the first criteria (32X to 139X).

To identify the mapping coverage of contigs, sampled trimmed short reads were mapped to the contigs using the “map reads to reference” tool of CLC-GWB with default parameters, but with a length fraction of 0.9 and a similarity fraction of 0.95, before recording mean mapping coverage of the contigs.

### Scaffolding using PacBio long reads

All mitochondrial contigs were sorted by descending size. The largest mitochondrial contig was used as the first seed to start building scaffold 1. For further seed extension by other mitochondrial contigs, a stepwise strategy was applied in which long reads were used as framework. For extension, the seed contig was analysed by BlastN (CLC-GWB; “blast” tool; default parameters) *vs* all long reads ≥ 10 Kbp. Long reads with best BlastN hits of at least 90% identity showing a complete overlap to the entire seed contig or a partial overlap of at least 10 Kbp to the 5’- or 3’-prime ends were selected and analysed by BlastN (default parameters; 90% identity in overlapping regions) *vs* all Illumina contigs to define the adjacent contigs in both directions (if possible). Connections were only built manually if at least three long reads supported each connection. Extension stopped if a clear connection could no longer be found (i.e., analysed long reads supported different adjacent mitochondrial contigs – often in case of repeat contigs – or further extension was not possible at all). If an extension of scaffold 1 was no longer possible, the second longest remaining contig was used as the next seed to build scaffold 2. The extension of the second seed by mitochondrial contigs was performed as described above. Then, the third longest remaining contig was used as next seed, and so on. However, contigs with repeat features (identified based on coverage; see Results) were included in two or more different scaffolds as guided by the results of the Blast analyses *vs* the long reads (described above).

### Polishing of scaffold sequences

N-regions in the scaffold sequences originating from N-stretches in Illumina contigs or MTPTs were manually replaced by the related sequence of the most similar PacBio read showing full coverage to the related mitogenome region. The PacBio read-derived scaffold regions were then polished by the following strategy: Trimmed Illumina reads were mapped *vs* the scaffold sequences and the chloroplast genome using the “map reads to reference” tool of CLC-GWB with default parameters, but with a length fraction of 0.9 and a similarity fraction of 0.9. After mapping, the consensus sequence was extracted using the “extract consensus sequence” tool of CLC-GWB with default parameters, but with a threshold of 5 for “low coverage definition”. A second more stringent mapping step was performed as described above, but with a length fraction of 0.9 and a similarity fraction of 0.95, followed by consensus extraction (see above). Original scaffold sequences were then refined in PacBio-read derived regions by manual replacement of the PacBio-sequence stretches by the related polished sequence stretches. For a final polishing of all scaffold sequences, trimmed Illumina reads were mapped *vs* the scaffold sequences and the chloroplast genome (default parameters, but with a length fraction of 0.9 and a similarity fraction of 0.95) followed by consensus sequence extraction as described above.

### PCR-based validation of potential circular scaffold structures and alternative contig connections

To validate a potential circular structure of a scaffold, the PCR primers were designed (Additional file 1: Table S2) based on priming sites located in the distal parts of the scaffold, so that the amplified fragment could connect these distal ends and encircle contigs. Alternative connections of contig 493 to other contigs in different scaffolds of the *A. alba* mitogenome assembly were validated by designing primers located in the distal part of contig 493 and the distal part of the related adjacent contig. Fragments were amplified by PCR using four different *A. alba* samples: (i) a HMW DNA preparation from needles of the *A. alba* reference tree AA_WSL01 (Aa19; Additional file 2), (ii) a total DNA extract of the same individual prepared from needles (needles for (i) and (ii) collected by Gabor Reiss, WSL; permission for collection was obtained from the local forest service) with a modified ATMAB standard protocol [78] using an additional phenol–chloroform cleaning step (Aa2g), (iii) total DNA extractions from needles (collection and species identification by Hilke Schroeder, Thünen Institute of Forest Genetics; permission for collection was obtained from Thünen Institute of Forest Genetics) of two other *A. alba* individuals grown in the Arboretum of the Thünen Institute of Forest Genetics (Grosshansdorf, Germany; Arboretum-IDs: 2726a and 2726b; original provenance: Central/South European mountains).

PCR amplification was performed in 20 µl volume with 10 ng DNA. For all primers (Additional file 1: Table S2), the reaction mixture contained 1 × BD buffer, 2 mM MgCl_2_, 0.2 mM of each dNTP, 1 × DMSO (NEB), 0.1 µM of each primer and 1 Unit *Taq* DNA polymerase (DCS Pol, DNA Cloning Service, Hamburg, Germany). The PCR was performed with the following program: 95 °C for 3 min, followed by 40 circles with 95 °C for 20 s, 57 °C for 30 s, 72 °C for 90 s and a final extension with additional 10 min at 72 °C.

### Annotation of the *A. alba* mitogenome

Draft structural and functional annotation of the assembled mitogenome scaffolds was done using the GeSeq server [79] with default settings except keeping best annotation only; BLAST search by default; 3rd party tRNA annotation yes: 1. use Aragorn v1.2.38; genetic code: yeast mitochondrial; max. intron length: 3,000 bp; 2. tRNA scan-SE v2.0.7 with sequence source: organellar tRNAs. The mitogenomes of *P. taeda* and *Fagus sylvatica* (Table 1) were used as references. Using the Sequin tool v13.05 [80], draft annotations were corrected where necessary (especially for potential protein-coding genes), guided by alignments to other well-characterized mtDNA sequences, including those of *Arabidopsis thaliana*, *G. biloba* and *L. tulipifera* (Table 2).

### Identification of MTPTs in the *A. alba* mitogenome

MTPTs with more than 90% similarity to the *A. alba* chloroplast genome sequence (NC_042410) [60] and hits of length above 50 bp were identified by BlastN alignment of the *A. alba* chloroplast sequence *vs* the *A. alba* mitochondrial scaffolds using the NCBI BlastN tool [59].

MTPTs were also identified in an alternative short read-assembly based on the trimmed Illumina reads, but including cpDNA reads and using CLC-GWB with the same assembly parameters as described above for Illumina reads after removal of cpDNA reads. All contigs of this assembly were analysed by BlastN *vs* the *A. alba* chloroplast genome sequence (NC_042410) [60] and hits of length above 50 bp with more than 90% similarity to the subject were further considered if the related query contig was of potential mtDNA origin as indicated by a related coverage value (see above).

### Building of phylogenetic trees and identification of repetitive elements (REs)

A phylogenetic tree of 12 gymnosperm and three angiosperm species (outgroups) with completely sequenced mitogenomes (Fig. 5A; accession numbers in Table 2) was built using the tree generator tool phyloT v2 [65] that is simply based on NCBI taxonomy [81]; thus, bootstrap scores cannot be provided. To build a phylogenetic tree (Fig. 5B) based on an alignment of CDS of 17 conserved protein-coding genes from all mitogenomes presented in Fig. 5A with available annotation (except *A. firma*, *Cycas debaoensis*, and *Picea abies*), the related annotated mitochondrial genome sequences were downloaded from GenBank using CLC-GWB v22.0.2 (“search for sequences at NCBI”-tool). All CDS sequences were extracted using the “extract annotated regions”-tool selecting CDS as “annotation types”. For each species, the CDS of the following genes were concatenated using the “join sequences”-tool: *atp1*, *cox1*, *atp8*, *cob*, *cox3*, *ccmC*, *ccmB*, *rps4*, *nad9*, *atp4*, *atp6*, *atp9*, *ccmFn*, *nad3*, *nad6*, *mttB*, and *nad4L*. The combined CDS of the 12 species were aligned using the “create alignment” tool with default parameters. Then, a phylogenetic tree (Fig. 5B) was constructed using the “create tree”-tool with default parameters, but selecting UPGMA as “tree construction method”.

Accession numbers and related references of other mitogenomes used for size comparisons and repeat analyses in this study are summarized in Table 2. Repeat structures in mitogenomes of different gymnosperm species were identified using the tool “ROUSFinde1_1” with default parameters [16]. The proportion of REs in the mitogenome was calculated as a ratio of total RE length to sequence length (both values are reported by the tool in the “_binned”-output table).

### Estimation of cellular copy number of the mitogenome in megagametophyte tissue

Sampled and trimmed paired-end Illumina reads were mapped to the *A. alba* mitogenome using CLC-GWB with default parameters, but with a length fraction of 0.9 and a similarity fraction of 0.95. The mean mapping coverage to the mitogenome was calculated (70.3X). The cellular copy number of the mitogenome in the haploid megagametophyte tissue was then calculated by the following ratio: mean mapping coverage to the mitogenome / haploid nuclear genome coverage of the input data. The haploid nuclear genome coverage was estimated as the ratio: amount of input data in Gbp / haploid nuclear genome size in Gbp. The amount of input data was 7.43 Gbp. The haploid nuclear genome size of 16.94 Gbp was used according to [82].

## Supplementary Information

**Additional file 1: Table S1.** Summary statistics of the two PacBio sequencing runs using five SMRT cells. *Abies alba* sample Aa06 was sequenced on three separate SMRT cells, but with different loading concentrations. **Table S2.** Primers used for experimental validation of the predicted circular structure of some mitochondrial scaffolds and of alternative contig connections. **Table S3.** Plastid-derived DNA sequences with more than 90% similarity to the *Abies alba* chloroplast genome sequence (NC_042410) [60]. **Table S4.** List of repeats in the *Abies alba* mitogenome based on sequence analysis with ROUSFinde1_1 [16]. **Table S5.** Genome sizes and repeat proportion of all published mitogenome sequences of gymnosperm species (without *Abies firma*) based on sequence analysis with ROUSFinde1_1 [16]. 

**Additional file 2. **High molecular weight DNA extraction from needles of *Abies alba* for PacBio sequencing and PCR amplification. 

**Additional file 3. **Alignment of *trnY-GUA* gene sequences and BlastN analyses of *trnY-GUA* *vs* different *Abies alba* (assembled) sequences (CLC-GWB). 

**Additional file 4. **DNA sequences of all different repeats ≥ 24 bp identified in the *Abies alba* mitogenome using ROUSFinde1_1 [16]. 

**Additional file 5. **Size distribution of repeats ≥ 50 bp identified in the *Abies alba* mitogenome (repeats according to Additional file 1: Table S4). 

## Data Availability

Raw sequence data of the PacBio sequencing are accessible at the European Nucleotide Archive (ENA; https://www.ebi.ac.uk/ena/) under project PRJEB52007 (accessions ERR9468746-ERR9468750). The annotated mitogenome sequence of *A. alba* (11 scaffolds) is accessible at the NCBI GenBank (https://www.ncbi.nlm.nih.gov/genbank/; accessions ON378818-ON378828).
