# Supplementary material for: The mitochondrial genome sequence of Abies alba Mill. reveals a high structural and combinatorial variation
Source: BMC Genomics. 2022 Nov 28;23:776. doi: 10.1186/s12864-022-08993-9 (PMC9703787; doi:10.1186/s12864-022-08993-9)
Supplement: Supplementary file 2 — Additional file 2. High molecular weight DNA extraction from needles of Abies alba for PacBio sequencing and PCR amplification. [file 12864_2022_8993_MOESM2_ESM.pdf]

Additional file for "The mitochondrial genome sequence of *Abies alba* Mill. reveals a high structural and combinatorial variation" by Birgit Kersten, Christian Rellstab, Hilke Schroeder, Sabine Brodbeck, Matthias Fladung, Konstantin V. Krutovsky, Felix Gugerli

Additional file 2: High molecular weight DNA extraction from needles of Abies alba for PacBio sequencing and PCR amplification

a) Detailed protocol

These nuclei extractions are based on Workmann *et al.* (2018) [71] with modifications on the basis of personal communication with Vikash Kumar and Zulema Carracedo from Umeå Plant Science Centre, Sweden.

**Step**	**Sample Aa06**	**Samples Aa18/Aa19/Aa20**
Source tissue:<br>Fresh needles harvested in May 2017, stored at –80˚C.	1.2 g	1.2/0.9 g
Grinding:<br>In liquid nitrogen with mortar and pestle until a very fine, light green powder is present.	30 min	25 min
Transfer:<br>Put cold plant powder into a pre-cooled 50mL Falcon tube containing 10-15 steel balls (d=3.2mm).	✓	✓
Sorbitol wash:<br>Immediately add 15 mL sorbitol wash buffer (V. Kumar, pers. comm.), shake by hand until all powder is in a homogenous suspension. Incubate tube on ice for 5 min, centrifuge suspension for 15 min at 3000x g at 4˚C and discard supernatant.	NO sorbitol wash performed	✓
Resuspension:<br>In ice-cold NIB, shake well until homogenous.	Resuspend plant powder directly after grinding in 20 mL NIB (no Sorbitol wash used)	Resuspend pellet in 15 mL NiB (pellet after Soribitol wash)
Incubation/lysis of cells:	On a rotating wheel at 20 rpm at room temperature for 20 min	On a horizontal shaker (Kühner, Mini Shaker) rotating at 75 rpm at 4˚C for 40 min
Filtration:<br>Collect filtrate in new 50 mL tube. Wring and press gauze/Miracloth with clean gloves to get all liquid out.	5 layers of Miracloth	2 layers of gauze, followed by two filtrations trough 5 layers of Miracloth
Centrifugation:<br>For 20 min at 4°C (SIGMA K15, Rotor 1150)	2000x g	1900x g
Washing:	4 washing steps	6 washing steps

Discard supernatant, add 1 mL of NIB, resuspend pellet with a synthetic brush. Transfer resuspended pellet using a wide bore pipet tip to a new 15 mL tube. Add volume to 15 mL with ice cold NIB, centrifuge 10 min with the same conditions as for nuclei centrifugation. Repeat washing steps.		
Resuspend the last pellet in 1 mL of ice-cold 1x HB, transfer suspension carefully with a wide bore pipet tip to a 1.5 mL tube.	✓	✓
Take aliquot to check 10x diluted nuclei-suspension with a phase contrast microscope. Keep aliquot on ice.	✓	✓
Centrifuge nuclei at 7000x g for 5 min at 4°C (SIGMA K15, rotor 12130). Discard supernatant. Snap freeze nuclei immediately in liquid nitrogen and store at –80°C	✓	✓
HMW-DNA extraction:<br>Performed with Nanobind Plant Nuclei Big DNA Kit (Circulomics, Menlo Park, CA, USA), following the user manual v0.17. Elution of HMW-DNA in EB-buffer.	Elution in 80 µL	Elution in 100 µL
QC measurements after one day at 4°C: QuantiFluor® ONE dsDNA System (Promega, Madison, WI, USA) for quantity, Nanodrop ND-1000 (Thermo Scientific, Waltham, MA, USA) for ratios at 260/280 nm and 260/230 nm, 100 ng were analyzed on 0.7% agarose gel to check the integrity of HMW-DNA.	✓	✓

b) DNA quantity and quality

Sample	Date of nuclei extraction	Date of HMW-DNA extraction	DNA concentration (Quantus ONE DNA) [ng/uL]	OD 260/280	OD 260/230
Aa06	13.03.2017	02.04.2019	1163	1.84	2.31
Aa18	07.01.2020	09.01.2020	495	1.83	2.23
Aa19	13.01.2020	14.01.2020	310	1.85	2.09
Aa20	13.01.2020	14.01.2020	315	1.90	2.07
