# Supplementary material for: The mitochondrial genome sequence of Abies alba Mill. reveals a high structural and combinatorial variation
Source: BMC Genomics. 2022 Nov 28;23:776. doi: 10.1186/s12864-022-08993-9 (PMC9703787; doi:10.1186/s12864-022-08993-9)

Additional file for "The mitochondrial genome sequence of *Abies alba* Mill. reveals a high structural and combinatorial variation" by Birgit Kersten, Christian Rellstab, Hilke Schroeder, Sabine Brodbeck, Matthias Fladung, Konstantin V. Krutovsky, Felix Gugerli

Additional file 3: Alignment of trnY-GUA gene sequences and BlastN analyses of trnY-GUA vs different Abies alba (assembled) sequences (CLC-GWB)

a) Alignment of *trnY-GUA* gene sequences (“create alignment” tool, default parameters)

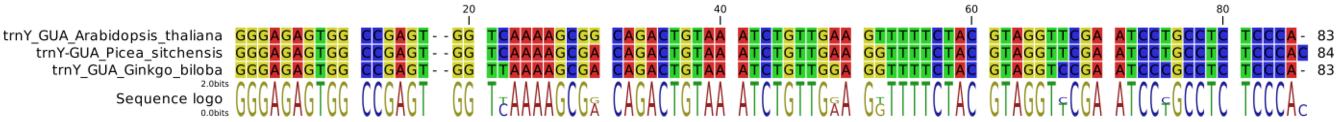

b) BlastN of *Picea sitchensis trnY-GUA vs* the *Abies alba* mitogenome (“blast” tool, default parameters)

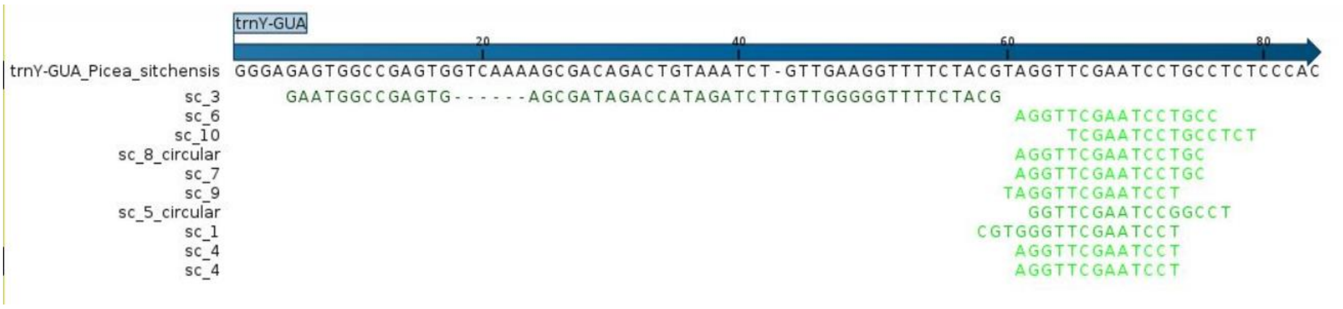

c) BlastN of *Picea sitchensis trnY-GUA vs* the *Abies alba* Illumina contigs (“blast” tool, default parameters)

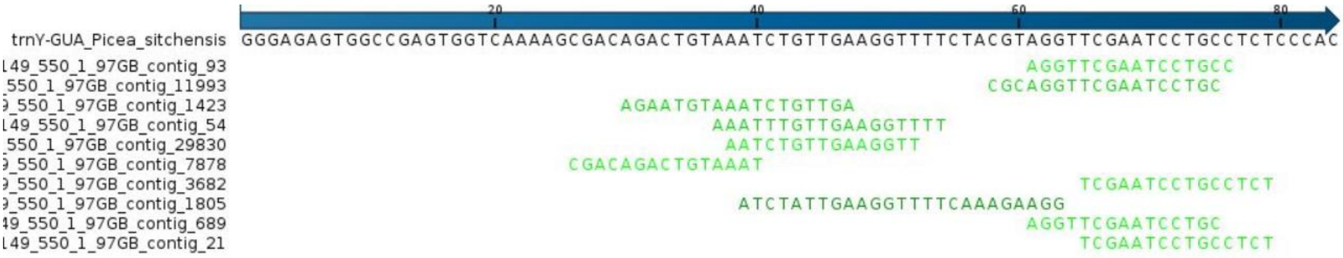

d) BlastN of *Picea sitchensis trnY-GUA vs* the *Abies alba* Illumina reads (“blast” tool, default parameters)

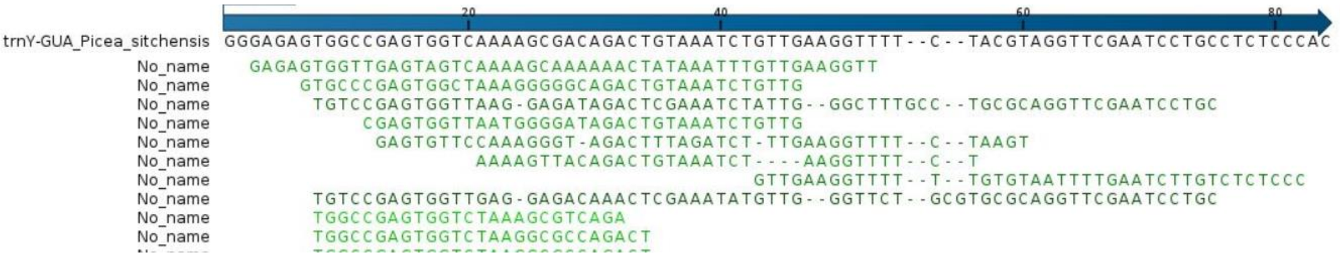

e) BlastN of *Picea sitchensis trnY-GUA vs* the *Abies alba* PacBio reads ("blast" tool, default parameters)

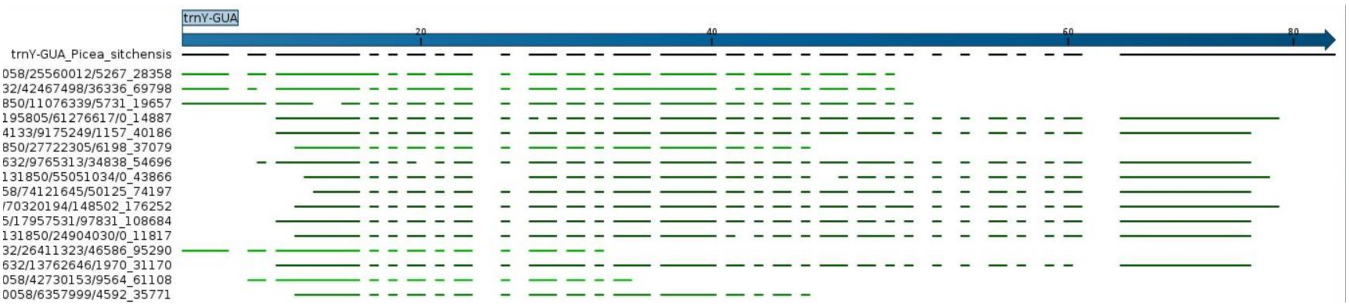

Supplement: Supplementary file 3 — Additional file 3. Alignment of trnY-GUA gene sequences and BlastN analyses of trnY-GUA vs different Abies alba (assembled) sequences (CLC-GWB). [file 12864_2022_8993_MOESM3_ESM.pdf]
