# Supplementary material for: The mitochondrial genome sequence of Abies alba Mill. reveals a high structural and combinatorial variation
Source: BMC Genomics. 2022 Nov 28;23:776. doi: 10.1186/s12864-022-08993-9 (PMC9703787; doi:10.1186/s12864-022-08993-9)
Supplement: Supplementary file 4 — Additional file 4. DNA sequences of all different repeats ≥ 24 bp identified in the Abies alba mitogenome using ROUSFinde1_1 [16]. [file 12864_2022_8993_MOESM4_ESM.pdf]

Additional file for "The mitochondrial genome sequence of Abies alba Mill. reveals a high structural and combinatorial variation" by Birgit Kersten, Christian Rellstab, Hilke Schroeder, Sabine Brodbeck, Matthias Fladung, Konstantin V. Krutovsky, Felix Gugerli

Additional file 4: DNA sequences of all different repeats equal/larger 24 bp identified in the Abies alba mitogenome using ROUSFinde1_1 [14].

>Repeat_9
AGGGAAATAAGCTTGCTTCCTCAGGGAAATAAGCTTGCTTCCTTCGTAGTGATTCATATGACTCGCAC
TCAAACATAAATCATATTATATATGTAATTATGCAACTAGTTTAAGTAGGGGGCCTGGCTCTTTCTCT
ATGAACTAGCAGCATTCCCAGGAGCGTACCTAGCAGCACAGCCTACCCCGTCCGCTATCGTCGATTTC
TGCCGCCAGCCAACTCCGGCTCCCCTACTGCTTGTGTCTCCGCTGCTTGCTCCTCTGAAGAAGCTAAG
ACTAGAGAAGCAGCAGTCGATTAGACAGTTGACTCAGTTGATCCAGTACCAGCAACCTCACTTCCCCA
CGCGGCTGTTCCAGATCGAAACCCAGACCCCTATAGCAATAAAGGAGGGAACACTCAATTTCACCTAC
TCAATTTGAGGGCTCACTCAACCGGTTTAGATCAGGCCTTTATATACGTCATTTCGGGCTGGCTTTAT
ATCTACCTTTTAGCCTATATGAGGCAAGTGCGAAAGCGTAATTACGTATATAATACGCTATTGATTCG
CATTATCATTATATATGTAATTATGCTTGCCTTATTACTTGAAAAGTACCAAAGAATTTATATGGGTC
GCAGCTCCTTCCTCATGCCCCCTAAAGGCATAAGCATGTAGCTAAGACGGGGATCCTGTAGCTTGCTT
CGTTCACTAAGGGTCAGGTCCAGCCTGCTAAGGTGGCTTCGAAGAGCTAAAACCACGGAACAGTTAAG
GAGCCTTGTTAGAAGAGGAAGGAGAGGGGTTGTGCTTAAGGCTCTGAAGTTACGAATTGGGGAGATAG
CTTTTTCTAAATTGGGGGGCGGCAACAGCTAGGAGTAGAGGGAGTAGGGATGGCTAGGTACCACGAGA
GGGGCTATCGTTGTGCTGAAGGATCTGTAGGGTAGAATCAGTTTCGGTAGATTCGTGGAGATAGGATA
GCGACCTAAGGAGCGATCAGTCTTTCCGGCTTGATCCATCAGCGGCTTGCCGGGCATAGTGGGTTACT
GCCTTTCTATGGCGTCGAGCAGCGCAGCTAAGCGACTTTCTCTTTCCTTACTATTGATTATCGGTTGG
GGCAGAAAACGTAACTCAACTGCTAGCTAAAAGCGCATTGGACATGTATGGCAACTAAGTACAATTAG
TTACCTAATATTACTCTATTCATTCTGGCCTACCTACTGCTATGCCTACTCCAGGCAGAACACACAAT
CCCAGCAGCCTTCAGTTACTTATACACTTAAAGATCCAGGTTTAGGTAATAATACAGTATTGGTATGA
GTTCATTGCCTCTATCATTGGTATGGACTGCCTCACTGTAGAAAGTCACGCTGGTCTCTCTGTTGAAA
CGCTAACCGGTTCATCGACTGGCATGGACTCCCGAACTGCCTCGCGTGGTGCAGTCTCCTTCCTTTGA
TCCAGATAGGCCACTTAGTTCACTCTTCAGAATGCGTGTAAGCCAGCTGATCCAGATAGGCCCTCCAT
CATAACATCCTTACAACTTTGGCTAGTTCCCCAGACACCTTCTTATTCGTGCTTTAGGGAGACATCCT
AGAGGGCATATTTATCTCTATTTTTTTTTTTATTGAACTGTTTCGTGCTTTAGGGACACAATAGAGTA
GATACCTCCCCGAGACAAACACCTTATTCGTGCTTTAGGGAGACAGTGTTATACATACCTATGACTTA
GAATTGAGGGAAGTATTGAGTTATGGGTGATGGATGTATGGCTGAGCCTTCCTTCCATAAGCGTGCGC
TGGAATCATGGAGCGTTCGCTGGAATCAGTTCGATGGACGCTGGAATGTCTCAGTGCCTGTATAAATG
ACTGTAGTTCCTTATCTGTCTTTGGATTAATGCGAACCTGCTAAAGCTGCTCTAAAGCGAGGAGCTGC
TCTAAAGCGCGGCTCTGTCCCAGAAGTAATGTGCTTCCTTATGAACCAGTTAGGCGGAGAACTCATGA
CTTGGACAGGTAACTTCCTTGTGATGGCTGGTTCCTTAACTATATCTAGGGTGATCCTATAAGGTGTA
AGGTGTTCCACAGATTTGCCTAAGAATAATAAACTTTGCCTAATAGATATAGCTGAGGGGCTAAAGAC
GAAGACTGGTTCCTTAACTATATGAAGGGTGTTCCTCTACATGGTTATGACTGGTTCTCCTTCCTTCC
TTGAATGAATCTATTTCCTTGCTTTGGGGATGGGGGACCTTCGTTGGTGTAACTATACTAATGAAGGT
TGGTAACTCTCTGGTTCCTTCGCTTGGGTGGCACCTTGGTGGTTGGATAGCTCCCTTTGGTGGCGTGG
TGTCATTGGTAACTATGCTTGACCACGGTATTGGGTGTGAGAGCGAGTGCTTCGTTCGGTGGCTTTCT
CTGGTAACAATTGCTTAGCGAGCGTTACTTTATCCTGTCCTTTAGCTACTTCTCTGGTAACAATTAGC
TACTTCCTATTTCGTTCCTGCGTAAGTTCTTCGTTGGGTGGCATAACTGCTAGGTACTGAGTAGAACT
AAGGTGGGAAGGATGGGTGACCTTGAGGCTGACCTACGTTTAATGTGGTTCCTTGCTATTGGGTGCCT
GGACCACCCTTGCTATTGTAAATAATCTATCTCCTTACCGCAGTGGCAGAAGCAGGTGGCAGAAGGAG
TTAATGAGTCAGTGCCTAATTCCTGAGCTGGAGACGCAGAATCGGGCGGGTGGACCCAATTCCCATCA
GTTTCTTTCCTTCTTAGTGGTATCACCCGTACTTAGCCAGCCAACTAACGATGCATTTCCCTCCCGTT
AGCCAGCGTTAGTGAATGAAAGAATAAGACATCGTGGGTATTTTATAATAAGTAGCTACACTTTACCA
ATTAGGTACATAATTGTACTGAGTAGCCACATTTTATCTACCGCCCAACTAACTATGCTTCCTCACCC
GAGGAGATAAAAGGGGTGCGTATTATAGGGGGGAACATCTGGTTGATCGTATCTAAAAGTATAGGGGT
GGAAGTGAAGTGACTCAATCTCGCCCATCCTGTGAACTAATACCAATGCTCACAATCATGGGTGTGTT
TCTTTTGACAGTAAATACCTGGGATCATAGATTTCTTTATAGCACACTGGTTTTTATCTCTTTACTGC
TAATGAAATCATTTCCGATCACTCCGTTTTTATCGGAGCAATTAAATGCGAATTTCCCTCTACAATTG
CTCCGAAACTTATCCGACTACGATTCTTTGCCTTCCCCTCCTACGTCGAGAAAGCGGCTTCACACCCC

ACGCCTTTAATAATAAGACGATTATTTTCCTTCGAATGGATTGCAGCATGAGGCGCTATATCTGCACT
CGCCCGGTATGCTTACGATTGAGCCGTTGGTTTCGTATCGGCCGCAGCTTTTACGTTCTCAGACTCTG
ATCTCGCGAAGGCCGTGTGGGTTACCGCGGTTCTATGGCCTTACTTAGCTTTGTCCACCATTCTATCT
ATCTATCCTATCAGCTATTTCTTTCGTTCGTTCATCCTAGTGACCTCTCTATTAGTGCTTCTTTCGCT
CATCTGCCGTTACTTAATTGTTACCAGAGGCTTCTCTTTGTATGCCCGTCCACCCTTTTATCGCTTGC
CCGTGTACTGGTGAGGGTGCAACGTGAGCTGTGATTTTCTTTCGTATTTTCCGGTCCCGGATAAAATG
CAATGGCTTCTACTTCCGCTGCCTTCTCCGTTGTTAGATACCGCGGGTTCTCTATCGGGATATTGATC
TATAAGGTATGCCGGGCGAGATGCTTGCTTTTCTGTCGCTTATGGATCTTTAGTCAACTAGTTCAACA
ACTTGATCAATTGGAATTTACTTTGTATCTGCCTTCGCGGGATCATTTACTGTTTCAAGGTCAACTCG
ATCTCGTATCTGCGCCCGGCAAGGACTATGACTCTATAAATGGAAGAGATGCCACTGGCTTTCGAACT
GGAAGGAATCCTTAGCAAAGTGGTGGTAGAGAAGCTAGGACATGAAGGTATACCGCGGGCTTCGCTGC
AACCTATGCCTATCCTGCCTATGTCTTTACTGGTCGCTGTGCTTCCGAAGCTGCTTTTACATTGTGCT
TGCTCTCAACTTCAATGCCTGAGCTTGCTCTGCTCTCTCCTCCACCGACTTCCTGGGGTCTGGTGCCG
CTGTTCCTCTATGGGGGTCTGGGGGTAGGGATCGATTGCCCCAGGAAGAGGTACTACCACTATTGCTT
CATCCATCCTCCAAAGAATGAACAAACAGGGCATTCTCCGTTGACCTGGTGGAAGAAAGAGCGTGTAC
AGTTAGGGACGCAACGAGACGAGACGCAACGAGACCGGACGGGAGTCGATAGAAAAAGGGCAATGGCT
TTGGAACCTTCCCATTATGAACTCCGGAAGCCAAACAACGCCAGTAAGCTATTTGACACATCCAAGAG
AAATAAGTGAAATCCGTTACACACCAACCCCTTCCCTGGGTGGGCAGCAGATCATTAGAAAGAGCCGG
GCCGATCGAGACACATTAGGAGGGGAATAAAGCATAGCGCTATGGGAATGACCGTCCCTAAATAACCG
TTTCGATGATGGCAATACCGTGCGTGTCCAAATAACCGGTTCTGAATGCCGTTGGGAATCCGTGTCCA
AATAACTGGAAATGATAACTGTTTTCGAATGCTGATGCTGCTTCTCTCTCTTACTCCTTCTCTCTCTT
CTCTATAAACAGCCCGGATGCTGAGAGCGTTCACTCCCTAATATGAAAGCGTTTACTACCTGGCCTGT
ATTCTCTCTCTACGGCCAGCACTTAAGAGACATGGCTGGATGGTATGGTAAAACTTGGATTTCATAGG
GCTTTTCATAGCTTCCCTTGCTCCCTATGATTGCTTGATAGCCGAGAGGAGCGAGTTCCCTTTAGCAG
AAATAAATCGTGTGGAACTGCCGGATGATTAATTCTCAACAGCCAGAACTTTAGAGACATGATGGCTA
GATGATGGGACATCGGAGAGCCTTGTTTTAGACGGGCTTCAGACTAGCTAGGGGATGCCATGATTGCT
AGGAGATGTTCACACCTTGCCTTTAGTAATCTTAGTTAGGAGGGACTGCTATTAACCTTCTTATCAGG
ATCAGGACCGGGCGGGATCACATAACCTTACCTCTTCCGTGTTTCCTAACTACCTGCCCACCTTTAGA
GCTAGACCTTTTAGTCCCTCCCTTTATGAGGTTGGCTTGCGAGACTATACTTAGGGGTGTGCCAGGAA
TTAAGAGGATTGATAAGGACCTTACTGGGCAGGACCTTTCCGGGGCAGGACCTTCTTTTCAGGACCTT
ACCTGAGCAGAGCTCGAGGAATCAATAAATAATAAGGGATAACCTTGTTCGAGCCGGCCGAAGAAAAG
GTGTACTTCCTGAAAGAAGGACGAATGACTCCGCACTCTAACAGGTCCTTCCTGCAAAGGAGTAGTGA
TAGGCGGGTGAATGACTGTGTTGCCTCCTTCTCGTTGGCAAGCCCCACACATTTGCTGGACCCACACA
GCGAGCGATAAGTGACATTCCCTATTGATTGGAAAGAATAGATCAATTGGGAAGTGAGCTTTCGATCG
ATTAGTTACCGACCACTAGTACGACAGATCGAGAAGACAACAATGTGCAAGGGCTGTGCACTCAGCAA
GCATGCCAAGACTACTTTCCCTAGCAGTGAGCATAGATCAAGAGGTCTTCTTTATATGATCCTATTTG
ACTTTGGAAATAGCCTAATGAAATGAAAATGGGCTTGCTCTGCTACTGGATCTATCGCTGTTACTGCT
GCCCCCTGGTTTCCACTCCGGCTCTCTTCCTTGCTTCCAGGTTATCAGAGTCAAATTTATCATACCTC
CCCTCTATTTTATTTGGACCCTGCTGGTTAGATAATATAATAAGTAGATAATATAAAGAAAATAGAGC
AAGCACTCCGTCCCTCGCTTCCACCCTTTAACTAGCGAATTTACCAGGTACGAGCGGGCGGGTTCTCT
CTTTCTTTAGGGATTTCTTGCTCACTTGAAGCGAGTTATTGCGTAAAGAAAGTGACTTCTTCAGTTAG
CCATAACAATTTATTCACCTTCAGACATAAGTCTTAATGGCAATAAAGTGAATAGAACCCCTCGGGAA
TCAAGAAGAGTACACCCCCGAGCCAATGAGCAGGCAGGTTGAGCCACCCCAAAGATATGGTTATTTGT
TTGCCATACTTCTAAGCCAAGACAATAGTGTAGAAGCCCGAGATCAGTCATGTCGACGTTTCATTAAG
GTCATTCTTTGTTGTGTCAATCATCTTCTCATCACTACCTGTGATTACAAAGTCATCGACCAAAAACC
TCCCCCTTGTTTCTTGAGGTAGAGGTTCGGATCATATGGACTCCGGCTCCGTGCTGCACCAAATATTC
ATCTATCGTTGATATACCAAAATAACTAACTGGGTTTAGAGTTTGAGACCATAGAGGGATTTTATGAG
TCTACATACCGTTCCTGACCTGGTGCAATGTATCCTTCTGGTTGGGTCATATAGACCTCCGACATAAA
CAAACCATTGAGAAAGGCGCCAAGTGAGTGAGCGTTACGTAACGGTAGTTAGGGCCATTTGATACATT
TAACAACATTACATATAGTATGTAATCTCCTTTGCAGCTAAAGCAAAGACCATTCTAATAGTATTTAT
CTTGGCTGTCGGAGCAAATGGCTACCATAACGTCCCTTCTTTTTGGGCAAAACCCTTAGCCACAAGTC
TTGCCTTATACTTGTCTATACTGCCATTTGCCCTATATTTGTTGGTCTTTAAGATCCATTTACAACCG
ATGGCTTTATTCCCCGGTGGAATACAAGCTCCCAACTCTTATTTGTAATTATTTAAGTATATTCTGCA
TCCATGGCTTCCTTCCAAGGCTTAGACTCTAGCGCTTCCTGAACAGTAGTAGGTAGTTGTAAACCACT
GTGCTCTAGAGTAGTGCTAGGTTAACCTCATTGCGAGCCATGCTGTCCTCGGTCCTGAAGTTGAGAAG
TCCGCCATTAAGCTTTGCATCATGAATGGTACTAGTGTACCACGCACACGTTGTGTGGAAAACAGGGT
GCTGATCCTGGTTGATCATCATCATACCCATCTTTTTCATCTCCAACCTAATCACTATGCTCATCTAG

ATTGATCATAGGAGCACTATCTGGGTCAGGTGAATATGTGGAAGGTGAGTTGGACTCACTCTTCTCAT
CCTAAACACCATCTATACTGATGTACAGCTTTCTAGTGGTAGGATCCGTCGGTAAGCCTTCGACTCAT
TGCTATACCCAATAAGGATGCATTTCAGGGATTTATCATCAAGCTTCTGTCGTTTTGGTTGGGAACAT
GCATGTATGCTTTGCAACCAAAGACACGAAGATGAGAGATGGAGGGCTTAACTCCTCCTGTCCATGCC
TCCGGAAGGTGTGATGCCAGAGCTTTGGTTGGTGCGCGATTTCATAAATAAAACCACCCCAAACGAAA
GAACACAAACAAAAAAACATGGGACTACGGACCGGCATCAACACACTTGCTCCGACCTACGGACGTCA
TAACTTGAGCAAGACGAATGAGCTGACCACAGGACAGAACATTCAATACTCTCAAGAACACACAAGCA
CAGCTTTCTCGCTAGACAGGCAAGCGAAGCGATACTCTAACGCACGGGAAAGCGAGCAGAGCCATACC
CTCATACATGTTACCTTGCCCGGCGGCGTCCTACGGCCTAGGATAGTATGGTAGGCCCTGAGAGCGAA
GGGTAGTTGTTCATGCTAATTCTTACCGGTCTCTGATACAAGCCTATCCAAAATGGGGATAAATGGTG
GTTCGTGGCCTCGACTTGGCCATTGGCATGGGGATATTTGGGAGAGGATAAATACTACTGGATGTGAT
GAAATAGAGGTCAACGCTGTGATGGATGGATTTGGAAAGGTGTACCATTATAAGAATTTATTGGAAAA
GGAACACCGAAGAATGTAAGGATGTGGTTGAGAAGGAAGCGACAGGGCAGTGGTGTGCTTAAGTAAGA
CAACTTTTACACACTTGGTGAAGTACTCGATGACCGTGATTATGAAACAGTGGCCACCGAAGGAGGGA
AAGGTGATTTTACCACTGGTGTTGAGGCCCCATTTCTATAAGGGCCAGATACAATCGATTCGATGAAG
GAGCCGTTGGCACTTGTCGCATTTCTGGACAAAGTGCGTGCGACGCAATCATTCTCCAAGGACGACCA
GTAGTCACCCATGTGGAGGACCTTCTTGGCGATGGCAATGCCACCGAAGTGTCCTCCACACAAGCCAC
TATGGATCTTTTGGATGGTGGGCAGATGTGAAAGAGGAATATTATAGCGGAGGAGAAGGTCGTCAAAG
CTCCTTCAGTATAGGATATCCCTGACGAGGCAATAGCGGTCGACCTTGCAATGGAAGGCACGGAGGGG
AATTTTTAGACAAGCCGAAGGGCGAGGAGGGAAATGAAAGGTTAAAACTTGGCCGAAGAAAAGGTGGG
AAAAAGTGCAAGAATGACCAATAATAACGCATCCCTAATTGCGTCAACTCCGCCGAGAACTTGAACGA
GCGGTCTTTGTCTCAAAAAATGAAACGGAGAGGGGGGGCCGCCCCCAGCTAAGGGGCGGTTTTTACCG
CTATATCTCGATATTTGACAGAACTCGACTTCGATTTTAGCTCTCATTTCCTAAAATGCATATTATAC
TCTATTTTTCGATCGAAGAATTGATCAAATCCTTTATTTAATTTACTTTATTTCTTTGTTCTTCCAGT
CATTATTTATAAAGGAAATCCACTAATAAATACTGGTTAGGGAGCGGGATCCGAAGTAAGGATGAGAG
AACGTGAAGGACATCAATACAACTATGTTCCCGAGCATAGCTGGGTGGGCGGAAAGCTCTAGTAGGTT
CTGGGCGATGGCCCAAGCCCGAAAGACCCTTACGATAATTATTTAGCAGCCGAGTGGTGGGTATCCTG
ACTCATCAATCAATAGGTACCAAAAAGCTTGACTCACGCTTCTTTCTGGTCGGTCTATAGAATCAAAA
ATCTTGGCTCAGCAATAAAATCCCGCCCGTTTGTGCGCGTATAGTGGCCAAAGGGATAGATATTGTAA
GCACGTTATGCGGAGGTTCGACATCAACCTCCCCCGACGATCTCACACGCTACGTGACTCCAGGCTTA
CTCATTTAATCTTTAAGCCTTTTTGTTTGTTTTTAGAGGTAACCACATAAATAAGGGAGGGGACTATT
TCAGGAAGTAAGCCCAAAGTAACTTGTCATTACTCGCTTTACCCCGCCAACGCTCCCAAGCATGGATG
GACGCAGGTGCCCACCCACCAAAATGTCCTTTATCCAGTAAAGAAGAAACGATCCCCCGCTTAGATAT
ATATGGTACCATTCTGATTATCGCTACTGATTGTGGTACAACGGCGCAAAAACTCACGCTTCTATCTG
TTAAGAAATTGGGGAAAATCGCTTCTTACTTCGGTTAGGGATTGGCTCGGCATGAACCGAGTTGTTTC
AACCCATTGCCAAGCGCGAAAGAGCATAAGTACTCGCTCGAGGGCCTCAAACAAAGTCAAAATAGTTA
ATAGCTGCTTTCCAGGGCGGTGGACAGAGCCGCTTCAAAGCCACTATGTTGCTTCTTCTCCATCCACG
GTTATCCCCATCTTATCGCTGCGACTCGACATAATAAATATTCTTACTTCTCCCTGCCTGCCCGGAAT
GATAGATTCGTAATGAATTGCTGGGCTGTGGGCTACTCGGGTGGTGGGAGTAATCTCCTTAGGCTACT
CATATATAGCTTGATACCGATCCATAGAGCCATAAGCCCTATTCTATTGATTATGAAACATCGACTTA
CAACATACTACTTGAAGTAAGTCCGACCTATAGGTAATTGAGTTCATTACGAAGTGTGATTTGACTGA
GATGAAGGGGGGGCCTTGGGGCTCCTTATCCATCGCCTCTGTGTGGTGGTTTTGTCCCTACTGAGTTG
AGTGAGTGCTTCCTCTCTCTACCACCGTGGGATCCTTACTCTACGCGAGCCACCCATGCCCGTAATGG
TTGAGTGGCCTATCTAGCATATAGAGGATGTAGAATCCGTGTGCACTTTCTCTGTGCGGGTCTCTCTC
TGCTCCTATCCCTATCGATTAAGTCCTCCTATCTCTACAGTGTGCGTGCTAGTGGTTGGTTTTGCTCC
TCTCTGGCTGGACGAATTCCTGTCATACTTTCTTATTGAATACTCGCCAATAGCGAAAGTCATGGCTG
GCTCGACATATATTATTTACATGTCAATTGTCGACTGGGGACGTTGCACTTCTCATTCATTGAAGATC
ACATCTCTGTTTTAAATCCAAACACCTGTCTCTGGGTTATACAGTAAGTATCCCTTTGTTTGTCCCGT
TACTAGATCCTAGGGATCGACATCGTTCACTATGGGGGGGAGTCCATTCAATTGAAACCTGATTTTAT
TGTTTTACATTCAATATGCGGAACATCTGGATACTCTCTGATCGGATGGGCCACTGAAGGTTTATTTC
CCATCCAGAAATACTCAATTTTATTGTCCCGCAATGCCCTAAGGAAGGAGCACTCACTCGATGGCCTG
CCGACAGGCACATGTAGCGGTTGATACTACTATGCCCAATAGCGATTGTCAATACCTGCATGTGTAAG
CAGACATCCGCCACACTCGATCGACAAGTGTCCTTTTCTGATTCTCGACAATTTTAGAAGTGACAGAT
TTAGAAGTGACAGATGGATGGGGTAGACAAAGCCTATAGCGTAATCCTAGCCTATCCCTGACCCTTAC
CTTTACCCTTACCCGTAATCGTTCCCACCCACCTCCATTCACTTTCCAGGTGTTTTCCCATAACCCGC
ACCTATTCCTCTAGAACCAGTAGCAAGTCATGAGGCGCGGTGTGGGGCAAGCTCTCCTAACTATTTGA
TAAGCCATTAAGCCATCCAGAAGCCATCCAGTTGACTGGCCTTCTTGCCTATAAACTGGCAAAGAGTA

TATCCTCAAATCCTAGAGGCGTGGGCGGGTGAATCTATCCATATAGGCGTGGGTTGTTAGCTCCGCAG
GACACAAAAGAGAGAATCTTTCCTTACTTTACCGATGGGTCTTGGAGCCTTCTTACCGATGGGCCCTT
ACCTTACCTTGGAGTGGGTAAGGACCGGCCCTTCGGTTGTTCCTAAGCTACGCCCCACACCCCGGATG
AGGGGCATCATTAGTAGTTGGTCGAGCTAGTTCCTTCCTCGCCCGATGCGGGACGAGCCATCCTTCCT
CACCTTCCTACCGTCAGGTGAGCCATACTTCCTCACTTGGCATCCGTCCACTCTTTGATATCCCTCAT
CCTTAATCCGTCCACGTCTATCTCTCCCTGTGATTGTGTTGTGACAGTCTAATCACGTATAATAAAGC
AAATAAAATGCATATCCACCACGGGTAGAGTATCGTTCGTAACGAAAGGTATCAGGTTGTGTTTGGGT
CTGAGGTTCCCAAGAAAGGAGTTGAAGGAAGGTCCCAAGGATAAGTGTAAGGCTGAGTCCCCAAGGCA
TTTCCCAAGGCAGAGGTATGAGTATGAGGTTTCAAAGGATATTATTCCAAAGGCTGTATGGAGTATAG
AGGTGAGGTCTCGAGATCAGTGTATCTGTGGAGGTCTTTTTCATATGGCTGCGAACTGAAAGCAGTAG
GCCAAGGGAAGGCCCAAGGTAACCATGAAAGGAACGAAGGAACCGAGGTAAGGAACCAAGTTTAGAAA
CCAAGGGAACAAATTTAGTAACAAGTAATGAAATTTAGTAATAGGCCGTGAATAACTCTCTCGATCAC
TTGGGTCAGCACCGGTTTATCCCTCCTTCCCTCTAGCCTCCTCGGAAGGCAGCCGCCCGTCTGTAATC
AAGCCAGCGAGCAAGGATCAAGGCGTAGGTATGAATCTGAGTATCCCGATAAAAGTTGGACCAAGGTA
TCTATCTGTTCAGGTCCGGTCAGCTCAGCGGGAAGCCCCATAAGTGGCCTGTTAATTGAAAACACGTA
ATGCATTTATCGGTGGTTCAGTTAATGGGTGACCTTACTCACTCCCACAACTTCCTTATTGCATTATA
TACGTTAAAACAAAAAAAGCAATCACACGCCCACTAACGCGTAGTGCGCCTGTCTTTACTTTAGACCG
AATTAAATACTGCTTAAATTAATCAATGCTCAATTAATCCAATGCTCTGTTTGTGATATCACGTATAT
ATACTCTGCTTTGTGTGTAGTGTTGCTCTAGCTCTACGTGTTGTGTTTGTGTGTGTAGTGGGTATACC
TGTAAGTGTATCTCTTTCTCAGTAAGCCGGGCTCTATTTATCACCTCGTGTTAAGCTCCGGCTCTGTT
ATGTTTCCATTCCTCTTGTTTAAATCCAAGCCTCTCTTGTGATTCTGTTCCTCTCTCGTGCTATGGGA
CTTGATCAGGCTCTGTTTATTATCTCCACACAGAATCAGCTCTGTTTGTGCTCTCCACGTCTATAATC
TACGAAGGCTGCTTTTTTTGTTACATAAAGCCCACTTTGTTTTGTAGGGAGGAAGCAAGAATGCCCAAT
TTGGAATGTCCACTTGGAATGCTCCCTTTGGTAGGTAAAAACCATAACTCATCATCAAACAAAGACTC
CCCTTCCTAATTGGTGACCCGTGTTCTACTCACACTACGTTGGCCTGCCTAACCTCCACAATGTGATC
CCTCCTCGATATTCTAAATAAACCAAGCGGATGAGACTTTAGAAGTTTTTCCAGAAGGTGTCAAGAAG
AAGTGCCTTTTGTTTATTGGAAACCAGTAATTGGATACCTCCTGGGAATGATGTGGTGACATACCTGT
GTCCTCCCTGTGTATTATTTCACACCCTGTGGAAATGTGGGGAAGTAATGGGTAGGTGCTAAATGGGG
TAGGTGCTAAGAGGATCACACCACCTATTGAAATGGGACTGGACACTGGAGGTCTGGAACCTTAGGTC
TTGACACTGGCTTATTCCCACTGGATTATTCTCCCATTGCTTATTGCCTTCCCATAGGGAGTGTATCG
ATGGCTTATCCCATTGGATTATTCCCCCTGTTGATTATTCCCCATTGGATTGGATTGGCTTAGTTATT
CCCATATCCTATCTCATATTTGTATGACCTCAACAAGGGTTTTTTCCTCAACCATGAGGTTCAAGCTG
TTATTTCGTCCTTATGTTACACCCGTCTCTGCCAACGATGTTGTCTGTGACAATGTGACTGGCGATGA
TACACTACCGGTGGCTTACCGGCTAGCACAAGGACCTTCTGGTGGGCACTTAATGAATCCATACCCCG
TACCGTACCGTGTACCGTTGACTGTGGAGATCTGGAACCTTAGGTCTGGACCATAGACCTGGAACAGC
ACCAAACCTGGAGGCAACGGACTAATGCTGGAAGTGTACAAACCTTTCGGCTATGGACAAGTTACACG
TCTATATGCGATTCATATTATAAGAAGTTTTTACATATTTTCCAACCAACTTTGTGGTGCCTGCCCCG
ATTTATAATGATACCTCTGGGCACCATTCCTTAAGGCTAGTACTACCTACCCTTACCTGTAATGACCT
ATTGGACTGGACTAGCCAGCTCAGACCCTCCTATGATAACTGTAAGACTGTAAGGTCTCTGGCTAATT
GAAAAAAGCACATAGAACCAATTACTATCCTGTTACTATCCTGTTCCCGTGCTGCTGCAACTGGTCGC
GCACGAACACACATCCTATCCTGGGCAAATGGTCTCAAGAATGAGCATGCATGTTGGATGAGAACTGT
TAAAACATGTAGAATCACCTCACTTCTAACCAACCACATCTAGGGCACAAGGAATCGGATCACGTCCA
ACTGGTAACGGGGAATTCTCGGGTGGGCCCATAAAATATCACCTCCTGTCTATGTCTCATGGGTGTTA
CAACTATTGGGAAGGGTAGGCAAGCTGCTGTATCTGCTATGCTAAAGAGTAGCAGTAGTGTAGTTCAC
TCTACCTAATGAAAGGGAAACGTAGATATATGCGATATAGTATCCTGTCTCCAGCCCCTGATCCCAGC
CCAGATCTTATGACTGGAAATTAGCAGTAAAAATATGGTACACACACTAGGGATGCATAATCTCATAT
ATATATGGAAAAGTATAACATAATAGATATGCGAGTTTGGTCGAGCCTCTTCAAATTGTTGATTTCCC
AGAATTGGACACTAGTTGTTACAATGTGACTGATATTATGTATAATAGGTTCATAAAAACTTATTGGG
CACTAACAGCACCTGTTATGCTCATGGGTAGTGCCGTTGTGAAGGCCTGTTGAGCTAGCTGTCCTTTA
TTGTATGGCGAAAACTACATAGCCTAACCTGGAGAAGTTGTAAGGTTGGTGCACCTAACACTTGGTAC
TACATCCGTATACTTCCCGTTGGTCTTGGTTTAGCTCCATAACGGGTATATCCCGGGTGGTTATTGGC
GGGTCTCGGGCACTGATGACTTTGCTTATGGCAGTGAAAGCCATAGAGTTAAGGCCTTAATAAGGTCT
TTTGGAATGGTTGTAGGAAGGAGCTACTAGTAGGGGAAATATGGGTTGCACCTGCCCCCGGCCACCTG
CTTAGACCAACATGGCGAATCGAGTGGAAAGCCTATTGAACTGGACTATCAGATATGGAGAACTGGAA
AGACTGTATGGCTAAAACATAGCCTCATGTATATGCTCGTTCTGATGGGTTACCTACAACCTCACATT
GGAGAATACTAGCTCTCCTATTGTAGTATATGTTCGTGAAGTCACCAACATGGCTGGCTCTATTATCC
TTTCGTCCTTATGTGACGACATCCCGAGAGAAAGAACCTTCCTGACATGGGAAATTTTCTTATGGCAA

GGTGGCGACCCTCTGAAAATGTGGATATACACTGGTGGCTATTTGACTATTGGAGCAAGGCTAGCAAT
GACCTTATGATGATAATAGTGGGCACTTAATGAATCCACCGATCTACTACCTAGGACTCATGCTGGAT
GTAAGCCGGACTTCCCATGGACTCCTGCTGTTGAATCCATTTATCATTGTGCCTGCTGCCCCGACTGA
ACAACCTGATGAGGCCATTGAAAACTCCATATTGCCTGGTAGGTACTGACCAACCTCACTCACTCACT
CACTCACTCACTCACTCACCTTCCTGGAAACGTGGGAACCTGAAGAGAGCTCTAGTTCCTTCTCCCTT
AGTGCAGTCCAGAGTAGTAGTGCAAGGCCAGTTCTGACACTACAGCTTAATAAGATTACGGAACGGAA
ATGGCTTGTCGATAGAGCTAGTACCGGGAAGTCTGGTCGGCGGCCAGTCCAAAGGGGCTTTACTGGCT
TTAACAGAAATGGAACTAAGTGTATGGAATCAGCAGATAGTGTCTACGGACAAGTTAAACGGCTCCAT
GCGTCGTATATCTATGTTTATGCCAGGATTTAAAACAATCTAGTCTACTAGCCTATCATAGAGAAGAC
TTACCCATATGGCTGCTGCTATGGCAAGGTCTGCTCTGGTTAGGGGTTTCCCGGCTAGGTATTCTTCT
CCTATTTTCAGGGAATAAAGAGAGACAGGAGGGACTAAGACAGGAACCATGGAAGGGGTATATCCCTA
TACGGGAAACTAGATACTGGGGAAAGCTAGTCCAGTCCAAGAGTGAAGTTAGTTCATGGAATAAAATT
GGAATTGAGTGCATAGAAACCCACGAAACCCACCTGGATTTCCACTGGACATGGAGGTAGTACCGGCA
AATGACTACAGAGATAGCTTATGGATTACCATTAGTTATAATTAGTTATAGCCTATGCCTTATGTTTT
TATAATGACCTGACACCAATGATTTGAGGAGACAACAAGAAGCTTATTCGCGCTAGAGCAGCTCTGAG
AAGTAGTTGTTCATATTTCTTTTTCATTGGCACATTTAAACCCATGGAGAGCTGTGTACCCCTCATAC
AAACTTTTAACGGCCCGATATGAAGAAGAAAGAGCATTCAGTTAAATAGGAGGACGCCTTATTTATGC
TTTCTCTCTTTACGCTTGATGTTACGAAGAGCATGAGGTTCAATATGCTGTATGTTATGAAGAAAGAG
CATGAGGTTCCATAGGGCAATGTATTATGCTTTCAATCCGCATGAACCTGTAGGCATTATGAGGAATT
ATGTTCCTTTCTATATCACCCTTGAGCTTACCTTACAAACAAGATGCTATATTAAACGAAGTGCAACC
TTGAGCTTTACTTACAACCTCGAAGCGATATTAAACAGATTGCCTTACAACCTACCACACCACATTAC
TGCTTGAGACACAACCTGCCCTACTAGAGGGAGATAAAAGGAAGGCCTAACTCACTACACACCCAATG
TTATAAATTACCCAGATTGACTTACGCGCTTACCCTGCCCTGCTGAACCCATGCTTTATATACGTTGC
CGTTGTTGCCAAACAAAGTAAGTTTAAGACACTGGCTGGTTAGACGTGGGAAACCACAGCAAGCAATC
CCCAGTCTGATTGGTCGAAATGATGGCTACAACCTAGCTTCACCAAGCCTTTACCCCATCCCATTCCC
CTGGCTCATCTTATTTCTTCCCTCGATTTCGCCCCGCTCATTGAGCTTAGTTAAACTATGAATAATAA
TGCATCCGCCTGCTGATTGATCCAATTTGCTTATCCACCTATTGCTACCAACCACCTGGACACCCATC
TTGATAGACGACCGGCTGACCTGAACTTTTAGAAATCCCTTCGAAAACTAAAAAAAACTTGCTTAAGG
AACAAAGTGGCAGGCAGCAGGTTTAAGCTGGACGGGTGGGGTCCTTCTTGCTGGAATAGCTGGAATAG
CGGGCTTGGCTTTTTTCTCTCATCTAGCAATAAGGGTACTTAGGATGGGTGTAGGAAGAGGTTAGGTT
AATGGGTGCACCTCAAACAGAGACTACATCACGCACCTCTCTGACTGACACTACCTGACCAGCGTGAA
TGGCTCAACCAACCGAATTCTCTTTTCCAACTCTAGCTGCCTTACACTCCCTGCAAGTATAGAAAAGC
GGTCCATCGCCTTATTTCCATTTCCTTTCCTAACGCGGTAGGCGCACTCTCTAACATATGTAGTAAAT
ACTATTCTCTTATATTCTGGTGGGTACTTTTAATGGGTAGAGGTAGTTTGGGGAATGGGTGCTACCTA
CCGCCTAACGCTGGGGACTACAACCACCTCATCCGTGCCCTCTGTCCGCGCTATTCGGCCAGCAGCCT
CATCAGAAGATTCAGTTTTCAATAAAATCCGTTTAACTAACTCAATCGATATGCTCGTAAGTAACCAA
GCATGGCTCAATCTCATCTGTTGGGAGGGGCACATTTCTTCTTTCATCTGTTGGCAACTTGAACTCAA
TCAATATGCTCGTCCGGTAACAACATGGCTCCTCACTCTCTTTATTCAGGTGGTCACTCACATGGGAA
CATAATCTCCTGCATCAGGTGGGTGAATGAATGGGTGTAGTGAATGAATATGTGTGACCAATCTGGTG
TAACGTATATAAGCGTCAATGTCTAAATCACCGCATCCCGTGGTCGGAGCGTCCACCCCGTATGGGGT
TATTCTGCGCATGACATCATTCTGTGATTCATTTCCTCATTTTCATCATTTTTCCACCCCGCCTTGTT
TTGCGGGGTAATCAATCATTCCGTGCACCTTCCTAACCCCCAGCCACTTACACCTCCATCTAGTCATA
AAGCTCAATCCTCCCGCCAGCCTAATGCATGATCCTCGGTTCTCGCAGCCCTCGTTTAATTGACTATC
CCTCCTCGTTTCCTCCTATCCCCGAGCAATAGGCGAAAGCAATTGATGGCATCCTGCCTGTCATAGGT
CAACGTTGTGAAAAGGCATAGTTAAGTAAATAAAAAATCTCCTTTAAGTGCCCAAATGAGGATTAAAC
CTCCGATCCCCTCAAAGCGAGGTTTTGTTGACTAAAACCATTAAATGAGAAGGTTTTCCCGAATCTTG
TCTAAGCGAACATAGTGCCTGCCCCTAGCCAGGTAGCCCATTGATAAATCACTAAATCAATATCTATC
TTTCCAGTATCTTCCTTAGGTTGGGGATAGTCTGTTGGGGTTAGTTGTCTCCTTCTTATCCAATCTGT
TGGGGACTTACTATTGCTGCTAAAGACGCTGCTGTGGATAGTAAAGGTAATGTGCGGTAATGTGCGAT
AATGAAAGGTATAAGCTATAGTTTAGGGTGGGTGCCCGTAAAGCTTGCTTATGGTGGGGAAAGCGACT
TTCATCTGGGGAAAGCGGGGCTGAGGAAAGCAGTGGGGAAAGCAGGGCCGTGGATAGTAATGGAAACA
CTTTTGGAAATACCTTTCAAAAATACTTTCGAGTGGAAGAGAAAGGTAAGGTATTCATATTAAGTACA
AGGCCATACTCGCTACGCTCTGCACTAGCAGGAAGGATATTATCTGTGGAAGGTAAGGACTAAAGAAT
AGCCCGCCCACGAGAAGCGGTAATACTGAGACTGATATGATTACTTTCCTTTGCTCACCAGCCCACCA
GAGACTAGGAATCCCACCAGAAAGGTTCGGACCCAGTGATAACCAGCAATGGGTAGGTTGGTTGGGAT
GGTTGACTAGCTAGCATGGTTGACTAATGGAAATTACGATTTGATATTGAAGGATATTTGATCTAGCA
GGAAGGATACACATTTAAGCCCGTATAAGTCGTATTAATCATGAGCCCGTACCCGTAGTCGTTGTATA

AGCCCGTTGTATAAGCCCGGTAGTATAAGCCCGTAAATCAAGGTGGGCCATCCCGGTAAGGTGGGCCA
TTCCGGTAAGGTATTGCTGCTGCTGCTGCTGCTGCTGTGGGTGGAAGGTTATTGCTGCTGCTGTTATG
GTTAGAAACCGAGATATGGTTAGAATATCGAAAAGTGGATTGTATCAGTCCCTAAAATAGGAGAGAAA
TCGCTCCCCTTACAACGGAGGGGGAGGAGATAAATCCATCGTATTGTTTAAGTGGTATCACCCTTCCT
CTTTAGGCATGGTTTAGTTACCTCTGTTCCCTTGCTTGTTAGCCAAAATAAAGGCATGGTTGTGCCAT
CTTAGTTAGCCAAAGTAAAGGTCAGTGAGAAACCCCACCCGTTAGCCCGTCCTGCACTAGCTAAGGAA
GGTCTTCCATCCTGCGTTAAGGCAAGGAAGCGAATGTATTCTTGGGGGAAAGGCTTTCACGAGGGAGT
GGCTTTTACGAGGCGGTTTATGGCTTCTCAAGCCGCCATCTTTCCCATCGCCCAGCCAAGCTAACTCT
TTTGCTATAAATAAAGGAGTTAGCCCTTCCTATAGTAGGCCCAGGAAAGGTCCTGAAGCTATAATGCT
CTCATGCCTATCTACTACCGGAAAGGATCCGATCTCTCATGCCCACCCCAGCTAATGAAAGGCAGGTT
GTTGCTCTTATGCCCAGCTAACATATAGAAGCTCTCATGCCTAGGAAATAGAAATGCTGGTTGCTCTC
ATGCCTTCGTAAAGCTATAGAAATATCCTCGCCTCATGAGTAGAAATAGAAAGGATCCGATCTCTATA
GAATAGCCCAATACCGCTAGCCCAATTACCCTAGCTGCTGAAAGGTTGGTTGCTCTCGAGTAGAAAAG
GAAAGTGACATGAAACACATTCTCATGCCTTTGTAGCCAAACCATCTATAAGCTCTCATGCCCAGAAG
GAAGGACAGCCGGTCCTGCATGAGTTGCTCTCATGCCCAGAAGGAAAGACCTCTCTACCGCCCTCTTC
TTTTTCAATTGACTCAATATCTGAACTAGGAACTTGCCAACCACACTCTAAAACAACAAGCAACAATC
GGAGCCTCTTGGGAGAAGGCATGCGTGTTCTCCTCTACTCTAAGAGAAAGAAATCAGGTGCAGCATTT
GCCCTCATTAATGCCCGTCACATCAAAGCCTGCTTGCCCAAGAAAGAAAATCCATATCCTATCCAAGC
AAGCTACGCCTTCCTCGGGCCCGGTATCCTTCCTTTATCCCACCCAGAAAAAAAAGCCCATCAAGGAGA
TTAGATCCCACCAACTCATCAAGGATAACATCTTTGTCCTTTCAGGCCAATTCATCTATCCAGGAAAG
TACGTATCTGTACCTTATAGTATGTATGATTCATAGCACGAGTAAAGGCACTTTCAGTCTAATTACTC
TCTCTAGGAAAGGATACGTACGAGTCATTGTCCTTTCAGTCTAATTACTCTAGCTATTATCAGTTCAA
TGAATCTATTCATTAGTAAGTCTTTTTCAAATAGTGCGTAATTTCTCCGGTTCTGAGCGGAACCACTT
TACTCCATCTCTGCGCACCTGTATAAATGAGGCTTTAGTGTTGCATGGCCCAGTATCTGATATGTTCA
AACTACCTCCATCCATCCGTTAGTTCCAAAACATAGAACTATTGGTTCGGTCAAGTAAGGCGAGAGGA
CCGCTAGTTTCTCCATCTTTCCCGCTTGTTGGCCATAGATAGCTATCCCTAATAAGATTCCATTGTCC
CAATGATGTGCGGATTCCATCTCTAATGACCTCCAGCATGAAGTGGGTTTGGGCGAGAGGGGGAACTT
GAAGCAGGCACATCGGGGGAACATGACCCGAGTTTTGAACAGCATCTAGAAGATCAAAGCAAATTAGG
TTTCGGAAGAGCGGATTTATAATGCGAGGACCAGCAAGCAAGCTTCCAGGGAACGAGGAGCGAATGAG
ACGAAAAGGCAAAAACAAGAAATATTTCTCCTTAAAATGATGATCCTTAAAATGCTGAAGAACGACCA
ATCAAAGCAGTTCCCTGACATGGCTCGATTTGGCTAATGCTAAAGTACCTAAGAAGCAGCTTTTCAGA
ATCCAGTCTTCTCTATCTCGCGGTCCCCACTACTAAGCCTAATATTTGCTTGGAAATATGAAGCAAGT
AGCTCAGTTTTGTGTCTAGCAACCAAATAAACACGCACAATGTAATTTCTCTTAGCCCTCGGTCGCTA
CTAAGTGTCTAAAGCGGAGTTGTGTCGCTGCTAAGTGCGTCAGCTATTATGTATATAATATTTCGAAC
TGCTATCTATGGTGGTCCGAATCGGTCAACCTTTCTCGCAGAATCCAGTCTTATAGTAGCTATATCCG
GTCGATTCTTAAAAGTAGGCTTTAGTGTTGGTTACCGGGCTCAGCAGAAGTAAAATGGTCGTTCGGGC
GGGGGACTTACCGGGGTCCCAGCCCAGGACTTGTGGGAGACCAACTCGAGTCAGGTTGTCTCCGCAGC
TAACCCACGAAAATGGAATCCGCTCCGCTGCCACGGAAATAAAGAGCGGGATCAGGCTTGTTAAACCT
GTAGCCTAAACTCCTGCAATTTTGTAGAGGATGAGAGGGTTTTCAACTGCTTCGGTGGTTCTTATATT
CCGGTGCATCTCCGGCCAACCAGTTCGGTATTTGTACCCAGTGCAGCGAATTCATCTGGTTCCTGCTG
TTTTCCCAGACTATAGGTTTTCGGTGCTACGAGTGAAGATCCCTGAAAAGCCAACTATTTTAACATGG
GACTTAATAGGAGGATAAGTTTAGTCGGACGGTTAATAGACTGAGCTACAAAAGTGCTTTTCCTCTTA
CCCTCCAGCTTTAAAGCACAATTCCTAAACTGTTAGGGCTCTCTCCTCTCCAAATTGTTAATTAAAAG
CGAGTGCGTGCCCGAACAATTTGCCAAAAATAAAGCGCCACTAAAACACTGTTGAAGTTACCGGGAAC
CCGTGTTCGGTCAACTCATGTGCACATAATTTCGAATCATGTTAGGTATCTGGGGTACTACTATGAAT
TGCTTAGGTATTCGAATACTACTGAAATTGGTCGTGTCCCTGTTTTGATTACATCAATGGTTGGGTAC
GGTTCCGTGTGGAAACATCAGTAGCAATAAGTCTAATGATCTGGTTGGGCATTTGCCAGTAAGAACAA
TGCATTTTTCCCATGCAGCAGCTATTATTTTACTAACAGCATCAAGACCAAGCCACTCATAAGGCCAG
GTTGATTAGTATAATTTGAAAGTTATAAGCAAAAATGGGTCATGCAGGGAAATTGACCAGGTCGTGAC
CCACCATGGCAGAGGGGATTTGACCATGTGTGACCGGCGGATCGTGCAGGGAGATTTACCCCCGGCTA
CTAGTCAATCTCCTTCCCCCTCGTTTATCCCTTTTATTTCCTTCATAGTTAGTTGGATTTGGACCAGT
TGCTTAGCTTTCACCCATTGACACAGGAGCAAAGCTAGTAGCTAGAGTAAAGGTAGCATCATAGATAT
AAGAATCACTATAAAAGGCATAAGCAGAATACGAGATTAGGGATGAATAACTTGATGATGATCATGAG
CGAGATCTAATAGCAGCATATCCAAACATAAATACAGTTGTTTACCCATCATAGGCATAGGTAGGTGC
TGCATTTAAACCAAAAGCATAACCTACAGCATATCTAGCACTATAAGCATATGTAGCTGGAACAAAGT
AAGCATATTAACCAGATGAAGAATCTTATGCTCATGATCCATCAATAGATCCTGCAGAGACAAAGGCT
ATGGCTAAAGCACTAGTAGTAGATCTGATAGCTATGTCCCTAGTAACAGAGCCTAAAAAAGTAGTTTC

GGTAGCACCCATAGTAATAAAGGCATGTGCATTACTAGTAGCAGTTCCAGTTATAAATAGTAATATAG
ATCTAGATCTAGACCCATATCCATAAGCATAAGTACTAGATCCTATTGATCCATAAGTGTCAAGGGAA
GAAGCAAGGGTTGAAGCTCTTGATTATGATCCAGTTGACTTAGCTTATCCACCTGTAACACCAGTAAT
TAGAGAAGTAGCACCAATGATATTAGTTTAAGCATCGGTACCAGTAGCAGTAATAGAGCCAATAGCAT
AGGTTACAAGGGTAGGTGTAAAGGCAGAGGCAGCTAAAGCATATACTAGAGAAGCAAGAGCATAGCTA
AGAGTAAAGGTATCAGTAGTAGTGGAAGAGCCAGTAGCAAACCCATAAGTAGGAACAGAGTAAGCATT
ATAGCTAGGAGTAGTAACAAAGATAAAGGAAGCAAGGCATTAGACACGAGAAAGGTTATAGGAATAAT
ATACATAACTAGAATAAGCATAGGTAGCAGAAGCTGATCCTCTAGTTTGATAGCCATATTCAGAGCCA
TAGCCTACTACTCCAGTTTAAGATCTTCCAGATGTCAGGCCATTACTCATAGATCCACTCGTTGTATA
TCTAGTAGCACTCATTGAAGTAGTGGAGAAGGCATAGGCGTCAGCACTACTACTAGTCTTATATTCTA
TTAGAGACCGAGTTGACCCAGCAACAGATCGATATGCAGTCCAAGCAATAAATCCAAAGGCATTGGAT
GCTAGGGTAGTAAGGGCTACGAAGGTTGATCGAGTTTATGACCGAATGAAAGTAATTGAAGTCATAGA
TCCAGATTAAGACCAAGATCCGATTCCATCATCAAAAGCAGAGGCATAAGAGTAAAAGCCTATTATTT
AAGACCCATAGCTTTCTACAGATCCGGTAGTAGAGTCCCTAGCTCCAGATTAAGTTGTAGATCTAATT
AACCTAGCAATGAAGGTCGTCGTGGAGATTTTATCTACACCGATGTAACGTGCTTTCGAACGGATTAT
TATATACCGGGTCCCACTTGTGCACCTGGAACTGGATATGCTGCTGGCTTTCGAACTGTTGCTTGCTC
CGCAGGTAGTGAATCAACAAGCTTACTTGCATCATCAACTGGATCTGGATCTGCTTCTGGGGGATCTA
CTTCGGGGTCTGCTATCTTTGCTTCTGCCTCCCTTGCCTCCGCCCCTGTTGCTAGGCTCGGGTAAACT
CAATCAACAACTACTGCTGGATATATAGGGAAGGATATATAGGGGAGGTAAGCTAGACTATCAATGAA
GTAAGAAGCTCAGATGCCGCTGGTGGTGCTTCCTCTCGATCACCTGTATCCAGGGCTGGAATATAAAA
TAGTGAAAGGTAAACGACTCGGGCAAATGCATCCACACGAGTAACGAGGTCATCGTACGGTGAAAGCA
CCAGCAACCAATTTTGAGAAAAACCCATTGCCCATGCTGGCTGCTCTATTCGCCATTGCAACCTCGCC
GTTCTTTACACGCATATCCATCCGATGCTATCCGACACGCCACATTGATTAGCCTGCCTGGACGCTTT
CTATTTCCCATTTTTACCTAGTAACTCTTCTCTACGCACTTTACACGATCAAATCTACACTACATGCA
TTGATTGGTTTGGCCCGCTGCTCTCTTCGCCATTATAGGCAGTGTCCAGCCGTCGCTCTATATATGTG
CATATTATATAACACAATCTATAAGCTGTACGTACATTGATAAAAAGCAAAGGTAGTGAAAGTTGCAG
TTGAATCAGCAACAGAGCGAGCGAGTAGCCGGGGAGGTGGGTACAGAGGCAAAGATAGAGACAAAGGT
ATAAGCACAGGCAGCAAAGATAGAGGGAGTAGCAGAGGTGGAAGCACCAATAACATAAGCATCTAAGC
CAGGAGCCCGCAGAGATACAAAGGCAGATATAGATTGAAATCCAGTGCTTTTTTATTAAGTTGTTTAA
TATCCATAGCAAATAGCGGACCTAGTTCCAGGTTCAGATTCAGAGTATAAAAATCCTAGCCTAACTAG
TGCTCATTATCCATCACCCAGAGAACCGGTATAGCCCGGTGTTATGGCTTTCAATCCATCTCGAAATC
GAGATATAGCGCCAGGGGTTTCGGTCCCGGATAAAGATAAAGATAAAGAAGGCGCATCCACCATTAAC
GGAGCCGTCTCGATACTACTTAGCGCCCACTGTCCAATCATGAAAAACGCAATGAGTGGAGATTACAG
AATCTTCACTTAGACCGGCCAAATCCCTGGAATATTTACGAGCCAATTTATCCGCCATTTAGTTAAAG
CTTAAGCAACAAAGGCAGAGACCACATAATATGGATCTACTGAGTAAAAGGTCCTTGCGGCGATATCT
ACGTTTTCCGAGCGGGTATAACCTAATCTGCACTGAGTTACCAGTAAATAAGCAATTGGCATACCTAT
ATAGGCATGGAGGATTGTTGAATACAGGGATAACACAGAGCTCGTACTGATTGGGATATTCATTTGGA
TATGGGTTTACCTAAGCTCTTACTTATTGGGAAGGTCGTTGCTTTGCTGGTAAGACCTTCGCTATTAC
TGATTTAGATACGGTAGGCATAGGACTTATAGGGATATGGAATTATCGATCTCTTGATGCTCTCATTT
TACTTTCGTTATTGACGTGGATCAAGGCATAGAGATCCTTACGCGCTATGATCTGGACTTTTTCGCTC
ACCATGCCACTGGACATTGAATGAGATCTTGATGCGTTAGCAATTCACACCTTTCATTGCTTCTAGTC
CCGGATCAATTGGATTTGGAACTGGCTCTGCTCCCCTAAGGAGGGATGCTGCGGGCTCGCTTCCTGTA
ATACCATAAAATGTGGGGATATAACTCCTATAAACATCTGCATTAGGACCTCAGAGTCCCCTCTTTCG
TCCTAGTATAACTTTCATTGCAAGTGAAACCCGAGTAGTGCGTTAGACATAAACTCCGAAAGGGGAAA
CCTTAAACCCAAACCCTAACTCCATCCTAGCAAACTCTCTTTTCTTAGAGAATGGGAATAGCCGGGAT
CTTATTAAGGGAGAAGGGCCTCTGCAACTATATATCGAGGTGCAGGTATTCTGAAACATCCGACCATT
TAATCTGAACCCTAACGTCGTTCTAGGTCCTATCTATACTATGATTCGGACCAACCCCTGACCGCGAT
AATGTTACCTACGGCCAACTGCCGGTCAACATTACTCGTTTTGCAGAAATAAAGCTGGTGCGATGTGA
GCTAACTACGTTCCCGAACTACCGAACTAACTAAGATACCGTGGTGAGTGGAGTGCCCAATCCGGGCA
GATCAAGACCCGTAAAGGAACTGCGGAAGCCTCAGAATGTCTGAGGCGGGGACTAATTCTGACTGGAA
CAAGGTTGAAACAAGGTTTCCCGACTGATGAGATTTGTACGCGGATGTCACCATTTCAACATAAGTGA
AGAATGTCGGATCGGATCGACCTCTCCTTTCTGATTCTAAAGGCTAATGACCAGCCATCTATACCTCA
AAAGTAACAATGGTGAGGACTGGGAAAAGATAGATTTGAATTGAAAGACTGCTTTAGTATATTAGGCC
AACCCTTCATTTATTCCAGATAGATAGCCGGGCGCATGCCGGATCGAGTTTCACTTTCGAAACTGTAG
TATTATCTCTACTTTCTGGTCATTCGATCAATTCTGTAGCGGCTCAACATCTATCCCAACCAAGTTGT
CTAAACCGGGTGGGTGGGAGTAAATAGCAACAGAGGCATAGGTGAGGGTGTGATCTGTCAGTGTGGGT
CGAGATGGAGATTGAGTTCCATGGCCCGTTTCGGATCCAGATATAGTTTCAGATTCTCCAGATGCGAA

TGCAGATCGAATTCCAGCAACCGAAGCAAGGGTATACTAAGAACCAACAAAGACAGGTGGCACTCCTT
AATTAAGAATCGTTAAAGACTGCCGCCCGGTTCCATTTCCGGATCCATAGTCTATTCCTGGGATCTCC
AGTGCCAGCTTTTTTATCAGCCAGTCTCGTCTTTAGGTAAATAATGAGTAATACGACTTTTTGCTTGC
ACATTTCTATTGCTTAGCGAGTCAAAGAACCAGCTTCCCTTCACTCGGTTCCAACCCCGGCAAGAGAT
CGAGTGCTATAGTCTGCTGGTTGTGGGTGGGAGTAAATAGTATAAGTTCCAATCTTGATCCGCTGCTT
CCTCTGTACCAACCTTTGTCTCTGCGGGATCAACTCCAACTCCTGCTCTCTATGTCCTTGCAAATGAA
ACTGAAGGTGGAACTGCTTCTTGCTTTGTTGCTGAGTCAACTCCAACTACTGCTACCTCTGCTACTGG
TTTTACTCAAATTATTTGGTGCTGCTTTCTCCAATTATTTATAAAGAACCTCGCCTATTAAGTCGTTC
CGCTGCTGCTTATACGGATGCCGGACTCCTTTTCAATAGTGCTTTTGCTCAATCAACAGGTGCTGATT
CAACTGAAACTGGGTCTGCTCCTCTCTTTACGCCCTCCACCTTAGCCGTTGCTTCCTATTAGCGGGTG
AAACTGTCCGTCTCACTTGTATTAATTCCTTTCGAAATGAAATTTCCTCCGCCTCCTATTCGGATGCT
ACTTCCACTGATGATGGGCCTGGGACCCCGTCACACGCCTATCGTAGCAGAGCGAATCATAGCGACGA
ATCGTAACGAATCGAACGGCAGTCCTGAACGATTATTAATAAGTAGATCGAAAACAGGTTCGCCAGCG
CCAAGCCCTATTATAATAAGTCGGAGACCGTGGCTCACAAGGACATCACGTGGCCTTTGTCACCGTTC
GTATAGATAACTAACCACCCGACGCACTATAATTCAGACTTTCCGTCTCACTTACGGAAGCCTTACTA
CAGCATATAAGCCCGTATCGTCCTTTCCATTTGAGCCCACAGAGAGAGATAGAAGCCGTGGTTTCAGC
ACCAGTCGCATATCCACCACCACCATCATTAGTGGATGCATAAAAGCAAACAGACGTAGAGCTAGGCC
TTGTACTATTAATAAGAACTTGGGCGGAGGCATTTGAATCGGATCTTTACCTTGTTTACTCAGCAATT
CGAAAGCTATCACCTTGCCTAATTCCAATCCTAGATACAGATCGAGATGCAATTCCATATTTAGTGGT
TGGAGGCAGTCTTTAACGATTATGAATATTGGCAGATTCACCAGTCGCGGAAGCAAAGATAGCATCTG
AGCCAGGCAGCCTTGAGCAATATATAAGTTATTAAGGCATCGCTCATTTCGGATCCTCCAGTGCCAGT
AGCAGTAGTTCCAGCAGCTACAGTAGCAGGAATAAAGACAAAGGCAGATCCAATTGATTCGGGATAAA
CAGAAGCAGTAGCATCAAGCACAAAGGTAGGAGCAAAGTAACCTATAGAGTAGGCAAGGCCAAAGGCT
GTAGAGGCATAGGTGGGGGGTAGAGGCAGCACAGCCAGTGCCAGTGAAAGCAGCAGATACGGGATATG
CACGTGTTCCAGGAAGTGTATTTAATACACAGAGAGGGACCTTAATTGGCAAGACAGAACAACGGGGA
GTAGAAACTACAGGGGCAAAGGCAGCCGTTGATCCAGCTTCACAATCAGCAGAACCATTTGCAATTAT
TATATTCAGTCTTTGTGAAAGCAAGAGGCACGGGAGGGACCCTAATAATCACTCGCAGAGGAAGAAGC
AGAGCGCCTAGCTGACCGTATGAAAGCAGCCAAAAGAGAGATTGCGGATCAAGATGCAGTTCCATTTG
ATGGCAAGGTGAAAGCAGTGCTACTCCGCATGCCCAGTATTCTTTGATAGATAGAGAGCCATAGCTCG
TACCTGCGCACAATCTTGAAAGCTCGCAGGTGTAGTTGTTAGCCTTATATAAGTCTTGGTCAGTTTTC
TAGCGGGATCCAGTCTCCAACTAATAGTGGGGTTGCCTGTTCTGTTCGGAGGTAAAGGTCACTCTTAT
GATTGATGAACCCCCATCGGTAACTCTTGCTCTTGCGTTTCCCGACACCTAGGTCAATCAGAACTAGT
CTCATTGTGGCGCAGTAGCTCAGGTAAGCAGAGCCACTTTCCCTACCCTACTGTTTAAGAGCCCGTGC
TAGCAGAATAACCTGTCGGGTGCTTTTATTCGGCTCATTACGCGCTTTCCGAAGCTCCTATGGTTCGG
TTCCTCTAGCCGCTCAGCCTGGCATCTATATCCGTATATGGATCATTCGGCATGGTTAGGGGATCTGC
AGTGTTCCGCAGAAGATCTATCTAATTATTTTCTGCCTGACCCCCGGCCTCTCAATACGTACCCGCGG
GCGGGTGCTTCCCGTCTCGAGCAGATGGAACCTGATCCAATTGTTATTCCGCCACTTTACCCAGATCC
ACCTGTTCCTACTATCTTTGCCGGATCTGCTACCTCCTATGCTATAGTTTCTGAAGGGCTACTATCTA
TCTATGTCTATCTATGCACTAATAGATGCGTAATCAATAGGGTAGGGCTTCTTTATTATAGGGAAAGG
TCTATTATAGACGTATAAAAAAATTTTTCCCGGTTGGAGATCAACAAATAATAATAGGGAAAAGCTTA
AGAGTGCAAGTTCGGCCCTCTCCAAACTATAAATGAAGGTCTTAAGAGCTTAAATGCCGCCCTCTCCC
TACGCCATTAATAGGCCCCTCCCTCCCTTAAATAATAATAAGGGAAAAGCGTAAAAGTGGGTGTATTC
AATAGATGCCTTAAGATGGGCAGGATCTCTAGAAATGAAGTAAGGTCATTTATTATCAAATAGGTGAC
TATAGCTTAGAGAAGGCTAGATTCGTCTTCTGATTGATTGTTTAGCCTAGGGGCATTATTTTTAGCCT
ATGACACCATGTCTCTCTCATTCATGGGTTCTCTCTTCAGTTCAAGCGGAGAATGTTATCACTTCCCG
GCAGGATTTCTCTATATGAGGATCTTAAGAGATCCCAAATTTCAGAGCTAAAACGACGCTCTAGGAAG
TTGGTTGCAGCAACATGACCGGTAGGAAAATGGGCGGTCATTAGAGATCTCAGTGCCATCATCCGGGA
TTCCCCACTCCCTGGGTATTCCCGACCTAGGTTCTATAAATCACGGGCTTGATGGAGGCGAAATGGCT
CGGGTATTTCAGTAGGGTGTTGGAACGATAATGAATCATTTTGGTCATTGTCGGGCATGATTTACGAT
GTTCTTTGTTCATTAGAAATCCTCTTTATTATTGATACCCATGTCAGCCCTGTCCGGCCACTGCCATA
TATAGCAGTTTATCACTGGATTTATTTCCGCTTGCCGATAGGAGACCAGATCTTAGGTGGTGTCAGAG
GTTTAGGAGCGATGGCTTGTCTAACCAGGGATTCCCTCTAGGGTTGGGGTTCTCTGGTATCGTCGAAC
GAGTTTGCCAGATTCATGTGAATTTGGATTAGCGGTTTAGCCACTCTCCCTAGAGATATCTACATTGC
AGTCTACTACTTCCTCCTAGCCTCTTCTAAGTTCGCTATTCACAGTGATTCAGTCGAGGGCCCATACC
TGGATTTGTATGCTGGCATCATTCAGTACTCAGTGTTGGGGAGGTCATCTTATTGGGTGACTTCAAGG
CCTGCACCAGAGCTCTCCAAATTCCTCTCCATGACCGCTCTCAGGACGTGTTCTGTGTTTAGGAGATA
GACCCAGATTTAGTGGGATTTCACTAGATGTCTGATGATGTTTTGGGGCTTAATAAAGCATATGGGGA

AAAATAAAGGGAATGGCAGGCACCTCCTGCGCCTTGGAGAATCTCATGAGCTATTGATCTTGAACGGT
CTCCCATGCCTCTCAGATTCTCGCTTCTTTGCCTACCTCATGGCAGAGGTGCGCGTGTAGTGGATTAC
GTCCTATCTAGCCAAGACCTACTTCCCTTTATTCGTCACCTTGGTAAACCCCATCCCCTTACATGCTC
TTCTCTCTTTCTCCCTTTGGGGGGACCCCCCTGCTAAACCCCTCCTCCCCCGGGCCCACCTCGGACCA
CTATCCTCTTTGATGAGGGGGACCTTGACACCTACTTGGGGTTATAGTTCATAGGACTTCGCTTTAGT
CTGCGGCATGCCCTTCAGCCTTGGATCAATAAGGGCTATTGATCCTTAGCCCTTTTCGAGATAGTGTT
TTTGGAACCACTTTAAGGATATATCGTCCAAGATGTCTCTCATGGACACCCTTGTTCGGCCTACAGTT
ATCTATGGTTCAAAGGTTTGGGGACTTGGTTTGCTAGAGTCGGATTGGGCCTCAATAGAGAGAGTGCA
GTCCTTTGGTGCATCATCAGATGCAAGCAGGCAGTCCCTTAGCCCATCATCCTTCAGAGTTTGGTGCC
TTGTCCTTCCAGCTTGAGATTGTGTTCAATCTTGTATCACTCCTACATCGCCTTTAAATATATGCTGA
CTTGACGAAGGGACGAGAGCAGTATCCATACTTGGCCTATTGCTCTTTGGAGTCCATTGCCTTAGCCA
ACCCTCCGAGTTGTACTTGTTGCTGTCATGCGGGGGTCTTTTATCTTTTGGAGTTTGTAGGCATTGCG
ATAGACTGGCTTCCTCCATTTAGATACTCTCTTTATGTGCCTGGCCACCTCCTACCTCTTAGGCAGGA
GTTTATCAGGATCAGCCGAGATGATATTTATAGGTAGTTTGTACAGGTCACTTGGAGAAAATCTAAGG
GGGCACTTCATCTGAAGATGGCAATCTACATTGGGCACTTCATGGATTTGAGAGACGGGCTCATTGTC
CGGCCCTATTACACACTCTACCATTGGATTTACGTTCTTCGTATTCCCCTTGGTCAGTTTAGAGTGGG
GTCACATAGACTTCGGGTAGATGTAGATCACCAGATTGACCCCTTAGATAGGATTTGCCAACTGTGCC
ATCTTTGGGAGGTTGAGACTAAGGAGCACTTCATCTTCCGTTGCCCCATCTACTACTATGAGATCTTA
GGGCGGTTCCATTGTCATTAGAGAGATTCAAACCTCTCCAGATTCTTCAAGTACCTTGACTAGAGATG
TCTGGCCTTATACATGCAGGAGGCCTTCCAACTCTGCTCCATTACCATATGGCCACATCCGGGACTAG
CTTCCACTCAGAAATAACTTGCTTTTTTTACGATGCTCCCATCTACTAAGGCCACCAAGAATATGGCC
ACCAGGAAGAAAGCCCTTTAGGTTAGGATTCAGAGTTCAATTAAATGCCCTGGCGGAGAAGCAGAGAA
GCGGTGTGGGTGCTCTCCCTCCTGCCCAGTCAATATATCCATCCAGTAAAGAAAGCCCTAGTGAAATT
GTGAAAGTTGTACCAGGACAACCAATAAATCAATCCTGATAGTCACATACATAACTCACTCTATTCAC
TCACTAGCGCTCGTGGATGCAGTCTCCTCGCTGGTGTTGGTGCCCTCCCTGCCCAGTCAATTTATCAA
GGAAGTCTGACTTGCCTTGAACAGATGAGCGTGTTTCTGACCCTTGACCCCCAAAGGGAGAGGGGAGC
CTAAAAGGGTTACGGCAGCACTCGATTCGAACAACCCTTGAGCTACAACGAACCTAGTCTTAGTACCC
AGTAATAGCCAGCTTGTCATTGCCGAGAGAGATACAGAAGTGCTTGTTTATAGCCGCTAGTACTTGCC
GGGAGTCCTTTACAATGCTTGCCTTTGCTACTGCATTAACTCTTGGATCGACAGCGTGAGGCATCTAT
TCCTGGCATCAAGTGCATTGATACGTGGCCTCGACTAAGACTCCTGGTAGCAGCTATTGGTGACCGGT
TGGGAATGAAGGGCTATGGGCTGTCAATGATTGGCGTATAACCCTTAACAGGTGCATCGGTAGTAATG
CATTGTTCATGTCATCCGATCGAGGTAGGAACTAACCGTATCGTTCAGTGTGGAGCAGGTAGGAACCA
TTGTTCCGTATGTACAACCCCGTAGGGTCCAGTCTCATGGGTCTGTCAGTGCGCCTATGATCGGTAGC
TCTGATCGGCAAGTCCTCCCGACAAGGCTTCCCCGACAAGCACTTCCCGTCTCTCTCTCGGCAATGCC
AACTGGGTATCGACAAGGACTTCATTCAACGACTAGCGACTATCAGCAGCACTGACCGTAGCTTCTAG
TCACTGAGTATCGAGCCCAGCATTTGCTAACCGAGTAGCCCAACCGAGCAGCCCGGAAGCTCACCCGC
CATCCAGCTCCAGCTCACCATCCAGCAAGCTAACCAGCAGGCCAGCAATAAGCAATTGGATTTGTTTG
GTCAAGAGCCGAACAATTGGAGTCATTATTTATGATTGGAGTTAAGTAGTTGTTAGTGGAGTTAGTTG
GTCTCCAGGCAAGTAAGAAAAGCACCAGCAAGTTTTAGTTGGGTAAAGGTTTCCAGGCAAGTGAGTAA
GCTCGCAAGTCCCAGGGCTGTTGGTTCGCTGCTTTCTTTCGGTTCGCTGACAAGATCAAATATATCAG
GTAGGCGGGACTGTGGCCTTGAGGTTATATCACCTGCCTAGGTCTGTTTAGCGTAAGTAAGATCTATT
TCAACGGGCAAGCAGCAATATCGCTCCTGTGTCCTATCGGTTTTCAAACCCTAGGTCCTGTTGTGTTC
AGTTCTTTCACAAGGTATCGATCAGCATTTCGTCATTCCATTCCAGGTAGGTGAGACTGTGTCCTATC
AGTTTTCACATCCCTAGGTCCTTTTATATCGTAAGTAAGCAAGAGACTCCCCCTTTGGCAAGCAGCAA
GTGGTATCGATCAGCAGGTAGGTGTTATTCGCAGTGTCTGTCGATGCTTCCGCCCCGTAGCGTAGCGT
ATCATCCCCGTGTTGATTCGATGTGCTGCTTGGCTCAATCTTCTCCTCTTCTGGTGAGAGCGTGGGAA
ACTCAATATCTAGGGTATGGGTGTGATGTTGAACGGGGCATGGTTGTTTAACGGCGGGTATCCTTCCT
GTGACGGGGGACTTCTCTCTCGAGTATGTTGTGATGGTGACTCGGTTATCCTGTGATGTTGAACTTCT
CTCATTGTCTGGTGGGACCATTGATTGATTTCGATTTCCATTATATATGTTTCAATGGTTTGCGCCAT
ACTGCCTCGAACCGAACACATAAGCGCAGTAATAGGAGCACAAAAGAATTATATGAAGATGAAACGAA
ACTAATGAAGAAGCAAACCACAGGACCCATTAATGAGCACCCAGATGTAGCAACAGGAACGGAAGGAA
ATAGTCGATACTGTAATGGATGGGAACATAGACGATCGGAATATTGTAAGTCAGCTGGTTACGGGAAG
AAGAGAAATTGGTAATGCGCATGAATGAGTCTTTCTCTTTTCCTGGCTCCCTATGAAGGAAGAATCTA
TCTTTAAGGCCCTGCCTGAAAAACGGCGTGGTGGGAGAGGCCAATATTGCTTTAAAGGCAGTGGGCTG
AGGTCCTGTGCCGGCCTTCCTTCCTTAAAGTGCCAACCTTCCTTAGTGAGTTCGGGGTAAACCCGCGG
TATAAAGTATAGAACAGAATCTCTCTTGATGTTCCACTGAGCTAACGGTCGGTTGGTCCTGATTGGTC
CTGATTGGTTTGTTTGATTAGGGGGGGCCTTTTGATTGATTGGTTCTGATTGGTCTTGCAGCATTGGT

TGGTTTCACCCAATTTAGTAAGGTCATGGTTAGTTAGTAAGGTCATGGGTTGATTCCGCGGAGCAGTG
TTCTAGTAAGGCTGCGATTTAGGGGGTGAGGGGAAGGGGGGAAGCACTAAAAGTGAGTTACTTTAAAA
CGGTCAGGGGCAGGTCATGACTCCACGGGAAGATATGATCTGCTGGGGGGCTTTTCATGCATGCCCAA
TCCCGCCCGCCAGACACCAGAGTTGGTCCTGACTGAAAGGTAAACCCGGATTTGATATAAACCCCTTC
CTTATTTCATGCATGAAGGATTGATCTTGAAAGGTGAATTGCCCCCACCCTTCTCTGCCGAATGAAGA
GTTGGCCTGTTCATTCTTCCAGTTATCCTTCTTTATAAACGTAAATACTTCGATCAAGGTCCTGAAAT
AGCATCTATAAGATGGCCACCACTGGCTGTCTGTTAAGTGAGGGAGGGCGGGCGCCATATGTACTTGG
CGTGCCTGCCGGTCAAATTATCTTATGAACCAGCACACCCGGTTCGGGGTTCGGGTCCCTGTTATTTG
TCTCGTCTATGTTTGTTTGATGAGGGTGATCCGATTCAGGAAGGCCTGACCAGAGTCCCTCCTGGCTG
TAAAGCCCTCTCCCTTGATCCGGTGAACTAAGGTAAGTAAGGTAGAAGACCAGCCGTCTATCTAATCT
CATGGTGTGCGCAAATTAGGTGTTGGTCCCGCAACTTAACTTATCCGACCGAAAGGCTTTGGGGGAAG
AAATATAGTGGCGCCCAGTCCGTCCCTTCCCTTGAATCCCTTAGGAACTAGACCCTACGGGATGGGGG
GGGATCAACCGCAAAAAAAGAGAGATATCATTCTTTGGCCAAGTATCTAAGTAATGTGCCCGGAAAGC
AAGAAAGCCAATAAAAGCCAGGAAAGCAAGCGGCCGGATTAGTAACCCGATACCGGGGTCAGGAAAGT
AAGGAACTAAAGGAAAGCGCTATCTCTCCAGGGCATCACATCAAGAATCTCGTATATAAGGGGCTCTC
TAAATGAATGATAGAATCCTTATATATGTAATTACGCTTTAGCATCGTACCCCCGAAGAATAAGTAAA
CCCAGATGCTCTTATGACATAATCTATCGTACCAGGTATATAATAGGGCTACCAGAAAGAAAGAGTGA
AAGTCCGGCATTCGTAGATTCAGAGACCCCCTGTGCCCCAAAATACTTTCCTAGGACCCCCACTATAG
AGGATAGGGCCCTTTATATAGTAGGGTCTATAGAAAGGGTGGATAGGGCGAGATTCCTCATAGCAAAA
GATCTGGAAGAGGCCTTCAGCAATCCGCGCTTCGTTCGAACTGGATAAATGTGTTTGTGCTGCTGGGA
TGATGGTATGTTTCGGCCAACCGACATGATCTCGTCTATACTAGATCTCGTTTCTAGGCACTAAGAAA
GAAGGGCGCCATTATAGAGTAGGGCCGCGGCCCTTTATAGTGGTAGTGGGCTTTAAAAACACCTACTA
TGGATAGGGACCCATTCCGATCTATGGTAGTACATGGATGCTGCCACTGAGAACCGCGAAACTAAGCT
GCGACTAAGATTGTTAATGACCGCTCTCCATCTCAATCCCGACCCAACCCATCCCATTATCTACGTTC
TTGAAGGCATGGGAATACGAACCCCTATAGATAGTAGGGCAAGCGAATCATGCGACTAGTTTCGTGTC
TTTCGCTCGCAGGGGAGTGAATGGGAAGAGACGCTCCCTGTCGCTATCTGTGCCTGACCGACCCGTCC
ATAGAATGAGTGGTGGTGGTGCACCGTGGTTCGCCCGGAGGGAACCTATACCGATCGGAAGAGAGAGA
ACGATATAGAAACGATCGATATAGAGTGGGGAATCACACTTACTCCTGGGGGTAGGGGTAGGGCGAGA
TAGGGTAGATAGCGCCTTCTCTACGTTTTTTCCCGCCGATCCACTTCAATCGCTAGGACGTAATATGA
CCGATGACCGGCATCTAGTTAGGTCTTGTTTGTTACCGTGTAGGTCCGTCCGATCGAACGCTAACCTA
ACTATATGCTTATGCTCAACACATGCGGCCCCTACTATCTATAGGGCCCTCCCTCTTCATATCTCCGT
GTTCGCCTGGGCGAATCCCTAGTGATGCCACCATCTATCTATCCATGCCGATCGAGTCAGTCTCTATT
CCGTGCCTGGATCAATCTCGGGCTAGATACGAACAAGTGAATCGATTCGGGAATGTATCGACATCTAT
CTTGTCTCTTGTTGGCCCTATGGGAATTTCTTTGTTTTTGATAATACCAAGATCTGTTCTGTCAGCAA
GTTTCATTTTAGGCCTGCCTGTGAATGATGTATGGGGCAGTCCAACCAAAAGCTCTAAAAAGCTATAT
TGGAAGCCGGTGCAAGGCTGTAAGGGCATAGTAAGTCGTGAGGTTGTGTAAGCGGGTGGATAGTTTCT
AATTGTAAAGGCAAATAATTGAATTAACTGGATAGGATAGGAAGAACCATACTTATTCATTTTAGGAC
CTGCTAACCAGGCGGGCACCCACCGGAACAAATATTCCTGGTCTCCCTATACAACTTGAATGAATCGC
TTCATGGGGACAGCTATGAACAACTTAACGCCTACCTTAGCAGTAGAAGATTGGGAGCCTTTACTCAC
AACCAACAATAGCCTCTTCTTTCATGCTGCCCAACCTTATCTTCGAAATCTTAACCTTAAGAGAAACC
TGCTTGAAAGATTCATAAGGCAAGGTAAGGAACTTTCAAAACTCCCTCCAAAGGTGCATTTTATAAAG
ATTGGTTTTGGGGGGTCTGAAGGCTATTATTCTACTGGGGTAACTAATTCACATTTGTATTCAGGCAA
CTTGCCTTGCTGATTGAATACATTTCAGGCAGGTTGCACCCTCCTTTCGAAAGGTGACAGCATAAGAG
GCGCGCAGCGGTGAGGAGCAGAGCCAAGAATATGAAACTAAAGATTCCGGGTCGAAGACGCAAATATT
CAAATATAGCATATATAAAGAATCACCTAACCTCCTTATATAAAACAAAAAGGTTCGGTGACAAGGTC
AGGGTAAGGTAGTGTCATCTGATCCCGGGTTATAGCTGTAGCTCCTAAGCATTAATTCATTCAGTGTG
ATAGGGCTAACAGGTGCTAGGTCTTCAGGTAGGGTAGTTAGCAAACACTGAAGTCAGGGGTAAAATAA
GGGTCAGGTTTTTTTATGCCATGCCGCACCCAGAGAATATAAGAAGGGTTAATAGCTCTACCTCCTAG
GCGCTTTCATTCAGTTTTGGTAAAAGACTACAGGTCTAGGTCTTCGAGTGGATAGCAGGTATTAAAGG
CAGGTAAGTCTCTGCAGTTCTAATCGCTGAATCGTATTGGCTCCTCGATGGGGAACAATAGGTAAGGT
ACCAAGAAGGAGGGGTACTACCCATAAGAATTAATAAGAAAAGGCATTAAGTAAAGAGTGCCCGATAG
CATTCCATCCATACTCATCCTGTACTGATCTTTCTAGTGGAATGAGTTGGGCAGCCCTCCACTCCATA
GACTAGGGTGATAAGTGAGTAATCAAGGTTTTATTCTAATTGCTTCATCGTCTCACTTCGTTCCTAAG
TAGGATAGGTAATAGCGGTACCCAGACGGTTCCCGGTTCATAAGGGAAGGCAGTAAGTAAAGTCTTGG
ATGCTCATAAGTAAAGTGTTAATAGCAATACCTACCCTACAAGGTGTTCCTGGCTTCATCCAATGGAT
TGGTAAGGGCAGCACTCAATAAACAAAGGTAGGGTGAAGTCAGTAGTAAGTAAAGTCTGGTAGAACAG
GTTGTAGATGACGTAAGTTCAGATAAGCATATATTTCCATGCCTACTTAAAAGACTTTATGTCGGGTC

GGATGGACAGCTCACTCTTCATTGCCTATAAACTACAGCCAAACGATCTTGCTTGGAAGGGCGGCTTG
CTTGACTTGAGTAACTGTGAGCGAACGCGACTCGGAACCCCACAACAAACTATCCGACCGATAGTGAG
CCCTTAATCCGGGTACTTAATCCGGGTAGTATCTTCACCCATCGGGTGTTCAGTGAAGGTAATAGCCC
ATCTCACCCGGGAAGGATGGGTAGGAGGTGTTCAGTTCAGGCGGCTGTCCATTATAATTGTTCACTCA
TCGTTACAGCTTTCTAGATGTGGACCACGAGGGATAGTTCCATTTTCTTTATTTTATGTTGGCTGGAC
CGTGGGTGGGAGGAAAGTCCTAACTCACTTGATACCGGTTGGCCTAACTAACTATAGTTGCTGTCCGA
GGGCAAGGAGAACTAACTTGAGTTCAACTATCTTCCCTTCCCTGTTCGAGCATCCCTCCTTAATCCGA
GCTGAACTCCTGAATCCGCCGAGCATCAATCTAAACGAGGAGCTCCCGGGGGAGAGGAAGGTCATTGC
AAAATAGTTGGTCATAGTCGATAGCCAGTTGTCGCTGATAGCCAGTCTCATTGCCATAGGGTCAGCTG
ATAGCCGATAGTCAGTGCTTGAAGGTCAGTGCCACTCATAGCCACTTGTCATAGCCGATGATTGCCGG
GAGTCCTTGCCAGAAGTCATAGCCAGAAATCATAGCCAGGAGTCATAGCCAATCATATTGCTCCCAAT
CATTGCCAGAAAGAAAGGTCATTGTCGATACCGATCATTGTCAGCCGGTCCTAGTCAGCCAGTCATTG
CCAGGAGGTCTTTGACATAGGGTAAGCCCATAGCCAGTGGTCATTGCCAGAAGGAAGGAGTAGCAGCC
TACATAGCCCCACTACTCTACAAGTCAGACAGAGCAGCAGCCCCACAGCCCACAACATAGAAGCAGCA
GCCATACAGAGAACTCGACCCTCTTCGGAGGAACCAACAACCATAGCCCTTGACCCTCAACCCTAGAA
CCTCTACCTCGACCTATACGGAAACCTGGGTGGTTAGAATGTGCTCACCTGGGCTAGATGGATGGAAG
GAATAATTACGATGCTTTACATACATTGATTCACAAGATGTAGGTTCATACGCCAGTCATACATAGCG
ATCAGAGCCAGTCATACCAGCACATTTGCAGTCTAGTTTTGAGGTCCCTCATCTCTCGGCCTATTGGA
TATTCTGATTGGCCTAATGAATCGGCTTTTCGGCCTAAAAGCGTGGGTAAGTGCTTGCCTACCCTATT
TGCTTTTTATCTTGCCTAAGAGCGTTAGTGAGGAGCTTAACTGTCTACCTTCTTTTATTTCTCGCCTG
AGTGAGGAACCTGACCTCAGAGCGGCTTAACGAACCACCTATAAGGCAAGGAAGGCCAAGGATAAGTA
GTTAGTATGAGCCCCAGCTTGCTCGACAGTAGGAGGGCAAGGAAAGCTATTTTCACCATTATCCCGGG
AAAGGAAGGTCAGTCAGTCTCTCTAGGAAGGAAAGCAAGTCTCTTTCTTATATAGGAAGACCCGTCTC
TATATCTCTCTTATAAGGCCACTTCTATAGCTCAAGGCCCATAATTCACCCAACAAGTGAAACAACCC
CTTACTTAGGTGACGTTCCCATTTCATAAAAGGATTTGTGAAAGAAAGCCCAGAGACAGGGAAGAAGG
CGACTATAGAGAATAGACAGAAGATCGGCTTAATTGGGCCGGCTTGAATAAGTGCTTTAACCTGTTCC
GATTCATGTTCCGAGAATCGGAGCATACTTGGACTCTATCTATTCCAATTCATGTTCCGATAAGATAA
ATACTAGCCCAGAATAATCAGGAAGGCCAGTATTTGGCCGGACCTGTTTCTCTTCCTGGCTCCTGCCT
AACGAAGATCGTATATAACATTTATTGTAATCAATAATGGAGGGAAAAAGTTATAATCTTATGTGGAA
ACCTTATGCGGCAAATCGATACTTCTATACATCCCTTAGACAGAGGTAACCCCACCTATGCTATAGAT
AGTATGCAGTTAACGGAACCGGGTAATACTTAAGCTATTCCTCGTGCAGACTGAGCTATGCGCTAATA
GATAGAGGGACTTCCTACCTTGCTCTTCCTTAACTATTTCTGGGGTTAGCCTATTAGGTGTAAGGTTT
CCTAAGGTGTCTGGAAGGATAATCGATTTGACGCATGCCGATAATCAATATGCCTATATAAGGAAGGA
AGCACCCCAACCTTTCAGGGGATCATTCCTCAGGGGCATTCATCCAGGCCTTAGCTCAGCGAGGAGCA
CTCCTTCTGCCCCTTAGCCAGAGGTTACGACACCTATAGTATAGATAGTAGCCAGTTACCGGGACCGG
CGAAGACTCAGGAGGAAATCCGGTAAACCAAAGGTAACGTGCTTCCCTTAAAGACAGCCAAAATGACA
TATCTAATCGCTGGTAAGACGGCATAAACCTAGCAACCAGGGCAAGGGATCAAGCTTAGCAATCGCAG
GGAAGGTCCACATCGGCTGCACATGACAATACAACGAACACTTATGTGCACACACATCTGCACCCATA
ATCCATTTGTGCACTCCCAGGCTTGCTATCAGTGTGAGCCTCTAAGTTGGTAAACCATCGAAGAACAC
AAGCAGAGGGAATCCTAAAGAACCAAAATAAGCCATGAAAATGGAGATTCTTTAGGGATCCATTCTAT
ATTTAGTATATAGGGACGCAACCTTGCCTTACCAACACTCCCTAACCTTACCGGGCGGGAAAAAGCTC
TAAGCACAAGGCTCGAAGAGATAAGCCTAAAGAGAGATAAGCATATCCAGCAGCAGAAGAGGGAGCAA
GGACAGCAGAAGTAAGGGTAGCTGGAGTAGCGACAGAGACGGGTGATCATATAATCAAGATTGAAACC
CAGCTTCTGAAGATACAGATCAAGACCCATATCCAGCAGCAGTAGATCTTTCTTAGCTATTTATTCTT
CAGTTGGTTATGCAGTCGATCCAGCACTAGCAGCAGTAGATTCAGACCCATACCCATAAGCACGAGTA
GCGTAGCAGAAGTCGCGTAGCACCCCGACCGAAGGCCCCAAGGAAATAAAGACCTAACCCCCTAAGAC
GAGGCCTAACAAAGCCACCTAGGGAGAGACAAGCCCCTTAACGAAGTATCCCATAATGCTAGTCTGGT
CTGACAAGCAAGCAAGATAAGGTGGGGAAATAACGTAATAGATAGATTGAGATCTACACCTCCTTACT
TCCTTCTTTCCTTAGACCCACGCCCATCTACATCTTCTTATCTCCTGAGATCCACGAAAGCCTTATAT
ATGGGATAATCACACTTAACGTAACATAATATAAGGAGGGGGAAGGAACCTTTGCTGAGCTCATCAAA
GCCCTCAGATGACAGCCAATAGATACTGATACCTCTTCACATGCTATCCCGGCCTTGGCTCCCTCCGG
CTCCATGCTCGGGCCTTTTCCCTATCTATATGCACCGAGCAGAGTAGGGGAATTCGATAGACTCCCCG
ATCTGTTCCTTTCTTATTTTGGCTTGGTTGCTTTTCTTTCTTTCCATCCCCTCTCCGACCTTCCCTGT
TCCTATCTCCTCCTATATGATAGCATGGCTTTTTCGAGCTACGCGAATAAAAAGGAAGACCTTATGCG
GACTATCACGAACCTATCACTACGTGGCAAGTTGCACCTAGCCACCCAATCGCGAATCGATCAAACTT
CCATCTCTATGTTTAATCAGCATCACAGCCAGCTATGTCATCAGAAGCAGGGCTTGTTGTTTTCCCCG
TCGGCCTAGAAGTAACAGGGGCAGTTTACTAAGAGTTCATTCCACAAAAGCAAGGGTTAAATAACCAA

TACCAAAGGCAGGGGTGTCGGTGCCAGAGGCCATTGGACCAAACCCCATTGATTCAGCCAAATAACCA
GAAGTAGGGGTGGCAAGGACAGGGGTTGACTTGGTTGAAGCAACAAGGACAGAGTCAAAATAAGGGGA
GCCCACAGAGCCTGTTAGAGCCCGCCTGTCTAAGACCCTAAAGGTTAAGATGCTGGGATTTACTACGC
AACTGGATCGATCATTGCATTGCGTAGAACCAATTATTCCCCTGTCCACTCCATTATAAAGTGATTGC
ATAGCTGGTATTATCACTTAGTTGGTCTCATCGCTCTACGCCGTGAGCAATCAATAGTGTCGTACAGG
GATGGCTGGGTGGTGTTGATTCCGAAGAACGCACTAGTGCTGTTGCAGATGCCCGGAGCTTCCTTAAG
TAAGCGCTATGTATGTCCTACCGTATCCTTCGCTTTGCACGATGAAGTGGGTTAATGCGCTCGCTTCT
GCTCCACTCATTGATTTAGTCTAGATCCCGAGCCGGGTCAAGCACCTCAAATAAAGTCATTTAGGAAG
AAGTTTTAGAACCGGGAAAGTACTAATATCCAAAGCCATATCATGCATATCATGTGCTACCGAGCAGC
AAGCTATTAACCGTTAAACAAGCGAGGCAGAGATTCAGTCACCCTTTAAGGCATATATATGACCCTTA
GAAAGCCAACCAAGCCATCAATATGCAGTTCCGCAGCTATGAATTAATTATAGAACTTGAACGATTCT
TAAGTAGTCGCGATATGTTAGTAAGGAAAGCCAAGCCCGCGGCAGTCTTGAACGATTATTAATTTAGT
CAAAGCAGAGGCTTCCCTACTCAGACCTATCTCTATCTCTCCTCGAATCATTGTGCAATGTCCTAATC
GAATGGCGCCTAGATTGCGAGGAGTAAGCTATGCCCATAACAATGTAACATTTCTATCCATGTCCTGT
GTGAAACGTAAATCTCTCATTATAGTTATTAAGCGGTACTCCGTTCCGGCCGGGATAGGGGCTAAGGT
TGATGCGTAAGATTTACGACCAGAAAGCACCTTATTAATGAACGGAGGCGGGTAGTGAAACGGAAAGT
CCAGTGCGGGACCTATTATTTACCTAAAGACGTCAACATTTATGGGAAAACTCGACGTGCGGCTTTCA
CCTGCTGATAGTTCGCTATTAATAAGCTGATCCAATCGGGCTAATATGGATATAAGTGGTGTAGTAGA
AAGCGAATATAGTGCAGGCGGCTAGATCACTTTGATCCCCGGCGCTACGGAATTAGGGATGCGCGTGT
TGCTACTGAAATAGGTGCTTAAATGGGTTATGATTCAACCTAGGCTATTAGCGTCATATGGTTGGCTA
TAGGTCATTGATAAGCGGCTTGTAAATGGCTTCGATGGCTAATTACGTAGTATAGATTGATTGGGCAT
AGGTTGGATAGCGTGGTATAGAACTACGGTCACATATACAACTAGATCATGGGTTGGATGGCATTGAA
CGGGTGAACAGAGAACAGACAACAAGGTAACTATTATCTCGGAATATGTGCATTTCCCTCCTGAATCG
AGGATCCATTTGCAGATCCTCTAATATAAAAGCCAGGGGCACCGAAGGCAGAGGTAGAAGGAAAGGCA
GGAGCAAGAGACGATATGGATTAGATCCCACTATGTATGTTCCACACGCGTGTAAGCGCATATCTTAC
AGACTTTTAAGTATTTCCACATTATAATTGGTCCTTTGTCCCTCAATACGTTGAAGGACTTATGGATG
ACTCCTTGTGTCGGAACTTAAGACTTATAAGTATTTCCACATTAGAATTGGTCCTTTGTCCTTCAATA
CGTTGAAGGACTTAGGGGAGACTCGGGGGGAAAACTCCTGTTTTGGAATCAAAAATTTGCTAAATCCA
CTAATGAAACTCGATAATTATAGCCTTATAATTTCTTCTGTGGTTGTGGACTTAGATATATTGTATAA
TGTCCTAGCTAAGTTCACTTCTTTTTATCAGAAGAGTTATCTTAGATGGAAGCTCTCTAGTCTGTTTC
TAGAGCCCTCCACTATCACTATGCCGATTCAAATTAGGGGACTTGCAGGCTGGGATCGTGTTCTTCGA
AGGATGATTGCGGAAGATCCCTACATCTTATTTGATATATCCCCTGAAGATAGGCCCTCTATAAGTAG
AAACTATAAGCTATTAAAGAGGGGGTACTCTGATAGAGTTAGAATTAAGCTCAATAGTAGATTGCAAA
GAAAGCTAATTTACTATCGTAGGAAACACAGAATGCTTTGGGTCGTCGCTTACATAAAAAGTGTGATA
TAATAAGATTCTATTCTTTCTTTAATGACAGATAGAGACTCAGTCTTACTGTATATCCAGTGTCACTT
ATTATTTCTAAATCTATCCCGTCTCATTACGTTTACTCCTTCCTCGGGCTCCGCCATCGCACTAAATC
ATTTTCATCTCCGGCTGGTAGGGTGGAGGCGCACCATTGGGACAGGGTGGGTTTAGGACCAACGTGGG
TGCGATGTCCGAGATGGCAAAAATCATGCAAAATAGGGAGTTGGTCTTAAAACTTTACTGTTTAGAAT
CCATGTTGGAAAGGGATACTAATAGTAAGGTCATTTATATCGTACTTGGTCCGATATTTCCCCTGTAT
TTGCGGTCGATAGGTTCAGATACTTTAGGTGCAGAATCCAATAGGGATATGGCCATGAAGTTGGGTCC
ATTGAATGCCAGCTGCCTGGTTCTTACTTGTCAGTCTGAGGGGGATATGAAGACCTTTCATATCATAG
CTAAGTATAAGGAATCGATCATGATAAGTGAGATTCGAGGGAGGATCTCTAGTCTGTTTCCCCCATCT
GTACCCGTTCAAAAAATTCTGGGACTTGAAGGCTGGGATCTTATCCTTCGAAGAATGCTTGCATTTGG
TCCCCTCGTGGTGAGTTTATTATATGTCCCAGATAAGGTTATAATATTTGAAGTGAAGAATAAGAGGT
GGTTTGATAGGATTAAAAGGAGTTTCACTTCCTTTCGTAGGGGTTGTCCCCGCTGTGGTCGTAGCTGT
CGTTGCTTAGGAAAAAAGCGTTAGCGTCCCTCTGCCTTTAAATCAATCAAGTGGTATTGGCATCTAAT
AAAGAACAGATTTTCTCCCAGCTGCTGAGTCATCTTAAACTACTTATTTATTTCTTTTGATAAACTGG
CTGTGCTTATGGGTAAACTGGTGTTAGTCTGGTTATAACCTTTTCCTTCCTCTGTCCGGTTAGCAATA
AATCCCCTTAGGTCTTCCTTTGCGTTATGTCTATCAGAAAGCGAGGGGTCAGTTATATTGAGTAAAAG
CGAGTTTCCAGGGTCAATGCTCTGGGCTGATTGGTTCTGGCTCTACAAAGAAGAGCCAATCAGCATAG
CCTGTTCCTGGGCCTGCAGAGTAATCACTGCTCTTGGCCCTTTTTCCCTAGACCTCGGTTTCCTATTA
TTATTTCTGTTACCTATCCCGTTACCTATCCCGCCCGTTACCTACCCGGAAACCTACCTATTGAGTAC
CTTACGACGACCTTAAATCCCAATTCTTCTGGTTTTTTTCCTTTTATTCACGCAGAATCTAAGGGTTT
TTTCCCTCTCAGGCTGGATGGGTGGAGGCGCACCATGGGACGGGGTTGGTGTTGGAACTTATTATTAT
TAAAGGCGTGGGGTGTGAAGCCTGAGATGGAATGTCCTTAGATGCTAGAGAGGAGCGCTACCTGGAAA
GTCAATCAATCCCTCTCCTTTCAGTAAAACAGAATTCCAGCCTTAACCTGTGTCTGGGGAATAGAACT
GAGCTCTATACAAGGAATTTATCTTTGGGTGGAATAGAGATAGGGCACATGAAGAGGGCAAAGGCAAG

GTTTGAGGCCTTCTCTTTGGGGAATTTGCATTCATATTATATATGTGATTATGCAAGGCTCCTGCCCC
TAAGCCTTTAAGGAACTAGAAGCCTTACCCTGAAAGGGGACTACTGGTAGGGTAGCGTTAGCGTCCCT
CGCTACTCCAGTCAATCAATCAATCTCCTTCCTTTCAGTGGGTCCCTTTAGCTTCTAAGCCCTGACCT
TAGTTGAGGCAACCTAGATACCTT
>Repeat_11
GCCCAACCCAGTCCCCCAGATAATCTCTAAAGCTAGGGTTGGCTCCTCTCAGTTCTCGTCCATCATCC
ACAAGTAGTGGATCAAAAGCAGAGCTATCTCCCCCTCTCTCTCCTTCATCCCAATTGCACCGGACGAA
CTGGTCCTCACCTCACCACACATACCCCTATAAAAGCAAAATGAATTTTTATGTGACCCGATAGATAG
ATCAATATGGTAAAAGAAACTCTTATTAGTTAGACCGGATATATCCGAGTCACTCTCGCTGTCTGACC
CGCCGATGGGACTATCAAAAGAGAAAGGCAACTTTATCCCCAGGATGGGTTTAATCTAGTATCCCCTG
TACCATTGATAAACCGGGACTATCTTCACCAGATGTTGACCCAATTAATAGTGTTCGAGACCCATACC
TTCGTGATCCATAGCCCATAGAAGCACCAGTGACAGGTCCAGATCCAGTCGAAGAAGCAGCGGCCCAG
GCTGAAGAAGAAGCGATCCGGCAGGATGATGAGCAAGCTGACTTAATAAAGTTCTCCTAAGTTCAGGC
TTTCGAGAGCAGAGGGCAGGCAGTCAATGGAAGAGTACTCATGGAAAGAAAGCAGCAATCTTTCAAAG
AGTCATGGAAGAGCTCCTCAGATGGTCAGTCCCTCTTGCATTCAGTCAGTTCCACTTCCCTCTTTCGC
GAATATGGAGAATATGACCTGTTTTGTGTCCATCCCAAGATCCAACCACCTACCCAAGCACAGGAATG
TGTGGAATGAAAGGGACTTGAACTGATCTACAGTCATTCATTTATACAGGCACTGATACATTCATTCC
GGGCTCGAGTGACTGGCTTTCCTTCCTCTAAAGCCTCCATTTCCATCAAACGGTCTGGATCAGTCAAC
CTCGCTCAAGTGCTTTCCTCCATGTTGGAAACTAATCTCTTTAAGGGTACCACAACCATGCAAAAGCC
AAAATCTATCACCAATTAGTGGCTACGGCTCCTTTGTCAGTCCAACCAACACTCGCAGTTCAGCTTCT
CCGGCCGTTTTCAAGCTCCCCCCCTCGAGAACCATTGCAGTTCGGCGCTTTTCCACATCTTCAAAGGC
TCCTGCTGGGCTTGGGCACACGTCTCCATTCACTTCACTGGGTATCCATTGCTGTTTGTGACGACACT
AATCCCTCCCTAGTAAAGAGCTCCCGCGAATTCGGGTGTTTACAGAGGGGGGCAGTAGCGATCAATAA
CCAACAAGGAGGCGAGCGATAATGCGGAAAACTGTGCCTTCCATTCCAGGGTTGTTTCGTTCATCTCG
TAGTAAGTTTGAAACGGGTGTCTTTTACCCTCTCCCTTAACATGTCCTGGGCGAATAAGTGAATCCAC
CTAACCCACTGTATGAATCGTATATAACCAAGCTGTTGTAAACCTAGCACTATCGATCCACCACCTCA
CACGGCTAATAATCCGTGCCGGCCCGCCTAACCTCCACGATGTGATCCCTCCTAAACAATATTCTAAT
CCAAGCGGATGGGTCTTGAGGATTGTTTCCTGAAAGCGTAATGGGGTCTGGAAGGGTATGCTTTAAAT
GCGGATTGAATGGAGCATGGGCAGGGTAAGTTCAATAGGAAAGTTATAAGTAGTAACTGTGTTCCGTA
CCGTTAGTTTAAGGGGGAAGGTGGGCAGTGATTCGTCCCCGTCCGTTGGCCCTGCCTAACCTTACAAT
ATGTTTTTTGTTGTCCGTCCGTGGTTGGTACCCGCCTATCAATTGGTGAGGAAGTATCCTGTTTCCGG
TGGTAACGACTATTGCGTAAGATATTTCATTTTATCAGCTTGCATTATATATGCTATTATCCAACAAT
GGCAATGCTGCTGGTTATTTCTTTCTGAATCAATCGTATCTAGAAGCTCGTTCCCCCGATAAGAATCT
AAATAGTGCAACCCTTTATTTCATTCCTCAACCTCAAGCAAGTGGTTGTTAAGGAATTCATCCATTCA
CAAAGGTTGGGTGAAGCACAGTAAAGTAAGTAAAGGATGGAAAAATGTGGATAGCATGCACTTCAGTA
AAGGTATTCATAGTAAAGATTCTATTCTAAGTGCTGTACCCACTAAACCCACTAAGCATTATTGCCTG
AAGCAAGGTATGGAATGGCATTCATTAATTCAATAACAAAGGTAGGGTAAGGGGAAAAGAAGGGTCCC
TTTAAATGGTAAAAGTAAAGTAAAAATAAAGGTAGTAGGCTAAAGGGTGAATAGATTAGTGCCGTACT
TCCTTTTCCTGGCTGAAGCAAGTAGTTGGTAAGGGGAGGCAACACTAAACAAATAGGATGAGGGACGA
AGTCAAGTAAAGAGAAAAGCTGGGTGCTCGGGTGGTAAGACTAAAAGTAAAGGCAAGTTGGGTAAAGG
TAATAGCAAAGGTGTGCCCAAACCCTTTCAATCTCCGTTGGGAGTAGTTCCTTCCTCAATTAAAGAAG
GCAGTTCCAAGCTTCTATATGAGATTCATTATGAGGGACAGAAGGAAACGAAGGGCTAGAGATCATGA
TGAAAGGAAAGGGCTCATGATTAAAAGGCTTGAGAGAAGGATAGAAAAAGTGATAGTGCGCTGTATGT
GATACTATCTGTAAGCTGGCTGGCTGGGTTCAGCCCGACCCAACAAGATCATAGTGCCTACCTCCCTA
AGTCTTAATGACGTAAGTGGTTAGCAAGGGCATGCAACACTCCTTCACTAAGTGGGTTGAGACAGTTC
ACAAGGTGGTTAACACGTGGATGCACATAAGTAAAGCAGGGTAGGCTGAGGAAAGTAAATAGTCAAGG
GTCTCCTAGCAACTAAAGATTAATACAGTCCAGTTTAGATACAGGAAGACTACACATAGAGCACAGGC
TTGGGGACAACAACCATGCCATGATGGGCACTTATGTTTAGTTTTGGTTGATCAAACCATGATGCCTT
GAAGCTGGACGAGAGGCACCTACCCCGGGGAAATGGAATTTCATTTACCATAGGAATCTTATCCCTTT
TTATTTGTTCCGGGAGGCAATCGGACGAGTAACGAAGTGTTTTTATGCTTTTGAGACAACCCGAATTT
GACTCCTGACTAGACCGCCTTATTCTGGGCACTCAGTAGAACCAACAGCCCAGTGGAACCTGATACAG
CTTAGCTTCTCAAGCCCCCCACAGTTCTCGAATCAATCAGTTCTCGTCACACCCAGTGGAACCTGATG
GATGCTGCAGTTCACTGAAAGCCCCAGTTTCAATAAATGCCCACAGTGAGTGGAACCTGATGCTGCTT
AAGCTGGCACTAAATCACCTTCCCGGTACCTCCCCGCATGAATGGCTCAACCGGACCTCCCCTCTTTC
CTTTTCCTCTCAATCTAAGGCCACACCCGAGCATTCAAGAGATTAACCCCGCAGTTCCATTGCCTACA
CTCTTGCTAGCTTTGGCTTTGCCCCTACACCTGAAGGATATGAAATTGGAATTGCTCGCGCTGAGGGT
GGGTCTTGATATCGATTTGGTTAAACCCCTTTTTCTGTTTAAAACGCTGGTGGTGAATCAACGGACTA

TGTTGCAATAGATCTCTTTGCCGGGAGAGAAGAGCGACGCCGGACCCTCCATGTGGTCGGTTCAGTTC
GAGGTCATGAAAGGTAAAGTCCAGTGACTGGTTCATAAAGGGAAGATACTAATGCAATAGCCTGCATA
AGCAACTTTCTCCTTCAGGTGTAGGGGCCATCTTCTCTGCAGCTTTTGTATTTGCTCTAGGTGCTCTA
GGCTATGCTTATACTGGTGCTTCTAGCTCTGCTACTGATAGCTTAGATGCTGGAACAACTGGTACTGC
CTCCGACGCTTCTGCCCTAGCTTATGGCTTTGCCTCTGTCCCAACAAGCTATGTCTTTGCCTTTGCTA
CTGATGGTGCTACTGGATATGTTACTAGGTATGCTGCTGTAGGCTATGCTACTGATGCTTAACCCATA
ACCATATCCAGTAGTTGTATTACCAGCAGTTGAACCAATTGATTTCATTGACCGCCCAGTGGAGGTAG
TAGCAAAGTCAGTGGTTTCACCCGATCCGGAACCAATAGTTGATTCGGGACCAGCATTCTCGCCTGTT
GGAGAAGTTTGTAATCCAGTTTTAGATCCTCCAGTCCCGAATACAGATCCAAATACATATGAAGATAT
AGTTGGATACCCATCAGCAGCAGTGGGGGTAGGAGCAAACACAGCAGTGGTGGAGGAAGCTAGAGATC
TTTGTATGGGTCCAAAGAAAGCATAGGCATCATAGGCGGGGGCACTACCAGTGGTTGAAGACCAAGTT
GCCAGGGCCCGGAGCAGTTGTTGATCCCATTGATCCAGAATCATTTGACCGAGTAAAAGAGACATGGA
CCATTGGTTGCAGAGCCAATTTAGAGAGCCAGCATCAGTAGCAGTAAGAGCGTCGACGGGGTAACTAG
CAGGGGCGGGTTCACCGGTTGTTGTTGACCAAGTTGAACCAATTGAAGCGGAACCCCTTCATTTATTT
ATCAATTAAACGGGGCAAAACCAAAGGCAGGATCAGATGCAGCTACGAAGCCCCCAGTTGAACCTATT
AGCCAGTCAACCAAGTTGTTTACCTCGCCCTGATACAGAACGAACAGCAGGAACAAAGGCATAAGTGG
TAAAGCCTTACCCTCCGGTCCTCCCATTCGATCGAACAGCACGGGTGTTGCTGAAGCGACTCATGAAT
CTATAGTTGTATCCTCTGCCCACGTCACTGAAAGCCGAGCATGCCCATTCTCCTAGTGCCCGAACCCG
CTGACTGAGCTATGACTCACTGAACTATGAACATGGTAGCGATCCAACGTTGCCACGTCCCCGAACCC
CAGCATTTTGAAAGGCATCGAGTGTCCCTTTTCAATAAATAGGAGGGGTGGATGGTACTTCGAAAGGG
ATCAATGCTGGCGAGGTTGGCTTCAAGGCTTGGGATATTGGGCATAAGGCGAGGAGCCAGAGGCGAAG
CGAACAAAGGGAGCTCCTTTTATGTATAGAATTTGCCGAGCAATTTTATGTATATGATTGTGAGCGAT
ATTGGCAATAAACCAAAGAAAGGGATCTTACTGAAGGGCGAGGTGCCGCAGGTTCTTCGCATGTTCTC
ATTGGGATACAGGCCATACATAGAATGACATCATCGGGCCTCTCATCGTAATTTGGGTGGACAACCCT
AACCATTCAGTTAGAGTGGACAACCCTTCCTTTCCCATTTATCGCACCAATACTTCTATTACGTATAG
AATACTTTGATTATTCGCTACTCTCTCTGCTAATAATTTGAACTTGACCAGATAACTTGGGTGATTCG
CTACTCTCTCTGCTAATAATTTGAACTTCCACCTATTGCTATCAAGCTTTCGTTTGGAGCAAACTAAT
TCATTCGAGCAAAGTTCGTTCGAATTCATATATTTTTTTTATAAGCTTAACCCGTTCTTCAGCGCCT
CAGCTTCACCCCTATATCGGTGTGCATAAAACATCATGGTAATAGATAAGAATGGTAATAGATTCAAT
TAAGTGTTCGCCCCGAGATGATAGCATAGTTGACACCATCAAAAGCTACTGCGCGCTGGAGCTATAGC
CTTGTACATAGTGAATTCCCGCTAGAATGGAGATGTTGGTTGAGGAAATGGCATGTGGGTACGAATGG
CATATATGGGTACTTAGAAGGCTTGGTGGTGGTATCCAAATAATATATCATATACGTTATTTCTAATC
ATGGAGAGAGCCATGGGCACATGCGGACAGAACCCTCGGCGTATTAGCGACTCCCCAATACGAAAGCC
CATTATTGAATCAAAAACCAGGAAGTGCCGCTCATCGATTGATACAGCTTTTTAGGAAACTAGCTTTA
TACAACCAACTTTGTACAACCAAGCCGTGCTTTCGAATCAATTGACGGGAATTAGATAGATAAGGATC
AGAGGTTGTATCCTCTGCAAGGATTCCGACTAGATAGGGATGCTTGGGGAGACTAGCATTATACAACC
AACTTTATACAATTACCGCAAGATTCCTTTCGAAGTACTATCCAGCTCTAGCCCACACATTTTACCTG
ACATCCAAGAGACTACCAATGCTCGGTAGTTCAGGAGGTCGAACTCGAGTTCGGCACTTTGGGGAGCC
AAAAAAAAAGATGGAGTCGTTCGCCCATCTCATCTGTTGGTGGGAGCGTGAAAGCACATCCGTTGGTG
AGATACCCTAGTTCCCCAATTATCCTGCACAGGAAACTCCTAGGAGCATTTGCCAGTCCCGGGGAACA
GACATTTAATTTTCGATACTGGATTTTCGATAAGCCTCTCCCCACTACAGCATTTTGGAATTGGAATA
CCCAGGATCGTAAGTAAGGGCCGGTCCACGCAAGATAAGGCACTTTGGTTCGTACGGGTCGAATCCAC
ACGGCACGCACGGTTCGGAAGTACAGGTTTGACACTGTTCCACTATCGGGAGATCGGAGTATGGGAAA
TTCCGAATTGGGGGAACATGGGGACTAAGGGACAGGGGCTTTCTTTCGTTCGTTTCATTCGCTACAGT
CGTATTAAACATCTGTATCTTGGTGGTAATAGGGAGTGGTTCGATTCCCTTATAGGTGGCGGAACGGG
AAAACACGAGCTTGATATCAACATGGGTGACGAAACCGGCGATTGATATTCATATTTATACTCACCCA
TTTATGAATGAGAGCGGGCGGTTGTTCAATCCAGGCGGGGCTATCGATTCAGAACTAGCCACTTTCGG
GCCCTACCTATATTCGTAGGCCCCGGTCGGTACGTCGAAAGGGATCAAGGCGATTCAAGGCGAGGGGC
GGGGTGCCGCAGGACGAGCTTATTAGAAGGGGGCGAGCGATGATGCCCAACCAGTATTGAGCTAGCTA
GTGGTTGGCCGGCCCCTTTCGCTTCTGCTTGAGGAGGTAGATTTATTTATGCAGCATTATCCTGGAAT
TTTCGAGAGCGTTCCCAATCCGATAGCTAAGGGGTTGTCGCCAACCCTTGATTCCTCGCTCTAGTTAC
TACATGGAATGGAATAAATTGTAGTGGTTGAACTGGAACATTCCAAGTGACTAACTCCCCGATGACTC
CAAAACTCAAGTTTCTAGTTAACAAATCAGTTGGTTGAAGGGTCCCATCCACTCACATGAAAATCAAC
TGACCATGAGAAAGGTTACAAGTAAAACATCCAGGGATAAATACCTTAAGGGGATATTCTTATCACTC
AGGCATGGTCAATAGATCAAATCATCCATTAAAACAAGCATCATCCCCACATGCTTGTGAAGAGCCAA
TGTTTCCCCCATCCTCCAGTTGCTAAAGCAATACTAAAATGGTAAAGGATTAGGAAAGGACAATTCCA
CTAATTTTGATAACATTATAAAATGAAAAAAATATAAGATTGATGAACGAGGGTGTATCTAGGATCCT

TGCTTGGCTTCTTTCTCCATCCATCTAGTATTTTACCCCCTCGTCTATGTTCCCTTAAGAAAGTATCA
GCCTCTTTGGGGTCATAGATTCTTCCCCCAATCGACAAACAAAACATATAATTCAAAGCCACAACATC
GTTCGTAAATCTTCCAATCAAACAAAGAGAATTAGTTTTTCGTTATACCTTTGACTTATTACCTGTGA
CTTTGACTGTTATCCTAAGTCACTTAACAACGTCCTTAACGTCACTTAACAACGTTCTTAACGTCCTT
TAAGTCCCAAACATTTCATTCCACACACCTTCAGTTATGGTGGCTCCGCCCAGTACTTACCACAAACG
TGCCTATGAATGGCTCCGCCCACTCCTTACCGCGATTGAAACAGCTCTGAGGAACAACGAGGAATTAA
GCAGTTGGAGAATAGAAAGTAAAGATGCGGTCTTCCCGGCCCACCCACGCTTCTTTCGCAGAGGTAGT
CGAGGGGGGCTCTCTGTGGAGCGATAAGCGAACCCCCCGATACGGAGCTGGGTTAGGATCACACGACT
GATCGGGATCACACGACTGACCTTTTCCTTCGATTGAACTTCTTTCTTCGATTGAAGAGAGAAAGAGT
TTCCTTCCGAGAGATAAGCCGAGTTGGTTATCCCTTACCTGATACTTTTCATTGAGGGAAGAAGCTGA
CTAATTAGCTCATAAATAAACTCTCAGGTAATAGCTTAACAAGCTTTGAACACGAACTATCACGCGAA
CTATTAAGCTATTTGGTAGAGTGACATTCCTCAAACGTAACATTACGAAATGGGGAAGGACATGAACA
TAAAATGGCAGGTCATGAACATCAAGGAACGAACATCAAGTGGCATAAACTAAAAGTCACACAGTAGG
TCGTTAGCACGAATGAATGTATATGCAAGTGCTATTCCTTTCCTTACCGCGTGACATGCCTTACCACC
TGAATGGCTCACTGACTGATGAATGGCTCACCTGACCGATGGTTGAATGGTTCACCTGACTGCGTGAC
ATTCCTTGCCGTAGGCGTCTATACGTCCCTGTGCTGGGCTCACCCACGAACTCTTCAGTGGGGCACGA
ACTATTAAGTTTCCTTCCTGCCACGAATTCATTCCTGTGCTGGGCTCACCTACGAACTCTTCAGTGCG
GCTCTTCAGTGCGGCACACAAACTCTTCAGTGGGGCACGAACTACTCTTCCTTAACACAAATTATTCG
ATTGTCATACTTAGTGATGTCTCTAATCTTCTTTACGGAAAAGGATCCTTCTCTCCCTTTTTCATCTG
GTTTGCCGGAACAATTGTGCCAGACCCGAACTAACAAGCGGGCTGTCAATTGCTCAATCTGAATCACT
GGCTCTATGGGAGACCATAGACTCGGGTAAGCCTTTCGTGGGTAGTCGGCTCGGAACACCAGATAGGT
GATAGCTGCAGGTCCTTTACTCATTGCCCGAGACTTCGTGAGACTATTAAGGGCATTGAAGGTCCTCT
CTTGATGTTATGCAAGTGTGGTACTTAGCGAAGAGAATCGAGCTGCTCGGAGTTAAGTGAGGAGTAGG
ACCAAAAGGGATATCCCTTGCAAAGCCAGGGTTTATTAACCTCCGACTGGCTTTGTTTTTGATACGCC
ACCTAATGTTAACTATGCAACGTCTATATGAAATCATAAAAATAAGAAGGGACTTCTTCTTATCCTTA
AACGTACAGGTTCCCGTCTCCGCTTGTACGGTCTGGCTTGTAAGTCATAGCTTGTATAGACACTGGAA
TGACTATAGACACTAGACACTTTCCTGTGCTTGGGTTGGATAGAGAGAGTAATGGCTTCCCGATTGGA
TTAAGCCTCCCGATTGAGAGAGTAATTACTTCCCGAAGAGTAATGAATGGCTGTAGATAGGAGAGGGC
AGGGCTTTCAACTGGTAATAAACTATCCATTAAGTAGGCAAGATTGAGGGTAGGAAATAGTAGGCTTG
AGGGTAGGGCTTTAAATGAGGGTAGGCGTGCGTATAAAGGGTAGGCTTTTAAAGTGAGGGTAGGCAAT
AGCAATAGTAGGCTTGAGGTTGGAATGAATGTATACTGCTATACGCTGGCCGGTTCAAAGAGCATCCA
CATCTTTCGTCGTTTTTATAATAGGGAAGGGAAGAAGAGTTCTTCTCGATCTCGCCCTTACCTTAGAT
TAGTTAAGAGAAGACGGGGACATCACCTTGAGTTATTCTCGATCTTTCCTATCGATGGAGTAGGAGTA
GAGATTTATTCCAAGAGTTCTTCTTGATAGATTTTCCCCTCAATGCGTATGAAAGGTTATCCCTTTCC
TGGCCTTCCTCCTGCCAAAGAGATGGGACCAACCTCGATTGGGTACGCATGGGTTTGGGTCCGCGACC
AGAGAAATGACCACTTGAACGCGCTGTATCACCACGAAAATCCTTTTCAGTTACGTTAGGGGCATGGG
CTTTCATAAAGGTCATCTTTCGTCGTTTTCATTAGGCAAGTAGTAGTGTCTTACTGGTATGATTGGGG
AATACCGATACATGACCTACGGACTAATAACCTATGGCGGGCCCGTTCTGGCCATGGCGGGTAAGATG
GCGGGTAAGCATGTCTATTATATTATAGGCGTGGGTACATATAAAGCGGTGCAAGTATCAATACCCGT
AAACGGGGTGGGTATATTCTAGCCCCGCAGGGCAAAGCAAGTAGGTTCGGCTTACTCCTTCCACTCAA
TAGATTTTACAAGCTCTCCTAACTATTGGATAGGCCATTAAGCCTGGTGGGACCAATGGGATGGTTAA
GGCCCGCACTATTGGGTAGGGGTGGGACCAACCCTTACCAAGGTCTGGTTATCTTGGGTAAGGCAAAG
GTCTTGGGTAAGGCCAAGTAGGAGTAGAAGTAGGCTTAGTGGGACCGCTAAAGGGAGGGCGATAGAAG
GTAGGCGTGGTAGGAGTTATCGATTTTTTCGTGTTCTACGAAATCATTTTCATTCTATCTTTGAACAG
ACAAACACACTGGTCCAACCTAGCCTCTATCATTGAGCTACCGGTGAACACCAATCTATTCAATCTCG
ACCAATCCCGACCTGGTGAGGTGTGGTTCCTATGCTACCTATGCCCTATCTATAGTGGGCTATCGGTG
CTTACCACTCATTCTCTTTCTCAACAGATCCAACCCTCGTTTAGGTGCGGGTGCATACTTAATAGAGG
GGAAAGTCCATCTCTACAGCCCTAGCTCATGGAGTACCGGTGCTCACCAAGCATTCTATCTATCCACA
GCGGCTAGTCCCATCCCATCCGAGGTGGATCCCATCTCTACCAATCGAGACAGACTTCTTCGAGGTGG
AGCAGCTGGTCAATACCGCAATTACAGGGTGGAGCAAAAGGATAAAGATAGATTAAGATTCGTTTAAG
TGGAATTTCCCTCTCCCTTATTTAGAGCCTGCTTGCGCTTCCTTGCCCTCCTATCGTCGAGCAAGCAA
TAAATTCCTCTTTCACCCATTTTGTTAAGAAGGGGAGGACCTTATCGTGCCGCCCCTCCATTCATAGA
TTGACCCGCTCCTAGGTTTGTACTCTTAGAGGTTATGCCCACCAGAGGAGAGGCCTTTAGGTTCGACC
TTATAAGTTATATCCGCTTAAATGGTGGCTCCATTTTAAGGCATTTAATTGCCACTTTCACCCAAGGA
ATTAAGAATGACCTTACTGGGTGGATTTACGTATATTATATATCTGTTCAATATTCCCTAAGAGCTAA
AGGATAGATACTCTTTTCCTACCTTATAGATTCCGCAGAGGTGTTCTCCTCGATACGGTAGCGAGGAG
ACTATTCACTTCGAGCGGTAGTGAGAATACGATTCACTCGAGGAGCTGTCAATAACGGGTTCTGTCAG

AAGTACAAAAACGTTTCAAATGGCTATTTGATTTCTAAAAAATTCCACTTTCATCCAAGGAAGGTATC
AAGACCTTACTGGGTGGGTTTACCATAAACTTTCTGTACGTATCGGCATCAAAAGAACTAAGTATTAC
GTGAGCCAAAGGGAGAGAGGAGATGTAGCTACAAGTTTCCATTACACGCCTAATTGTACAAGGAAAGA
AATCCATTCATTGCTGCTCCATCCGAGAATCTAAGGCCAGCTGCTCCACCCGAGAATCTACGGTCGCC
ATCCGGTCTTTATCCTGCTAGTGAGCGGGCTTACTCCTGCTGCGGGCTTACTCCTGCTAGCAAGCGGT
TGTGGGCCCCACAACTTAGTGGATTGCTTATCCATTCTATTGACCAGCTGCTCCACCCATTCCTTGGT
TCTGTCTCCCTGTCTCCCAGGACGAGTTACCCAGTCTCCCAGTCACCCAGTCAAGGAAGCCAGTCACC
CATCCCGGTTGTTGCTCTCATGCCCGAACCCACTGATAAGACCTTTCCTTGGTTCGGGACCTTGGAGT
ACTTGCCTACATTGGAGTACTTGCCTACAAGGAAGGTATTCGGTCAGCTAAGGTCTTCGATAAGGGCG
AGGGTCTTCGATAAGGGTCTTCGTTAAGTGTGACGAGGAAATAGCCTTCGAGTAAGAGTGCTTGCCTA
CAATTGAATGGCTTATCGGAGTTACGAAGTTAGTGGACTTAGTTACGAAGTTAGCGGATAAACTAGTC
CAAGCTTGCGGACTATAGCGAAAATATCTGCTGCTGCAGAGAGAAAAGTACTTACCCACCTACTTACC
CACCTACTTACCCACCTTAGAGAGAAAAGTACTTACCCGTGCTCTTGGGTCACTGAATAGTACATATC
TAGGTCTTACCCCAACCCAATAAAAAAAACTATATATTTATCAGCTCTTAATAACTCTTTCCTCTCTT
TTCATTATCTTGGGTCAGAAAGAGAAAGATACTGGGGCATTGAGTCAGACACCCTATAGTAGTAAGGA
CTACTCCCTCGTGTATACAGGGTGCGTCGTACAGGTGCCCCCCTCGTGTCTATGCCCTCTCTCGCTTT
GATGCTTCGAGGGCTCGCACTCGTAGAATTGGGGGGCCCCTGTCCTCCTTCCCTTCTCACTCGTCCAA
AGGGGAGTAGTCCTTACTCCTAAGCTACTGTCCTCACTCTCCCTCAGGGCCATATGAGCTGTACTCAT
TTCTTCTCTTTAGGCACTGAACAGACTTACGGGACATGGACTTATAAGGTGCTATTTATTATATTTTT
CTATGGCTTATACCTTCTCTTTTAGCGCCTTCAAGTGGACTTAAAGTTGCCTTCCTTATGACTAGCAG
TAAATAGATTTATTATCTGTGGACCTGTAATTAGTAGGAGGGCCTTTAATGAATTAATAGGAGGGCCC
GTAAGGAAAAGGCTTTTCATTAATAGGAGGGCCTTTCCTACCTATGTATTCTAATATGGATATGAAAG
ACAAGGAAATACATCCATTCATTACAGCCGATAGGCACTCTTCGGGGTGTCATTCCTTCCACTATAAT
AGATTCATTGATATTAAATAAGTTGCCTTCCTTAGGAGGGCCTGCCTACTAATATATTCTGCCTGCCT
ACTAAGATCTGGAGACCAACGCCACATTCCGATTCAGGACCTTCGGCATACATACGTGGATTTATTAT
CTTATCTTTACGGCATACCCACATAGATATTAAATAAGTTGCCTTTTGAGTAGGAGGGCCCGTAATAT
GACTAGCTGGTCGTAACTAGGTTGCGTTTCTGTGGTAGTCGTGTCGTGGTTGCCTTCCTTATTACTAG
CAGGAAATAGATTGATTTATGACTTATAATCTGACTTACCTCGCTACCCTAAATTGTAATTCCTCGCT
ACCCTAAATATGACTTACCACCCGAGAATATAATAACTTTAGTTGTCTCCTTCTTATCCGATCTGTTG
GGCGGATCCCAGCGGTTATCGGAGGGGGCTCCGCCCCCCCGAACCCCCCAAGTTACCGGCATGAGCAT
GAATAAAATAGTTTCGGGCTAAAGGCTGTTATCGGGCTAAAGTAGGTTATCGGACTAAAGGCATGGTT
TAGAGGGCTAGCGGTTATCGGGCTAAAGGTCGGGGCTATATGGCTGATACCTTATAAAGGGGTAGTCT
TAACCGGGGTAGTCTTATGAAGACCTTTCCTTACCGGTGAAGACCTTTCTTTACTAACTGGGGAAGAC
CTTACTAACCGGTGAAGACCTTAATGCCTTAATTCCTTCCTTATGACTTTCCACCAGAGAATATAAGG
AAGAAGAAAGAGATAGATTGGGATAAGGAAAGATGGGTAGAGAAGATGGGCCATTAAAGGTAGACCAG
TAAAGCGACTTCACTAAGGTCTGGTGGGGAAAGTCCGGTGGGGAAGGACTAGTGGAAGAGATAGATGG
GTGGAAGAGAAGGTCTGGTGGGGATTGAGGGAAGAAAAGAGTATCCTGAAAGGTAAACCCTTAGCTCT
GCGTGCGAAGGGTTAACCTTACCTTCTAGTTAGCTCTTTGAGGGGAAGAAATCAAGGGCCAGGACCTT
ACCCGCCCGAGGGGTGGGGAAAAGGCATTCTATAGCTACAATAAGTTAACCCAAGGTCCAGAATTTGT
GCAACTTCAATAAGGCCAGAATTTGTGCAACTACTGCTTGCTATAGTGAATTTGCCGTAATTCACAAA
GCTAGACTATAGTGACAGAATTTGCACAAATTCTGAACCTCGGCGTGCGTGGAGCTTTCAAGATGAGG
AAAGCCCCCCCCTTTGAATAGATAGGTTTCGATCACGGGGCGGAGCAAGTCACTTTACCCCCCCCCTT
CCCCATTTGCGGTGTTTCACCTTTACCTCTCTTCGCTACACTTACATGGAATACTGTTTCGCTGCATG
CATCTCTGCCCGGTATTGGTATCTCGGAATGGAGATCTCGGCTAAGGAAAAATCTCCAGACCCCACAC
ATCATGATGATTAGAATAATAACAGTAAGAACCCACATTTGGAAAAGTTCTGTTAGGTTCTTAGTAGC
AGCCGGCGACCTCCGTTTTTTCTTCTGCTTTACATAGCTTCTCACAAGGTCTCCTTGATAGCTGGAAG
TTCTTCAGGAGTATGAAGAGCTGGAGGACTTTGTACCATCCATTCCGGTGTGGTTGGATTATGCTCAA
CAGCCCGGGGACTTGGAGCACATCTTTTGTTGTTTCCACCGCCTAAAGTGATTGTTACGACCACGAAG
AAACGACGAATCCCAACTACGGATACATAAGGGCCGAAACTGCTAAGGGCATTCCATCCAGCGTAAGC
ATCTGGATAATCTGGAATGCGACGTGGCATACCCGAAAGCCCCAAGAAATGCATGGGAAAGAAGGTCG
AATTCACCCCGAAAAGAGTGATCCGAAGATGGATTTGACCTAAAGTTTCAGGGTATGTTCGACCAGGG
ATTTTACCCACCCGGAAGTGAGATCCTGCAGATGAAGCAAAAACGGCTCCCATAGGAAGTACATAATG
GGAATGTGCAACCACATAATGAGTATCATGCGGAGCAATGTCTAGCCCAGGATTTGCCAGGACTATTC
CAGTGAGTCCTCCTATGGTGGACGGAAAGATGGACCCTGCAGCAGATAACATGGGTGTTTTGTATCGT
ATCGAACCTCCCCACATGGTAGCGATCCGACTAGAGATTTTGATTCCAGTGGGGACAGCTATGATCAT
GGTAGCTGCGGTAGAGTGAGCACGCGTATCAACGTCTGAGCCCACAGTAGACATATGATGAGCCCGAA
CAGGAGATCCAGGAACACCAATACTGATCATGGCATAAACCATGCCTGGATACCCGAATACCGGTTTT

CCCGGAAAAGTCGATACGATATGACTAATGATACCGAATCCGGGCGGAATGGGAATATACACCTCTGG
ATGACCGGAGAACCGAAGGAGATGCTGGTATAATATCGGGTCTCCCCCTCCAGCAGGATCGGAAAAGG
TTGTATTAAAGCTTCGATCGGTTGATAACATGGTAATTGCCCCTGCCGGTACCGGAAGTGATGATAAG
AGTAGGAATGCTGTCACTGGAACGGACCGCACAAATAGGGGTGATCTATGCATAGTCATTCCAGGCCC
GCGCATGTTGGGGATAGTAGTTATGAGATTGATAGAACCTGAAATGGATGAAACACCTGATAGATGAG
GACTAGAAATCGCTGGATCAGCAGCTCCTCCGGAATGACTGGTAATACCACTTAGGGGCGGATAGACC
GTCCACCCAGTGCCGCTACCCACTTCTACCGAGGCTGGGCTTAATGGGAGCAACAGCGAAGGTGGCAA
CAACCGGGATGGAATATTATTCAATCGTGGAAATGCCATGTCAGGTGCACCTATAGGAATCGGAACGG
ACCAATTACCAGATCCACCTATCACCGCCGGCATAACCATAAAAGGGATCATTGGAGAAGCGTGAGCC
GTTATTGACACATTATGAGGTTGATGATTCCCACCAAGAATTTGATCGCCGGGTTGTGCTAATTCCAT
ACGAATTGGTACTGAGAAGCATGTGCCCATCACTCCAGCAATGGCACCGAAGATTGAATGTGGAGTCC
CTATATCCTTGTGGTTAGTGGGGAACGGCCATCGAACCAAATTTTTCGTAGAATTCTGATTATTTCGT
TTCATTCCTTATCAGAGAGGGGCCGGCCCCGAGGAGCGGGGCGGCGGCTTATTGGGCGCGCCCCCCCC
CACCAGTCAGTGTCTCTTTCTCTTAAGCGGGTGAGGGTGGTTCTGAGAGGGGGAGATAAACTCCGGAA
ATAATAACCCTCACCCGGGCTAACAGCCAGCATTTTTCCAGATCCTTCAATCCAACCAACCAATCCGG
GCGGGTAAGGTCCTTCAAGGTCAACCCAGTCAGGTCCTTATAAACCCTGCTATTTTTAACCCGCGAAG
TCAACCCTCCATTGGACGGACCTTATAATGCCTCACTGTACCGAATACTTATTTCCTTGATCTCCTTC
TGCCTACCGGGGGAGTGGCTCCGCCCCTCGATCTCCCGTCCGACGGGAGATCGAGTGAGAAATCCCTC
ACCCTACTATATGGGGGTCGGGTGAGCGATTTCATCGATGGCGGCCCTTTCTAAGGGCGAGGTACGTT
AAGTGACTCGCCCGACCTAAGAAGGGGGAGGTACGTTAAGTGACTCGCCCGACCTAACTGACCCTCCC
TCTCTTTGAACCTTGTAAGTATTTAACTCACAGATAGAAACTTAGTGCCGTTTGATGAGTAACTAAGA
ACGAGGGAGAGGGATACGGGAAGGATGAAGTAATGGAAGAGAAAGTAATTGCTAGTAGTAACGACTTG
TCACGATCCATTGGTTTATATATCATCCATGTTCTAGGTGTATCGGGATACATTATTTTCGCTAAGCG
TATATAATAAAATTGGGTGGAAGGGTCCACCCCGCGACGAAGGATGGGGTACTAACTGCCCCAAGACC
AAGGGGGTACTAACCATTATTTTAGCCCCTGTCCTCTCCGAACCGCAGGAGATAGTTGCCCATCATAC
GGCTCACCAACTTCACTTGCTCCGAGAATGTTAGGTCGGGGGGGCTCGGGCGGGTTCACAAACGAAGG
TCCTCCTGAGCTAGGCTAGTAAGGTCCTGATCTTACGCATAGCGAAGCTAACTCAGGCTTTGAGGGGA
AGAAATATAAGTGAAGTATGGACTCTATTTATTCCCCTCAAAGAGCATAGCGTAAGTAGCGGAGCCCA
TGGTTGGTTTGTTTGGTGAAGAGGCAAGGGGAAGGGATGGGAAGGAAGGGGCGGGATAAGCCGAATTT
TGGCAAATCGATCTATTTGTGTAGGAAAAGCTTATTTTGCCGTCTCCCTTTTGTCCCCTTTGAGGCTT
TTTAGGTTATCCCAGAATGCACTATAGATAGATCGTAGTTGCTGAGCTATAAGGTCATCACTTACCTT
CCTTCTAGCGGGATAACCTTCCCCTCGCTACCGGTCTTACCTGAGCATAGCGAAGGCCTTGAGCTCTT
TCTTAAGTGCTTCCCTCCACACCTTTACTTACCTGCCTGGAAGGGCCTTACCTGAGCGAGTTAGTTCG
GGAAGATAAAACCCTTTCTTTTGACCCGGTCCTCTCAGGCGGTCGGTAAGGTCTTACCGGGTGGGTTG
GTCCTTCCTGAAAGCCCTTGATTTCTTCCCCTCAAAGAGCGCTTATAAAGTGACTCTTTCTTCAAAAG
TCCATTTCTTCCAGTCATTCTTTATATATGTAATAACACTAGATTCGTGATTTTATGCGATCCGAAAG
CGTGAAACTAATGCCCTTTCGCTTCAAATGCAGTTCTTCCTCACCTTCATACTTCAGGTGAGCCAATT
CTTCCTAAAGTTTCTACTTCAGCAATTATTCCAGTCATTCTTTATATATGTAATAACACTCGATTCGA
TTCGATGTGAGTCGAAGGTACGGAGTCGCTCGATTGACATGGGAGATCGAGGGGTGAGGGTACGAACC
GCTCGCCCGACAGCAGGAGGGAAAGGAATGTGAAGTGAATCACCCTAACTTGACGTTCGTGAAACACT
TCGTTTCAAGTCATAAGGCAAGCGTAATTACATCTATAATGAGATATATCTTTGTATACGATATTATT
CGATCATGTTTTCGTTATGATTCGGGTTCAGAAGACTCGCAAAGCAAAGGAAGTAAGTGCCTTACCTG
TTAGGGGCAGTAGTAAACAGACAGTAATAAAGGCAGTGCGGTAAGTAAGATCGGGTAGTTAACAGTAG
GCGTGGCGGGTTGGCGAATAGAATATGAATATCTGGATGGAATAATCGCCTTACGATACACCAAATAC
GAAATAATTACAAGAAAGATAAGGAGAAGAGTTTCCCACCTTCAGACAGATAAGGAGAAGTAACCCAC
CTTAACCTTTAGATAAGTAAAAGTACCCCACCTTAACCTTAACGAAATAATCACCAAATAACTCACCT
ACCTTGCAAGGGTTTTTACTTTTCATTCATTTAGGTAGGGGCTTGCCATCAAAGAAATATAGGCATCT
TAATTGGACTTTTCATAGGCGTTAGAAGATGCGAAACCTTGAAACTCAGGTATAACCGGATACTTTGC
CATGCCGTATGTCATCCTATTTTCAGGTGCCCTCAATGCTATGCCTGCTATCTTTCTTTTTTTCCTTG
ACTGGTCCGAAGCTCGCAGGACCTTGAGCCAATAAACGGATAGATGGTCGATTATTTGTAGTTACCCT
TGCTCCCCCGATTGCTGGATGGCACGGATTAGAATTACCCCATACTGCTGCAGTTGGACAGGTTGGAT
CGTCATGCCTTAGTAATTAATCCCAGAACCTTGAGCAGAGATTGTTTCACTGCTGGATAGATTCTCAA
CTGCCAGAATTTCCGAGACATGACTTCAGAAACATAGCTGGATGGTCTCGAATAGCCGAGGACTTGTA
GTAACATGCATGCTCCTGCGGGAGGATGGTCAGCCAATAAGGATTGACTTTTAGCTTATATCGTTGAA
CTGAATCTTCCGGATAGCTCAATTATGGGCCATCAGAAAGCCTCGTTGGGATCGCATGAGGATTCACC
CAACCCTAGAAGTTTTTAACACCTCAGGTTCAGGGACATGCTATGAAACACTTCCTTTGCCTTTCTTT
CACTTTTCAGAAATACACCTGGTGGAGCCAGCAGATACCTGGAATCACGTTGTCAGTAAGTCAACCAT

CTAGAGACCTTTGAAGCCTGGGCTGGGTGGCATCCTGCAATCGTTGGATCGGAATCGGAATTTAGCGC
CCTTACCTTTGAATACTAGCCACCTGCCAGAAAAAGATTCTATGTACAAACCTAGCTACATGCTTACA
AGGGAGGGAATGCTCAGACCAGGAGAAGTGCCGGATCAGATCGGATGATGTGGGAGGGGAATCACCTT
ACATGCTATCAGCAATTGACTTGTCTACATAGAAATGCACGTTTTTATATCGCGGTGGAACCATTAAA
TGACAGGTTGAATATAGCTAGAAACGCTTCTCAGAAGCTTTTTGATATCGACCGGACGGAACAACAAA
AGGGATTAACATTGCTTTTATATCGTGTATATCGTGCTAGAGAGCGCTGGAAATCAGTGACTTGCTTA
ATGAAACATTACATTTTTAGAACCGGATGAGACCCATCGGCATTACTTTTATATCTTGCTTCAGGGAA
AGGGACGGAGTGAAATGCTTCCTAGATTTTATATACGTAATTACGATGCGGCAGAAAAGCGAGTGGGC
ACCTTGCTTGGTTTTACCCGTACTACTTCGGGCGGGCAACCACACTAGGAACTCATACTTCTTAGGTC
GGTCGGGCCAGTTAGTTAATTAATTACCTTCTACTGCGGCTCCGCTAGCCAGTTACCTAAAGAGAAAT
TTGAGTTCATGTGAGTTCTCGACCTCGATGGGGAATATGCTTTGGACCCCTCTCAACACGAAGCGCGT
AGCGACGGAATGGATGGGATGAAGGGCATGGAATCACCCAGAGTAACCCCTTCAGTTTTGAATTGGCA
GCAGCACATGCTAGAATAAACTACCTTGCCCCTTTCTTTCACTTGCCAGAGCTAGACCTACCTGGAGC
CATAGAACTGAAAGAATCACCTAGCTAATGTGAGTCAGGAATCTTGAGCCTTAGAAGCAAGCCCCGGG
TTTATGGGAGGACGGATGGAGGAGGAACTTACCAACAACCCAGCCAGGAATGAGACCAGCAAGATCTG
TAGGGGACACTCTTTTCATACCCCTTACCTTGTTCATACAATACACCTGCTATACATACATTCTATGT
AACAGCCCTATATCATGAAGCCTTTAGAGGGAATGCTATAAGACCAGTTTAAGTGCCGGATCGGATTG
GATCATTGGATGCAAATGGGTGAGAATTGGAATTGGAATCATCCACATGGCCATGGGAATTGGCTTGG
GGATGCCTCTTTAGACCAGAATCCTCAGAGCAGACAGTATACACTATAGAAGCCTTTAATACTTCGAC
AGGAAGCAGGGGCATCTAGTATACACTACCTTTTTACTATTTACCTGCACCCACTTGTAGACCTGGAG
CTAGAGACACCTGGAATGCACGTTTTCAGTTTCAGTGAGTCGATCATCATTAAAAGCACTTTGAAAGC
AAGCCCCCGGAAGATGCCATTTATGCTTAAGAACCTTACCCTTAGACCTTTGAATGCCGAACGAAATA
GGCTGGCTAGCACACGATAGAAGAACTTTATTGAAACCTGGAAGGGATTAGCTGTAGGCAACTGTTTC
AGAAGGCCCCTTACCTTGTTCATACTATACACCTCCCAGACAGATATTCTATGTACAGCCCTAGCTAA
TGAGGGCTTTGGAGGAATCTACAAGACCTTAGGAGCCACAGACAGATTGGATCGTTGGATCTTGGATG
GGAAGGGAATTTGAATCGGTTCATTAAACCTTTGAAGCCCCAGGTTTATGCCATGGAGCCACTTACCC
AGGAATTTGAGAGACCCAGAATAGATGTTGGAGAGGGACAGTCTCTTATCAGAAGGCCTTACCTTGTG
GAAGGAATACTACCACCTGCCTTAAAGACTTTCTACAGCCCTAGCTACATGCTTATAACGGAATGACA
AAACCAAGCAGGATCAGTACCGGCCGGATCTTATTTTATTTTATGCCAGAGGGGAGGGGAGAGAAGGG
AGGATGGACGGAACGACGCGGATTAAATGCTATCAGTCATATACTTCCCTAAAGAGAATCGCTTTTTA
GAACACCCGATAACTGATTACTTTACCCAGTTAAACAACGGGTTTCTTTATATGAAACATCCCAGATG
ATAAGCTTTTTCTGATTCACCGGAACCGACCTTGTAGCCCCCAATATTGCAGGCACGCTCTGAACAGG
CCCGAAAGCTGGACGGAACAAACTATCTTTTAATAAGAATTACTCTTATTACATATATAATAATATCA
TATCACACTTTTAACAACACCAGGGCGGGATTTGACTGTTACCAACCTTTAAGTCCACCCAGAATAGC
TTTACCATATGGATATATTACATTCCATCCACCCTTCTTACTCTCCTATCAAGACCGGACAACCTAGG
ACAATTTTGTCATGGAGGATGCCTAATCCAGGACCTTCATCTTAGAAAAAGCAGAGCAGCACCGGCAT
TAGGGCATTGAGTCTAGAGAAGCCAGGAACAGTCCAGTAGCTGGAAGGTATCGCTTACATACCCTATC
ACTCTTTCACCTGCTACACAGACTTTCTTTCTATGGAGTGGAGCAGTCCTAGCTACATGCTGGAAACA
GGAATCTACCAGACCAGGACCAGTGCCGGATACAATTTGGATGGATGGGCGATAGGAGATCGGGAATC
GGAATTTGGAATTGCTTCATTAGACAAGCCACCTCCCAGACAGAGAGAGATTGTGGGAGCCCCATATC
ATTAGGTCTTTGTAGGCACTACTAGAAGAATCTTTGGAGCAACAACTGATTGGATCTTGCTTCGTGGG
ACGGGATTTGCAATTGGTTCATCAGACCTTAGATGCCCTGCTTGCTTTATTGGGTTGCTCTTCTTGCC
TTACCCTTATCGATTCTTACTGCTGGAAGAATAGGCTACGACAATTCAGTGAGTCACCTCAACATCAG
ATCACTAGGGGACTCTTTACACCTTACCTTGGTCATACTTGTTGAAGCTACATTTAGGGGCCTTGGAG
GGAGGCACTGCTAGAAGAGCTTCATAACAACAGATCAGATTGGGATGGATTTAGAAATTCGCGTTATT
ACATATATAATACACGAACCAACCGACTCATTAATTAGATAGACCAGAGAGACCAGGAAGCGAAGATT
CATTGCTGTAGGGAACAGTCTCACTATAACTTTACTTTGAATACTAACACCTGCTAGACAGACCTTTA
TCTGGAACTGTACTACAACATGCTGCAAAGGGAATGCTTTCAGAACAGGACCGGATCATAGGCAATAG
GTAGGGCATCGGGTCATTAGACCGAACAAGCCCCAGGTTGATTCGATGGAGGAACTTACTCTTTAGAC
CAGGAAGAGATGTAGCGGTAGCGAACCTATCACAATCACTTTCCTTGTTATGACAATATACCTCCTAG
ACAGACTTTATCTCAGCCCTAGAACGAGAGGTTAGCTGGACCCTTCCTTCCTAGAAGTAGTGGGTGGA
AGGATATACTCAGAGGAGCACCAGATTCGCTTGGTTCATCAGCCCTAGAAATACTTTAGAATCGTAAT
GCCTTTTACCTGCTGCATGCACCCCTTTCATTCAGTTATTACTACCCTACAACTTACAGGCTCTTTAC
CCACTACAGTTGCTTACAGAACCAGACCCCTTACCTTGAAACCATACACCTACTAGACAGAAGAGAAA
GAAGAAGGGAAGACTTTCTCTGCCCTAAAACGAGAGAGAGGTACTTTATACCGGTTACAGGGCACACT
CCTAGAAGACACAGCAGCACCAGATTAATCGGGTGGGTGGGTTGTAAACAAGGGAAGGTAAGGTCTTA
TAGCTGTCCCTCCAAAGACTTAATGTTCTAGGGCTGAGATAAAGGTCTTTCTTTGTGGCAGGTGGTGG

CTTGTCTGAACAAGATCTAATGAACCGATTAAACATCCATCCCACGATCGATCCCATCAGCAGCTTTT
TTAGGTCTACCGGTGACTACCAACACGAAAATGCTCTGATTCAAGGTCTAGGGCTGAAGATATCCTAT
CCTAAGGTGGGTGAAAGAGTAAACCAAGAGTAAGGCCATCCCTTCCAGCTACTGTTTGTTGTGTTCCT
GGCTTACTTACTCAATCCCAATCCCAACCCATTCCATAATGTGGTGCTGCTTCTAAGGTATTCTATTC
GTGGTTCTACCCGGTAACTTGAAGGTAACTGGACCTCATGGTGGCAGCTACAGAGAAAGTCTTTCTGG
CAGGTATCTGGATTGTATGAACAAGTAAAGGGTATTCTGAGACAGCTGTTCCCTACTACTACAGATTG
TCCTGGTCTAATATCTGGGTGGGTAAAGTAAACATAATCCCACCGCTGTGTCTAGGGTTCTGGGCAGT
AAAACCTAAGGGTAAGGCTGATTCATCCATATCACCATCAAATAAACCCGGGGCTATTAAGGTCTGAT
ATGGGTCTAACTGGTAACCTAATTCTATAGGGGTGCTCCATGCGTGGTAAGCCAAGCCCCTTATAAAA
GTCGAGTAGACGTAGCCGTGTGCCCAGTCCCTTGTCCAGCCCCTGATCCAAACCAAGTATCCCAGCCC
GATCAACCCAGGTAGGATCATTCAAATGTTTTCCGTAGCATTAAGTGAGGTCGAGGTATAGGTATATT
AATCTTTTTATTCGTCGGGCTATTTCTTTTGTAAAGCGCGAAGAGTCAACAAGCACAAAGGCCCTCAA
CCTCCAATTGAATCCATCCTGCCTGTCATATAAAACCTTCCCGAAAGGCCTAGTGGAGTAAGAAGAAA
AACCACTTTCATGTGCCGATGATATGTCTACCTCACAGAGGCATATTGCCCATTAATTTCCGCTCATC
GCTGGGCTTGGATCATTGGAATCATTGCCTTGGATCATCGGCTCGGCTGCTCAAGCTTGGGATTTTGC
CCTTGCCTCATCGGCTGGGCTGGGATACTTGACTCGGAGCAGGTACTAAGTTCGCTTGGAACTAATTT
AACCGCTGCGCCCCTCATTCACCATTCTTGGCTAAATAAATTGCTCTTTTTAGGAAGCCGAGTGATTT
CCTCATCAGAGCAATGAAACGGTATTTTCTCCTCACTGAACTATACCCTTTCAGATCGTTTACCTATG
ATTCCCTGGTGGTGATTCGATGACAGGAGGTAATTCACTCGATGCTGGTAGGAGATCCAAGAGGCGAT
ACAAGTGTTGATGCATTCAATGTAAGGTAGGTGCCCATGTATTCTTGTCTGGGAACCGATGGAAGACG
TCTTCTTGGAACCGATGCGCGATCTTACAATGGCACCTCTTTTGAGAAGCTAAGCCCATTTTTGTATA
ATTACATATATAATGCACGCTTAAAAACTGACCTGCCAACAAAGGCGTTACCCAGATACCCAGTCCGC
ATTACACATTCAATCCGCATTACCGCATTCAGCCCAATCCCCACCAGCAGTTGCAATACCCCGTGCCC
TGGAATGAGGTGGCGGTACATTTCAATAATAAGTTACAACAGCTTGTTATATACGGAAATATACCCGA
TATAGCGTAGAAAAGACAATAATCAGGTTGCGCTGCCGGTACTCATGCTGGTACTCGATCTGGTACTA
AATGCCGGTACTCAATTCCAAGAGTCGAGCCAAGAGGCGATCACTCGATGACAGGTGGTGATCCCAGG
AGGAAATGCCAGTCATAGATGGGCTGGGATACTCGGCTGCTTGGCTTTTGGCTGCTTGGCTAATCGAC
TTGGCTTGGATACCGGGATCGGTTTCTCAGTTGGGATATTCAGTTGGAAACATGAGCTGCTTGGCTGG
GATTACTTAGATGGATGGAATGACTTAGTTAGCAACTGGTCAATTTGAAAGTGAAAGGGTGGCCTATC
CAAGTGGTAAATTCAGGAACAAAAGGTGGCGTGTCAAGATGATATAGAGAAGAGAATGCTCACACGTC
CTTGCCCGATGAAGGACAAGACCTACCGGACCTATCAATACGCCGCCTGCTTGAGCCTATAAATGCCC
TATTTACAACAAACCCTTCCGCCCAGAACAGTCCGCGTAAGGCACAGTCCGCCCAGTAAGGCACCCCC
CCCTGTAAGTCCAACTGGTCCCATTCCTACGGCGCTAGATCTGAGATACACTAGGCGATAGAGCCTTT
CATAGTCGATCTTGATCATAGCCAGAAGGGCCCAATAGCCCAAACCCCAGTAGTTGTAACCAACCAGT
CATAGCCAGTCTCATTACCAGAAGGTGCCAATAAACTTGTTGGAAAGACTTGTCGGGAGGACCTCATT
CCCACACGGTCACTGTCAGTCAGACGAGAGGAGATCCCCACCCCAGGAGGTGCCGATTCCCGATAGTT
GATGCTGTCGATCCACTCGATGACAGGTTGCAATGCCAGAGCTGCGCGGGCTTTTATACTTTACTCGG
TTGGGCTTGGTTACTCAGATTGGCTTGGCCCCTCAACTGGGCTCCTGAGATTGGGGGCTCGGCTACTC
GCCTGGACTACCCGGATCGGAGCAGGAAATAAGTATTGTTGGAAATCAATTCTAAAAAACTCTTAAGT
TACTCATTGGGTGGGGGGGCGACTACTAATGGCAAGTCCCTATGGGTCCTCTTTTTGAGGGATCCGAG
CATGCATGCATAGCTCATCGGGGCGCTGAGATGGATTTTCTTCTTTATGAAACATGCCCTTTCAGAAC
ATTTCTATAGAGATTGGCGGGATGGGCATCATTTGCTTTTAGCTTGAGCGTCTTTATCCTTGCTCGCT
TTACAAATTCACCCATCGAACGATATCACACGATATCAATTATGTTACCCCGTGTTCCCATCCCACCT
ACCTGCTTGAGATATTGCTGCTTGCCCATTAGTACCGCTAACGCTCGATACCCGAACTCTATGGACGG
AACACCGTTCCTACAATCCAATGAGGGAAATATGACATGCCAATACTACAATAGAGACACTGAACCAC
TTTCTTTTCACCCGCCCAGGAAGGGCACTCTCTCTCTCCAACCTGCCCACCTACTTTCAAGCCCGTGT
ATAGTCGCTTATTCTGGCTTGGGTCGCTAGATGCCAGCGGTCTTGGGTTGATAGATGCCAGCTACCTA
GCCTGTGGATGGATATATTTGGTTATTAGTTCTAGCATTGGGCCTAGAGAAAGCAAGTAAAGCAGGGA
ATTAGCAGTTCCGCCTGTAGAAAGCTGTTCAGCAGTTCTGTTCCGTTCTAAACCGTTCATAACCCAGC
AGTTCATCCATTCATAACCCTGAGCCCTCACCCTATCGAATCAATTCTATGGGTACTTAAAGAAACAA
GGGACCTAAGGAAGAAAAGAAGGAGGCTCCATCGAATAAACCATGAGGCTCTCAAGGGCGAGGCCCAA
GCCGGACCCACTACACTTGCTATTCAGGGCTATACTCACTATACATTTCAGGGCTACACTGAGGACGT
ATGAGGTTACACTTACTATTCCTGCCTCTACCGGGTATCTAGCCTCTACCTAGCCTCTACGGGGTAGC
TATCGAACTGGTTATAACGTTGTCTAAACCGGGCTATAGAAGCCTCTGCCGAATAGCTATCGAAGCTG
GGTAGCTATAGAGTTAGTTGCCCTGCAGCTCGTTAGTTGCCGCAGCTAGTGACGCTAGTCGCCCCTAA
AGAAGCTATTGACGCTATAGAATCCCTTCCGTTCACCCTCCATTCACCCTCCTAAATAAGCTCAATAA
TAATTGACGTAGCAATTGACGTTCTAAAGCTATTGACATATCAATACGAACGATTTACAACCCTCATT

TCAAGCCCTAACCTCATTATACCAGGGTGGGGTAGCGACCATAACCTGCCTATCACTACTCCATACAT
AAGCACGTTTGAGGGAAGGAATGGCACTCGACCAATTGCCATAAAGACCGGTAGTAATGGCCCAATTT
ACCATACCAATTTCATCTCTATTTATCTCTCCAACGGTAAGCCGTCGGTACGTACTTCGGGTTTTCAT
CCATAATCCCAGGAAGGGGCCTTTCCATGCACGCACGTATCCACCCACATAAGCTAAAAGCGAGTTAT
TTACCTCCTGATGGAGCCATACCTGTTGGAACCCCCAAACAACACACCGTGCCTTGTCACCGAACCTT
CAGTAGAATATGCGACGGGATAGACTATTTTATTGATAACCCCTTCTTCTTGCTAGGATTTTCACCCT
CAAACCTGCCTGGTAGGTGCCCCAATACTAGGACCCCCGGTGCCACAAGGAAGGATGGTTCAGACCTT
CTTACACTAGGTATCACACTATAAAACAACCCCTGATTGGAGGGACAGGTTTAGTCTACAGGAACACA
GGAAGTGGTAGGGAGAACAGCTACCACGTGGGAACTGCTTGATGAATGCGCAGCATTTAAGGTGATTC
CTTCCTCTAAGGCTAGTGGGGCAGCATAAAGAGGCTGGAGCGGGGTAGCCACCTTTTCATTATAGGCA
ACGCCACCCTTCAGTATAGAATTTATCTCTTTGCACTAGGGTGAGGGAGTGTGAGAGCGACTTAGGGA
GGTAAAGCTAGCTATAGGTCGTTGGGCAGGAGGCTTAGGCGGGTGAGGAGCTTCAGCTATTAAAAGTC
CATTGGGCACCCGAGAAGCTAGTTTCGTATTTGTAGATTGGTTTAATCGATCAAAAGCGCTCCCACCT
AATGCACGTTTTCTAAATCAAAGTGGTGTTGTTGGGGATTTGAGTTCTGTAACCCAGAATGGAGGAGG
AATATCATGCCCAGGACCCCCGACAAACAAGGAAGGTCTTCCTTATAAAAAACGCATATCAGAAGGTT
TTTGGAATTGGGACCAATCGGCATGGCTTTTAGATCTCTTGCTTCAGGGCATGGCTCGTTGTCTATTG
CTTTAAATAAATTTCAATTGGTACATTGAAGCCAGATAGACAACCTTTCATTGGTGCCAATAAGAGAC
AACAGCTTTTCAACAGCTTTTAATTAGTGTCAATTAGTGTCAATAAGAGACAATAGAGAGGCAAGCTA
GAACAGAGGCAACTAACTGCAATTCAAGCTAGAACAACCAACTGCAATTTCTATTTTCATTAGTGTCA
GTAAGCCAGAGAGCCTGCCAGCCAGTCTTTCAACTCTGCAATTCAATCTAGAACAGATAGGCAAGCTA
GAAACAGTAAGCTAGTCAGTCTTGCCAGTAAGTCTTGCCAGAGAGGCAACCAACTACAATTTATTAGT
GTAAATTAGTGCCAATAAGCCTTTCAACTAACTGCAATTTATAAGTGTAAATAAGAAGAACAGAGAGG
CAAGCTAGAACAGAGAGCCTGCCAGTTAGCCTGCCAATAAGAGACAACAGAGAGGCAAGCTAGAACAG
ACTGCCAATAAGCTATTCAATTTCATTACATTTGTAAAGCCTTTTAGCAGCAGTTTCAGCACTACTTT
AACCATCCTCATTCCCAGCAAGCAGAGTAGCACCAGCAGCACTAGTAGAACCAGCATTATGATTATCA
GTTGTTCCAGGATCATTTCCATCAGCAGTTGAGCACGGTCATTTATAAGAAACGGAATAATTCCCAGC
AGAACAAGGATTCCCAGCCCAAGCAGCAAAAGGAATCCCAGGATCATTTGAACCAGCATTCAAAGCAG
CATTAGAACCAGCAGCCCTAGCTGCAATTGGACCGGCATCATGACAAGGAGCAGTTGAACCAGCAGAA
TCAGAAGTAGTTCCAACACCAACTAGTTGAATAGGACCAAGAGTAGCAGTCCCAGGATCATTCTTCCA
AGCAGCATAACCAGTAGAAGCATCACCAATTGAACCGACAGCAGCCCAAGCAGAACGACCAGCACTAT
GACCAGCATTTAAAGCAGCAGCCGAACCAGGAGCACTAGGATTCCCAGCAAAAAAAGCATAATGATCA
GGAAGGACCACTAAAACCAGTGAACCAATAAAGGGCAGGCCAAGCATTATTGAAATCAGCATTATCAG
GAGCAGCATTACCATCAGCATTCTTCCCAGCATTCTTCCCAGCATCAGTGGAACCGGCAAAGGCAGCA
CAAGCTGAACAACCAGCACAAGTATGATTCTCAGTACGAGCAGCACAAGGAGTCCCAGCATATTAACC
AGTTACCGTAGCAGTTTAAGCCTAAAAACCAGGAGCACTACAATCCACTAAGCTTGCTGCTTTTCAGA
GATCGATCCGCTCTTTCCCAAAGCAAGGACTATGCCATGAAACCCATTGTCGAGTCATTATCTTCATA
CCTATTGTATGTATGGCTTTCTTCCATCGCTGTTCAATCTCTTTTATAGAGCAAAAACTCTTTCCACT
TCACTTGTGTTACGGAGAATGCAATGCCGCGCTTAACATCATGTTCTGATGAAGCCGGGTTGACTTGA
GAAACGGGGCTCCTTTTGTTCAACTCCGTGCACATCAAACGTTATATGTGCTTGGGAAGCGGAAGGAC
AACCATAGTGCAGCGTAGCGGAAGGGGCTTTAGGTGAGCTCGTAACAGCAAACAGACATGAACTTGCC
TATGTTGCTCCAGTTCTTCGCTGTTCTAAAGTTATTCATTTAGGGTGTAGGGCACTTAGCTTTACGTC
ATTTATCTCAGTGTGCTTTTAGAGACTTTAGTTATACGGCTCAGCCTAAGAAGATTAATTAGGGAGGC
GATGAATGTGGGTTTACAAGCACATTGTTCGTTCTGTCGAACTCGGCTTTTTGAAAAGCTTCACCCCG
AGTTCACTTTACACCTTACGCCTTACACGTTTGGATTTGTTTCATTATGGTTAGTTCCAAAAGAACTG
GTAAGATTCAGATTCTACGATTGACTCATATATAATATAGTGAGTGGCTTGCTTGCATAACTACTCTT
TCTACTATGCAGCAGCCCACCTAACTAATTTCTGGCATGTGAGAAGATATAAGATGATTCTAGGTCGT
TTGGAAGATGCAGTTCGAAAAACTATACACAGAGCAACCAAGCGTAGATCAACTCCTCAGAACTAAGT
GAACTATATTTCTTCCCCTAAAATCGCTAGCTAGAAGGTAAGGTTATCCCTATATTTATTCCCCTCAA
GAGTGAGGACTTGTTTCCGATGTCACAGGCTATGAACCTGTCCGCACCAAAGAGTGGAACGCCATCAA
TTGATCACGTTCCTTAATCTCTTCTCTATTTCTACACTATTCTTCTTGGCCTTGGCTTTGGCTGTACC
TACTTTTTTTCGCTTGGCCTTATTGCTTGCGTGCGCGAACTAAGGCAATAGTAGGTGAGTTAGCGTGT
TACGATAGAATCCTGGTAGATCCAGTCAAGTCCTGATTCAATACGTTAAAGCAATACTTCGTCAATTA
AGAGCCATTGTTTTTATGGAGTTATGTACTTACTAACTTTACTTATTTCCTTGGCTTGGCCTTGGCCT
TATTCTATCTCTAGTTACCGAAAGCATACGAGTCATTAAAGCCATACGGGTCGCAAAATAGCAGAGCT
ACATAAATACTTTCCAGGTAGGTGTACACTTCGTTACATATTGCAGGTCCGCCTTAAGCATCCTCTCT
GCCGTGGTCCAGCAACCAACCCTATTTTGGTTTTGAAGGTGTTCGCTATACTTTCCGTCCCTCTCAAG
TTGTTTCTGAACAGTGAAAGGAATCCCCCAGCTATTCTCTCTCCCATATGTATGTTCCCGGTCCGCTT

TCCCTCTTACCGACCGGTATGATGAGAAAGGGCGCTTGACCGATTTCTAAGGGTACGGAATCGGGTGA
TCAACTTTTCTTTCTTCCCCTCAAACTATCGTTCTGTCTGACTGAACCAAACCATAGAAGGCCGCACT
CGACACACAACGGAGGGAGAGGGGATGGGGGAGATAGGACAGGCAATCCCTAGTTCCGGGACAGCCAG
CACCGCACGGAAACTCACACGAGCGAGAGTGCCAGCCCACCGGTTCTGAAAGGACCTTATCTCATGAA
CTTTTCGAATACTTCGTGGAAGGTCAGCTCTAGAGGGAGGGCAGACGGCCCTGAAAGGTCCAGCCAGT
AAGGCCCTAACCCGGCCGGCTTCGACTTGTCCTGATGGACCCGTCTTACTTAATCATTTGTTTTATTC
TGGTTTTGCGATGTAAATCCAGTCAAGTCTCGATCCTTCTAATGCGTGCGATTGTTTCTACGGATGGT
GTGGTGTTTTTTCCTTATCCTTATCCTTATCCTGATTCTTTTCCTGGCCCTGGCCCTGATATCCGAAA
AGGAAGGAATGAAGTCCGTACTATGCCATACTTCCCTCGTGAAGTAAGGGGCAACATACATAAGGTTC
TGATCTCTTTCCTTCCTTTTCCGGTCAATACTTTTAAAGTAAGGTCCTGAAAGGCCTTATTTGGTCTT
GATTGAAGGGCCTGGGTCGCCGGTCCCTCGAAGAGTCCTGACTGCTGAAAGGTCAACCCTTCTAAGAT
AATGCCCTGAAAGGGGTCCTTAAAGGCCTTATTTGGTTGGTCCAGAAAGGTCTTACCTGGATCCTGCC
TGAAAGGGAAACAAGCGATCGATAAGGGGCCTTATCATCAGCGCGGTCAAGCAAGTCAGGGGTGGGTA
CTGGCCCTTGATTTCTTCCCCTCAAAGAACTAGAAGGGGAAGTATTTAGCTTGAGCATAGTTCAGGCC
CTGAAAGGCCCTTCGAAGGCTTGTCCTGATTCACCCTTCTCGCTATTCTTAGCCCCGTAATCTTTATT
TCTATCCTGGTCAATTCGGGTACTTGTTAGAAGGGAGGGATCCCACTCAGGTCATGCCTTCTGAAATC
CCATCCATTTACCTCACATCCCACAGTTCCGCCTTCTGATTTCGGCTCGACAGCGGACCCCATCACAT
TTATTATTATACTTACTGGGAGGGAGGCTGGTATTCCCAGTGGCCATTCTTAGGCTTATTATGTCGCA
CTTGACTCATATCGACCTCTCTTGCTGCTGATCCCGCCTACCTTCCTGCCTTATTAGATAAACCACCT
GATAACCTCCGAACGACTTACCTTCGACCAACTAACTCGACCAACTGCCTGAAAGATATCTTGCCGCT
TGCCTTAGATCGACCTTGCGAACTTCCCCTGATAAAGTCATCGACCGAACTGCCTGCCTTCATCGAAC
TTACCGCTGCCTTTGACCGGACTAACGGCCTGAGAGGAGATATCTTGCTGCCTTATCCCGCCTACCTG
CTTCGACTTTTTACCTGATTGAGACCCTTATTGAGACCCATCTCGATATTGCGACCTGGACGTCTATC
CATTGCAACCTTACAACGATGCGCTTTACACGGATAGCTTAGGCAACTAACTAACCCGTCATCAATAA
GCACATACACAGTTTCACCTGTAGCCCGCACACCTGATGATCAATCTATTGATTGATGCTTGCTGGGA
GAATAAGGGGCTTACAATATAACCCTAAGAAGCTGGTCCCGTGGATGCTAGAAACCGAGGGCTGGGAG
AACCAACGATGGATGAATAGATGGCGGGCTGGCTGGTCTAATCGCCGGGGCTTGCTCTCGCTTGCTCT
GGCTAGATAGATTACAGGCGGGCCCCCCCACCAAATAAGTAAAATGAGAATTTACTATCCAGATTCGG
TCTTTGCCCGATGCGGACAAGCAACTATGTACCAAAGATGGATGGAATATGCTTGCTCGTTAAGAAAG
AAAGAAAGATGCTATTCACCCCTCCTGAGGACCTTACCTGAGCTATCTCGGGAAATAAATAATAATAT
TTAGCATTTTCTTTTGGGTCTGCTCATCTCATGTTCGCCAAGCCGGTTTGCGAGCCGGGAAGAGAAGG
GTCTCTAAGCAGTCCCACCCAACTAAACTATTAATGAAGCAAGTGGGTTCCGTAGTTAGCTCAACAGG
TAAGTAAGGCACGGAGTAGTGCAAGGTATGCGCGCTATAGGAGTGTCTGAAGCAAGGCACTTCAAGTG
AAGGTTGGGTTCTTATTTCCTTCCCCGTCAGCAGGAAAGTTATTAGGGTTGGGTTATCCCCCTGCTCT
TTCCTCTGATGTATAATTACGTATATAATTACGTTTGCTGAAAGTCAGAACAAATCCCTTACTTACGC
GGAGCTCAGGCTTTTAGGGGATTCGGTGATGCGCTTCTTGAACCAGATTGATACTCAATAGTCTATTT
TGGCAAATAGAGGTATTTCCCTCGCTAATTGACCCGGCTAAATGCCTTAGTCCTAATAGATATAGGAA
CAGACTTCCTACTCGGCGTACGCTATTTCAACTCTTCAAAATAAAGGACTATGTTCCTCAAATTGCGA
ATCGACGTATATTTAGCGATGTGTTATCAGCATCTTGGTGGGCCGGCCCATGGTTCTTTTCTTTTACT
GGTGGGCTATCGTCGGCACGGTAAGGTTTAAACACTCTGAACTGCCTGCTGATCGATCCAACGTGATG
CTTGCCGAAGGGTTTTCTCTTGCTTACTTATGCTAACAATATCTAGGGATGTGAACACCGAAAAGACA
CAGTCATGCCTACCTGTGTTTCTCTCTTGCCGCCTGCTTGAGCCTAGAGCTTACCGTCAAGCCTTGTC
CACACAGTAATAGTCCGCGTAAAGCACAGTCAGGACGCCCAGGAAGGCACCCACCAAGTCCGCCCGGT
CCACCTACTCCTACAGCAACAGCACATAGAGCACCTCACATAGATGCCTCACACCATAAGCACCTGAC
ATAGCCGATCATAGCCAGTTGTCGTTGCCAGGAGGTCATTGCCGAGAGCCAGTAGTACTAGCCTTAAG
GTCATTCCAGTTGTCATAGTCAGAAGGAACCGATCATAGCCAGCAGGAGGTCTCGATCATTGCCAGAT
GATCATTGCAATACAGCCAATTGCCAGCCCCTTTACCTTTTATTTGCGGGGCTGGGATACTCGGCTTA
GCTACCGGGCTGGGATGGAAACAGTCGTTGCCTATAAGCTCTAGTCAGTGCTAGAAGCTCTTGTCACT
GCAGCTACAAGCTCTACTCACTGCTGCTAGAACTCTTGTCAGTCAGTGCTAGAAAGAATAAGCTCTAG
TCAGGGAAGCTCTAGCCAGTGCATGAAGACCCAGTGCAGCTAGTCATTGCAGCTACTCAGTCCCAGTC
AGAGCACGGAGCTGCTCACATATACCCATACAAGCAGACGCAGATAACACACGACACAGATAACACAC
ACACCACACAGAGAGACATTCAGAGAGGGCACGTTGAGACATCACTAAAGCCATACAGGAAAGGAAGG
AAGAGAGGGGGCTTTAAATCATTGATTTGCCTACAGACAAATAGGCGGACACCTGGAATCCATCTTAC
CAGCCTTTTCATGACCTTATTTAAATGCGCGGTTGGATACGTTTTTAGTATTGACCGGACCAATCCCA
ATGGATAACTAGAGCCAACTTTGAGTTCTTGATCCCAGATGGAAATATCCTCGGACACCCCAACCAAC
AAGGTTTTACTTTATAAAATGCCTGTTTCAAAACGTTTTTATATCGACCGGAACCACTCCACGAATA
CCAAACTTGCTAGTAGTCGGGTAGCCTATGCACTTTGAATAGAATAGGTTCTCCTTCTAGTTCCAGCG

TTAGGTACACCCATTTTGCATATCTACGTCGTTCATTTTGAACTACCATTTTAATATAGTTGATCCTC
TTTAGCAGCACCCCATCTGAAATCCTGGCTCTGCTCCAGAGAGCGAAGTAAAGTCGTATGAGTTAGGG
CACCGAAGCGAACTCCTTCTGCGGATATCCCATGCCCACCCCTCACCTTGGGGAGTTAGCATAGGGGG
AAGGGACGAATAATGCTGACCTGGGACTGCTGGTTTCTTAGTTCTCTGCTCTCGGAGAAACTTCGGAG
GAGCTGCTTGAAACCCTTAATAAAATGGATTTGATTTCATGCCCAACTTCGCGGAATACCTAAATTTG
AAAAATCGATCAATACATCCATCGTGTGATATAAGCTTAAGTTCTGTACGATATCTGCTTAAAGGTCT
CCCCCTATTGGCTAGGACCATCCATCTTTCGGTGCTGCTTCTAATCGAAGGGATCATAGCAGCCGTCC
CGACTTCCTAAGTGCATTCATGATAGAAGGCTACAGGTCTAGTTCTTCAGGTGGATAGGTTGTATGAC
CCTTAATAAAAGGCAAGGGGCAAGGGTGAGAGGGTTGTTCATAGTAAGAGTGCCGTACCAACCAACAA
AGCATTCATTAAGCAAGTGTTATAAAAGGCTTTTCTTATCCTAGCAATGTCTGAAGCCCGTCCATCCT
CCAACGAGGCTCTCTGATGGCCAATAATAAGAGTTATCCGGCAGTGATATCAGCAGCTAAAGGTAAGT
AAACCCTCCCTCTTTCTTTATTCGAGCCGAGCCTTTATTCAATGCTCTGATCAATTCTCTGTATCTCC
GCATCTCCACATCGGAGAAGTATCCGTTGAAAGTATCCGTTTTTCCTTATTAATTACAAGGAAGCGCT
GTTGATAGGCATTTCTTAATAAAGAGAATGGTCTATTATGGTCTATTAAAGAAAAGAAGGGCTTGCTA
TACCTATGCCATGATCAGTATTAGTGTTCCTGGATCTCCTGCTTCGGGCTTTGCGAGCATTTTAGAAG
TGGGCGCCCGCTATCAATCATGTGCTGCGGGTAAAGGCAGATAAAATGATTGCGGATACAGAGGGGAA
GCTTCAAGAATTCTGGCCCCGAGTTAGCAATGAGTTGGCTTAAGTCCCAGAATACATTCCCGGCTACC
CGCTGGCCACCATAATCGAGTTTATTACGGACGCCTGGGACATGGATACCCCTCTTTGTAGGCGCTTT
ATACTTATAACTCCGAAAGTTGCTTATGTCCCAACCTATGCCTTTGCTCCTGGTACTTTAGCCTATAC
CTCTGTACCTAGCTATGCTATCTGTGCTACTGATGCTACTTGGTCTACTGGGTAAGCTACAGGTACTA
CTGTTGTTGGTGCTGCTGTCTCTGACTCTATATCTCGATCTCGATCAACTTCATTTTTAGAATCAACC
GCTGCAACTGCTGCTCCTGGTGATGGTGCTGGATATACTGGTACTTCTACTGGATCAATAAGGTAAGC
TGGGGCTGGAAGAGATGCTAGGCAAGGTGCTGGCTCTAAAACTAGGAGGGATGCTACTACTAGCTTGG
ATGCTGGCTATCGATCTCGATACGGAAGGAATGCTGAGGGCTACGGATCTATAGGTTCTGGATATCAG
GCCCGAACTGGATCACGATCATGAAGGTAAGACTCTGCTGGATCTCGTTCTTTACAATCATAGTCGTA
AGCGGCTGCGAGATCCACCGCTGGATCCACTGCTACTGATAGCTGTGCATCCATATATGGATATTAAT
ATGGTCTAGCTGGTATAGCTGCTGCCTTTCCTGCAACCGCTGCTCCTGGTTCAAATGCCCGAATTCAT
AAGGGAAAGGTAAGGTGATCCATCCATCCCATCATCCGGTACTTATCCATCCGGTGCTTATAAGATAA
AGTAAGTAGCAGTCCCGACTCACTAAGTGCATTCATTAATCAAGAGATATAGGGATGACCAATCCGGA
AAGAAATCATCGTGAAGGGACGGAGACCAATACCAAGGCGGAGGGACAATTACGAATTAAATGAGTTT
CCTATAGAAAAAAAACAGAGTTCTTGACCCCACAGTAGGCAATGACGCCTTAAGACATCCAAAACACA
TATTGGTAATTCTGAAAAAGTGTGTTTGGTAGGTTGTTTTGTATCGGCCGGACCCCACCCCCGCGAGC
ATGAGCGGGCAGCACCGGACCCCAAAAAAGGAAACAAGATAGAGAGGACAGCCCCCCCGTGGTATGCC
CTTGTTGATGTTAGTGTAATTAATACCGTAACTATCCAAGCCTTTGCCTTTGCTGGCTCAGATGCTTC
TTCCTTCGCTCTAGTTATCTTTGTTTTTTGTGGTCCCGAATCAACAGGCTATGCCTATCGACCTGGAA
GGTACGTTGGGCGGGTTAATGGCTCTCGAACTTACTTGGATGGCGGTGAATCAAAAGGCTCTGGATCT
TAATCTGCCTCTATTGCCTCATAATCACTTCCAAGAACAACGGGCTATGCCTTACAAGCAAGCTATCG
ATCTGGGACTGGAAGGGGCGCTTGTAAGAAAGCTGCCCTAGCTAATGTGATAGGACAGCAGACGACCC
TATTATCGACAGCTGCAAAGGCTCCTCCGACGAATTAGGCATTAGAAGCGCCAAGAAACCCAACCTTG
GTAACAAAGTAACAGTTACACACTAGCGAGAAGCACTGATAGCCGTTACTACACCCTGCCAGTAAAAT
ATCCAAATCCATCTTGCTCGGGAGCCCTTCCATTCCGAGTACCAGAGTCTCTAGATTAAAAACCATCA
TCTCGCCTCGCGTTCCTTCCCGTTTGAAAGGCATAGCCTCCCCCGGATAAAGTCGAATAATAACTACC
GGGGGCAGAGCCAGTAGCAGAAGAGGGAGCAAGGGCAGCAGAAGTAAGGGTAGCCAGAGTAGATACAG
GGACGGGTGCACCATATAATAAATATTTAGACCCAGCTTATGAAGATACAGATCAAGACCCATATTCA
GTAGCAGAGACAGAGTCAGCAGAGCCAGCACTAGCAGCAGTAGATTAAGGAGTCGAAGATCCAACAGC
CAGCTTGCCAGTAGCAAATCCTGCAGAGCCAGTTGCTATGACAGTTCTAGACCCACCAGTATTGGTAG
ATCCAGCAACAAAGCAAGTAGCACCGATAGTAAAGGCCCCTTTAAATGGTTATGCCCTTGGTTACGCC
CTTTATTATGATCTTGATCCACTTGATTATGTAAGTGATTCAGAAGTCGGTGAAGCAGAAGTAGATCC
AGCTTATCCATCATCAGTAGAGGCATAGGTAGCTGGAACATATTCTGTATAGTTTACCCAGTAGCTTA
ATAACCAGTTGGATAGCCAGAACCAGCAGCTAAGACAGTTTATTAAAGAGATCTTGTTGATTCTGTTG
AGTCATAAGCATAAGCAAAGGCATTAGAGCCAGAAGCAAAGGTAGCATAGGTAAGAGCATAAGCAAGT
GCAAGGGCAGGGGAAGCACACGTTGGTGAGTACGTTGATCCAGCAGATTAAGCCTTTGATTCTGACCT
TGATTATGCAGTCCTTGATACAGATCCAAAGCCAGCACGAGTAGCAAAGCTAGTTGTTTACTCATAAG
CAAGGGCAGGTGCCTCAGTCGATCCATATCGAGATCCATACCCAAGTTAAGCTTTTGATCCAGCACCA
GTATCGGTATATGAAGCAGCAGTATATTTATCTATATATTAAGCTGTTGATTCTGTCGACCAATAAGT
AGGACCAGTAGCTTCGTACCTAGTTTACCTTGTTATAGCACGTGTATAAGCTTTTGATCCTGTACCGG
CAGCATACCTAGAAGCATACCAAGCATACCTAGGTACATACCTTCCATTTCAATTGATCCAGATCGAG

AGCCAGCCAGATCCTGCATCTTGGAAGGAAGCGCTTGCTTGGGTGGGCAGGCACTTTCAGCATTGCTT
GCGCACCGCTACGCCCCAGCAGCCTAACTTCCCTTTACCTTAACTGTTACCAACAACTCTGTTATTTT
TACTTGAAGACTCCGTGCCTCACCTTTTGTGGAATATGTGAAGAGAGGGACTAGTATTTATACACCTT
CTTAAAGCCACGGGTGGTAGATCTTCTTTAGAACCGCCTTTAGTTTCTTCTTATTGAGCACTCCGCTA
CGCCCCTCATTCCATGCCTTGAAACAGATAAACAAAGCAGAACTCTGACACCAACGGATGAAGATTAG
TTACTGCCATTTCCTGTTACAAGCCCTGAGCCATAATAAGATAAGTGGTTATCTTAAATAAGCGTTCT
ATGCTACATAAGAGCATTCCAGGAAGAGCTACTTTATCAGCAGTCTATATTTTGCTACATAAGAGCTT
TATTTTAATGGACCTGTGAACTGGTTAAACTGAGGTTATTCTCTCTTATTGATACCCAGTCTGGAACT
GATAACCGTGTCTTTCTTCAGACGATGCATTCTTTGCTTCCGGAAAAGCCCAGTTAGGGCATAAGCAG
TGAAAATAAAAGGTGCACTTCCAAGCGTAGCGAGGGGGATCATCGATCTTCTGATTTGGTTGAATCAC
ACTTGCACTACGACCTCTGCCTGCTATCTTGGTGGGGACTTTAGATGGATCTGCTTCAATTGGTGATG
CTACCTTAGGTGGGGACTTTAGATGCCATAGGAGTGGGGGCTTAAACTACTAATAATTGATGCTTTGC
TTCTAGTATTTGCTTGCTGATCTATCAGTTCTATCAGTGGAAATTGCTGCATCAGTGATGTGTGGGTG
GTGGCATGTGGGCAGCTTACTGTTTGGATATGAAACTGGTACAACGACAACTGGAATTGGTTCTTAGT
CAACTGGCTCTGAAAAAAGCATAATCTACAGGGATTTATCGCTTTCCCGCATCGGGGAAGGGGGCTGG
TTTCACGAATTTGGACTGGTTTCACGAATTTGGACTGGTTTCACGAATTTGGTTTCCTATAAGAGGCA
GGTCTTACCCCCCCGTTGACTGGATTCTGTATTCATCAGGTTGGCAGGGAAGGGCCTGAAACTAATTT
ATATATTGAATTGACTTACTATAACTTATGGATTGACTGATTAATTCACTTATAGATACTGACGAGGC
TGCATTAACTCAAACTTTGATCACCCGCTCTTGGCAAGAAGTTGAGGGAAAATCTTTCTGCAAGGAAC
GGAGTACTCGCCCGACCTAAGAAGGGCGAGGAGCTTTTCGCGGCTCAACTGCTTAGGTTTTAGTTACT
GAAAAGTTCGTTTCTGCCTAGTGCGACGCTAAATCCATTTATTTCACCGTCGATTGAACCAAAAAGAC
GCTTTCTCTGAGTGAGTCTGCCTTTACTGATGCAGGCTCCCTAAATACTCTCGTTATTGTTTGCGCTA
GCTGCGTTGGAACCTTCCCCTTAGGTGGGGCATTGATCCCGGAGTGGAGGATGCCCATTACTTACCGG
GTTTTCCACCCTTTTGATCCTAAGCCAAATCTATTAACCACAGGGACTTCTCGTGTATTATATTATAT
ATGCCTTTTCTGAGCCTTTTTACGAGGACGCCCTTCCTTTTACAGCTCCCCACGCAATCTCCTCTCGT
CTCGTTCTTTGCCTAGCGGGGTTCGGACTTCTATTTATCTTCCCTCAAACGGGCAGTCATCCTGCAGC
GGATAATGCACATTTATTAAGCTTTTGACCTTCCTTTGTAAGCTCTGGTATCTCTGGACCCCAAAGCA
CCTTCCCGTGCTTTATATTATATTAAATCGATCATTAGATCCATAAACCTGGAGCTCTTATACATGTT
CGCATAGATAAGGAAGTAGACTCAAGTAAAGGTCTTACCAGATATACGGTTGAAACCCTTACAGGACT
TACCGGAATAGACTTTCTCTATGCTAAGTTGCCTCGGGCTTGGACCCGCACCTCGTGCTTGTTGGTGC
GCGGATAATCCCTATTGCTTCTCTCGATAGCTAGGGTCGTATCCCCCAGCACTTATTGCTACCTCGTA
TCGTACTTCTATTGCTGGGCTGATAATCTGTCAGTGACCGAACCCAAGAAAGAGAATTGGTCAGTGAT
AAGCTGTGACCCTACTAACCTATAGTTGCAGGTTGAAGCTGTTACCGAAGCAGAAGCAAAGATAAGAT
AGTTGGTCTAATAACATCTTTCCAAGCTAGTTGTTCAGGCAGGTAAGGTATAAGCTTTGAACCAGACC
TCGTGTCCTCGTACCTCGTGGCATGGGGAATACATTCTCACGTGAAACAATAAATGGAGATGCTGTTG
TCTCGTGTGAACTCAACCTGGTATACACGAAGTTGTACAAACAACATGAAAATGGTCTAACCAGATGT
TGTCCAACCAAACCTGGACGAACCAAGCTGATCACGGGAAAACCTATTCTCAATGGGAAGATATACTG
TGCAAAGCTCAACCTCGTATCGTCCCACCAATTGTCCAATAAAAACCGGTATAAACGGAACTTGTACC
ATAAAAGTGGTTAACCCAAACCTCTCTTGAAACTGCTGGTCCAACCGAAACATACCCATCGCTAGCTA
ACGAATCATCCAAGTCATCAAACCAATCAGTTGTCCAATAAACATTGTATAACCCGATCTATGGGACA
CGAACAAATGGTCTAACCCGAACCAGTGGCACACGAACCAATAGCTAATCACGGGAAAAGGAAATAGT
TCTCGTTCCTCGCCCTTCTTAGGTCGGGCGAGTCACTTAACGTACCTCGTGTCCTCGTACCTAGTGTC
ATGGGGAATACATAGTCCCGTGAAACAATATCATGGGAAAGATAAGAAACAGACAAAGTCTCACGGGA
AACCATTAGCAAGAGCTCGCGAACCCTGCAGAGCCAGGTGAAGCCTAAGTAGAATAGAAAACTTGTTT
GTAAAAAGCGATAGCCTTTCGCGGCATGGCCACCGGTAAACCTTTATGTCCTGCGGAGCGAGCCCCCC
GACCCTTGAGTAGCAGGGTTGGTAAGTATGGGAAGGTAGGTAAGTGACTATCCTGTACAGTGTTCTGG
CTGTATCCCCATTAGGTATCTGAGGTAAGAATGTCATTATCATTCCCCTTTAGTAAAGCTTTGTTTGG
GCGGGTAATAGAGGGAAGAGTATTTTGCATTCCAATCGGTAAGAGAGAGAAGATCCCTAGGATGGCAC
CCTTTCACTATCTCTTCACCCTTAAGCATCTTAGCATGCCACCCTTGTATGATCTCTTGACCCTTCCA
ACCGGTAAGGGAGATAGAGGAGCCCGGCCTCCGGAGGAGACACATTTCTCGCCCTAACTATCTAACGC
TTCCTGCGGTATAAGTAAAAGTAAGTGTTTTATGACATGTTCGATAAGGCATTTAGGGATTGCCACTC
AAAGTGAATGAAATAATATTCCTAGCTCATAATAAGAGAAGATATGTAGACTGTAGCTTGGCGGTGGG
TCTTGCTAAAGTATCTTACATCTATCAAATTCCCAAGAGGGATAATTTGCTTGGCGGTAGGTCTTGCT
CAAGGCTCTGGCTTGTCTGATTGTTTAAAAGGAGGGGAAAGCTCCGCAGGACAAATCATGGTAAGGTG
TACCCCTTTTGCTCATAACTTGAAAGACTAGAGATGTACATTAGGCTCTGCCCTGGGGGATCAATACC
ACAATAGAACTAGATCGCTTAAGTAGGAAAAGGAGGAGTCGGTCTAGTGAGTTCAAATAGCATATACT
TGTAAAGGATAAGAAACTTATCCTCGATGACGCATAGGATCTGCTCGGGCTAACCACCTTGAAACTAA

AAAGGATAGTGAGGGAAGGAAAGGAGGGCGAGGTTCCGGCACTAGTAAGGGAAGGTACGAGGCAATAT
GCAGGGAAGGTTAGGATAGAACTAGTAAGTATGTCTCCTCAAAAAGGTAAGTAGGGGCAAGGAGAGAG
AGTACCTTAGCAGCCTATGTCTAATGATCAATAGCACTTTCCTGGGAACTAAATACGACTTCTTCGAA
CTAAAGCATCTTCCTGGAAGTGAGATAAGTGCCCTATAAAGTAAAGAGAGGGAGACTAGAGCTTGGCG
GTAGGTCTTGCTGAAGAATCTTTCTCAAGGGGAAGTATCGGGGGGGATTCTGTGGAGCTACGGGAGCC
CCCCCGAGGAGAGTCCAGTGGGTCTCTTTAATTGCGGTCTAATTACTGAATCTATGCAGTCACTATTA
TTCGTGGGCTGTTGCCCCGGGTGTGTTGGTGGATAGGGCTTAGGGTGCTATACGAACTAGGGATGGGA
GCGTGCTTTATAGATAGGGGAGAAAGCGTATAAGGAGGAAAGCTAGGCAGATGAGATCTTGACAATTA
TGCTCATTATACTGCTCCTGGTTCCAATTAAGCTGGAA
>Repeat_12
AAGCTAGGGTTGGCTCCTCTCAGTTCTCGTCCATCATCCACAAGTAGTGGATCAAAAGCAGAGCTATC
TCCCCCTCTCTCTCCTTCATCCCAATTGCACCGGACGAACTGGTCCTCACCTCACCACACATACCCCT
ATAAAAGCAAAATGAATTTTTATGTGACCCGATAGATAGATCAATATGGTAAAAGAAACTCTTATTAG
TTAGACCGGATATATCCGAGTCACTCTCGCTGTCTGACCCGCCGATGGGACTATCAAAAGAGAAAGGC
AACTTTATCCCCAGGATGGGTTTAATCTAGTATCCCCTGTACCATTGATAAACCGGGACTATCTTCAC
CAGATGTTGACCCAATTAATAGTGTTCGAGACCCATACCTTCGTGATCCATAGCCCATAGAAGCACCA
GTGACAGGTCCAGATCCAGTCGAAGAAGCAGCGGCCCAGGCTGAAGAAGAAGCGATCCGGCAGGATGA
TGAGCAAGCTGACTTAATAAAGTTCTCCTAAGTTCAGGCTTTCGAGAGCAGAGGGCAGGCAGTCAATG
GAAGAGTACTCATGGAAAGAAAGCAGCAATCTTTCAAAGAGTCATGGAAGAGCTCCTCAGATGGTCAG
TCCCTCTTGCATTCAGTCAGTTCCACTTCCCTCTTTCGCGAATATGGAGAATATGACCTGTTTTGTGT
CCATCCCAAGATCCAACCACCTACCCAAGCACAGGAATGTGTGGAATGAAAGGGACTTGAACTGATCT
ACAGTCATTCATTTATACAGGCACTGATACATTCATTCCGGGCTCGAGTGACTGGCTTTCCTTCCTCT
AAAGCCTCCATTTCCATCAAACGGTCTGGATCAGTCAACCTCGCTCAAGTGCTTTCCTCCATGTTGGA
AACTAATCTCTTTAAGGGTACCACAACCATGCAAAAGCCAAAATCTATCACCAATTAGTGGCTACGGC
TCCTTTGTCAGTCCAACCAACACTCGCAGTTCAGCTTCTCCGGCCGTTTTCAAGCTCCCCCCCTCGAG
AACCATTGCAGTTCGGCGCTTTTCCACATCTTCAAAGGCTCCTGCTGGGCTTGGGCACACGTCTCCAT
TCACTTCACTGGGTATCCATTGCTGTTTGTGACGACACTAATCCCTCCCTAGTAAAGAGCTCCCGCGA
ATTCGGGTGTTTACAGAGGGGGGCAGTAGCGATCAATAACCAACAAGGAGGCGAGCGATAATGCGGAA
AACTGTGCCTTCCATTCCAGGGTTGTTTCGTTCATCTCGTAGTAAGTTTGAAACGGGTGTCTTTTACC
CTCTCCCTTAACATGTCCTGGGCGAATAAGTGAATCCACCTAACCCACTGTATGAATCGTATATAACC
AAGCTGTTGTAAACCTAGCACTATCGATCCACCACCTCACACGGCTAATAATCCGTGCCGGCCCGCCT
AACCTCCACGATGTGATCCCTCCTAAACAATATTCTAATCCAAGCGGATGGGTCTTGAGGATTGTTTC
CTGAAAGCGTAATGGGGTCTGGAAGGGTATGCTTTAAATGCGGATTGAATGGAGCATGGGCAGGGTAA
GTTCAATAGGAAAGTTATAAGTAGTAACTGTGTTCCGTACCGTTAGTTTAAGGGGGAAGGTGGGCAGT
GATTCGTCCCCGTCCGTTGGCCCTGCCTAACCTTACAATATGTTTTTTGTTGTCCGTCCGTGGTTGGT
ACCCGCCTATCAATTGGTGAGGAAGTATCCTGTTTCCGGTGGTAACGACTATTGCGTAAGATATTTCA
TTTTATCAGCTTGCATTATATATGCTATTATCCAACAATGGCAATGCTGCTGGTTATTTCTTTCTGAA
TCAATCGTATCTAGAAGCTCGTTCCCCCGATAAGAATCTAAATAGTGCAACCCTTTATTTCATTCCTC
AACCTCAAGCAAGTGGTTGTTAAGGAATTCATCCATTCACAAAGGTTGGGTGAAGCACAGTAAAGTAA
GTAAAGGATGGAAAAATGTGGATAGCATGCACTTCAGTAAAGGTATTCATAGTAAAGATTCTATTCTA
AGTGCTGTACCCACTAAACCCACTAAGCATTATTGCCTGAAGCAAGGTATGGAATGGCATTCATTAAT
TCAATAACAAAGGTAGGGTAAGGGGAAAAGAAGGGTCCCTTTAAATGGTAAAAGTAAAGTAAAAATAA
AGGTAGTAGGCTAAAGGGTGAATAGATTAGTGCCGTACTTCCTTTTCCTGGCTGAAGCAAGTAGTTGG
TAAGGGGAGGCAACACTAAACAAATAGGATGAGGGACGAAGTCAAGTAAAGAGAAAAGCTGGGTGCTC
GGGTGGTAAGACTAAAAGTAAAGGCAAGTTGGGTAAAGGTAATAGCAAAGGTGTGCCCAAACCCTTTC
AATCTCCGTTGGGAGTAGTTCCTTCCTCAATTAAAGAAGGCAGTTCCAAGCTTCTATATGAGATTCAT
TATGAGGGACAGAAGGAAACGAAGGGCTAGAGATCATGATGAAAGGAAAGGGCTCATGATTAAAAGGC
TTGAGAGAAGGATAGAAAAAGTGATAGTGCGCTGTATGTGATACTATCTGTAAGCTGGCTGGCTGGGT
TCAGCCCGACCCAACAAGATCATAGTGCCTACCTCCCTAAGTCTTAATGACGTAAGTGGTTAGCAAGG
GCATGCAACACTCCTTCACTAAGTGGGTTGAGACAGTTCACAAGGTGGTTAACACGTGGATGCACATA
AGTAAAGCAGGGTAGGCTGAGGAAAGTAAATAGTCAAGGGTCTCCTAGCAACTAAAGATTAATACAGT
CCAGTTTAGATACAGGAAGACTACACATAGAGCACAGGCTTGGGGACAACAACCATGCCATGATGGGC
ACTTATGTTTAGTTTTGGTTGATCAAACCATGATGCCTTGAAGCTGGACGAGAGGCACCTACCCCGGG
GAAATGGAATTTCATTTACCATAGGAATCTTATCCCTTTTTATTTGTTCCGGGAGGCAATCGGACGAG
TAACGAAGTGTTTTTATGCTTTTGAGACAACCCGAATTTGACTCCTGACTAGACCGCCTTATTCTGGG
CACTCAGTAGAACCAACAGCCCAGTGGAACCTGATACAGCTTAGCTTCTCAAGCCCCCCACAGTTCTC
GAATCAATCAGTTCTCGTCACACCCAGTGGAACCTGATGGATGCTGCAGTTCACTGAAAGCCCCAGTT

TCAATAAATGCCCACAGTGAGTGGAACCTGATGCTGCTTAAGCTGGCACTAAATCACCTTCCCGGTAC
CTCCCCGCATGAATGGCTCAACCGGACCTCCCCTCTTTCCTTTTCCTCTCAATCTAAGGCCACACCCG
AGCATTCAAGAGATTAACCCCGCAGTTCCATTGCCTACACTCTTGCTAGCTTTGGCTTTGCCCCTACA
CCTGAAGGATATGAAATTGGAATTGCTCGCGCTGAGGGTGGGTCTTGATATCGATTTGGTTAAACCCC
TTTTTCTGTTTAAAACGCTGGTGGTGAATCAACGGACTATGTTGCAATAGATCTCTTTGCCGGGAGAG
AAGAGCGACGCCGGACCCTCCATGTGGTCGGTTCAGTTCGAGGTCATGAAAGGTAAAGTCCAGTGACT
GGTTCATAAAGGGAAGATACTAATGCAATAGCCTGCATAAGCAACTTTCTCCTTCAGGTGTAGGGGCC
ATCTTCTCTGCAGCTTTTGTATTTGCTCTAGGTGCTCTAGGCTATGCTTATACTGGTGCTTCTAGCTC
TGCTACTGATAGCTTAGATGCTGGAACAACTGGTACTGCCTCCGACGCTTCTGCCCTAGCTTATGGCT
TTGCCTCTGTCCCAACAAGCTATGTCTTTGCCTTTGCTACTGATGGTGCTACTGGATATGTTACTAGG
TATGCTGCTGTAGGCTATGCTACTGATGCTTAACCCATAACCATATCCAGTAGTTGTATTACCAGCAG
TTGAACCAATTGATTTCATTGACCGCCCAGTGGAGGTAGTAGCAAAGTCAGTGGTTTCACCCGATCCG
GAACCAATAGTTGATTCGGGACCAGCATTCTCGCCTGTTGGAGAAGTTTGTAATCCAGTTTTAGATCC
TCCAGTCCCGAATACAGATCCAAATACATATGAAGATATAGTTGGATACCCATCAGCAGCAGTGGGGG
TAGGAGCAAACACAGCAGTGGTGGAGGAAGCTAGAGATCTTTGTATGGGTCCAAAGAAAGCATAGGCA
TCATAGGCGGGGGCACTACCAGTGGTTGAAGACCAAGTTGCCAGGGCCCGGAGCAGTTGTTGATCCCA
TTGATCCAGAATCATTTGACCGAGTAAAAGAGACATGGACCATTGGTTGCAGAGCCAATTTAGAGAGC
CAGCATCAGTAGCAGTAAGAGCGTCGACGGGGTAACTAGCAGGGGCGGGTTCACCGGTTGTTGTTGAC
CAAGTTGAACCAATTGAAGCGGAACCCCTTCATTTATTTATCAATTAAACGGGGCAAAACCAAAGGCA
GGATCAGATGCAGCTACGAAGCCCCCAGTTGAACCTATTAGCCAGTCAACCAAGTTGTTTACCTCGCC
CTGATACAGAACGAACAGCAGGAACAAAGGCATAAGTGGTAAAGCCTTACCCTCCGGTCCTCCCATTC
GATCGAACAGCACGGGTGTTGCTGAAGCGACTCATGAATCTATAGTTGTATCCTCTGCCCACGTCACT
GAAAGCCGAGCATGCCCATTCTCCTAGTGCCCGAACCCGCTGACTGAGCTATGACTCACTGAACTATG
AACATGGTAGCGATCCAACGTTGCCACGTCCCCGAACCCCAGCATTTTGAAAGGCATCGAGTGTCCCT
TTTCAATAAATAGGAGGGGTGGATGGTACTTCGAAAGGGATCAATGCTGGCGAGGTTGGCTTCAAGGC
TTGGGATATTGGGCATAAGGCGAGGAGCCAGAGGCGAAGCGAACAAAGGGAGCTCCTTTTATGTATAG
AATTTGCCGAGCAATTTTATGTATATGATTGTGAGCGATATTGGCAATAAACCAAAGAAAGGATCTT
ACTGAAGGGCGAGGTGCCGCAGGTTCTTCGCATGTTCTCATTGGGATACAGGCCATACATAGAATGAC
ATCATCGGGCCTCTCATCGTAATTTGGGTGGACAACCCTAACCATTCAGTTAGAGTGGACAACCCTTC
CTTTCCCATTTATCGCACCAATACTTCTATTACGTATAGAATACTTTGATTATTCGCTACTCTCTCTG
CTAATAATTTGAACTTGACCAGATAACTTGGGTGATTCGCTACTCTCTCTGCTAATAATTTGAACTTC
CACCTATTGCTATCAAGCTTTCGTTTGGAGCAAACTAATTCATTCGAGCAAAGTTCGTTCGAATTCAT
ATATTTTTTTTTATAAGCTTAACCCGTTCTTCAGCGCCTCAGCTTCACCCCTATATCGGTGTGCATAA
AACATCATGGTAATAGATAAGAATGGTAATAGATTCAATTAAGTGTTCGCCCCGAGATGATAGCATAG
TTGACACCATCAAAAGCTACTGCGCGCTGGAGCTATAGCCTTGTACATAGTGAATTCCCGCTAGAATG
GAGATGTTGGTTGAGGAAATGGCATGTGGGTACGAATGGCATATATGGGTACTTAGAAGGCTTGGTGG
TGGTATCCAAATAATATATCATATACGTTATTTCTAATCATGGAGAGAGCCATGGGCACATGCGGACA
GAACCCTCGGCGTATTAGCGACTCCCCAATACGAAAGCCCATTATTGAATCAAAAACCAGGAAGTGCC
GCTCATCGATTGATACAGCTTTTTAGGAAACTAGCTTTATACAACCAACTTTGTACAACCAAGCCGTG
CTTTCGAATCAATTGACGGGAATTAGATAGATAAGGATCAGAGGTTGTATCCTCTGCAAGGATTCCGA
CTAGATAGGGATGCTTGGGGAGACTAGCATTATACAACCAACTTTATACAATTACCGCAAGATTCCTT
TCGAAGTACTATCCAGCTCTAGCCCACACATTTTACCTGACATCCAAGAGACTACCAATGCTCGGTAG
TTCAGGAGGTCGAACTCGAGTTCGGCACTTTGGGGAGCCAAAAAAAAAGATGGAGTCGTTCGCCCATC
TCATCTGTTGGTGGGAGCGTGAAAGCACATCCGTTGGTGAGATACCCTAGTTCCCCAATTATCCTGCA
CAGGAAACTCCTAGGAGCATTTGCCAGTCCCGGGGAACAGACATTTAATTTTCGATACTGGATTTTCG
ATAAGCCTCTCCCCACTACAGCATTTTGGAATTGGAATACCCAGGATCGTAAGTAAGGGCCGGTCCAC
GCAAGATAAGGCACTTTGGTTCGTACGGGTCGAATCCACACGGCACGCACGGTTCGGAAGTACAGGTT
TGACACTGTTCCACTATCGGGAGATCGGAGTATGGGAAATTCCGAATTGGGGGAACATGGGGACTAAG
GGACAGGGGCTTTCTTTCGTTCGTTTCATTCGCTACAGTCGTATTAAACATCTGTATCTTGGTGGTAA
TAGGGAGTGGTTCGATTCCCTTATAGGTGGCGGAACGGGAAAACACGAGCTTGATATCAACATGGGTG
ACGAAACCGGCGATTGATATTCATATTTATACTCACCCATTTATGAATGAGAGCGGGCGGTTGTTCAA
TCCAGGCGGGGCTATCGATTCAGAACTAGCCACTTTCGGGCCCTACCTATATTCGTAGGCCCCGGTCG
GTACGTCGAAAGGGATCAAGGCGATTCAAGGCGAGGGGCGGGGTGCCGCAGGACGAGCTTATTAGAAG
GGGGCGAGCGATGATGCCCAACCAGTATTGAGCTAGCTAGTGGTTGGCCGGCCCCTTTCGCTTCTGCT
TGAGGAGGTAGATTTATTTATGCAGCATTATCCTGGAATTTTCGAGAGCGTTCCCAATCCGATAGCTA
AGGGGTTGTCGCCAACCCTTGATTCCTCGCTCTAGTTACTACATGGAATGGAATAAATTGTAGTGGTT
GAACTGGAACATTCCAAGTGACTAACTCCCCGATGACTCCAAAACTCAAGTTTCTAGTTAACAAATCA

GTTGGTTGAAGGGTCCCATCCACTCACATGAAAATCAACTGACCATGAGAAAGGTTACAAGTAAAACA
TCCAGGGATAAATACCTTAAGGGGATATTCTTATCACTCAGGCATGGTCAATAGATCAAATCATCCAT
TAAAACAAGCATCATCCCCACATGCTTGTGAAGAGCCAATGTTTCCCCCATCCTCCAGTTGCTAAAGC
AATACTAAAATGGTAAAGGATTAGGAAAGGACAATTCCACTAATTTTGATAACATTATAAAATGAAAA
AAATATAAGATTGATGAACGAGGGTGTATCTAGGATCCTTGCTTGGCTTCTTTCTCCATCCATCTAGT
ATTTTACCCCCTCGTCTATGTTCCCTTAAGAAAGTATCAGCCTCTTTGGGGTCATAGATTCTTCCCCC
AATCGACAAACAAAACATATAATTCAAAGCCACAACATCGTTCGTAAATCTTCCAATCAAACAAAGAG
AATTAGTTTTTCGTTATACCTTTGACTTATTACCTGTGACTTTGACTGTTATCCTAAGTCACTTAACA
ACGTCCTTAACGTCACTTAACAACGTTCTTAACGTCCTTTAAGTCCCAAACATTTCATTCCACACACC
TTCAGTTATGGTGGCTCCGCCCAGTACTTACCACAAACGTGCCTATGAATGGCTCCGCCCACTCCTTA
CCGCGATTGAAACAGCTCTGAGGAACAACGAGGAATTAAGCAGTTGGAGAATAGAAAGTAAAGATGCG
GTCTTCCCGGCCCACCCACGCTTCTTTCGCAGAGGTAGTCGAGGGGGGCTCTCTGTGGAGCGATAAGC
GAACCCCCCGATACGGAGCTGGGTTAGGATCACACGACTGATCGGGATCACACGACTGACCTTTTCCT
TCGATTGAACTTCTTTCTTCGATTGAAGAGAGAAAGAGTTTCCTTCCGAGAGATAAGCCGAGTTGGTT
ATCCCTTACCTGATACTTTTCATTGAGGGAAGAAGCTGACTAATTAGCTCATAAATAAACTCTCAGGT
AATAGCTTAACAAGCTTTGAACACGAACTATCACGCGAACTATTAAGCTATTTGGTAGAGTGACATTC
CTCAAACGTAACATTACGAAATGGGGAAGGACATGAACATAAAATGGCAGGTCATGAACATCAAGGAA
CGAACATCAAGTGGCATAAACTAAAAGTCACACAGTAGGTCGTTAGCACGAATGAATGTATATGCAAG
TGCTATTCCTTTCCTTACCGCGTGACATGCCTTACCACCTGAATGGCTCACTGACTGATGAATGGCTC
ACCTGACCGATGGTTGAATGGTTCACCTGACTGCGTGACATTCCTTGCCGTAGGCGTCTATACGTCCC
TGTGCTGGGCTCACCCACGAACTCTTCAGTGGGGCACGAACTATTAAGTTTCCTTCCTGCCACGAATT
CATTCCTGTGCTGGGCTCACCTACGAACTCTTCAGTGCGGCTCTTCAGTGCGGCACACAAACTCTTCA
GTGGGGCACGAACTACTCTTCCTTAACACAAATTATTCGATTGTCATACTTAGTGATGTCTCTAATCT
TCTTTACGGAAAAGGATCCTTCTCTCCCTTTTTCATCTGGTTTGCCGGAACAATTGTGCCAGACCCGA
ACTAACAAGCGGGCTGTCAATTGCTCAATCTGAATCACTGGCTCTATGGGAGACCATAGACTCGGGTA
AGCCTTTCGTGGGTAGTCGGCTCGGAACACCAGATAGGTGATAGCTGCAGGTCCTTTACTCATTGCCC
GAGACTTCGTGAGACTATTAAGGGCATTGAAGGTCCTCTCTTGATGTTATGCAAGTGTGGTACTTAGC
GAAGAGAATCGAGCTGCTCGGAGTTAAGTGAGGAGTAGGACCAAAAGGGATATCCCTTGCAAAGCCAG
GGTTTATTAACCTCCGACTGGCTTTGTTTTTGATACGCCACCTAATGTTAACTATGCAACGTCTATAT
GAAATCATAAAAATAAGAAGGGACTTCTTCTTATCCTTAAACGTACAGGTTCCCGTCTCCGCTTGTAC
GGTCTGGCTTGTAAGTCATAGCTTGTATAGACACTGGAATGACTATAGACACTAGACACTTTCCTGTG
CTTGGGTTGGATAGAGAGAGTAATGGCTTCCCGATTGGATTAAGCCTCCCGATTGAGAGAGTAATTAC
TTCCCGAAGAGTAATGAATGGCTGTAGATAGGAGAGGGCAGGGCTTTCAACTGGTAATAAACTATCCA
TTAAGTAGGCAAGATTGAGGGTAGGAAATAGTAGGCTTGAGGGTAGGGCTTTAAATGAGGGTAGGCGT
GCGTATAAAGGGTAGGCTTTTAAAGTGAGGGTAGGCAATAGCAATAGTAGGCTTGAGGTTGGAATGAA
TGTATACTGCTATACGCTGGCCGGTTCAAAGAGCATCCACATCTTTCGTCGTTTTTATAATAGGGAAG
GGAAGAAGAGTTCTTCTCGATCTCGCCCTTACCTTAGATTAGTTAAGAGAAGACGGGGACATCACCTT
GAGTTATTCTCGATCTTTCCTATCGATGGAGTAGGAGTAGAGATTTATTCCAAGAGTTCTTCTTGATA
GATTTTCCCCTCAATGCGTATGAAAGGTTATCCCTTTCCTGGCCTTCCTCCTGCCAAAGAGATGGGAC
CAACCTCGATTGGGTACGCATGGGTTTGGGTCCGCGACCAGAGAAATGACCACTTGAACGCGCTGTAT
CACCACGAAAATCCTTTTCAGTTACGTTAGGGGCATGGGCTTTCATAAAGGTCATCTTTCGTCGTTTT
CATTAGGCAAGTAGTAGTGTCTTACTGGTATGATTGGGGAATACCGATACATGACCTACGGACTAATA
ACCTATGGCGGGCCCGTTCTGGCCATGGCGGGTAAGATGGCGGGTAAGCATGTCTATTATATTATAGG
CGTGGGTACATATAAAGCGGTGCAAGTATCAATACCCGTAAACGGGGTGGGTATATTCTAGCCCCGCA
GGGCAAAGCAAGTAGGTTCGGCTTACTCCTTCCACTCAATAGATTTTACAAGCTCTCCTAACTATTGG
ATAGGCCATTAAGCCTGGTGGGACCAATGGGATGGTTAAGGCCCGCACTATTGGGTAGGGGTGGGACC
AACCCTTACCAAGGTCTGGTTATCTTGGGTAAGGCAAAGGTCTTGGGTAAGGCCAAGTAGGAGTAGAA
GTAGGCTTAGTGGGACCGCTAAAGGGAGGGCGATAGAAGGTAGGCGTGGTAGGAGTTATCGATTTTTT
CGTGTTCTACGAAATCATTTTCATTCTATCTTTGAACAGACAAACACACTGGTCCAACCTAGCCTCTA
TCATTGAGCTACCGGTGAACACCAATCTATTCAATCTCGACCAATCCCGACCTGGTGAGGTGTGGTTC
CTATGCTACCTATGCCCTATCTATAGTGGGCTATCGGTGCTTACCACTCATTCTCTTTCTCAACAGAT
CCAACCCTCGTTTAGGTGCGGGTGCATACTTAATAGAGGGGAAAGTCCATCTCTACAGCCCTAGCTCA
TGGAGTACCGGTGCTCACCAAGCATTCTATCTATCCACAGCGGCTAGTCCCATCCCATCCGAGGTGGA
TCCCATCTCTACCAATCGAGACAGACTTCTTCGAGGTGGAGCAGCTGGTCAATACCGCAATTACAGGG
TGGAGCAAAAGGATAAAGATAGATTAAGATTCGTTTAAGTGGAATTTCCCTCTCCCTTATTTAGAGCC
TGCTTGCGCTTCCTTGCCCTCCTATCGTCGAGCAAGCAATAAATTCCTCTTTCACCCATTTTGTTAAG
AAGGGGAGGACCTTATCGTGCCGCCCCTCCATTCATAGATTGACCCGCTCCTAGGTTTGTACTCTTAG

AGGTTATGCCCACCAGAGGAGAGGCCTTTAGGTTCGACCTTATAAGTTATATCCGCTTAAATGGTGGC
TCCATTTTAAGGCATTTAATTGCCACTTTCACCCAAGGAATTAAGAATGACCTTACTGGGTGGATTTA
CGTATATTATATATCTGTTCAATATTCCCTAAGAGCTAAAGGATAGATACTCTTTTCCTACCTTATAG
ATTCCGCAGAGGTGTTCTCCTCGATACGGTAGCGAGGAGACTATTCACTTCGAGCGGTAGTGAGAATA
CGATTCACTCGAGGAGCTGTCAATAACGGGTTCTGTCAGAAGTACAAAAACGTTTCAAATGGCTATTT
GATTTCTAAAAAATTCCACTTTCATCCAAGGAAGGTATCAAGACCTTACTGGGTGGGTTTACCATAAA
CTTTCTGTACGTATCGGCATCAAAAGAACTAAGTATTACGTGAGCCAAAGGGAGAGAGGAGATGTAGC
TACAAGTTTCCATTACACGCCTAATTGTACAAGGAAAGAAATCCATTCATTGCTGCTCCATCCGAGAA
TCTAAGGCCAGCTGCTCCACCCGAGAATCTACGGTCGCCATCCGGTCTTTATCCTGCTAGTGAGCGGG
CTTACTCCTGCTGCGGGCTTACTCCTGCTAGCAAGCGGTTGTGGGCCCCACAACTTAGTGGATTGCTT
ATCCATTCTATTGACCAGCTGCTCCACCCATTCCTTGGTTCTGTCTCCCTGTCTCCCAGGACGAGTTA
CCCAGTCTCCCAGTCACCCAGTCAAGGAAGCCAGTCACCCATCCCGGTTGTTGCTCTCATGCCCGAAC
CCACTGATAAGACCTTTCCTTGGTTCGGGACCTTGGAGTACTTGCCTACATTGGAGTACTTGCCTACA
AGGAAGGTATTCGGTCAGCTAAGGTCTTCGATAAGGGCGAGGGTCTTCGATAAGGGTCTTCGTTAAGT
GTGACGAGGAAATAGCCTTCGAGTAAGAGTGCTTGCCTACAATTGAATGGCTTATCGGAGTTACGAAG
TTAGTGGACTTAGTTACGAAGTTAGCGGATAAACTAGTCCAAGCTTGCGGACTATAGCGAAAATATCT
GCTGCTGCAGAGAGAAAAGTACTTACCCACCTACTTACCCACCTACTTACCCACCTTAGAGAGAAAAG
TACTTACCCGTGCTCTTGGGTCACTGAATAGTACATATCTAGGTCTTACCCCAACCCAATAAAAAAAA
CTATATATTTATCAGCTCTTAATAACTCTTTCCTCTCTTTTCATTATCTTGGGTCAGAAAGAGAAAGA
TACTGGGGCATTGAGTCAGACACCCTATAGTAGTAAGGACTACTCCCTCGTGTATACAGGGTGCGTCG
TACAGGTGCCCCCCTCGTGTCTATGCCCTCTCTCGCTTTGATGCTTCGAGGGCTCGCACTCGTAGAAT
TGGGGGGCCCCTGTCCTCCTTCCCTTCTCACTCGTCCAAAGGGGAGTAGTCCTTACTCCTAAGCTACT
GTCCTCACTCTCCCTCAGGGCCATATGAGCTGTACTCATTTCTTCTCTTTAGGCACTGAACAGACTTA
CGGGACATGGACTTATAAGGTGCTATTTATTATATTTTTCTATGGCTTATACCTTCTCTTTTAGCGCC
TTCAAGTGGACTTAAAGTTGCCTTCCTTATGACTAGCAGTAAATAGATTTATTATCTGTGGACCTGTA
ATTAGTAGGAGGGCCTTTAATGAATTAATAGGAGGGCCCGTAAGGAAAAGGCTTTTCATTAATAGGAG
GGCCTTTCCTACCTATGTATTCTAATATGGATATGAAAGACAAGGAAATACATCCATTCATTACAGCC
GATAGGCACTCTTCGGGGTGTCATTCCTTCCACTATAATAGATTCATTGATATTAAATAAGTTGCCTT
CCTTAGGAGGGCCTGCCTACTAATATATTCTGCCTGCCTACTAAGATCTGGAGACCAACGCCACATTC
CGATTCAGGACCTTCGGCATACATACGTGGATTTATTATCTTATCTTTACGGCATACCCACATAGATA
TTAAATAAGTTGCCTTTTGAGTAGGAGGGCCCGTAATATGACTAGCTGGTCGTAACTAGGTTGCGTTT
CTGTGGTAGTCGTGTCGTGGTTGCCTTCCTTATTACTAGCAGGAAATAGATTGATTTATGACTTATAA
TCTGACTTACCTCGCTACCCTAAATTGTAATTCCTCGCTACCCTAAATATGACTTACCACCCGAGAAT
ATAATAACTTTAGTTGTCTCCTTCTTATCCGATCTGTTGGGCGGATCCCAGCGGTTATCGGAGGGGGC
TCCGCCCCCCCGAACCCCCCAAGTTACCGGCATGAGCATGAATAAAATAGTTTCGGGCTAAAGGCTGT
TATCGGGCTAAAGTAGGTTATCGGACTAAAGGCATGGTTTAGAGGGCTAGCGGTTATCGGGCTAAAGG
TCGGGGCTATATGGCTGATACCTTATAAAGGGGTAGTCTTAACCGGGGTAGTCTTATGAAGACCTTTC
CTTACCGGTGAAGACCTTTCTTTACTAACTGGGGAAGACCTTACTAACCGGTGAAGACCTTAATGCCT
TAATTCCTTCCTTATGACTTTCCACCAGAGAATATAAGGAAGAAGAAAGAGATAGATTGGGATAAGGA
AAGATGGGTAGAGAAGATGGGCCATTAAAGGTAGACCAGTAAAGCGACTTCACTAAGGTCTGGTGGGG
AAAGTCCGGTGGGGAAGGACTAGTGGAAGAGATAGATGGGTGGAAGAGAAGGTCTGGTGGGGATTGAG
GGAAGAAAAGAGTATCCTGAAAGGTAAACCCTTAGCTCTGCGTGCGAAGGGTTAACCTTACCTTCTAG
TTAGCTCTTTGAGGGGAAGAAATCAAGGGCCAGGACCTTACCCGCCCGAGGGGTGGGGAAAAGGCATT
CTATAGCTACAATAAGTTAACCCAAGGTCCAGAATTTGTGCAACTTCAATAAGGCCAGAATTTGTGCA
ACTACTGCTTGCTATAGTGAATTTGCCGTAATTCACAAAGCTAGACTATAGTGACAGAATTTGCACAA
ATTCTGAACCTCGGCGTGCGTGGAGCTTTCAAGATGAGGAAAGCCCCCCCCTTTGAATAGATAGGTTT
CGATCACGGGGCGGAGCAAGTCACTTTACCCCCCCCCTTCCCCATTTGCGGTGTTTCACCTTTACCTC
TCTTCGCTACACTTACATGGAATACTGTTTCGCTGCATGCATCTCTGCCCGGTATTGGTATCTCGGAA
TGGAGATCTCGGCTAAGGAAAAATCTCCAGACCCCACACATCATGATGATTAGAATAATAACAGTAAG
AACCCACATTTGGAAAAGTTCTGTTAGGTTCTTAGTAGCAGCCGGCGACCTCCGTTTTTTCTTCTGCT
TTACATAGCTTCTCACAAGGTCTCCTTGATAGCTGGAAGTTCTTCAGGAGTATGAAGAGCTGGAGGAC
TTTGTACCATCCATTCCGGTGTGGTTGGATTATGCTCAACAGCCCGGGGACTTGGAGCACATCTTTTG
TTGTTTCCACCGCCTAAAGTGATTGTTACGACCACGAAGAAACGACGAATCCCAACTACGGATACATA
AGGGCCGAAACTGCTAAGGGCATTCCATCCAGCGTAAGCATCTGGATAATCTGGAATGCGACGTGGCA
TACCCGAAAGCCCCAAGAAATGCATGGGAAAGAAGGTCGAATTCACCCCGAAAAGAGTGATCCGAAGA
TGGATTTGACCTAAAGTTTCAGGGTATGTTCGACCAGGGATTTTACCCACCCGGAAGTGAGATCCTGC
AGATGAAGCAAAAACGGCTCCCATAGGAAGTACATAATGGGAATGTGCAACCACATAATGAGTATCAT

GCGGAGCAATGTCTAGCCCAGGATTTGCCAGGACTATTCCAGTGAGTCCTCCTATGGTGGACGGAAAG
ATGGACCCTGCAGCAGATAACATGGGTGTTTTGTATCGTATCGAACCTCCCCACATGGTAGCGATCCG
ACTAGAGATTTTGATTCCAGTGGGGACAGCTATGATCATGGTAGCTGCGGTAGAGTGAGCACGCGTAT
CAACGTCTGAGCCCACAGTAGACATATGATGAGCCCGAACAGGAGATCCAGGAACACCAATACTGATC
ATGGCATAAACCATGCCTGGATACCCGAATACCGGTTTTCCCGGAAAAGTCGATACGATATGACTAAT
GATACCGAATCCGGGCGGAATGGGAATATACACCTCTGGATGACCGGAGAACCGAAGGAGATGCTGGT
ATAATATCGGGTCTCCCCCTCCAGCAGGATCGGAAAAGGTTGTATTAAAGCTTCGATCGGTTGATAAC
ATGGTAATTGCCCCTGCCGGTACCGGAAGTGATGATAAGAGTAGGAATGCTGTCACTGGAACGGACCG
CACAAATAGGGGTGATCTATGCATAGTCATTCCAGGCCCGCGCATGTTGGGGATAGTAGTTATGAGAT
TGATAGAACCTGAAATGGATGAAACACCTGATAGATGAGGACTAGAAATCGCTGGATCAGCAGCTCCT
CCGGAATGACTGGTAATACCACTTAGGGGCGGATAGACCGTCCACCCAGTGCCGCTACCCACTTCTAC
CGAGGCTGGGCTTAATGGGAGCAACAGCGAAGGTGGCAACAACCGGGATGGAATATTATTCAATCGTG
GAAATGCCATGTCAGGTGCACCTATAGGAATCGGAACGGACCAATTACCAGATCCACCTATCACCGCC
GGCATAACCATAAAAGGGATCATTGGAGAAGCGTGAGCCGTTATTGACACATTATGAGGTTGATGATT
CCCACCAAGAATTTGATCGCCGGGTTGTGCTAATTCCATACGAATTGGTACTGAGAAGCATGTGCCCA
TCACTCCAGCAATGGCACCGAAGATTGAATGTGGAGTCCCTATATCCTTGTGGTTAGTGGGGAACGGC
CATCGAACCAAATTTTTCGTAGAATTCTGATTATTTCGTTTCATTCCTTATCAGAGAGGGGCCGGCCC
CGAGGAGCGGGGCGGCGGCTTATTGGGCGCGCCCCCCCCCACCAGTCAGTGTCTCTTTCTCTTAAGCG
GGTGAGGGTGGTTCTGAGAGGGGGAGATAAACTCCGGAAATAATAACCCTCACCCGGGCTAACAGCCA
GCATTTTTCCAGATCCTTCAATCCAACCAACCAATCCGGGCGGGTAAGGTCCTTCAAGGTCAACCCAG
TCAGGTCCTTATAAACCCTGCTATTTTTAACCCGCGAAGTCAACCCTCCATTGGACGGACCTTATAAT
GCCTCACTGTACCGAATACTTATTTCCTTGATCTCCTTCTGCCTACCGGGGGAGTGGCTCCGCCCCTC
GATCTCCCGTCCGACGGGAGATCGAGTGAGAAATCCCTCACCCTACTATATGGGGGTCGGGTGAGCGA
TTTCATCGATGGCGGCCCTTTCTAAGGGCGAGGTACGTTAAGTGACTCGCCCGACCTAAGAAGGGGGA
GGTACGTTAAGTGACTCGCCCGACCTAACTGACCCTCCCTCTCTTTGAACCTTGTAAGTATTTAACTC
ACAGATAGAAACTTAGTGCCGTTTGATGAGTAACTAAGAACGAGGGAGAGGGATACGGGAAGGATGAA
GTAATGGAAGAGAAAGTAATTGCTAGTAGTAACGACTTGTCACGATCCATTGGTTTATATATCATCCA
TGTTCTAGGTGTATCGGGATACATTATTTTCGCTAAGCGTATATAATAAAATTGGGTGGAAGGGTCCA
CCCCGCGACGAAGGATGGGGTACTAACTGCCCCAAGACCAAGGGGGTACTAACCATTATTTTAGCCCC
TGTCCTCTCCGAACCGCAGGAGATAGTTGCCCATCATACGGCTCACCAACTTCACTTGCTCCGAGAAT
GTTAGGTCGGGGGGGCTCGGGCGGGTTCACAAACGAAGGTCCTCCTGAGCTAGGCTAGTAAGGTCCTG
ATCTTACGCATAGCGAAGCTAACTCAGGCTTTGAGGGGAAGAAATATAAGTGAAGTATGGACTCTATT
TATTCCCCTCAAAGAGCATAGCGTAAGTAGCGGAGCCCATGGTTGGTTTGTTTGGTGAAGAGGCAAGG
GGAAGGGATGGGAAGGAAGGGGCGGGATAAGCCGAATTTTGGCAAATCGATCTATTTGTGTAGGAAAA
GCTTATTTTGCCGTCTCCCTTTTGTCCCCTTTGAGGCTTTTTAGGTTATCCCAGAATGCACTATAGAT
AGATCGTAGTTGCTGAGCTATAAGGTCATCACTTACCTTCCTTCTAGCGGGATAACCTTCCCCTCGCT
ACCGGTCTTACCTGAGCATAGCGAAGGCCTTGAGCTCTTTCTTAAGTGCTTCCCTCCACACCTTTACT
TACCTGCCTGGAAGGGCCTTACCTGAGCGAGTTAGTTCGGGAAGATAAAACCCTTTCTTTTGACCCGG
TCCTCTCAGGCGGTCGGTAAGGTCTTACCGGGTGGGTTGGTCCTTCCTGAAAGCCCTTGATTTCTTCC
CCTCAAAGAGCGCTTATAAAGTGACTCTTTCTTCAAAAGTCCATTTCTTCCAGTCATTCTTTATATAT
GTAATAACACTAGATTCGTGATTTTATGCGATCCGAAAGCGTGAAACTAATGCCCTTTCGCTTCAAAT
GCAGTTCTTCCTCACCTTCATACTTCAGGTGAGCCAATTCTTCCTAAAGTTTCTACTTCAGCAATTAT
TCCAGTCATTCTTTATATATGTAATAACACTCGATTCGATTCGATGTGAGTCGAAGGTACGGAGTCGC
TCGATTGACATGGGAGATCGAGGGGTGAGGGTACGAACCGCTCGCCCGACAGCAGGAGGGAAAGGAAT
GTGAAGTGAATCACCCTAACTTGACGTTCGTGAAACACTTCGTTTCAAGTCATAAGGCAAGCGTAATT
ACATCTATAATGAGATATATCTTTGTATACGATATTATTCGATCATGTTTTCGTTATGATTCGGGTTC
AGAAGACTCGCAAAGCAAAGGAAGTAAGTGCCTTACCTGTTAGGGGCAGTAGTAAACAGACAGTAATA
AAGGCAGTGCGGTAAGTAAGATCGGGTAGTTAACAGTAGGCGTGGCGGGTTGGCGAATAGAATATGAA
TATCTGGATGGAATAATCGCCTTACGATACACCAAATACGAAATAATTACAAGAAAGATAAGGAGAAG
AGTTTCCCACCTTCAGACAGATAAGGAGAAGTAACCCACCTTAACCTTTAGATAAGTAAAAGTACCCC
ACCTTAACCTTAACGAAATAATCACCAAATAACTCACCTACCTTGCAAGGGTTTTTACTTTTCATTCA
TTTAGGTAGGGGCTTGCCATCAAAGAAATATAGGCATCTTAATTGGACTTTTCATAGGCGTTAGAAGA
TGCGAAACCTTGAAACTCAGGTATAACCGGATACTTTGCCATGCCGTATGTCATCCTATTTTCAGGTG
CCCTCAATGCTATGCCTGCTATCTTTCTTTTTTTCCTTGACTGGTCCGAAGCTCGCAGGACCTTGAGC
CAATAAACGGATAGATGGTCGATTATTTGTAGTTACCCTTGCTCCCCCGATTGCTGGATGGCACGGAT
TAGAATTACCCCATACTGCTGCAGTTGGACAGGTTGGATCGTCATGCCTTAGTAATTAATCCCAGAAC
CTTGAGCAGAGATTGTTTCACTGCTGGATAGATTCTCAACTGCCAGAATTTCCGAGACATGACTTCAG

AAACATAGCTGGATGGTCTCGAATAGCCGAGGACTTGTAGTAACATGCATGCTCCTGCGGGAGGATGG
TCAGCCAATAAGGATTGACTTTTAGCTTATATCGTTGAACTGAATCTTCCGGATAGCTCAATTATGGG
CCATCAGAAAGCCTCGTTGGGATCGCATGAGGATTCACCCAACCCTAGAAGTTTTTAACACCTCAGGT
TCAGGGACATGCTATGAAACACTTCCTTTGCCTTTCTTTCACTTTTCAGAAATACACCTGGTGGAGCC
AGCAGATACCTGGAATCACGTTGTCAGTAAGTCAACCATCTAGAGACCTTTGAAGCCTGGGCTGGGTG
GCATCCTGCAATCGTTGGATCGGAATCGGAATTTAGCGCCCTTACCTTTGAATACTAGCCACCTGCCA
GAAAAAGATTCTATGTACAAACCTAGCTACATGCTTACAAGGGAGGGAATGCTCAGACCAGGAGAAGT
GCCGGATCAGATCGGATGATGTGGGAGGGGAATCACCTTACATGCTATCAGCAATTGACTTGTCTACA
TAGAAATGCACGTTTTTATATCGCGGTGGAACCATTAAATGACAGGTTGAATATAGCTAGAAACGCTT
CTCAGAAGCTTTTTGATATCGACCGGACGGAACAACAAAAGGGATTAACATTGCTTTTATATCGTGTA
TATCGTGCTAGAGAGCGCTGGAAATCAGTGACTTGCTTAATGAAACATTACATTTTTAGAACCGGATG
AGACCCATCGGCATTACTTTTATATCTTGCTTCAGGGAAAGGGACGGAGTGAAATGCTTCCTAGATTT
TATATACGTAATTACGATGCGGCAGAAAAGCGAGTGGGCACCTTGCTTGGTTTTACCCGTACTACTTC
GGGCGGGCAACCACACTAGGAACTCATACTTCTTAGGTCGGTCGGGCCAGTTAGTTAATTAATTACCT
TCTACTGCGGCTCCGCTAGCCAGTTACCTAAAGAGAAATTTGAGTTCATGTGAGTTCTCGACCTCGAT
GGGGAATATGCTTTGGACCCCTCTCAACACGAAGCGCGTAGCGACGGAATGGATGGGATGAAGGGCAT
GGAATCACCCAGAGTAACCCCTTCAGTTTTGAATTGGCAGCAGCACATGCTAGAATAAACTACCTTGC
CCCTTTCTTTCACTTGCCAGAGCTAGACCTACCTGGAGCCATAGAACTGAAAGAATCACCTAGCTAAT
GTGAGTCAGGAATCTTGAGCCTTAGAAGCAAGCCCCGGGTTTATGGGAGGACGGATGGAGGAGGAACT
TACCAACAACCCAGCCAGGAATGAGACCAGCAAGATCTGTAGGGGACACTCTTTTCATACCCCTTACC
TTGTTCATACAATACACCTGCTATACATACATTCTATGTAACAGCCCTATATCATGAAGCCTTTAGAG
GGAATGCTATAAGACCAGTTTAAGTGCCGGATCGGATTGGATCATTGGATGCAAATGGGTGAGAATTG
GAATTGGAATCATCCACATGGCCATGGGAATTGGCTTGGGGATGCCTCTTTAGACCAGAATCCTCAGA
GCAGACAGTATACACTATAGAAGCCTTTAATACTTCGACAGGAAGCAGGGGCATCTAGTATACACTAC
CTTTTTACTATTTACCTGCACCCACTTGTAGACCTGGAGCTAGAGACACCTGGAATGCACGTTTTCAG
TTTCAGTGAGTCGATCATCATTAAAAGCACTTTGAAAGCAAGCCCCCGGAAGATGCCATTTATGCTTA
AGAACCTTACCCTTAGACCTTTGAATGCCGAACGAAATAGGCTGGCTAGCACACGATAGAAGAACTTT
ATTGAAACCTGGAAGGGATTAGCTGTAGGCAACTGTTTCAGAAGGCCCCTTACCTTGTTCATACTATA
CACCTCCCAGACAGATATTCTATGTACAGCCCTAGCTAATGAGGGCTTTGGAGGAATCTACAAGACCT
TAGGAGCCACAGACAGATTGGATCGTTGGATCTTGGATGGGAAGGGAATTTGAATCGGTTCATTAAAC
CTTTGAAGCCCCAGGTTTATGCCATGGAGCCACTTACCCAGGAATTTGAGAGACCCAGAATAGATGTT
GGAGAGGGACAGTCTCTTATCAGAAGGCCTTACCTTGTGGAAGGAATACTACCACCTGCCTTAAAGAC
TTTCTACAGCCCTAGCTACATGCTTATAACGGAATGACAAAACCAAGCAGGATCAGTACCGGCCGGAT
CTTATTTTATTTTATGCCAGAGGGGAGGGGAGAGAAGGGAGGATGGACGGAACGACGCGGATTAAATG
CTATCAGTCATATACTTCCCTAAAGAGAATCGCTTTTTAGAACACCCGATAACTGATTACTTTACCCA
GTTAAACAACGGGTTTCTTTATATGAAACATCCCAGATGATAAGCTTTTTCTGATTCACCGGAACCGA
CCTTGTAGCCCCCAATATTGCAGGCACGCTCTGAACAGGCCCGAAAGCTGGACGGAACAAACTATCTT
TTAATAAGAATTACTCTTATTACATATATAATAATATCATATCACACTTTTAACAACACCAGGGCGGG
ATTTGACTGTTACCAACCTTTAAGTCCACCCAGAATAGCTTTACCATATGGATATATTACATTCCATC
CACCCTTCTTACTCTCCTATCAAGACCGGACAACCTAGGACAATTTTGTCATGGAGGATGCCTAATCC
AGGACCTTCATCTTAGAAAAAGCAGAGCAGCACCGGCATTAGGGCATTGAGTCTAGAGAAGCCAGGAA
CAGTCCAGTAGCTGGAAGGTATCGCTTACATACCCTATCACTCTTTCACCTGCTACACAGACTTTCTT
TCTATGGAGTGGAGCAGTCCTAGCTACATGCTGGAAACAGGAATCTACCAGACCAGGACCAGTGCCGG
ATACAATTTGGATGGATGGGCGATAGGAGATCGGGAATCGGAATTTGGAATTGCTTCATTAGACAAGC
CACCTCCCAGACAGAGAGAGATTGTGGGAGCCCCATATCATTAGGTCTTTGTAGGCACTACTAGAAGA
ATCTTTGGAGCAACAACTGATTGGATCTTGCTTCGTGGGACGGGATTTGCAATTGGTTCATCAGACCT
TAGATGCCCTGCTTGCTTTATTGGGTTGCTCTTCTTGCCTTACCCTTATCGATTCTTACTGCTGGAAG
AATAGGCTACGACAATTCAGTGAGTCACCTCAACATCAGATCACTAGGGGACTCTTTACACCTTACCT
TGGTCATACTTGTTGAAGCTACATTTAGGGGCCTTGGAGGGAGGCACTGCTAGAAGAGCTTCATAACA
ACAGATCAGATTGGGATGGATTTAGAAATTCGCGTTATTACATATATAATACACGAACCAACCGACTC
ATTAATTAGATAGACCAGAGAGACCAGGAAGCGAAGATTCATTGCTGTAGGGAACAGTCTCACTATAA
CTTTACTTTGAATACTAACACCTGCTAGACAGACCTTTATCTGGAACTGTACTACAACATGCTGCAAA
GGGAATGCTTTCAGAACAGGACCGGATCATAGGCAATAGGTAGGGCATCGGGTCATTAGACCGAACAA
GCCCCAGGTTGATTCGATGGAGGAACTTACTCTTTAGACCAGGAAGAGATGTAGCGGTAGCGAACCTA
TCACAATCACTTTCCTTGTTATGACAATATACCTCCTAGACAGACTTTATCTCAGCCCTAGAACGAGA
GGTTAGCTGGACCCTTCCTTCCTAGAAGTAGTGGGTGGAAGGATATACTCAGAGGAGCACCAGATTCG
CTTGGTTCATCAGCCCTAGAAATACTTTAGAATCGTAATGCCTTTTACCTGCTGCATGCACCCCTTTC

ATTCAGTTATTACTACCCTACAACTTACAGGCTCTTTACCCACTACAGTTGCTTACAGAACCAGACCC
CTTACCTTGAAACCATACACCTACTAGACAGAAGAGAAAGAAGAAGGGAAGACTTTCTCTGCCCTAAA
ACGAGAGAGAGGTACTTTATACCGGTTACAGGGCACACTCCTAGAAGACACAGCAGCACCAGATTAAT
CGGGTGGGTGGGTTGTAAACAAGGGAAGGTAAGGTCTTATAGCTGTCCCTCCAAAGACTTAATGTTCT
AGGGCTGAGATAAAGGTCTTTCTTTGTGGCAGGTGGTGGCTTGTCTGAACAAGATCTAATGAACCGAT
TAAACATCCATCCCACGATCGATCCCATCAGCAGCTTTTTTAGGTCTACCGGTGACTACCAACACGAA
AATGCTCTGATTCAAGGTCTAGGGCTGAAGATATCCTATCCTAAGGTGGGTGAAAGAGTAAACCAAGA
GTAAGGCCATCCCTTCCAGCTACTGTTTGTTGTGTTCCTGGCTTACTTACTCAATCCCAATCCCAACC
CATTCCATAATGTGGTGCTGCTTCTAAGGTATTCTATTCGTGGTTCTACCCGGTAACTTGAAGGTAAC
TGGACCTCATGGTGGCAGCTACAGAGAAAGTCTTTCTGGCAGGTATCTGGATTGTATGAACAAGTAAA
GGGTATTCTGAGACAGCTGTTCCCTACTACTACAGATTGTCCTGGTCTAATATCTGGGTGGGTAAAGT
AAACATAATCCCACCGCTGTGTCTAGGGTTCTGGGCAGTAAAACCTAAGGGTAAGGCTGATTCATCCA
TATCACCATCAAATAAACCCGGGGCTATTAAGGTCTGATATGGGTCTAACTGGTAACCTAATTCTATA
GGGGTGCTCCATGCGTGGTAAGCCAAGCCCCTTATAAAAGTCGAGTAGACGTAGCCGTGTGCCCAGTC
CCTTGTCCAGCCCCTGATCCAAACCAAGTATCCCAGCCCGATCAACCCAGGTAGGATCATTCAAATGT
TTTCCGTAGCATTAAGTGAGGTCGAGGTATAGGTATATTAATCTTTTTATTCGTCGGGCTATTTCTTT
TGTAAAGCGCGAAGAGTCAACAAGCACAAAGGCCCTCAACCTCCAATTGAATCCATCCTGCCTGTCAT
ATAAAACCTTCCCGAAAGGCCTAGTGGAGTAAGAAGAAAAACCACTTTCATGTGCCGATGATATGTCT
ACCTCACAGAGGCATATTGCCCATTAATTTCCGCTCATCGCTGGGCTTGGATCATTGGAATCATTGCC
TTGGATCATCGGCTCGGCTGCTCAAGCTTGGGATTTTGCCCTTGCCTCATCGGCTGGGCTGGGATACT
TGACTCGGAGCAGGTACTAAGTTCGCTTGGAACTAATTTAACCGCTGCGCCCCTCATTCACCATTCTT
GGCTAAATAAATTGCTCTTTTTAGGAAGCCGAGTGATTTCCTCATCAGAGCAATGAAACGGTATTTTC
TCCTCACTGAACTATACCCTTTCAGATCGTTTACCTATGATTCCCTGGTGGTGATTCGATGACAGGAG
GTAATTCACTCGATGCTGGTAGGAGATCCAAGAGGCGATACAAGTGTTGATGCATTCAATGTAAGGTA
GGTGCCCATGTATTCTTGTCTGGGAACCGATGGAAGACGTCTTCTTGGAACCGATGCGCGATCTTACA
ATGGCACCTCTTTTGAGAAGCTAAGCCCATTTTTGTATAATTACATATATAATGCACGCTTAAAAACT
GACCTGCCAACAAAGGCGTTACCCAGATACCCAGTCCGCATTACACATTCAATCCGCATTACCGCATT
CAGCCCAATCCCCACCAGCAGTTGCAATACCCCGTGCCCTGGAATGAGGTGGCGGTACATTTCAATAA
TAAGTTACAACAGCTTGTTATATACGGAAATATACCCGATATAGCGTAGAAAAGACAATAATCAGGTT
GCGCTGCCGGTACTCATGCTGGTACTCGATCTGGTACTAAATGCCGGTACTCAATTCCAAGAGTCGAG
CCAAGAGGCGATCACTCGATGACAGGTGGTGATCCCAGGAGGAAATGCCAGTCATAGATGGGCTGGGA
TACTCGGCTGCTTGGCTTTTGGCTGCTTGGCTAATCGACTTGGCTTGGATACCGGGATCGGTTTCTCA
GTTGGGATATTCAGTTGGAAACATGAGCTGCTTGGCTGGGATTACTTAGATGGATGGAATGACTTAGT
TAGCAACTGGTCAATTTGAAAGTGAAAGGGTGGCCTATCCAAGTGGTAAATTCAGGAACAAAAGGTGG
CGTGTCAAGATGATATAGAGAAGAGAATGCTCACACGTCCTTGCCCGATGAAGGACAAGACCTACCGG
ACCTATCAATACGCCGCCTGCTTGAGCCTATAAATGCCCTATTTACAACAAACCCTTCCGCCCAGAAC
AGTCCGCGTAAGGCACAGTCCGCCCAGTAAGGCACCCCCCCCTGTAAGTCCAACTGGTCCCATTCCTA
CGGCGCTAGATCTGAGATACACTAGGCGATAGAGCCTTTCATAGTCGATCTTGATCATAGCCAGAAGG
GCCCAATAGCCCAAACCCCAGTAGTTGTAACCAACCAGTCATAGCCAGTCTCATTACCAGAAGGTGCC
AATAAACTTGTTGGAAAGACTTGTCGGGAGGACCTCATTCCCACACGGTCACTGTCAGTCAGACGAGA
GGAGATCCCCACCCCAGGAGGTGCCGATTCCCGATAGTTGATGCTGTCGATCCACTCGATGACAGGTT
GCAATGCCAGAGCTGCGCGGGCTTTTATACTTTACTCGGTTGGGCTTGGTTACTCAGATTGGCTTGGC
CCCTCAACTGGGCTCCTGAGATTGGGGGCTCGGCTACTCGCCTGGACTACCCGGATCGGAGCAGGAAA
TAAGTATTGTTGGAAATCAATTCTAAAAAACTCTTAAGTTACTCATTGGGTGGGGGGGCGACTACTAA
TGGCAAGTCCCTATGGGTCCTCTTTTTGAGGGATCCGAGCATGCATGCATAGCTCATCGGGGCGCTGA
GATGGATTTTCTTCTTTATGAAACATGCCCTTTCAGAACATTTCTATAGAGATTGGCGGGATGGGCAT
CATTTGCTTTTAGCTTGAGCGTCTTTATCCTTGCTCGCTTTACAAATTCACCCATCGAACGATATCAC
ACGATATCAATTATGTTACCCCGTGTTCCCATCCCACCTACCTGCTTGAGATATTGCTGCTTGCCCAT
TAGTACCGCTAACGCTCGATACCCGAACTCTATGGACGGAACACCGTTCCTACAATCCAATGAGGGAA
ATATGACATGCCAATACTACAATAGAGACACTGAACCACTTTCTTTTCACCCGCCCAGGAAGGGCACT
CTCTCTCTCCAACCTGCCCACCTACTTTCAAGCCCGTGTATAGTCGCTTATTCTGGCTTGGGTCGCTA
GATGCCAGCGGTCTTGGGTTGATAGATGCCAGCTACCTAGCCTGTGGATGGATATATTTGGTTATTAG
TTCTAGCATTGGGCCTAGAGAAAGCAAGTAAAGCAGGGAATTAGCAGTTCCGCCTGTAGAAAGCTGTT
CAGCAGTTCTGTTCCGTTCTAAACCGTTCATAACCCAGCAGTTCATCCATTCATAACCCTGAGCCCTC
ACCCTATCGAATCAATTCTATGGGTACTTAAAGAAACAAGGGACCTAAGGAAGAAAAGAAGGAGGCTC
CATCGAATAAACCATGAGGCTCTCAAGGGCGAGGCCCAAGCCGGACCCACTACACTTGCTATTCAGGG
CTATACTCACTATACATTTCAGGGCTACACTGAGGACGTATGAGGTTACACTTACTATTCCTGCCTCT

ACCGGGTATCTAGCCTCTACCTAGCCTCTACGGGGTAGCTATCGAACTGGTTATAACGTTGTCTAAAC
CGGGCTATAGAAGCCTCTGCCGAATAGCTATCGAAGCTGGGTAGCTATAGAGTTAGTTGCCCTGCAGC
TCGTTAGTTGCCGCAGCTAGTGACGCTAGTCGCCCCTAAAGAAGCTATTGACGCTATAGAATCCCTTC
CGTTCACCCTCCATTCACCCTCCTAAATAAGCTCAATAATAATTGACGTAGCAATTGACGTTCTAAAG
CTATTGACATATCAATACGAACGATTTACAACCCTCATTTCAAGCCCTAACCTCATTATACCAGGGTG
GGGTAGCGACCATAACCTGCCTATCACTACTCCATACATAAGCACGTTTGAGGGAAGGAATGGCACTC
GACCAATTGCCATAAAGACCGGTAGTAATGGCCCAATTTACCATACCAATTTCATCTCTATTTATCTC
TCCAACGGTAAGCCGTCGGTACGTACTTCGGGTTTTCATCCATAATCCCAGGAAGGGGCCTTTCCATG
CACGCACGTATCCACCCACATAAGCTAAAAGCGAGTTATTTACCTCCTGATGGAGCCATACCTGTTGG
AACCCCCAAACAACACACCGTGCCTTGTCACCGAACCTTCAGTAGAATATGCGACGGGATAGACTATT
TTATTGATAACCCCTTCTTCTTGCTAGGATTTTCACCCTCAAACCTGCCTGGTAGGTGCCCCAATACT
AGGACCCCCGGTGCCACAAGGAAGGATGGTTCAGACCTTCTTACACTAGGTATCACACTATAAAACAA
CCCCTGATTGGAGGGACAGGTTTAGTCTACAGGAACACAGGAAGTGGTAGGGAGAACAGCTACCACGT
GGGAACTGCTTGATGAATGCGCAGCATTTAAGGTGATTCCTTCCTCTAAGGCTAGTGGGGCAGCATAA
AGAGGCTGGAGCGGGGTAGCCACCTTTTCATTATAGGCAACGCCACCCTTCAGTATAGAATTTATCTC
TTTGCACTAGGGTGAGGGAGTGTGAGAGCGACTTAGGGAGGTAAAGCTAGCTATAGGTCGTTGGGCAG
GAGGCTTAGGCGGGTGAGGAGCTTCAGCTATTAAAAGTCCATTGGGCACCCGAGAAGCTAGTTTCGTA
TTTGTAGATTGGTTTAATCGATCAAAAGCGCTCCCACCTAATGCACGTTTTCTAAATCAAAGTGGTGT
TGTTGGGGATTTGAGTTCTGTAACCCAGAATGGAGGAGGAATATCATGCCCAGGACCCCCGACAAACA
AGGAAGGTCTTCCTTATAAAAAACGCATATCAGAAGGTTTTTGGAATTGGGACCAATCGGCATGGCTT
TTAGATCTCTTGCTTCAGGGCATGGCTCGTTGTCTATTGCTTTAAATAAATTTCAATTGGTACATTGA
AGCCAGATAGACAACCTTTCATTGGTGCCAATAAGAGACAACAGCTTTTCAACAGCTTTTAATTAGTG
TCAATTAGTGTCAATAAGAGACAATAGAGAGGCAAGCTAGAACAGAGGCAACTAACTGCAATTCAAGC
TAGAACAACCAACTGCAATTTCTATTTTCATTAGTGTCAGTAAGCCAGAGAGCCTGCCAGCCAGTCTT
TCAACTCTGCAATTCAATCTAGAACAGATAGGCAAGCTAGAAACAGTAAGCTAGTCAGTCTTGCCAGT
AAGTCTTGCCAGAGAGGCAACCAACTACAATTTATTAGTGTAAATTAGTGCCAATAAGCCTTTCAACT
AACTGCAATTTATAAGTGTAAATAAGAAGAACAGAGAGGCAAGCTAGAACAGAGAGCCTGCCAGTTAG
CCTGCCAATAAGAGACAACAGAGAGGCAAGCTAGAACAGACTGCCAATAAGCTATTCAATTTCATTAC
ATTTGTAAAGCCTTTTAGCAGCAGTTTCAGCACTACTTTAACCATCCTCATTCCCAGCAAGCAGAGTA
GCACCAGCAGCACTAGTAGAACCAGCATTATGATTATCAGTTGTTCCAGGATCATTTCCATCAGCAGT
TGAGCACGGTCATTTATAAGAAACGGAATAATTCCCAGCAGAACAAGGATTCCCAGCCCAAGCAGCAA
AAGGAATCCCAGGATCATTTGAACCAGCATTCAAAGCAGCATTAGAACCAGCAGCCCTAGCTGCAATT
GGACCGGCATCATGACAAGGAGCAGTTGAACCAGCAGAATCAGAAGTAGTTCCAACACCAACTAGTTG
AATAGGACCAAGAGTAGCAGTCCCAGGATCATTCTTCCAAGCAGCATAACCAGTAGAAGCATCACCAA
TTGAACCGACAGCAGCCCAAGCAGAACGACCAGCACTATGACCAGCATTTAAAGCAGCAGCCGAACCA
GGAGCACTAGGATTCCCAGCAAAAAAAGCATAATGATCAGGAAGGACCACTAAAACCAGTGAACCAAT
AAAGGGCAGGCCAAGCATTATTGAAATCAGCATTATCAGGAGCAGCATTACCATCAGCATTCTTCCCA
GCATTCTTCCCAGCATCAGTGGAACCGGCAAAGGCAGCACAAGCTGAACAACCAGCACAAGTATGATT
CTCAGTACGAGCAGCACAAGGAGTCCCAGCATATTAACCAGTTACCGTAGCAGTTTAAGCCTAAAAAC
CAGGAGCACTACAATCCACTAAGCTTGCTGCTTTTCAGAGATCGATCCGCTCTTTCCCAAAGCAAGGA
CTATGCCATGAAACCCATTGTCGAGTCATTATCTTCATACCTATTGTATGTATGGCTTTCTTCCATCG
CTGTTCAATCTCTTTTATAGAGCAAAAACTCTTTCCACTTCACTTGTGTTACGGAGAATGCAATGCCG
CGCTTAACATCATGTTCTGATGAAGCCGGGTTGACTTGAGAAACGGGGCTCCTTTTGTTCAACTCCGT
GCACATCAAACGTTATATGTGCTTGGGAAGCGGAAGGACAACCATAGTGCAGCGTAGCGGAAGGGGCT
TTAGGTGAGCTCGTAACAGCAAACAGACATGAACTTGCCTATGTTGCTCCAGTTCTTCGCTGTTCTAA
AGTTATTCATTTAGGGTGTAGGGCACTTAGCTTTACGTCATTTATCTCAGTGTGCTTTTAGAGACTTT
AGTTATACGGCTCAGCCTAAGAAGATTAATTAGGGAGGCGATGAATGTGGGTTTACAAGCACATTGTT
CGTTCTGTCGAACTCGGCTTTTTGAAAAGCTTCACCCCGAGTTCACTTTACACCTTACGCCTTACACG
TTTGGATTTGTTTCATTATGGTTAGTTCCAAAAGAACTGGTAAGATTCAGATTCTACGATTGACTCAT
ATATAATATAGTGAGTGGCTTGCTTGCATAACTACTCTTTCTACTATGCAGCAGCCCACCTAACTAAT
TTCTGGCATGTGAGAAGATATAAGATGATTCTAGGTCGTTTGGAAGATGCAGTTCGAAAAACTATACA
CAGAGCAACCAAGCGTAGATCAACTCCTCAGAACTAAGTGAACTATATTTCTTCCCCTAAAATCGCTA
GCTAGAAGGTAAGGTTATCCCTATATTTATTCCCCTCAAGAGTGAGGACTTGTTTCCGATGTCACAGG
CTATGAACCTGTCCGCACCAAAGAGTGGAACGCCATCAATTGATCACGTTCCTTAATCTCTTCTCTAT
TTCTACACTATTCTTCTTGGCCTTGGCTTTGGCTGTACCTACTTTTTTTCGCTTGGCCTTATTGCTTG
CGTGCGCGAACTAAGGCAATAGTAGGTGAGTTAGCGTGTTACGATAGAATCCTGGTAGATCCAGTCAA
GTCCTGATTCAATACGTTAAAGCAATACTTCGTCAATTAAGAGCCATTGTTTTTATGGAGTTATGTAC

TTACTAACTTTACTTATTTCCTTGGCTTGGCCTTGGCCTTATTCTATCTCTAGTTACCGAAAGCATAC
GAGTCATTAAAGCCATACGGGTCGCAAAATAGCAGAGCTACATAAATACTTTCCAGGTAGGTGTACAC
TTCGTTACATATTGCAGGTCCGCCTTAAGCATCCTCTCTGCCGTGGTCCAGCAACCAACCCTATTTTG
GTTTTGAAGGTGTTCGCTATACTTTCCGTCCCTCTCAAGTTGTTTCTGAACAGTGAAAGGAATCCCCC
AGCTATTCTCTCTCCCATATGTATGTTCCCGGTCCGCTTTCCCTCTTACCGACCGGTATGATGAGAAA
GGGCGCTTGACCGATTTCTAAGGGTACGGAATCGGGTGATCAACTTTTCTTTCTTCCCCTCAAACTAT
CGTTCTGTCTGACTGAACCAAACCATAGAAGGCCGCACTCGACACACAACGGAGGGAGAGGGGATGGG
GGAGATAGGACAGGCAATCCCTAGTTCCGGGACAGCCAGCACCGCACGGAAACTCACACGAGCGAGAG
TGCCAGCCCACCGGTTCTGAAAGGACCTTATCTCATGAACTTTTCGAATACTTCGTGGAAGGTCAGCT
CTAGAGGGAGGGCAGACGGCCCTGAAAGGTCCAGCCAGTAAGGCCCTAACCCGGCCGGCTTCGACTTG
TCCTGATGGACCCGTCTTACTTAATCATTTGTTTTATTCTGGTTTTGCGATGTAAATCCAGTCAAGTC
TCGATCCTTCTAATGCGTGCGATTGTTTCTACGGATGGTGTGGTGTTTTTTCCTTATCCTTATCCTTA
TCCTGATTCTTTTCCTGGCCCTGGCCCTGATATCCGAAAAGGAAGGAATGAAGTCCGTACTATGCCAT
ACTTCCCTCGTGAAGTAAGGGGCAACATACATAAGGTTCTGATCTCTTTCCTTCCTTTTCCGGTCAAT
ACTTTTAAAGTAAGGTCCTGAAAGGCCTTATTTGGTCTTGATTGAAGGGCCTGGGTCGCCGGTCCCTC
GAAGAGTCCTGACTGCTGAAAGGTCAACCCTTCTAAGATAATGCCCTGAAAGGGGTCCTTAAAGGCCT
TATTTGGTTGGTCCAGAAAGGTCTTACCTGGATCCTGCCTGAAAGGGAAACAAGCGATCGATAAGGGG
CCTTATCATCAGCGCGGTCAAGCAAGTCAGGGGTGGGTACTGGCCCTTGATTTCTTCCCCTCAAAGAA
CTAGAAGGGGAAGTATTTAGCTTGAGCATAGTTCAGGCCCTGAAAGGCCCTTCGAAGGCTTGTCCTGA
TTCACCCTTCTCGCTATTCTTAGCCCCGTAATCTTTATTTCTATCCTGGTCAATTCGGGTACTTGTTA
GAAGGGAGGGATCCCACTCAGGTCATGCCTTCTGAAATCCCATCCATTTACCTCACATCCCACAGTTC
CGCCTTCTGATTTCGGCTCGACAGCGGACCCCATCACATTTATTATTATACTTACTGGGAGGGAGGCT
GGTATTCCCAGTGGCCATTCTTAGGCTTATTATGTCGCACTTGACTCATATCGACCTCTCTTGCTGCT
GATCCCGCCTACCTTCCTGCCTTATTAGATAAACCACCTGATAACCTCCGAACGACTTACCTTCGACC
AACTAACTCGACCAACTGCCTGAAAGATATCTTGCCGCTTGCCTTAGATCGACCTTGCGAACTTCCCC
TGATAAAGTCATCGACCGAACTGCCTGCCTTCATCGAACTTACCGCTGCCTTTGACCGGACTAACGGC
CTGAGAGGAGATATCTTGCTGCCTTATCCCGCCTACCTGCTTCGACTTTTTACCTGATTGAGACCCTT
ATTGAGACCCATCTCGATATTGCGACCTGGACGTCTATCCATTGCAACCTTACAACGATGCGCTTTAC
ACGGATAGCTTAGGCAACTAACTAACCCGTCATCAATAAGCACATACACAGTTTCACCTGTAGCCCGC
ACACCTGATGATCAATCTATTGATTGATGCTTGCTGGGAGAATAAGGGGCTTACAATATAACCCTAAG
AAGCTGGTCCCGTGGATGCTAGAAACCGAGGGCTGGGAGAACCAACGATGGATGAATAGATGGCGGGC
TGGCTGGTCTAATCGCCGGGGCTTGCTCTCGCTTGCTCTGGCTAGATAGATTACAGGCGGGCCCCCCC
ACCAAATAAGTAAAATGAGAATTTACTATCCAGATTCGGTCTTTGCCCGATGCGGACAAGCAACTATG
TACCAAAGATGGATGGAATATGCTTGCTCGTTAAGAAAGAAAGAAAGATGCTATTCACCCCTCCTGAG
GACCTTACCTGAGCTATCTCGGGAAATAAATAATAATATTTAGCATTTTCTTTTGGGTCTGCTCATCT
CATGTTCGCCAAGCCGGTTTGCGAGCCGGGAAGAGAAGGGTCTCTAAGCAGTCCCACCCAACTAAACT
ATTAATGAAGCAAGTGGGTTCCGTAGTTAGCTCAACAGGTAAGTAAGGCACGGAGTAGTGCAAGGTAT
GCGCGCTATAGGAGTGTCTGAAGCAAGGCACTTCAAGTGAAGGTTGGGTTCTTATTTCCTTCCCCGTC
AGCAGGAAAGTTATTAGGGTTGGGTTATCCCCCTGCTCTTTCCTCTGATGTATAATTACGTATATAAT
TACGTTTGCTGAAAGTCAGAACAAATCCCTTACTTACGCGGAGCTCAGGCTTTTAGGGGATTCGGTGA
TGCGCTTCTTGAACCAGATTGATACTCAATAGTCTATTTTGGCAAATAGAGGTATTTCCCTCGCTAAT
TGACCCGGCTAAATGCCTTAGTCCTAATAGATATAGGAACAGACTTCCTACTCGGCGTACGCTATTTC
AACTCTTCAAAATAAAGGACTATGTTCCTCAAATTGCGAATCGACGTATATTTAGCGATGTGTTATCA
GCATCTTGGTGGGCCGGCCCATGGTTCTTTTCTTTTACTGGTGGGCTATCGTCGGCACGGTAAGGTTT
AAACACTCTGAACTGCCTGCTGATCGATCCAACGTGATGCTTGCCGAAGGGTTTTCTCTTGCTTACTT
ATGCTAACAATATCTAGGGATGTGAACACCGAAAAGACACAGTCATGCCTACCTGTGTTTCTCTCTTG
CCGCCTGCTTGAGCCTAGAGCTTACCGTCAAGCCTTGTCCACACAGTAATAGTCCGCGTAAAGCACAG
TCAGGACGCCCAGGAAGGCACCCACCAAGTCCGCCCGGTCCACCTACTCCTACAGCAACAGCACATAG
AGCACCTCACATAGATGCCTCACACCATAAGCACCTGACATAGCCGATCATAGCCAGTTGTCGTTGCC
AGGAGGTCATTGCCGAGAGCCAGTAGTACTAGCCTTAAGGTCATTCCAGTTGTCATAGTCAGAAGGAA
CCGATCATAGCCAGCAGGAGGTCTCGATCATTGCCAGATGATCATTGCAATACAGCCAATTGCCAGCC
CCTTTACCTTTTATTTGCGGGGCTGGGATACTCGGCTTAGCTACCGGGCTGGGATGGAAACAGTCGTT
GCCTATAAGCTCTAGTCAGTGCTAGAAGCTCTTGTCACTGCAGCTACAAGCTCTACTCACTGCTGCTA
GAACTCTTGTCAGTCAGTGCTAGAAAGAATAAGCTCTAGTCAGGGAAGCTCTAGCCAGTGCATGAAGA
CCCAGTGCAGCTAGTCATTGCAGCTACTCAGTCCCAGTCAGAGCACGGAGCTGCTCACATATACCCAT
ACAAGCAGACGCAGATAACACACGACACAGATAACACACACACCACACAGAGAGACATTCAGAGAGGG
CACGTTGAGACATCACTAAAGCCATACAGGAAAGGAAGGAAGAGAGGGGGCTTTAAATCATTGATTTG

CCTACAGACAAATAGGCGGACACCTGGAATCCATCTTACCAGCCTTTTCATGACCTTATTTAAATGCG
CGGTTGGATACGTTTTTAGTATTGACCGGACCAATCCCAATGGATAACTAGAGCCAACTTTGAGTTCT
TGATCCCAGATGGAAATATCCTCGGACACCCCAACCAACAAGGTTTTACTTTATAAAATGCCTGTTTC
AAAACGTTTTTTATATCGACCGGAACCACTCCACGAATACCAAACTTGCTAGTAGTCGGGTAGCCTAT
GCACTTTGAATAGAATAGGTTCTCCTTCTAGTTCCAGCGTTAGGTACACCCATTTTGCATATCTACGT
CGTTCATTTTGAACTACCATTTTAATATAGTTGATCCTCTTTAGCAGCACCCCATCTGAAATCCTGGC
TCTGCTCCAGAGAGCGAAGTAAAGTCGTATGAGTTAGGGCACCGAAGCGAACTCCTTCTGCGGATATC
CCATGCCCACCCCTCACCTTGGGGAGTTAGCATAGGGGGAAGGGACGAATAATGCTGACCTGGGACTG
CTGGTTTCTTAGTTCTCTGCTCTCGGAGAAACTTCGGAGGAGCTGCTTGAAACCCTTAATAAAATGGA
TTTGATTTCATGCCCAACTTCGCGGAATACCTAAATTTGAAAAATCGATCAATACATCCATCGTGTGA
TATAAGCTTAAGTTCTGTACGATATCTGCTTAAAGGTCTCCCCCTATTGGCTAGGACCATCCATCTTT
CGGTGCTGCTTCTAATCGAAGGGATCATAGCAGCCGTCCCGACTTCCTAAGTGCATTCATGATAGAAG
GCTACAGGTCTAGTTCTTCAGGTGGATAGGTTGTATGACCCTTAATAAAAGGCAAGGGGCAAGGGTGA
GAGGGTTGTTCATAGTAAGAGTGCCGTACCAACCAACAAAGCATTCATTAAGCAAGTGTTATAAAAGG
CTTTTCTTATCCTAGCAATGTCTGAAGCCCGTCCATCCTCCAACGAGGCTCTCTGATGGCCAATAATA
AGAGTTATCCGGCAGTGATATCAGCAGCTAAAGGTAAGTAAACCCTCCCTCTTTCTTTATTCGAGCCG
AGCCTTTATTCAATGCTCTGATCAATTCTCTGTATCTCCGCATCTCCACATCGGAGAAGTATCCGTTG
AAAGTATCCGTTTTTCCTTATTAATTACAAGGAAGCGCTGTTGATAGGCATTTCTTAATAAAGAGAAT
GGTCTATTATGGTCTATTAAAGAAAAGAAGGGCTTGCTATACCTATGCCATGATCAGTATTAGTGTTC
CTGGATCTCCTGCTTCGGGCTTTGCGAGCATTTTAGAAGTGGGCGCCCGCTATCAATCATGTGCTGCG
GGTAAAGGCAGATAAAATGATTGCGGATACAGAGGGGAAGCTTCAAGAATTCTGGCCCCGAGTTAGCA
ATGAGTTGGCTTAAGTCCCAGAATACATTCCCGGCTACCCGCTGGCCACCATAATCGAGTTTATTACG
GACGCCTGGGACATGGATACCCCTCTTTGTAGGCGCTTTATACTTATAACTCCGAAAGTTGCTTATGT
CCCAACCTATGCCTTTGCTCCTGGTACTTTAGCCTATACCTCTGTACCTAGCTATGCTATCTGTGCTA
CTGATGCTACTTGGTCTACTGGGTAAGCTACAGGTACTACTGTTGTTGGTGCTGCTGTCTCTGACTCT
ATATCTCGATCTCGATCAACTTCATTTTTAGAATCAACCGCTGCAACTGCTGCTCCTGGTGATGGTGC
TGGATATACTGGTACTTCTACTGGATCAATAAGGTAAGCTGGGGCTGGAAGAGATGCTAGGCAAGGTG
CTGGCTCTAAAACTAGGAGGGATGCTACTACTAGCTTGGATGCTGGCTATCGATCTCGATACGGAAGG
AATGCTGAGGGCTACGGATCTATAGGTTCTGGATATCAGGCCCGAACTGGATCACGATCATGAAGGTA
AGACTCTGCTGGATCTCGTTCTTTACAATCATAGTCGTAAGCGGCTGCGAGATCCACCGCTGGATCCA
CTGCTACTGATAGCTGTGCATCCATATATGGATATTAATATGGTCTAGCTGGTATAGCTGCTGCCTTT
CCTGCAACCGCTGCTCCTGGTTCAAATGCCCGAATTCATAAGGGAAAGGTAAGGTGATCCATCCATCC
CATCATCCGGTACTTATCCATCCGGTGCTTATAAGATAAAGTAAGTAGCAGTCCCGACTCACTAAGTG
CATTCATTAATCAAGAGATATAGGGATGACCAATCCGGAAAGAAATCATCGTGAAGGGACGGAGACCA
ATACCAAGGCGGAGGGACAATTACGAATTAAATGAGTTTCCTATAGAAAAAAAACAGAGTTCTTGACC
CCACAGTAGGCAATGACGCCTTAAGACATCCAAAACACATATTGGTAATTCTGAAAAAGTGTGTTTGG
TAGGTTGTTTTGTATCGGCCGGACCCCACCCCCGCGAGCATGAGCGGGCAGCACCGGACCCCAAAAAA
GGAAACAAGATAGAGAGGACAGCCCCCCCGTGGTATGCCCTTGTTGATGTTAGTGTAATTAATACCGT
AACTATCCAAGCCTTTGCCTTTGCTGGCTCAGATGCTTCTTCCTTCGCTCTAGTTATCTTTGTTTTTT
GTGGTCCCGAATCAACAGGCTATGCCTATCGACCTGGAAGGTACGTTGGGCGGGTTAATGGCTCTCGA
ACTTACTTGGATGGCGGTGAATCAAAAGGCTCTGGATCTTAATCTGCCTCTATTGCCTCATAATCACT
TCCAAGAACAACGGGCTATGCCTTACAAGCAAGCTATCGATCTGGGACTGGAAGGGGCGCTTGTAAGA
AAGCTGCCCTAGCTAATGTGATAGGACAGCAGACGACCCTATTATCGACAGCTGCAAAGGCTCCTCCG
ACGAATTAGGCATTAGAAGCGCCAAGAAACCCAACCTTGGTAACAAAGTAACAGTTACACACTAGCGA
GAAGCACTGATAGCCGTTACTACACCCTGCCAGTAAAATATCCAAATCCATCTTGCTCGGGAGCCCTT
CCATTCCGAGTACCAGAGTCTCTAGATTAAAAACCATCATCTCGCCTCGCGTTCCTTCCCGTTTGAAA
GGCATAGCCTCCCCCGGATAAAGTCGAATAATAACTACCGGGGGCAGAGCCAGTAGCAGAAGAGGGAG
CAAGGGCAGCAGAAGTAAGGGTAGCCAGAGTAGATACAGGGACGGGTGCACCATATAATAAATATTTA
GACCCAGCTTATGAAGATACAGATCAAGACCCATATTCAGTAGCAGAGACAGAGTCAGCAGAGCCAGC
ACTAGCAGCAGTAGATTAAGGAGTCGAAGATCCAACAGCCAGCTTGCCAGTAGCAAATCCTGCAGAGC
CAGTTGCTATGACAGTTCTAGACCCACCAGTATTGGTAGATCCAGCAACAAAGCAAGTAGCACCGATA
GTAAAGGCCCCTTTAAATGGTTATGCCCTTGGTTACGCCCTTTATTATGATCTTGATCCACTTGATTA
TGTAAGTGATTCAGAAGTCGGTGAAGCAGAAGTAGATCCAGCTTATCCATCATCAGTAGAGGCATAGG
TAGCTGGAACATATTCTGTATAGTTTACCCAGTAGCTTAATAACCAGTTGGATAGCCAGAACCAGCAG
CTAAGACAGTTTATTAAAGAGATCTTGTTGATTCTGTTGAGTCATAAGCATAAGCAAAGGCATTAGAG
CCAGAAGCAAAGGTAGCATAGGTAAGAGCATAAGCAAGTGCAAGGGCAGGGGAAGCACACGTTGGTGA
GTACGTTGATCCAGCAGATTAAGCCTTTGATTCTGACCTTGATTATGCAGTCCTTGATACAGATCCAA

AGCCAGCACGAGTAGCAAAGCTAGTTGTTTACTCATAAGCAAGGGCAGGTGCCTCAGTCGATCCATAT
CGAGATCCATACCCAAGTTAAGCTTTTGATCCAGCACCAGTATCGGTATATGAAGCAGCAGTATATTT
ATCTATATATTAAGCTGTTGATTCTGTCGACCAATAAGTAGGACCAGTAGCTTCGTACCTAGTTTACC
TTGTTATAGCACGTGTATAAGCTTTTGATCCTGTACCGGCAGCATACCTAGAAGCATACCAAGCATAC
CTAGGTACATACCTTCCATTTCAATTGATCCAGATCGAGAGCCAGCCAGATCCTGCATCTTGGAAGGA
AGCGCTTGCTTGGGTGGGCAGGCACTTTCAGCATTGCTTGCGCACCGCTACGCCCCAGCAGCCTAACT
TCCCTTTACCTTAACTGTTACCAACAACTCTGTTATTTTTACTTGAAGACTCCGTGCCTCACCTTTTG
TGGAATATGTGAAGAGAGGGACTAGTATTTATACACCTTCTTAAAGCCACGGGTGGTAGATCTTCTTT
AGAACCGCCTTTAGTTTCTTCTTATTGAGCACTCCGCTACGCCCCTCATTCCATGCCTTGAAACAGAT
AAACAAAGCAGAACTCTGACACCAACGGATGAAGATTAGTTACTGCCATTTCCTGTTACAAGCCCTGA
GCCATAATAAGATAAGTGGTTATCTTAAATAAGCGTTCTATGCTACATAAGAGCATTCCAGGAAGAGC
TACTTTATCAGCAGTCTATATTTTGCTACATAAGAGCTTTATTTTAATGGACCTGTGAACTGGTTAAA
CTGAGGTTATTCTCTCTTATTGATACCCAGTCTGGAACTGATAACCGTGTCTTTCTTCAGACGATGCA
TTCTTTGCTTCCGGAAAAGCCCAGTTAGGGCATAAGCAGTGAAAATAAAAGGTGCACTTCCAAGCGTA
GCGAGGGGGATCATCGATCTTCTGATTTGGTTGAATCACACTTGCACTACGACCTCTGCCTGCTATCT
TGGTGGGGACTTTAGATGGATCTGCTTCAATTGGTGATGCTACCTTAGGTGGGGACTTTAGATGCCAT
AGGAGTGGGGGCTTAAACTACTAATAATTGATGCTTTGCTTCTAGTATTTGCTTGCTGATCTATCAGT
TCTATCAGTGGAAATTGCTGCATCAGTGATGTGTGGGTGGTGGCATGTGGGCAGCTTACTGTTTGGAT
ATGAAACTGGTACAACGACAACTGGAATTGGTTCTTAGTCAACTGGCTCTGAAAAAAGCATAATCTAC
AGGGATTTATCGCTTTCCCGCATCGGGGAAGGGGGCTGGTTTCACGAATTTGGACTGGTTTCACGAAT
TTGGACTGGTTTCACGAATTTGGTTTCCTATAAGAGGCAGGTCTTACCCCCCCGTTGACTGGATTCTG
TATTCATCAGGTTGGCAGGGAAGGGCCTGAAACTAATTTATATATTGAATTGACTTACTATAACTTAT
GGATTGACTGATTAATTCACTTATAGATACTGACGAGGCTGCATTAACTCAAACTTTGATCACCCGCT
CTTGGCAAGAAGTTGAGGGAAAATCTTTCTGCAAGGAACGGAGTACTCGCCCGACCTAAGAAGGGCGA
GGAGCTTTTCGCGGCTCAACTGCTTAGGTTTTAGTTACTGAAAAGTTCGTTTCTGCCTAGTGCGACGC
TAAATCCATTTATTTCACCGTCGATTGAACCAAAAAGACGCTTTCTCTGAGTGAGTCTGCCTTTACTG
ATGCAGGCTCCCTAAATACTCTCGTTATTGTTTGCGCTAGCTGCGTTGGAACCTTCCCCTTAGGTGGG
GCATTGATCCCGGAGTGGAGGATGCCCATTACTTACCGGGTTTTCCACCCTTTTGATCCTAAGCCAAA
TCTATTAACCACAGGGACTTCTCGTGTATTATATTATATATGCCTTTTCTGAGCCTTTTTACGAGGAC
GCCCTTCCTTTTACAGCTCCCCACGCAATCTCCTCTCGTCTCGTTCTTTGCCTAGCGGGGTTCGGACT
TCTATTTATCTTCCCTCAAACGGGCAGTCATCCTGCAGCGGATAATGCACATTTATTAAGCTTTTGAC
CTTCCTTTGTAAGCTCTGGTATCTCTGGACCCCAAAGCACCTTCCCGTGCTTTATATTATATTAAATC
GATCATTAGATCCATAAACCTGGAGCTCTTATACATGTTCGCATAGATAAGGAAGTAGACTCAAGTAA
AGGTCTTACCAGATATACGGTTGAAACCCTTACAGGACTTACCGGAATAGACTTTCTCTATGCTAAGT
TGCCTCGGGCTTGGACCCGCACCTCGTGCTTGTTGGTGCGCGGATAATCCCTATTGCTTCTCTCGATA
GCTAGGGTCGTATCCCCCAGCACTTATTGCTACCTCGTATCGTACTTCTATTGCTGGGCTGATAATCT
GTCAGTGACCGAACCCAAGAAAGAGAATTGGTCAGTGATAAGCTGTGACCCTACTAACCTATAGTTGC
AGGTTGAAGCTGTTACCGAAGCAGAAGCAAAGATAAGATAGTTGGTCTAATAACATCTTTCCAAGCTA
GTTGTTCAGGCAGGTAAGGTATAAGCTTTGAACCAGACCTCGTGTCCTCGTACCTCGTGGCATGGGGA
ATACATTCTCACGTGAAACAATAAATGGAGATGCTGTTGTCTCGTGTGAACTCAACCTGGTATACACG
AAGTTGTACAAACAACATGAAAATGGTCTAACCAGATGTTGTCCAACCAAACCTGGACGAACCAAGCT
GATCACGGGAAAACCTATTCTCAATGGGAAGATATACTGTGCAAAGCTCAACCTCGTATCGTCCCACC
AATTGTCCAATAAAAACCGGTATAAACGGAACTTGTACCATAAAAGTGGTTAACCCAAACCTCTCTTG
AAACTGCTGGTCCAACCGAAACATACCCATCGCTAGCTAACGAATCATCCAAGTCATCAAACCAATCA
GTTGTCCAATAAACATTGTATAACCCGATCTATGGGACACGAACAAATGGTCTAACCCGAACCAGTGG
CACACGAACCAATAGCTAATCACGGGAAAAGGAAATAGTTCTCGTTCCTCGCCCTTCTTAGGTCGGGC
GAGTCACTTAACGTACCTCGTGTCCTCGTACCTAGTGTCATGGGGAATACATAGTCCCGTGAAACAAT
ATCATGGGAAAGATAAGAAACAGACAAAGTCTCACGGGAAACCATTAGCAAGAGCTCGCGAACCCTGC
AGAGCCAGGTGAAGCCTAAGTAGAATAGAAAACTTGTTTGTAAAAAGCGATAGCCTTTCGCGGCATGG
CCACCGGTAAACCTTTATGTCCTGCGGAGCGAGCCCCCCGACCCTTGAGTAGCAGGGTTGGTAAGTAT
GGGAAGGTAGGTAAGTGACTATCCTGTACAGTGTTCTGGCTGTATCCCCATTAGGTATCTGAGGTAAG
AATGTCATTATCATTCCCCTTTAGTAAAGCTTTGTTTGGGCGGGTAATAGAGGGAAGAGTATTTTGCA
TTCCAATCGGTAAGAGAGAGAGAAGATCCCTAGGATGGCACCCTTTCACTATCTCTTCACCCTTAAGCAT
CTTAGCATGCCACCCTTGTATGATCTCTTGACCCTTCCAACCGGTAAGGGAGATAGAGGAGCCCGGCC
TCCGGAGGAGACACATTTCTCGCCCTAACTATCTAACGCTTCCTGCGGTATAAGTAAAAGTAAGTGTT
TTATGACATGTTCGATAAGGCATTTAGGGATTGCCACTCAAAGTGAATGAAATAATATTCCTAGCTCA
TAATAAGAGAAGATATGTAGACTGTAGCTTGGCGGTGGGTCTTGCTAAAGTATCTTACATCTATCAAA

TTCCCAAGAGGGATAATTTGCTTGGCGGTAGGTCTTGCTCAAGGCTCTGGCTTGTCTGATTGTTTAAA
AGGAGGGGAAAGCTCCGCAGGACAAATCATGGTAAGGTGTACCCCTTTTGCTCATAACTTGAAAGACT
AGAGATGTACATTAGGCTCTGCCCTGGGGGATCAATACCACAATAGAACTAGATCGCTTAAGTAGGAA
AAGGAGGAGTCGGTCTAGTGAGTTCAAATAGCATATACTTGTAAAGGATAAGAAACTTATCCTCGATG
ACGCATAGGATCTGCTCGGGCTAACCACCTTGAAACTAAAAAGGATAGTGAGGGAAGGAAAGGAGGGC
GAGGTTCCGGCACTAGTAAGGGAAGGTACGAGGCAATATGCAGGGAAGGTTAGGATAGAACTAGTAAG
TATGTCTCCTCAAAAAGGTAAGTAGGGGCAAGGAGAGAGAGTACCTTAGCAGCCTATGTCTAATGATC
AATAGCACTTTCCTGGGAACTAAATACGACTTCTTCGAACTAAAGCATCTTCCTGGAAGTGAGATAAG
TGCCCTATAAAGTAAAGAGAGGGAGACTAGAGCTTGGCGGTAGGTCTTGCTGAAGAATCTTTCTCAAG
GGGAAGTATCGGGGGGGATTCTGTGGAGCTACGGGAGCCCCCCCGAGGAGAGTCCAGTGGGTCTCTTT
AATTGCGGTCTAATTACTGAATCTATGCAGTCACTATTATTCGTGGGCTGTTGCCCCGGGTGTGTTGG
TGGATAGGGCTTAGGGTGCTATACGAACTAGGGATGGGAGCGTGCTTTATAGATAGGGGAGAAAGCGT
ATAAGGAGGAAAGCTAGGCAGATGAGATCTTGACAATTATGCTCATTATACTGCTCCTGGTTCCAATT
AAGCTGGAACTGCT
>Repeat_13
AAGCTAGGGTTGGCTCCTCTCAGTTCTCGTCCATCATCCACAAGTAGTGGATCAAAAGCAGAGCTATC
TCCCCCTCTCTCTCCTTCATCCCAATTGCACCGGACGAACTGGTCCTCACCTCACCACACATACCCCT
ATAAAAGCAAAATGAATTTTTATGTGACCCGATAGATAGATCAATATGGTAAAAGAAACTCTTATTAG
TTAGACCGGATATATCCGAGTCACTCTCGCTGTCTGACCCGCCGATGGGACTATCAAAAGAGAAAGGC
AACTTTATCCCCAGGATGGGTTTAATCTAGTATCCCCTGTACCATTGATAAACCGGGACTATCTTCAC
CAGATGTTGACCCAATTAATAGTGTTCGAGACCCATACCTTCGTGATCCATAGCCCATAGAAGCACCA
GTGACAGGTCCAGATCCAGTCGAAGAAGCAGCGGCCCAGGCTGAAGAAGAAGCGATCCGGCAGGATGA
TGAGCAAGCTGACTTAATAAAGTTCTCCTAAGTTCAGGCTTTCGAGAGCAGAGGGCAGGCAGTCAATG
GAAGAGTACTCATGGAAAGAAAGCAGCAATCTTTCAAAGAGTCATGGAAGAGCTCCTCAGATGGTCAG
TCCCTCTTGCATTCAGTCAGTTCCACTTCCCTCTTTCGCGAATATGGAGAATATGACCTGTTTTGTGT
CCATCCCAAGATCCAACCACCTACCCAAGCACAGGAATGTGTGGAATGAAAGGGACTTGAACTGATCT
ACAGTCATTCATTTATACAGGCACTGATACATTCATTCCGGGCTCGAGTGACTGGCTTTCCTTCCTCT
AAAGCCTCCATTTCCATCAAACGGTCTGGATCAGTCAACCTCGCTCAAGTGCTTTCCTCCATGTTGGA
AACTAATCTCTTTAAGGGTACCACAACCATGCAAAAGCCAAAATCTATCACCAATTAGTGGCTACGGC
TCCTTTGTCAGTCCAACCAACACTCGCAGTTCAGCTTCTCCGGCCGTTTTCAAGCTCCCCCCCTCGAG
AACCATTGCAGTTCGGCGCTTTTCCACATCTTCAAAGGCTCCTGCTGGGCTTGGGCACACGTCTCCAT
TCACTTCACTGGGTATCCATTGCTGTTTGTGACGACACTAATCCCTCCCTAGTAAAGAGCTCCCGCGA
ATTCGGGTGTTTACAGAGGGGGGCAGTAGCGATCAATAACCAACAAGGAGGCGAGCGATAATGCGGAA
AACTGTGCCTTCCATTCCAGGGTTGTTTCGTTCATCTCGTAGTAAGTTTGAAACGGGTGTCTTTTACC
CTCTCCCTTAACATGTCCTGGGCGAATAAGTGAATCCACCTAACCCACTGTATGAATCGTATATAACC
AAGCTGTTGTAAACCTAGCACTATCGATCCACCACCTCACACGGCTAATAATCCGTGCCGGCCCGCCT
AACCTCCACGATGTGATCCCTCCTAAACAATATTCTAATCCAAGCGGATGGGTCTTGAGGATTGTTTC
CTGAAAGCGTAATGGGGTCTGGAAGGGTATGCTTTAAATGCGGATTGAATGGAGCATGGGCAGGGTAA
GTTCAATAGGAAAGTTATAAGTAGTAACTGTGTTCCGTACCGTTAGTTTAAGGGGGAAGGTGGGCAGT
GATTCGTCCCCGTCCGTTGGCCCTGCCTAACCTTACAATATGTTTTTTGTTGTCCGTCCGTGGTTGGT
ACCCGCCTATCAATTGGTGAGGAAGTATCCTGTTTCCGGTGGTAACGACTATTGCGTAAGATATTTCA
TTTTATCAGCTTGCATTATATATGCTATTATCCAACAATGGCAATGCTGCTGGTTATTTCTTTCTGAA
TCAATCGTATCTAGAAGCTCGTTCCCCCGATAAGAATCTAAATAGTGCAACCCTTTATTTCATTCCTC
AACCTCAAGCAAGTGGTTGTTAAGGAATTCATCCATTCACAAAGGTTGGGTGAAGCACAGTAAAGTAA
GTAAAGGATGGAAAAATGTGGATAGCATGCACTTCAGTAAAGGTATTCATAGTAAAGATTCTATTCTA
AGTGCTGTACCCACTAAACCCACTAAGCATTATTGCCTGAAGCAAGGTATGGAATGGCATTCATTAAT
TCAATAACAAAGGTAGGGTAAGGGGAAAAGAAGGGTCCCTTTAAATGGTAAAAGTAAAGTAAAAATAA
AGGTAGTAGGCTAAAGGGTGAATAGATTAGTGCCGTACTTCCTTTTCCTGGCTGAAGCAAGTAGTTGG
TAAGGGGAGGCAACACTAAACAAATAGGATGAGGGACGAAGTCAAGTAAAGAGAAAAGCTGGGTGCTC
GGGTGGTAAGACTAAAAGTAAAGGCAAGTTGGGTAAAGGTAATAGCAAAGGTGTGCCCAAACCCTTTC
AATCTCCGTTGGGAGTAGTTCCTTCCTCAATTAAAGAAGGCAGTTCCAAGCTTCTATATGAGATTCAT
TATGAGGGACAGAAGGAAACGAAGGGCTAGAGATCATGATGAAAGGAAAGGGCTCATGATTAAAAGGC
TTGAGAGAAGGATAGAAAAAGTGATAGTGCGCTGTATGTGATACTATCTGTAAGCTGGCTGGCTGGGT
TCAGCCCGACCCAACAAGATCATAGTGCCTACCTCCCTAAGTCTTAATGACGTAAGTGGTTAGCAAGG
GCATGCAACACTCCTTCACTAAGTGGGTTGAGACAGTTCACAAGGTGGTTAACACGTGGATGCACATA
AGTAAAGCAGGGTAGGCTGAGGAAAGTAAATAGTCAAGGGTCTCCTAGCAACTAAAGATTAATACAGT
CCAGTTTAGATACAGGAAGACTACACATAGAGCACAGGCTTGGGGACAACAACCATGCCATGATGGGC

ACTTATGTTTAGTTTTGGTTGATCAAACCATGATGCCTTGAAGCTGGACGAGAGGCACCTACCCCGGG
GAAATGGAATTTCATTTACCATAGGAATCTTATCCCTTTTTATTTGTTCCGGGAGGCAATCGGACGAG
TAACGAAGTGTTTTTATGCTTTTGAGACAACCCGAATTTGACTCCTGACTAGACCGCCTTATTCTGGG
CACTCAGTAGAACCAACAGCCCAGTGGAACCTGATACAGCTTAGCTTCTCAAGCCCCCCACAGTTCTC
GAATCAATCAGTTCTCGTCACACCCAGTGGAACCTGATGGATGCTGCAGTTCACTGAAAGCCCCAGTT
TCAATAAATGCCCACAGTGAGTGGAACCTGATGCTGCTTAAGCTGGCACTAAATCACCTTCCCGGTAC
CTCCCCGCATGAATGGCTCAACCGGACCTCCCCTCTTTCCTTTTCCTCTCAATCTAAGGCCACACCCG
AGCATTCAAGAGATTAACCCCGCAGTTCCATTGCCTACACTCTTGCTAGCTTTGGCTTTGCCCCTACA
CCTGAAGGATATGAAATTGGAATTGCTCGCGCTGAGGGTGGGTCTTGATATCGATTTGGTTAAACCCC
TTTTTCTGTTTAAAACGCTGGTGGTGAATCAACGGACTATGTTGCAATAGATCTCTTTGCCGGGAGAG
AAGAGCGACGCCGGACCCTCCATGTGGTCGGTTCAGTTCGAGGTCATGAAAGGTAAAGTCCAGTGACT
GGTTCATAAAGGGAAGATACTAATGCAATAGCCTGCATAAGCAACTTTCTCCTTCAGGTGTAGGGGCC
ATCTTCTCTGCAGCTTTTGTATTTGCTCTAGGTGCTCTAGGCTATGCTTATACTGGTGCTTCTAGCTC
TGCTACTGATAGCTTAGATGCTGGAACAACTGGTACTGCCTCCGACGCTTCTGCCCTAGCTTATGGCT
TTGCCTCTGTCCCAACAAGCTATGTCTTTGCCTTTGCTACTGATGGTGCTACTGGATATGTTACTAGG
TATGCTGCTGTAGGCTATGCTACTGATGCTTAACCCATAACCATATCCAGTAGTTGTATTACCAGCAG
TTGAACCAATTGATTTCATTGACCGCCCAGTGGAGGTAGTAGCAAAGTCAGTGGTTTCACCCGATCCG
GAACCAATAGTTGATTCGGGACCAGCATTCTCGCCTGTTGGAGAAGTTTGTAATCCAGTTTTAGATCC
TCCAGTCCCGAATACAGATCCAAATACATATGAAGATATAGTTGGATACCCATCAGCAGCAGTGGGGG
TAGGAGCAAACACAGCAGTGGTGGAGGAAGCTAGAGATCTTTGTATGGGTCCAAAGAAAGCATAGGCA
TCATAGGCGGGGGCACTACCAGTGGTTGAAGACCAAGTTGCCAGGGCCCGGAGCAGTTGTTGATCCCA
TTGATCCAGAATCATTTGACCGAGTAAAAGAGACATGGACCATTGGTTGCAGAGCCAATTTAGAGAGC
CAGCATCAGTAGCAGTAAGAGCGTCGACGGGGTAACTAGCAGGGGCGGGTTCACCGGTTGTTGTTGAC
CAAGTTGAACCAATTGAAGCGGAACCCCTTCATTTATTTATCAATTAAACGGGGCAAAACCAAAGGCA
GGATCAGATGCAGCTACGAAGCCCCCAGTTGAACCTATTAGCCAGTCAACCAAGTTGTTTACCTCGCC
CTGATACAGAACGAACAGCAGGAACAAAGGCATAAGTGGTAAAGCCTTACCCTCCGGTCCTCCCATTC
GATCGAACAGCACGGGTGTTGCTGAAGCGACTCATGAATCTATAGTTGTATCCTCTGCCCACGTCACT
GAAAGCCGAGCATGCCCATTCTCCTAGTGCCCGAACCCGCTGACTGAGCTATGACTCACTGAACTATG
AACATGGTAGCGATCCAACGTTGCCACGTCCCCGAACCCCAGCATTTTGAAAGGCATCGAGTGTCCCT
TTTCAATAAATAGGAGGGGTGGATGGTACTTCGAAAGGGATCAATGCTGGCGAGGTTGGCTTCAAGGC
TTGGGATATTGGGCATAAGGCGAGGAGCCAGAGGCGAAGCGAACAAAGGGAGCTCCTTTTATGTATAG
AATTTGCCGAGCAATTTTATGTATATGATTGTGAGCGATATTGGCAATAAACCAAAGAAAGGGATCTT
ACTGAAGGGCGAGGTGCCGCAGGTTCTTCGCATGTTCTCATTGGGATACAGGCCATACATAGAATGAC
ATCATCGGGCCTCTCATCGTAATTTGGGTGGACAACCCTAACCATTCAGTTAGAGTGGACAACCCTTC
CTTTCCCATTTATCGCACCAATACTTCTATTACGTATAGAATACTTTGATTATTCGCTACTCTCTCTG
CTAATAATTTGAACTTGACCAGATAACTTGGGTGATTCGCTACTCTCTCTGCTAATAATTTGAACTTC
CACCTATTGCTATCAAGCTTTCGTTTGGAGCAAACTAATTCATTCGAGCAAAGTTCGTTCGAATTCAT
ATATTTTTTTTTATAAGCTTAACCCGTTCTTCAGCGCCTCAGCTTCACCCCTATATCGGTGTGCATAA
AACATCATGGTAATAGATAAGAATGGTAATAGATTCAATTAAGTGTTCGCCCCGAGATGATAGCATAG
TTGACACCATCAAAAGCTACTGCGCGCTGGAGCTATAGCCTTGTACATAGTGAATTCCCGCTAGAATG
GAGATGTTGGTTGAGGAAATGGCATGTGGGTACGAATGGCATATATGGGTACTTAGAAGGCTTGGTGG
TGGTATCCAAATAATATATCATATACGTTATTTCTAATCATGGAGAGAGCCATGGGCACATGCGGACA
GAACCCTCGGCGTATTAGCGACTCCCCAATACGAAAGCCCATTATTGAATCAAAAACCAGGAAGTGCC
GCTCATCGATTGATACAGCTTTTTAGGAAACTAGCTTTATACAACCAACTTTGTACAACCAAGCCGTG
CTTTCGAATCAATTGACGGGAATTAGATAGATAAGGATCAGAGGTTGTATCCTCTGCAAGGATTCCGA
CTAGATAGGGATGCTTGGGGAGACTAGCATTATACAACCAACTTTATACAATTACCGCAAGATTCCTT
TCGAAGTACTATCCAGCTCTAGCCCACACATTTTACCTGACATCCAAGAGACTACCAATGCTCGGTAG
TTCAGGAGGTCGAACTCGAGTTCGGCACTTTGGGGAGCCAAAAAAAAAGATGGAGTCGTTCGCCCATC
TCATCTGTTGGTGGGAGCGTGAAAGCACATCCGTTGGTGAGATACCCTAGTTCCCCAATTATCCTGCA
CAGGAAACTCCTAGGAGCATTTGCCAGTCCCGGGGAACAGACATTTAATTTTCGATACTGGATTTTCG
ATAAGCCTCTCCCCACTACAGCATTTTGGAATTGGAATACCCAGGATCGTAAGTAAGGGCCGGTCCAC
GCAAGATAAGGCACTTTGGTTCGTACGGGTCGAATCCACACGGCACGCACGGTTCGGAAGTACAGGTT
TGACACTGTTCCACTATCGGGAGATCGGAGTATGGGAAATTCCGAATTGGGGGAACATGGGGACTAAG
GGACAGGGGCTTTCTTTCGTTCGTTTCATTCGCTACAGTCGTATTAAACATCTGTATCTTGGTGGTAA
TAGGGAGTGGTTCGATTCCCTTATAGGTGGCGGAACGGGAAAACACGAGCTTGATATCAACATGGGTG
ACGAAACCGGCGATTGATATTCATATTTATACTCACCCATTTATGAATGAGAGCGGGCGGTTGTTCAA
TCCAGGCGGGGCTATCGATTCAGAACTAGCCACTTTCGGGCCCTACCTATATTCGTAGGCCCCGGTCG

GTACGTCGAAAGGGATCAAGGCGATTCAAGGCGAGGGGCGGGGTGCCGCAGGACGAGCTTATTAGAAG
GGGGCGAGCGATGATGCCCAACCAGTATTGAGCTAGCTAGTGGTTGGCCGGCCCCTTTCGCTTCTGCT
TGAGGAGGTAGATTTATTTATGCAGCATTATCCTGGAATTTTCGAGAGCGTTCCCAATCCGATAGCTA
AGGGGTTGTCGCCAACCCTTGATTCCTCGCTCTAGTTACTACATGGAATGGAATAAATTGTAGTGGTT
GAACTGGAACATTCCAAGTGACTAACTCCCCGATGACTCCAAAACTCAAGTTTCTAGTTAACAAATCA
GTTGGTTGAAGGGTCCCATCCACTCACATGAAAATCAACTGACCATGAGAAAGGTTACAAGTAAAACA
TCCAGGGATAAATACCTTAAGGGGATATTCTTATCACTCAGGCATGGTCAATAGATCAAATCATCCAT
TAAAACAAGCATCATCCCCACATGCTTGTGAAGAGCCAATGTTTCCCCCATCCTCCAGTTGCTAAAGC
AATACTAAAATGGTAAAGGATTAGGAAAGGACAATTCCACTAATTTTGATAACATTATAAAATGAAAA
AAATATAAGATTGATGAACGAGGGTGTATCTAGGATCCTTGCTTGGCTTCTTTCTCCATCCATCTAGT
ATTTTACCCCCTCGTCTATGTTCCCTTAAGAAAGTATCAGCCTCTTTGGGGTCATAGATTCTTCCCCC
AATCGACAAACAAAACATATAATTCAAAGCCACAACATCGTTCGTAAATCTTCCAATCAAACAAAGAG
AATTAGTTTTTCGTTATACCTTTGACTTATTACCTGTGACTTTGACTGTTATCCTAAGTCACTTAACA
ACGTCCTTAACGTCACTTAACAACGTTCTTAACGTCCTTTAAGTCCCAAACATTTCATTCCACACACC
TTCAGTTATGGTGGCTCCGCCCAGTACTTACCACAAACGTGCCTATGAATGGCTCCGCCCACTCCTTA
CCGCGATTGAAACAGCTCTGAGGAACAACGAGGAATTAAGCAGTTGGAGAATAGAAAGTAAAGATGCG
GTCTTCCCGGCCCACCCACGCTTCTTTCGCAGAGGTAGTCGAGGGGGGCTCTCTGTGGAGCGATAAGC
GAACCCCCCGATACGGAGCTGGGTTAGGATCACACGACTGATCGGGATCACACGACTGACCTTTTCCT
TCGATTGAACTTCTTTCTTCGATTGAAGAGAGAAAGAGTTTCCTTCCGAGAGATAAGCCGAGTTGGTT
ATCCCTTACCTGATACTTTTCATTGAGGGAAGAAGCTGACTAATTAGCTCATAAATAAACTCTCAGGT
AATAGCTTAACAAGCTTTGAACACGAACTATCACGCGAACTATTAAGCTATTTGGTAGAGTGACATTC
CTCAAACGTAACATTACGAAATGGGGAAGGACATGAACATAAAATGGCAGGTCATGAACATCAAGGAA
CGAACATCAAGTGGCATAAACTAAAAGTCACACAGTAGGTCGTTAGCACGAATGAATGTATATGCAAG
TGCTATTCCTTTCCTTACCGCGTGACATGCCTTACCACCTGAATGGCTCACTGACTGATGAATGGCTC
ACCTGACCGATGGTTGAATGGTTCACCTGACTGCGTGACATTCCTTGCCGTAGGCGTCTATACGTCCC
TGTGCTGGGCTCACCCACGAACTCTTCAGTGGGGCACGAACTATTAAGTTTCCTTCCTGCCACGAATT
CATTCCTGTGCTGGGCTCACCTACGAACTCTTCAGTGCGGCTCTTCAGTGCGGCACACAAACTCTTCA
GTGGGGCACGAACTACTCTTCCTTAACACAAATTATTCGATTGTCATACTTAGTGATGTCTCTAATCT
TCTTTACGGAAAAGGATCCTTCTCTCCCTTTTTCATCTGGTTTGCCGGAACAATTGTGCCAGACCCGA
ACTAACAAGCGGGCTGTCAATTGCTCAATCTGAATCACTGGCTCTATGGGAGACCATAGACTCGGGTA
AGCCTTTCGTGGGTAGTCGGCTCGGAACACCAGATAGGTGATAGCTGCAGGTCCTTTACTCATTGCCC
GAGACTTCGTGAGACTATTAAGGGCATTGAAGGTCCTCTCTTGATGTTATGCAAGTGTGGTACTTAGC
GAAGAGAATCGAGCTGCTCGGAGTTAAGTGAGGAGTAGGACCAAAAGGGATATCCCTTGCAAAGCCAG
GGTTTATTAACCTCCGACTGGCTTTGTTTTTGATACGCCACCTAATGTTAACTATGCAACGTCTATAT
GAAATCATAAAAATAAGAAGGGACTTCTTCTTATCCTTAAACGTACAGGTTCCCGTCTCCGCTTGTAC
GGTCTGGCTTGTAAGTCATAGCTTGTATAGACACTGGAATGACTATAGACACTAGACACTTTCCTGTG
CTTGGGTTGGATAGAGAGAGTAATGGCTTCCCGATTGGATTAAGCCTCCCGATTGAGAGAGTAATTAC
TTCCCGAAGAGTAATGAATGGCTGTAGATAGGAGAGGGCAGGGCTTTCAACTGGTAATAAACTATCCA
TTAAGTAGGCAAGATTGAGGGTAGGAAATAGTAGGCTTGAGGGTAGGGCTTTAAATGAGGGTAGGCGT
GCGTATAAAGGGTAGGCTTTTAAAGTGAGGGTAGGCAATAGCAATAGTAGGCTTGAGGTTGGAATGAA
TGTATACTGCTATACGCTGGCCGGTTCAAAGAGCATCCACATCTTTCGTCGTTTTTATAATAGGGAAG
GGAAGAAGAGTTCTTCTCGATCTCGCCCTTACCTTAGATTAGTTAAGAGAAGACGGGGACATCACCTT
GAGTTATTCTCGATCTTTCCTATCGATGGAGTAGGAGTAGAGATTTATTCCAAGAGTTCTTCTTGATA
GATTTTCCCCTCAATGCGTATGAAAGGTTATCCCTTTCCTGGCCTTCCTCCTGCCAAAGAGATGGGAC
CAACCTCGATTGGGTACGCATGGGTTTGGGTCCGCGACCAGAGAAATGACCACTTGAACGCGCTGTAT
CACCACGAAAATCCTTTTCAGTTACGTTAGGGGCATGGGCTTTCATAAAGGTCATCTTTCGTCGTTTT
CATTAGGCAAGTAGTAGTGTCTTACTGGTATGATTGGGGAATACCGATACATGACCTACGGACTAATA
ACCTATGGCGGGCCCGTTCTGGCCATGGCGGGTAAGATGGCGGGTAAGCATGTCTATTATATTATAGG
CGTGGGTACATATAAAGCGGTGCAAGTATCAATACCCGTAAACGGGGTGGGTATATTCTAGCCCCGCA
GGGCAAAGCAAGTAGGTTCGGCTTACTCCTTCCACTCAATAGATTTTACAAGCTCTCCTAACTATTGG
ATAGGCCATTAAGCCTGGTGGGACCAATGGGATGGTTAAGGCCCGCACTATTGGGTAGGGGTGGGACC
AACCCTTACCAAGGTCTGGTTATCTTGGGTAAGGCAAAGGTCTTGGGTAAGGCCAAGTAGGAGTAGAA
GTAGGCTTAGTGGGACCGCTAAAGGGAGGGCGATAGAAGGTAGGCGTGGTAGGAGTTATCGATTTTTT
CGTGTTCTACGAAATCATTTTCATTCTATCTTTGAACAGACAAACACACTGGTCCAACCTAGCCTCTA
TCATTGAGCTACCGGTGAACACCAATCTATTCAATCTCGACCAATCCCGACCTGGTGAGGTGTGGTTC
CTATGCTACCTATGCCCTATCTATAGTGGGCTATCGGTGCTTACCACTCATTCTCTTTCTCAACAGAT
CCAACCCTCGTTTAGGTGCGGGTGCATACTTAATAGAGGGGAAAGTCCATCTCTACAGCCCTAGCTCA

TGGAGTACCGGTGCTCACCAAGCATTCTATCTATCCACAGCGGCTAGTCCCATCCCATCCGAGGTGGA
TCCCATCTCTACCAATCGAGACAGACTTCTTCGAGGTGGAGCAGCTGGTCAATACCGCAATTACAGGG
TGGAGCAAAAGGATAAAGATAGATTAAGATTCGTTTAAGTGGAATTTCCCTCTCCCTTATTTAGAGCC
TGCTTGCGCTTCCTTGCCCTCCTATCGTCGAGCAAGCAATAAATTCCTCTTTCACCCATTTTGTTAAG
AAGGGGAGGACCTTATCGTGCCGCCCCTCCATTCATAGATTGACCCGCTCCTAGGTTTGTACTCTTAG
AGGTTATGCCCACCAGAGGAGAGGCCTTTAGGTTCGACCTTATAAGTTATATCCGCTTAAATGGTGGC
TCCATTTTAAGGCATTTAATTGCCACTTTCACCCAAGGAATTAAGAATGACCTTACTGGGTGGATTTA
CGTATATTATATATCTGTTCAATATTCCCTAAGAGCTAAAGGATAGATACTCTTTTCCTACCTTATAG
ATTCCGCAGAGGTGTTCTCCTCGATACGGTAGCGAGGAGACTATTCACTTCGAGCGGTAGTGAGAATA
CGATTCACTCGAGGAGCTGTCAATAACGGGTTCTGTCAGAAGTACAAAAACGTTTCAAATGGCTATTT
GATTTCTAAAAAATTCCACTTTCATCCAAGGAAGGTATCAAGACCTTACTGGGTGGGTTTACCATAAA
CTTTCTGTACGTATCGGCATCAAAAGAACTAAGTATTACGTGAGCCAAAGGGAGAGAGGAGATGTAGC
TACAAGTTTCCATTACACGCCTAATTGTACAAGGAAAGAAATCCATTCATTGCTGCTCCATCCGAGAA
TCTAAGGCCAGCTGCTCCACCCGAGAATCTACGGTCGCCATCCGGTCTTTATCCTGCTAGTGAGCGGG
CTTACTCCTGCTGCGGGCTTACTCCTGCTAGCAAGCGGTTGTGGGCCCCACAACTTAGTGGATTGCTT
ATCCATTCTATTGACCAGCTGCTCCACCCATTCCTTGGTTCTGTCTCCCTGTCTCCCAGGACGAGTTA
CCCAGTCTCCCAGTCACCCAGTCAAGGAAGCCAGTCACCCATCCCGGTTGTTGCTCTCATGCCCGAAC
CCACTGATAAGACCTTTCCTTGGTTCGGGACCTTGGAGTACTTGCCTACATTGGAGTACTTGCCTACA
AGGAAGGTATTCGGTCAGCTAAGGTCTTCGATAAGGGCGAGGGTCTTCGATAAGGGTCTTCGTTAAGT
GTGACGAGGAAATAGCCTTCGAGTAAGAGTGCTTGCCTACAATTGAATGGCTTATCGGAGTTACGAAG
TTAGTGGACTTAGTTACGAAGTTAGCGGATAAACTAGTCCAAGCTTGCGGACTATAGCGAAAATATCT
GCTGCTGCAGAGAGAAAAGTACTTACCCACCTACTTACCCACCTACTTACCCACCTTAGAGAGAAAAG
TACTTACCCGTGCTCTTGGGTCACTGAATAGTACATATCTAGGTCTTACCCCAACCCAATAAAAAAAA
CTATATATTTATCAGCTCTTAATAACTCTTTCCTCTCTTTTCATTATCTTGGGTCAGAAAGAGAAAGA
TACTGGGGCATTGAGTCAGACACCCTATAGTAGTAAGGACTACTCCCTCGTGTATACAGGGTGCGTCG
TACAGGTGCCCCCCTCGTGTCTATGCCCTCTCTCGCTTTGATGCTTCGAGGGCTCGCACTCGTAGAAT
TGGGGGGCCCCTGTCCTCCTTCCCTTCTCACTCGTCCAAAGGGGAGTAGTCCTTACTCCTAAGCTACT
GTCCTCACTCTCCCTCAGGGCCATATGAGCTGTACTCATTTCTTCTCTTTAGGCACTGAACAGACTTA
CGGGACATGGACTTATAAGGTGCTATTTATTATATTTTTCTATGGCTTATACCTTCTCTTTTAGCGCC
TTCAAGTGGACTTAAAGTTGCCTTCCTTATGACTAGCAGTAAATAGATTTATTATCTGTGGACCTGTA
ATTAGTAGGAGGGCCTTTAATGAATTAATAGGAGGGCCCGTAAGGAAAAGGCTTTTCATTAATAGGAG
GGCCTTTCCTACCTATGTATTCTAATATGGATATGAAAGACAAGGAAATACATCCATTCATTACAGCC
GATAGGCACTCTTCGGGGTGTCATTCCTTCCACTATAATAGATTCATTGATATTAAATAAGTTGCCTT
CCTTAGGAGGGCCTGCCTACTAATATATTCTGCCTGCCTACTAAGATCTGGAGACCAACGCCACATTC
CGATTCAGGACCTTCGGCATACATACGTGGATTTATTATCTTATCTTTACGGCATACCCACATAGATA
TTAAATAAGTTGCCTTTTGAGTAGGAGGGCCCGTAATATGACTAGCTGGTCGTAACTAGGTTGCGTTT
CTGTGGTAGTCGTGTCGTGGTTGCCTTCCTTATTACTAGCAGGAAATAGATTGATTTATGACTTATAA
TCTGACTTACCTCGCTACCCTAAATTGTAATTCCTCGCTACCCTAAATATGACTTACCACCCGAGAAT
ATAATAACTTTAGTTGTCTCCTTCTTATCCGATCTGTTGGGCGGATCCCAGCGGTTATCGGAGGGGGC
TCCGCCCCCCCGAACCCCCCAAGTTACCGGCATGAGCATGAATAAAATAGTTTCGGGCTAAAGGCTGT
TATCGGGCTAAAGTAGGTTATCGGACTAAAGGCATGGTTTAGAGGGCTAGCGGTTATCGGGCTAAAGG
TCGGGGCTATATGGCTGATACCTTATAAAGGGGTAGTCTTAACCGGGGTAGTCTTATGAAGACCTTTC
CTTACCGGTGAAGACCTTTCTTTACTAACTGGGGAAGACCTTACTAACCGGTGAAGACCTTAATGCCT
TAATTCCTTCCTTATGACTTTCCACCAGAGAATATAAGGAAGAAGAAAGAGATAGATTGGGATAAGGA
AAGATGGGTAGAGAAGATGGGCCATTAAAGGTAGACCAGTAAAGCGACTTCACTAAGGTCTGGTGGGG
AAAGTCCGGTGGGGAAGGACTAGTGGAAGAGATAGATGGGTGGAAGAGAAGGTCTGGTGGGGATTGAG
GGAAGAAAAGAGTATCCTGAAAGGTAAACCCTTAGCTCTGCGTGCGAAGGGTTAACCTTACCTTCTAG
TTAGCTCTTTGAGGGGAAGAAATCAAGGGCCAGGACCTTACCCGCCCGAGGGGTGGGGAAAAGGCATT
CTATAGCTACAATAAGTTAACCCAAGGTCCAGAATTTGTGCAACTTCAATAAGGCCAGAATTTGTGCA
ACTACTGCTTGCTATAGTGAATTTGCCGTAATTCACAAAGCTAGACTATAGTGACAGAATTTGCACAA
ATTCTGAACCTCGGCGTGCGTGGAGCTTTCAAGATGAGGAAAGCCCCCCCCTTTGAATAGATAGGTTT
CGATCACGGGGCGGAGCAAGTCACTTTACCCCCCCCCTTCCCCATTTGCGGTGTTTCACCTTTACCTC
TCTTCGCTACACTTACATGGAATACTGTTTCGCTGCATGCATCTCTGCCCGGTATTGGTATCTCGGAA
TGGAGATCTCGGCTAAGGAAAAATCTCCAGACCCCACACATCATGATGATTAGAATAATAACAGTAAG
AACCCACATTTGGAAAAGTTCTGTTAGGTTCTTAGTAGCAGCCGGCGACCTCCGTTTTTTCTTCTGCT
TTACATAGCTTCTCACAAGGTCTCCTTGATAGCTGGAAGTTCTTCAGGAGTATGAAGAGCTGGAGGAC
TTTGTACCATCCATTCCGGTGTGGTTGGATTATGCTCAACAGCCCGGGGACTTGGAGCACATCTTTTG

TTGTTTCCACCGCCTAAAGTGATTGTTACGACCACGAAGAAACGACGAATCCCAACTACGGATACATA
AGGGCCGAAACTGCTAAGGGCATTCCATCCAGCGTAAGCATCTGGATAATCTGGAATGCGACGTGGCA
TACCCGAAAGCCCCAAGAAATGCATGGGAAAGAAGGTCGAATTCACCCCGAAAAGAGTGATCCGAAGA
TGGATTTGACCTAAAGTTTCAGGGTATGTTCGACCAGGGATTTTACCCACCCGGAAGTGAGATCCTGC
AGATGAAGCAAAAACGGCTCCCATAGGAAGTACATAATGGGAATGTGCAACCACATAATGAGTATCAT
GCGGAGCAATGTCTAGCCCAGGATTTGCCAGGACTATTCCAGTGAGTCCTCCTATGGTGGACGGAAAG
ATGGACCCTGCAGCAGATAACATGGGTGTTTTGTATCGTATCGAACCTCCCCACATGGTAGCGATCCG
ACTAGAGATTTTGATTCCAGTGGGGACAGCTATGATCATGGTAGCTGCGGTAGAGTGAGCACGCGTAT
CAACGTCTGAGCCCACAGTAGACATATGATGAGCCCGAACAGGAGATCCAGGAACACCAATACTGATC
ATGGCATAAACCATGCCTGGATACCCGAATACCGGTTTTCCCGGAAAAGTCGATACGATATGACTAAT
GATACCGAATCCGGGCGGAATGGGAATATACACCTCTGGATGACCGGAGAACCGAAGGAGATGCTGGT
ATAATATCGGGTCTCCCCCTCCAGCAGGATCGGAAAAGGTTGTATTAAAGCTTCGATCGGTTGATAAC
ATGGTAATTGCCCCTGCCGGTACCGGAAGTGATGATAAGAGTAGGAATGCTGTCACTGGAACGGACCG
CACAAATAGGGGTGATCTATGCATAGTCATTCCAGGCCCGCGCATGTTGGGGATAGTAGTTATGAGAT
TGATAGAACCTGAAATGGATGAAACACCTGATAGATGAGGACTAGAAATCGCTGGATCAGCAGCTCCT
CCGGAATGACTGGTAATACCACTTAGGGGCGGATAGACCGTCCACCCAGTGCCGCTACCCACTTCTAC
CGAGGCTGGGCTTAATGGGAGCAACAGCGAAGGTGGCAACAACCGGGATGGAATATTATTCAATCGTG
GAAATGCCATGTCAGGTGCACCTATAGGAATCGGAACGGACCAATTACCAGATCCACCTATCACCGCC
GGCATAACCATAAAAGGGATCATTGGAGAAGCGTGAGCCGTTATTGACACATTATGAGGTTGATGATT
CCCACCAAGAATTTGATCGCCGGGTTGTGCTAATTCCATACGAATTGGTACTGAGAAGCATGTGCCCA
TCACTCCAGCAATGGCACCGAAGATTGAATGTGGAGTCCCTATATCCTTGTGGTTAGTGGGGAACGGC
CATCGAACCAAATTTTTCGTAGAATTCTGATTATTTCGTTTCATTCCTTATCAGAGAGGGGCCGGCCC
CGAGGAGCGGGGCGGCGGCTTATTGGGCGCGCCCCCCCCCACCAGTCAGTGTCTCTTTCTCTTAAGCG
GGTGAGGGTGGTTCTGAGAGGGGGAGATAAACTCCGGAAATAATAACCCTCACCCGGGCTAACAGCCA
GCATTTTTCCAGATCCTTCAATCCAACCAACCAATCCGGGCGGGTAAGGTCCTTCAAGGTCAACCCAG
TCAGGTCCTTATAAACCCTGCTATTTTTAACCCGCGAAGTCAACCCTCCATTGGACGGACCTTATAAT
GCCTCACTGTACCGAATACTTATTTCCTTGATCTCCTTCTGCCTACCGGGGGAGTGGCTCCGCCCCTC
GATCTCCCGTCCGACGGGAGATCGAGTGAGAAATCCCTCACCCTACTATATGGGGGTCGGGTGAGCGA
TTTCATCGATGGCGGCCCTTTCTAAGGGCGAGGTACGTTAAGTGACTCGCCCGACCTAAGAAGGGGGA
GGTACGTTAAGTGACTCGCCCGACCTAACTGACCCTCCCTCTCTTTGAACCTTGTAAGTATTTAACTC
ACAGATAGAAACTTAGTGCCGTTTGATGAGTAACTAAGAACGAGGGAGAGGGATACGGGAAGGATGAA
GTAATGGAAGAGAAAGTAATTGCTAGTAGTAACGACTTGTCACGATCCATTGGTTTATATATCATCCA
TGTTCTAGGTGTATCGGGATACATTATTTTCGCTAAGCGTATATAATAAAATTGGGTGGAAGGGTCCA
CCCCGCGACGAAGGATGGGGTACTAACTGCCCCAAGACCAAGGGGGTACTAACCATTATTTTAGCCCC
TGTCCTCTCCGAACCGCAGGAGATAGTTGCCCATCATACGGCTCACCAACTTCACTTGCTCCGAGAAT
GTTAGGTCGGGGGGGCTCGGGCGGGTTCACAAACGAAGGTCCTCCTGAGCTAGGCTAGTAAGGTCCTG
ATCTTACGCATAGCGAAGCTAACTCAGGCTTTGAGGGGAAGAAATATAAGTGAAGTATGGACTCTATT
TATTCCCCTCAAAGAGCATAGCGTAAGTAGCGGAGCCCATGGTTGGTTTGTTTGGTGAAGAGGCAAGG
GGAAGGGATGGGAAGGAAGGGGCGGGATAAGCCGAATTTTGGCAAATCGATCTATTTGTGTAGGAAAA
GCTTATTTTGCCGTCTCCCTTTTGTCCCCTTTGAGGCTTTTTAGGTTATCCCAGAATGCACTATAGAT
AGATCGTAGTTGCTGAGCTATAAGGTCATCACTTACCTTCCTTCTAGCGGGATAACCTTCCCCTCGCT
ACCGGTCTTACCTGAGCATAGCGAAGGCCTTGAGCTCTTTCTTAAGTGCTTCCCTCCACACCTTTACT
TACCTGCCTGGAAGGGCCTTACCTGAGCGAGTTAGTTCGGGAAGATAAAACCCTTTCTTTTGACCCGG
TCCTCTCAGGCGGTCGGTAAGGTCTTACCGGGTGGGTTGGTCCTTCCTGAAAGCCCTTGATTTCTTCC
CCTCAAAGAGCGCTTATAAAGTGACTCTTTCTTCAAAAGTCCATTTCTTCCAGTCATTCTTTATATAT
GTAATAACACTAGATTCGTGATTTTATGCGATCCGAAAGCGTGAAACTAATGCCCTTTCGCTTCAAAT
GCAGTTCTTCCTCACCTTCATACTTCAGGTGAGCCAATTCTTCCTAAAGTTTCTACTTCAGCAATTAT
TCCAGTCATTCTTTATATATGTAATAACACTCGATTCGATTCGATGTGAGTCGAAGGTACGGAGTCGC
TCGATTGACATGGGAGATCGAGGGGTGAGGGTACGAACCGCTCGCCCGACAGCAGGAGGGAAAGGAAT
GTGAAGTGAATCACCCTAACTTGACGTTCGTGAAACACTTCGTTTCAAGTCATAAGGCAAGCGTAATT
ACATCTATAATGAGATATATCTTTGTATACGATATTATTCGATCATGTTTTCGTTATGATTCGGGTTC
AGAAGACTCGCAAAGCAAAGGAAGTAAGTGCCTTACCTGTTAGGGGCAGTAGTAAACAGACAGTAATA
AAGGCAGTGCGGTAAGTAAGATCGGGTAGTTAACAGTAGGCGTGGCGGGTTGGCGAATAGAATATGAA
TATCTGGATGGAATAATCGCCTTACGATACACCAAATACGAAATAATTACAAGAAAGATAAGGAGAAG
AGTTTCCCACCTTCAGACAGATAAGGAGAAGTAACCCACCTTAACCTTTAGATAAGTAAAAGTACCCC
ACCTTAACCTTAACGAAATAATCACCAAATAACTCACCTACCTTGCAAGGGTTTTTACTTTTCATTCA
TTTAGGTAGGGGCTTGCCATCAAAGAAATATAGGCATCTTAATTGGACTTTTCATAGGCGTTAGAAGA

TGCGAAACCTTGAAACTCAGGTATAACCGGATACTTTGCCATGCCGTATGTCATCCTATTTTCAGGTG
CCCTCAATGCTATGCCTGCTATCTTTCTTTTTTTCCTTGACTGGTCCGAAGCTCGCAGGACCTTGAGC
CAATAAACGGATAGATGGTCGATTATTTGTAGTTACCCTTGCTCCCCCGATTGCTGGATGGCACGGAT
TAGAATTACCCCATACTGCTGCAGTTGGACAGGTTGGATCGTCATGCCTTAGTAATTAATCCCAGAAC
CTTGAGCAGAGATTGTTTCACTGCTGGATAGATTCTCAACTGCCAGAATTTCCGAGACATGACTTCAG
AAACATAGCTGGATGGTCTCGAATAGCCGAGGACTTGTAGTAACATGCATGCTCCTGCGGGAGGATGG
TCAGCCAATAAGGATTGACTTTTAGCTTATATCGTTGAACTGAATCTTCCGGATAGCTCAATTATGGG
CCATCAGAAAGCCTCGTTGGGATCGCATGAGGATTCACCCAACCCTAGAAGTTTTTAACACCTCAGGT
TCAGGGACATGCTATGAAACACTTCCTTTGCCTTTCTTTCACTTTTCAGAAATACACCTGGTGGAGCC
AGCAGATACCTGGAATCACGTTGTCAGTAAGTCAACCATCTAGAGACCTTTGAAGCCTGGGCTGGGTG
GCATCCTGCAATCGTTGGATCGGAATCGGAATTTAGCGCCCTTACCTTTGAATACTAGCCACCTGCCA
GAAAAAGATTCTATGTACAAACCTAGCTACATGCTTACAAGGGAGGGAATGCTCAGACCAGGAGAAGT
GCCGGATCAGATCGGATGATGTGGGAGGGGAATCACCTTACATGCTATCAGCAATTGACTTGTCTACA
TAGAAATGCACGTTTTTATATCGCGGTGGAACCATTAAATGACAGGTTGAATATAGCTAGAAACGCTT
CTCAGAAGCTTTTTGATATCGACCGGACGGAACAACAAAAGGGATTAACATTGCTTTTATATCGTGTA
TATCGTGCTAGAGAGCGCTGGAAATCAGTGACTTGCTTAATGAAACATTACATTTTTAGAACCGGATG
AGACCCATCGGCATTACTTTTATATCTTGCTTCAGGGAAAGGGACGGAGTGAAATGCTTCCTAGATTT
TATATACGTAATTACGATGCGGCAGAAAAGCGAGTGGGCACCTTGCTTGGTTTTACCCGTACTACTTC
GGGCGGGCAACCACACTAGGAACTCATACTTCTTAGGTCGGTCGGGCCAGTTAGTTAATTAATTACCT
TCTACTGCGGCTCCGCTAGCCAGTTACCTAAAGAGAAATTTGAGTTCATGTGAGTTCTCGACCTCGAT
GGGGAATATGCTTTGGACCCCTCTCAACACGAAGCGCGTAGCGACGGAATGGATGGGATGAAGGGCAT
GGAATCACCCAGAGTAACCCCTTCAGTTTTGAATTGGCAGCAGCACATGCTAGAATAAACTACCTTGC
CCCTTTCTTTCACTTGCCAGAGCTAGACCTACCTGGAGCCATAGAACTGAAAGAATCACCTAGCTAAT
GTGAGTCAGGAATCTTGAGCCTTAGAAGCAAGCCCCGGGTTTATGGGAGGACGGATGGAGGAGGAACT
TACCAACAACCCAGCCAGGAATGAGACCAGCAAGATCTGTAGGGGACACTCTTTTCATACCCCTTACC
TTGTTCATACAATACACCTGCTATACATACATTCTATGTAACAGCCCTATATCATGAAGCCTTTAGAG
GGAATGCTATAAGACCAGTTTAAGTGCCGGATCGGATTGGATCATTGGATGCAAATGGGTGAGAATTG
GAATTGGAATCATCCACATGGCCATGGGAATTGGCTTGGGGATGCCTCTTTAGACCAGAATCCTCAGA
GCAGACAGTATACACTATAGAAGCCTTTAATACTTCGACAGGAAGCAGGGGCATCTAGTATACACTAC
CTTTTTACTATTTACCTGCACCCACTTGTAGACCTGGAGCTAGAGACACCTGGAATGCACGTTTTCAG
TTTCAGTGAGTCGATCATCATTAAAAGCACTTTGAAAGCAAGCCCCCGGAAGATGCCATTTATGCTTA
AGAACCTTACCCTTAGACCTTTGAATGCCGAACGAAATAGGCTGGCTAGCACACGATAGAAGAACTTT
ATTGAAACCTGGAAGGGATTAGCTGTAGGCAACTGTTTCAGAAGGCCCCTTACCTTGTTCATACTATA
CACCTCCCAGACAGATATTCTATGTACAGCCCTAGCTAATGAGGGCTTTGGAGGAATCTACAAGACCT
TAGGAGCCACAGACAGATTGGATCGTTGGATCTTGGATGGGAAGGGAATTTGAATCGGTTCATTAAAC
CTTTGAAGCCCCAGGTTTATGCCATGGAGCCACTTACCCAGGAATTTGAGAGACCCAGAATAGATGTT
GGAGAGGGACAGTCTCTTATCAGAAGGCCTTACCTTGTGGAAGGAATACTACCACCTGCCTTAAAGAC
TTTCTACAGCCCTAGCTACATGCTTATAACGGAATGACAAAACCAAGCAGGATCAGTACCGGCCGGAT
CTTATTTTATTTTATGCCAGAGGGGAGGGGAGAGAAGGGAGGATGGACGGAACGACGCGGATTAAATG
CTATCAGTCATATACTTCCCTAAAGAGAATCGCTTTTTAGAACACCCGATAACTGATTACTTTACCCA
GTTAAACAACGGGTTTCTTTATATGAAACATCCCAGATGATAAGCTTTTTCTGATTCACCGGAACCGA
CCTTGTAGCCCCCAATATTGCAGGCACGCTCTGAACAGGCCCGAAAGCTGGACGGAACAAACTATCTT
TTAATAAGAATTACTCTTATTACATATATAATAATATCATATCACACTTTTAACAACACCAGGGCGGG
ATTTGACTGTTACCAACCTTTAAGTCCACCCAGAATAGCTTTACCATATGGATATATTACATTCCATC
CACCCTTCTTACTCTCCTATCAAGACCGGACAACCTAGGACAATTTTGTCATGGAGGATGCCTAATCC
AGGACCTTCATCTTAGAAAAAGCAGAGCAGCACCGGCATTAGGGCATTGAGTCTAGAGAAGCCAGGAA
CAGTCCAGTAGCTGGAAGGTATCGCTTACATACCCTATCACTCTTTCACCTGCTACACAGACTTTCTT
TCTATGGAGTGGAGCAGTCCTAGCTACATGCTGGAAACAGGAATCTACCAGACCAGGACCAGTGCCGG
ATACAATTTGGATGGATGGGCGATAGGAGATCGGGAATCGGAATTTGGAATTGCTTCATTAGACAAGC
CACCTCCCAGACAGAGAGAGATTGTGGGAGCCCCATATCATTAGGTCTTTGTAGGCACTACTAGAAGA
ATCTTTGGAGCAACAACTGATTGGATCTTGCTTCGTGGGACGGGATTTGCAATTGGTTCATCAGACCT
TAGATGCCCTGCTTGCTTTATTGGGTTGCTCTTCTTGCCTTACCCTTATCGATTCTTACTGCTGGAAG
AATAGGCTACGACAATTCAGTGAGTCACCTCAACATCAGATCACTAGGGGACTCTTTACACCTTACCT
TGGTCATACTTGTTGAAGCTACATTTAGGGGCCTTGGAGGGAGGCACTGCTAGAAGAGCTTCATAACA
ACAGATCAGATTGGGATGGATTTAGAAATTCGCGTTATTACATATATAATACACGAACCAACCGACTC
ATTAATTAGATAGACCAGAGAGACCAGGAAGCGAAGATTCATTGCTGTAGGGAACAGTCTCACTATAA
CTTTACTTTGAATACTAACACCTGCTAGACAGACCTTTATCTGGAACTGTACTACAACATGCTGCAAA

GGGAATGCTTTCAGAACAGGACCGGATCATAGGCAATAGGTAGGGCATCGGGTCATTAGACCGAACAA
GCCCCAGGTTGATTCGATGGAGGAACTTACTCTTTAGACCAGGAAGAGATGTAGCGGTAGCGAACCTA
TCACAATCACTTTCCTTGTTATGACAATATACCTCCTAGACAGACTTTATCTCAGCCCTAGAACGAGA
GGTTAGCTGGACCCTTCCTTCCTAGAAGTAGTGGGTGGAAGGATATACTCAGAGGAGCACCAGATTCG
CTTGGTTCATCAGCCCTAGAAATACTTTAGAATCGTAATGCCTTTTACCTGCTGCATGCACCCCTTTC
ATTCAGTTATTACTACCCTACAACTTACAGGCTCTTTACCCACTACAGTTGCTTACAGAACCAGACCC
CTTACCTTGAAACCATACACCTACTAGACAGAAGAGAAAGAAGAAGGGAAGACTTTCTCTGCCCTAAA
ACGAGAGAGAGGTACTTTATACCGGTTACAGGGCACACTCCTAGAAGACACAGCAGCACCAGATTAAT
CGGGTGGGTGGGTTGTAAACAAGGGAAGGTAAGGTCTTATAGCTGTCCCTCCAAAGACTTAATGTTCT
AGGGCTGAGATAAAGGTCTTTCTTTGTGGCAGGTGGTGGCTTGTCTGAACAAGATCTAATGAACCGAT
TAAACATCCATCCCACGATCGATCCCATCAGCAGCTTTTTTAGGTCTACCGGTGACTACCAACACGAA
AATGCTCTGATTCAAGGTCTAGGGCTGAAGATATCCTATCCTAAGGTGGGTGAAAGAGTAAACCAAGA
GTAAGGCCATCCCTTCCAGCTACTGTTTGTTGTGTTCCTGGCTTACTTACTCAATCCCAATCCCAACC
CATTCCATAATGTGGTGCTGCTTCTAAGGTATTCTATTCGTGGTTCTACCCGGTAACTTGAAGGTAAC
TGGACCTCATGGTGGCAGCTACAGAGAAAGTCTTTCTGGCAGGTATCTGGATTGTATGAACAAGTAAA
GGGTATTCTGAGACAGCTGTTCCCTACTACTACAGATTGTCCTGGTCTAATATCTGGGTGGGTAAAGT
AAACATAATCCCACCGCTGTGTCTAGGGTTCTGGGCAGTAAAACCTAAGGGTAAGGCTGATTCATCCA
TATCACCATCAAATAAACCCGGGGCTATTAAGGTCTGATATGGGTCTAACTGGTAACCTAATTCTATA
GGGGTGCTCCATGCGTGGTAAGCCAAGCCCCTTATAAAAGTCGAGTAGACGTAGCCGTGTGCCCAGTC
CCTTGTCCAGCCCCTGATCCAAACCAAGTATCCCAGCCCGATCAACCCAGGTAGGATCATTCAAATGT
TTTCCGTAGCATTAAGTGAGGTCGAGGTATAGGTATATTAATCTTTTTATTCGTCGGGCTATTTCTTT
TGTAAAGCGCGAAGAGTCAACAAGCACAAAGGCCCTCAACCTCCAATTGAATCCATCCTGCCTGTCAT
ATAAAACCTTCCCGAAAGGCCTAGTGGAGTAAGAAGAAAAACCACTTTCATGTGCCGATGATATGTCT
ACCTCACAGAGGCATATTGCCCATTAATTTCCGCTCATCGCTGGGCTTGGATCATTGGAATCATTGCC
TTGGATCATCGGCTCGGCTGCTCAAGCTTGGGATTTTGCCCTTGCCTCATCGGCTGGGCTGGGATACT
TGACTCGGAGCAGGTACTAAGTTCGCTTGGAACTAATTTAACCGCTGCGCCCCTCATTCACCATTCTT
GGCTAAATAAATTGCTCTTTTTAGGAAGCCGAGTGATTTCCTCATCAGAGCAATGAAACGGTATTTTC
TCCTCACTGAACTATACCCTTTCAGATCGTTTACCTATGATTCCCTGGTGGTGATTCGATGACAGGAG
GTAATTCACTCGATGCTGGTAGGAGATCCAAGAGGCGATACAAGTGTTGATGCATTCAATGTAAGGTA
GGTGCCCATGTATTCTTGTCTGGGAACCGATGGAAGACGTCTTCTTGGAACCGATGCGCGATCTTACA
ATGGCACCTCTTTTGAGAAGCTAAGCCCATTTTTGTATAATTACATATATAATGCACGCTTAAAAACT
GACCTGCCAACAAAGGCGTTACCCAGATACCCAGTCCGCATTACACATTCAATCCGCATTACCGCATT
CAGCCCAATCCCCACCAGCAGTTGCAATACCCCGTGCCCTGGAATGAGGTGGCGGTACATTTCAATAA
TAAGTTACAACAGCTTGTTATATACGGAAATATACCCGATATAGCGTAGAAAAGACAATAATCAGGTT
GCGCTGCCGGTACTCATGCTGGTACTCGATCTGGTACTAAATGCCGGTACTCAATTCCAAGAGTCGAG
CCAAGAGGCGATCACTCGATGACAGGTGGTGATCCCAGGAGGAAATGCCAGTCATAGATGGGCTGGGA
TACTCGGCTGCTTGGCTTTTGGCTGCTTGGCTAATCGACTTGGCTTGGATACCGGGATCGGTTTCTCA
GTTGGGATATTCAGTTGGAAACATGAGCTGCTTGGCTGGGATTACTTAGATGGATGGAATGACTTAGT
TAGCAACTGGTCAATTTGAAAGTGAAAGGGTGGCCTATCCAAGTGGTAAATTCAGGAACAAAAGGTGG
CGTGTCAAGATGATATAGAGAAGAGAATGCTCACACGTCCTTGCCCGATGAAGGACAAGACCTACCGG
ACCTATCAATACGCCGCCTGCTTGAGCCTATAAATGCCCTATTTACAACAAACCCTTCCGCCCAGAAC
AGTCCGCGTAAGGCACAGTCCGCCCAGTAAGGCACCCCCCCCTGTAAGTCCAACTGGTCCCATTCCTA
CGGCGCTAGATCTGAGATACACTAGGCGATAGAGCCTTTCATAGTCGATCTTGATCATAGCCAGAAGG
GCCCAATAGCCCAAACCCCAGTAGTTGTAACCAACCAGTCATAGCCAGTCTCATTACCAGAAGGTGCC
AATAAACTTGTTGGAAAGACTTGTCGGGAGGACCTCATTCCCACACGGTCACTGTCAGTCAGACGAGA
GGAGATCCCCACCCCAGGAGGTGCCGATTCCCGATAGTTGATGCTGTCGATCCACTCGATGACAGGTT
GCAATGCCAGAGCTGCGCGGGCTTTTATACTTTACTCGGTTGGGCTTGGTTACTCAGATTGGCTTGGC
CCCTCAACTGGGCTCCTGAGATTGGGGGCTCGGCTACTCGCCTGGACTACCCGGATCGGAGCAGGAAA
TAAGTATTGTTGGAAATCAATTCTAAAAAACTCTTAAGTTACTCATTGGGTGGGGGGGCGACTACTAA
TGGCAAGTCCCTATGGGTCCTCTTTTTGAGGGATCCGAGCATGCATGCATAGCTCATCGGGGCGCTGA
GATGGATTTTCTTCTTTATGAAACATGCCCTTTCAGAACATTTCTATAGAGATTGGCGGGATGGGCAT
CATTTGCTTTTAGCTTGAGCGTCTTTATCCTTGCTCGCTTTACAAATTCACCCATCGAACGATATCAC
ACGATATCAATTATGTTACCCCGTGTTCCCATCCCACCTACCTGCTTGAGATATTGCTGCTTGCCCAT
TAGTACCGCTAACGCTCGATACCCGAACTCTATGGACGGAACACCGTTCCTACAATCCAATGAGGGAA
ATATGACATGCCAATACTACAATAGAGACACTGAACCACTTTCTTTTCACCCGCCCAGGAAGGGCACT
CTCTCTCTCCAACCTGCCCACCTACTTTCAAGCCCGTGTATAGTCGCTTATTCTGGCTTGGGTCGCTA
GATGCCAGCGGTCTTGGGTTGATAGATGCCAGCTACCTAGCCTGTGGATGGATATATTTGGTTATTAG

TTCTAGCATTGGGCCTAGAGAAAGCAAGTAAAGCAGGGAATTAGCAGTTCCGCCTGTAGAAAGCTGTT
CAGCAGTTCTGTTCCGTTCTAAACCGTTCATAACCCAGCAGTTCATCCATTCATAACCCTGAGCCCTC
ACCCTATCGAATCAATTCTATGGGTACTTAAAGAAACAAGGGACCTAAGGAAGAAAAGAAGGAGGCTC
CATCGAATAAACCATGAGGCTCTCAAGGGCGAGGCCCAAGCCGGACCCACTACACTTGCTATTCAGGG
CTATACTCACTATACATTTCAGGGCTACACTGAGGACGTATGAGGTTACACTTACTATTCCTGCCTCT
ACCGGGTATCTAGCCTCTACCTAGCCTCTACGGGGTAGCTATCGAACTGGTTATAACGTTGTCTAAAC
CGGGCTATAGAAGCCTCTGCCGAATAGCTATCGAAGCTGGGTAGCTATAGAGTTAGTTGCCCTGCAGC
TCGTTAGTTGCCGCAGCTAGTGACGCTAGTCGCCCCTAAAGAAGCTATTGACGCTATAGAATCCCTTC
CGTTCACCCTCCATTCACCCTCCTAAATAAGCTCAATAATAATTGACGTAGCAATTGACGTTCTAAAG
CTATTGACATATCAATACGAACGATTTACAACCCTCATTTCAAGCCCTAACCTCATTATACCAGGGTG
GGGTAGCGACCATAACCTGCCTATCACTACTCCATACATAAGCACGTTTGAGGGAAGGAATGGCACTC
GACCAATTGCCATAAAGACCGGTAGTAATGGCCCAATTTACCATACCAATTTCATCTCTATTTATCTC
TCCAACGGTAAGCCGTCGGTACGTACTTCGGGTTTTCATCCATAATCCCAGGAAGGGGCCTTTCCATG
CACGCACGTATCCACCCACATAAGCTAAAAGCGAGTTATTTACCTCCTGATGGAGCCATACCTGTTGG
AACCCCCAAACAACACACCGTGCCTTGTCACCGAACCTTCAGTAGAATATGCGACGGATAGACTATT
TTATTGATAACCCCTTCTTCTTGCTAGGATTTTCACCCTCAAACCTGCCTGGTAGGTGCCCCAATACT
AGGACCCCCGGTGCCACAAGGAAGGATGGTTCAGACCTTCTTACACTAGGTATCACACTATAAAACAA
CCCCTGATTGGAGGGACAGGTTTAGTCTACAGGAACACAGGAAGTGGTAGGGAGAACAGCTACCACGT
GGGAACTGCTTGATGAATGCGCAGCATTTAAGGTGATTCCTTCCTCTAAGGCTAGTGGGGCAGCATAA
AGAGGCTGGAGCGGGGTAGCCACCTTTTCATTATAGGCAACGCCACCCTTCAGTATAGAATTTATCTC
TTTGCACTAGGGTGAGGGAGTGTGAGAGCGACTTAGGGAGGTAAAGCTAGCTATAGGTCGTTGGGCAG
GAGGCTTAGGCGGGTGAGGAGCTTCAGCTATTAAAAGTCCATTGGGCACCCGAGAAGCTAGTTTCGTA
TTTGTAGATTGGTTTAATCGATCAAAAGCGCTCCCACCTAATGCACGTTTTCTAAATCAAAGTGGTGT
TGTTGGGGATTTGAGTTCTGTAACCCAGAATGGAGGAGGAATATCATGCCCAGGACCCCCGACAAACA
AGGAAGGTCTTCCTTATAAAAAACGCATATCAGAAGGTTTTTGGAATTGGGACCAATCGGCATGGCTT
TTAGATCTCTTGCTTCAGGGCATGGCTCGTTGTCTATTGCTTTAAATAAATTTCAATTGGTACATTGA
AGCCAGATAGACAACCTTTCATTGGTGCCAATAAGAGACAACAGCTTTTCAACAGCTTTTAATTAGTG
TCAATTAGTGTCAATAAGAGACAATAGAGAGGCAAGCTAGAACAGAGGCAACTAACTGCAATTCAAGC
TAGAACAACCAACTGCAATTTCTATTTTCATTAGTGTCAGTAAGCCAGAGAGCCTGCCAGCCAGTCTT
TCAACTCTGCAATTCAATCTAGAACAGATAGGCAAGCTAGAAACAGTAAGCTAGTCAGTCTTGCCAGT
AAGTCTTGCCAGAGAGGCAACCAACTACAATTTATTAGTGTAAATTAGTGCCAATAAGCCTTTCAACT
AACTGCAATTTATAAGTGTAAATAAGAAGAACAGAGAGGCAAGCTAGAACAGAGAGCCTGCCAGTTAG
CCTGCCAATAAGAGACAACAGAGAGGCAAGCTAGAACAGACTGCCAATAAGCTATTCAATTTCATTAC
ATTTGTAAAGCCTTTTAGCAGCAGTTTCAGCACTACTTTAACCATCCTCATTCCCAGCAAGCAGAGTA
GCACCAGCAGCACTAGTAGAACCAGCATTATGATTATCAGTTGTTCCAGGATCATTTCCATCAGCAGT
TGAGCACGGTCATTTATAAGAAACGGAATAATTCCCAGCAGAACAAGGATTCCCAGCCCAAGCAGCAA
AAGGAATCCCAGGATCATTTGAACCAGCATTCAAAGCAGCATTAGAACCAGCAGCCCTAGCTGCAATT
GGACCGGCATCATGACAAGGAGCAGTTGAACCAGCAGAATCAGAAGTAGTTCCAACACCAACTAGTTG
AATAGGACCAAGAGTAGCAGTCCCAGGATCATTCTTCCAAGCAGCATAACCAGTAGAAGCATCACCAA
TTGAACCGACAGCAGCCCAAGCAGAACGACCAGCACTATGACCAGCATTTAAAGCAGCAGCCGAACCA
GGAGCACTAGGATTCCCAGCAAAAAAAGCATAATGATCAGGAAGGACCACTAAAACCAGTGAACCAAT
AAAGGGCAGGCCAAGCATTATTGAAATCAGCATTATCAGGAGCAGCATTACCATCAGCATTCTTCCCA
GCATTCTTCCCAGCATCAGTGGAACCGGCAAAGGCAGCACAAGCTGAACAACCAGCACAAGTATGATT
CTCAGTACGAGCAGCACAAGGAGTCCCAGCATATTAACCAGTTACCGTAGCAGTTTAAGCCTAAAAAC
CAGGAGCACTACAATCCACTAAGCTTGCTGCTTTTCAGAGATCGATCCGCTCTTTCCCAAAGCAAGGA
CTATGCCATGAAACCCATTGTCGAGTCATTATCTTCATACCTATTGTATGTATGGCTTTCTTCCATCG
CTGTTCAATCTCTTTTATAGAGCAAAAACTCTTTCCACTTCACTTGTGTTACGGAGAATGCAATGCCG
CGCTTAACATCATGTTCTGATGAAGCCGGGTTGACTTGAGAAACGGGGCTCCTTTTGTTCAACTCCGT
GCACATCAAACGTTATATGTGCTTGGGAAGCGGAAGGACAACCATAGTGCAGCGTAGCGGAAGGGGCT
TTAGGTGAGCTCGTAACAGCAAACAGACATGAACTTGCCTATGTTGCTCCAGTTCTTCGCTGTTCTAA
AGTTATTCATTTAGGGTGTAGGGCACTTAGCTTTACGTCATTTATCTCAGTGTGCTTTTAGAGACTTT
AGTTATACGGCTCAGCCTAAGAAGATTAATTAGGGAGGCGATGAATGTGGGTTTACAAGCACATTGTT
CGTTCTGTCGAACTCGGCTTTTTGAAAAGCTTCACCCCGAGTTCACTTTACACCTTACGCCTTACACG
TTTGGATTTGTTTCATTATGGTTAGTTCCAAAAGAACTGGTAAGATTCAGATTCTACGATTGACTCAT
ATATAATATAGTGAGTGGCTTGCTTGCATAACTACTCTTTCTACTATGCAGCAGCCCACCTAACTAAT
TTCTGGCATGTGAGAAGATATAAGATGATTCTAGGTCGTTTGGAAGATGCAGTTCGAAAAACTATACA
CAGAGCAACCAAGCGTAGATCAACTCCTCAGAACTAAGTGAACTATATTTCTTCCCCTAAAATCGCTA

GCTAGAAGGTAAGGTTATCCCTATATTTATTCCCCTCAAGAGTGAGGACTTGTTTCCGATGTCACAGG
CTATGAACCTGTCCGCACCAAAGAGTGGAACGCCATCAATTGATCACGTTCCTTAATCTCTTCTCTAT
TTCTACACTATTCTTCTTGGCCTTGGCTTTGGCTGTACCTACTTTTTTTCGCTTGGCCTTATTGCTTG
CGTGCGCGAACTAAGGCAATAGTAGGTGAGTTAGCGTGTTACGATAGAATCCTGGTAGATCCAGTCAA
GTCCTGATTCAATACGTTAAAGCAATACTTCGTCAATTAAGAGCCATTGTTTTTATGGAGTTATGTAC
TTACTAACTTTACTTATTTCCTTGGCTTGGCCTTGGCCTTATTCTATCTCTAGTTACCGAAAGCATAC
GAGTCATTAAAGCCATACGGGTCGCAAAATAGCAGAGCTACATAAATACTTTCCAGGTAGGTGTACAC
TTCGTTACATATTGCAGGTCCGCCTTAAGCATCCTCTCTGCCGTGGTCCAGCAACCAACCCTATTTTG
GTTTTGAAGGTGTTCGCTATACTTTCCGTCCCTCTCAAGTTGTTTCTGAACAGTGAAAGGAATCCCCC
AGCTATTCTCTCTCCCATATGTATGTTCCCGGTCCGCTTTCCCTCTTACCGACCGGTATGATGAGAAA
GGGCGCTTGACCGATTTCTAAGGGTACGGAATCGGGTGATCAACTTTTCTTTCTTCCCCTCAAACTAT
CGTTCTGTCTGACTGAACCAAACCATAGAAGGCCGCACTCGACACACAACGGAGGGAGAGGGGATGGG
GGAGATAGGACAGGCAATCCCTAGTTCCGGGACAGCCAGCACCGCACGGAAACTCACACGAGCGAGAG
TGCCAGCCCACCGGTTCTGAAAGGACCTTATCTCATGAACTTTTCGAATACTTCGTGGAAGGTCAGCT
CTAGAGGGAGGGCAGACGGCCCTGAAAGGTCCAGCCAGTAAGGCCCTAACCCGGCCGGCTTCGACTTG
TCCTGATGGACCCGTCTTACTTAATCATTTGTTTTATTCTGGTTTTGCGATGTAAATCCAGTCAAGTC
TCGATCCTTCTAATGCGTGCGATTGTTTCTACGGATGGTGTGGTGTTTTTTCCTTATCCTTATCCTTA
TCCTGATTCTTTTCCTGGCCCTGGCCCTGATATCCGAAAAGGAAGGAATGAAGTCCGTACTATGCCAT
ACTTCCCTCGTGAAGTAAGGGGCAACATACATAAGGTTCTGATCTCTTTCCTTCCTTTTCCGGTCAAT
ACTTTTAAAGTAAGGTCCTGAAAGGCCTTATTTGGTCTTGATTGAAGGGCCTGGGTCGCCGGTCCCTC
GAAGAGTCCTGACTGCTGAAAGGTCAACCCTTCTAAGATAATGCCCTGAAAGGGGTCCTTAAAGGCCT
TATTTGGTTGGTCCAGAAAGGTCTTACCTGGATCCTGCCTGAAAGGGAAACAAGCGATCGATAAGGGG
CCTTATCATCAGCGCGGTCAAGCAAGTCAGGGGTGGGTACTGGCCCTTGATTTCTTCCCCTCAAAGAA
CTAGAAGGGGAAGTATTTAGCTTGAGCATAGTTCAGGCCCTGAAAGGCCCTTCGAAGGCTTGTCCTGA
TTCACCCTTCTCGCTATTCTTAGCCCCGTAATCTTTATTTCTATCCTGGTCAATTCGGGTACTTGTTA
GAAGGGAGGGATCCCACTCAGGTCATGCCTTCTGAAATCCCATCCATTTACCTCACATCCCACAGTTC
CGCCTTCTGATTTCGGCTCGACAGCGGACCCCATCACATTTATTATTATACTTACTGGGAGGGAGGCT
GGTATTCCCAGTGGCCATTCTTAGGCTTATTATGTCGCACTTGACTCATATCGACCTCTCTTGCTGCT
GATCCCGCCTACCTTCCTGCCTTATTAGATAAACCACCTGATAACCTCCGAACGACTTACCTTCGACC
AACTAACTCGACCAACTGCCTGAAAGATATCTTGCCGCTTGCCTTAGATCGACCTTGCGAACTTCCCC
TGATAAAGTCATCGACCGAACTGCCTGCCTTCATCGAACTTACCGCTGCCTTTGACCGGACTAACGGC
CTGAGAGGAGATATCTTGCTGCCTTATCCCGCCTACCTGCTTCGACTTTTTACCTGATTGAGACCCTT
ATTGAGACCCATCTCGATATTGCGACCTGGACGTCTATCCATTGCAACCTTACAACGATGCGCTTTAC
ACGGATAGCTTAGGCAACTAACTAACCCGTCATCAATAAGCACATACACAGTTTCACCTGTAGCCCGC
ACACCTGATGATCAATCTATTGATTGATGCTTGCTGGGAGAATAAGGGGCTTACAATATAACCCTAAG
AAGCTGGTCCCGTGGATGCTAGAAACCGAGGGCTGGGAGAACCAACGATGGATGAATAGATGGCGGGC
TGGCTGGTCTAATCGCCGGGGCTTGCTCTCGCTTGCTCTGGCTAGATAGATTACAGGCGGGCCCCCCC
ACCAAATAAGTAAAATGAGAATTTACTATCCAGATTCGGTCTTTGCCCGATGCGGACAAGCAACTATG
TACCAAAGATGGATGGAATATGCTTGCTCGTTAAGAAAGAAAGAAAGATGCTATTCACCCCTCCTGAG
GACCTTACCTGAGCTATCTCGGGAAATAAATAATAATATTTAGCATTTTCTTTTGGGTCTGCTCATCT
CATGTTCGCCAAGCCGGTTTGCGAGCCGGGAAGAGAAGGGTCTCTAAGCAGTCCCACCCAACTAAACT
ATTAATGAAGCAAGTGGGTTCCGTAGTTAGCTCAACAGGTAAGTAAGGCACGGAGTAGTGCAAGGTAT
GCGCGCTATAGGAGTGTCTGAAGCAAGGCACTTCAAGTGAAGGTTGGGTTCTTATTTCCTTCCCCGTC
AGCAGGAAAGTTATTAGGGTTGGGTTATCCCCCTGCTCTTTCCTCTGATGTATAATTACGTATATAAT
TACGTTTGCTGAAAGTCAGAACAAATCCCTTACTTACGCGGAGCTCAGGCTTTTAGGGGATTCGGTGA
TGCGCTTCTTGAACCAGATTGATACTCAATAGTCTATTTTGGCAAATAGAGGTATTTCCCTCGCTAAT
TGACCCGGCTAAATGCCTTAGTCCTAATAGATATAGGAACAGACTTCCTACTCGGCGTACGCTATTTC
AACTCTTCAAAATAAAGGACTATGTTCCTCAAATTGCGAATCGACGTATATTTAGCGATGTGTTATCA
GCATCTTGGTGGGCCGGCCCATGGTTCTTTTCTTTTACTGGTGGGCTATCGTCGGCACGGTAAGGTTT
AAACACTCTGAACTGCCTGCTGATCGATCCAACGTGATGCTTGCCGAAGGGTTTTCTCTTGCTTACTT
ATGCTAACAATATCTAGGGATGTGAACACCGAAAAGACACAGTCATGCCTACCTGTGTTTCTCTCTTG
CCGCCTGCTTGAGCCTAGAGCTTACCGTCAAGCCTTGTCCACACAGTAATAGTCCGCGTAAAGCACAG
TCAGGACGCCCAGGAAGGCACCCACCAAGTCCGCCCGGTCCACCTACTCCTACAGCAACAGCACATAG
AGCACCTCACATAGATGCCTCACACCATAAGCACCTGACATAGCCGATCATAGCCAGTTGTCGTTGCC
AGGAGGTCATTGCCGAGAGCCAGTAGTACTAGCCTTAAGGTCATTCCAGTTGTCATAGTCAGAAGGAA
CCGATCATAGCCAGCAGGAGGTCTCGATCATTGCCAGATGATCATTGCAATACAGCCAATTGCCAGCC
CCTTTACCTTTTATTTGCGGGGCTGGGATACTCGGCTTAGCTACCGGGCTGGGATGGAAACAGTCGTT

GCCTATAAGCTCTAGTCAGTGCTAGAAGCTCTTGTCACTGCAGCTACAAGCTCTACTCACTGCTGCTA
GAACTCTTGTCAGTCAGTGCTAGAAAGAATAAGCTCTAGTCAGGGAAGCTCTAGCCAGTGCATGAAGA
CCCAGTGCAGCTAGTCATTGCAGCTACTCAGTCCCAGTCAGAGCACGGAGCTGCTCACATATACCCAT
ACAAGCAGACGCAGATAACACACGACACAGATAACACACACACCACACAGAGAGACATTCAGAGAGGG
CACGTTGAGACATCACTAAAGCCATACAGGAAAGGAAGGAAGAGAGGGGGCTTTAAATCATTGATTTG
CCTACAGACAAATAGGCGGACACCTGGAATCCATCTTACCAGCCTTTTCATGACCTTATTTAAATGCG
CGGTTGGATACGTTTTTAGTATTGACCGGACCAATCCCAATGGATAACTAGAGCCAACTTTGAGTTCT
TGATCCCAGATGGAAATATCCTCGGACACCCCAACCAACAAGGTTTTACTTTATAAAATGCCTGTTTC
AAAACGTTTTTTATATCGACCGGAACCACTCCACGAATACCAAACTTGCTAGTAGTCGGGTAGCCTAT
GCACTTTGAATAGAATAGGTTCTCCTTCTAGTTCCAGCGTTAGGTACACCCATTTTGCATATCTACGT
CGTTCATTTTGAACTACCATTTTAATATAGTTGATCCTCTTTAGCAGCACCCCATCTGAAATCCTGGC
TCTGCTCCAGAGAGCGAAGTAAAGTCGTATGAGTTAGGGCACCGAAGCGAACTCCTTCTGCGGATATC
CCATGCCCACCCCTCACCTTGGGGAGTTAGCATAGGGGGAAGGGACGAATAATGCTGACCTGGGACTG
CTGGTTTCTTAGTTCTCTGCTCTCGGAGAAACTTCGGAGGAGCTGCTTGAAACCCTTAATAAAATGGA
TTTGATTTCATGCCCAACTTCGCGGAATACCTAAATTTGAAAAATCGATCAATACATCCATCGTGTGA
TATAAGCTTAAGTTCTGTACGATATCTGCTTAAAGGTCTCCCCCTATTGGCTAGGACCATCCATCTTT
CGGTGCTGCTTCTAATCGAAGGGATCATAGCAGCCGTCCCGACTTCCTAAGTGCATTCATGATAGAAG
GCTACAGGTCTAGTTCTTCAGGTGGATAGGTTGTATGACCCTTAATAAAAGGCAAGGGGCAAGGGTGA
GAGGGTTGTTCATAGTAAGAGTGCCGTACCAACCAACAAAGCATTCATTAAGCAAGTGTTATAAAAGG
CTTTTCTTATCCTAGCAATGTCTGAAGCCCGTCCATCCTCCAACGAGGCTCTCTGATGGCCAATAATA
AGAGTTATCCGGCAGTGATATCAGCAGCTAAAGGTAAGTAAACCCTCCCTCTTTCTTTATTCGAGCCG
AGCCTTTATTCAATGCTCTGATCAATTCTCTGTATCTCCGCATCTCCACATCGGAGAAGTATCCGTTG
AAAGTATCCGTTTTTCCTTATTAATTACAAGGAAGCGCTGTTGATAGGCATTTCTTAATAAAGAGAAT
GGTCTATTATGGTCTATTAAAGAAAAGAAGGGCTTGCTATACCTATGCCATGATCAGTATTAGTGTTC
CTGGATCTCCTGCTTCGGGCTTTGCGAGCATTTTAGAAGTGGGCGCCCGCTATCAATCATGTGCTGCG
GGTAAAGGCAGATAAAATGATTGCGGATACAGAGGGGAAGCTTCAAGAATTCTGGCCCCGAGTTAGCA
ATGAGTTGGCTTAAGTCCCAGAATACATTCCCGGCTACCCGCTGGCCACCATAATCGAGTTTATTACG
GACGCCTGGGACATGGATACCCCTCTTTGTAGGCGCTTTATACTTATAACTCCGAAAGTTGCTTATGT
CCCAACCTATGCCTTTGCTCCTGGTACTTTAGCCTATACCTCTGTACCTAGCTATGCTATCTGTGCTA
CTGATGCTACTTGGTCTACTGGGTAAGCTACAGGTACTACTGTTGTTGGTGCTGCTGTCTCTGACTCT
ATATCTCGATCTCGATCAACTTCATTTTTAGAATCAACCGCTGCAACTGCTGCTCCTGGTGATGGTGC
TGGATATACTGGTACTTCTACTGGATCAATAAGGTAAGCTGGGGCTGGAAGAGATGCTAGGCAAGGTG
CTGGCTCTAAAACTAGGAGGGATGCTACTACTAGCTTGGATGCTGGCTATCGATCTCGATACGGAAGG
AATGCTGAGGGCTACGGATCTATAGGTTCTGGATATCAGGCCCGAACTGGATCACGATCATGAAGGTA
AGACTCTGCTGGATCTCGTTCTTTACAATCATAGTCGTAAGCGGCTGCGAGATCCACCGCTGGATCCA
CTGCTACTGATAGCTGTGCATCCATATATGGATATTAATATGGTCTAGCTGGTATAGCTGCTGCCTTT
CCTGCAACCGCTGCTCCTGGTTCAAATGCCCGAATTCATAAGGGAAAGGTAAGGTGATCCATCCATCC
CATCATCCGGTACTTATCCATCCGGTGCTTATAAGATAAAGTAAGTAGCAGTCCCGACTCACTAAGTG
CATTCATTAATCAAGAGATATAGGGATGACCAATCCGGAAAGAAATCATCGTGAAGGGACGGAGACCA
ATACCAAGGCGGAGGGACAATTACGAATTAAATGAGTTTCCTATAGAAAAAAAACAGAGTTCTTGACC
CCACAGTAGGCAATGACGCCTTAAGACATCCAAAACACATATTGGTAATTCTGAAAAAGTGTGTTTGG
TAGGTTGTTTTGTATCGGCCGGACCCCACCCCCGCGAGCATGAGCGGGCAGCACCGGACCCCAAAAAA
GGAAACAAGATAGAGAGGACAGCCCCCCCGTGGTATGCCCTTGTTGATGTTAGTGTAATTAATACCGT
AACTATCCAAGCCTTTGCCTTTGCTGGCTCAGATGCTTCTTCCTTCGCTCTAGTTATCTTTGTTTTTT
GTGGTCCCGAATCAACAGGCTATGCCTATCGACCTGGAAGGTACGTTGGGCGGGTTAATGGCTCTCGA
ACTTACTTGGATGGCGGTGAATCAAAAGGCTCTGGATCTTAATCTGCCTCTATTGCCTCATAATCACT
TCCAAGAACAACGGGCTATGCCTTACAAGCAAGCTATCGATCTGGGACTGGAAGGGGCGCTTGTAAGA
AAGCTGCCCTAGCTAATGTGATAGGACAGCAGACGACCCTATTATCGACAGCTGCAAAGGCTCCTCCG
ACGAATTAGGCATTAGAAGCGCCAAGAAACCCAACCTTGGTAACAAAGTAACAGTTACACACTAGCGA
GAAGCACTGATAGCCGTTACTACACCCTGCCAGTAAAATATCCAAATCCATCTTGCTCGGGAGCCCTT
CCATTCCGAGTACCAGAGTCTCTAGATTAAAAACCATCATCTCGCCTCGCGTTCCTTCCCGTTTGAAA
GGCATAGCCTCCCCCGGATAAAGTCGAATAATAACTACCGGGGGCAGAGCCAGTAGCAGAAGAGGGAG
CAAGGGCAGCAGAAGTAAGGGTAGCCAGAGTAGATACAGGGACGGGTGCACCATATAATAAATATTTA
GACCCAGCTTATGAAGATACAGATCAAGACCCATATTCAGTAGCAGAGACAGAGTCAGCAGAGCCAGC
ACTAGCAGCAGTAGATTAAGGAGTCGAAGATCCAACAGCCAGCTTGCCAGTAGCAAATCCTGCAGAGC
CAGTTGCTATGACAGTTCTAGACCCACCAGTATTGGTAGATCCAGCAACAAAGCAAGTAGCACCGATA
GTAAAGGCCCCTTTAAATGGTTATGCCCTTGGTTACGCCCTTTATTATGATCTTGATCCACTTGATTA

TGTAAGTGATTCAGAAGTCGGTGAAGCAGAAGTAGATCCAGCTTATCCATCATCAGTAGAGGCATAGG
TAGCTGGAACATATTCTGTATAGTTTACCCAGTAGCTTAATAACCAGTTGGATAGCCAGAACCAGCAG
CTAAGACAGTTTATTAAAGAGATCTTGTTGATTCTGTTGAGTCATAAGCATAAGCAAAGGCATTAGAG
CCAGAAGCAAAGGTAGCATAGGTAAGAGCATAAGCAAGTGCAAGGGCAGGGGAAGCACACGTTGGTGA
GTACGTTGATCCAGCAGATTAAGCCTTTGATTCTGACCTTGATTATGCAGTCCTTGATACAGATCCAA
AGCCAGCACGAGTAGCAAAGCTAGTTGTTTACTCATAAGCAAGGGCAGGTGCCTCAGTCGATCCATAT
CGAGATCCATACCCAAGTTAAGCTTTTGATCCAGCACCAGTATCGGTATATGAAGCAGCAGTATATTT
ATCTATATATTAAGCTGTTGATTCTGTCGACCAATAAGTAGGACCAGTAGCTTCGTACCTAGTTTACC
TTGTTATAGCACGTGTATAAGCTTTTGATCCTGTACCGGCAGCATACCTAGAAGCATACCAAGCATAC
CTAGGTACATACCTTCCATTTCAATTGATCCAGATCGAGAGCCAGCCAGATCCTGCATCTTGGAAGGA
AGCGCTTGCTTGGGTGGGCAGGCACTTTCAGCATTGCTTGCGCACCGCTACGCCCCAGCAGCCTAACT
TCCCTTTACCTTAACTGTTACCAACAACTCTGTTATTTTTACTTGAAGACTCCGTGCCTCACCTTTTG
TGGAATATGTGAAGAGAGGGACTAGTATTTATACACCTTCTTAAAGCCACGGGTGGTAGATCTTCTTT
AGAACCGCCTTTAGTTTCTTCTTATTGAGCACTCCGCTACGCCCCTCATTCCATGCCTTGAAACAGAT
AAACAAAGCAGAACTCTGACACCAACGGATGAAGATTAGTTACTGCCATTTCCTGTTACAAGCCCTGA
GCCATAATAAGATAAGTGGTTATCTTAAATAAGCGTTCTATGCTACATAAGAGCATTCCAGGAAGAGC
TACTTTATCAGCAGTCTATATTTTGCTACATAAGAGCTTTATTTTAATGGACCTGTGAACTGGTTAAA
CTGAGGTTATTCTCTCTTATTGATACCCAGTCTGGAACTGATAACCGTGTCTTTCTTCAGACGATGCA
TTCTTTGCTTCCGGAAAAGCCCAGTTAGGGCATAAGCAGTGAAAATAAAAGGTGCACTTCCAAGCGTA
GCGAGGGGGATCATCGATCTTCTGATTTGGTTGAATCACACTTGCACTACGACCTCTGCCTGCTATCT
TGGTGGGGACTTTAGATGGATCTGCTTCAATTGGTGATGCTACCTTAGGTGGGGACTTTAGATGCCAT
AGGAGTGGGGGCTTAAACTACTAATAATTGATGCTTTGCTTCTAGTATTTGCTTGCTGATCTATCAGT
TCTATCAGTGGAAATTGCTGCATCAGTGATGTGTGGGTGGTGGCATGTGGGCAGCTTACTGTTTGGAT
ATGAAACTGGTACAACGACAACTGGAATTGGTTCTTAGTCAACTGGCTCTGAAAAAAGCATAATCTAC
AGGGATTTATCGCTTTCCCGCATCGGGGAAGGGGGCTGGTTTCACGAATTTGGACTGGTTTCACGAAT
TTGGACTGGTTTCACGAATTTGGTTTCCTATAAGAGGCAGGTCTTACCCCCCCGTTGACTGGATTCTG
TATTCATCAGGTTGGCAGGGAAGGGCCTGAAACTAATTTATATATTGAATTGACTTACTATAACTTAT
GGATTGACTGATTAATTCACTTATAGATACTGACGAGGCTGCATTAACTCAAACTTTGATCACCCGCT
CTTGGCAAGAAGTTGAGGGAAAATCTTTCTGCAAGGAACGGAGTACTCGCCCGACCTAAGAAGGGCGA
GGAGCTTTTCGCGGCTCAACTGCTTAGGTTTTAGTTACTGAAAAGTTCGTTTCTGCCTAGTGCGACGC
TAAATCCATTTATTTCACCGTCGATTGAACCAAAAAGACGCTTTCTCTGAGTGAGTCTGCCTTTACTG
ATGCAGGCTCCCTAAATACTCTCGTTATTGTTTGCGCTAGCTGCGTTGGAACCTTCCCCTTAGGTGGG
GCATTGATCCCGGAGTGGAGGATGCCCATTACTTACCGGGTTTTCCACCCTTTTGATCCTAAGCCAAA
TCTATTAACCACAGGGACTTCTCGTGTATTATATTATATATGCCTTTTCTGAGCCTTTTTACGAGGAC
GCCCTTCCTTTTACAGCTCCCCACGCAATCTCCTCTCGTCTCGTTCTTTGCCTAGCGGGGTTCGGACT
TCTATTTATCTTCCCTCAAACGGGCAGTCATCCTGCAGCGGATAATGCACATTTATTAAGCTTTTGAC
CTTCCTTTGTAAGCTCTGGTATCTCTGGACCCCAAAGCACCTTCCCGTGCTTTATATTATATTAAATC
GATCATTAGATCCATAAACCTGGAGCTCTTATACATGTTCGCATAGATAAGGAAGTAGACTCAAGTAA
AGGTCTTACCAGATATACGGTTGAAACCCTTACAGGACTTACCGGAATAGACTTTCTCTATGCTAAGT
TGCCTCGGGCTTGGACCCGCACCTCGTGCTTGTTGGTGCGCGGATAATCCCTATTGCTTCTCTCGATA
GCTAGGGTCGTATCCCCCAGCACTTATTGCTACCTCGTATCGTACTTCTATTGCTGGGCTGATAATCT
GTCAGTGACCGAACCCAAGAAAGAGAATTGGTCAGTGATAAGCTGTGACCCTACTAACCTATAGTTGC
AGGTTGAAGCTGTTACCGAAGCAGAAGCAAAGATAAGATAGTTGGTCTAATAACATCTTTCCAAGCTA
GTTGTTCAGGCAGGTAAGGTATAAGCTTTGAACCAGACCTCGTGTCCTCGTACCTCGTGGCATGGGGA
ATACATTCTCACGTGAAACAATAAATGGAGATGCTGTTGTCTCGTGTGAACTCAACCTGGTATACACG
AAGTTGTACAAACAACATGAAAATGGTCTAACCAGATGTTGTCCAACCAAACCTGGACGAACCAAGCT
GATCACGGGAAAACCTATTCTCAATGGGAAGATATACTGTGCAAAGCTCAACCTCGTATCGTCCCACC
AATTGTCCAATAAAAACCGGTATAAACGGAACTTGTACCATAAAAGTGGTTAACCCAAACCTCTCTTG
AAACTGCTGGTCCAACCGAAACATACCCATCGCTAGCTAACGAATCATCCAAGTCATCAAACCAATCA
GTTGTCCAATAAACATTGTATAACCCGATCTATGGGACACGAACAAATGGTCTAACCCGAACCAGTGG
CACACGAACCAATAGCTAATCACGGGAAAAGGAAATAGTTCTCGTTCCTCGCCCTTCTTAGGTCGGGC
GAGTCACTTAACGTACCTCGTGTCCTCGTACCTAGTGTCATGGGGAATACATAGTCCCGTGAAACAAT
ATCATGGGAAAGATAAGAAACAGACAAAGTCTCACGGGAAACCATTAGCAAGAGCTCGCGAACCCTGC
AGAGCCAGGTGAAGCCTAAGTAGAATAGAAAACTTGTTTGTAAAAAGCGATAGCCTTTCGCGGCATGG
CCACCGGTAAACCTTTATGTCCTGCGGAGCGAGCCCCCCGACCCTTGAGTAGCAGGGTTGGTAAGTAT
GGGAAGGTAGGTAAGTGACTATCCTGTACAGTGTTCTGGCTGTATCCCCATTAGGTATCTGAGGTAAG
AATGTCATTATCATTCCCCTTTAGTAAAGCTTTGTTTGGGCGGGTAATAGAGGGAAGAGTATTTTGCA

TTCCAATCGGTAAGAGAGAGAAGATCCCTAGGATGGCACCCTTTCACTATCTCTTCACCCTTAAGCAT
CTTAGCATGCCACCCTTGTATGATCTCTTGACCCTTCCAACCGGTAAGGGAGATAGAGGAGCCCGGCC
TCCGGAGGAGACACATTTCTCGCCCTAACTATCTAACGCTTCCTGCGGTATAAGTAAAAGTAAGTGTT
TTATGACATGTTCGATAAGGCATTTAGGGATTGCCACTCAAAGTGAATGAAATAATATTCCTAGCTCA
TAATAAGAGAAGATATGTAGACTGTAGCTTGGCGGTGGGTCTTGCTAAAGTATCTTACATCTATCAAA
TTCCCAAGAGGGATAATTTGCTTGGCGGTAGGTCTTGCTCAAGGCTCTGGCTTGTCTGATTGTTTAAA
AGGAGGGGAAAGCTCCGCAGGACAAATCATGGTAAGGTGTACCCCTTTTGCTCATAACTTGAAAGACT
AGAGATGTACATTAGGCTCTGCCCTGGGGGATCAATACCACAATAGAACTAGATCGCTTAAGTAGGAA
AAGGAGGAGTCGGTCTAGTGAGTTCAAATAGCATATACTTGTAAAGGATAAGAAACTTATCCTCGATG
ACGCATAGGATCTGCTCGGGCTAACCACCTTGAAACTAAAAAGGATAGTGAGGGAAGGAAAGGAGGGC
GAGGTTCCGGCACTAGTAAGGGAAGGTACGAGGCAATATGCAGGGAAGGTTAGGATAGAACTAGTAAG
TATGTCTCCTCAAAAAGGTAAGTAGGGGCAAGGAGAGAGAGTACCTTAGCAGCCTATGTCTAATGATC
AATAGCACTTTCCTGGGAACTAAATACGACTTCTTCGAACTAAAGCATCTTCCTGGAAGTGAGATAAG
TGCCCTATAAAGTAAAGAGAGGGAGACTAGAGCTTGGCGGTAGGTCTTGCTGAAGAATCTTTCTCAAG
GGGAAGTATCGGGGGGGATTCTGTGGAGCTACGGGAGCCCCCCCGAGGAGAGTCCAGTGGGTCTCTTT
AATTGCGGTCTAATTACTGAATCTATGCAGTCACTATTATTCGTGGGCTGTTGCCCCGGGTGTGTTGG
TGGATAGGGCTTAGGGTGCTATACGAACTAGGGATGGGAGCGTGCTTTATAGATAGGGGAGAAAGCGT
ATAAGGAGGAAAGCTAGGCAGATGAGATCTTGACAATTATGCTCATTATACTGCTCCTGGTTCCAATT
AAGCTGGAA
>Repeat_14
AGGTATTTTGGGCAGAGGTGCTATGGGTCCCTGAAAGGAAGGTTACAAACAAATTACAATGAAATAGA
AGCCGAAGTGCGTTTCACCCGAAATAAAGAAATATTAGGAGCCTGGAAAAGGACCCATCCCACCGGAT
TAACAAAGGGACCACCCCGGACGGGATTTAAACAGGGACACCCGCCGTCTATCAATGCTACTGTTTGG
CTATCAATGCTCCTGTTCGTGAGACGGAGGTTCGTGAGACCATGAGGGATTGCCGTACGGGCAAGGAC
AGGTACACTTGAAAGCACGAGGGGTTGCCCGTCCCAGCCAAGGGGGTCTTGGACGTCAATGGAATCAT
TCAAGTCTTAGGGGCGCCTGGACCCAGCCCAATATATATTAGGGGTTTCCATCAGCCAAATTGACTCC
AATTAGTTCGGGGTTTTTTTTAGTTCTTGGGTTTTACATAACATTAGTTCGTAGTTACCATTTTTACT
TGATCTCGGCTCCGGACTTAGCAAGGAGAACAACCGCCCCCCCATTTTACACACGTATGAGTCGCATT
TCGGATGAGGCCCTACACACAGGGCAAAGAAGATAAGCGGAATGCACATATCCCAGAAGGAGAAAGAT
CAAAGAAGCTAGGGGTCCCCATATATATTGAATATCCCACACTTCAGTCAGGGGGGTTATCAGCCTTA
GGGGTCTCGGCCAGCAATCCAGAGTCGGAACGGGCATTGTTCGGGGTCACACACCAACCCAGTGACAC
ATAGACACCGCTGCCTAATAGACAGTTAACGGGAAGGGGCACAGTGACACATAGACACCGCTGCCCAA
TAGACAGTCCCTGGGGAGGGCCTCCGTTAGCCTCGAGGACCTTTACAATCTATGTATTATGGGCATAT
TAAGTACCCGCAGCAGCCGGGTTTATCAATGCAATATTACATCTATAATAAGATCGCAATACAAGCGC
GAGGATGCGGCGGTTAGAACAGAAAGATTGGACACCCTGAGCCCAGCAAAAGGAATGCTGGGACCAAA
GTCTAATGCCTCGATAGAACTCCTCCCCCGACCGTAGCTTGAGAAAACCGAGGGACAGATACATAGGG
GACGGGAGTCCCTAAGAGAAAACGACCCGTTTCAAAAAAGAACTTATAGTCCCATTTCATGGACGATA
GGCACTCAAGAGCTATTACCCATCCCACTGCTTACCCATAGCACCACCTACACGTCAGGTTAAAGACC
AGATTTACACCTATTATATGTTACACCTAGGCCGTCCAGGGAGAGTTACCTACATTAGTTACAAAAGA
ATCAAAAGAGGCGCCCGCCACAAAGAGACTGACTTTTGAGAAGCCCATCAGACCAGATAGATCGCGCG
GCCCAATCATGGTCGTCCCGGTCGTTTTTCCAGCCCCCCCACTCCCGAAGCGCTATCATGCATGCTTT
GATTGGTGCTTGCTCGCCGCAACTTCTATTTGTTTTGGTGCGAGTCTCGCGTAGCCATACGCTTCTCA
CTGGTCATTGTTCGGCCCCGAAATTTCGTTCATTCATTCAGAGTGTTAGATCGGGTCAGTGGGAACAA
TGCTAACAGCCCGCAGTTCAGGGAGAAGAACGAGATCCGAAGGGAGGGTAATGCCAATGATCGATCGC
ACAGCCGCTTTCTTTCCTCCGTCCCCCTCTTAGATTGACTCTGTACGGGAGGACGGGGGAAGGCCTGG
AATCCGGCGGAGCTCTTCTCTTCCTCGGAGTCGTTCCATCATCTAATCTAGTTCGGCGTGATTGCGTA
ACTATCTGCGTAGTGGGGGGGAGGCCCGGAGGAGCTAAACTAAGGGGCGTATCCGAAGCTCCCTATTT
TATTCCCCCCTCTACGAGGTTCCTTATTGTGCCAGTTCCCAATCCCCCCGTTCTTGGTACCGCAACCC
GCCCGGTACGTTCGAGTGAATCGCTTGAGAGGAGATACCATCGATCGTAGGTCGTCGATATCGCACGG
GCCCAGGAGGGAGGCGTCTTAGTCACGTGATTTGATTTCCTTCCACGGTCACATGAGCTTTCGGGAGC
CGTTCTTCTATATGTATGTCTTTCGTCCAGCATCTCCCCCCTCATATTAGCCAGCCCCCCGTGGTTGA
TCTGTTGCAACTAACTTCCCTGCGCTATCGCGTTCGTCCCTTCGCCTATCTGGCAAGGCAGGCTCGGG
TGGTTCTACTTGGAACATCTCCTCACCTCACCCTTGGATCTCTAGCTGAAGCCAGCTCCCTTCCCCTT
CCAGCGATACCAACAACTTTCTTTCCTTCCTCTCAAGGCCCCTTTATAGAGCCGTTCAGAGCCTCCCT
TAAAGGTTCTCAGCTGTTCTATTATTCGTAGCCCAGTCTTTTCCTTTCCGTTGAGCCAAGCACAAGCC
GCCGAAGGAATAAGCATGTCATGTTAAGGCTAACGATTCCTAAATAATATCCCCTTTCTTTGGTTGAG
CTTGAGCTAAATCACTTGATTCGAAGGTCCGAAATTCCAGAAAGTGAGTTGATGAGACGAGTGCGGGT

CGATTCGATAGGAGATCGCCCCCAACTATTCTCTCCGGCCGGGTCGAGTGCACAAAGATAGATGAACC
TTAAGAGTGCATTAGCGCTATCTCCCGCAGATCCCAAGTGTCACTTAGGGATCAACCGAGCAACATTT
CTCTATATCATCGGTTGAGGGTCTTAAGAGGGCGGGCTCCACGGGCATCTATTTATCGGTAACGGGTC
GATCAATCAATCTCCGAAAGTGAAGTTGTCCATATTCTCGAGTGCAGTAGCGACGCAAAAACGAATGC
ACGCGTTCATGCCAAATTCATATATATTGGATCGGTCGAGTGTCCAAACGAAGGAGATCGAACCCGAG
CAGTTCAATGCCTGAGAAATGTATCAGAGGGAATCGATGAAGCAGGTGAAATGCCATTTATTTATTTT
TTTATTTCAAGTATCGGAATGCAAATGTCGATGTGGTGATGGAATCCAATCGATCAATGGGTCGTCTG
ATCCCGTATCAATAATGGGTTCGGGTTCCACGGTTGGCAGAAACAAGAAGATTATGCGAAAGACAATA
AGAGAAGTTGAAACTCCGAACCCGAACATCCAATTGCTCCGCCCATGCCCGTATCTCTTTTCCTATTC
CAATCCGGTCGACCCTTCCTTTCAGTCAGAGGAATGCTGTAAGCGGCGCTAACTGGTAAGCGGATAAA
TTAGGTCCACTTGGTAAGCCCTGTATTTCTTCCCCTCAAGACCTCGCAACTGGTTTATAATGACTCGT
ATAATGTCGGTAACTAGAGCCAGGGAGACCAATAAGAGAAGTTAAAAAAAAAGAGAGAGAAGACACAA
CTCTCTAAAAGTCTACTACTATTTTAGCCCAACTACTTAATTAAGTAGTTCGTTAGGTCAATACTCCG
GGAAAGGCGGAGCAATTTGGGGGCATTTAAAGTCTGGCATTCGTAAAGCAGAGGCCCCCCTTACTGTG
CCCCAAAAGACTTTCCTAGTGTCTTGAGCCAGGAAGGCGTAACAGATTGAAGCCAAAGCCAAAGCCAG
GCTATATATAAGTAGGGGGCCAGGGCCAGCCAAGGTAATAAGTAAGTGAAGTGGATTTGACGAATGCC
GGACTTTCCGTCGGCATGTATTTATTTAAAGCTCTCTTTGTCTATATACTATATTCTGGTACGATTTG
GGAGATTATATTTCTTTACTTTTACTACTCTCTTTCCCTTTCGAATGCATTTCACTCCCTAGACCCTC
TCTCCTGAGTGAGAGAGAAAGAGCTCAGGTAGGTACTACTTGAATTATATATAAGGGGGAAAGGATAT
AAGTAAGCCAAGCCAAGGAAATAAGTGAGAAGGGTTGGGTACTAAAATTATAAGGGGGCTTAACTAAC
TACTAAAATTATAAGGGGCCTTCGCGTAGCTCAGGTAGGTAAGGTACTTACCGGCCGGGTATTAGAAG
GGTTGGGATATTCGTAGCTAGGTAGGTGGGATGCCTATGCCTGGCATGCTCGTAAGTAAGGTGTTATG
CCTAGCCTGGTCCAGGTAGGCAAAAGTCATATAGCGCCTAGCCTGGTATGCCTGGCATGAGGAAGGTA
GGCATGCATGAGGGAGGGTAGGCATGAGTGCGCCCAGAGGATGGCATGCTCGTAAGTAATATGCTTGG
GTAGGCATGCATGGCAGTAGTGCCTAGCCTGGCCACCTACCATATCGCTGGTAGGTGGGGTGCCCATG
CCTGGTTGGGATACTATGGCGGGTATGAGAAGGGCCCGAAATGAGAAGGGTTGGGATACAATACAAGA
CCTGGCATTGGAATGACTTTCCAGGTGTACACTTACTTCGTGACATAAGGCATAGCATTGAGGGAAGA
AAACAGGGCACGCACGTTATAGGCGGACCTATTTTATACAGTCAAGTGCATGTAAATAAAGTAAGAGG
CATTGCCAGGACCCCCGGTGTGGGCCTTCGTTACATAAGGCATGGCAAGGCCCTTACACGTTACTTAG
GCATGGCAAGGCCAAGGTAGGTGAATAGTTCTAGGCGGACCTATTTGAAGAAGAAAGGCCCTATCAGG
TAGGTAGGGGTAAATACTTCCTTCTAGGCGGACCCATTTGAAGAAGAAAGGTAGGTCTTCTTCAGGGT
GAATACTTCGTTCCTTCCAGCCAGTAAGGCCTTGAAAGGTCTGGTCCTTTTAGGGACTTCGAGAAGGC
TTCGGAACTAAGGTATTCTTTTGAAAGGTCCTGGAGGGAAAGGACTTATCACCTGAAAGGCCCTTTAT
TTATTCCCCTCAAAGACTTAGCTCTGCTCAGGTAAGGGATAACCTACACAAATTATGGTATAACCTTG
ACGGACAATGAGATATCCATCCGGCTCGACTCTACCGTCCATCCATAATGCCTTCGGTCCCGTTCGAC
CGGTTCCACTCTGTAGAAAATGCCGCCATCCATACGGAAGTCCAGCAAACGCCCCCCCTACCTATAAT
TATAGTAGCGCCCGTAGCCGCCGAATAATAGGTACCCAAACCCAATGCAGAGTTAGTGAGCCGTGTAA
TAGGCGACCATCTCGCGCGGTTCGGAGGGCACTTGAGTAAGCCTAGGCTGCGTCTTGACCCCCTATCC
AATTCTCGGGCCAATCCCCCTTCCGTACTACCCAAAAATGAGATTCTTGCCGAATCTGAGTTTGCTGC
TCCAACCATTACCGAACCAATACCTATTCTGTCTAGTACTTTAGGTGCTTTCGTGGCGTATAATGTAA
ATCTCGTAGCGGATCAATTCCAACGAGCCTTTGAAACTAGTACTTCTGGTAATCGACTCCATTGCTCT
TTAAATAAACGCTGGTTCCCCGATCAAGTTTTTAATGACTTTATAGCCTGTCAGATCGTTCGTTTCGG
ATATGAAGTCTCATTCGAAGCTTCAGACAAAGGTGCTATTGAGATATTGGGCCCTTATGGTATTTCGT
ACACATTCCTACAATTGGCCAAGCGAATGAGTCAACTTCAAAGTGGATTCGTCGTGCGAGGAGTGCGT
TATAACCCGTGGCCGCCTGCCCCCCGATAAGTAGGGGGGCGGCTCCTCCGTTGTGGGTAAACGGTAAA
CCCGACTCTACGAACCCGAAGGGCAGCAGTAGTGGGGGCGTTAAGACCGGAGCTTTCTAGTAGTGCCA
GCAGGAATGCAAGTTAATGAATCCCTACAATAAGTTCCCTCCTTTCGTCCACGAGGCTGTAAGAATAG
AGATCTCTAAAGGGGCGGCTATCTAGCGGGAGTCGCGGCGGTAGTATGCCACGAGGTCCCTATGGACA
AGGGGACAAGTGAATCATCGCTTTTGGGCGCAGGCAGCCCTCTACCATCCCTCCCATTGCATTATATC
GTATTGTGCCGTATTAGACTATATATAATATATATATATATATATTAGGATACATGGATATATCGATG
GCCATGTCTATTAGATATATCTAGATTTATATATATTTATATATAAATTCCATATCTCAATATGTATC
AAGATAGATATATGTATCGTCATCATTATCGATTTCTATATTATTGCCATTATATATCTCGGATCGTC
ATTGTCCCTAGCCTAACTACATGTACGGTCAATAGTCTAGGGAGCGTAATTGCCAGAGCACGGGGGAG
AGAATGAATAGCAATGATTTCGGCAGGAGCAGCCGGACTGCTGCTATAGCAGTGCGAGCCTCTGGGAT
CTCCTGTAAACCTCCCCATGATGTGGCAAAGGGGGGGATATTGGGGGAAGCAGTGAGTGGATATTCCC
CTGCAGAGAGCCGGATGAGGGGGGACCTTCACGTCCGGTCCGGAGGGCGGGGATATCCCGACCCTACT
ATCACTATGCCTTTGCAATGCTACTTGGTTTAACTATATTTGTGACCTTTTCTCGTATGTGGGATTTT

ATATCTCCTTGGGTAGATAATCGATCGTCTTTCATTTCGATAGTGAGTAGTTTCTCCCCATAGGATTC
ATTATTTATTAAGTAAGGGGGGTAGAACTAAAGGAGCTTGCCAGGATGGGGCCGCCGCCCTGCCTGCT
GGGAGGGAGGGCGCCTCCATCCAGTAGTTTGTTCCTTTCTCTCCCTGATCCCGGTTCTTTCGAGAAGA
CTAAGGGATAGGCCCCCCGGACCCGACAAAACCTGACAAAAGCAGCTTAAAAGAAAGTGCCCGCCCGC
TCTCTGTTGGGTGACTTGGGACTCTTTGGGCTAACGGCCAACACGTGAATCGGCCGAGCCCTCCCTTC
TAGTATGTATGCTTCAGTTATCCGCCCGATGCATGGCGTGGATGAGAAACTTGTAGGAGGATAAAGCA
TGCTATTGGTGAGGCATGCTCTTGCCGAGCGCTCGATGGGAGATATGATTGGTAGATACGATCGATTT
TGTCACTTTTCTACACTATGCTTTTTCTATCATCGGTTAGCACAGAAACAAAAGCAACGAATGCGGGT
AGATCGGCGTGATCGGGGAATATATAATAGTGCCTTTTTTTATTCGAAACCTGTTTCTATTTGATAAA
GGGGTTATCATAGATAGATGAGGGACGGCCATCAACTTTGATCGAGCTTTACGGGGGAAGGAACCAAT
CGCTCACCTCCCCCCCCGCCCTTTCTCTGCCTGTTCGATTCCGTTTTCTGCTCGATTGATCTGATCAT
ATACGTTGCCTTTTCTGGTAAAACAGGATCATCGTAGCCTACTGGCATACGGTTTCCCTCGTGCGCGG
GAAAGGGATCTGGGGGGGTGTGTTTTATTTTCCCCCCACCTCTTCAAGTAAAGTATTTTCCAAGAGGC
AGCTCGGAGGATGGGATCTGCGCGGCTTTATATACTTTTATTTCTAGGAGCCTACCTACCATAGGGCG
GGTGTAGGTCGTGCTAAACTATATGAATACTAAGAACTATTGTCCGGGTGGGCTACCGGGCCATCGCG
TGAAACTAACCATGAAAGATAAATCCAAAGGCTAAACTGTATGCAATCTAAGTACAATCAGAGTTTAT
TTTTAAACATATAAAATCATTTATAAAGTTGGGCTGGCTTCCTATTCAAAGTCCCTACCTACTGAAAG
TCGGGCTCAATGGCGGGTAGGGAGGGTGGTGGGCTGGAGGTTGCCCAAATAATAGTAGGAAGCCCACC
TTCATAATGCCTGAAATCAGGTTATACCTAAATATGCTATGCGGACGTCTCGAACGAAGTGCCACCAT
CCAAATTTCGAATCAGTTTGAAACAGAGAGTACAGCCTCCATTGGGATTGGGTCAATTGGATTTGGTG
GCATCCTTCCTTATTCCATCCTTGTTCCCATCCTTATTCCTTACTGGCGCTCGTTGGAAGGAAAAAAA
GAGTCTTCTTTAGGGAAAGCCCAGGGAAGACCTTAGAACCGCTCTTATCAAGGTTCAAAGATTAGTGA
CCTTTCATAAGACCCTAGCTCTCGTCTTTATACCAACACTTGGGAGCAAGAGCGAGGAAGAATAAGTC
ATATAGCTTTCCTGGAGAGAAGGTCTTCCCGTGAAGTATATAAGTCTACCTCATAATATCTATAATCT
CATATATAATGAGGATAAGATCGATTGAGGACAGTGGCCTTTAGCTAACGTGGTTTCTCAACCGTGTG
GCGGTCTTCCCCGAGTGATCCTGACCTTAGAGAAAATCCTTTCATCGCCTTTAAGTCTCAGTCCTAAT
GTCTATCTAGAGTAGGAAAGAGGCAGTTCATGAGGCAAGCCCCTATTGAAAGACTTTGGTGGGCGTCT
CTATATGTGTAAAGCTCTAGTTTGTCATAATTTCTGAGCATAAACAGAAGCCATAAATTGTCGGTTTT
CCCATTTTTCCAGGGTCAATAACTAAATAAATAGGAGGAAAAACAGAAGGAGGGACAGAGAATGTCCC
TGCAGGAGAAAACTAAACTTGAGGTTGTAGGAGCCTCCTCTTACACCATCCAGTCATCCCCCTCCACC
TCTACCAAATAATCTAATTTACTCTCCCTTGCTATACTAGGAGTCCCCCCCAGCACACCTGGTGATGG
TTCCTCACAGACCTCCAGCCCTTTATCTTCCTGGTTTGGTACCATCAATCCCCTCTATTCGATTGAAC
CCCTTGCCCCGCTAAGATAAGAAAAGGAGAAGAGGAAAGCTTGGGAGAGCTCTATGGCTGCACAAGAA
CTTGCAGATCCCATTGCATGCCCTGGGCTATTTCCAAACTATGATGATGAGGGTGGCCTATTCACAAT
GACTACCTTAGATCCCTTACCCTCCAAGCCCTACTCTAAGATGAGAACTGAGGATTGGAAAGATGCCC
AAGTTATATTCCAAAATTATAAATTTCCCCCTTGCCCTCGGAAGGACAAGCGACTACGTTCCTTCAGA
CAACATTAGAATGGACAAGAGTAGCAAAACCATGGTATATTACAATTATTACCGAGCTACTCAAGAAA
ATGAGAGCCTAAAGCATAGTTCTAGTTCCCTGGATACAAAAAATAGGTATCTGACAGCAAGGGTAAAG
ACACTAGAGGCTGAAAAGGGGGTACTAGAGAGAAAACTTATTGTTGTAGGGAAAGATCCACAGGCAGC
TAGAGAAGATGTCTCCCTACTTAAGTAAAATAAAGGATAAATAGAGGCATATTTCATTTTCAAATAAC
CCCAAAACAATACTATTAAAAAGCACATTTGGAAGTAGTTGCCTCATGGAATGCATGGGAATTCATGT
GATTCCATTTTGATTTGCAATGCCCACTGCATGTAAGGAATGTGGCATTGGTTAATTATGATCCATCA
AAAGCAAAGTCTCAGGCAGATCGATTTGTTTATAAGGGCGGAGACTAGTGACTGAATCCCTTGAGACC
ACCCCACCGCACCTCATTCTATTTGATAGGATAGATTTCTTTTACTGGCGGGCTTTAGCATCTTCGGC
GTCTGCATTTATCATGAGAAGTTACCCTTAATAGGTGGTCTTTCCTTGCTATGCGACTAGGATGGCTA
TGTATGTGAAAAAGGTACATTTACGCCTGCTTCTCCTCATTGACGCCTACCCAAATAAATAATAGCCT
ACCACAATGCGCAATAAATAATAGCCTACCAAAAGCAACGTCTTCAGCAGCATTGTGAGTTCGCCTAC
AAAAAAAAGAACTCGTGCTATGAATCCTTCCTGTAAGGGCGGGCTTTACTGGATGGATATTTTTTATC
ACTTAAGAGTAGAACGAGCTTTCAGCCAGCAACTCTTCTCGGACAGGGTTGGTGATACCAGCCTTAGG
GGAGACAGGTTAATCAATCAATCGTTCGGTTGGTGATACCAGCCTTAGGTGAGACAGGATACTCGTAC
AGTGAGCTTCCAGCCAGCAACTCATATCACCCAAGAGTAGAACGAGGTAGGTCTTATCCCCCAAGAAC
GAGGTAGGAATGCCAGTGAGTAGCGGTAGGGATTATGGTATTGAATCCTTTAGTCCGTGGGCCTACAT
CGACTCTGTAGAGCTGGCGGAATGCCATGGCGAGGGAGGGCATAATGAAGGAAGGCAGGCAGAACTAC
TGCTGCTGAAAGTAAGGCAGGTGCTGCTGAAAGGAGGATGTTGCGCCAGAAACTACTGCTGCTGACAG
AACGAGGAAGAATTGCTCACCTGAAGAATGAAGGTGAGGAAGGATTGCTTGCTCTTAGGTAGGAGGGC
AAGGAAAGATAAGATCGAGTAAGTCGAGTAAGCCGCCCAGAACCAAAAAGTGGGTGGAAAGGGCCCGC
CTCCACTCCCTTCCCCCCCCGGAGGGATAGGCAGGCCTATCTATCTATACCTCCTGGGGGCCAACAGA

TACAGATGAAGCAGAAGTTTCCGTCTACGTCACTCCATCTTTCCCCAACTCCATATGAAATGTGCTCC
TTCGCATGACCAGAAGCCACTCCGGCTCAGCAGAGGGTTACGCTTTTTGAAAAAGCATCCGGTTCCAT
CTCTATTTCGTGGGTTAACTCACCTTTTTCCAGAATGGATTGTAGTAATGAAGGATCTATACTACTTA
AAATGGCTCTCTCATATCGGGAAATTCTGTCTAGTGGCATTCGATCACCAAACCCTTTTACAGCTGCA
TGAATAACTAGAATTTGTTTCTCAATAGGAAGTGGTGAATATTGCGGTTGTTTCATAACTTCAGTGAG
CCTAGCACCTCTATTTAATAGTGCCTGAGTAGCAGCATCAAGGTCTGACCCAAATTGAGCAAAGGCGG
CCACTTCACGGTATTGTGCCAATTCTAGTTTTAAACTACCACATACTTGTTTCATAGCTTTCAACTGA
GCGGCAGACCCGACGCGACTGACAGATAAGCCGACGTTAATAGCAGGTCTAATTCCGCGATAGAATAG
CTCTGTTTCCAGACAGATCTGTCCATCTGTAATGGAGATCACATTGGTAGGGATATAGGCCGACACGT
CTCCAGCTTGTGTTTCAATGACGGGTAACGCGGTCAAGCTACCCGCACCTGTCTGGTCCGACCGTTTA
GCGGCTCTTTCTAATGGACGGGAATGTAAATAGAAAACATCCCCTGGGAAAGCCTCACGGCCTGGTGG
TCGGCGTAGCAATAATGACATTTGTCGATATGCCACCGCCTGTTTACTAAGATCATCATAGATTATCA
ATGCGTGCATTCCATTATCGCGGAAATATTCTCCCATAGCACACCCGGAATATGGGGCCAGAAATTGC
AGAGGAGCAGGATCCGAAGCGGTGGCGGCTACGGCAATGGAATATTCCAAAGCATCTGCTTCCGAGAT
AATTTGAACCAATTGTGCCACGGTCGAGCGTTTCTGTCCAATCGCTACATAGACGCAATACAATTTAT
CACTATCAGCCTTTGACTTTGAGTTCATTCGCTTCTGGTTCGATATGATATCAATAGCTATAGCGGTT
TTTCCAGTTTGTCGGTCCCCGATTATCAGTTCTCGTTGACCACGGCCTATAGGGACCAGGCTATCCAC
CGCTTTTAACCCTGTTTGCATAGGTTCGTGCACAGATTTACGTTCAATAATACCTGGGGCTTTCGCTT
CGACACGTCTGAGTAAGTGCTCGCTTAAAGCCCCTTTACCATCAATAGGTACTCCCAACGCATCGACC
ACACGACCTAACATGGCCTTTCCCACAGGAACAGACACAATAGATCCAGTGCGCTTGACGAGAGATCC
TTCTTTGATGGCGGTATCACTACCAAATACCACGATACCTACATTCTCATTCTCCAGATTCAACGCTA
TTCCTTTCACACCGCTGGCAAATTCAACCATTTCCCCGGCTTGAATCTCGTTCAATCCATAGACACGT
GCAATACCATCTCCAACTGAGACCACTCGACCGATCTCATCAACTTGTAGATTGGTGCTATAGTTGGT
AATTCTCCTTTCTAATAGAGTAGTGAGTTCCGCAGCTCCAGGATCTAATTTCATACTTCAGGAACGAA
TGGATAGGGAGAAATCTACTTTACAAGATGATAAATAAAAGAGAATGGGGCCTGGAAAAGTTTGTCAC
GAAGTACCCCTTTAGGGCCTGCCTGACTGTAACGAACGAAAAGGTCGAGCGGCTCTACAACTCGCCCT
CGCCCTTTTTTTCTCTCCGGACGCCGGATCTTCCGGTCATTCAGATTATCTACGTAATTACGCAATCG
AAGAATTGATCAAATAATTGATCGAATTATAGCTATTATGTTCCAAACCTTTCGAATGCTTCAAATGC
AGTTATTCCAGTCATTCTTTATATATGTAATAACACGCTTTAGGCTCTCATAATCGATCGTACCCTAA
TAGAATAATATCGTATACAAAGATATATCTAATTATATATGTAATTACGAGCGTCATATGGGATGATT
TCTGCTCTCATAATTGATCGTATCCTATCTAGATATCCGTATACAAATAGAGATATCTAATTATATAT
GTAATTATGCTTTCTTATACTTGAAACGAAGTCTTTCACGAACGTTTAGTTAGGGTTCTTCACAACGC
GTACCTCGTACCTCACCCTTGATTCCTTCCCTCAATTGCTAATAAGGCCTTCCAGCCAGTGATCTTAC
CACCAGTACCTTTGAAGGGAGATCCACATCCTCGTCCTCCTGGTTAACGGAGGGGTGGAAGCTATACT
CTCTTAATCCGTAACTTGGCAATCCTTACCTTCCTTACTTGCTTGTTCGAGGTTTACCTTCAGGACCT
TACCTAAGACCATAACGAAGTATACGACTTCTTTTCTTGGTCCTTGTTCTTCAAATAGCTACGCCTAT
AACGCGAAGTATTTCTCCCCACTTGCTTCGCTGCGCACCTCGCCTTTTTAAGCCTGCACCTTCACGAA
AGAAAGAGAAAGAGTTGGAGACCCAACCTTCTACGTACGAAGTAATCACCAAAGCCTTAGCTTACCTA
CCCTGAAGCCATATCCCCTACCTTGCGATTCAAGCCAAAAGGATATGTTCTAAGTACTTGATTGAGTT
CCCTTCCTTAGGAAGATCAACACACGGTTGATAAGGTTATTCCAGAATAACTACTTTTTTCTTTATAA
GGGCAACGCAGGTCCAAGAACCAGGCTGGTCGAATCAACCCCGGCCGGCTGGCTAGGTTTCTGTATCA
GGGCGTGACCGGACGAAGGAATAAGCTATGTAGCGAGGTGAGTTTCAACGGATAGATATCTACCTGGC
CAGAAAGAGCAGACAGATGAGGGAGGAGGTCTTCGGGAGAGACAAGGAGATACTGCTCCATCTTTGGG
TTGGGTTATAGTGAAAGAACAATTGATTGATCAATTGTTGGACAACATATGTGGGAGTAGGCGGTACA
CCGGAAAAACAACAGCCAACAGATAGATATACCGAGGGGTGGTCCAAAAGACGAACGACAGCCAACGG
ATAGAGGAAGGGAGGGGGAGGGAGTCAACGGAACACGGCCGGCTTTGAGGCTTGACAAACTAACCCGA
CGGACGATGATGCTTCGCCTCACCGAATGCATTGACCTGCCTTGACATCCCACACTGGCACCCATTGT
GAAACAGTCTTGGCATTTACCCATTGCACCCATCACATGATACGAGAGAGATTGAGGGAACCCATCGA
ACCATGGAATGAGATAGATTTTGTTCCCCCGTGTTGTTGCCACGATATGAGATATTGAGGTCACCTTA
GTTGCCACCATACTATATTATTGAGTTCAAGTTGTCACGATACAAGATTGAGTTCCCAATTTTTCCAC
CATACGAGAGATTGAGGGAACCCAAGCTGCCACCATACGGGACACATCGACCCAACACGGCATGGATT
GGGCACGGAGCAAGCACTGGAATAACTGGGGCAATCACGGGAGACGCTTCGCTAGAAGCTGTTTACAG
AATCCGTTCACATCACAGGACACAGAAGCCATTCACACGGAGAAGGTCCACAGAAGCCATTCACACAC
AGAAACCATCCGTTCACAAACTGAACAGAAGCCATAAATAAACTGCATGCGCGAAGGCATCAATTAAC
TGCATGCACATAGCTGCCATGGTTTACGGCTCATAGTCCCGTCCGGGGTTCTCTCTCGTTTATGGATT
GAACGGGATCGAGTCTCACGTAGAGATCGAGTATAATATGTGAAAACGTATATATGTGTGTATCATAT
GTATAGACTAGCTTCTGGTGCGTTCACATTATCAATCTTGTTCAATAAACGAGAGATCAACATAAAGG

TACGAAGCCGCTTGTCCTTCCGAGGGTATGGGTAATCTGAATATGCAATCTGATTATGAATGCTACTC
CACTGCGCTTGGATACTACTCGGCTTGGCTACTCTACTCGGGCTACACATCTCTATACAATACTATCT
ACATCAATACATCTCTATTTTGATTGATTTATTTTGATTGATTGCCCACATATTACTAAAATAGGCCA
TTTTCAAAAGTTTCCCTTGGTGGGTATGCCCTTGCACCCAGGCCCACTTCCATAGCTGATAGGTTGGA
TTGGCTTGAATACTCGGATTGGATACCTTTTATAGAGAATCACCACAGTCGCCATTTTAATACTTTCG
CCTTTTTAGCGGGTCAGAGTAGGGACTTTGGGCTCCAAATTGCATTTGATTCTTACTGCACATTGCCT
TTTAAGATAGTTTACCTAGTATAGCCGAGCATACCCGATTTCCAGGTGGCGATCCCAAGAGGCAATGC
CATTCATATATGGATGGGGGGCTGTACGGTTCGGAACAACGCGATATGCGTTACTTACGATCACCCGT
CGAGTCATGCAACAGACAAGTGATAGTCTCCACCGGTAGTGCCAGTATTGGAAGGGAGGTAGATAATG
CTTGATAGTTATTGAGTGATCGATACTGAATGTCTTCTGAGAGATTTATCCGCTCGACCATTAGGGAG
AGGAGCGCACAAAAGGATGGGGGCCAACAGAATACAACTGCCATCTACAGGTGGGGTGGCATGGGGTG
GAACTACGGACGGATCAATACAGAAGACCCAATCAAGTGTGTGAGTCAGGTGCGCAGATACCAATTGG
AGTCTTACACAGATACTGGCTAAGAAGATCAGTCTGTCCGCATTCAACCATTACCTAAACTTATCCCC
TTTCACTTGCCCTGCTTGATTTAGATTAGGGTGGTGTAATTATGACGCTACTCCATTGCCAGTATTAG
AAGCCAAGTAGTTGATACCCAGCATGCGAGGTTGATGCTTTATGATACCCCTACTTGATACATCTCCA
TACTGCGGTAGAGGGGCGGGAATGATACTGGGCCTTCCATTCATTCCAGGGTGGGTTCGTTCTTCTCG
TCGTGAGTTGGGAGGTCTTGACCGACGATTGAGGCCTGGAACACATGAGAGAGGTCTTTACACGTACG
CTTTCAGTTAACAATTTCATTACGTTATGTATGCTTTCAGTTCCACCATTTGGCCTTTCCCAATCCTC
TATTACGCTTTCTGGAACCAATCCTCAATCAAAATACCGCACGCATTACACATTCTCCCATTACCCAG
TATACCGCATCCACGCATACATACATGCCCCGCATCCGCGCATTCCCCAGCAGTCCGCCGTTCAGTAC
GCCCAGGACACGTCAACCAGGATAGCACAGTATGATCTACCCGCTTACCATCCATGCTCACCATCCAC
GCCATTCTCCCTTTATTGCATTACCGCGCTCCCCCATTCCCATTAACGCATTCAGTACGCCTGCCCAA
TCCTCGTCACCCAGGATCGCCCAGTACGCGTAAGGAAGGAGGATGATAACGCCCAGGACACATATTGG
CGAAAAAGGAAGGAACAGTAAGTGGATTGAATTTGATACCGGCGGGACTAGGGATGCCGAGGTATAAA
GGTCTTCCAAATGAGCGGGCCTACGAACGGATCCTTACTGAAAACCGAGAATGTTTGTGAGGTGCGTG
GTCACCGATGATCAGTAGTAATTGATTTGATTCATGGCATAGAAGATAGTAGCTGGTTGGGGTGGGCC
CGGTGATCGAGTTATTCGATTGATTGATTATATTAGTCCCTATTCGCCCGCATTAATGCATTCTCCCA
ATTAAACCCATTCTCCCGCATTTAAAAGAAGTATCAGGAGGCAAAACAAGATATAAGCATTTAAAACC
TTTGCTTAGTAGAACAGCAGCTGTTGAAATAGCCCTGGTGAATGAGCCCCGGTTAAAGATGAGCCCTG
GTTGAAAGAAAGAGCCTGGTGAGAAAGAGCCCTGGTGAGAATCGAAAGCTAGCTTGTTAGCTGTCGGT
GAGCTATTCAGAGGGAGTTTTTTTCATAAGTTGAAGGCAGAGCAGTTAGAAGTTGAGGTGGATACCGG
AAGAAATATATTCTATGCACACTTTAGTGCTCTGGAAACAATATTTTGAAACCTTTTATGATACTTTC
AACTGAACTGAGAAAGCTGCTCCGGCTCTTGCCTGTGCTTGAGTGGGGAATTCCAGCTATTCTTCTCT
TGCTTGTTTGCGCGGAATGTTTGGCGTAGCTTCGATCATCGTTCGTATTGGGGTGTATCTTGGTCACC
TCGGCCATCTCTCTCTTTCCCTCGGGGGTGGCTGCTGCGCTACTCGGCTTAGCTCCCAGGCTGCTGGG
CTGGGCTCATCGTGGGCTGGGCTGGAAACTTACATAGACGTAGATAAGGGTCCATCATAGTCGAGCAT
AGTTGATCATAGCCAGCTGGTCATAGCCAACCATTGCTGCCAGAAGGTTATTGCCGATAGCCGATAGT
CATTGCTTGAAGGGGCCATACAGCTAGGCATTGACAGTCTCTCATAGACTTTAGGTCATTTATAGGTC
TATGGTTGGATAATATCATATATAATATAAGTTACCAAACCTATACCTTAAGATGCCATAAGCAGCGG
ATCCACTCGAATAAGTCCCCTTAAGGAAGAGGGGAAACCGGAAAGAAGGCAAGGTTCCTCATCTGAAA
GGGAGAGGCAGATAGAGCCCCTTGACCCTACGGATGGATAAAGAGACCTTACCTAGCCCCCGGAGATG
GATAAGTAAAAACTGATAACATTTTTTTGTAAGGTTGGAAAATATACCGGAGTTGTATTTCAATTCCA
AATAAATCATCTTGGAAAGATAGATATTAAAGAATCACTTAAAGTATCTTCCAATGGTTATCATTTTC
TGGTCGAAGCAAATGTGGTTGAATATCACATCACGCGAAATGTTGTTTCATTCTTGTTTCAATTTACG
TATAAATAATAATGGGGGGTGTTTGTTGTTACGGGGCTGAAGAGTCGAAGGGCCTTACTTCCTTCTGT
GGGGCTACAGGGCTATCTGAGGTACCCTCCCCGAAAAACCCAAGTAAGGGCCATATTGGGTGGGGGAA
TACACAAACCACTACGGTGTTGGAATCGCCGATATCAAGGTGGCGCATGCGTGCGTGGAACATGTGAT
CATAGTGTTATTGAGTACTAGATATCCGGTATAGGGAATCGTTTTTACTCGAACGGGGTTATTCGGTT
CACCCGAGTTCATATAAAGCGTTAGTAAGAGGAAGGATGGCTCACCCGACCTAAGAAGGGTGAGGAAG
GCATTTTGGGCAATTTGATGACCTTTTATCCAATTTAAGCAAGGAAATTGAAGGGAAGGGTCAAAGCA
TTGGTATATGGTAATTGGTGTATAGTATTCGGTATATAAACTATCACATGCCGATCTCCCTGGAGTGC
CCATGGGAGTAGGTATCGAAACCAATTACAGTGGAAACAACACACGATTGAGCAAAGAATAAGTTCGG
GACAAGCCTTCAGACCGATCAACAAGCTCAACCACCTTACTATCAAGACCCTATAGAGTCAAACCTAT
ACCAGACCTCTCACACCCTAGATTGAACATAGAACTATGGATCAACTAAGGAGCAAAGGAACCGGAAG
AGAACACCACACATCACACCCACGAAGGATGCACGCCGCTACGTTGGATCATCACACATTTAACCTCC
ATTACCGTAGGCATCGAACATTAACCAGTGGAAGTAAGAAACGGATGGAACCCGATATGAATTCAGAT
TTCGGGACAGAGAAAAGGAAGAGCGAGGATCACACCTATCCTACGCACGCTTATGAGGCCTTATAATC

TTCTTTCCACGTAAGCCAACGCTGGATGATTTGAAACGCAGGGAGGTATCGCACAGTAGAATATACCC
CAAGAAGGATACACGCTGCAGGAACTAGAACTATTATAACTTCAGCTATAGGGGACAAGATTGAGCCG
CTTTATACCTCTATAGGGAATAGGATCACCAGGTAGGAGGATTACTTTATGATTGTCGCTATCGATCT
TACTCCCGCCGCACACACTATTACTCCCACAGCAGTGGCGCTAACTCTTGCCAAGGTCGCTCTCTTTC
ATACTACCATAGTATTTCTTATTATAGCTGCTAATGCTTTTACTTTGGCTGATGGCTTTCGGATTATG
GAGGACGCTGGACAGATTCTTGCTTATGGGATCCGGATTATTAAAACTTATGTATGGATTATGCCTGC
TGCCGTTCCGATTCTAATAATTGGTTCTTTTCACGCAATCAAATAATTATAGGTTATTAACTTTGATG
AGCTGTAGTGGTCTCATCTCAGAGTTGCAGGTTCGAATCCTGCTTGCTCCAGCCGTAAGGTACTAAAA
GCTGTGAGATGCATCGTCATATAGAGGATAACCCCATAGCTTAGAGATGCATCATACGTATTCCCTTT
CAATATTTAGTTGATTGGACCAAGAATCGATAGATAACTGAATGGCTAATTGATGACTGAATGCTTGT
GTAAGCTCTCGGTGTGAAGGTGATACCAAGCGCTTGGAACTGGAGACCACTCTTTAGGAAAGATCCAG
ATGCATCGGTTTGCTGCGGCGTAATTGCCATTTTATTACCCCGATCTACTGGATTGAGTAATTCTGAG
TGGGCTCACGTATTCATAGTGGTTTCGGTTCATCTGATCGCCTGAGCTACTATGCTCTTATGGCTAGA
AATAACAAATCTGAGATTGCCCCTTTGACTGGAGGACCGTATAAATCACGGTTGGATGCATCGGTCCG
CTGCGGCTATTTGCCATTTTATTACCCCGATCTACCCATCTCAGTACTTCTAATCGTTACCTTCATTC
ACGGATCTGCTCTGGTTCCTTTCTAGTGCAGGGAGAAGAAGAACCCCATAAAGTCGTGGGTAGCAGCG
GATTTGTTAGGGAGGAACTAATGTAGGTTGTCAAAGTTATCACCATCGCATCTCTATGCTCCTTCCTT
TTGTGCCCATCCAGCAGGTAGTGGACTCGGATCAGTCTTTCCAACCACTATATATCAAGCTAGGCTAA
CCTTGTGCCCTCTGTCATTCCACGTTCTGATTCGACCTAACTCTGGCCAATTACAAGAGAGCGCTCTG
GAACATCGATTATGAACACCAACACTATGTGACACCCCGAGGATGGACTACCGTAAGACAGCAGGAGA
CGCCCCTTGCTTGATGGACCATCTTGAGAAAACGCAGCACGGGCCAGATCCATCGAAAGCACCATGCC
AGCCGGATGCTTCCATAGCCCTTGATTGAAGGAAAGAATCACTTCACGAACCATCACTTCACGGACCA
TCACTTCACGGACCAGGAAAGACTATAAATAAGTCTATTGGAAGGTCATCTATTGGAAGGCCATCTCA
ACTAATCATCGCATCCTCATTTTGATGAGATTTACTTAGTAACATGATCCAGTAATTAAGTTCCACCC
ATAGCAGTAGTTTAACAGCGTTGGAAACATATAAAGTTTCAGTCCCATAACCAATAGTAGCGCCCTTT
CCCTCTCGAGAGCCACCAAGTGTGGAGTCCCTAGTCCCAAGCGCGGATACATATCCAGGCACGGTTCG
AGAGTCAGTCCTATAGCCAATAGCAGCAAATCCGGTTCTATTTGATCCAATTGCCCGTAAACCAGTCC
CTTAACTAATAGTTTTAAAAGCATATTTAAGCAAGTGTGATTGCAGGCCTGTGAATACTGTATGGAAG
CCTGTCCATCCAAGCCAGGTTGTTCGCCACCTACAACATGAGTTGTGTAGGCGCGGAGCGGTAATACA
ACTGATCGGCAAGTGCCCACGCAAGCCTTTTCTCAATCTCTGCTCTGCTGGGATTCCCCAATTCAAGA
AGGTAGTTCCGACGCCACGATTAAACTAGCTGCCCAACAGAAAGAAATCATCAAGATTCAGAGTATAA
TGGAGAAATAACGTCTATAAAGGCCCAATATGACCAGTTGAGGTGCGGAGCGAAGATCCCCATATGAA
ACAGGTACCAGGTTGGAGGGTGGCACCAATAAGTTAGTTGGGAAAGGTGGGTGCCCTTGGACGCTCCA
TAAAAAAACAAAATGTGCTGCTTCCTATCCCGTCCTGCTGATGCTGATAAGAGGTTCATAGTTGTACC
TTTTAAGCGCTTCATTCATCCAAGTATGGGGGATATAGTTCGTAAACACTTATTCAGTAAGGGAGAAG
GTAAAGGAAGGGGTTCATGCCTGCTATGCTATGCCTTCCAGCCATATTGATCATGGGAGCAAACTCGT
TGTCGAATCAACTTTCCAGCCAGTCGTTTCTCCTCTTCCATTCTGCGAGAATCCTCCAATCGGGCAGT
TCACGACGTGCTGAAGTTCTGGTGAGAATCAATCAATCCAACAGTTCTGCCGTTTATCTGAAGTTCTA
GCCGTGTCTCATCAGTTCTGGCTGAATCCATAAGTTCCCACGTCACAATCCAGCGGCTACTGGTTGTG
GTGCCACTTCCAAAAGCAATAAGCATCTAAAGTTCCTAGGAATGCTGTCTGTGTTCATTAATCAATCA
TATCTGGAGGTTAAAACCTAGCTGTCCAACCAACAGAGTATTCTTATAGGCAGGCTTCTTCAAAGGGT
AATAGGCAATGGTCTAAGTAACTCCCACCCATGCCATCCATGTGCTGACCCCGATTCGTTCGATATCT
AATAAGTTTTGGCTGAATCACTAGGGTAGTTAGTTCTCCTTCCTCATGCCACGACGTACGATCCAACT
GCTACTAGTTGTTCCTGTGCCACTTCCATTCCCTTCCATTCCTTGTGTAAGCATCTAAAGGTCCTTCC
TTCCTATCCTAGCAAGTGGTTTCAAATGACTGACTCGTCTCACTTCGTTCCTCGCCCTCCTTCTTAGG
TCGGGCGATAACTTAACGTTCCTTGCTCCTCGGCGGGAACATGGTACCAACAAGAAGGTAATACGGGG
TACCCCAGAATATTATATAGCAAGTTCATAAGGTAAAGGGAGGGTTATTAGGGTAAAGGGAAATATCC
TAGTGCCAACTCTACAGAGCATTCCTGGCTTCAGCAATAGGCAGCATCACTCCATAGGTTGTTGTTGG
GTGAAGGGAGTAATAAAGTAATAAGGTTGGATCATCCCATCTGGTGCTTATAAGAAAAGTGAGCAGTA
TGTACCTTCCTCCTAAGCGATTAATGAAGTTCGTGGTATAGGGACGGGTCTATTTATTCAGGTGGAGT
AAATAGTTAATAGCACTCTGTGCCTCGTCTTTCTGGCTTCAGCAATGACTGCATCTCCTAGGTTGAAG
GCAGCAGTAAGTAAAAGTCTGGTCTAACACTAACAGGTAACATGCACTTCAGTAAGGTAAGGGGTGTT
GTAAGGTGGGATAGCTGCTACGAGGATGTGAGGGGCCAAGTATACATTCTAAAGCGGGTTCCGGGCTC
CGCAGGACACCACCCATGTTGTTTGTTCGTTTGCTCGAAATTACGTATATAAATAAAGCCCCAGAGCT
AAAGCAGTGAGAATCGCAGAGAAGATCCTAATTTGAAATAGACACCAGGGTGGGACACACGAATGGTC
GATCCAAAAAGCTTCGCCAAACGCGCGCGTATTTAAGGTGGTTAAGACACGAACATGAAAGCATATCC
TGGGGCAATGCTAGCTGTAGAAATTCATCACTAGCATCTGGGGTTTAAGCCCGTCCCCCCAAGAATCT

AAATAGTGAGACCCCTCTCCCTCTAAGCATTCATTCAGATTTAGCAAGTGGTTGCTTTGGAAGCCCGC
CCGTTAATATAAAAGTTCAGATCTGTGATCCTATGCAAGGGTAAATCATACAGGTAGGTTGCTGGTAG
CACTAAAATGAAATGTGTAGAGATCTTCCTAGCCAGTGGGTTCTAAGGAATCAATCGTATCCTGAGAC
TCGGCCAGAATAGGTAAGGTACCAGCATCTGTAAGCAAATGTGAGTCCAGTAAATACTTGGAAGGGGA
AAGCTAGGTCTATAGTTGGTGAAGCTGTTTGGGGCATGACAAAGTTAAGTTATAAGGTTAAGCAGGTG
GGTAGTTTATAAGAGTAAAGGTAAATAGGGAGGTGTATACCTAGCAACTAAGTAAGGTATTCCCGGCT
GGAGCAAGAGGTGAGGGTATCCAAAGGCGGGGTAGGTGAAGTGAGGGACGCAGCGGAAATAATATCCA
TATAGGTGTTTTCAGACAAGTCTGTCCGTCCCCATATGAAGCTAAAGGTTAGGTGCTTGCTAAAACTA
GCCTAATCTTCCATATAAGATAAGTGGTTATCTTAAATAAGCGTTCTATGCTACATAAGAGCATTCCA
GGAAGAGCTACTTTATCAGCAGTCTATTTTTTGCTACATAAGAGCTTTGGAGATTGATTGATTGCCAA
CATCATTTTAGGGGAAGGGAACACTCCTTTACTTAACCGGGTCACCTACTCAATTCAAACCGTTCAAT
TCAAACCGTATCTATTACACCCCAAGGGAAATTCCTCAAGTGTATAGAGCTCAGTACTTAAACTAGAG
AGAGTTGAGGGAACGTTCAATTCAAACCGTTCAATTCAAACCGTTCAATTCAAACCGTTCAATTCAAA
ACGTTCAATTCAATTTGAGACGTGCCTTGATAAACTCCCTAAAGTCAAGCAATCAAGATCCTTTACTT
AAACTAGTCAGGCCCCTCCTTCTTAGGTCGATTAATTGATTGCCAACATCATTTTGGGAAGGGAACAC
TCAATTTCACCTAAGCTTTCCCACCAGACCTAGTTTATTACCATAAGTATCTAAGAGCAATATACAGA
TGTACCATAAGCAAAGGCCTAAGGCAAAGGCATCGAACCGGTAGCTAAGGTAGTACCATAAGCAAAGG
CAAGGGAAGCACCAAAGGAGAGGGCATAGCCAGTTTCTTTCGAGAGAGCAGTTGAGTTCAGTTCCGCT
CCAGATGAAGATCCAGATCGTGATCGAGACCTAGTTCCTACCGATGATCTACAGCGTGATCCAAGCCC
TCGTGAGCGAGATCCATATCCTATAGAAAGCAAGACCCAGTCACAGAAGCACCCGCAGCATCTCGCCC
AGCATATCCATTTCCAGGCCCAGTAAAAGCTAGGGCATCACCAGTCTAATCAGCTTCATTCCCAGCAT
CATTATTATTCTCAGCTGCACCAGTTGAAAGTGTCAGGGGCAGCCCATAAGCATCGGTTGAACCATTC
CCGGCAGCAGTCTAATCAGTATTCCCTCAGTTGAAGGATTTCCAGAAATCAGTATTCCCAGGCCTAGC
AGCAGCACTACTCTCAGCAGTCAGCATCAGCATAAGCGAAGCAGAAGGACAGCTCCATCAGCACCAGT
TGAAAGAGCATAATTCCAAGTTTAAGAAGCACGAAATGAACTAGCCTCATTCCTGGCAGCACCAGTTT
GATCAGCTTGCAGCCCCAGCAGCGAAAGGAGATAATTCTTCCCGGCAGCAGCAAAAGTGGTCCCAGCA
GCATCTCCATAAGCAGTTTAAGGAGCATAATAACTATAGTTTCTAGATTTTGAAATAGAATCCCTGGG
AACTAAGTAATACCCTTACTCTACCAGCTGAGTAAGCCCTTTCTTTCAAGGTCAAAGGAACTTGAACC
AAACCTTGTCCCTTATAAACCTAGGTAGGAGATGGAGTACCAACCCTTTGATGAAGCCAGGAATACTC
AAGTAGGCAGGTATTGCATGCTATGAACTACCCCCCTTGGTTTATGCAAGAACTAGACCTTTAGCCCT
AGAACACTTGAGGAATTAATGCACTTAGTAAAGAAGGTTAAGTGAGTCGGGACTGCGTTGTTTTATGC
CATTCCGAGTTGATAGTGGTACCTCCTAAGGTCTTTCTTCATAAAGTCAAGTGAAACATGCCTAGCTC
TGAAGTCTGAAGGTGTCTAGGTTGTTGGTATGTCCAGGCAGGTAAATGCGTATGGTCGGTTAGCTCTG
GTCTCGCGAAGCATAATCTCGTAAGTAAAGCGAAGCAGATAAATCTCGTCGGGTAGTTTTGTCTCGTC
ACACGTCATTCTCGCCACACGTTCTAGCGTCACTTCGTGCCTCGCCTCACGTTCTAGCGTCACGGGTT
GGTCTAGGGTCAAAGCAAGCAAGCTCCGTCAAGTTAATCATCGTCAGGTGGCAAGAAGAGAGAGAAAC
GGGTATAAATCGAGAATATCCTAGGCAGGTATCGAGAGAGAAGATCTCAGGCAGGGATATAGAATATC
TCTAAGCAGGTATCAAGATCGATACGTACAGGTATAAAGATCAAAGATCTCAGGTCGAAGGTTCGTTG
CTCTTGTTGAGCAAAGCAGAATCGGTTTGTTTGTCGAGCGAAGCATAATCGTCACGGGTGGTTCTATC
CACAGGTTCTAGCGTCACGTTCTAGCCTCATGGGTTGTCTCGTCTTCTCGCCACACGGTCTCGCGTCA
CGGAATCGTCTAGCCTCCGGTTGTATCGTCCGTCGTCAGGCAAACAAACCGACCACCGTTTTACCTTC
GACTTGATCGAGACCTCGATTGAGACCCCCCATCTCGATTGCTACCACCCGGATCTCCATTACCCGGA
TCTCTCAACTTGACATATTTGATCTTTATACCTCACGACAGTTGCCACGGGTCTGCTTGCTTGAAGGA
GGTTGGCCGGCTGGCTGGCTTGACAAAACCGTGATGCGAGAACGTGGAGGTAGTGACTAGACTTTAGG
CTTGATGAATCAACTATATGTGCCCGGGACGGGGTGGGATAATGAAGGAGGAGCCAACCGATCTAGGA
GATCTTTACCATCCCACCAAGAGCTACATGAGTCATCCTACCAAAATGCTGATACCACATCGCATTAT
ATACGTTGCTACACCCGGAGCTTCCATTTAGCTTCTATTTACTTTTTTAATTGATAGATGTTACTCAA
CCGAATCTCTTATAGTTGCATTTGGTAAATTGATTCTAGTTGCTTTTGTGTCTTGATCGCTAGATCGG
TCACACAAACTATATCACCCAGCACACCCAGTCATCAACCCACCATATGCCAGCAGATATAATGTTCA
CCAAGTATACACCCCATACGAGGAGAGGGGTTCATGTTGTTACCAGACAAGCATAGCGATTGAGCCAG
GGTGGCAACTAGAACGAAGGACTTACCACCAGATGAGAGAGATGTTACCGTCAGACCAGAGGAAAGTA
CTTACCGACAGACCAGAGAGAGGGTCACCATCACAACATACCCGACCGAGAGATAAGTTACCCATCAA
TGGAGAGAGAAGGAAGGAGCACATCACTGGCAAAGAGTATAAATACCCCATGTTCACAACAGATGAGA
GAATAGATGTACCCCAAGTTTATCGCCATACAGGATTGAGTTCATCATCACTGGTCGAGAGGAGAAGT
CCCCCAAGTTCCCACATAAGTCACCTTCACCACAGACCCCGTTCACCTTCAACCCATACCCGAGAGGA
GAGAGAAGGAGTCCTGAGAAGGAAGTTCAAGAGCCTGTAACCTGGAAGGAAGGAGCCTTTAGCCTTCA
CAGCGCTTCAAAGGGCATTTGCTTGCGTTCTATACTTAGCCATCACTGCCGGTCATTGAAGAGGAAGG

ATAAGTTCTTTTAGGCTTAAGGGCCCCGGGGAGCTACGATGCCGATGCAACCACCAAAGGGAGATAGT
AACCCTAAAGAAAGAAGATGAAACTCCATCAACACAGTTGGCTTTACCCATGGTTTAGATATTAAAAT
GGTGCTTAAACCACTGCTACTGGTGGTGCTGGATAAGCTGCAGGATCAACGACTGGATCGCTGATAAA
ACTGGGGCCTACTTTCAACGCTTCTCCTGCGACTGCCTTAGCTGCAGGGCAAGGAGTTGTATGTAATA
GTCCGTGCCAATTAGTCAATCATAGTCGAGAGCCAGTAATCCAGGTGTAGATGCCGGTACTCAATACT
GGTGTTGATCCACCAGATGACGGTACTCTCATGTCAAGTTTCAATGCCAGGTTTCAATGACCCCAGGT
TTCCATCACTCGATGCCAAGAGTCAATGCACTCGAGTCGATCCCAAGAGTTGATGCCAGGTGGTTATC
ACTCGATGCCGGTAGGAGATCATCCTGCTGGTAGGATTCCAGGGTTCAGTCCAGTGGTCGCTGAGTGG
AAGGACAAGTTGATGTCAAAGAAGCAAGTTGAAGTCCAAGTTGATCGCTTTATATACTTCACCCCAGC
AGGACTAAGGAAGCCAGGGTTAGACAAGGTGGCTGGGAAGACAGGGTTGGATGGTTGGAGTGGTGCCT
ACCAGACCTATAGGGTTGATCGTGAACAGCCTGCTGGATTATCTCTATCTACTGCTAGTTAGGAGAGC
ATGACTTGTGATAGACATAACGCAAAGGAAGACCTTACCTTATTCCACCCCAAGAGGAAATTCCTCAA
GTATATAGAGCTCAGTTCTACTATACCTTATTCCACCCCAAGAGGAAATTCCTCAAGTATATAGAGCT
CAGTTCTACTATTTATGATTACCTTTATAGGGATTTATCCTCGCATCGGGGAAGGTGCGTAGCGATGA
AGGCAGGTTGGAAGGCTGCAAGGTCGAGGAGGGAGAGAGAGAAGCTTCAACTCAGCAGCAGGGAGAAA
ATCTGTATTTATTAGATGCCAATACCACTTAAGGTTCTGGGCCTCTCAATGGGGAACTTATAGGTGGA
CTAAGGCCCAGTAAAAGCAAGGGCATCACCCTTAGTTGTGGGTGAGGCCGGGCCAAGAGATATATGGC
TAGCAGAATGCTGAGGTACCGCTGATGCAGGGCTTGATCGGACCCTGAAGCCCTGACCCACGTTGAGG
TGGTCAATATGTTGAGGCTCTCATAAGGTATCTAGGGCGCCTCAACTAAGGTCAGGGCTCCTCAGAAA
AAGCAAGCCAGCCTGACAGAAAGCCAGACCTGTCTCTATTCCACCCAAAGATAAATTCCTCGAGTATA
TAGAGCTCAGTTCTTCTATTAGGTTACCTTAAAAGAGGGACGCTAACGCTTTCAGTCTAATTCAAAAA
AGATCCTTACTGAAAGGATCTTGATTGAATGAGACTGAAAGGCTTATCCTATAAGGCATCCAGTTCCT
TAATTGATTGAATGAGACAGTGAGGGTATTTATTGAATGAGACTTCAAGGCTTATAGAGGAGTATAAG
GCTTATAGTTCATAAACGCTACCCACTTCGACACTTCGAGGAGACAAGCGAAGTGAGTGAATAGAGTT
AGTAATGAAGTTTAGTGCGAAAGAAAAGCGTAATTACGTATATAATGAGGGAAGGTGCTGGTTAGAGT
GATGCTGCTTACTTGCCTTAGCCCGGCACTTCATTGACTTTGCTCCCTTTGCTACTGACTCAGTCAAT
CAGTCTCAGTCAATCAGTCTTAGTCCCAGTCGTTCAAAGGGAATAAAGCTAGGCAATATAGGCGATAT
AGATAGGCATCAAGCACACGTGCAAAGCGGAAAAGCTATGACTCGCTTACACTCAAACATCTCATTAT
ATATGTAATTACGCTATGAATTTATTGAACGGAGTATACTTGCCTTCTTTCTTTCTATCCGGAAAGAG
GTCCTATACTTGCTTTCTCTTGCCTTGGAAACCCGACCTAAAAGAGGGATTTATTGCTCACCTGACAG
AGGAAGGAAAACGTTATCGCCAGACGAACGAAAGACAGGTTCCAGACAGAAAGAGGTTGAGCCAAGTT
GCCACCATACGCGGGAGAGAAGTTTCCCTTCACACTATAACCGAGAGAGGATAAGTCCCCCATTTTTC
CATGGAATGAGATTGAGTTAGTTCCCCCGTGTTTACACGATACGAGATAGATTATGTTACCCCAAGTT
CCCACATACCGGAATCACCTTCACCACATGTAACAGACACCCAACATAGTACGGGGGCCGAGTGAGCC
ACAGCCTTAGGGTATCTAATAGCTGATCCTTTACAGTCATTGCCTTAGGATCTTTGCCAAAGGGTCAT
TGCCAGAGATAGGGTGGTCACCGATAGAAGCCAGTTTTCATTGCCGGGAGGTCAGTCATTAGGAGGTC
ATTGCCTGGTGGTCATAGCCAACCAATCATAGCCAGTGCTCATAGTCAGGTCATTGTATTGATAGCCA
ATCATTGTAAGCCGATAGCCCATACCCACCGGTCTATGCCAGTCAGCCCATAGGAAATCATTCCACCA
TTGAACCTCATTGCCGTAGATCATTGCCGAGATAGAGCTGGTAGTACTTGCCTGGGTTAGTCGAGACC
AATTTACTTGTTGAGCCCAATTACCCTCTTGCTACCAATTGCCTTCTTGAGACCAATTACTTGAGACC
GATTAACCTGTTGCTCCCAATTCACTTGTTTAGGACAATTAACTAGTTGTAACCAATTGCCTTCTTGA
TGCCGATTGCCTTCTTGGGAACAATTACCTTCTTGATCCCGATTCCAATAGCCCAATTACTACCCATT
CCTAGTTGAGCCCCAGTCCCATAGCCCCAGTACCTTGTTGTTACCGATTCCGATAGCACGAGTCAATA
TACAATTATCCTTGTTGTTACCAATTCCTTTAGCTCCAGTAACTATCCTACGGGATGCACAGGGTTCA
GCTATTTACACCCCCCCGGTCACTGAACGATACGGAACATGAGGTCCTACCTGCACTTAGTGATACGG
CTACTACGGCTACGGCAGCAACGGCAACGGCACACAGATGGGTACGGTTACAGCACGGCACTAGATAT
AAACTACAACCCCGTATACGCATAGCCCATGCCAACCGGGAAACAGTCAGCCGATAGCCCAGCAGTCA
CAGGAGGTACCAATTTACACTAGCCAATACATGGGAGAACCTCATTAAACTCCAGACCAGTCAGTTGA
CACAGGGAGGAAGGCACCTAATCAAGGGGGATACTCAATGGCGTTGGCAAGGGCAAAGAACGATACAA
CCTACGCGATGGAAAGCTTATCGATACAAAACGGAGGTACTACAGCTACTCAATACTCTTTCACTTAC
CGAGACGGAACACACGCTCCTACCTGGCTGGACCGCATTCACACATTGACACATTTCCATTACCGCAT
TACCGCATTACACATTCACGCATTCCGTTCAATCCGCTCCCAGTCTCCCAGCATTCTACCAGCATTCT
ACCATTCCCCATTCAATCCGCATTCTCCCAGTCTCGTATAACAGTAGACCATTCTCCCATTCACGCCC
GCAGCCTCCCATCCCCACATTATCCCAGTCTCCCAGCATTCTCTCATTCTACCATTACCGCCACGCAT
CCCCACACGTCACCCAAGACCCAGTACACGTAAGGAAGAAGGAAGGATACCGCCCAGGCCACGTATAC
CAAGCAACGCGCCACACAGTACACGTCTTACAAGTGTGCCATAAACCAACCTATATCCTCAACCCTAT
ACCGAATACGTTAAGTCTATATACGTCGAAACATTACCCATCCCGAGTGTGTCAGGTCACCGATTAGG

AGTTATTTATTAGGAGTTATTGATCAGATTGATTGCCAACATGAGCGGATACGGATACTACGGATACT
ACTACGGCAACGGATACGGCAACAGATACCGATACTGACACACACAATGTTATATACGTATATATACA
GATTACAGAATAGCGTAGAAAATACAATAGCTAAAGAACAGGCGCGCTTACTTACGTGCCTACTGGCT
AGATTTTTGTATGTTTGGGTTAAAGCAGGCAGGCGTATTTCCGATCTTGATCCTGACCCTGTTGATTC
AGAATTCAAATAATGCACTTTAAAGATCTAATACTGATCCTCCAACAGCTTATATAGAATCCCCATAT
GCATATATTTTAAAAGGCCATATTTGCACGTTGCCGACCCATTTGATGCGGGTTCTGCCTCCCTCTCC
TCCCTCATACCCATTCGGCTGACCCAGTGATCCTGCCATCCAGTCAAGGAAATACATCAGGGCAGGTA
TAAAAATCGAAGATATAAGGTATATAAAGATAAAAGAGAGAAGGCGGGTAGTGAAACTGCTTTTGGTT
GTTACTGTGTCCCAAGAACTTGTTGTGCTGTCTTAGTGTATCCCCTGCCGGCCTTGGTGGCTTTGGTG
TTCTAGGCTTTTTACTTCTGGTGGGCTTGGCTAAAGGCTTCTGGCTAAAGGCTTCTAGCTTCCCTCCC
TGGTGTGTTTGTGACTCCGGTATAGTGACTCATCCAACTCCACCTCCTCATCGGGATACAAATAAATT
GATAAATAGTGCACCGGGCTCCCCCGATTGGGGGTATAAAATGAAAGGCAGAAGCGAAGCGTCCCGAG
TTCATTGTGCCGGTTGGCTATGGGGATAAATACAACTCACCCAGAAAGAAGGAAACTAGTCCCCCTTC
TCCCAGCTGGGCTCTATCTTCCCCCTCCTATATGGCCCTACCTTCCTATTCGGCATCTTGTTGCCCTG
GCTTGTCGTTCCTTTCACTTCTGTCTGTATTTGTTGTGTTCTTCGTGGGGGTGGATGGCAATGCATGA
GTGTTTTGTGCTTGTGCAATGGGTCCGGGAATTCGGGGGGTATCCATCTAGTTTTCTCCAGTTTTATC
TGGTATGAGCAAGGCCCCACCAAGAGGATGGGAAGGAGACAGACAGAAAGACAGGGCCCATGGAATCC
GGATTTACGAATTGACCGGGAGGGAAATAATGAAATATGAGATAAGGAGAACCTATCACGTAGGTAAA
CGAATATGGGTTCGAATGATAGAATCAATATCTATTCTATCTCTATCTATTCTTATTGGGAATAAGGA
ATAGGGATAATCTCAGAAATAATGAGAGTCTAACTATCTTTCTATTTATTATAATTGGTAAAAAGGAA
AGAACATGGGGCATAATCATATAATAGCGATGGTGCAAAATAGGCATGAAGCCACCTTTTACTTGGGA
AAATAGAGCTTAAGTGCGAAACGAAACGAAGGAAGGTTTCACCTCCTTTACACGGTAGCGAAACCTGT
CGCAGAAGTAGCGACCTGCATGAACGGTGGAACAACTGCCCGCTTTATCCGATGGACCCAGTCAAATT
GAATTCTCCGTAAAACTTCACTGACAAGACAGAAACCTTCGAGCGAGATTGAATGCTGGAAGGTGTTA
AATGAATAAAGTAAATTTGCATAATCTCATATATAAGGCATGAGATGAAATTTATGAGTGAGGGGAAG
AAATAAAGAGAAGGTTATCACGGCTTTAATACCATTTTTTTTTAGAGGGGATCCTCCCCATTAATTCG
GAAACTTAGACAGAAACAGAGTCGATACAAGCCAGAATGAAGGCCGGGTCGAAGGTTAGGGGAAGCAT
TACTTAAGTGTTACCACTAACCCTGTTAGATTTACCAACAACCTCTTCGATCCTCACTTATCCAACTT
TTGCCCAAACCTCTTGTGCTTCAGCTAGGAATTTTGGTGGGTACAGCAAACACTTACAACCTTATACT
ATTATGCTCCCTACTTACCTTATACTTAAGTCCATGCTACCAACTGTTCAGTTACTACTGCTTCAACC
TACTTAGAATATGTATGGACGGATGGAGAAAGAGGGAGTGCTGCCAACCAATTCATTCAGCCGGGGAT
CAAAGGAGGGTAGACACTAGTGTATGAACCCTACACCCTACTATTTTTTCTTATTCTTTTTCTGGTAT
AACTATTATTATCCCGCTGAGGAGCGAGGATACATTAAGTATATTGCCCGACCTAAAAAGGGCGAGGA
TACGAGCCGCGAAGCCCGACCCAAGAAGGGTGAGGAGCCAATAAGATTCATCAATTAGAAGAACCCAC
TGCAAGGAAAGGAAGAAATATCTATCTGCCTTTCATTTACTGCCTGTCACCTTATAGTTATCCATCTT
AAGCACCTAGGCTTTTAGCCCTATACCACTTGATGAAATGAATGTGCTTAGTGGGTAGGTAGGACAGA
ACCAGCTATAAAGCTTTGTAGATATAACAAGTTTAACAACTTCGTGAAATGCCATTATTCGTGGCTGT
AGCGTCATTTTGTAGGTTCCAAATAGCACCAAAATTGCAAGTGGAGAATTGTAGGGATGGCTTACAGA
TAGGGCGATGGGTGGTGGTCCAGGTGGTTCATAAATCGGTACCAAGAGAGAGTCGTCTCTTAGTAGTC
CTAGATAAAAAAGCCACCTTCTTAGATACTCTAGCTTAGTCGTCCAAGCAGCCGGTGGTATTGCCTTT
TGGCTTGGTCCACCATAGAGTGAAAGCCTAAAGAAACAAGCTTTTATTAGTCCCGCTTAGTAGTCCCG
CCTGAGCCCTCTGGACACTAACTTAAGTATTAGCCTATTTGGACCTGGAATAGTACCCCTATGTGGAT
GATGGAATAAAGCCTTAAGCCCCAGTTCCTCGGAAACAAGCCTAAAAAGAGACAAGCTTATTAGTCCA
GTTAGTCCCACCTGGAGATGAACACTTGCTTTAGTTTTAAGCAGCAGCTGTAGAGCTCCCTATTGGAC
TGGACACCTAGTTCCCCCTATGGATGGATAGAACGCCTTTAGCCCAAGAGTTCAGGCCTATACAAAGA
GTAGTCTCTTGGAGCTTCGGAGAGTAAGAGCTCTAGCGCGCTAATTGGAAGAGAAAACCCAATATTTT
GTACTATGAACACCAATACTTATTGAACCGGTGTCTTTCTTTGATTTCAAGATTTCACTGAAGAATGA
TTCTTTCACTATTTCTTTTCATCGGCTAGTTCCCCCATGTGCCCCATCAAACAATCCCAATAATACTG
CCCCCAATGTTTGTTTCAATCAATAAAACCGAATAGATTCTATTTTCTCCCTTATTGCGTTTTAATAA
TTCTTTTCAACTCGAGCTTTCTTTCATGCCTATAGCGCACGCGCACACCTATATATGCCATTTTCCCT
GCTTCCACTGCGGAGAGGAAGAGCTCTTCGGAAACTAGCCTTAAGAGACAAGCCCTAGTCTGTACAGA
GTAAGCCCTAAGACACTAGCTTGAGCTCAACTTGCTGTAGTATTAAGCCTAGGTGACTAGCCCCATAC
ATAGTGTCTCCATTCTTTTTTCCCCGCGTGAAAGCCTTAAGAGACAAGCCCTACTAGTCTGGTACAGT
AAGTAAGAGCCCTATGAACTATATTAGCTGTAAAGTGTAGTCCACATGCACACTATAACTTCTAGCTG
ACCGACAATAGCTTAATGCTGCACTGACTGACTGGCTGGTGGGAAATTTCTGTCTCCTTTCATAATAT
TGGGCAGTAGTTGTTGGGAACGCCTTTCTTGGAAGATGTATCAGTTTATCATATTGCATTAGATATGT
GCATATCCACCCAAAATAATAAAAGCACCACCTCACACTCCTTCCACTTGTTCGGTCCCCTTCTTAGT

GGGCTCCTTCCCGGCCAGCAAGCAGGATCATTGTAAGAATCAAATAAGTATCATGGTTTCGCGTACTC
GGTGTACCTACCCACCCGACTCACACAACTTGGTTTCTTTCTTCCACCACCAACCACGGATCGTTGCT
CATTCCCCAACGAGTAAGGCGGTAGGACCTGAGCGTTAGCGTAACATATTCCCTATATTTCTCCTCTG
TATAAGGAAGGAGTAGAAGGAAGGGGAAGGGAGAGGGAGGCAAAGAAGTAGTTCCAGCGGAATCAGCA
GCCTTAGTCCCAGCATTTGAAGCAGCATTCGTTCCAGCATCCCCAGGATAATCTCCATAAGCAGTTGA
ACGGGCATCATTCCAATGAGCAGTTGAACGAGCTAAATTCCTAGCATCGGTCGAAGCAGCATTACAAC
CAGCAGCAGCACCACCAGCAGCAGCAGCACCAGAGCGCTATGTATCATTCCCCTCCCAAAAGGTAGGC
GATAAAGATCAGCGTAAGAGAAACAAGGTTCCGTTAAGTTTAAGCGTACGAGGAACAATGTTCCGGTA
GTGAGGCTTAAGCAAATTAGGAACAATGTTCCGATCGTGGGCCCATGGTAGGGTAGGTTGTTTACTCC
CTCCCTTACTAACGTGGTTCGGCCTCCGGAGGAGATCAAGGGAAGGTTACAAAGCCATCGCCCTTTCC
TTTCTTTCGACTCCCGTTCTGACTCTCCTAAGCGTACCCGTACTGGTGGATCCCGCCCTGATACACCA
ATGCAGCATATATAAAGCTAACCGTTCGACTCGTTTCTCGCTCAACCACAGGTTCGCTACTCGCAATC
AACAGGTTCTTCTTAACACATCCATCATAACAGGGTGAGTTGTTATCTCTCTTGACCCCCCGCATCCT
GGGAATAAGCGATCAATCTCTCTTTGTCCAGAGGTTTCACCCGTATTGACTCTTCTCGTCCTGACTCC
TTACCTAACTGGACCTTCCCTGATAAGACCTAACCGGACACGCTCTTTCTTTCCGCCAGGTTTATCTC
ATGGAGCAAGTGAAAGGTCCTGTTTCTTTTTCGGATGGACTAAAATGAGATCCTGTAGAAAGCAGCTT
AAAGCCTGTTTAGCATCCAAGACTTGTCGCAGGAAATAGGGGGTGTAGGGGGCCTAGCCAAGGTTCAA
GTCATTGTATAGTGATGAAATCCGAGGGGTAGTATGCAAGCGGATCAGAAGGGTTGTGATCAATCCAA
TCAAGCTCATGGTCCGACCAAGCAAGCTCAAGGGTCGAAGCTCTGGTTTATTCGAATGGGGACTCTGA
TTTAGTAGAATTGGGATAATGATTTAGTAGAGCTGGGACTATGATTTAGTAGAAATGGGGTAACGATT
CAGTATCGGGGTCGGTAGGTAAAGTAAAGCGGCCATCGGACAAAAAGCGTATCATAACGCGAACATAA
AGCGGAAGGTGATCCTCCTATCAAAGAGAATATAAGGAAAACGAAGAAGAGCTCACCCCTATTTTACT
TCCACCAGGGGTGAGGCGTAGTCTGTAGTATTCCCGGCTTGTGTTAATGTCCTAATCTGTTGTCCATG
TGATCGAGGTGTATTTATAGATGGAGGGTGTAAGTGATTTGTAATGTTGAAAAGCTTTTGTGGGTACT
ATTCCAGGATGTCCAGGCAAAGGGTCAAGCAATATTGATCTGACCAACCTGAAGGGTAGAAGCTTCTA
TCAAGTTAAAGTGGGTAAATTGATGGGGGCACAATTCTTTCCAAAAGGTCAAGCAAACAAGCAAGCAG
CACACCTACCTTCTTCGATACACCACCTATTCTTTCTGTTGAGTGATTGATCCCGGCCAGCATATTAA
GAACGCTGCCTACAAGTGTATAAGAAACCTTTTTTCATCAATTGTGTCACCTCACACGACAAAATGAT
CCGTTCGTTGAACCACCCGGGCTCACACAAGATTGGCAGTTGTCCGCCAGAATGCCCACTTTGTAAGG
TCAAACCCAGAAATCATCATCAAAGAAAGACTCCTATGGTTACCTACCTGAAACGATATGTTTTGTGT
GTTCCCATCCATCCTTCTTCCCGTCTGCCCCCAGCCAAGGTGTTAGCTATGGTTCTTGGTTCCATAAG
GCGTAATGTGGTATTGGTAATGAGAGGCTTGGTAAGTCTAAATAGAGGATTGTTTCTTTCCTGAAATC
AAAAAGTGTAGATGCTGATCGATGCTTGGACTCCCTGTAATTCCTCTCTGTCTAACGTACGAGGTGGG
TAAAAGCGAGTTTAGGTCAAAGTAAGTCGAGCTCCCCCCTCCACCAGTGAAGAGCCGGGGTTGATTGT
AGACTTAAGTATTGACCGGCTCAGGGTCAAGAGCTAATTTTATGTGGATGCGGTAAAGCGGATAGTTT
AAAGGGTGCAGTAATGCTGACTGACTGCGTATGGCGAACTGACAACCAATGTAAAAAATAAAGCGCTA
AGGCGAGGGGTAAGGTTAAATATTAATCCAAGCTAATGGGTATAAGTTTTCAATATCCTGTTTTTTCG
TGGAAATTTCCGGGGGAACGATTTCTTAGCGGTAACAAAGCAAGCAAGCAGTTGTAGCAATCCTTCCA
ACCTCCTCATCAAGCTGTGTCAATCCTTCGACAAGTGGTCAAGCGTTAGTGATCTTTCCTTCGAGCAT
GAGCGTTATCTCGATTAACTGGTGACTATTCCGGCAAGCGATAGCGATCATGGGCAAGTGTTGATCAT
CTATCCAAGCAACCTCAAGGTTCGAAGCTTCTGTCCTGATTAAGCGGCGGGTTAAACGCAAGGGAAAA
AATAGTATGTAGCGGGTAACAATAAAGCAAGCTGTGGCGATCCTTCAACCCAGCCTCATGGGGGTAGT
ATCCCTCCATTCAACCAACCTCAACGCGCTAGGCGCCCGCGTGTGTAAAGTAATAGCGATAATAATAA
TGCGTGTAAGTATATCCACGTGTATAACACAACTCATCATCGCGTGTTTATTGGCTATGCTCTTCTGA
TTTCCCAACTAGGACGTTCATTCATTCGGTTCCTTCCATCGGATTCATTTATCCTTCCATTCATTCAT
TTGGCTCCTTTCATCGGATCCTTTCATCCTTCCATTCATTCATGGATTCATTCGGGTCCATCCATCTA
TCCTTCCATCCATCAACCATGAGGTTATCACTAAACTGGTACGAATCTTTCTTTCACTGTGTGTTCGG
CCCCCGGAGGGGATCATGGGAAGGTGAAAAAGAAAGCCATCATCGCCCTTTCGACTCCCGGAATATCT
CACTCGCTCTCGGCCTCACTTACCTGACCTGACCTCACTGGATAAGACCATACAATCCCTGATAAGAC
CAGCCCTTATGACCGGTACCTGCCCTTATAAGACCTTAAAAGACCATACCATATTATTTTGTATCTAT
CCACTGGGCTATCGCTCGCACCACAATGTATGTACTCTCGTGTCTATGCGGTATATTCAAAAGAATCC
GACTGCGGTCTCTCAACCGGCGTAAGCGCTCCAACCTACACCTCAAGGGAAGGTTAAAAAGCTTCTTT
CCAGTTTTCAGAGTTTAATCGGAGCAGAAAGCAGTCGAAACAGAAGGCAGTAAGGTAAGTCGGTAAGG
TAAGGCAGTTGAAAGTGATGGATAATGCGTAAAGAGAAGAAGGTATAACGTGTCTTGAACTGAATGGT
CATCTCTTCTTGTTTATATCGGGCCGTTCGAAGTGAAAGCATTCTAAGGGAGGACCACGAAGTTGAGT
GAACCCCTGGCATTTTACTAGAGCCGGAGGACGAGCATAGACTGTAGTTGTCCCCGCTTGGGCTAAAG
GTTATTTCTTGTAGTGCGTTCGAAGGTATAAGCGATGCAAGCGTAAGGCTAATGGGTAGTGTTCGGGT

CCGCGGGTTCCTAACTCCCTTCCCTCCCGGGCAAGTAAAAGAATCAAACCACCGTTCATGGAGTTTAT
TCTAAGTATTCCCAGTGTTCCGTACCGTTCACAGTGTGGTTGGGGGTATCATTATCGGTCCTAATCCG
GGGGGGGATTCCCCCTAAATTCGGCAAGCGTTAGCGATCAGGGTAAGTGTCAATTTATCCAGACAACC
TCAAGGGGTAAGGTGTTGTATCGATAATTCCTCGTCAAGGGGTCAAAAAGGGTATTAGCTTTTTTTAG
GTCCTGTATTATATATGCGAATATGCACTTTCTCTTTGACAATTAATTCTTTTCAATATCTACCTGAG
CTAGACTTCAAATAAGGAAGGCAGTCTAAAAGAGATGACTCACTAACAAACGTCCTAACTCGGCTGAT
TGTCCCATATCGGTAGTGGAACCTCAAATGGTGTATTGTGTCAAGGCCACTCGTTAAGGTACTTGGCT
TGTTGGGTGAATGTGTCAATGTGAGTCGTAAATTGATTGAAGGGCTCGTGCTCTGTTCCTCTATGGTG
TGTTCCAGGCCTCAAGTTTCACTCCCGGAATGACTGACTCACAACCGTCCTAACCCGCGCAATCATAG
AGAAAGTTTCTTAAAAGCCAAATTTGAGTAATAAAGAATCAACTTGAGTGGCCCGGAGTCATCAATTC
TTTGGACCTTCTGAAAATAAGATTTCATTAAATAAGAATGTGAATTTAGACTGTTTTGTGAAATCCGT
ATAAAACTGGGTGCCTGCTCCAAACGTTGTAGCTTAGTAAAAAGCGTATCAACCGGAATCCCTATCAA
GGGGTGGTAACTCACATTATATATGAGCATATGCATTTTCTTTCTTTGTTTGTTTTCCACGTCCCTTC
GCTTATGGGTCAAAATATTGATCTGCTGCCTGTTGTGTGCTGATAAGGGGCAGGGGTAGATGCTTTTG
TCCTGTTTACACGGGGTAATAGCAAGGGGAACTATTCAGCGCTAAAGCTCCTCAAGGTATAAGTGTCT
GTCCAGTGTTACTGGGGTAGTTGGGTAGAAGGTAAGCAGTGGAATCAAAAGACAAGAAGGCCCATGTT
CGTTTCCCGTACTGTACAGGTGTTTAGTCGATGTCTTCACTGTGTCCCGTACTGTAAATTGAATGTAG
TAGGGAAGCGTTGTTGAATATAGTGGAAGCATAGTGATAGCTAGGAGGCAGTGAAAAACATCCCAACA
AGTAGGGAAGCGGTATTGAAATAGCCCAAGTAGGAGGCAAGGCGGTATCAATATTTCCACCAAACAAG
TTGGCAAGCGGCGGCGATCCTTTATAATAATGCAACCCGCGGTAGCGCTCCAAGGTCAAGGGTTAAAG
GTTCAAAGCATAATTACTGTTTCAGCGTAGTGATCGCAAGGGGAATGATTCAGTAGCACAAATAAAGC
AAGCAGTATGATCCACCCACAAGCTCAAGGGGTAAAGCGGTTCGCTCGCTCTTCTTTCTAATGACCCA
ACCATTCTATCACCCCTCCCCTTATTTCCGCCTTCCCACGTCCTCATACAGTAGAGATTTCCCCATTT
AATAGCTAACGCTAATGACTCCACAGAAAGGGTTAATGGTAATGCGTATTGATCGAACTGGGGCTTAT
GGAACGTGGGTTGAACTGGTATCGTGATGCGGTATGCTCGGTAATGCGGATGGGGTAATACGGACTTC
CTGGACGACATCATACGTCCGCTAACAGGGTTCCATATGTAGAGCTAGGCGAATGCAGATATAGAGAA
ATACTAACAGAAGCGAACTGACTGACTGACTGACTGGGGCGGATTGACTAGGGACTTCCTGATGTGGA
CTGCGGACGGACATCCATCGGCACAGACTGAAAGCGGAATGACTGATGAATGCTCACTAACAGAAAAG
GGTCATGGTCAGCGAATGATATATAAGCTAACAGAAAGGGGCGAACATCCTATGGTTCACAGAGGCAA
ACAGCTCACAGAATAGGCCATCATGCAGCTTCGGACTGAAGGGTCCTCAGGCAAGCTCGTTCATAGGT
GGCTTATGCATAAATAGTTTAAGTCTGTTCCCGTATTCCAAAGTCTATTCCCACCCAAGGCAGCAGCT
ATTCCATACGACCCGATCCACCGACGAGGTTTCATGGGCCCAATCACGAGGGTTCATTGCTCATCGGG
AGACTCTAAAGTGAATGTTTTATTTTAGAATATTGCCTTTTCAGGATGTTTCACTATCACAGGCCGGT
CAATTTGCTTTGAGGTTGGTTGGTTGGGCGGGGACTGATTCTACTCTAAATAGATGTGCGTCTGGTAG
CTCATCGTCGATACCTATGGCTTCTTCTCCTGTTTCCACCCTAGAGAGGTTATCTGGGCCAACGTTAG
ACTTACCTGGTCGTATTATGACTTCGAAATTAGAACTCTTGGAATAGTAGTAACCATCGACAAATTCG
GCCTTGGTGGACGGGCTTTTTTACTAGGTACTTGAGCGCTTGGTGGTCGGTGTAGAAAACGAATGGAT
TAGCTAGCAAGTAGTGTTGGAACTTCTGCAGGGCAAATCAATTCCTAACCCTTATTTTACTTATTGGG
TGGGGGGATACTTTTAGTATATGAACTATGTAGGAGAATTGCAAGAATTGCATCGGAGAATTGCAATG
TCGGAGAATTGTCATCCTACCTAAGAGGGAATAGCCTATTTGCGGAGATCGGAGTTTACCTAATAAAT
CCCCTAATAGAGGGGGTCAAATGATCAACCGTACGTAGATTGGTTTCGGAGATCCCCTAAGGAACCTT
TTCGGAGTTTCCCTAATAAACCTTTCGGAGTTTCCCTACGCAAGCGCCTAAACCGCCATTATTTGATA
AATTGTGCTTCCACTTGGGCAACTAAGTCCACTGGATAAAGATCAATTGGTTCGCTGGTTCACTGGGA
TAGCTGGGATAGCTGGCTTGCAATTGCTGGCTGTGCTTCTGGTGCTTAAACGTCTGCTGCTGGTGAAT
CAACGAGTGAAACTACAACCACTGCAGGGTAAACTTATTTTGGGCCTGTAGGCGATTGATCCTTCTTC
TCCCTCTTACGGGGAAATAGCCGTTGAAGCCACGTCTCGGAGCTAGGAAATATGGTCGTTCCTTAGAT
CTGGTTGGTACCGCTCCTGCCTGTTGACTACCTCTTTCCTTAGAATAGGATGATTGGCACCGCCAGAA
CGGGTACCATGTGGGGTGATTGGCACCACCAGATGGTATTGAATTACTGGTCTTCCTTACCGCCTGCT
AGTTGTTACAGATCCTCTCTAATACTAGTCCTCGCTACCTTAAGCCTTCTTACTTGCCTTAAGGAAGG
GAACCTCAAGCCGAGCCCAAAGGCCAAAAACCAAACACTGTGGGGTTATCCTTCATAGTGTAAAGAAC
GAACACTAGAGAGGTGCAAGAACCTAGCTATAAACCCGAGCCAATCCATAACGAATATGATTCCACTT
CATCATAATGCGACTCTTCGCAAATATATATTCCAGCTAATGTAACCAAGATTAAGACAGAGCAAGAG
ACTGGTTAAGCTACCAATAGGTATTGGTATCCGAGGTAGTTCTATACCGACCGACAAGGTAGTCGCCT
GGTAGTTTAGTTTTATAACTACAAGGTAGGCGTAGCCTAGGTACCCGTAGACCAATTAGGTAGTCGGA
TCTTCGGTAATCGTATACCTCTATACCCATACTAGTCGTAGCCCAGGTGGTCATATACCCATAACCAT
TGGAATAGCGCATCTACGGGAGTACAATAACAATAGTAGGTGTATCTCCGGTATTCGTATACCCATAA
TCCATATATCCCAACGGATTCCGATAACTCCATTAGAAACCTACACTGATATATACGTATATAGCTAG

ATACTAGCAACAATAGAGCACGGAAGAACAATAGATAGGAGTTCCATTAGGGGCACACAACCAAGACA
CCAAAAACGATAGGGACGGGGGACATGCTACCGGCGGGCGGGGGAGGGGTTATAGACAGGGATAGTGA
CTGCAGGGGCTACAAACCACCAAAGGAAAGGGCATTACAACAGCGAAGAGAAACAACTACCACTAACA
AACAACAGGGAGAAGCATAAGGAGAAGAGAATAGAAGTGCTCGCCTGGCTCGCAACACCTAAAAGAAC
CTCATCCATCAAGCCCCTGATATAAACCTCCCCTTATCTCACCATAGCCTTATAGACACCTAACCTTA
TTCACCCAAACCCCTATACCCTTATTACAGTTTAGGGATCTGCCTAAACACGTCAACTCGGACCTCAT
ACTATAGGCTTCTCCCACGGATCCGGATACCACAGGAAGCAATCTCCCGTATCCGGCCATACCACAGG
AATCTAGCACGGCTCCAGTCTCCTACGGATCCGTGACAGGTATCCCCCGTAGCCGTATACCACAGTAC
TGAGGGGCCCCGTTCTCAAAGGAGCATATTGGAAAGATGAGGACATATTGAATGGAACAAGACAATTA
ACTTATTGGTACCAGAATGACTCGCGAATGACCAGAAGCTTTCGGACGGGATCCCTTCGCCTCGCTCC
TTTTATCCGCTCAGCTCAGATGGGTCGAATAATATGCTCGTAGGGTGAGGTCTTGGGTTGCGGTTGCT
GTTGGGATTCATTAACAGCAAACACCTTGAAACCACTGCATATAATATATGGAACGCGAGCCAGCCTG
AGATCTCACTCATCATCTTTTCCCCAAGCCAGCCACAGGTTCCACTTGGGCTGGATCCAATCATAGAG
GAACGAAACGACAGTCGTATATAAACCTTATAAGGTAGGGGGACATTGACGCTTTACCCCCACACTGA
CCCTCCCCAATAGATTAGGGATCTTATTATTTACCAACAGTGCCCATCAACCAAGGAGAGCTGCCCCA
GATATATGCGGTCCTCCCTTGGTTCGTTGCAAACCTTCCCTATCTTTCTATTCTATACGGTGGGCTCT
CCAGTAGCGGGCTCTCTCCCGGCCCTCCACTTGCTTCCTTCCTGCCCGACCTACTTGTGCTTCTTTCT
GTTACCGCTAGGCTCCGCCGTGCTTCTGTCCTACGGAAAATGTGCCCCGTGGTCCACGGGAATGTGGT
CAGCTCTGGCCATGGATCACTCGGTTGTATCCGCCCATGG
>Repeat_15
ATTGTTCACAATTCAACCGCGTCATGATATCTATGTCCCGCTAATCAGAGTGAATCTCAATGTCCCAC
ATAAAGAATAAGCCGTGTAAGTAGGGGGTCAGTTCCAGTCATAGTTTGAGATTCAAAGCCCGTTGATC
CACCTGTTCAAGTTCGATAGCCCAATCCCATGTTAGTAAAGTAAAAGCTACACGCCAGGAAAGAGAGG
GCTTGCTTGCTTGGTTATTCCAGTAAGTGCCTTTGCCGAAAGAGAGAATGCCTTTACCGGAAGAGAAG
AGCAACGGCCGGGGGGGCGTAAAAAGAAAAGGGAGGGGGTAAGTTCCAGACGTAGGTCTGCTGTGTCA
TTCGTCCTGTCGATGTTGGGCTATATCCGGTAAAATAGTTTAAATATTCTCCATAAGTAGAGGTAGCA
TCCTTTCCAGGTTGATAGCTAGGAGCATCCCTTACAATTAGGAAGCCATAGCCTGGTGTTCTGGTTCC
CATTCCATTACCAGTCCTAGATCCTACAAAACCAATGGATGGATCCCGATCTCGGTCAATTGAATCTG
GGACTGCTGGATTTGCATATGCATCATTCACTGCTTATAGGGAAACTCAGTACCCTGGTACGGGATCC
CACTCTGGAATAGGATCTTAGTAAACAAGATCAACTGATTCAACCGCTGCTTTCGGTGCTGCTACCTC
CTCTCCTGTGGGTGGTTCCAGATGAACTGAATCAAGGTCAATTGCAGCTGCTACCCCCGCCTTAGCTA
ATGATGGTTAAACGGTAGCTATCTGAATCTTCGGGTTATAGGTCCGTTCGCTCTGAATCTGAACCTGG
AACTGGGTCGCTACTGCTGCTGGAATTGACTTTGCAATGGCTGGTGCCTATATGCCTTTGCTTTAGGT
GGATCAACTGCTGCTCGCTCTAGCTACCTTTGCATCTACTGCCTGTGATTCTGCCTAAGCTGCTGTCT
CGGCTGCCTATGCTGGTGATGAATCAACAATATAAACCGGATCTGCTGCTGGAACTAGATCTGTAGGC
AAAGGAGCTAGAGCTTCAACTGGGTCAACTAGTTCACCTTACAAAATTGGGTCAACTCCAATTGCTTC
AACGAGGTAAACTGGCCCTGGAACCGGATCTGCCGTTGGGTCTCAATGTCGATCTAAAAATTGAGATG
AAATGGGCTATGGATATAGAGCTAAATCTATCACTGGTTTTGGAATGGATCTAACCCTTGCCTTGCTT
CTGCTAGCTCTCGAACGGGCTATGCTACCTTTATAGAACGGACATATAAGAGAGGGCTCATTTATAGA
TAGGAAACGCTTAACCCGCCCGCCGATAAGAGGTTATAAAAATTAATTGATTTGGTTGGTTTGGAGGG
ACGGATGAAGATTAGTTACTACCTTGTTAAGGTATTAAAGAATATGTAGCTCTGGAGGGAAAGCTCTG
AAACTCAAAGGATATTACAACTATATTGGTGCTGCTTCAACTCGATCAATTGAATGATATGGAACTAA
AAGTGATGCTTCCGCTGATGGCTATGATGCTGAAACGGGTGCTTCAATAGGTGAAACAAAAACTGCCT
TTGATTCTTCAATCAGATCAACATCAACTGAGTCAACGGGGTATGATTCAACCTAGGCTACTCGGTCT
CTATTAACTGGGTATCGATCTGCTGGGTCTGGATCAAGATCAACTGGAACAGGCTATTAATTTGGAAC
TGACTTTGCAACTGGTTATGGGTGTGGCTCCTCCGCCCTGCACTCCTTTTCCGGAATGAGTCACCGGA
AAGCGAATGCACCTAACTTATGGGACCTTGGGGACACCCCTTCTAGGATCACTGTAAAATGCGAGCAC
TTCTGAGTAGCCTAATGTCGGAATCGTATCTTCTATAAACAAATAGAGATGCCGGGTCAACTGCTTTC
ACTCGGTCCATGAGGTAAACTGGTGTCCTTGCAACCACTGCTGCTACCTGTGCTACTTTTTATGGATA
AACTCGGTAAACTAGTGCTTTTTCCACCTTGGCTATGCGCTTTGGATCTTTAACTGATCTCGATTGTG
ATTGAATCAACTGACTCTGGATCTAGAGACTCTGGAACCGGGTAAACTCAATCCCGGGCTATCAAACG
GTAACTGTAACCAGTCCTGAATCAAATAAATCAATAGGCACTTCCTCTTCCACTGAAGCTGCCTCCGC
CGCGGATACTTAAGTTCCCGCCCCCGCTCCTGGTGGTTCTAGGTAAACAGGTGAAACTTAAACTGGAA
AATATCTTACTTCTCAATCGACGAGGTAAACCATAGCCTCTCCTATAATACCCTTGCTACTATTGGTG
GCTATGGATCTCCAATCCTTCCTAGTACTGGGCTTATAAATGACCTATAGGCTGCGGGCAATGGAACC
ATACGAAGGGATGTGGCGGGCTATCGATCCCGATCAGTAGTTTACCCAGTTCCCTTTAACCTATTGAT
TCAGTTGCTATCATGATAAAATGTGAAACTCCGGACCTGGCATGAGAGAAGAGTGCACCCCAGACAAT

CAATCTTGAGAAAACGTACGTATCGGTTGCAGCTATAGGTATGCACGTTTATAGTAACAAGCTAGAAT
GTGCGTAAAGTCGGAACATGGTAAAAATCTCGGAACACGGCATATATGGTAGTAAAAGTGCACAGTTC
GAGGAACAACGCTATATAGGTAATATAGAGTATGCACTGTTTATATTATACAGTATGAAGCTAAGCCT
AGCGTTTGATTAATAGTTCACCAAACTAAAATGCATTGAGGCAACAAAACCAACTACCGAAACGACAC
CTTTATAGCCAATCGCACCGGTTGCAACTCCCTTTATATTAAGAAAATCGGGAGTAATTAGTCGTAGC
ACATTCAAGCGCAATCACCGCTCATCCATCGATTTCAATGCCAATGCTGATCCTGGGTTATGGGTGAG
AGAAATAGGTGGTACGTCAAGGCCAGAGAGCAGAGTTTAAGGATAGGAAGGCAGGAGGACAGGGCAAC
ACATACGATCGGAGAAACAGTTGGTCGGGGGTACACATAGGGTCGGTCAGAACACAGACGAATGAACC
ACAGCCTACAACCACAGGCCTAGCTCTCGAACGGCTACTGGCTCGGATATTGGCTATAAAACTGCAGG
GAATGTACTGTAACGGGCTATCAAAATGAAATGGGCTCTGCAGCTAGAGGATCTGGAACAATCTATAA
AACAGGAAGGGATGCCCTATTAATAAGCTCTCAAATTGGATGTGCTCCGGTTGCTTCTGGGTCTTTAT
CTTTATAAACTGGATCAAGGTAAACTGGGTCTCGATCAATCGGCTCAGATGTTGGTGGATAAAAAAGA
GAAACTGATTCTTCGATTGGTTCAAATAAAACTCCCCAATTGGAGATGGAACGGGGGATTCGAAAACT
CGATCAATTGGATGGCGAATCACTTGCACCTGTAATGGGATCTGTAACTCGCTATGAATCTGCACCTG
AACCCGGATCTGCTACTATTGGCTGGCTGGCACTCGATCTCGAAATGAAATAGGTTCCACCTTATGAA
TGAGGGGCTCCACCTTTCCTGTAGGGATTCGGGCGCTATGGGCGCTTATCTACGCCGATTTATTATAT
ACGATAATTCAGTGGCGTAGGTAGATTCGATCCAGCCGTGTCTAATTAGTGCTGGCTGTAGGTGGCTG
TCCAAAAGAAAGGTAGTCTTGTAGTAGGCTGACCAAGTTCAGGAGGTGATCCGCAATGCTCACCCACT
CCAAAGAAAATGTCGGCAAATACGGAATTGATCATACTTGTAGAGGAGAGGTTCTATAATAAGGAAAA
GGGGCTACGCACAGGTATTACCAGAGGTGGCACTACCCAGAGGTTATACACAGAGGGTCCGGTGAACA
GATAGGTTATACAATTAGGTAAGCAATAACATACCCAAACCGAGTTTCGAACCGTTAAGCAAAACTGA
TTAGCTTAGTTGAGTAACCGAGCTGAGTAGGGTATCCAAGCCCACCAGTAAAAGAAAAGAACCATGGG
CCGGCCCACCAAGATGCTGATAACACATCGCTAAATATACGTCGATTCGCAATTTGAGGAACATAGTC
CTTTAGGACTAAGGCATTTAGCCGGGTCAATTAGCGAGGGAAATACCTCTATTTGCCGAAATAGACTA
TTGAGTATCAATCTGGTTCAAGAAGCGCATCACCGAATCCCCTAAAAGCCTGAGCTCCGCGTAAGTAA
GGGATTTGTTCTGACTTTCAGCAAACGTAATTATATACGTAATTACACAGTCAGTCACTGATTGGGTG
GATGGAATGGAATGGAATAGATAGATGGGAAACGCAGAGCAGGTTTCTCTAGTTAGGAGAGGCTGGAA
GGGCTCGCTGCTTCTCTTATCTCATAGGAAGGAGCTCTGTCTTGTCTGTCTCATTATAGGAGAGCTGA
ACAACTTACACATTGGAGATTGATTGAGAGACTGAAAGGGACGCTATAGAGCTCCCTCTGTCCTCCCC
TCTGTCCGCTTTCTGATAGCAGAAAAGCGATCTATAAGGGCGGGCGAAGCGTGGAATGCGAGTTGGCT
CAACTAAATTAGATGTCGTGAACGCCAGACCAAATTGAGTGGTTCGGAGAAAGAGAATCTAAAGAATC
TAAAGAAAGAGACTCTAAAGACTCTAAAGAAAGAGACTCTAAAGATAAGATAGTTCCTTTCGGAGGAA
AGAAAGATGCTAACCGGGAAGGATGGTCTAGCAGCTATTTATCAGGCAGGTAACTTGAACTGAACTAC
CATCCAATGAACTACCATCCAAAGTTTCTGCCCCTCGCTGAATGTCTCTCTCTATCAACCTGCCCTCC
CTGACCTGTATGGTTCTGACTGTATGGCTCTTACCCTTCTGGCAATGAGACTGACAAAGACCTTAAGG
CTTAATGCGACAATGACTGCTGGCTCCTTCACTGATAGGTAGCATCGGTAAGCATCGGTCGTATAGAA
TACGGGTTAGGGAGAGCACGTGGGTAAAGCCTTAGCTACAGGAAGCACCCTAGTCCTAGTGTATCCGA
GCCAGTCTACTCTCCTGGTTAAGCCACTGAAGAGCTAGCAGTAGTAGTTTCTTCAATTGACCCAGCAG
TTTATATTGACCGAGTGAAAGCATAAGATCCAATTCTAGTCAAAGTTCCAGTGCCATTCATTGGATAT
CCAGGTCCAGCAGCAAGGGCGGTAGAAGCAAAGATACGAGAAACATCCTCGCCTGTATAATCGGCATC
CGGACCAGCACCTAGCACAGATCTATATCCAAATCCTAGCCTAGTGACAAAGTCTAATCCATTGAGTC
GGTTGATCCCGCATAGGCACGGGCAAGGGTATCAAAGCCAACGAATCATGTTCCCAATCCAATCTCTG
CTAATTCGTTTCAATTCCAATTGAAGCATTACATATAGATTGCATTGACCATCAACAGGAGAGGCAGC
AGGAGGAGAGGCAAGCATAGCTAAGGTGAAAACAGAAGTTCCAAAGCCAGATACAGATCAAGATCGAC
CCTCGCCGGGTTAGAGAGCCGAAGTAGGGAGTTTCACTAGCAACAGAAGGAAGGGCAGGAAACCTAGG
TTGAATCAGAACCGCTACCAGCTGCAGAGGCATTTCCAGGTGCTTATTGAGTTCCAAAGCCTATTCAT
GTTCTGGGTGAAGATCCAAACCCAGATCCCGTGCTAGTTGGAGAGCTACCACCTAACCCAGCATCTCT
TTCATTTCTAGAACCAGCATCAAAGCAAGTACAAGATCAATATAAAAGTAGCGATCGATGCACTCGAT
GACAGGTGGGCAGCCAGCCCTGCTCCTCCCTCCCTGTTGTTCCTTGGTCGTGTGTTGTTCCATAGCAG
CGGATCCACTCAAATCTGATTGCTTGCATAATGACATAGCTCCCCTCTCCCTAACAGAGACGGCCATT
CAGTCCATTCCTTAAATAGTTGTTCGTCAGGGTTCCACGCTCACTTACTAGTGCTTTCCTCCTGCTTT
CCTCTGCTTTCAAAACGTCTGCGCATTTGATTAGGGCACCCAATATTCTATGCCGAGAGAGATACAGA
AGTGCTTGTTTATAGCCGCTAGTACTTGCCGGGAGTACTCGCTTGAAGGAAAGTCAGTGGCACACATA
GTAAGTTCTCATTGCCGGTTCTCTTTGCTGATAGCCAATAGTTGATACAGGAGTCCCGGCACATAAAC
TGGGTCGATGCCGGGAGTATTTGCCACTAGTTGAGGTCAGGATTCGATGCCGGTACTCGATCCAGGTG
TAAATGCACTCAATGCCAGGTGCCAATACCAGGTTTCCATGCACTCGATCCAAGAGTAGATCCCAGGT
TTCAGTGACAGTCATAGCCCGTAGGCAACAGTCTCATAGACAGTCAGTGATAGAAATAACTCCGGTCA

GTCAGTCTAGAATGAAGCTCTACTCAGCGAACCTATACTCAGTGCATTAAGCTCTAGTCTGTACCTAA
TAGTACGTGTGTGCCCACTAGTCATTCAGGAAGCGATGCCAGGACTCGATGCTCCTACTCGATCAGTC
GATGGATGCCAAGAGTCGATGCTAATAGTCGATCCAGGAGTCAGTATGAGCTAGAAGGTAAGGTGCAG
CATGAAGCTATGGTTAGTGCAGCTATAAACCTCTGGTCAGTGCCAAGTACTCATTTCCAATCGATTGA
TCATAGCCAATCTGAGATACCCAAAGGTGCTGATAGTCGGTAGTCGTTTAATGAAGTCTTAATTGCCG
GGATTCATTGCTGATAGTTGATAGTCGCTAGTAATTGATGAGTTGGGATACTCAGCTACTCAGTTGGG
CTTGGTTACTCAGAATTGGATACTCGGGCTGGGCTACTCTCTCTAGACTTGGACATTATACTCAGCTA
CTCTACACTGGGGTAGGATACTCTACTGGATGGAAGGAGCTCCCGGGATGGCTGCTGGGGGACATTGA
TTCTGTGAACGGATTCTGGGACATCGCCTCTATGCTCTAATTGCAAGTGAGTTTCCCATGTTATCACC
GGGAGGGAGGGACGCACGGGACGGTACGGGGCCCAATGAGTGAGTTAGGCACAGACATAGGGCCATTC
CCTATAGCCAATACCCAGTAGCCAATAGATTGATAGCCGGTAACAATTGACGGTAGCAGATAGACAGT
AGACAATACCCGCCCGGTAAAAATTATCACCTTATCTGGGTCGCGGTTGATTAGGTTATCCCTAATGA
AGGTTATCCCACATAGTATGGATCCTTGCCTGGATTAGGAAGTGGCTGGATCGCTTCGCTTGCCTGGA
CCTATCCTTAATAGTAGAACTGAGCTCTATATACTTGAGGAATTTCTCTTTGAGTGGAATAAGGTTAG
GTGGGGCTGATCGATATCGCTTGTTGTGGAAGGTCAGGTAGTCAGGGAAGGTGCGTCTACTGAACCAC
TTTTATTAATTCCTGCTTTCTCTCTCTTATCTCTATATCAAACTTATGTGATGCTGAGGGCGTTTCTT
CTATCTTCTCTCTGGTCCAAACTGTTGAACCAATATTAGTATTATTATTCCCAGCCTCAGTTCCCGGA
TCACCATTTGAACCAGTCTTATTCCCAGCGTCAACAGTTGAACCAACATTTACATAATTAGTCGAAGG
GGCTCCCAAGAGCAGTTTAACCAACGGCATTCCCATCAGCAGTTGAAAGAGCATTCTTCCCAGGATAA
GTTCCATCCGCATTTGCATCATCAGTCAATGGAATTATTTGAATAAGCTGTTTCAGGAGCATTATTCC
CAGCTTAATAAGCACCAGTCGAATTAGCATCAATTGCAGGTCTAAGAGGTTCGATTTATCTGTCCCAC
CACGTCCCCGTTAGGGCACTGTAGGCAGTCGTTCGTCCTTTGGACGCGCCACCCACCCACCCTACATC
TGTAGGTCGTAAATCGAAAAATAACTCTCTGTCTAAATCGTTTTAAGGCCGCAAGATCTCGATCAGCA
TGAAGTGCCGCCAGGGGGCGAGGGAGAGGTCTATGGTTCCGTAGAGTTAGAGGGTTGAGGGTGAGTTC
GAGGGCTAGAGTTCGAGGGCTATGTTTCCTACATCGAGGTTCTATTTCGAGGAATAGATCTAGGGTCT
AGGTGGGGGTCTAGGGAGATTCCGTAGCGTTTATCTGTGTTTGTCCAGCCGATGCAAGCAATGACTGT
GTCCATCCCGGCTATGTGTTTCCAGCCCAGTTTATTAGAGCCCAGCCCGGTAGCGGAGCCCAGTGTAT
TAGCCCCAGTGGATTGGATCAGCGGGGTAGCCTAGCGTATTGGATCAGTCAATTAGCCAAGCGGAGTA
GCCAAGTGTATTAGCGGAGCCCGGTGTATTATAGCCAGGGGATTGGATCAGTGGATTAGGCCCAGCAG
ATTGTATCGGCAAATGGAATCAGCCGGGTAGCGATCCGAGTCGATTAGCGTAGCGGATTAGAGCCAGT
GGGATTAGAGTCTAGGGGATTAGAGGAACCAAACCGAGTATCCCAGCAAATCCCAGCTATGACTGACA
TCTCCACCAGGCATCAATCTCTTGGGATCAAGTACCAGCATAGAGTACCGGCATCGAGAGTGATCGCC
ACCTGTATAAAGTACCGGCTATTTAAACCTTCTGTAAATGACTACCTCCTTACTGCTAATGAGACTGG
CTATGAGTGGAAATGACCTTAAAGCAATGACAAGAGAGTTGCACTGACTGAGTAGAGCTTTCCAAGAG
TAGAGCTTCTTTCTGTTCTGGCACTGAGAAGAGCTTCATGCACCAACTAGAGCTTCTAGGCCTCCATT
GATAGGCATACTTTATCGGGTATGTTTGAACGGGGTGAGGTACGAACCCGCTCCCCCGACCAAAGAAG
GGGGAGGGGTCTTTGTTTCTATTTAGGGTTTGTGTTTATGGTTACTCTCACTCGGATCTGTTGAACAG
GTTCTGTTGTTTGAACGGCGGGTCTCCAATGATGGGTACCTTCTATTGTGGTCTGGCGAAAAGGACTT
CCCTGTCGTCTGGCGGCGGCCTTCCGTCTGTTGGAAACTAGGGGGTCAATTTCTATTCTGGTGGTCAC
ATGGGGGCGATGATAAAGCAAGCGAACGTAGGCAAGTTATTAATTTCCAGGGATGTCAAGATCGCTAA
AGCGAGCTAACTTCGGCAAGCCAAAAGTTCGATCAATAAGCAGGCCAGGTGTAAGGGTAAGTGGGCAA
GGTCGATATAAGTCTGTAATCAATCTATCAGGCAGCTGCCCTTCTACCCGCAATCAATTTAGCAAGCA
GCTTTAGCTCAACCTAAAAGCAAATGATGCCACCATCCCGGCTATCATCGGCAATGACACCTGGCAAT
GACTCCTATCTATGATCAACTATGATCAGCAATGATCGATCGGCTCCTTCTGACTATGATCGACTGTC
TATGACTGACTACTGGCTATGACTATAGCTTCAATGATTAGAGCTTCTAGCAATGACCTTGTAGCAAT
GACTGGAAATGACTGTCTCCTGCTATGACGACTGGCAATGATAAGCTCTCGGCACTGACCTTCAGGCA
ATGACAACTAACTGGCAATGAGCGTTTTACTATGACAACTGCTCTGACTGGCAATGATAAGCTCTTGG
CTTTGACCACTGAAAATAACCTGGCTCTGACCACTGGAATGACCTTCTGCCAATGATTGGCAATGACT
GGCTGGCTATGAAAACGGGGCTATCAACTGGACTATCGGCGGCAATGCCCTTTTGGCTAGTACTCCTT
ACTATTATCGTCAATGTCGGGTGCTGATTGTGTGAGTCTGCTATGTGAGGTGCTCTATGTGCTGTTTC
TGTATAGGTGGCCCGGTCGTACTTCAAGGGCGGGTGCCTGCGGACTGACTATGGCTTACGCGGACTGA
TACTGTGCGTACAGTTAGCTCTAGGCTCGAGCAGGCAGCAATAGAAAGGGCAGGTAGGTGAGAGTGTG
TCCAATTGTGTTTTTTTCCACGTCCCTCCCTAGGTCTAGTTATATCGTAAGTAAGGGATATACCTACC
TTCGGCAAGAAGCAAGTAGGATCAGCAGGCAGGTGCGTAAAGTTGTCAAGTTCGAAAGAAGCCCGTCC
TCAATCTAGCAAACAGCGAGGTTCCCGCGGAGCCTAAAAGCAATCGACGTGATCCTGCCTTTCATGGG
TAAACTTTATGAAATGCCAATATTCAGTAAGAATAAAATCTACTTTATGTGCCCCGATGAGATTTCAA
GAAAGGCGTTTTTATTAGCAAAGAATGTTTAATGAGGCTGTTTTCTCTAACCGGTATCAAAGCCAAGT

ATTCGAGCCAAGCCTAATAAGGCAGCAGCCACAGTTGAGTAGCCCAACCGAGCAGTCCAGCCCAGCAG
GTATAAGCATTAAAATGTTGTTCCGCCATAGCGTTAAGTGAGTTCGAGTTCTCTGTAGGGTATATAGG
TCTACGGTTCCGTAGAGTTTAGGTCTAGGTATACTTCTATGATTGGCTGCTCTAGGCTCATACTACGG
TTCTATGCCCCTCATCATCCTTCCACCATGTAGAGCGGCGAGAGTGAATATATGAGCGTAGCAGGCAG
AGCAATGAATCTATAGAGCAATAAAAAAACAGTAGTGTCAGTGTGCGCATAGCGATAGTGTGTCAGGA
AAGAGCGTAGCAGGCAGAGCAATGAAAAAGAAAGAAAGATCTCCATATGCCCCCATACAACTTAATGA
GCTGTATATAGCTCTTATTGGTGGGATATGATGAGCTATAAAAGTGATGATATAGCCTATTAATCTTA
TCTTTTCTTACATAGATATAGCTATCTTTTCTGATTGACGGGGGCCCCTACCCTATCCCCTTTTGCAA
GCTATTGACCCTGCCTTCCTCCTTCATCGAACGAATGATCCCCTAAATGCCCACCTCCTTTATTAGCG
ACCCCCTGTCAATGACAACCCACCTCCTTATCGTTGACCACCGGCTCTGGGCTGACCTTCTGGCATAA
GCTATGTGACTTTTGTATTCACTTCTTGTGAATTAATTAATGTCTGTAACACATCTTATTACTTACAT
TGTTCCGAACCGAGTAGTAGGCCCCGGCCAAGTATCTGCTGAGTATCCCAGTTGAGTAACTAAGCCCA
TCGGAGTAACCAAGCCAAGCAGCTGAGTAGATCCCCATTTGAATAGCCCATTGTTGTATTATAACATA
TATAATGAGCGCTGACAAAGATGAACCATCTTACAAGAAAGGTGTTACCCCGATTACCCTGATTCCCC
AGTCCCGCATTCTATTATTATTGCATTCCCGCTCTCCCCATAAGGCTCAGTACGCGTAAGCAAGGAGG
ATACCACCCGGGCAGGACACATATTGTCGAAGAAGGAAGGACCAGTAAATGAATTGAATTTGATACCT
GCAGCACCCCGGCGGGACTAATATGCCGGGGTAGGGAGGTCTTCGAAATGAGTGGACTTCCCTTCCGT
TCACCCGAGCCCGGCCTAGCAAGCCTTCGCAAGCGTTATAAGGTAAGGCCCGAGTGGGCCCGGAAAGC
ACATGATTTTAGAGCTTTCAACACCCACTATCACAATCCTGCTCGAGCTACTTCTCTCTCCTGACCAC
TGGCACAATCACCTTACCTGGGAGCATGGGATCCACTGGTTAGGTAGGCGGGGCATGGTATCTACCCA
CTGAAACCATCCATCCTCACTCTCACTTACCCGGGGGGAACAGGAGGCACGAAGTTCTTTTATTGCTC
CAGGCCATAGCGTCGCCTATCAATTACTTCTTTGGGCCGCAGGAGGCTTTTTCTGGCAAGAGTCCTAT
TAGAAGAAATCCCCCTTCATGGAGACGAAGTAGGGGGGCTCTCTACCCCACCCGTATACCACATTGCA
TACCAAAGTGTAGTAATTCTCTACTCTCACACACAACACATAGTGGGAAAAGTATGGACACCTAGCAA
CATGGGATGGGGGAGGACCTTTACCGCCCTTTAAACCTGAAAGGCAAGGCAAGCAAGATCTGTACCTG
TAGGGGACAGTAGATGAAGACCTTTACTTTTAATACTACCCACCTGCCATAAATTATTCTATTGAAA
GCCCTAGCTACATGCTTCCCAGGGAATGAATGCTTGAAGACCTTAGGAGCACCAGATATCGTTGGATC
GGTGGATGGGATTGGGATGGGAATCAACATGGCCATGGGAATCCGTTCATTAGCCCTTACCTTGCTCA
GACAATACACCAGAATTACCTTTCTATTAGCCCTAGACCATGCTGAAATGGGAATGCTATCAGACCTG
TATCCGGATCAGATCGATGCGATTATGCAACTGGGATGAGGTTCATTAAGCCCTTACCTTGGAAATAC
AACCCACCTGCCAGACAGACTTTCTGCTGCCCTAGATTAGGAAGTCTTACCAGTTACCAGTTAGACGA
GTATCAGACGAGGGGAGGTACCACTAGCAAACTACCCTACAAGCAGCGACCTAGGACGCCAAATTGAT
TGCCATGGGTTCAGTCATTAGCCCTAGACCTTTGAGAATATGCTACCTTATCCTTAGACACCTTCATC
ACCCCAGTCAGTCAGTTATAACTACCCTAGAACTCACAGGCTTAACCTTTCTCTTACCCACTACTTAG
TTGCTTACAGGACCCATACCCTTCTCTTTCTTTGTTATGACAATACACCTCCTATACAGAAGGAACAC
TTTATCTGCCCTAGAACTTCAGCTTAGCTACAGCCTTATAATGCGAGAAGCGGCTACAGTATAGTGCG
TGGTGCGTGAGGAGCACCCTCAGTATATAGACGCGCACCAGATTTGTTGGGTTCATCAGCCCTAAAAC
CTTGAGAATTGTACGGTCGCCCAGAGCCAGTCGCCAGGAGGTCTTTTTTGATAGCCGCTAGTCGATCC
AGCAGTCCATGCCAGGTGTCGATGCAGTCAATGCCTGGTGTCAGTGCCAGTCAGGGAGGGAGGTGGCT
GATGCTCGCCCGGAGAGATCTTGATTAATTGCCTACAGATTCGGCCCAGGCACCAAGTTGGCTTGGAA
CAGATTTCACAAAACCTTCTCATTTGACATTCTTCTTATGCTCCGCACCTCGGTTTTAGCAGGTCCAA
GCTTGAGTGATCGGGGGTACTAAATTGGATTTTTTTATTACCGAAGATTGGCATTTCTCGGTAGTAG
ATACAACAGTAGATCCAACAGTAGATGCACTCGATGCACTCGATGCCAGGAGTCGATCCCGCTAATCG
ATCCCGGTACTCAATGCTGGTAGTCGATCCAGGAGTGTCGATCCAAGAGGCGATCACTCGATGACAGG
TGTTGATTCACTCGATCCCAAGAGTTCAAGATGTCGATGTCAGGAGTCAATTTATTCTTGTCTGGTTG
CGAACCGATGGAGACGATCTTACGATTCACCTATCCTTGCTGGTTAGCTATCGTTTGCTTGCTGACTA
GACGTTTGGATAGACGAGATAACCCGAAGAAGAAGCTGGTCCAACAAACGCATAATGATAGAACCTGT
GGTGGAAAAACGTTAGGCTGGGACAATAACCCGGCTGGCGGGAATCGAAAGCTAGCTGGTGGGCTTAC
TAACTGGGATGGTTTTTTTGCTTGCTGTTCAATTTGAAAGCCTAAAACCCGTCCTTGCACCCTATGAA
GAAGGGCAAAACCTATAAGAACTATTCAGCAGAAGGTGGCCTATCACTTAGTCAATTATATTCCTGCC
TGGAAATTGGCTAGTGAAAAGGATTACGTATTAAGGGCAAGACCTGTCAAACCTTCCCTTAAGCGGAA
TTTAAGAGTTAAAAAGTGGCCTATCAGTTGGAAATTCTACGCCTTGTCAACACATGGTAACTTCACTT
CTTTTAACTTAGCCTATGGGGGTGGTAAATTCCTCTCTGTTAACCTGGCCTATCACTCATTTGATTCC
GGGGCTTGCTCTGTTTCGTCATATGAGGAGGGTAAGGCAGCAAATATAGGCGATCCTCGCTTCTTTGA
ACGGTTGTAGCCTATACTGGTACCGGTACTGGGGGAATGCAGATACTTCCTCCTTTTTTAAGCCAACA
AAACCCCGGAATACCGAAGAAAGCGAAGGACAACGCAGCGGGGGGAAAGATCATCCCAGAAGCAAACC
CTGGAACATTGTGAGAGGAAGGACTCCCTTAAGCCCAGCAGCATTGGAAGATCCAGAGCCTTAAGCCC

AGGAAGGAGGGCAGGAAGGCGCCAGCCTTAAATGCATTAATTTCAGCGGCTTACTAGATCAATTTTGA
GTTTGGGACCCACATGGATCATATTACTTGGAACCCGGATAAACACAACTTAGATTTACCCAATACGC
GGCGAAGGTTCTAATTGACCGGAACCACCCTAACAGAAACAAATAGCTTTTCGGGTCATAGTTTCTGG
CTGGCTCTCCCGCCCCAAGCCAATCAGGTTATCCCTGAATTATAAATATTTTTTTGGGGGGAGGCACA
TAGAATAAGATTTTCAGTATTTAGCAGACTTTAACGCTCCCCTATTATGGGTTTATTCCGTGCACCCG
GTCCCGGGGTTCGTTTCATAACTTATTATTTATCCCAGCCCGGATTCAATCAGCTATTACCCAACTAC
TCGATCTATCTAACTATTAATCGCTCCCCCTGCTATAGCGATAGGCTCTTTTGGACGTCCTGTCCTTC
TATGGGTCGGCGGCTGGCCCTCTCTTCATTCGCTCTGGGCTTTCATTCATTCATGACCATTGCATGCG
GCTATCCCTATCATTGATTAATATAGATAGAGGGAGGTTTGCAACCTCGGCCTCGGCTTCATTCTCTC
TTTCTCCATCCTTACTCAGCTGGCTTCCTAAGCTTGCTGCCCGCTCTTCAATCCAAAGAGCACAGAAG
GTTTTTGCATGTGTTACGCATATGGATAGATAGCCCTAGCCAGCATATAGCTAGCGGTGTTGAGCCGC
GTTCGTGAAAGAGATTAAAGACGCTTTTAATATGACTGACTTAAACGAACTATAGAGGAGCGACTTAG
TACTGGCTCCCGATACACGCTATTCAAGCTTCCATGACAATGACTCGGTGACCTTGGAACCTTTTATA
TAGTTCCCGCCCACCCCTTCAATTAGGCAGCGGACTAGACGGAAAATAGAATCAAAGTATTTAATTAG
AGCCCTCTGGAATAGGAAGAGCCTTTCATTATTTATAAGGTCCGAACCCGCAAGATCTGTAGAAGCCA
ACCGATGTTAATTCGTCTATTCAAGATGGCAAACTATACTTCTGTTCCAGGTCGTGGCCTCCTATCCC
CCACCATTTCAAAGGGCTTTTTTAGGGGTTTCGGGCTTTCTTCTTTCCGAACGATATTCTCAAATTCT
TAAACCTTAAAATAGAGAGGGAGGGCTCTGGCTCCACGAAGAATTCTGCTCCGTTCCTTCCCCTCTTA
AAATAGAGAGCGCCCGGTCCCAGTTATTCCCCCCCCCATGGTCGCATACCAAGGGAGAACGGGTATGG
GGTGGAGGGATTCGGGCTTCTTCCTAACCCTATGGGTGGTAGATAATAATTCTTTTTTAGGCTCCGTC
CATTTCAGATGCCCCATACTCCTTAAAAAGAGAGGGGGCGCTCCTCACCCTATTATGTCGGGTGAGAG
CTTTTCGCTCCTCTCCCCGAAGAAAGATAGATGGGTCCGGTTTTATATAGGGGTGACTATGGCCAGAT
CTGAATGCAGATCGAGCGAATTCGATGGATATATAGGAATAGGAGAGATTCCGGCTACCGAACTCCAG
GTTATTTCGCTACCAAACCGGAGGGGGGGAAGTGGGCTTAGTAGGGAACTATCTAGTAGATACCCATT
TATCTAAGCTTTAAGCTCTAAGCTCTAAGGCGGGCGGCGCAGAACGACTTACAGAGATACCTTTTATT
TAAGTAGTTGCCTTTTGTCCAAAGTCCCCTGCTCAGTAGGAGTAGCTCGGTGCCCTTCGGAGAACACC
TCATGGATCTGGCTCTCCTCATGAAAGAACAAATCTATCTATCCAGTCCCTTACCGAAAAGGAAGGAT
CTCTCACCTAATAGGAAGGATAAGCTCATATATAATAGCGTGTGAGGGACGACCCATGCCTGTCCAAA
GGGGATACGTTATAGGGAAGACCCTGCATTGCTGCAATCTAGCTGGAGCAGTCCTATTGCATTGACTA
GGGGACATGTAGTGGTGGAAACCCGGGTAATGGGGCAAATCGGTAATGGGCTACGGGAGCAGAAGTTA
TGGCAAACCGGGATTGGCTACGGGACCTGTAGTGGTGGAAACCCGGGTAATGGGGCAAACCGGTAATA
AGGCAAACCGGGATATAGAACTGACCAGACCAGAACTGACAAGACAAGACCTGAACTTACCAGGCTTC
TGGTCAGGCACACGATGACAGACATCACTTTTATCAGTTATTATACCATGTAATTGACTCGTATATAA
TGGATAACGACTCCTTGGCTAACGACCCCAATTACGGAAATAGAAGAGCCATTACCTGAATACCTTAC
CTTACCTGAAGACCTTACCTGACTTTACGGCGCTCACTAGCACCGACAAGCACCCGCTAGACCGACAA
GCACCCGAAAGCACGACAAGCACCCACTAGCGCCCACTAGCACCCACGAGCACCGACCGACTAGCACT
GACCCGCTAGCACTGACCCGCTAGCACCGACGAGCTCTCCTTCCAATAAAGAAATCCCATTAGTCACT
GGTGTAGAGGGAAGACCCAGATTGATGCTAGGTGGATCGTTGATTTGTAGAGCAGTTATGAGGGGAGA
CCTTTACTTAATGGGGAATATGAAATCTGGCTTCCTTTCCTTAATACGGACTACGGAATGTGGGTCGG
TGGATTGGGAATACCCTGATTCCTGCTATGTGGGTTATCACTTTCTATAGCATTTCTCTAGGGTTGAC
TCGTATATAATGAATGGATCTAGTCCCACCCTCCTTGAGTAGTGAGTTTGTGTAGGTTATCACTTCTT
TATAGCGGTGCGATGGGAGCAGTTCCAGTTCTATAGCATTTCTCTAGGGGAGTTTATGGAGGGAAGAC
CCTATGAAGAATGAATAAAAAGGGGAAGGCCCAATTTGTTTGATAGGATTGGAGCGGTATTTTCGGCC
AATCGATCAGATCCTTCTATATACGATACGTTATTTCCCATCGCAGGAGAGGGCTAAATAGAATCAGC
AATTCTATATGGATCAGCTAGAGGGGGGGAAGGAATCGAATCTCGGCCAGCAACGGTAGCAACCTTCAT
CCGAGCACACATACTGAACCTGGAGAATGGGCATCCTACATAGCCTCGTTGTCCGCATGGTACTTCCG
TGCCATTGCATTGCGGGTTGGTCGTGTCATTGCTGCTGCAAGTGCTTATAGGCTCAATCAAGATTACA
TACAGATTGCTTGATCCTCGTCTTTTTATGAGGAGTTAGCTGCGCATTGCATTGTGGCATGGTCGATT
GTTCCGTTCGCCCCGTTCGAAGGCCAACTCAACTATATATCAATCTATATAGTCCTTCGTTAATTCAA
ACGTAGGGTCATGCGCTGCGCCAATTGAATTGATAAGTAGAGCGCTGGGCCGCCTTGCGTTGTAGCAT
GGTCTTTCTCCCCATCGCAGTCGCTCATTACTCCATTTGAGGCCTGATCGATATAGGGGCGGCATGCG
CTGGCTTTTCATGCATGGTCGGTTTGTTCCTTTGCACGCACGGTGGTGGCTTGATCGATTGATTGATC
CGATCCTTAGTAGCACCATAAGTACACCACTGGAATAGAATCCCTGTCCTCCATGACTGGAATAGAAT
CCATGTCCTCCATGTTAGTTATGAGTGGATCTGCTCTAACCTTTGGCGAGAGGAAGGAGGAGGAAAGA
GGATACTCCCTCAGTCAGTCAGTAAGGGTAATAATCCAATGGAAATAAGCCAATGGACTTATACTTC
CAATCCAATGCAATCCGATGGGGGAAGATAAAAAGCGTAATTACATATATAATAATATATTTATTAAA

TTGGCTTTGAGGGGCATCAATATGGGCGTTGGTCGAGCATCTCAAGATCCTCAAATAGCTCTGCGTAA
GGGAGAACCTTACCTTACTTAGAAGGAAGGGGAAGA
>Repeat_16
TCACAATTCAACCGCGTCATGATATCTATGTCCCGCTAATCAGAGTGAATCTCAATGTCCCACATAAA
GAATAAGCCGTGTAAGTAGGGGGTCAGTTCCAGTCATAGTTTGAGATTCAAAGCCCGTTGATCCACCT
GTTCAAGTTCGATAGCCCAATCCCATGTTAGTAAAGTAAAAGCTACACGCCAGGAAAGAGAGGGCTTG
CTTGCTTGGTTATTCCAGTAAGTGCCTTTGCCGAAAGAGAGAATGCCTTTACCGGAAGAGAAGAGCAA
CGGCCGGGGGGGCGTAAAAAGAAAAGGGAGGGGGTAAGTTCCAGACGTAGGTCTGCTGTGTCATTCGT
CCTGTCGATGTTGGGCTATATCCGGTAAAATAGTTTAAATATTCTCCATAAGTAGAGGTAGCATCCTT
TCCAGGTTGATAGCTAGGAGCATCCCTTACAATTAGGAAGCCATAGCCTGGTGTTCTGGTTCCCATTC
CATTACCAGTCCTAGATCCTACAAAACCAATGGATGGATCCCGATCTCGGTCAATTGAATCTGGGACT
GCTGGATTTGCATATGCATCATTCACTGCTTATAGGGAAACTCAGTACCCTGGTACGGGATCCCACTC
TGGAATAGGATCTTAGTAAACAAGATCAACTGATTCAACCGCTGCTTTCGGTGCTGCTACCTCCTCTC
CTGTGGGTGGTTCCAGATGAACTGAATCAAGGTCAATTGCAGCTGCTACCCCCGCCTTAGCTAATGAT
GGTTAAACGGTAGCTATCTGAATCTTCGGGTTATAGGTCCGTTCGCTCTGAATCTGAACCTGGAACTG
GGTCGCTACTGCTGCTGGAATTGACTTTGCAATGGCTGGTGCCTATATGCCTTTGCTTTAGGTGGATC
AACTGCTGCTCGCTCTAGCTACCTTTGCATCTACTGCCTGTGATTCTGCCTAAGCTGCTGTCTCGGCT
GCCTATGCTGGTGATGAATCAACAATATAAACCGGATCTGCTGCTGGAACTAGATCTGTAGGCAAAGG
AGCTAGAGCTTCAACTGGGTCAACTAGTTCACCTTACAAAATTGGGTCAACTCCAATTGCTTCAACGA
GGTAAACTGGCCCTGGAACCGGATCTGCCGTTGGGTCTCAATGTCGATCTAAAAATTGAGATGAAATG
GGCTATGGATATAGAGCTAAATCTATCACTGGTTTTGGAATGGATCTAACCCTTGCCTTGCTTCTGCT
AGCTCTCGAACGGGCTATGCTACCTTTATAGAACGGACATATAAGAGAGGGCTCATTTATAGATAGGA
AACGCTTAACCCGCCCGCCGATAAGAGGTTATAAAAATTAATTGATTTGGTTGGTTTGGAGGGACGGA
TGAAGATTAGTTACTACCTTGTTAAGGTATTAAAGAATATGTAGCTCTGGAGGGAAAGCTCTGAAACT
CAAAGGATATTACAACTATATTGGTGCTGCTTCAACTCGATCAATTGAATGATATGGAACTAAAAGTG
ATGCTTCCGCTGATGGCTATGATGCTGAAACGGGTGCTTCAATAGGTGAAACAAAAACTGCCTTTGAT
TCTTCAATCAGATCAACATCAACTGAGTCAACGGGGTATGATTCAACCTAGGCTACTCGGTCTCTATT
AACTGGGTATCGATCTGCTGGGTCTGGATCAAGATCAACTGGAACAGGCTATTAATTTGGAACTGACT
TTGCAACTGGTTATGGGTGTGGCTCCTCCGCCCTGCACTCCTTTTCCGGAATGAGTCACCGGAAAGCG
AATGCACCTAACTTATGGGACCTTGGGGACACCCCTTCTAGGATCACTGTAAAATGCGAGCACTTCTG
AGTAGCCTAATGTCGGAATCGTATCTTCTATAAACAAATAGAGATGCCGGGTCAACTGCTTTCACTCG
GTCCATGAGGTAAACTGGTGTCCTTGCAACCACTGCTGCTACCTGTGCTACTTTTTATGGATAAACTC
GGTAAACTAGTGCTTTTTCCACCTTGGCTATGCGCTTTGGATCTTTAACTGATCTCGATTGTGATTGA
ATCAACTGACTCTGGATCTAGAGACTCTGGAACCGGGTAAACTCAATCCCGGGCTATCAAACGGTAAC
TGTAACCAGTCCTGAATCAAATAAATCAATAGGCACTTCCTCTTCCACTGAAGCTGCCTCCGCCGCGG
ATACTTAAGTTCCCGCCCCCGCTCCTGGTGGTTCTAGGTAAACAGGTGAAACTTAAACTGGAAAATAT
CTTACTTCTCAATCGACGAGGTAAACCATAGCCTCTCCTATAATACCCTTGCTACTATTGGTGGCTAT
GGATCTCCAATCCTTCCTAGTACTGGGCTTATAAATGACCTATAGGCTGCGGGCAATGGAACCATACG
AAGGGATGTGGCGGGCTATCGATCCCGATCAGTAGTTTACCCAGTTCCCTTTAACCTATTGATTCAGT
TGCTATCATGATAAAATGTGAAACTCCGGACCTGGCATGAGAGAAGAGTGCACCCCAGACAATCAATC
TTGAGAAAACGTACGTATCGGTTGCAGCTATAGGTATGCACGTTTATAGTAACAAGCTAGAATGTGCG
TAAAGTCGGAACATGGTAAAAATCTCGGAACACGGCATATATGGTAGTAAAAGTGCACAGTTCGAGGA
ACAACGCTATATAGGTAATATAGAGTATGCACTGTTTATATTATACAGTATGAAGCTAAGCCTAGCGT
TTGATTAATAGTTCACCAAACTAAAATGCATTGAGGCAACAAAACCAACTACCGAAACGACACCTTTA
TAGCCAATCGCACCGGTTGCAACTCCCTTTATATTAAGAAAATCGGGAGTAATTAGTCGTAGCACATT
CAAGCGCAATCACCGCTCATCCATCGATTTCAATGCCAATGCTGATCCTGGGTTATGGGTGAGAGAAA
TAGGTGGTACGTCAAGGCCAGAGAGCAGAGTTTAAGGATAGGAAGGCAGGAGGACAGGGCAACACATA
CGATCGGAGAAACAGTTGGTCGGGGGTACACATAGGGTCGGTCAGAACACAGACGAATGAACCACAGC
CTACAACCACAGGCCTAGCTCTCGAACGGCTACTGGCTCGGATATTGGCTATAAAACTGCAGGGAATG
TACTGTAACGGGCTATCAAAATGAAATGGGCTCTGCAGCTAGAGGATCTGGAACAATCTATAAAACAG
GAAGGGATGCCCTATTAATAAGCTCTCAAATTGGATGTGCTCCGGTTGCTTCTGGGTCTTTATCTTTA
TAAACTGGATCAAGGTAAACTGGGTCTCGATCAATCGGCTCAGATGTTGGTGGATAAAAAAGAGAAAC
TGATTCTTCGATTGGTTCAAATAAAACTCCCCAATTGGAGATGGAACGGGGGATTCGAAAACTCGATC
AATTGGATGGCGAATCACTTGCACCTGTAATGGGATCTGTAACTCGCTATGAATCTGCACCTGAACCC
GGATCTGCTACTATTGGCTGGCTGGCACTCGATCTCGAAATGAAATAGGTTCCACCTTATGAATGAGG
GGCTCCACCTTTCCTGTAGGGATTCGGGCGCTATGGGCGCTTATCTACGCCGATTTATTATATACGAT
AATTCAGTGGCGTAGGTAGATTCGATCCAGCCGTGTCTAATTAGTGCTGGCTGTAGGTGGCTGTCCAA

AAGAAAGGTAGTCTTGTAGTAGGCTGACCAAGTTCAGGAGGTGATCCGCAATGCTCACCCACTCCAAA
GAAAATGTCGGCAAATACGGAATTGATCATACTTGTAGAGGAGAGGTTCTATAATAAGGAAAAGGGGC
TACGCACAGGTATTACCAGAGGTGGCACTACCCAGAGGTTATACACAGAGGGTCCGGTGAACAGATAG
GTTATACAATTAGGTAAGCAATAACATACCCAAACCGAGTTTCGAACCGTTAAGCAAAACTGATTAGC
TTAGTTGAGTAACCGAGCTGAGTAGGGTATCCAAGCCCACCAGTAAAAGAAAAGAACCATGGGCCGGC
CCACCAAGATGCTGATAACACATCGCTAAATATACGTCGATTCGCAATTTGAGGAACATAGTCCTTTA
GGACTAAGGCATTTAGCCGGGTCAATTAGCGAGGGAAATACCTCTATTTGCCGAAATAGACTATTGAG
TATCAATCTGGTTCAAGAAGCGCATCACCGAATCCCCTAAAAGCCTGAGCTCCGCGTAAGTAAGGGAT
TTGTTCTGACTTTCAGCAAACGTAATTATATACGTAATTACACAGTCAGTCACTGATTGGGTGGATGG
AATGGAATGGAATAGATAGATGGGAAACGCAGAGCAGGTTTCTCTAGTTAGGAGAGGCTGGAAGGGCT
CGCTGCTTCTCTTATCTCATAGGAAGGAGCTCTGTCTTGTCTGTCTCATTATAGGAGAGCTGAACAAC
TTACACATTGGAGATTGATTGAGAGACTGAAAGGGACGCTATAGAGCTCCCTCTGTCCTCCCCTCTGT
CCGCTTTCTGATAGCAGAAAAGCGATCTATAAGGGCGGGCGAAGCGTGGAATGCGAGTTGGCTCAACT
AAATTAGATGTCGTGAACGCCAGACCAAATTGAGTGGTTCGGAGAAAGAGAATCTAAAGAATCTAAAG
AAAGAGACTCTAAAGACTCTAAAGAAAGAGACTCTAAAGATAAGATAGTTCCTTTCGGAGGAAAGAAA
GATGCTAACCGGGAAGGATGGTCTAGCAGCTATTTATCAGGCAGGTAACTTGAACTGAACTACCATCC
AATGAACTACCATCCAAAGTTTCTGCCCCTCGCTGAATGTCTCTCTCTATCAACCTGCCCTCCCTGAC
CTGTATGGTTCTGACTGTATGGCTCTTACCCTTCTGGCAATGAGACTGACAAAGACCTTAAGGCTTAA
TGCGACAATGACTGCTGGCTCCTTCACTGATAGGTAGCATCGGTAAGCATCGGTCGTATAGAATACGG
GTTAGGGAGAGCACGTGGGTAAAGCCTTAGCTACAGGAAGCACCCTAGTCCTAGTGTATCCGAGCCAG
TCTACTCTCCTGGTTAAGCCACTGAAGAGCTAGCAGTAGTAGTTTCTTCAATTGACCCAGCAGTTTAT
ATTGACCGAGTGAAAGCATAAGATCCAATTCTAGTCAAAGTTCCAGTGCCATTCATTGGATATCCAGG
TCCAGCAGCAAGGGCGGTAGAAGCAAAGATACGAGAAACATCCTCGCCTGTATAATCGGCATCCGGAC
CAGCACCTAGCACAGATCTATATCCAAATCCTAGCCTAGTGACAAAGTCTAATCCATTGAGTCGGTTG
ATCCCGCATAGGCACGGGCAAGGGTATCAAAGCCAACGAATCATGTTCCCAATCCAATCTCTGCTAAT
TCGTTTCAATTCCAATTGAAGCATTACATATAGATTGCATTGACCATCAACAGGAGAGGCAGCAGGAG
GAGAGGCAAGCATAGCTAAGGTGAAAACAGAAGTTCCAAAGCCAGATACAGATCAAGATCGACCCTCG
CCGGGTTAGAGAGCCGAAGTAGGGAGTTTCACTAGCAACAGAAGGAAGGGCAGGAAACCTAGGTTGAA
TCAGAACCGCTACCAGCTGCAGAGGCATTTCCAGGTGCTTATTGAGTTCCAAAGCCTATTCATGTTCT
GGGTGAAGATCCAAACCCAGATCCCGTGCTAGTTGGAGAGCTACCACCTAACCCAGCATCTCTTTCAT
TTCTAGAACCAGCATCAAAGCAAGTACAAGATCAATATAAAAGTAGCGATCGATGCACTCGATGACAG
GTGGGCAGCCAGCCCTGCTCCTCCCTCCCTGTTGTTCCTTGGTCGTGTGTTGTTCCATAGCAGCGGAT
CCACTCAAATCTGATTGCTTGCATAATGACATAGCTCCCCTCTCCCTAACAGAGACGGCCATTCAGTC
CATTCCTTAAATAGTTGTTCGTCAGGGTTCCACGCTCACTTACTAGTGCTTTCCTCCTGCTTTCCTCT
GCTTTCAAAACGTCTGCGCATTTGATTAGGGCACCCAATATTCTATGCCGAGAGAGATACAGAAGTGC
TTGTTTATAGCCGCTAGTACTTGCCGGGAGTACTCGCTTGAAGGAAAGTCAGTGGCACACATAGTAAG
TTCTCATTGCCGGTTCTCTTTGCTGATAGCCAATAGTTGATACAGGAGTCCCGGCACATAAACTGGGT
CGATGCCGGGAGTATTTGCCACTAGTTGAGGTCAGGATTCGATGCCGGTACTCGATCCAGGTGTAAAT
GCACTCAATGCCAGGTGCCAATACCAGGTTTCCATGCACTCGATCCAAGAGTAGATCCCAGGTTTCAG
TGACAGTCATAGCCCGTAGGCAACAGTCTCATAGACAGTCAGTGATAGAAATAACTCCGGTCAGTCAG
TCTAGAATGAAGCTCTACTCAGCGAACCTATACTCAGTGCATTAAGCTCTAGTCTGTACCTAATAGTA
CGTGTGTGCCCACTAGTCATTCAGGAAGCGATGCCAGGACTCGATGCTCCTACTCGATCAGTCGATGG
ATGCCAAGAGTCGATGCTAATAGTCGATCCAGGAGTCAGTATGAGCTAGAAGGTAAGGTGCAGCATGA
AGCTATGGTTAGTGCAGCTATAAACCTCTGGTCAGTGCCAAGTACTCATTTCCAATCGATTGATCATA
GCCAATCTGAGATACCCAAAGGTGCTGATAGTCGGTAGTCGTTTAATGAAGTCTTAATTGCCGGGATT
CATTGCTGATAGTTGATAGTCGCTAGTAATTGATGAGTTGGGATACTCAGCTACTCAGTTGGGCTTGG
TTACTCAGAATTGGATACTCGGGCTGGGCTACTCTCTCTAGACTTGGACATTATACTCAGCTACTCTA
CACTGGGGTAGGATACTCTACTGGATGGAAGGAGCTCCCGGGATGGCTGCTGGGGGACATTGATTCTG
TGAACGGATTCTGGGACATCGCCTCTATGCTCTAATTGCAAGTGAGTTTCCCATGTTATCACCGGGAG
GGAGGGACGCACGGGACGGTACGGGGCCCAATGAGTGAGTTAGGCACAGACATAGGGCCATTCCCTAT
AGCCAATACCCAGTAGCCAATAGATTGATAGCCGGTAACAATTGACGGTAGCAGATAGACAGTAGACA
ATACCCGCCCGGTAAAAATTATCACCTTATCTGGGTCGCGGTTGATTAGGTTATCCCTAATGAAGGTT
ATCCCACATAGTATGGATCCTTGCCTGGATTAGGAAGTGGCTGGATCGCTTCGCTTGCCTGGACCTAT
CCTTAATAGTAGAACTGAGCTCTATATACTTGAGGAATTTCTCTTTGAGTGGAATAAGGTTAGGTGGG
GCTGATCGATATCGCTTGTTGTGGAAGGTCAGGTAGTCAGGGAAGGTGCGTCTACTGAACCACTTTTA
TTAATTCCTGCTTTCTCTCTCTTATCTCTATATCAAACTTATGTGATGCTGAGGGCGTTTCTTCTATC
TTCTCTCTGGTCCAAACTGTTGAACCAATATTAGTATTATTATTCCCAGCCTCAGTTCCCGGATCACC

ATTTGAACCAGTCTTATTCCCAGCGTCAACAGTTGAACCAACATTTACATAATTAGTCGAAGGGGCTC
CCAAGAGCAGTTTAACCAACGGCATTCCCATCAGCAGTTGAAAGAGCATTCTTCCCAGGATAAGTTCC
ATCCGCATTTGCATCATCAGTCAATGGAATTATTTGAATAAGCTGTTTCAGGAGCATTATTCCCAGCT
TAATAAGCACCAGTCGAATTAGCATCAATTGCAGGTCTAAGAGGTTCGATTTATCTGTCCCACCACGT
CCCCGTTAGGGCACTGTAGGCAGTCGTTCGTCCTTTGGACGCGCCACCCACCCACCCTACATCTGTAG
GTCGTAAATCGAAAAATAACTCTCTGTCTAAATCGTTTTAAGGCCGCAAGATCTCGATCAGCATGAAG
TGCCGCCAGGGGGCGAGGGAGAGGTCTATGGTTCCGTAGAGTTAGAGGGTTGAGGGTGAGTTCGAGGG
CTAGAGTTCGAGGGCTATGTTTCCTACATCGAGGTTCTATTTCGAGGAATAGATCTAGGGTCTAGGTG
GGGGTCTAGGGAGATTCCGTAGCGTTTATCTGTGTTTGTCCAGCCGATGCAAGCAATGACTGTGTCCA
TCCCGGCTATGTGTTTCCAGCCCAGTTTATTAGAGCCCAGCCCGGTAGCGGAGCCCAGTGTATTAGCC
CCAGTGGATTGGATCAGCGGGGTAGCCTAGCGTATTGGATCAGTCAATTAGCCAAGCGGAGTAGCCAA
GTGTATTAGCGGAGCCCGGTGTATTATAGCCAGGGGATTGGATCAGTGGATTAGGCCCAGCAGATTGT
ATCGGCAAATGGAATCAGCCGGGTAGCGATCCGAGTCGATTAGCGTAGCGGATTAGAGCCAGTGGGAT
TAGAGTCTAGGGGATTAGAGGAACCAAACCGAGTATCCCAGCAAATCCCAGCTATGACTGACATCTCC
ACCAGGCATCAATCTCTTGGGATCAAGTACCAGCATAGAGTACCGGCATCGAGAGTGATCGCCACCTG
TATAAAGTACCGGCTATTTAAACCTTCTGTAAATGACTACCTCCTTACTGCTAATGAGACTGGCTATG
AGTGGAAATGACCTTAAAGCAATGACAAGAGAGTTGCACTGACTGAGTAGAGCTTTCCAAGAGTAGAG
CTTCTTTCTGTTCTGGCACTGAGAAGAGCTTCATGCACCAACTAGAGCTTCTAGGCCTCCATTGATAG
GCATACTTTATCGGGTATGTTTGAACGGGGTGAGGTACGAACCCGCTCCCCCGACCAAAGAAGGGGGA
GGGGTCTTTGTTTCTATTTAGGGTTTGTGTTTATGGTTACTCTCACTCGGATCTGTTGAACAGGTTCT
GTTGTTTGAACGGCGGGTCTCCAATGATGGGTACCTTCTATTGTGGTCTGGCGAAAAGGACTTCCCTG
TCGTCTGGCGGCGGCCTTCCGTCTGTTGGAAACTAGGGGGTCAATTTCTATTCTGGTGGTCACATGGG
GGCGATGATAAAGCAAGCGAACGTAGGCAAGTTATTAATTTCCAGGGATGTCAAGATCGCTAAAGCGA
GCTAACTTCGGCAAGCCAAAAGTTCGATCAATAAGCAGGCCAGGTGTAAGGGTAAGTGGGCAAGGTCG
ATATAAGTCTGTAATCAATCTATCAGGCAGCTGCCCTTCTACCCGCAATCAATTTAGCAAGCAGCTTT
AGCTCAACCTAAAAGCAAATGATGCCACCATCCCGGCTATCATCGGCAATGACACCTGGCAATGACTC
CTATCTATGATCAACTATGATCAGCAATGATCGATCGGCTCCTTCTGACTATGATCGACTGTCTATGA
CTGACTACTGGCTATGACTATAGCTTCAATGATTAGAGCTTCTAGCAATGACCTTGTAGCAATGACTG
GAAATGACTGTCTCCTGCTATGACGACTGGCAATGATAAGCTCTCGGCACTGACCTTCAGGCAATGAC
AACTAACTGGCAATGAGCGTTTTACTATGACAACTGCTCTGACTGGCAATGATAAGCTCTTGGCTTTG
ACCACTGAAAATAACCTGGCTCTGACCACTGGAATGACCTTCTGCCAATGATTGGCAATGACTGGCTG
GCTATGAAAACGGGGCTATCAACTGGACTATCGGCGGCAATGCCCTTTTGGCTAGTACTCCTTACTAT
TATCGTCAATGTCGGGTGCTGATTGTGTGAGTCTGCTATGTGAGGTGCTCTATGTGCTGTTTCTGTAT
AGGTGGCCCGGTCGTACTTCAAGGGCGGGTGCCTGCGGACTGACTATGGCTTACGCGGACTGATACTG
TGCGTACAGTTAGCTCTAGGCTCGAGCAGGCAGCAATAGAAAGGGCAGGTAGGTGAGAGTGTGTCCAA
TTGTGTTTTTTTCCACGTCCCTCCCTAGGTCTAGTTATATCGTAAGTAAGGGATATACCTACCTTCGG
CAAGAAGCAAGTAGGATCAGCAGGCAGGTGCGTAAAGTTGTCAAGTTCGAAAGAAGCCCGTCCTCAAT
CTAGCAAACAGCGAGGTTCCCGCGGAGCCTAAAAGCAATCGACGTGATCCTGCCTTTCATGGGTAAAC
TTTATGAAATGCCAATATTCAGTAAGAATAAAATCTACTTTATGTGCCCCGATGAGATTTCAAGAAAG
GCGTTTTTATTAGCAAAGAATGTTTAATGAGGCTGTTTTCTCTAACCGGTATCAAAGCCAAGTATTCG
AGCCAAGCCTAATAAGGCAGCAGCCACAGTTGAGTAGCCCAACCGAGCAGTCCAGCCCAGCAGGTATA
AGCATTAAAATGTTGTTCCGCCATAGCGTTAAGTGAGTTCGAGTTCTCTGTAGGGTATATAGGTCTAC
GGTTCCGTAGAGTTTAGGTCTAGGTATACTTCTATGATTGGCTGCTCTAGGCTCATACTACGGTTCTA
TGCCCCTCATCATCCTTCCACCATGTAGAGCGGCGAGAGTGAATATATGAGCGTAGCAGGCAGAGCAA
TGAATCTATAGAGCAATAAAAAAACAGTAGTGTCAGTGTGCGCATAGCGATAGTGTGTCAGGAAAGAG
CGTAGCAGGCAGAGCAATGAAAAAGAAAGAAAGATCTCCATATGCCCCCATACAACTTAATGAGCTGT
ATATAGCTCTTATTGGTGGGATATGATGAGCTATAAAAGTGATGATATAGCCTATTAATCTTATCTTT
TCTTACATAGATATAGCTATCTTTTCTGATTGACGGGGGCCCCTACCCTATCCCCTTTTGCAAGCTAT
TGACCCTGCCTTCCTCCTTCATCGAACGAATGATCCCCTAAATGCCCACCTCCTTTATTAGCGACCCC
CTGTCAATGACAACCCACCTCCTTATCGTTGACCACCGGCTCTGGGCTGACCTTCTGGCATAAGCTAT
GTGACTTTTGTATTCACTTCTTGTGAATTAATTAATGTCTGTAACACATCTTATTACTTACATTGTTC
CGAACCGAGTAGTAGGCCCCGGCCAAGTATCTGCTGAGTATCCCAGTTGAGTAACTAAGCCCATCGGA
GTAACCAAGCCAAGCAGCTGAGTAGATCCCCATTTGAATAGCCCATTGTTGTATTATAACATATATAA
TGAGCGCTGACAAAGATGAACCATCTTACAAGAAAGGTGTTACCCCGATTACCCTGATTCCCCAGTCC
CGCATTCTATTATTATTGCATTCCCGCTCTCCCCATAAGGCTCAGTACGCGTAAGCAAGGAGGATACC
ACCCGGGCAGGACACATATTGTCGAAGAAGGAAGGACCAGTAAATGAATTGAATTTGATACCTGCAGC
ACCCCGGCGGGACTAATATGCCGGGGTAGGGAGGTCTTCGAAATGAGTGGACTTCCCTTCCGTTCACC

CGAGCCCGGCCTAGCAAGCCTTCGCAAGCGTTATAAGGTAAGGCCCGAGTGGGCCCGGAAAGCACATG
ATTTTAGAGCTTTCAACACCCACTATCACAATCCTGCTCGAGCTACTTCTCTCTCCTGACCACTGGCA
CAATCACCTTACCTGGGAGCATGGGATCCACTGGTTAGGTAGGCGGGGCATGGTATCTACCCACTGAA
ACCATCCATCCTCACTCTCACTTACCCGGGGGGAACAGGAGGCACGAAGTTCTTTTATTGCTCCAGGC
CATAGCGTCGCCTATCAATTACTTCTTTGGGCCGCAGGAGGCTTTTTCTGGCAAGAGTCCTATTAGAA
GAAATCCCCCTTCATGGAGACGAAGTAGGGGGGCTCTCTACCCCACCCGTATACCACATTGCATACCA
AAGTGTAGTAATTCTCTACTCTCACACACAACACATAGTGGGAAAAGTATGGACACCTAGCAACATGG
GATGGGGGAGGACCTTTACCGCCCTTTAAACCTGAAAGGCAAGGCAAGCAAGATCTGTACCTGTAGGG
GACAGTAGATGAAGACCTTTACTTTTTAATACTACCCACCTGCCATAAATTATTCTATTGAAAGCCCT
AGCTACATGCTTCCCAGGGAATGAATGCTTGAAGACCTTAGGAGCACCAGATATCGTTGGATCGGTGG
ATGGGATTGGGATGGGAATCAACATGGCCATGGGAATCCGTTCATTAGCCCTTACCTTGCTCAGACAA
TACACCAGAATTACCTTTCTATTAGCCCTAGACCATGCTGAAATGGGAATGCTATCAGACCTGTATCC
GGATCAGATCGATGCGATTATGCAACTGGGATGAGGTTCATTAAGCCCTTACCTTGGAAATACAACCC
ACCTGCCAGACAGACTTTCTGCTGCCCTAGATTAGGAAGTCTTACCAGTTACCAGTTAGACGAGTATC
AGACGAGGGGAGGTACCACTAGCAAACTACCCTACAAGCAGCGACCTAGGACGCCAAATTGATTGCCA
TGGGTTCAGTCATTAGCCCTAGACCTTTGAGAATATGCTACCTTATCCTTAGACACCTTCATCACCCC
AGTCAGTCAGTTATAACTACCCTAGAACTCACAGGCTTAACCTTTCTCTTACCCACTACTTAGTTGCT
TACAGGACCCATACCCTTCTCTTTCTTTGTTATGACAATACACCTCCTATACAGAAGGAACACTTTAT
CTGCCCTAGAACTTCAGCTTAGCTACAGCCTTATAATGCGAGAAGCGGCTACAGTATAGTGCGTGGTG
CGTGAGGAGCACCCTCAGTATATAGACGCGCACCAGATTTGTTGGGTTCATCAGCCCTAAAACCTTGA
GAATTGTACGGTCGCCCAGAGCCAGTCGCCAGGAGGTCTTTTTTGATAGCCGCTAGTCGATCCAGCAG
TCCATGCCAGGTGTCGATGCAGTCAATGCCTGGTGTCAGTGCCAGTCAGGGAGGGAGGTGGCTGATGC
TCGCCCGGAGAGATCTTGATTAATTGCCTACAGATTCGGCCCAGGCACCAAGTTGGCTTGGAACAGAT
TTCACAAAACCTTCTCATTTGACATTCTTCTTATGCTCCGCACCTCGGTTTTAGCAGGTCCAAGCTTG
AGTGATCGGGGGTACTAAATTGGATTTTTTTTATTACCGAAGATTGGCATTTCTCGGTAGTAGATACA
ACAGTAGATCCAACAGTAGATGCACTCGATGCACTCGATGCCAGGAGTCGATCCCGCTAATCGATCCC
GGTACTCAATGCTGGTAGTCGATCCAGGAGTGTCGATCCAAGAGGCGATCACTCGATGACAGGTGTTG
ATTCACTCGATCCCAAGAGTTCAAGATGTCGATGTCAGGAGTCAATTTATTCTTGTCTGGTTGCGAAC
CGATGGAGACGATCTTACGATTCACCTATCCTTGCTGGTTAGCTATCGTTTGCTTGCTGACTAGACGT
TTGGATAGACGAGATAACCCGAAGAAGAAGCTGGTCCAACAAACGCATAATGATAGAACCTGTGGTGG
AAAAACGTTAGGCTGGGACAATAACCCGGCTGGCGGGAATCGAAAGCTAGCTGGTGGGCTTACTAACT
GGGATGGTTTTTTTGCTTGCTGTTCAATTTGAAAGCCTAAAACCCGTCCTTGCACCCTATGAAGAAGG
GCAAAACCTATAAGAACTATTCAGCAGAAGGTGGCCTATCACTTAGTCAATTATATTCCTGCCTGGAA
ATTGGCTAGTGAAAAGGATTACGTATTAAGGGCAAGACCTGTCAAACCTTCCCTTAAGCGGAATTTAA
GAGTTAAAAAGTGGCCTATCAGTTGGAAATTCTACGCCTTGTCAACACATGGTAACTTCACTTCTTTT
AACTTAGCCTATGGGGGTGGTAAATTCCTCTCTGTTAACCTGGCCTATCACTCATTTGATTCCGGGGC
TTGCTCTGTTTCGTCATATGAGGAGGGTAAGGCAGCAAATATAGGCGATCCTCGCTTCTTTGAACGGT
TGTAGCCTATACTGGTACCGGTACTGGGGGAATGCAGATACTTCCTCCTTTTTTAAGCCAACAAAACC
CCGGAATACCGAAGAAAGCGAAGGACAACGCAGCGGGGGGAAAGATCATCCCAGAAGCAAACCCTGGA
ACATTGTGAGAGGAAGGACTCCCTTAAGCCCAGCAGCATTGGAAGATCCAGAGCCTTAAGCCCAGGAA
GGAGGGCAGGAAGGCGCCAGCCTTAAATGCATTAATTTCAGCGGCTTACTAGATCAATTTTGAGTTTG
GGACCCACATGGATCATATTACTTGGAACCCGGATAAACACAACTTAGATTTACCCAATACGCGGCGA
AGGTTCTAATTGACCGGAACCACCCTAACAGAAACAAATAGCTTTTCGGGTCATAGTTTCTGGCTGGC
TCTCCCGCCCCAAGCCAATCAGGTTATCCCTGAATTATAAATATTTTTTTGGGGGGAGGCACATAGAA
TAAGATTTTCAGTATTTAGCAGACTTTAACGCTCCCCTATTATGGGTTTATTCCGTGCACCCGGTCCC
GGGGTTCGTTTCATAACTTATTATTTATCCCAGCCCGGATTCAATCAGCTATTACCCAACTACTCGAT
CTATCTAACTATTAATCGCTCCCCCTGCTATAGCGATAGGCTCTTTTGGACGTCCTGTCCTTCTATGG
GTCGGCGGCTGGCCCTCTCTTCATTCGCTCTGGGCTTTCATTCATTCATGACCATTGCATGCGGCTAT
CCCTATCATTGATTAATATAGATAGAGGGAGGTTTGCAACCTCGGCCTCGGCTTCATTCTCTCTTTCT
CCATCCTTACTCAGCTGGCTTCCTAAGCTTGCTGCCCGCTCTTCAATCCAAAGAGCACAGAAGGTTTT
TGCATGTGTTACGCATATGGATAGATAGCCCTAGCCAGCATATAGCTAGCGGTGTTGAGCCGCGTTCG
TGAAAGAGATTAAAGACGCTTTTAATATGACTGACTTAAACGAACTATAGAGGAGCGACTTAGTACTG
GCTCCCGATACACGCTATTCAAGCTTCCATGACAATGACTCGGTGACCTTGGAACCTTTTATATAGTT
CCCGCCCACCCCTTCAATTAGGCAGCGGACTAGACGGAAAATAGAATCAAAGTATTTAATTAGAGCCC
TCTGGAATAGGAAGAGCCTTTCATTATTTATAAGGTCCGAACCCGCAAGATCTGTAGAAGCCAACCGA
TGTTAATTCGTCTATTCAAGATGGCAAACTATACTTCTGTTCCAGGTCGTGGCCTCCTATCCCCCACC
ATTTCAAAGGGCTTTTTTAGGGGTTTCGGGCTTTCTTCTTTCCGAACGATATTCTCAAATTCTTAAAC

CTTAAAATAGAGAGGGAGGGCTCTGGCTCCACGAAGAATTCTGCTCCGTTCCTTCCCCTCTTAAAATA
GAGAGCGCCCGGTCCCAGTTATTCCCCCCCCCATGGTCGCATACCAAGGGAGAACGGGTATGGGGTGG
AGGGATTCGGGCTTCTTCCTAACCCTATGGGTGGTAGATAATAATTCTTTTTTAGGCTCCGTCCATTT
CAGATGCCCCATACTCCTTAAAAAGAGAGGGGGCGCTCCTCACCCTATTATGTCGGGTGAGAGCTTTT
CGCTCCTCTCCCCGAAGAAAGATAGATGGGTCCGGTTTTATATAGGGGTGACTATGGCCAGATCTGAA
TGCAGATCGAGCGAATTCGATGGATATATAGGAATAGGAGAGATTCCGGCTACCGAACTCCAGGTTAT
TTCGCTACCAAACCGGAGGGGGGGAAGTGGGCTTAGTAGGGAACTATCTAGTAGATACCCATTTATCT
AAGCTTTAAGCTCTAAGCTCTAAGGCGGGCGGCGCAGAACGACTTACAGAGATACCTTTTATTTAAGT
AGTTGCCTTTTGTCCAAAGTCCCCTGCTCAGTAGGAGTAGCTCGGTGCCCTTCGGAGAACACCTCATG
GATCTGGCTCTCCTCATGAAAGAACAAATCTATCTATCCAGTCCCTTACCGAAAAGGAAGGATCTCTC
ACCTAATAGGAAGGATAAGCTCATATATAATAGCGTGTGAGGGACGACCCATGCCTGTCCAAAGGGGA
TACGTTATAGGGAAGACCCTGCATTGCTGCAATCTAGCTGGAGCAGTCCTATTGCATTGACTAGGGGA
CATGTAGTGGTGGAAACCCGGGTAATGGGGCAAATCGGTAATGGGCTACGGGAGCAGAAGTTATGGCA
AACCGGGATTGGCTACGGGACCTGTAGTGGTGGAAACCCGGGTAATGGGGCAAACCGGTAATAAGGCA
AACCGGGATATAGAACTGACCAGACCAGAACTGACAAGACAAGACCTGAACTTACCAGGCTTCTGGTC
AGGCACACGATGACAGACATCACTTTTATCAGTTATTATACCATGTAATTGACTCGTATATAATGGAT
AACGACTCCTTGGCTAACGACCCCAATTACGGAAATAGAAGAGCCATTACCTGAATACCTTACCTTAC
CTGAAGACCTTACCTGACTTTACGGCGCTCACTAGCACCGACAAGCACCCGCTAGACCGACAAGCACC
CGAAAGCACGACAAGCACCCACTAGCGCCCACTAGCACCCACGAGCACCGACCGACTAGCACTGACCC
GCTAGCACTGACCCGCTAGCACCGACGAGCTCTCCTTCCAATAAAGAAATCCCATTAGTCACTGGTGT
AGAGGGAAGACCCAGATTGATGCTAGGTGGATCGTTGATTTGTAGAGCAGTTATGAGGGGAGACCTTT
ACTTAATGGGGAATATGAAATCTGGCTTCCTTTCCTTAATACGGACTACGGAATGTGGGTCGGTGGAT
TGGGAATACCCTGATTCCTGCTATGTGGGTTATCACTTTCTATAGCATTTCTCTAGGGTTGACTCGTA
TATAATGAATGGATCTAGTCCCACCCTCCTTGAGTAGTGAGTTTGTGTAGGTTATCACTTCTTTATAG
CGGTGCGATGGGAGCAGTTCCAGTTCTATAGCATTTCTCTAGGGGAGTTTATGGAGGGAAGACCCTAT
GAAGAATGAATAAAAAGGGGAAGGCCCAATTTGTTTGATAGGATTGGAGCGGTATTTTCGGCCAATCG
ATCAGATCCTTCTATATACGATACGTTATTTCCCATCGCAGGAGAGGGCTAAATAGAATCAGCAATTC
TATATGGATCAGCTAGAGGGGGGAAGGAATCGAATCTCGGCCAGCAACGGTAGCAACCTTCATCCGAG
CACACATACTGAACCTGGAGAATGGGCATCCTACATAGCCTCGTTGTCCGCATGGTACTTCCGTGCCA
TTGCATTGCGGGTTGGTCGTGTCATTGCTGCTGCAAGTGCTTATAGGCTCAATCAAGATTACATACAG
ATTGCTTGATCCTCGTCTTTTTATGAGGAGTTAGCTGCGCATTGCATTGTGGCATGGTCGATTGTTCC
GTTCGCCCCGTTCGAAGGCCAACTCAACTATATATCAATCTATATAGTCCTTCGTTAATTCAAACGTA
GGGTCATGCGCTGCGCCAATTGAATTGATAAGTAGAGCGCTGGGCCGCCTTGCGTTGTAGCATGGTCT
TTCTCCCCATCGCAGTCGCTCATTACTCCATTTGAGGCCTGATCGATATAGGGGCGGCATGCGCTGGC
TTTTCATGCATGGTCGGTTTGTTCCTTTGCACGCACGGTGGTGGCTTGATCGATTGATTGATCCGATC
CTTAGTAGCACCATAAGTACACCACTGGAATAGAATCCCTGTCCTCCATGACTGGAATAGAATCCATG
TCCTCCATGTTAGTTATGAGTGGATCTGCTCTAACCTTTGGCGAGAGGAAGGAGGAGGAAAGAGGATA
CTCCCTCAGTCAGTCAGTAAGGGTAATAATCCAATGGAAATAAGCCAATGGGACTTATACTTCCAATC
CAATGCAATCCGATGGGGGAAGATAAAAAGCGTAATTACATATATAATAATATATTTATTAAATTGGC
TTTGAGGGGCATCAATATGGGCGTTGGTCGAGCATCTCAAGATCCTCAAATAGCTCTGCGTAAGGGAG
AACCTTACCTTACTTAGAAGGAAGGGGAAGAAA
>Repeat_17
TCACAATTCAACCGCGTCATGATATCTATGTCCCGCTAATCAGAGTGAATCTCAATGTCCCACATAAA
GAATAAGCCGTGTAAGTAGGGGGTCAGTTCCAGTCATAGTTTGAGATTCAAAGCCCGTTGATCCACCT
GTTCAAGTTCGATAGCCCAATCCCATGTTAGTAAAGTAAAAGCTACACGCCAGGAAAGAGAGGGCTTG
CTTGCTTGGTTATTCCAGTAAGTGCCTTTGCCGAAAGAGAGAATGCCTTTACCGGAAGAGAAGAGCAA
CGGCCGGGGGGGCGTAAAAAGAAAAGGGAGGGGGTAAGTTCCAGACGTAGGTCTGCTGTGTCATTCGT
CCTGTCGATGTTGGGCTATATCCGGTAAAATAGTTTAAATATTCTCCATAAGTAGAGGTAGCATCCTT
TCCAGGTTGATAGCTAGGAGCATCCCTTACAATTAGGAAGCCATAGCCTGGTGTTCTGGTTCCCATTC
CATTACCAGTCCTAGATCCTACAAAACCAATGGATGGATCCCGATCTCGGTCAATTGAATCTGGGACT
GCTGGATTTGCATATGCATCATTCACTGCTTATAGGGAAACTCAGTACCCTGGTACGGGATCCCACTC
TGGAATAGGATCTTAGTAAACAAGATCAACTGATTCAACCGCTGCTTTCGGTGCTGCTACCTCCTCTC
CTGTGGGTGGTTCCAGATGAACTGAATCAAGGTCAATTGCAGCTGCTACCCCCGCCTTAGCTAATGAT
GGTTAAACGGTAGCTATCTGAATCTTCGGGTTATAGGTCCGTTCGCTCTGAATCTGAACCTGGAACTG
GGTCGCTACTGCTGCTGGAATTGACTTTGCAATGGCTGGTGCCTATATGCCTTTGCTTTAGGTGGATC
AACTGCTGCTCGCTCTAGCTACCTTTGCATCTACTGCCTGTGATTCTGCCTAAGCTGCTGTCTCGGCT
GCCTATGCTGGTGATGAATCAACAATATAAACCGGATCTGCTGCTGGAACTAGATCTGTAGGCAAAGG

AGCTAGAGCTTCAACTGGGTCAACTAGTTCACCTTACAAAATTGGGTCAACTCCAATTGCTTCAACGA
GGTAAACTGGCCCTGGAACCGGATCTGCCGTTGGGTCTCAATGTCGATCTAAAAATTGAGATGAAATG
GGCTATGGATATAGAGCTAAATCTATCACTGGTTTTGGAATGGATCTAACCCTTGCCTTGCTTCTGCT
AGCTCTCGAACGGGCTATGCTACCTTTATAGAACGGACATATAAGAGAGGGCTCATTTATAGATAGGA
AACGCTTAACCCGCCCGCCGATAAGAGGTTATAAAAATTAATTGATTTGGTTGGTTTGGAGGGACGGA
TGAAGATTAGTTACTACCTTGTTAAGGTATTAAAGAATATGTAGCTCTGGAGGGAAAGCTCTGAAACT
CAAAGGATATTACAACTATATTGGTGCTGCTTCAACTCGATCAATTGAATGATATGGAACTAAAAGTG
ATGCTTCCGCTGATGGCTATGATGCTGAAACGGGTGCTTCAATAGGTGAAACAAAAACTGCCTTTGAT
TCTTCAATCAGATCAACATCAACTGAGTCAACGGGGTATGATTCAACCTAGGCTACTCGGTCTCTATT
AACTGGGTATCGATCTGCTGGGTCTGGATCAAGATCAACTGGAACAGGCTATTAATTTGGAACTGACT
TTGCAACTGGTTATGGGTGTGGCTCCTCCGCCCTGCACTCCTTTTCCGGAATGAGTCACCGGAAAGCG
AATGCACCTAACTTATGGGACCTTGGGGACACCCCTTCTAGGATCACTGTAAAATGCGAGCACTTCTG
AGTAGCCTAATGTCGGAATCGTATCTTCTATAAACAAATAGAGATGCCGGGTCAACTGCTTTCACTCG
GTCCATGAGGTAAACTGGTGTCCTTGCAACCACTGCTGCTACCTGTGCTACTTTTTATGGATAAACTC
GGTAAACTAGTGCTTTTTCCACCTTGGCTATGCGCTTTGGATCTTTAACTGATCTCGATTGTGATTGA
ATCAACTGACTCTGGATCTAGAGACTCTGGAACCGGGTAAACTCAATCCCGGGCTATCAAACGGTAAC
TGTAACCAGTCCTGAATCAAATAAATCAATAGGCACTTCCTCTTCCACTGAAGCTGCCTCCGCCGCGG
ATACTTAAGTTCCCGCCCCCGCTCCTGGTGGTTCTAGGTAAACAGGTGAAACTTAAACTGGAAAATAT
CTTACTTCTCAATCGACGAGGTAAACCATAGCCTCTCCTATAATACCCTTGCTACTATTGGTGGCTAT
GGATCTCCAATCCTTCCTAGTACTGGGCTTATAAATGACCTATAGGCTGCGGGCAATGGAACCATACG
AAGGGATGTGGCGGGCTATCGATCCCGATCAGTAGTTTACCCAGTTCCCTTTAACCTATTGATTCAGT
TGCTATCATGATAAAATGTGAAACTCCGGACCTGGCATGAGAGAAGAGTGCACCCCAGACAATCAATC
TTGAGAAAACGTACGTATCGGTTGCAGCTATAGGTATGCACGTTTATAGTAACAAGCTAGAATGTGCG
TAAAGTCGGAACATGGTAAAAATCTCGGAACACGGCATATATGGTAGTAAAAGTGCACAGTTCGAGGA
ACAACGCTATATAGGTAATATAGAGTATGCACTGTTTATATTATACAGTATGAAGCTAAGCCTAGCGT
TTGATTAATAGTTCACCAAACTAAAATGCATTGAGGCAACAAAACCAACTACCGAAACGACACCTTTA
TAGCCAATCGCACCGGTTGCAACTCCCTTTATATTAAGAAAATCGGGAGTAATTAGTCGTAGCACATT
CAAGCGCAATCACCGCTCATCCATCGATTTCAATGCCAATGCTGATCCTGGGTTATGGGTGAGAGAAA
TAGGTGGTACGTCAAGGCCAGAGAGCAGAGTTTAAGGATAGGAAGGCAGGAGGACAGGGCAACACATA
CGATCGGAGAAACAGTTGGTCGGGGGTACACATAGGGTCGGTCAGAACACAGACGAATGAACCACAGC
CTACAACCACAGGCCTAGCTCTCGAACGGCTACTGGCTCGGATATTGGCTATAAAACTGCAGGGAATG
TACTGTAACGGGCTATCAAAATGAAATGGGCTCTGCAGCTAGAGGATCTGGAACAATCTATAAAACAG
GAAGGGATGCCCTATTAATAAGCTCTCAAATTGGATGTGCTCCGGTTGCTTCTGGGTCTTTATCTTTA
TAAACTGGATCAAGGTAAACTGGGTCTCGATCAATCGGCTCAGATGTTGGTGGATAAAAAAGAGAAAC
TGATTCTTCGATTGGTTCAAATAAAACTCCCCAATTGGAGATGGAACGGGGGATTCGAAAACTCGATC
AATTGGATGGCGAATCACTTGCACCTGTAATGGGATCTGTAACTCGCTATGAATCTGCACCTGAACCC
GGATCTGCTACTATTGGCTGGCTGGCACTCGATCTCGAAATGAAATAGGTTCCACCTTATGAATGAGG
GGCTCCACCTTTCCTGTAGGGATTCGGGCGCTATGGGCGCTTATCTACGCCGATTTATTATATACGAT
AATTCAGTGGCGTAGGTAGATTCGATCCAGCCGTGTCTAATTAGTGCTGGCTGTAGGTGGCTGTCCAA
AAGAAAGGTAGTCTTGTAGTAGGCTGACCAAGTTCAGGAGGTGATCCGCAATGCTCACCCACTCCAAA
GAAAATGTCGGCAAATACGGAATTGATCATACTTGTAGAGGAGAGGTTCTATAATAAGGAAAAGGGGC
TACGCACAGGTATTACCAGAGGTGGCACTACCCAGAGGTTATACACAGAGGGTCCGGTGAACAGATAG
GTTATACAATTAGGTAAGCAATAACATACCCAAACCGAGTTTCGAACCGTTAAGCAAAACTGATTAGC
TTAGTTGAGTAACCGAGCTGAGTAGGGTATCCAAGCCCACCAGTAAAAGAAAAGAACCATGGGCCGGC
CCACCAAGATGCTGATAACACATCGCTAAATATACGTCGATTCGCAATTTGAGGAACATAGTCCTTTA
GGACTAAGGCATTTAGCCGGGTCAATTAGCGAGGGAAATACCTCTATTTGCCGAAATAGACTATTGAG
TATCAATCTGGTTCAAGAAGCGCATCACCGAATCCCCTAAAAGCCTGAGCTCCGCGTAAGTAAGGGAT
TTGTTCTGACTTTCAGCAAACGTAATTATATACGTAATTACACAGTCAGTCACTGATTGGGTGGATGG
AATGGAATGGAATAGATAGATGGGAAACGCAGAGCAGGTTTCTCTAGTTAGGAGAGGCTGGAAGGGCT
CGCTGCTTCTCTTATCTCATAGGAAGGAGCTCTGTCTTGTCTGTCTCATTATAGGAGAGCTGAACAAC
TTACACATTGGAGATTGATTGAGAGACTGAAAGGGACGCTATAGAGCTCCCTCTGTCCTCCCCTCTGT
CCGCTTTCTGATAGCAGAAAAGCGATCTATAAGGGCGGGCGAAGCGTGGAATGCGAGTTGGCTCAACT
AAATTAGATGTCGTGAACGCCAGACCAAATTGAGTGGTTCGGAGAAAGAGAATCTAAAGAATCTAAAG
AAAGAGACTCTAAAGACTCTAAAGAAAGAGACTCTAAAGATAAGATAGTTCCTTTCGGAGGAAAGAAA
GATGCTAACCGGGAAGGATGGTCTAGCAGCTATTTATCAGGCAGGTAACTTGAACTGAACTACCATCC
AATGAACTACCATCCAAAGTTTCTGCCCCTCGCTGAATGTCTCTCTCTATCAACCTGCCCTCCCTGAC
CTGTATGGTTCTGACTGTATGGCTCTTACCCTTCTGGCAATGAGACTGACAAAGACCTTAAGGCTTAA

TGCGACAATGACTGCTGGCTCCTTCACTGATAGGTAGCATCGGTAAGCATCGGTCGTATAGAATACGG
GTTAGGGAGAGCACGTGGGTAAAGCCTTAGCTACAGGAAGCACCCTAGTCCTAGTGTATCCGAGCCAG
TCTACTCTCCTGGTTAAGCCACTGAAGAGCTAGCAGTAGTAGTTTCTTCAATTGACCCAGCAGTTTAT
ATTGACCGAGTGAAAGCATAAGATCCAATTCTAGTCAAAGTTCCAGTGCCATTCATTGGATATCCAGG
TCCAGCAGCAAGGGCGGTAGAAGCAAAGATACGAGAAACATCCTCGCCTGTATAATCGGCATCCGGAC
CAGCACCTAGCACAGATCTATATCCAAATCCTAGCCTAGTGACAAAGTCTAATCCATTGAGTCGGTTG
ATCCCGCATAGGCACGGGCAAGGGTATCAAAGCCAACGAATCATGTTCCCAATCCAATCTCTGCTAAT
TCGTTTCAATTCCAATTGAAGCATTACATATAGATTGCATTGACCATCAACAGGAGAGGCAGCAGGAG
GAGAGGCAAGCATAGCTAAGGTGAAAACAGAAGTTCCAAAGCCAGATACAGATCAAGATCGACCCTCG
CCGGGTTAGAGAGCCGAAGTAGGGAGTTTCACTAGCAACAGAAGGAAGGGCAGGAAACCTAGGTTGAA
TCAGAACCGCTACCAGCTGCAGAGGCATTTCCAGGTGCTTATTGAGTTCCAAAGCCTATTCATGTTCT
GGGTGAAGATCCAAACCCAGATCCCGTGCTAGTTGGAGAGCTACCACCTAACCCAGCATCTCTTTCAT
TTCTAGAACCAGCATCAAAGCAAGTACAAGATCAATATAAAAGTAGCGATCGATGCACTCGATGACAG
GTGGGCAGCCAGCCCTGCTCCTCCCTCCCTGTTGTTCCTTGGTCGTGTGTTGTTCCATAGCAGCGGAT
CCACTCAAATCTGATTGCTTGCATAATGACATAGCTCCCCTCTCCCTAACAGAGACGGCCATTCAGTC
CATTCCTTAAATAGTTGTTCGTCAGGGTTCCACGCTCACTTACTAGTGCTTTCCTCCTGCTTTCCTCT
GCTTTCAAAACGTCTGCGCATTTGATTAGGGCACCCAATATTCTATGCCGAGAGAGATACAGAAGTGC
TTGTTTATAGCCGCTAGTACTTGCCGGGAGTACTCGCTTGAAGGAAAGTCAGTGGCACACATAGTAAG
TTCTCATTGCCGGTTCTCTTTGCTGATAGCCAATAGTTGATACAGGAGTCCCGGCACATAAACTGGGT
CGATGCCGGGAGTATTTGCCACTAGTTGAGGTCAGGATTCGATGCCGGTACTCGATCCAGGTGTAAAT
GCACTCAATGCCAGGTGCCAATACCAGGTTTCCATGCACTCGATCCAAGAGTAGATCCCAGGTTTCAG
TGACAGTCATAGCCCGTAGGCAACAGTCTCATAGACAGTCAGTGATAGAAATAACTCCGGTCAGTCAG
TCTAGAATGAAGCTCTACTCAGCGAACCTATACTCAGTGCATTAAGCTCTAGTCTGTACCTAATAGTA
CGTGTGTGCCCACTAGTCATTCAGGAAGCGATGCCAGGACTCGATGCTCCTACTCGATCAGTCGATGG
ATGCCAAGAGTCGATGCTAATAGTCGATCCAGGAGTCAGTATGAGCTAGAAGGTAAGGTGCAGCATGA
AGCTATGGTTAGTGCAGCTATAAACCTCTGGTCAGTGCCAAGTACTCATTTCCAATCGATTGATCATA
GCCAATCTGAGATACCCAAAGGTGCTGATAGTCGGTAGTCGTTTAATGAAGTCTTAATTGCCGGGATT
CATTGCTGATAGTTGATAGTCGCTAGTAATTGATGAGTTGGGATACTCAGCTACTCAGTTGGGCTTGG
TTACTCAGAATTGGATACTCGGGCTGGGCTACTCTCTCTAGACTTGGACATTATACTCAGCTACTCTA
CACTGGGGTAGGATACTCTACTGGATGGAAGGAGCTCCCGGGATGGCTGCTGGGGGACATTGATTCTG
TGAACGGATTCTGGGACATCGCCTCTATGCTCTAATTGCAAGTGAGTTTCCCATGTTATCACCGGGAG
GGAGGGACGCACGGGACGGTACGGGGCCCAATGAGTGAGTTAGGCACAGACATAGGGCCATTCCCTAT
AGCCAATACCCAGTAGCCAATAGATTGATAGCCGGTAACAATTGACGGTAGCAGATAGACAGTAGACA
ATACCCGCCCGGTAAAAATTATCACCTTATCTGGGTCGCGGTTGATTAGGTTATCCCTAATGAAGGTT
ATCCCACATAGTATGGATCCTTGCCTGGATTAGGAAGTGGCTGGATCGCTTCGCTTGCCTGGACCTAT
CCTTAATAGTAGAACTGAGCTCTATATACTTGAGGAATTTCTCTTTGAGTGGAATAAGGTTAGGTGGG
GCTGATCGATATCGCTTGTTGTGGAAGGTCAGGTAGTCAGGGAAGGTGCGTCTACTGAACCACTTTTA
TTAATTCCTGCTTTCTCTCTCTTATCTCTATATCAAACTTATGTGATGCTGAGGGCGTTTCTTCTATC
TTCTCTCTGGTCCAAACTGTTGAACCAATATTAGTATTATTATTCCCAGCCTCAGTTCCCGGATCACC
ATTTGAACCAGTCTTATTCCCAGCGTCAACAGTTGAACCAACATTTACATAATTAGTCGAAGGGGCTC
CCAAGAGCAGTTTAACCAACGGCATTCCCATCAGCAGTTGAAAGAGCATTCTTCCCAGGATAAGTTCC
ATCCGCATTTGCATCATCAGTCAATGGAATTATTTGAATAAGCTGTTTCAGGAGCATTATTCCCAGCT
TAATAAGCACCAGTCGAATTAGCATCAATTGCAGGTCTAAGAGGTTCGATTTATCTGTCCCACCACGT
CCCCGTTAGGGCACTGTAGGCAGTCGTTCGTCCTTTGGACGCGCCACCCACCCACCCTACATCTGTAG
GTCGTAAATCGAAAAATAACTCTCTGTCTAAATCGTTTTAAGGCCGCAAGATCTCGATCAGCATGAAG
TGCCGCCAGGGGGCGAGGGAGAGGTCTATGGTTCCGTAGAGTTAGAGGGTTGAGGGTGAGTTCGAGGG
CTAGAGTTCGAGGGCTATGTTTCCTACATCGAGGTTCTATTTCGAGGAATAGATCTAGGGTCTAGGTG
GGGGTCTAGGGAGATTCCGTAGCGTTTATCTGTGTTTGTCCAGCCGATGCAAGCAATGACTGTGTCCA
TCCCGGCTATGTGTTTCCAGCCCAGTTTATTAGAGCCCAGCCCGGTAGCGGAGCCCAGTGTATTAGCC
CCAGTGGATTGGATCAGCGGGGTAGCCTAGCGTATTGGATCAGTCAATTAGCCAAGCGGAGTAGCCAA
GTGTATTAGCGGAGCCCGGTGTATTATAGCCAGGGGATTGGATCAGTGGATTAGGCCCAGCAGATTGT
ATCGGCAAATGGAATCAGCCGGGTAGCGATCCGAGTCGATTAGCGTAGCGGATTAGAGCCAGTGGGAT
TAGAGTCTAGGGGATTAGAGGAACCAAACCGAGTATCCCAGCAAATCCCAGCTATGACTGACATCTCC
ACCAGGCATCAATCTCTTGGGATCAAGTACCAGCATAGAGTACCGGCATCGAGAGTGATCGCCACCTG
TATAAAGTACCGGCTATTTAAACCTTCTGTAAATGACTACCTCCTTACTGCTAATGAGACTGGCTATG
AGTGGAAATGACCTTAAAGCAATGACAAGAGAGTTGCACTGACTGAGTAGAGCTTTCCAAGAGTAGAG
CTTCTTTCTGTTCTGGCACTGAGAAGAGCTTCATGCACCAACTAGAGCTTCTAGGCCTCCATTGATAG

GCATACTTTATCGGGTATGTTTGAACGGGGTGAGGTACGAACCCGCTCCCCCGACCAAAGAAGGGGGA
GGGGTCTTTGTTTCTATTTAGGGTTTGTGTTTATGGTTACTCTCACTCGGATCTGTTGAACAGGTTCT
GTTGTTTGAACGGCGGGTCTCCAATGATGGGTACCTTCTATTGTGGTCTGGCGAAAAGGACTTCCCTG
TCGTCTGGCGGCGGCCTTCCGTCTGTTGGAAACTAGGGGGTCAATTTCTATTCTGGTGGTCACATGGG
GGCGATGATAAAGCAAGCGAACGTAGGCAAGTTATTAATTTCCAGGGATGTCAAGATCGCTAAAGCGA
GCTAACTTCGGCAAGCCAAAAGTTCGATCAATAAGCAGGCCAGGTGTAAGGGTAAGTGGGCAAGGTCG
ATATAAGTCTGTAATCAATCTATCAGGCAGCTGCCCTTCTACCCGCAATCAATTTAGCAAGCAGCTTT
AGCTCAACCTAAAAGCAAATGATGCCACCATCCCGGCTATCATCGGCAATGACACCTGGCAATGACTC
CTATCTATGATCAACTATGATCAGCAATGATCGATCGGCTCCTTCTGACTATGATCGACTGTCTATGA
CTGACTACTGGCTATGACTATAGCTTCAATGATTAGAGCTTCTAGCAATGACCTTGTAGCAATGACTG
GAAATGACTGTCTCCTGCTATGACGACTGGCAATGATAAGCTCTCGGCACTGACCTTCAGGCAATGAC
AACTAACTGGCAATGAGCGTTTTACTATGACAACTGCTCTGACTGGCAATGATAAGCTCTTGGCTTTG
ACCACTGAAAATAACCTGGCTCTGACCACTGGAATGACCTTCTGCCAATGATTGGCAATGACTGGCTG
GCTATGAAAACGGGGCTATCAACTGGACTATCGGCGGCAATGCCCTTTTGGCTAGTACTCCTTACTAT
TATCGTCAATGTCGGGTGCTGATTGTGTGAGTCTGCTATGTGAGGTGCTCTATGTGCTGTTTCTGTAT
AGGTGGCCCGGTCGTACTTCAAGGGCGGGTGCCTGCGGACTGACTATGGCTTACGCGGACTGATACTG
TGCGTACAGTTAGCTCTAGGCTCGAGCAGGCAGCAATAGAAAGGGCAGGTAGGTGAGAGTGTGTCCAA
TTGTGTTTTTTTCCACGTCCCTCCCTAGGTCTAGTTATATCGTAAGTAAGGGATATACCTACCTTCGG
CAAGAAGCAAGTAGGATCAGCAGGCAGGTGCGTAAAGTTGTCAAGTTCGAAAGAAGCCCGTCCTCAAT
CTAGCAAACAGCGAGGTTCCCGCGGAGCCTAAAAGCAATCGACGTGATCCTGCCTTTCATGGGTAAAC
TTTATGAAATGCCAATATTCAGTAAGAATAAAATCTACTTTATGTGCCCCGATGAGATTTCAAGAAAG
GCGTTTTTATTAGCAAAGAATGTTTAATGAGGCTGTTTTCTCTAACCGGTATCAAAGCCAAGTATTCG
AGCCAAGCCTAATAAGGCAGCAGCCACAGTTGAGTAGCCCAACCGAGCAGTCCAGCCCAGCAGGTATA
AGCATTAAAATGTTGTTCCGCCATAGCGTTAAGTGAGTTCGAGTTCTCTGTAGGGTATATAGGTCTAC
GGTTCCGTAGAGTTTAGGTCTAGGTATACTTCTATGATTGGCTGCTCTAGGCTCATACTACGGTTCTA
TGCCCCTCATCATCCTTCCACCATGTAGAGCGGCGAGAGTGAATATATGAGCGTAGCAGGCAGAGCAA
TGAATCTATAGAGCAATAAAAAAACAGTAGTGTCAGTGTGCGCATAGCGATAGTGTGTCAGGAAAGAG
CGTAGCAGGCAGAGCAATGAAAAAGAAAGAAAGATCTCCATATGCCCCCATACAACTTAATGAGCTGT
ATATAGCTCTTATTGGTGGGATATGATGAGCTATAAAAGTGATGATATAGCCTATTAATCTTATCTTT
TCTTACATAGATATAGCTATCTTTTCTGATTGACGGGGGCCCCTACCCTATCCCCTTTTGCAAGCTAT
TGACCCTGCCTTCCTCCTTCATCGAACGAATGATCCCCTAAATGCCCACCTCCTTTATTAGCGACCCC
CTGTCAATGACAACCCACCTCCTTATCGTTGACCACCGGCTCTGGGCTGACCTTCTGGCATAAGCTAT
GTGACTTTTGTATTCACTTCTTGTGAATTAATTAATGTCTGTAACACATCTTATTACTTACATTGTTC
CGAACCGAGTAGTAGGCCCCGGCCAAGTATCTGCTGAGTATCCCAGTTGAGTAACTAAGCCCATCGGA
GTAACCAAGCCAAGCAGCTGAGTAGATCCCCATTTGAATAGCCCATTGTTGTATTATAACATATATAA
TGAGCGCTGACAAAGATGAACCATCTTACAAGAAAGGTGTTACCCCGATTACCCTGATTCCCCAGTCC
CGCATTCTATTATTATTGCATTCCCGCTCTCCCCATAAGGCTCAGTACGCGTAAGCAAGGAGGATACC
ACCCGGGCAGGACACATATTGTCGAAGAAGGAAGGACCAGTAAATGAATTGAATTTGATACCTGCAGC
ACCCCGGCGGGACTAATATGCCGGGGTAGGGAGGTCTTCGAAATGAGTGGACTTCCCTTCCGTTCACC
CGAGCCCGGCCTAGCAAGCCTTCGCAAGCGTTATAAGGTAAGGCCCGAGTGGGCCCGGAAAGCACATG
ATTTTAGAGCTTTCAACACCCACTATCACAATCCTGCTCGAGCTACTTCTCTCTCCTGACCACTGGCA
CAATCACCTTACCTGGGAGCATGGGATCCACTGGTTAGGTAGGCGGGGCATGGTATCTACCCACTGAA
ACCATCCATCCTCACTCTCACTTACCCGGGGGGAACAGGAGGCACGAAGTTCTTTTATTGCTCCAGGC
CATAGCGTCGCCTATCAATTACTTCTTTGGGCCGCAGGAGGCTTTTTCTGGCAAGAGTCCTATTAGAA
GAAATCCCCCTTCATGGAGACGAAGTAGGGGGGCTCTCTACCCCACCCGTATACCACATTGCATACCA
AAGTGTAGTAATTCTCTACTCTCACACACAACACATAGTGGGAAAAGTATGGACACCTAGCAACATGG
GATGGGGGAGGACCTTTACCGCCCTTTAAACCTGAAAGGCAAGGCAAGCAAGATCTGTACCTGTAGGG
GACAGTAGATGAAGACCTTTACTTTTTAATACTACCCACCTGCCATAAATTATTCTATTGAAAGCCCT
AGCTACATGCTTCCCAGGGAATGAATGCTTGAAGACCTTAGGAGCACCAGATATCGTTGGATCGGTGG
ATGGGATTGGGATGGGAATCAACATGGCCATGGGAATCCGTTCATTAGCCCTTACCTTGCTCAGACAA
TACACCAGAATTACCTTTCTATTAGCCCTAGACCATGCTGAAATGGGAATGCTATCAGACCTGTATCC
GGATCAGATCGATGCGATTATGCAACTGGGATGAGGTTCATTAAGCCCTTACCTTGGAAATACAACCC
ACCTGCCAGACAGACTTTCTGCTGCCCTAGATTAGGAAGTCTTACCAGTTACCAGTTAGACGAGTATC
AGACGAGGGGAGGTACCACTAGCAAACTACCCTACAAGCAGCGACCTAGGACGCCAAATTGATTGCCA
TGGGTTCAGTCATTAGCCCTAGACCTTTGAGAATATGCTACCTTATCCTTAGACACCTTCATCACCCC
AGTCAGTCAGTTATAACTACCCTAGAACTCACAGGCTTAACCTTTCTCTTACCCACTACTTAGTTGCT
TACAGGACCCATACCCTTCTCTTTCTTTGTTATGACAATACACCTCCTATACAGAAGGAACACTTTAT

CTGCCCTAGAACTTCAGCTTAGCTACAGCCTTATAATGCGAGAAGCGGCTACAGTATAGTGCGTGGTG
CGTGAGGAGCACCCTCAGTATATAGACGCGCACCAGATTTGTTGGGTTCATCAGCCCTAAAACCTTGA
GAATTGTACGGTCGCCCAGAGCCAGTCGCCAGGAGGTCTTTTTTGATAGCCGCTAGTCGATCCAGCAG
TCCATGCCAGGTGTCGATGCAGTCAATGCCTGGTGTCAGTGCCAGTCAGGGAGGGAGGTGGCTGATGC
TCGCCCGGAGAGATCTTGATTAATTGCCTACAGATTCGGCCCAGGCACCAAGTTGGCTTGGAACAGAT
TTCACAAAACCTTCTCATTTGACATTCTTCTTATGCTCCGCACCTCGGTTTTAGCAGGTCCAAGCTTG
AGTGATCGGGGGTACTAAATTGGATTTTTTTTATTACCGAAGATTGGCATTTCTCGGTAGTAGATACA
ACAGTAGATCCAACAGTAGATGCACTCGATGCACTCGATGCCAGGAGTCGATCCCGCTAATCGATCCC
GGTACTCAATGCTGGTAGTCGATCCAGGAGTGTCGATCCAAGAGGCGATCACTCGATGACAGGTGTTG
ATTCACTCGATCCCAAGAGTTCAAGATGTCGATGTCAGGAGTCAATTTATTCTTGTCTGGTTGCGAAC
CGATGGAGACGATCTTACGATTCACCTATCCTTGCTGGTTAGCTATCGTTTGCTTGCTGACTAGACGT
TTGGATAGACGAGATAACCCGAAGAAGAAGCTGGTCCAACAAACGCATAATGATAGAACCTGTGGTGG
AAAAACGTTAGGCTGGGACAATAACCCGGCTGGCGGGAATCGAAAGCTAGCTGGTGGGCTTACTAACT
GGGATGGTTTTTTTGCTTGCTGTTCAATTTGAAAGCCTAAAACCCGTCCTTGCACCCTATGAAGAAGG
GCAAAACCTATAAGAACTATTCAGCAGAAGGTGGCCTATCACTTAGTCAATTATATTCCTGCCTGGAA
ATTGGCTAGTGAAAAGGATTACGTATTAAGGGCAAGACCTGTCAAACCTTCCCTTAAGCGGAATTTAA
GAGTTAAAAAGTGGCCTATCAGTTGGAAATTCTACGCCTTGTCAACACATGGTAACTTCACTTCTTTT
AACTTAGCCTATGGGGGTGGTAAATTCCTCTCTGTTAACCTGGCCTATCACTCATTTGATTCCGGGGC
TTGCTCTGTTTCGTCATATGAGGAGGGTAAGGCAGCAAATATAGGCGATCCTCGCTTCTTTGAACGGT
TGTAGCCTATACTGGTACCGGTACTGGGGGAATGCAGATACTTCCTCCTTTTTTAAGCCAACAAAACC
CCGGAATACCGAAGAAAGCGAAGGACAACGCAGCGGGGGGAAAGATCATCCCAGAAGCAAACCCTGGA
ACATTGTGAGAGGAAGGACTCCCTTAAGCCCAGCAGCATTGGAAGATCCAGAGCCTTAAGCCCAGGAA
GGAGGGCAGGAAGGCGCCAGCCTTAAATGCATTAATTTCAGCGGCTTACTAGATCAATTTTGAGTTTG
GGACCCACATGGATCATATTACTTGGAACCCGGATAAACACAACTTAGATTTACCCAATACGCGGCGA
AGGTTCTAATTGACCGGAACCACCCTAACAGAAACAAATAGCTTTTCGGGTCATAGTTTCTGGCTGGC
TCTCCCGCCCCAAGCCAATCAGGTTATCCCTGAATTATAAATATTTTTTTGGGGGGAGGCACATAGAA
TAAGATTTTCAGTATTTAGCAGACTTTAACGCTCCCCTATTATGGGTTTATTCCGTGCACCCGGTCCC
GGGGTTCGTTTCATAACTTATTATTTATCCCAGCCCGGATTCAATCAGCTATTACCCAACTACTCGAT
CTATCTAACTATTAATCGCTCCCCCTGCTATAGCGATAGGCTCTTTTGGACGTCCTGTCCTTCTATGG
GTCGGCGGCTGGCCCTCTCTTCATTCGCTCTGGGCTTTCATTCATTCATGACCATTGCATGCGGCTAT
CCCTATCATTGATTAATATAGATAGAGGGAGGTTTGCAACCTCGGCCTCGGCTTCATTCTCTCTTTCT
CCATCCTTACTCAGCTGGCTTCCTAAGCTTGCTGCCCGCTCTTCAATCCAAAGAGCACAGAAGGTTTT
TGCATGTGTTACGCATATGGATAGATAGCCCTAGCCAGCATATAGCTAGCGGTGTTGAGCCGCGTTCG
TGAAAGAGATTAAAGACGCTTTTAATATGACTGACTTAAACGAACTATAGAGGAGCGACTTAGTACTG
GCTCCCGATACACGCTATTCAAGCTTCCATGACAATGACTCGGTGACCTTGGAACCTTTTATATAGTT
CCCGCCCACCCCTTCAATTAGGCAGCGGACTAGACGGAAAATAGAATCAAAGTATTTAATTAGAGCCC
TCTGGAATAGGAAGAGCCTTTCATTATTTATAAGGTCCGAACCCGCAAGATCTGTAGAAGCCAACCGA
TGTTAATTCGTCTATTCAAGATGGCAAACTATACTTCTGTTCCAGGTCGTGGCCTCCTATCCCCCACC
ATTTCAAAGGGCTTTTTTAGGGGTTTCGGGCTTTCTTCTTTCCGAACGATATTCTCAAATTCTTAAAC
CTTAAAATAGAGAGGGAGGGCTCTGGCTCCACGAAGAATTCTGCTCCGTTCCTTCCCCTCTTAAAATA
GAGAGCGCCCGGTCCCAGTTATTCCCCCCCCCATGGTCGCATACCAAGGGAGAACGGGTATGGGGTGG
AGGGATTCGGGCTTCTTCCTAACCCTATGGGTGGTAGATAATAATTCTTTTTTAGGCTCCGTCCATTT
CAGATGCCCCATACTCCTTAAAAAGAGAGGGGGCGCTCCTCACCCTATTATGTCGGGTGAGAGCTTTT
CGCTCCTCTCCCCGAAGAAAGATAGATGGGTCCGGTTTTATATAGGGGTGACTATGGCCAGATCTGAA
TGCAGATCGAGCGAATTCGATGGATATATAGGAATAGGAGAGATTCCGGCTACCGAACTCCAGGTTAT
TTCGCTACCAAACCGGAGGGGGGGAAGTGGGCTTAGTAGGGAACTATCTAGTAGATACCCATTTATCT
AAGCTTTAAGCTCTAAGCTCTAAGGCGGGCGGCGCAGAACGACTTACAGAGATACCTTTTATTTAAGT
AGTTGCCTTTTGTCCAAAGTCCCCTGCTCAGTAGGAGTAGCTCGGTGCCCTTCGGAGAACACCTCATG
GATCTGGCTCTCCTCATGAAAGAACAAATCTATCTATCCAGTCCCTTACCGAAAAGGAAGGATCTCTC
ACCTAATAGGAAGGATAAGCTCATATATAATAGCGTGTGAGGGACGACCCATGCCTGTCCAAAGGGGA
TACGTTATAGGGAAGACCCTGCATTGCTGCAATCTAGCTGGAGCAGTCCTATTGCATTGACTAGGGGA
CATGTAGTGGTGGAAACCCGGGTAATGGGGCAAATCGGTAATGGGCTACGGGAGCAGAAGTTATGGCA
AACCGGGATTGGCTACGGGACCTGTAGTGGTGGAAACCCGGGTAATGGGGCAAACCGGTAATAAGGCA
AACCGGGATATAGAACTGACCAGACCAGAACTGACAAGACAAGACCTGAACTTACCAGGCTTCTGGTC
AGGCACACGATGACAGACATCACTTTTATCAGTTATTATACCATGTAATTGACTCGTATATAATGGAT
AACGACTCCTTGGCTAACGACCCCAATTACGGAAATAGAAGAGCCATTACCTGAATACCTTACCTTAC
CTGAAGACCTTACCTGACTTTACGGCGCTCACTAGCACCGACAAGCACCCGCTAGACCGACAAGCACC

CGAAAGCACGACAAGCACCCACTAGCGCCCACTAGCACCCACGAGCACCGACCGACTAGCACTGACCC
GCTAGCACTGACCCGCTAGCACCGACGAGCTCTCCTTCCAATAAAGAAATCCCATTAGTCACTGGTGT
AGAGGGAAGACCCAGATTGATGCTAGGTGGATCGTTGATTTGTAGAGCAGTTATGAGGGGAGACCTTT
ACTTAATGGGGAATATGAAATCTGGCTTCCTTTCCTTAATACGGACTACGGAATGTGGGTCGGTGGAT
TGGGAATACCCTGATTCCTGCTATGTGGGTTATCACTTTCTATAGCATTTCTCTAGGGTTGACTCGTA
TATAATGAATGGATCTAGTCCCACCCTCCTTGAGTAGTGAGTTTGTGTAGGTTATCACTTCTTTATAG
CGGTGCGATGGGAGCAGTTCCAGTTCTATAGCATTTCTCTAGGGGAGTTTATGGAGGGAAGACCCTAT
GAAGAATGAATAAAAAGGGGAAGGCCCAATTTGTTTGATAGGATTGGAGCGGTATTTTCGGCCAATCG
ATCAGATCCTTCTATATACGATACGTTATTTCCCATCGCAGGAGAGGGCTAAATAGAATCAGCAATTC
TATATGGATCAGCTAGAGGGGGGAAGGAATCGAATCTCGGCCAGCAACGGTAGCAACCTTCATCCGAG
CACACATACTGAACCTGGAGAATGGGCATCCTACATAGCCTCGTTGTCCGCATGGTACTTCCGTGCCA
TTGCATTGCGGGTTGGTCGTGTCATTGCTGCTGCAAGTGCTTATAGGCTCAATCAAGATTACATACAG
ATTGCTTGATCCTCGTCTTTTTATGAGGAGTTAGCTGCGCATTGCATTGTGGCATGGTCGATTGTTCC
GTTCGCCCCGTTCGAAGGCCAACTCAACTATATATCAATCTATATAGTCCTTCGTTAATTCAAACGTA
GGGTCATGCGCTGCGCCAATTGAATTGATAAGTAGAGCGCTGGGCCGCCTTGCGTTGTAGCATGGTCT
TTCTCCCCATCGCAGTCGCTCATTACTCCATTTGAGGCCTGATCGATATAGGGGCGGCATGCGCTGGC
TTTTCATGCATGGTCGGTTTGTTCCTTTGCACGCACGGTGGTGGCTTGATCGATTGATTGATCCGATC
CTTAGTAGCACCATAAGTACACCACTGGAATAGAATCCCTGTCCTCCATGACTGGAATAGAATCCATG
TCCTCCATGTTAGTTATGAGTGGATCTGCTCTAACCTTTGGCGAGAGGAAGGAGGAGGAAAGAGGATA
CTCCCTCAGTCAGTCAGTAAGGGTAATAATCCAATGGAAATAAGCCAATGGGACTTATACTTCCAATC
CAATGCAATCCGATGGGGGAAGATAAAAAGCGTAATTACATATATAATAATATATTTATTAAATTGGC
TTTGAGGGGCATCAATATGGGCGTTGGTCGAGCATCTCAAGATCCTCAAATAGCTCTGCGTAAGGGAG
AACCTTACCTTACTTAGAAGGAAGGGGAAGA
>Repeat_18
ATACAAGCTTCTGCTTAACCTGGACCTGGAACTGCTCGTGCGGCATCATCTGAACCTGTAACTGCTCT
TGCTGCTTCTTCTGTTACCTATGCCTTTTCCTATGGTGGTGATTATTACTTAGGGTTTTAGGGTGCTT
CCACTGCTGCTTTTGGTAGTGCTGCTTCCGCTTCTAGCTTTCGAGATGGTGGAAGGTACGCTCGCTCT
CGCTCTGCAGAATATACTTAAACAACATAGTCGTAAACCAACCGAATCAACCTCCGCCTCGGATGCCT
TTGCTTCTGGGTTACGGGTAACTGGTTCAAGGTAAACAACTTAACCTAGTACTTTTGCTTCTAATGCC
TCCGCCTTTATTTCTGCGACCTTTGCTTCCATCCGTGGTGCTTCATCTGGATCATCAGGAACTGCATC
ACGAACTTCATAATATCGATCACGAACACGAACATCAGACTCTGGCTATCGATCTGGATTATCTGAAT
CTGGATATGGATCCTAGCGAAGGGAAGCTGGTTTTGAAACTGGATCGACGGGCAACTGGAACTGGAAC
TACTCAAAGGGATGTGGGCTCTGTCCCTGCTTACGCCTTTGTCTACGGTACCTTTGCTATTAGTCGTT
ATGGTGCCTAAACCACTGCTTCTTATGCTTGGTAAGCTGCTTAAACTCTTCAGAATTACCGCAGCTAC
TGGTGCATCTATCTTAGCTATTGGTGCTCCTACCCTTGCTTCTGCGCATGATGTCTCTATATATTATG
CTACCTTCGCTCCTGCTGCCCTAGATCTTTGCTGCATCTGTCTTTCCTAGAACAGCGTAAAGTAAAGC
TACCTTTGTATCCGCTGGATCTCCTGCTGCTTACTCAGCCACTGGATCGCCTCTGTTACCTTCTCCAT
TGGTGGTGCCTATGCCTTTGTAGGCTATGCGTATGGATATGTTCGAGGACCCAGATGTAGCGTTGGTC
GGTCAGCTGAATCATAAGAATCAACAGACTCTACATCGACAGCATAATCATCAAAGCCAGGTAGTGAA
AGGAATGATGAAATTCCATAGCCTGGTGCGCGGAAAGAGAGATGCCTTGGCCTTTTGCCAAGTACACT
AAGTCCCGCAAATGCCAGCCTTGAACCTGTTGAAAGAAGCCTTTCACCTTCTGCGCTTTGCTTATGAC
CACGCTTGTTGACCATGCATGTGGAAGCCTCGACCAGACCCCTATCCCTTGTCTGAGGTAACCGAAAG
CGAGCTGAGCTGCCGGCATATATCTCTTGGCCCGGCTACTCGTTTTTGACTCCCACCCACAACTACTT
GGTGATGCCCTTGCTTTTACTGGGCCTGGAACTGGAAATGGATATGCTGGGCGAGATGCTGCGGGTGC
TTCTGTGACTGGGTCTTGCTTTCTATAGGATATGGATGGATCTCGCTCACGAGGGCTTGGATCACGCT
GTAGATCATCGGTAGGAACTAGGTCTCGATCACGATCTGGATCTTCATCTGGAGCGGAACTGGACCAA
CTCAACTGCGGAATCGAAAGAGTCCAACTGGCTATGCCCTCTCCTTTGTGTGGGTGCTTCCCTTGCTT
TTGCTTATGGTACTACCTTAGCTACCGGTGGTGGTACCTCGGGTTATGCCTTTGTATCTGCAGCCATC
CGCACCTATGCTTCCGCAACTGATGGTAGATCCACTGACTCTGCTGTCCCTCTAGATGCCTTTGCCCT
AGCATCTTTCACCGTAGCCTCTGCCCCAACCTTTGCCTCTGCTTACTCTGTAACCTACGCTACTTACT
ATGCAGCTGCGGGTGAATCTGCTCGCTATGAATCTATAACTGGAGGGTGTCAAACGGAAACATAAACA
ACTGGATCAATTAGCGTGGGATCCGAATCTTTATTATCTGTGCCCGGAGCTCTTTCTCTGCTGGTTTT
GGGACTGGCTCTCGAACGGTAAGGAATGCTCGCTCTGTTATTGGTAATGCTTATTACTCTGGATCTTG
ATATGGAATCGGAAAAATGGACAGTGGGGAAGCTAGCACCGGGTAAACTCCAACCGGATCCGCTATGG
AACCTGCTGGATATAGATCTGGATCTATTGGTGTTGGCTCTTAGTCAACTGGTTCCAGGTCAACAACT
GCTGAGGGGTCACCTGGATGAGAAACTGCCTTATTAGCTGCCTTTGTATCCCCTGCCTTTGCCTTAAA
TACGTAATATTCAAAGATCTTAGGTAAGGTTTGAATTTGTTCCCCGGATCGATTTCCAGATGGTCGCT

AGGTAGGGGCAAAAAGTGCGAGGTGGGGCGCGGCGAAAGATATCGGTTAAGGTATACTTGGTGAACGT
AGGGAGCTACCCGTGTGGATCCGCTCGAACTCTAACTGGGTCTGCCGGAACTACTACCGTTACCACCT
AGAATAGGTGCTGATCAATTGTGCTTCTCTTATGGATATGTGCCTCCGCCTCTGATGCTGCTTGGTTA
TGTCGGGTCGGTCGGTGTTAGTGAGCTGCGCTTACCCTATGTGTGGTTCGATCGGTTATTGGAAATCA
ATTTGTTCGTGTCGTTTCTTCGGCGAGCAGGTGCCTGCGATCGATCGGTGTTGCTTCTATTCGTGCTT
GGTGCTTTCCGGCCGATGTGTTCTTCCCTTTCCTTGTCGATGCTTTGTATTTCCCCCCGATGCTCTGT
CCCTCCCTCCCCTTCCTTTCTCGCTGCGGGCCTTCGATCTCGATCTGCAGGGTCTGGATCTCGGTCTA
AAAATGTATCTGCGTTGATAGCTGTGGGCTGATGGTGGTGCCCCTTCCTTCCCTGCGGCCTTCACCTT
TGTGGGTGGTGCTTTCACCGCTCTACTGAAACTAGTGGGGGTGCTACCTTTCCTCCTGGTGCCTTCGC
TTATGGCTTTGACTTTGCAACCGAAGAAGCCGCCTATACGGAATCAACTTCACCAGGTCCTAATACCA
TCTATGCCATTATTTACGCCTTTGCTCCTAGAGATGATGCAGAGACAAGAGAGGTAGAGCCATCACCA
CCTTGCCCCAGAATACCTTCCGGTTCCATAGGCATTTAACCAAGTGAAAGCGCCAGTTGATCTTGATC
TATAAGCATAAACGACAGCAGTCATTGACCCAGATACAGATCCATAGCGCGCGGCATCCCTTCCAGTT
ACATATCCATTTCGAAAGCCAGAGCCCGTAGTTCCACCAGCAGATGACATGGATCCAGCGCCATATGA
CCGAGGAGCGAATTCACCAGTGGCAGAGCGAAAGGCATAGCTATCAAAGAGCAAAGGGGCCAGTTAGT
ATTCATAGGCCAGTATTTTACATATAAACATCTATAAAGCAGTACGGGAAACACTATAGATAGATAGA
AAGCCGCCGCTTTTGAATCCTTCCAACTATTCACACCTATATTCGAAGACACCTGGTAGGGAAGGAGT
AGGGAAGGGAGCAGAGACAGGAAAAGCAAAGGTAGTGCGGGTGGTAAAGCTTATTACATACCTACAAT
AGTTTATCACCCGTCCAAGGAGCATCACTAGCACGCATAGGAGGAAAAATACATCCCACGTAGACACC
TTATTTCGGTCGAAAACGTACTGATTGCCCATTTCACAGGGAGAAGAACGCATGTTACCCAGTGGATG
GATGCGAGTAAAAGGTACGTTTCATGGCCCAATATACCCTGATCCATAGGAGGTAATCTCGCCGGGTC
ATTTCTTTGAGTCGCTAAGTGGTTCAAGCTAAGTGGGGAAAGAGAGCCAGACGGAGAATCTCTTGCTT
GTTAAGCCGGGTGGAAAATAGATTATTTCATTGGTTATCCTCTTTGTGCTCTTTCTTTCTAATTATTC
CCACCCACAACCCTTGCTTTAAGACACTATGGTGAAACTGGTAAGATACCATGACACTATGGTTTAAT
ACGGCACGGAGGACTATTGGTGTTTTTATGCGCTTAAAGACTGAACTACGTACAGGGTGCTTTGATTG
ACTGGAATAGAGACGACGAAAGGACACTTTGACTCATTGGTTATCTAATAAACCTCTCTTTGAGCCCG
TTTCATAAACACCCTACGCATAAGTAGAGCCAAGGACAAAGGCCCGGGTGGATCTGCTGTTGGGACTG
GCGTTGGTGCGCGCTTCAATGGATTTCCTTCACAACTTGAATTGATTTCTTTCATTGGATCCCCTAGA
AGCTATAGCAATTCAGTGGACTAGGGAGAGGAACAGACCCGGTTAGCTTCTTTCGTTTACTTTTCCTG
ATGCCCCATGGGCAGAGGTCAAGGTAAAGGTAGCTAGAACAGTTCCATACCCAGAAGCAGGTGCATAA
GCAAGGGCAGTAGCATATCCAGTTTTTGACTCAGTAGCACTAGCAGCAGATCCAGACGCATATCCAGA
TCCTCTAGCAAAGCCAGGTGTTCCATACCCAACATCTATACTACTAGTAGATTCTGATGTTGCTTATG
AGTTTGATCCAGAAATCCAGTAGCATCAGTACGAGAGTCAGTAGTTCCAGTTCCTGATTCTTATGTTC
CAGATCGAGATACAGTAGCAGAGGAAGCAGATGGTGAGTCAGTAGAGCGAGATGGAGATTCTGTCTTT
CTAGATCGATACCCTTTTGAACTTGTTTCAAAATAGGGGGCCAGCAAGAGCAGTTCCATACGCGGCAG
AGCTTCCAATAGTGGACTTAACTTCCCCACTAGTCGCGGCTAGATCGAAACCCAGACCCTTTCAAAAT
AGGGGGCCAGCTTCAGTCGCAGGAGAAGCGGGGGTACCTAGAGCAGTAGATCCAGTCGAAGAACCAGC
AGGTGTAGCAGCTCAATTAATAAGGGGGGCTGGAGATGCATAGAAAGATCCAGGACCAGCATAACCTA
TTGATTCAGGTGGTTCAATAGCAGATTCTGTCGAGGTAGTTGATTCAGTCGTTATAGGCGCGAAAGCG
GGAGAGAATTTCCTCCTCACGAAGCCGGTTTAGATCAGGCCTTTATATACGTCATTTCGGGCAGGCTT
TATATCTACCTTTAGCCTATATGAGGCAAGTGCGAAAGCGTAATTACGTATATAATGAGCTATTGATT
CGCATCTATATTATATATGTGATTATGCGAGCAATCAGAGGCCTTAAGGGCCCCGGGATTTTGAGAGA
CAGAAAGGGTATTTATTGAATGAGACTGAAAGGCTTATAGAGGAGTATAAGGCTTATAGTTCATAAAC
GCTATAGGTCTTCATCACTTCCCCCCCTGTCCCTACTTATTGGGTGGGGGGCACTTATTGGGTGGGGG
GCAACAGAACTCCTGTTAGAAAGCCTACTAGAAGAGCTTCAGAGCACCAGTTTCCTTAAACACTCGCT
ACCAGCCTAAGAATAATTTCCGGGATTGCTTGGTATTTCACGAATAGAATCATCTTTGGAGCCACAGC
TTATTGGGCCCACGTGGGAAGGTCTTGGCAAGGAAAGCGGAAATAAATGGACTTAAAGCCATATAAGC
TAAGCCACCAGACAGACAAAGCTCTCTTCAATTAATAAGGGGGGCTGGCAAACGGAGAGGCGCTGCCC
TATATAAGGCACACCCTTCCCTTATAGTACATTAACCAGACCCAAAGCCAGTAGCAGAGCCTGTTGCA
TTATAAGCAAGGAAAGGTACAGATACATTCATCCAATTCACTGAATCGCACAGAGAGGTCAACCGGGG
TGAGAAAATGAGAAGGTTATCCCCAAAGCTTCGCCCTCAAAGCTTGCAATTCTCCGACTCAACTCCGA
CTCAACTCCGACTCAACATGGTAGACCCGATCTTTCAAAATCTTCTCCCAAACGGAGGCTCGTATCCT
CGCTCCTATCATTCAGCATTTTCTTTTAGTGAAATCTCCTTGTTAGGGCTCGCAGGCTCATCGGGCAC
ACTAAAAAGGATTTCTTTTTACTAAATTTCGCCTTTTCAGCTACTTTTCCTATAAAAAGGGGCAGGAT
TGATTCGATTGGAGGTCGAGTTAGCCCCTTTAGGATTGATCCCTGATAGGGCCTTTTTACGAACTCAA
CCTACGAAGCTGCAGCGATCACACGGGCGAATGCTTTATACCTTTACAGCTAGAGTTAGTTATTGCGG
GCGGGGCAGGGGTACGCAGGGGCGGCAAAGAAGAAGACATGGGCCCAGCAAGGAAAAGGTCAAGAGCA

GGGGATATTCACAGGGGCAACAATACGGAGGAAGCCACACTGAGAGCTACACTTTGAAAGTAACACAT
AGATTTATTTGCATATTCGAATATATAATACAGGAGCTAAAAAGGCCCCCTTTGACCCCTTGACGAGG
AATGATCAATACCATCACCTTACCCCTTGAGCCCCTGATCGCTACTTTCCCCCATGATTAGGACCGAT
AACGCTCGATAGAGGTCTTTGATAGAGGCCTGGGAATAGAAAGAGGTCTTGACACACGTTATGCCTCA
CATTTTCACTTTTGCCCGGGAAAGACCTAACGCTTAACCCTGTTAATCAGGACTGACGCTTCGAACCT
TGCACCTTGATTTCGAACCTCCCCTTTTAGCTCTTACATTACCCGATTACCCCGCTTCAACAGGATGG
ACTCACGCTTCGAACCTTGAACCCTTCAGCTCCAGATAAAACCTGCTACTTGCCCGGGAGGGAAGGGA
CTACGGAACCAACGGCTCCACTAACAGATAATAGCTGCTGCTACTTGCCCGAGAGGGAAGGGAGGGAG
TAAGGATACAACGGATCAACCAATGGAACAGACAGCTTATAGATTTAAAAACATAAAGGGAGGGACGT
GGGGAACAAAGAAAGTAAATGCATATCTCATATATAATGTGCGAGTTACCACCTATATCTTTATCCGA
ATTCCAGGTAATACGCTTTTTAATAAGCTGGGAGCAGGACACGAAGTGGCTTGAAACAGATTTAACAA
AACAGTATATATTAACATTATTATTAAGAGAAATCTTCTTTTAAGCTGGTCACAGGGAGCCCTCATCG
CTGGTACTAAAATGAATTGATTCTTACTCAATTTTGGCATTTAATGAACTTTCTCTATGTTAGTCGGG
ACGGATTAAACAGAGTCATTGGCTGAGCGGGGATACTCAGCTGGGATACTAGGCAGGGACGCTTTGCT
ACTCCGCTTGCCTTTGATACTTGGCACTTGCCTGGGCTCCCGATGGGATTGGTCGCGCCTATGGGCTG
ACCTTGGATACTTGGTTGGGATACTTCAATTGGATACCGGTTAAATAAAATGATCTCATTTAACATTA
TTGGCCAATAACATTCTATTTTTAGGAGGCCAAGGAGCGTAGCCCTCATCAGAACAATGAAATGGATT
TTATCTTTACCAAACTATACCCTTCCAGAGAAATCCCTATGATTGCCAAGTAGTGCGATGACAGGATG
CCGGTACTTGATGCCAAGAGTCGATGCCTGCAATGCCAGTATAACCAGTTGTCAGTATGAGCTAAAAG
GAGGTGCAGCAAGAACCTATACTCCGTGCTTGAAGCTCCAGTACGTGACCTGTTCTCGATCCCCCGTA
CCCAAAGAAAAGGAGGTGTGTGGGATGGCCATGCGTGAGGATGGCCCATTCGGTGCTCGGGTTTGTCG
AGCGAAGCGTTCTGCCAAGGGAGAAGGAGGAAGGGCAGTGAGGGCAGATTCAGAGAGCATAGAGGGTG
AGGCTATGAGTAAACTCTTGCTGAACCGACTGCAAATAGTGCTCTATTAGGCACTTGCCTAGGGAAAC
TCCGAAAGGTTCATTAGGGAAACTCCGAAGGGTTTATTAGGGCTTCTCCGAAAAGATTTGAATGAAGT
CTGACTGGAACGCCAATTGAACCCTTCTCTAGGCGCTTGCCTAGGGAAACTCCGAAAAGTTGCTTAGG
GGAACTCCGAACATGAAATAGAGTACAAATCAGACGCTTCGTTGGCAGTGAGTGAATCTTCCATGTCA
TATAATCATTTTTTCTGGTACTCTGCTAGGTAGGGGTAGGAGGATCAGGCACTTACTTTCCTTAGGGC
AACGCCACAGGGCAACGCCCACTTTGCTTTCTCAAATTCCCACTTATCAGTTGAGAGTTCGCTGCCTC
ACCTTATCGGTATGGTGGTAATTTGGAGAGTGAAAAGGGGCTCTTCATCTTTTTTCCTCCCCTTCCTC
GTGACACAAAAGGGATAACCTTTTTTTTATTCCTGAGGTAAGGGGATCCGGTTCTGAAAGGGAAAGTG
GGCCTCCACACTAAGGCAATAAATTCCTTCCTAAAGTGGTCTAGAACTAACAAGTCTAGGTGGGTAAC
TTATAAGGAGACATGAACCTGAAAGACAGGTCTCTGCAGTGGATGCTAATTCCATAATATAGGCTCCT
CCTCGGCGGTAACACACGATACCGAGAAGGTAATAACCAATAAGAAGGGTAAAGTATTAGGGTAAAGG
ATAAATAGAAGCCCCGTACATCCTAAGATTCCTAAAGGCAAGTGTTATCGAAGCATATCCTGGTTGTC
TGGCTGTCTGGGCATCATATTCTAGGTAAGAAGTCCATCCCCATCTAACGAGGTTGCTCTGATGGACC
ATAATCCAAGTCATCCGGCAGTTGAACAAAGCTGAAGTTCTGTACGATATCTGCCTAAAGGGAAACCA
ACCCTTTCAGCTAATAGACAATAATCCTGCAGGAGCATGCTACTAAAAGTTCTACCATTATCCGATCG
GTGGTTATAAGAGAAGTGCATAGGTTGTAAAGCACTCAATCTATTCTCTTATAGGGTGGGCAAGTATT
GAGTAAAGGTCTGTTGGAACAGATAATGCACTTAAGTAAAGAAAAGAAAGGTAAAGTTGTTTAAATCC
TTCCCATCCAAGTATCTAGCGGTACCTTCTAAGCATTCATGAAGGAAGTGCTCTAGGGCTGCTAACAA
GTCTAGGTCATAAGGTGGATGGGTACACGAAGGATGTGAGGTCCTAAAGGGTAAAGGTCTCTGAGGTC
TATTCAAATGAAAATTCCTAAGGCAGTAGTTTCTAATTCCTAATGAGTGTCCGGCCGGTACAAGATTC
ATTGATAGCTATTAGATGTTGATGGATTCATTTGTAGCAGTACCCAAGGCAGATGAGTCAGGAGGTTA
GGTCTCCTCGAGGTCATTGTCTAAGCGCAGGTCCGGTCATTGGTAACACATCATGCCATAATGTTTTT
CGGGTTAATTGGTGACCCACCTCACTCGCACACTCGGGTTCTGGCTACGAACTTACACAATGGTGAAC
AGAAAGCGTATGTAAAGGGGAAGGCAAGGGGTAGGGCAAGGTTAGGAAGGCAAGGTTGGTCGTAGGGT
AAGTGTAGGAAGGCTCGTTCAGAGGTCAAGGGTAGGCAGGATCGTTCATAAGGCAGAGTTTAAGTTCC
CAAGGTGTAGGTATGATTTTGAGGTTCCCAAGGAAGGCCCAAAGGCAGAGTGTAAGATCTCAAGGAAT
AAAGAAGGTCCCAAGGAGGAGGTTAAGTTCCCCCGGCTGTAGGGATGGAGTAGTTTGATGAGGCATAG
TTTCCAAAGGCTAATTCCAGCCAAGGTGGAGGTATGAATCTGAGGATCCCAAGGAAGTAAGGATGCTC
CTAAGGAGTTTAAGGACCAAGGGAGTGTATCTATCTGTCCAGGTCCGGTCAGTGGTAACAGTAATGGG
AGGTTTGTAAGTTGAATCACTGCACCTCTTTCTACAGGCAAACGATACCCTCATTGGGCTCAGGGAGA
AAACCACGTGATATATTTCGATCAGCCCAAGTAATTTCCTTGTTAAGCAAGCCAACTGAAGCCTCCCT
CCGTAACGCAGGGTTTGTGGATCGAACTAAGCCAGGTGTAGAGCAAGGAAAAGGCAGTGAGCAGCGGG
GCAGGCGGTAGATGGTCTGTGTTGCTTCTACTAACTCGTCGTTGCCACCGCCTCCGCTCTCGTTCTGG
TTGTTTCCACCTTCCCTCCCATTCCGATTGTCGCCACCCCTGTTGCTCCAGCGAACAGTGAGTGAATG
ATTGATGTCATAGCGTGGATTTATCCATACTTGCATCGCGGATTACTTCGTTCGGGTAAGAGTAGGAG

CAAAGATGGAAAGGAATGGAAGTGCTCTGCCATTGTGTAAATAAGTCACTAGATGGAGAGGGAGAAGG
AGAGAAATGAAGTTTCCTTCCTGTGCAGAGAGGGGGATATATCTGGGGCAGGTTGGAGAGATAAAGTA
AAAATGCGGTCTTCCTTAAGCTAGTTCAGCTAGTTATCTCCCCCTTCATAAAAGCTTTAATAATTACG
AAAATGCATTAGTGCCTATTAATGGTGGCTTCCGCCCAGTACTTTATTATTATCTTTCCCAGAAGGGG
GTTAGCCCTTCCATTGTCCTCGCATAGAGAAGGTTCGGTGACTAATGTGTCACTAGAGGTGGAAGGGG
TGCCCCCAAGTCGTGATGATTGGGTCTGGGTTAGGGAGGTAAATATTGTGGGAAGATGGGTTACCCAC
TTTAGGGGTAACAGCTACAGTCGATGCTTGATGGGTATCGACCACCCCTTGCGTTGGGAAGGGGTTCT
GGAACACTGCTGGCTGAGAAGCTATAGGGGGGTTCTGCGGATTTCCCCCCTGCCCTGCAAGAGGACTA
GTACTAGTACTGGCTCCTGCAGCAGCCATCCAATGATGGATCTCGGGTTCACAAGGACAGTGTTCGCT
ACGATGACCAGGGAAGCCGCAAATTGCACAAGGAAAGGTTGTACTTTCAAAGTTTACCTGTTCTTGTG
CAGGTGGATTAGTGGAATCCTAGTTCTGGTTTGTGGCATTCTGGTTGCCCACAGCATTAGCAGCCTGA
TTAGGTGGGTTGCCCCCAGCGTTCTGGTTCTGGTTTTGAATCTTATTCTTCCCCTCGTTCTTTTTCTT
ATTGTTCTACGTATTTGGCGCAGTTTGTTGCGCAAAGACTTGGCCTTGCGGGGGAGGATTAGGTTTAA
CCGTATTGGGTTGGGCCGGGGGTGCTTGAGCTTGAGGTGCCAGAGCTACTGGCGGAGGACCAGGAAAT
GGATAATAAGGGTTTTGGTGTGGCCCACCATACTACGAACTCGCACCTCCACATGAGGAACTGGTACA
CGAAGGACCATGGTGATGAGGGTGTACACTAGATCCTGAAGTACTAGCTCCTAAATGAGAAGACGAGC
AATTTGGGTCATAGGGGGCACTAGGATACCCTTGAGGCATAGATGGCTGCCTGTAAGGATTTTGGTAA
TAGGACGGATTGGGTGGGGGAAATAAAGGCACCCCTTATAAATGTTTCATTTATAAATCAGTGAAAGG
GGCACATTGCAAGATCCAAATTAAGTTACAGTTTACCGAGTGAAAGCATCAAAGCCAGAAGTGAAAGC
GGGGTAAGCGCAGGTGGCTAGAGCATCATAGTAGTAGATAGCACCCGGAGCAAAGTAATAGGCACTAG
CAAAGGCATCACCCGCGGAGTAAGAGGCCGGTTTAGAGCCGGAACCGAAGCGAGCAGACCCAACGCTA
GAACCTGCCCCAGCACCGGCAGGCCTTTCGTTCGTGAGACATAAATAAACCCAAAAGTATCGGTTGCA
GAGCCCATTCCCGTCCCAGGCGGGGATCCAGCCCTATTCATATATTTTGTAGTTGGGTATTAACCAGT
TATATATAGATCAAGATCCAGCAACATTAGCAAAGGCAGCACCATAAATAGCTTCACCAGCAGCAAAG
GCGAAGACAAGGGCAGCATAGGAAGCAAAGGAAGCAAAGGCACGATCTATTGCAGCATAGCCTGTTGA
TCCACCAGTTTATCATGTTTACCTTGATCCGATTGAAGCTATTGATCTTAGGGGTTAGTGCGCTTGTA
GGTTTGTGCACTAAATATATTGTTCCAGGGGGGAAAAGAGCCTATAGATCCAGTAATTAGATATCGAG
ATTTCGTGCCTGTCCGAGAAGCGATTTACCCGGTCCCAGAAACAGAGGTGGAGGCAGGACCAGTAGCT
TAGTCAGTTGGCCATTCTTTAGGTGTATTTCCTTCTCGGGCACAGTTGATCGTATGTGGCTACGCACT
CCGGTCCACCCATTAAGACACGGTGAACTGGCTCTGAAGCTCAATATCGATCTGGAATAGAATGAAGA
TCAACTACTAGTTCAAGGCCAACTTGATAAAATACCTTTCCTACGGCTGCCTTTGTCTCTAACCTCAC
CCCAGGTTACTTTGCTCCTGCTCCTGTATCTCTCCCGCCTGTGCTCATATCAATTTAAGAGGGTTCCT
TTCCTATTGGTGGACTATATTAAATGGTATAAACACTAGCGAAAAATGCTAGAGCAAAGCTTCTAGGA
TCAATAGCTTTTCTTGGGACACCGCCCATAGGGACTGGAATTCGCATAGCAACAGGGACTGCGGCTGG
ATCAGCGAGAGGTAGAGTAAGGTTCTCTATCCAAGCAACCATCTAGGGACACGCACAGGAATCGTCCT
TATTTCCCTGGTCAAGTAAGTTAGCGCCTTACTTGTTTGGACTCAGTATCTCTCTCACCCTTGGACTT
GGCCCATGGGTAAAGGTAGAAAGCAGTCACAGTGCTGCTTCAATCCTAGCTGCTTTGCTTCGATAGTG
AGCTCTCAAAGAGAATAGAACTCTATCTAAACATTGGAGAAGGGTCGACTAAGCAGGGCGGGGGGGAC
TAAAACACTGGCTGGAGGTGCTTCCTGCATTGCTAGCGGCGGGTGTACGAACTTTTCTCGATAGAAGC
AGGTGTCGATGCCAGTCATAGCTGGGCTTGGCTGGGATACTCATTGCCTAGAAGCTCCGGTCAGTGCA
TGAAGCTCTTCTCAGTGCCAGAACAGAAAGAAGCTCTAGTCATGGAAGCTCTAGTCGGTGCATGAACG
AAGCTCTGGTCGGTCCAGCTAGCCCTCATTATATACGTAATTACACCTTAAGATCGAATCATTGTAAG
TCGGTAATAGCCGGCCTGGCCTGGACTCATGGGATGGGCTTGGCTGGGAAGTCGGTAGCTACATATTC
TATGCTTGGTTAGCCCAATTTTATCAGCGCTTAGCTCCCGGGATCGATGCTTTGGAACCTTGACTGCT
GGCTCCTTCAATGACTGTATATGACTAGCTGCTGCTATGGCACTACTCAGCTCTCTTCAATTAATAAG
GGGGGCTGGCAATGCACCTTATGGGCTAGTCCTCCTGGCCTGCTGTCTCTGCCACCACCAGTAGTTAG
GCCTCCTGTCTCTGACTGGTGGCTGTGGACAGTGGTAGTCCGTGGGAGCACCCTCTCATGTGGGCTTC
CGCCCTATTCAATCAAGGGGAGCTGCCCTTTTCAAGTGGGCATTAGTAAAACCCCCTCTCTGAAGGAG
CACGCTGATTGATTGCCAACATCATTTAGGGAGGTGTCATAGTCCAGTAATTGCCGGTCATTACCAGC
CGGTCCATTGACCCTACTTCCTCATGGGGTCATAGCCAATAGCCATTCCCGGTCACTGCCAGTCAGCC
AATAGGTATCGAGGTGTCAATGTCAGGTAGCGATGCTGTCGATTCCGTCTTTTCCGGTAATCCAAGAG
TCCATGCCAGGTAGCGATCGATGCACTCGATGACAGGTGGGGGCCTGCCCTGCTCCTCCCTCCCTGTT
GTTCCTTGGTCGTGTGTTGTTCCATAGCAGCGGATCCACTCAAATCTGAAACAGAGACCAATAAGCAA
GGTAGGACCAGACCGATCTCTCGAAGCCTTCCTCTGCAGCGTATTATCACGTATATAAATAAAAGATA
CAATATTAGGAATACCTAATCAAGCTTGAAAGACGTTTTGATTGACTGAGACAAGCACTTAATCTCCT
CTCATACGCTAGTGGATGGAGCCCAGACCAAATTGAGTAGTGATCCTGGTATTGTCAGGTTGTAGAGA
GTCTCCCGTACCCGTATTGTAGAAAGAGAGTCAGTGAAGAGATACAAGGCAGTCAGTTAAGAGATACA

AGGGTTCGTTTCCCAGTTCCTATTGATCCATAACCAGCATCCCTACCTCCACCAGTAATCCAGTTGAT
TCTTCAGTTCTGCTGTTGATGGAGTTGTATATGAAGCTTATCTTGTCGATCTATCAAGTTGTTGCACG
TGGGTGACTGGCTATAATGGAGCTTGGTAAGGAGCACTACCTACCATCTATACCTTTACTTACGTGCA
CTGTTTTCTACTCAATTTGGTCTGGCCGCTTCAACCTACTTAAGAGATTTAGTGCTGTCAGCCCTTAA
TAATAGAACAAGAACTTACCCTACTTACTTATAACCAATTGCTGAAGCCAGGAAGGAGGAATGTGGGT
ACAGCACACCTTGACTATTGACTTTGACTATGTTGCCTATCTTTCCTTTACCGTTCGTTCAACCCACC
TGCTTCACTAACCAGAATACCTATACCTCTTAGCTCTATACACTTGCTTCATTAATCCTTCTAAGCTA
GGCACTTAGACCATTCATTTGTATTCCCTTTATAAGCCAATTCAGTGACCACTTAAGGCCTTACCCTG
AAGACTCATACCTAATAAAATAAGGGGCTTACTGGTATGCCCCCCCTTTATATTTTGGGGACTTGCTT
CGATGGAAGAGAGCCAGTTTACCCATAAGCAATGGCTGGACGATTCAAATGCGGTTATCCTTACGTGT
GACTTTGAAGCGGTATCGCAAAATAAGGGATGAAGAACTTGGTTGGTTGGCTATTAGATCCGTGTGAC
TTTGAAGCGGTATCGCATACTGATAAAACTGGCAGCCCACCCACTTATTGATCCGCGGGAAGAAAAGT
AAAGTGAGCAAGCCTCCCCTTTCTATTTTGGGGACTAGCTTCGGGAGAATCTCTTATGGGGATTCGAT
CCAGCCGCAGGTTTCCCTACGACTACTCAATTTGGTCTGGGTTACGAACGACTGAACCACTCAATTTG
GTCTGGCGTTCACGACATCAACTGAACCATGAGTACTCCCTAGCGGGAGGAGGAAAGTTGACACCGCC
AACTCGCATACCACGCTTCGCCCGCCCTTATCGATCGATCGAACAAATAGAAAAAAAGAAGAAATAGA
AAGCGTAACGTCTTGCAAGCCGGGGAAGGTTCATATGAATGGCAGGCATCCCTGCCAGAGAGTATTGC
CAGCCGA
>Repeat_19
TAAGTATACTTTTAAGTCAAGAATATTTGATAAATCACTAAGTATGACAATTGAAGAATTCGTGTGAA
GAGTTCGTGCCCCACTAGTTCTTGGGGGAGTCCCCGATATAGAGTCTTGGGTGAGCCCCAGCACAGGA
AGGAAACTTACTTCTTTCGTAACTGCCCGGTTGGTTTCTCCTTCTGGCTTGAGGAGAGAAACTATTGG
AGTAGCCTTATACACTTGCTTCATGAATCCTTTGTTGCTAGGCACTTTGACCCTTGATGACTATTGAC
TTTGTTATGCCCCCAACTTCGTTCGTTGAACGGACGGTCCTATAGAGGGAGGAGCTGTTGGATGGGAT
GGATGTACGGGCTTCCAGAAAGCATTCACAGGCCTGGAATCACACTTGCTAAAAGGACGGACATATAC
GGACAGATTCGGTGGTACCACTAGGACTATTCTGGTCCGGCCGAGCCTTGCCCAGTAAGGATACACTT
AAGGAAGGATACACTTTAAGAATTCTTATGGGGTAACATACTTCTCTTGCTTCTGGGTCTCAATCTAT
CTGGAGATGGATTTGTTTCTTTCACTGAAACTCCTGCCTTTGTTCTTGGTGTGGATACCTATGCTATT
GCCTCTATGACTGAAACTACTTCTGGTGGTGCTGGGCGGTAAACTGCCTGTGCTTCTGGCTTTGCTCG
GTCAACTTAAACCATTGCTGCTTGCTTTAGTGCTGCTTCTGGAACGGCTGCAAACCTAAATATCATTC
CATGGGTCGCTGGGGGGCTAGTGATGACGAGTGCGCTGCAGCATCAGGTTCCACTGGGTGCGAATGAT
CACTGGTTTATTTACCGTGGGCCCCTTTAAATGGTAACTGAGATTGGGTAACTGGGGCTTTTAGGTGA
ACTGCTTGCAGCTGCTATTAACTGGTGGGGCTTTCGGTGCCCCTAGGTCTTGGCTTTATCTTGTAGGT
CGTTCTCGTTCGGTGAGAACATGGGGAACTCAAACTTTTCCCGCCGCTTTCCGTCCGGGGGGGGTTAA
AGAATTCCGTAAACCCCGAGTTGCAAGTCGAAAAATCTATCCATCTATTTCCTTGCCTAACGTTCTAG
CCAACTCCCTCTAGCCACACACACATTTCTAGCCACATGGTTTTAAAACTAGCCTCGTGTTCAACCAG
CCTCGCTCGAATTAGTTTGTCAAGCGTTTTTCCACCCACACGTTCTATCAAGTAAGAAAGCAAGCCCT
CAGTTCTTGGGCCATCCCGGTTTGATTTGACGCGCATCCGTGTCCGTGTTCTTCAAGCCCGCAATCTA
GCCATTCAGGAAGAACATGAGTAATCCCGCCCGCCTGCCGTGTTCGTTTTCTTGTCTCAGCCCATCCC
GTTCGTTTTTCGGACCAGCCGGGGTTTCTTCGACCACCAGTAAACAGCAGCTGCCGGGTTCGTTCGTC
TAGCATCAAAGCAAGCAAGCCCGGTTCTTTGTTTGTTCGACCAGCCAGGCAGGGTTTGTTTCTTCTGT
CCAGGCAGGGTAAGTCGGTTCAGTCTCGGCAGGTGGGGTTTTATCTTCATCGGTTGGCGGGTGTTCAT
CAATCAATCATCGGTGTGCGGCGTTGTTGACAGGTGCTCCGTGGCAGACAGGCAGAAGTTACACTATG
GGAGGAAGGATTCTCTTATATATCAGCATGAGATGAACTTGAGTGAGGGAGGGAGATCTCTATTATTA
TAAGGGAATAGAATCAATCTCTATGGTTCTACCTCTATATCTTAGTATTTTACATATGGAATGGGAAT
AATATCATATATAATAGGAGATTAAATTGATGAGTGAGGGGAAGAAAGAGAAGGTTATCCAAACCCTT
CGTGTAGCTCAGGTAGGTCAGGGACTGACTGGCCTGGGATTTTAAGGGTTGGAAAACGAAGTAGCCAC
GTAGGTGGGAGGGATCATGCCTATATTACTGAGCCTGGCATGTGAGTAAGTAAGGTGTGACGCTTAGG
TATGCCTGGGGTTGGTTGAGTTTCGTAGCTTGGTTGGAATACCGAGTGATAATTCGAAGGGAAAGGGT
ACAAGACCTGGCATTGGAATGACATTGATTGAACCCAAAGAATCACGAAAGAGATGAATGGAGAAACA
AACTCGTATATAATATATCTGGTTCGAAATTGTGTATATATACGTATATATATCTATGCTGTGTGGTG
TAGGCGGATCTAGCTCTACGTTGTAGCTCACTGAAGACTCATGCATTGTACCTCATCGATCGTGTAAC
CTCTACGTGTAGCCGCATTGACTAGTCCCCTCCCTGGATAAATCTCGTCTGGCGGGACCATGGCTAAA
TCGATATGCTCATCTGTAAACAAAATGGCTCAACGTCTTCTGGTGGGAACATGGAATGGGTGGAGTAG
TTAGGGGAATAGTGCTACCGCCTAACGCAGGGCTACATAACCTGCCTGAAGACACTACCTGCCTGCCT
TGCGTGAATGGCCTGACCTCCTCAAATGTCACTACCTGCCTGCCCTGCGTGAATGGCTCAACCTGACC
TCCCTGACTTGACACTACCTGAGACTAGCACAATGTGTCTGGCGCATGATCCTCGACAGTTGCATCGT

ATATAATGCGTGTTCATTCCCTCCATCTAGCCCACCCCGGTAACCACATTTCCAAGGCTCCTTATTGT
TAGAGGATCTATTTCGAGGTTCTATTTCGAGGGTAAGTTCGATGTCTAGGGTCGAGGGCTATGTCTGA
CTGTCTCTAGTTGATTAGCCCGGCCAAGTATCCAAGGCAAGCAATGACCGGCTTCCAATGATTGGCTA
CGTGAGGTGCCCTAAGTGCTGCTGATACGTCGTGGGGTTTCTATCTATAATGGGTGCTATCACCTCTG
AACAGGGCCGGGGCGCAGGACAAAAGGCTTTCATTTCTTTATGGGCGATGCTAGCTGGTCGAAAGAAA
CCAAAGCTGCTCTGCTCAAACCCGTTAATCTAACTGTAGTGGAGAAACGTTAAGCGGAAATAAATAAA
GGCTTTACGAACTAAATCGATGAGGCTGGTCGAGCTATCTCTTTATGCTTGCTGGCCGGCTGCTGGCT
TACATCCCAAACTTCGCATACCCCATTTACCCGTCACACAATACCAGAGAGAGGCTTGCCCCGGGGGC
CCCGACCCCCTTTACCTGGGAAATCCAGGATGGAAAACACTAGACACAGCTTGACGAAGATAGAAAGA
ACCCGACGAAGCTGGTCAGTGTTGCTAGGACGTTTTGCTAGGATGTGTGGCGAGACAAACGAAACGAG
GCTGGCTGGTGGGCTGGTCCTGCTGGTTGGGAGAAGGATAAATGGGCTAGCGGGGCATCCTTGCCACC
ATGGGCTGCTTGAAGAGGTGGGATCGATGCATTAACCCAATCGCGGAGATAGGAATGCGGGCTTAATG
CGTATTTCACTGCCCTTCCTCACCTTGTGACTAGGGACTATGACTAGGGACTCTCAGCGAAGACAACT
GGGTATCAACAAGCACTTATATTCAACGACTAACAACTATAAGCAGCACTTCCTAGAGCTTCTTCATT
AGAGATGGTAGCTAAAGATCGAGATAGAGATCCAATGAGTATCAGAGCATCATGAACTTCCAATCCGT
AGAGAAAGAACGCAGCCACCGATAAACGTCGAAATCGTCCTTCTTCCCTCATAGGTAGGGAAGGGGTT
CGAGCTCGTGTTTACAGGTGCGGCTGTATTCAGGCAAGTACTCGAACCTTTACTTAACCGGCAAGCAC
TCAAACCGGTCCTTTTTAGAACCATTCCTTGCCTTCCCTTACCCGAAGACCTTACCTTTCCTTGTTCG
CACAGCGAATGAAGGCCGGGTGGAAGGGCGCCCCTTATATAACATAATAAGTGTTCCTCCATAGACGC
GAACTATGCCAGCCAACCAGAGTGGAGCTAAGTAACTGTGAAACTATAGTGCTTCGGATCGTTCAAAG
GAAGCTTTGATTCGCCAATAGACACACTCATTCCGTTGATAGTTGCTTGCTCGTGAAAGGAATGTGCT
GAATGGTTGGAAAGTCGAAGCGGGAATTTGTCTATGTCTATAGGTTGGGAATGAATAGTAAGTTCCAC
CTCACTAATAGGAGATAGCCCACTAGTGCTAATTAGTTCTATCGCCCGCTAAGACATTAGCTTAGCTC
TCCCCAGTGCACATCCTCTTTGGTTCATTCAGTATAGTTAGCCCACCCGATCAATAAGTATCAGCCAC
GCCCCTCATTCATGTTTTTGATTGACCGGACCCAGCCTCTTCAAGTGGGTGGGCATAGTTGGGTAGGC
ACTTCTACTGATTGATCATCTTTCAACGTTCAATTGATTATAGTTCACGGAATGAACCATCAACCCCT
AAGGTTACTCCTAAAAGTAAGGCAAGAGAACCCCCATGGGATTTCTTTTATGAAAACCCGAGAGGTAA
GGTGCGTTTCTTTCCCGCTACTTCTTTTAGGGGCCTCATTTTAGGGGCCTCTCACCCGATAACTCACC
CTATATGCTGCTCTCTTACTTCTCTAAATGGGGATCTCCATAAGCACCCAACTGCCCTGCTCTTAGAA
ATAAACACAGCACGAGGAGCCCAAATAGAGAGAATGAAACCAAAGAGATAGATCCCCAAATTGAACGA
ATGAAACACTTAAGAACGAATAGATTATTGATTTGCCTATAACGTTACAGGACCTTGCCTTATGTTTA
GAATCATCACAGCACAGCGAAGCATAGTCCCCCCTACCGATGATAAGCATGTCAACCCGACACCAAAC
CCTTATGGCGATTACCCATGACCCATGCGGACAAGCGATTTCATTCCTTACCTTACCTCATATTTATT
CCTCCCCTCAAAGAGCCGCAGAGGCTTGGAACCTTAATCTTCAGGGCTCCTCGAGTCTACCATCCATT
AAAGGAAGGAAGTGGGAGTGATAGTTGTGGCTATTGATTAGGGATACAGTCATTATCGGGTTACTGCC
TTTGCTGAAGATAAGGCCACCCGAGAAGATCTCGATTATAGAGGAGCGTTATGAGCGGTACGGGCTCG
CGGTTACTTATTGAAAGGGGGTGAGGAGCGTAAGGAGAAATAGAGGAACCTTATGAGCGCCCAAAGAT
AGGAGCGTAAATCCCTCATCCCTCATTGGTCATCATCCCTACTAATTCCTACCCTATTAATTACTACT
TTTCGGCTCTATAGCGCGCTGTACGAGCAACGCAAAGCATGGTAAAGGGCCAATACCTCTGAAACATG
GCGATCTAAAAATTATGGTACTTGGACACAAGCTACACGGTCTCGGGTCCACTTTTGGAGCTAGGTGA
GCAGGCACAGAATCGTTCACAGAATCTTTATGTGGAGTGGGTAGTTGGAGTCGAGTGGGAAAAAGGAA
AGGGGGGAAGGAACAAGGAAACATGAAGATGGCATGATGATGGATGGCGGGTAACAATGGTGGGTGCC
TTTAGGAGTGTGGTAGAGGGAAATCGCTTTAGGCAACCAGGTTAACCTTAGATATTGGACCTGTGGGA
TTTAGGCTGGCGACCATCCATGTTCTGTTCACCCCCGCAGACCAGTGATAACCAAAAGGACCTGCCAT
ACCCCAACTAAAACTGAACCCCAGAAAGGATTCTTGCCTAAGGCGCCATGCCCCTGCCCCTACTCTTT
ACCATAGGTAATGCGTCTTACTCCCCTAACAACGAGTAAAGAGCATTAACTAGCTCTATCTCCCTAAA
AATAGAAGTCAATGTCCAAAGATGAAAAACGATTTCTCTCATTTTACTAATTAATCGCGCATTTCTTT
AATTCAAAACTATCCGAAATGAATGAATTAAGTAATTACTTAAGATAGAGTGTTTTAAAGTTATTACA
TACATAATATCCTAAGAGGTTCGCCTCACTGTAGTTTTTATAGGTCCTGACGATTCTAAACATAGTCC
GTTGGTCGAGGGCAAGGCTAAGGAACTCATTAAAGCTAGTCACTTCGCATTCATCAAAACGTAATTAA
CTCAAACAAGGCTTCGCTGCGCTCGGTAAGAACTCATTTCATTAAAGCTGCTAGTAACTTCTTCATGT
TAATCAACTCATGCTAGTCACTTCATAGCTATTTCACCCTTTTCCGCCTTGGAATGGCTAGTAGCATG
AAAAGAGATCCGAGTAAGGTGTCTAGTCAAAATTGATATGCTGCCCCGATCTTTCTTTGTTTGTCCCG
AACCCCGTACCCCTAAAATAGTACTAATCTCTTTTTTTCTATCCATGCTACTGAGGATAGAAAGGTTC
CAAAGGAAGATCGAGCTAGCTTAGCCATCGCATTTCCCTCTGTTTCCCCCGATCCCAGTAAGTCTAGG
TTTGTAGCTCACATAGTTTTTCCTCCTTGCTCGAAGAGACAGATCGAACTACTGGAATCCAAAAGCGG
GCATAATAGCAATCTTACTCGTATTTCCCCGGGTGTAAGGAAGAGTATGAGAAAGGTGTAAGAAGAAT

ACTCCTCAAATACAGTGCACTCGCAAAGGGAGAAATAAGCCTTTCCTTCCTCTCTATCTAACTAGTAC
TGTTGGGGAGCTCCAGTTGGAGCATGAAGTGGAACGAAAACAGAAAGACCGGGAGTAACGGATAGATA
GCCAGGTGGAGTAGCAGGATGAAACTACACTATATCAAATAGGTGGTACGAAATAGATTATCACGGGT
ACTGGGCAAATCCTTCTTTCTCGCTAAGGCAGCTTATCCAATCCTCTTTATAAGCCAATGTCCATAAA
AGAAGCCCGGGTCCACGGAGACGAATGAGAAACATGGTACACGAACTAGAGTCGATATCGGCCCCGGG
ATCTATCGATCTATTGACTGAGAATGGAAGGAACGGAACTCACTTGATCGGTTTTGGAACCTCCTTCT
CGTCCCGGGCGGAAGAGAACTAACTCAATCAGCATCGCAGGCTTTCAACCCGCACATTGAAGCTAGCT
CGAAACCGCCATGATAATTCAGCCGTTGCCGAAACCACCTCTTGCTCTCTACAACCGACAATGCTTCC
GGCTATCATATTTCCTCGGAAAAGCTCTCTACAACTTCCCCAGCCTTCATTCACCCTAATGTGCCAGG
TCGAGTACATGCTCTCTCCCAACAAGTGGAGTCTATTATTAGATACGTATTCACAACTCGTTTCAGGG
ATCGCATAGAAAGGTTGGTCGAGCACCAAACAGCTCTCTCCCGCATTCCAACTCTTCTCAATTCAAGG
TCGAGTCCTCAACCTATTTATAGATAAGGTTGGTTGAGCGCCCCATTCTTTCTATAGCGAGTCCTCAA
CATGTTGTCGAGGGAATCCTATTATTGGTGCTCAAGTGCATAAATGCTATAACTCTTGTTCGTTGTCG
GAAGTGAGGAGAGTATATTATAAACGTATTAACCACTCATCACTTATCCGAACGAAAGAGAGTGCACT
CGATCGATAGATAGGGCATTTATGCACTCGACCCACTCGGGAGAGGAAGTACAGTGAGCGGTATGATG
CGAACTGACGCGACTCCGAATGGAAGCTCCGGTACAATATTAGGCAGCAGTATTAAGATAGCGATATG
ATGTGAGTCTAGCCGGGTATAGCTCCTTGCATAGACTGATAGGCTGGCGTAGCACTTCCAATTGTACA
ATGGGCAGGATGCGCTTTCTCTAGATTGTACGGTAGGCATCGCGCTTTATAATGAGTGTCCACTTATT
ATGAAATTACCCATAAATTCTTGTTGATCAATGATGTCTCGGGCATACAGGCCAATAGCTACTAGAAG
TAGTGTTCCGACATTGCCATTAATTCCTAAATGATGCGCTGTTCCGTCATTATCATTAGGATCTGTTC
CAACTATAGGTGTTTCAACAAATGCATTTGCTCCAAGAAGTTAAGCTCTGGTATTTGCACGTGTTCCT
AAATATGCAGAAGCTAAAGCAGTAGAGCCTGCAACCCTTCGTTCATTTCCATGTTTCCATTTACTTCT
GCCTTCTTTAGTGTAGTGAGCTAGTAGGTAGGTACTTTTGACTTGCCAAAAAGCAGCGTACTTTTTAC
CGACCGTGTACTTAATTGTCATTGTTCACAATTCAAC
>Repeat_20
TGAGGCCTTTCGGTCGGCGAAGCTAAGTCAATATTTCGGGCCACCAAACCCTGCAACCGGAAAAGCGA
ACGTACGTTGTCACACTCCCTGCCTTCCTTAGGTGCCTAGATAGAGGACAGGCCAGACGACGGACGCA
GCAGAGCGACCGGGAGCGGATTACCCACCTAGGGGGGACAAGAGACGGCCATCTCGAAGCACATTGCG
ACCTACAGGCAGCACCGGCGAGACCCAACACAAGGGGGAAGGCAACCCGACGATTGGGAGTCAGAGGA
TCCATAGCACCTGCCTACCTCGATTTGGAAAGGACCTCGTCTTTCTTAATCATTTTGGCACCTACTTA
CTAAGGGTAAGGGATCTATTCTCTGCAACCGTGCAGATTTAACAACCTAACGATACATCCAATACCAA
TTAGTTAGTGAATACGGTTCCAGCACAGATTGGATGCGTTGCTAAATCTCTGGATCTCCAAAACCAAG
ATCAGACCAAGCAAAAATAAAGGGGAAGGAAAGGAGGTCAGGTTATCGCAGAGCTATTTGAGGGGAAT
AAACATAGTATCCTGAAAGGTCTTACCAGGCGGGTTGGGATAACTGTAACAAATTATGAACACAAATT
CGGCAGAAAGGTTGGTTACCCTAGATTGGAGTCTGCTGATCAATCAGACAAAGGTCCCCAGGCCAATC
AATAAATAAATGAATGAATGGATAAGATAAAGTTAAGATAGGTGGGTATAGCTAGGGCTAGGTTAGCA
TTTAAATCAATCTAGTTAGGTACATGGATCAATCTAGATGGGTAGGCAGGACTGGGCATCACACGAAG
TAAGATAGATAGGAAGGGAGGGGGGATGCATTCAGGCGGGCATGGAAGAGGCACACAAGGTAAGGCAG
ATAGGAAGGAGCAGGTTCAATCCACAACTGCCAACTACACTGGTGGTTCAGATATACTACTTGTTTCC
ATATAACTCCCCCGGTAGTTTTTATTTAGTTAACTCCCCGTATCAAGCCTCGCATCCAACCACGCTTA
CATCCATGTATCCACGCCACGCATAGAAATATAGCAAGCAAGCTCAATAAGCTAATAAGCCAGCAATA
AATCATAATGGGTTGTTTGACCAGCCGGTATTCAACCCCAGCCCGCTCGCTTTATATCCTCTTCCCAG
CTAGGAAACCTAACAACCAGAAGACCTACCTCATTGCAGCCATATCCTAATACTAAATTTTAATTATT
CTATTTGGGAGACACTTACTTGCCATAAACAACAAATATAATAAATCTATTTCACGTATGATATATCA
GCATGGATAAGTTTAAAAAGTCTCTATAAAAGTATCTACTGGTGTTACTAAATGATATCTACCATATA
TATACTATACATGTTACTCTATGATGTCTCCATGGAAAAATAGTAATTGCCATGAATAAATAGAAGAC
TAAATAGGCTGGTCTTCCCGCTATCATTCGTTATTTTTTTAGGAATAAGATGAATAAGATTTCATTTT
TATGGGGTCCGGGGAGCATTACTCATCAGGGATCGAAAATGGATATTATTTTGAATAAACTATTGGAA
ATAATGATGAGGCTGGAAATGATATTAGAACTGCTACGGGAACTGAGGAATCCGGGAATACTGGGCTG
CTCCTTCAATTGCTGATTCAACTGCTGATCGAACTGCTCTTGGAACTGTTGCTCCTGGGGCTGGGAAT
AATGCTTCTAGAAACTGGAACTGATGACCCTGGAAATGCCGGTGAAACTGGTGTTTATGGGAATGAAG
CTGGTTAAACTGTTGGTTCAACTTATGCTGATGGGCCTTCCACTGCCCGTTCAACTGATGATGGGAAT
TATCTTGGAACTGCTGCTTTTCCTGCTGCTACTGGGAGGAATGCTCTTGGTACTGCCGGGAATTACGC
CGGAACTGCTGGTTGTTCTTTTGAAAACCTCCGCATTTAAATGTGGCTTTAGCAAATTATTTATGGGG
GGAGGGCGAGGAGGCTATATATGGAAAATAATACAATAACTCCTACCTATTACGCAAAACGATCAACG
CAAGCTAACTTAGCTTAGCCCTACGAATATGGGCAGGTGCAGAATGAAAAATTATGTCACTATCGTTG
CCTACACAAATTCTGGGCGAGCCATACGAGTGGTTAGTCGAAAGGCGGTTCTGCGTGCGGGCTGCTGG

GCTGGGCTGGAAACAGTAATAAACAATAGACAATAGCCAGTCATATTGTTCCCCATCATAGCCGCTAG
TCGATAAAGGTGTTGATACGCTAAATGTTTGTACTCAATGCTTGTAGTCAATGCCTGGTACTCCCGGT
TATATGTAAGGAGTAAATGTATTATTGTAGGTTTTGGAAACGATGGAGAGGAATTCGAGAAAATGCTA
TTAGCCATCAACTTCCCTAATTATAATCCACATTTTCACACTTGGTTGAGAAGAGATTATGCCTCCAC
CCGGGCCAACCAAGGGTTTATAGCTCTATAATCGCATTTAAGAATGTTTATCGTCGTTCCTACATACA
TACTTAGATAGAAGATGTACCCCCCATATGAGAGATATAATGGAGAGACATGTGTCGCTAGAGTTAGT
TTACTATGCAATTTTGGGCCAAGGGGCGAAGAATATTACTTTTTTGTTGTGAACAACTTTGTGCCCGG
AGGCTATGTGCATGCAGTGAAAAAGCTATGTGCTACCCCAGGGAGAGGCTAGGTGAGCGATACCATCA
CAATCATTGTTTCTTCCATACCCGAAAGAAATAAGTTCCCCATCATAGTTAGCCCCATCATTCTAGCT
GATAATTTACAGAAGGTCATTTTCAATAGCCGGTAGTCAAAGCCGCCTAAAGGTGCGGAGGTGGTCAT
TGAAAGGAGGTCGATAGCCGGGGTAGTAGGTGCATAACCCTATACCCACTCTATACCGCTACTGAACA
CACGTTCCTCCCTCAACGGTATGGAATGAATTATGAAGCGCGTTACATATGGTGATTCACTTGCATCT
AAAAGAGGTAGTAACATTTCATTGTTCCATAGTGTTAAGTTTGGGTATGGGGAACTTTCTTTTTCGGG
TATGATGGGGACTTATCTTGTCGTCTGGCGGTGCGGACTTATCTCTCATTTGTTGGAAACTTGGAGGT
ACTTCTTTATCTTATGGTGATAACACGGGGAACTCCAGAAGCTAGCTGTTCACTCGCTGCACTGCACG
ACATTCACTTACTGCACATAGAAGCCATTCACTGGTTAAATTCTCTTTGGGGTATTTATTTATGGATA
TTGTCTGTTGATAATATGATATTTATATCTTTTGTCTGGTGTGAACATGGGGGCCTTCTCTGTGATGG
GACTTATATTTCGCGTATGGTGCCAACTTGGTTCACATGTTTATATCACGTATGGTAATTACTTAATT
TTCCGTTTTGATCAACAAGGCCACGCCACCCCTTCTTACTCTTCAATTTATCATCCATCTCTTGCACG
TTCGCTTCGCACCTTAACTTAGTTTTACCTACTCTATCAACCTACTTACCTGTCTTTACCAACCTACA
CTCCGTGTCTCACTTAAAACTACTTAGGAGATGGAGTGCTGCCTGCCCAACCAACTCATTCAGCCAAG
AATACAGAGTGGGTAGACAGACACTATCGCTATTCACCTTTACCCTACTTTACCTTTTACTTTACTGT
TACCAACTTAAACCCACTTCCTTCACCAACCTCACTACTTACTTTTTAATGCCCCAAACAGACAGCAG
CGTTCACTGGCTTCCCTTATAGCTCTTGGATGGACCGCCTTCCAGACAGCATTCACAGGCATCACACT
TGGCTTACAGGAATTATTCCTTGGTACCACCTATTACTATTACTATTACTATTGTTCCGGGGCCCAGC
GTTGCCCAGGAGACAATTGAGTAATTATCACCCCATTGCTATGAATAGACCTATTTGCCTTTCGCGTT
TTAGTGTCTGCTACCAACCCTTCTTTTCTCTTTACCTTGAATAGTGCATTGGTGCGCACGGGCGTTCA
GATAGATACAGCATTCACCTGCGTGTGTTCCATAACACTTACTGAATTAATTCCTGGGTGGGTATGAA
TATTTTCCCGGCCGAAGACTAATTTTGAATTATCCACTATTGCCCTAGGAATAGAAGACTTCACAAAC
TTTAAAAACCTTATACCCGCTTTACCTTTATGTCAGATCGTTGAATCGTCGGATCGGAATTGCCCTAG
TTTTAATTCCTTACAGCCAGAACTGATTAGATCAGATTATGGGATGGGGTCGGCCGGGGTCATCACTT
ACAACCTATCCACCTGAAGGCCTATAACTTCTCACTTGCTTCATTAATTGCTTAGTCACTACTGACTG
CTATTTATCCTATTCTCAGGACGGGATCAGACTACCTGACCCTACTTACTTTTGCATCAGCTAGGAAT
GCTTCGTAGTGGAAGGGAGGCATGCATGCTAGTCATGAACCCTTTAACCTTTTACCTTTGATTAAGTG
TTTCCATAAAACCTCACCTTATGACCTAGTAGAACTGTTAGCCCTAGACCACTTTAGTAATTAATCTT
AGGAGGGGCAGCTATTAAAAACCCTTCTCTTTTCAGAAACGGATGGATTGCTTCGCACCTTACCCCTT
ACTTATGTGTTTGTTTCCTAACCTCCTACCTGCCCACCTTAAGAGCTAGACATGTTAGCACCACTTGC
ACCCCTATCACAGGTCACCCGTTGAACCAACACTGCCAACACCAGTATTCCCATCGAAATAGCAGCAC
CAGCATTAAAAGGATCATTCCCATAAACATAATTCCCCTGAGCAGTTGAAGGAACAGTTGAAAGAGCG
TAAAAAGCATAACCAGTTGTTGAACCGGCTTCCTCATTCCCAGTCTCAGTTCTAGCAGCTGTTGAAAA
GGCAGTTTAAGCAAATTCTTACCTGGTGTAACCGTGCAATGCTGTAAGAAAGACTCATATAGTAGGGT
GAAGCCCGGCCGAAAACGAGAAAGTAAATACGAATGCTCAACGTGCTGCCTAAGCAAGCCCGCCCGTG
CAATCAATCTTCTTCTCTAAGTTCCGAGATAATTTCCATCAGCACTAGTTTAACCAGGATTATTTACA
GCAGCAGTTGCAGCGGCATTACGATCAGCATTTCAAGCTTCATTAGAACCAGCAGTTCCAAGAACAGT
AGTACCAAGAATAGTAGAAGTGGCATAATTGGAACTGACATTATTTTAATCCCAGTATAATTATTATT
TCCATAATCATTTTTACACGGGGGAAATTGAAAACGGGCATTCCTGCATAATTTGAACTGGCATCATT
CCTGGCAGCAATATAATAAGCATTATTCTATCATACCAGCATTCCCATACTGGCAGTTCCACACGACC
ATCCCAGGAGCAGAGATAGTTGATGTCATCGTGGATGGCGGCGGTTATGCGTTGTGTTACCAGAGTCC
ACATGGCGGGCCGGTCCCCCACCTTGAAGCTGCCACGCCACAAGGAGTCGATCGAGCTTTGCTGCTGT
AGTACCAACATACATCCATCAAGCCGGAGTAACTACGCTACTGAAGGGAACCAACCAAATGCATGCAA
CTTAGACTGCTGGAAGAACTAATTGAATCAACAGATGATGGGCAACCAACAAGTTATGTTTATTGGGC
CTTGGACGTCAGAATACAACGTTAACAACCAATATCCCTACCTGAGCATTCCACACGTATTTTTAATT
CAGGTATAACCCTCTCTATTCATTCATCAATGCACTCCCCTATCCTTGAATAGGGATGGATGTACGAG
CCCTGACTAAATCCCGAACTGAACACTGAGTAATACTCGGGAAGAAGTGTCTGTCGATATCCCTCACT
CACTGGCTCGCTGTATGGATGGATCGACTCTTCAATAGATATATGGATATATGTGTGAAGTCCCGTTT
CCAAAGTCTGAATTCCAAGAATGCCTCCTCTCCCAGAATACGTTTCCTCCATGCACCCATGCGATCGA
TCAATCTATCGAGCAAGAAGCACGTAAGTAATAGCCTCAATCTTCTAGCCGGCCGGCTACCTCCCATT

TGACCCATCCATCCCATCCAGGTTGGAATTGGTAAGGTCGATCCGAAAAACCCAGATACGTCGAAAAG
CAAACTTTGCATATCTGGGTTCAGAGGCAATTGATCCTCCATCCGGGTCGATAATTAAAATTTGTATT
GAGCCAAAATTGCTGATTTAAACCCTTTTAAGTTATTACCCATTTCCCTCCATCTAATCCGGCGGGTA
GGAAACCTACGTAAGTATCAAATATATCTCCTGAAATCTCCCATGCTTTACGAAAGAAATGATGCCCC
AACTGGATATATCCGCTTATTTAACACAATTCTTCCGGTTATGCCTGTTCTGTTAGGTGGTGAGGCTA
TGTCGTCGGGTGACTATGTCGGTTGTCCCCTTGCTGGTGCGGTAGTTCGTTCCGGGTGGATGCGGCGT
GGTGAAAGCCGGGTATTGTGGGGCCTTATTATCCTCTTGGCCAATGGTGACGTGGGCTGTGATCCGTC
CGGTGTACCGCCTAACCTCAACTACGTAGGGGCGGGTAGGGTCCGGAAGGCAAGGACTAGGCACGCTG
GGCGAAACCTAAAAATGTGGTGCCTGGGCCGAATCTGTTGGCTCTGTGAGGATCGAGGGCCATGGTGG
GGTAGAGGTATAGGTTCTAGGTCTGGCTACTATGGCGAGGTCTGGTGGTAAGATTTCTTGTTCCTCAT
TCGTTGTTCCGTGGAGCACTTCTCTCAAAATATGTATCCTGTGCCCGGTGGAGCGCCCATTTGGAAAG
ATGTGATTTATCGCGAATCAATCAATTTGATCATCTCGCGTTTACATGTCTGTTGTCTGCCATCCTCT
TATTTGGTGCGCCCCTCTCAGAAGCCATTCAGTCTGGATAAAGAAATCAATATAAAAGCCCAACCACT
TCCCTTACTGTACCTCTTTCTTCCTAGCGTGAACTCAAGCATATGCCTGTCCTAGTATCCTTAGGTGC
GCAAGTAATGATCATAATTCCTACAATGCTCCTAACCATTAATCCGAGCCTAGTAGCCCAGCTCGAGT
ATCCAAAGCCGAGCAGCCAAACTGAGCAGCTCCGTGCATTCCAGCCCACCCAGGTAATAACATTTTAA
AGGCTCTGTATCGGTGAG
>Repeat_21
GCTGCTGGATCCACTTAATATACAAGCTTCTGCTTAACCTGGACCTGGAACTGCTCGTGCGGCATCAT
CTGAACCTGTAACTGCTCTTGCTGCTTCTTCTGTTACCTATGCCTTTTCCTATGGTGGTGATTATTAC
TTAGGGTTTTAGGGTGCTTCCACTGCTGCTTTTGGTAGTGCTGCTTCCGCTTCTAGCTTTCGAGATGG
TGGAAGGTACGCTCGCTCTCGCTCTGCAGAATATACTTAAACAACATAGTCGTAAACCAACCGAATCA
ACCTCCGCCTCGGATGCCTTTGCTTCTGGGTTACGGGTAACTGGTTCAAGGTAAACAACTTAACCTAG
TACTTTTGCTTCTAATGCCTCCGCCTTTATTTCTGCGACCTTTGCTTCCATCCGTGGTGCTTCATCTG
GATCATCAGGAACTGCATCACGAACTTCATAATATCGATCACGAACACGAACATCAGACTCTGGCTAT
CGATCTGGATTATCTGAATCTGGATATGGATCCTAGCGAAGGGAAGCTGGTTTTGAAACTGGATCGAC
GGGCAACTGGAACTGGAACTACTCAAAGGGATGTGGGCTCTGTCCCTGCTTACGCCTTTGTCTACGGT
ACCTTTGCTATTAGTCGTTATGGTGCCTAAACCACTGCTTCTTATGCTTGGTAAGCTGCTTAAACTCT
TCAGAATTACCGCAGCTACTGGTGCATCTATCTTAGCTATTGGTGCTCCTACCCTTGCTTCTGCGCAT
GATGTCTCTATATATTATGCTACCTTCGCTCCTGCTGCCCTAGATCTTTGCTGCATCTGTCTTTCCTA
GAACAGCGTAAAGTAAAGCTACCTTTGTATCCGCTGGATCTCCTGCTGCTTACTCAGCCACTGGATCG
CCTCTGTTACCTTCTCCATTGGTGGTGCCTATGCCTTTGTAGGCTATGCGTATGGATATGTTCGAGGA
CCCAGATGTAGCGTTGGTCGGTCAGCTGAATCATAAGAATCAACAGACTCTACATCGACAGCATAATC
ATCAAAGCCAGGTAGTGAAAGGAATGATGAAATTCCATAGCCTGGTGCGCGGAAAGAGAGATGCCTTG
GCCTTTTGCCAAGTACACTAAGTCCCGCAAATGCCAGCCTTGAACCTGTTGAAAGAAGCCTTTCACCT
TCTGCGCTTTGCTTATGACCACGCTTGTTGACCATGCATGTGGAAGCCTCGACCAGACCCCTATCCCT
TGTCTGAGGTAACCGAAAGCGAGCTGAGCTGCCGGCATATATCTCTTGGCCCGGCTACTCGTTTTTGA
CTCCCACCCACAACTACTTGGTGATGCCCTTGCTTTTACTGGGCCTGGAACTGGAAATGGATATGCTG
GGCGAGATGCTGCGGGTGCTTCTGTGACTGGGTCTTGCTTTCTATAGGATATGGATGGATCTCGCTCA
CGAGGGCTTGGATCACGCTGTAGATCATCGGTAGGAACTAGGTCTCGATCACGATCTGGATCTTCATC
TGGAGCGGAACTGGACCAACTCAACTGCGGAATCGAAAGAGTCCAACTGGCTATGCCCTCTCCTTTGT
GTGGGTGCTTCCCTTGCTTTTGCTTATGGTACTACCTTAGCTACCGGTGGTGGTACCTCGGGTTATGC
CTTTGTATCTGCAGCCATCCGCACCTATGCTTCCGCAACTGATGGTAGATCCACTGACTCTGCTGTCC
CTCTAGATGCCTTTGCCCTAGCATCTTTCACCGTAGCCTCTGCCCCAACCTTTGCCTCTGCTTACTCT
GTAACCTACGCTACTTACTATGCAGCTGCGGGTGAATCTGCTCGCTATGAATCTATAACTGGAGGGTG
TCAAACGGAAACATAAACAACTGGATCAATTAGCGTGGGATCCGAATCTTTATTATCTGTGCCCGGAG
CTCTTTCTCTGCTGGTTTTGGGACTGGCTCTCGAACGGTAAGGAATGCTCGCTCTGTTATTGGTAATG
CTTATTACTCTGGATCTTGATATGGAATCGGAAAAATGGACAGTGGGGAAGCTAGCACCGGGTAAACT
CCAACCGGATCCGCTATGGAACCTGCTGGATATAGATCTGGATCTATTGGTGTTGGCTCTTAGTCAAC
TGGTTCCAGGTCAACAACTGCTGAGGGGTCACCTGGATGAGAAACTGCCTTATTAGCTGCCTTTGTAT
CCCCTGCCTTTGCCTTAAATACGTAATATTCAAAGATCTTAGGTAAGGTTTGAATTTGTTCCCCGGAT
CGATTTCCAGATGGTCGCTAGGTAGGGGCAAAAAGTGCGAGGTGGGGCGCGGCGAAAGATATCGGTTA
AGGTATACTTGGTGAACGTAGGGAGCTACCCGTGTGGATCCGCTCGAACTCTAACTGGGTCTGCCGGA
ACTACTACCGTTACCACCTAGAATAGGTGCTGATCAATTGTGCTTCTCTTATGGATATGTGCCTCCGC
CTCTGATGCTGCTTGGTTATGTCGGGTCGGTCGGTGTTAGTGAGCTGCGCTTACCCTATGTGTGGTTC
GATCGGTTATTGGAAATCAATTTGTTCGTGTCGTTTCTTCGGCGAGCAGGTGCCTGCGATCGATCGGT
GTTGCTTCTATTCGTGCTTGGTGCTTTCCGGCCGATGTGTTCTTCCCTTTCCTTGTCGATGCTTTGTA

TTTCCCCCCGATGCTCTGTCCCTCCCTCCCCTTCCTTTCTCGCTGCGGGCCTTCGATCTCGATCTGCA
GGGTCTGGATCTCGGTCTAAAAATGTATCTGCGTTGATAGCTGTGGGCTGATGGTGGTGCCCCTTCCT
TCCCTGCGGCCTTCACCTTTGTGGGTGGTGCTTTCACCGCTCTACTGAAACTAGTGGGGGTGCTACCT
TTCCTCCTGGTGCCTTCGCTTATGGCTTTGACTTTGCAACCGAAGAAGCCGCCTATACGGAATCAACT
TCACCAGGTCCTAATACCATCTATGCCATTATTTACGCCTTTGCTCCTAGAGATGATGCAGAGACAAG
AGAGGTAGAGCCATCACCACCTTGCCCCAGAATACCTTCCGGTTCCATAGGCATTTAACCAAGTGAAA
GCGCCAGTTGATCTTGATCTATAAGCATAAACGACAGCAGTCATTGACCCAGATACAGATCCATAGCG
CGCGGCATCCCTTCCAGTTACATATCCATTTCGAAAGCCAGAGCCCGTAGTTCCACCAGCAGATGACA
TGGATCCAGCGCCATATGACCGAGGAGCGAATTCACCAGTGGCAGAGCGAAAGGCATAGCTATCAAAG
AGCAAAGGGGCCAGTTAGTATTCATAGGCCAGTATTTTACATATAAACATCTATAAAGCAGTACGGGA
AACACTATAGATAGATAGAAAGCCGCCGCTTTTGAATCCTTCCAACTATTCACACCTATATTCGAAGA
CACCTGGTAGGGAAGGAGTAGGGAAGGGAGCAGAGACAGGAAAAGCAAAGGTAGTGCGGGTGGTAAAG
CTTATTACATACCTACAATAGTTTATCACCCGTCCAAGGAGCATCACTAGCACGCATAGGAGGAAAAA
TACATCCCACGTAGACACCTTATTTCGGTCGAAAACGTACTGATTGCCCATTTCACAGGGAGAAGAAC
GCATGTTACCCAGTGGATGGATGCGAGTAAAAGGTACGTTTCATGGCCCAATATACCCTGATCCATAG
GAGGTAATCTCGCCGGGTCATTTCTTTGAGTCGCTAAGTGGTTCAAGCTAAGTGGGGAAAGAGAGCCA
GACGGAGAATCTCTTGCTTGTTAAGCCGGGTGGAAAATAGATTATTTCATTGGTTATCCTCTTTGTGC
TCTTTCTTTCTAATTATTCCCACCCACAACCCTTGCTTTAAGACACTATGGTGAAACTGGTAAGATAC
CATGACACTATGGTTTAATACGGCACGGAGGACTATTGGTGTTTTTATGCGCTTAAAGACTGAACTAC
GTACAGGGTGCTTTGATTGACTGGAATAGAGACGACGAAAGGACACTTTGACTCATTGGTTATCTAAT
AAACCTCTCTTTGAGCCCGTTTCATAAACACCCTACGCATAAGTAGAGCCAAGGACAAAGGCCCGGGT
GGATCTGCTGTTGGGACTGGCGTTGGTGCGCGCTTCAATGGATTTCCTTCACAACTTGAATTGATTTC
TTTCATTGGATCCCCTAGAAGCTATAGCAATTCAGTGGACTAGGGAGAGGAACAGACCCGGTTAGCTT
CTTTCGTTTACTTTTCCTGATGCCCCATGGGCAGAGGTCAAGGTAAAGGTAGCTAGAACAGTTCCATA
CCCAGAAGCAGGTGCATAAGCAAGGGCAGTAGCATATCCAGTTTTTGACTCAGTAGCACTAGCAGCAG
ATCCAGACGCATATCCAGATCCTCTAGCAAAGCCAGGTGTTCCATACCCAACATCTATACTACTAGTA
GATTCTGATGTTGCTTATGAGTTTGATCCAGAAATCCAGTAGCATCAGTACGAGAGTCAGTAGTTCCA
GTTCCTGATTCTTATGTTCCAGATCGAGATACAGTAGCAGAGGAAGCAGATGGTGAGTCAGTAGAGCG
AGATGGAGATTCTGTCTTTCTAGATCGATACCCTTTTGAACTTGTTTCAAAATAGGGGGCCAGCAAGA
GCAGTTCCATACGCGGCAGAGCTTCCAATAGTGGACTTAACTTCCCCACTAGTCGCGGCTAGATCGAA
ACCCAGACCCTTTCAAAATAGGGGGCCAGCTTCAGTCGCAGGAGAAGCGGGGGTACCTAGAGCAGTAG
ATCCAGTCGAAGAACCAGCAGGTGTAGCAGCTCAATTAATAAGGGGGGCTGGAGATGCATAGAAAGAT
CCAGGACCAGCATAACCTATTGATTCAGGTGGTTCAATAGCAGATTCTGTCGAGGTAGTTGATTCAGT
CGTTATAGGCGCGAAAGCGGGAGAGAATTTCCTCCTCACGAAGCCGGTTTAGATCAGGCCTTTATATA
CGTCATTTCGGGCAGGCTTTATATCTACCTTTAGCCTATATGAGGCAAGTGCGAAAGCGTAATTACGT
ATATAATGAGGG
>Repeat_22
CTCATTATATACGTAATTACGCTTTCGCACTTGCCTCATATAGGCTAAAGGTAGATATAAAGCCTGCC
CGAAATGACGTATATAAAGGCCTGATCTAAACCGGCTTCGTGAGGAGGAAATTCTCTCCCGCTTTCGC
GCCTATAACGACTGAATCAACTACCTCGACAGAATCTGCTATTGAACCACCTGAATCAATAGGTTATG
CTGGTCCTGGATCTTTCTATGCATCTCCAGCCCCCCTTATTAATTGAGCTGCTACACCTGCTGGTTCT
TCGACTGGATCTACTGCTCTAGGTACCCCCGCTTCTCCTGCGACTGAAGCTGGCCCCCTATTTTGAAA
GGGTCTGGGTTTCGATCTAGCCGCGACTAGTGGGGAAGTTAAGTCCACTATTGGAAGCTCTGCCGCGT
ATGGAACTGCTCTTGCTGGCCCCCTATTTTGAAACAAGTTCAAAAGGGTATCGATCTAGAAAGACAGA
ATCTCCATCTCGCTCTACTGACTCACCATCTGCTTCCTCTGCTACTGTATCTCGATCTGGAACATAAG
AATCAGGAACTGGAACTACTGACTCTCGTACTGATGCTACTGGATTTCTGGATCAAACTCATAAGCAA
CATCAGAATCTACTAGTAGTATAGATGTTGGGTATGGAACACCTGGCTTTGCTAGAGGATCTGGATAT
GCGTCTGGATCTGCTGCTAGTGCTACTGAGTCAAAAACTGGATATGCTACTGCCCTTGCTTATGCACC
TGCTTCTGGGTATGGAACTGTTCTAGCTACCTTTACCTTGACCTCTGCCCATGGGGCATCAGGAAAAG
TAAACGAAAGAAGCTAACCGGGTCTGTTCCTCTCCCTAGTCCACTGAATTGCTATAGCTTCTAGGGGA
TCCAATGAAAGAAATCAATTCAAGTTGTGAAGGAAATCCATTGAAGCGCGCACCAACGCCAGTCCCAA
CAGCAGATCCACCCGGGCCTTTGTCCTTGGCTCTACTTATGCGTAGGGTGTTTATGAAACGGGCTCAA
AGAGAGGTTTATTAGATAACCAATGAGTCAAAGTGTCCTTTCGTCGTCTCTATTCCAGTCAATCAAAG
CACCCTGTACGTAGTTCAGTCTTTAAGCGCATAAAAACACCAATAGTCCTCCGTGCCGTATTAAACCA
TAGTGTCATGGTATCTTACCAGTTTCACCATAGTGTCTTAAAGCAAGGGTTGTGGGTGGAATAATTA
GAAAGAAAGAGCACAAAGAGGATAACCAATGAAATAATCTATTTTCCACCCGGCTTAACAAGCAAGAG
ATTCTCCGTCTGGCTCTCTTTCCCCACTTAGCTTGAACCACTTAGCGACTCAAAGAATGACCCGGCG

AGATTACCTCCTATGGATCAGGGTATATTGGGCCATGAAACGTACCTTTTACTCGCATCCATCCACTG
GGTAACATGCGTTCTTCTCCCTGTGAAATGGGCAATCAGTACGTTTTCGACCGAAATAAGGTGTCTAC
GTGGGATGTATTTTTCCTCCTATGCGTGCTAGTGATGCTCCTTGGACGGGTGATAAACTATTGTAGGT
ATGTAATAAGCTTTACCACCCGCACTACCTTTGCTTTTCCTGTCTCTGCTCCCTTCCCTACTCCTTCC
CTACCAGGTGTCTTCGAATATAGGTGTGAATAGTTGGAAGGATTCAAAAGCGGCGGCTTTCTATCTAT
CTATAGTGTTTCCCGTACTGCTTTATAGATGTTTATATGTAAAATACTGGCCTATGAATACTAACTGG
CCCCTTTGCTCTTTGATAGCTATGCCTTTCGCTCTGCCACTGGTGAATTCGCTCCTCGGTCATATGGC
GCTGGATCCATGTCATCTGCTGGTGGAACTACGGGCTCTGGCTTTCGAAATGGATATGTAACTGGAAG
GGATGCCGCGCGCTATGGATCTGTATCTGGGTCAATGACTGCTGTCGTTTATGCTTATAGATCAAGAT
CAACTGGCGCTTTCACTTGGTTAAATGCCTATGGAACCGGAAGGTATTCTGGGGCAAGGTGGTGATGG
CTCTACCTCTCTTGTCTCTGCATCATCTCTAGGAGCAAAGGCGTAAATAATGGCATAGATGGTATTAG
GACCTGGTGAAGTTGATTCCGTATAGGCGGCTTCTTCGGTTGCAAAGTCAAAGCCATAAGCGAAGGCA
CCAGGAGGAAAGGTAGCACCCCCACTAGTTTCAGTAGAGCGGTGAAAGCACCACCCACAAAGGTGAAG
GCCGCAGGGAAGGAAGGGGCACCACCATCAGCCCACAGCTATCAACGCAGATACATTTTTAGACCGAG
ATCCAGACCCTGCAGATCGAGATCGAAGGCCCGCAGCGAGAAAGGAAGGGGAGGGAGGGACAGAGCAT
CGGGGGGAAATACAAAGCATCGACAAGGAAAGGGAAGAACACATCGGCCGGAAAGCACCAAGCACGAA
TAGAAGCAACACCGATCGATCGCAGGCACCTGCTCGCCGAAGAAACGACACGAACAAATTGATTTCCA
ATAACCGATCGAACCACACATAGGGTAAGCGCAGCTCACTAACACCGACCGACCCGACATAACCAAGC
AGCATCAGAGGCGGAGGCACATATCCATAAGAGAAGCACAATTGATCAGCACCTATTCTAGGTGGTAA
CGGTAGTAGTTCCGGCAGACCCAGTTAGAGTTCGAGCGGATCCACACGGGTAGCTCCCTACGTTCACC
AAGTATACCTTAACCGATATCTTTCGCCGCGCCCCACCTCGCACTTTTTGCCCCTACCTAGCGACCAT
CTGGAAATCGATCCGGGGAACAAATTCAAACCTTACCTAAGATCTTTGAATATTACGTATTTAAGGCA
AAGGCAGGGGATACAAAGGCAGCTAATAAGGCAGTTTCTCATCCAGGTGACCCCTCAGCAGTTGTTGA
CCTGGAACCAGTTGACTAAGAGCCAACACCAATAGATCCAGATCTATATCCAGCAGGTTCCATAGCGG
ATCCGGTTGGAGTTTACCCGGTGCTAGCTTCCCCACTGTCCATTTTTCCGATTCCATATCAAGATCCA
GAGTAATAAGCATTACCAATAACAGAGCGAGCATTCCTTACCGTTCGAGAGCCAGTCCCAAAACCAGC
AGAGAAAGAGCTCCGGGCACAGATAATAAAGATTCGGATCCCACGCTAATTGATCCAGTTGTTTATGT
TTCCGTTTGACACCCTCCAGTTATAGATTCATAGCGAGCAGATTCACCCGCAGCTGCATAGTAAGTAG
CGTAGGTTACAGAGTAAGCAGAGGCAAAGGTTGGGGCAGAGGCTACGGTGAAAGATGCTAGGGCAAAG
GCATCTAGAGGGACAGCAGAGTCAGTGGATCTACCATCAGTTGCGGAAGCATAGGTGCGGATGGCTGC
AGATACAAAGGCATAACCCGAGGTACCACCACCGGTAGCTAAGGTAGTACCATAAGCAAAAGCAAGGG
AAGCACCCACACAAAGGAGAGGGCATAGCCAGTTGGACTCTTTCGATTCCGCAGTTGAGTTGGTCCAG
TTCCGCTCCAGATGAAGATCCAGATCGTGATCGAGACCTAGTTCCTACCGATGATCTACAGCGTGATC
CAAGCCCTCGTGAGCGAGATCCATCCATATCCTATAGAAAGCAAGACCCAGTCACAGAAGCACCCGCA
GCATCTCGCCCAGCATATCCATTTCCAGTTCCAGGCCCAGTAAAAGCAAGGGCATCACCAAGTAGTTG
TGGGTGGGAGTCAAAAACGAGTAGCCGGGCCAAGAGATATATGCCGGCAGCTCAGCTCGCTTTCGGTT
ACCTCAGACAAGGGATAGGGGTCTGGTCGAGGCTTCCACATGCATGGTCAACAAGCGTGGTCATAAGC
AAAGCGCAGAAGGTGAAAGGCTTCTTTCAACAGGTTCAAGGCTGGCATTTGCGGGACTTAGTGTACTT
GGCAAAAGGCCAAGGCATCTCTCTTTCCGCGCACCAGGCTATGGAATTTCATCATTCCTTTCACTACC
TGGCTTTGATGATTATGCTGTCGATGTAGAGTCTGTTGATTCTTATGATTCAGCTGACCGACCAACGC
TACATCTGGGTCCTCGAACATATCCATACGCATAGCCTACAAAGGCATAGGCACCACCAATGGAGAAG
GTAACAGAGGCGATCCAGTGGCTGAGTAAGCAGCAGGAGATCCAGCGGATACAAAGGTAGCTTTACTT
TACGCTGTTCTAGGAAAGACAGATGCAGCAAAGATCTAGGGCAGCAGGAGCGAAGGTAGCATAATATA
TAGAGACATCATGCGCAGAAGCAAGGGTAGGAGCACCAATAGCTAAGATAGATGCACCAGTAGCTGCG
GTAATTCTGAAGAGTTTAAGCAGCTTACCAAGCATAAGAAGCAGTGGTTTAGGCACCATAACGACTAA
TAGCAAAGGTACCGTAGACAAAGGCGTAAGCAGGGACAGAGCCCACATCCCTTTGAGTAGTTCCAGTT
CCAGTTGCCCGTCGATCCAGTTTCAAAACCAGCTTCCCTTCGCTAGGATCCATATCCAGATTCAGATA
ATCCAGATCGATAGCCAGAGTCTGATGTTCGTGTTCGTGATCGATATTATGAAGTTCGTGATGCAGTT
CCTGATGATCCAGATGAAGCACCACGGATGGAAGCAAAGGTCGCAGAAATAAAGGCGGAGGCATTAGA
AGCAAAAGTACTAGGTTAAGTTGTTTACCTTGAACCAGTTACCCGTAACCCAGAAGCAAAGGCATCCG
AGGCGGAGGTTGATTCGGTTGGTTTACGACTATGTTGTTTAAGTATATTCTGCAGAGCGAGAGCGAGC
GTACCTTCCACCATCTCGAAAGCTAGAAGCGGAAGCAGCACTACCAAAAGCAGCAGTGGAAGCACCCCT
AAAACCCTAAGTAATAATCACCACCATAGGAAAAGGCATAGGTAACAGAAGAAGCAGCAAGAGCAGTT
ACAGGTTCAGATGATGCCGCACGAGCAGTTCCAGGTCCAGGTTAAGCAGAAGCTTGTATATTAAGTGG
ATCCAGCAGCC
>Repeat_23

GCTGCTGGATCCACTTAATATACAAGCTTCTGCTTAACCTGGACCTGGAACTGCTCGTGCGGCATCAT
CTGAACCTGTAACTGCTCTTGCTGCTTCTTCTGTTACCTATGCCTTTTCCTATGGTGGTGATTATTAC
TTAGGGTTTTAGGGTGCTTCCACTGCTGCTTTTGGTAGTGCTGCTTCCGCTTCTAGCTTTCGAGATGG
TGGAAGGTACGCTCGCTCTCGCTCTGCAGAATATACTTAAACAACATAGTCGTAAACCAACCGAATCA
ACCTCCGCCTCGGATGCCTTTGCTTCTGGGTTACGGGTAACTGGTTCAAGGTAAACAACTTAACCTAG
TACTTTTGCTTCTAATGCCTCCGCCTTTATTTCTGCGACCTTTGCTTCCATCCGTGGTGCTTCATCTG
GATCATCAGGAACTGCATCACGAACTTCATAATATCGATCACGAACACGAACATCAGACTCTGGCTAT
CGATCTGGATTATCTGAATCTGGATATGGATCCTAGCGAAGGGAAGCTGGTTTTGAAACTGGATCGAC
GGGCAACTGGAACTGGAACTACTCAAAGGGATGTGGGCTCTGTCCCTGCTTACGCCTTTGTCTACGGT
ACCTTTGCTATTAGTCGTTATGGTGCCTAAACCACTGCTTCTTATGCTTGGTAAGCTGCTTAAACTCT
TCAGAATTACCGCAGCTACTGGTGCATCTATCTTAGCTATTGGTGCTCCTACCCTTGCTTCTGCGCAT
GATGTCTCTATATATTATGCTACCTTCGCTCCTGCTGCCCTAGATCTTTGCTGCATCTGTCTTTCCTA
GAACAGCGTAAAGTAAAGCTACCTTTGTATCCGCTGGATCTCCTGCTGCTTACTCAGCCACTGGATCG
CCTCTGTTACCTTCTCCATTGGTGGTGCCTATGCCTTTGTAGGCTATGCGTATGGATATGTTCGAGGA
CCCAGATGTAGCGTTGGTCGGTCAGCTGAATCATAAGAATCAACAGACTCTACATCGACAGCATAATC
ATCAAAGCCAGGTAGTGAAAGGAATGATGAAATTCCATAGCCTGGTGCGCGGAAAGAGAGATGCCTTG
GCCTTTTGCCAAGTACACTAAGTCCCGCAAATGCCAGCCTTGAACCTGTTGAAAGAAGCCTTTCACCT
TCTGCGCTTTGCTTATGACCACGCTTGTTGACCATGCATGTGGAAGCCTCGACCAGACCCCTATCCCT
TGTCTGAGGTAACCGAAAGCGAGCTGAGCTGCCGGCATATATCTCTTGGCCCGGCTACTCGTTTTTGA
CTCCCACCCACAACTACTTGGTGATGCCCTTGCTTTTACTGGGCCTGGAACTGGAAATGGATATGCTG
GGCGAGATGCTGCGGGTGCTTCTGTGACTGGGTCTTGCTTTCTATAGGATATGGATGGATCTCGCTCA
CGAGGGCTTGGATCACGCTGTAGATCATCGGTAGGAACTAGGTCTCGATCACGATCTGGATCTTCATC
TGGAGCGGAACTGGACCAACTCAACTGCGGAATCGAAAGAGTCCAACTGGCTATGCCCTCTCCTTTGT
GTGGGTGCTTCCCTTGCTTTTGCTTATGGTACTACCTTAGCTACCGGTGGTGGTACCTCGGGTTATGC
CTTTGTATCTGCAGCCATCCGCACCTATGCTTCCGCAACTGATGGTAGATCCACTGACTCTGCTGTCC
CTCTAGATGCCTTTGCCCTAGCATCTTTCACCGTAGCCTCTGCCCCAACCTTTGCCTCTGCTTACTCT
GTAACCTACGCTACTTACTATGCAGCTGCGGGTGAATCTGCTCGCTATGAATCTATAACTGGAGGGTG
TCAAACGGAAACATAAACAACTGGATCAATTAGCGTGGGATCCGAATCTTTATTATCTGTGCCCGGAG
CTCTTTCTCTGCTGGTTTTGGGACTGGCTCTCGAACGGTAAGGAATGCTCGCTCTGTTATTGGTAATG
CTTATTACTCTGGATCTTGATATGGAATCGGAAAAATGGACAGTGGGGAAGCTAGCACCGGGTAAACT
CCAACCGGATCCGCTATGGAACCTGCTGGATATAGATCTGGATCTATTGGTGTTGGCTCTTAGTCAAC
TGGTTCCAGGTCAACAACTGCTGAGGGGTCACCTGGATGAGAAACTGCCTTATTAGCTGCCTTTGTAT
CCCCTGCCTTTGCCTTAAATACGTAATATTCAAAGATCTTAGGTAAGGTTTGAATTTGTTCCCCGGAT
CGATTTCCAGATGGTCGCTAGGTAGGGGCAAAAAGTGCGAGGTGGGGCGCGGCGAAAGATATCGGTTA
AGGTATACTTGGTGAACGTAGGGAGCTACCCGTGTGGATCCGCTCGAACTCTAACTGGGTCTGCCGGA
ACTACTACCGTTACCACCTAGAATAGGTGCTGATCAATTGTGCTTCTCTTATGGATATGTGCCTCCGC
CTCTGATGCTGCTTGGTTATGTCGGGTCGGTCGGTGTTAGTGAGCTGCGCTTACCCTATGTGTGGTTC
GATCGGTTATTGGAAATCAATTTGTTCGTGTCGTTTCTTCGGCGAGCAGGTGCCTGCGATCGATCGGT
GTTGCTTCTATTCGTGCTTGGTGCTTTCCGGCCGATGTGTTCTTCCCTTTCCTTGTCGATGCTTTGTA
TTTCCCCCCGATGCTCTGTCCCTCCCTCCCCTTCCTTTCTCGCTGCGGGCCTTCGATCTCGATCTGCA
GGGTCTGGATCTCGGTCTAAAAATGTATCTGCGTTGATAGCTGTGGGCTGATGGTGGTGCCCCTTCCT
TCCCTGCGGCCTTCACCTTTGTGGGTGGTGCTTTCACCGCTCTACTGAAACTAGTGGGGGTGCTACCT
TTCCTCCTGGTGCCTTCGCTTATGGCTTTGACTTTGCAACCGAAGAAGCCGCCTATACGGAATCAACT
TCACCAGGTCCTAATACCATCTATGCCATTATTTACGCCTTTGCTCCTAGAGATGATGCAGAGACAAG
AGAGGTAGAGCCATCACCACCTTGCCCCAGAATACCTTCCGGTTCCATAGGCATTTAACCAAGTGAAA
GCGCCAGTTGATCTTGATCTATAAGCATAAACGACAGCAGTCATTGACCCAGATACAGATCCATAGCG
CGCGGCATCCCTTCCAGTTACATATCCATTTCGAAAGCCAGAGCCCGTAGTTCCACCAGCAGATGACA
TGGATCCAGCGCCATATGACCGAGGAGCGAATTCACCAGTGGCAGAGCGAAAGGCATAGCTATCAAAG
AGCAAAGGGGCCAGTTAGTATTCATAGGCCAGTATTTTACATATAAACATCTATAAAGCAGTACGGGA
AACACTATAGATAGATAGAAAGCCGCCGCTTTTGAATCCTTCCAACTATTCACACCTATATTCGAAGA
CACCTGGTAGGGAAGGAGTAGGGAAGGGAGCAGAGACAGGAAAAGCAAAGGTAGTGCGGGTGGTAAAG
CTTATTACATACCTACAATAGTTTATCACCCGTCCAAGGAGCATCACTAGCACGCATAGGAGGAAAAA
TACATCCCACGTAGACACCTTATTTCGGTCGAAAACGTACTGATTGCCCATTTCACAGGGAGAAGAAC
GCATGTTACCCAGTGGATGGATGCGAGTAAAAGGTACGTTTCATGGCCCAATATACCCTGATCCATAG
GAGGTAATCTCGCCGGGTCATTTCTTTGAGTCGCTAAGTGGTTCAAGCTAAGTGGGGAAAGAGAGCCA
GACGGAGAATCTCTTGCTTGTTAAGCCGGGTGGAAAATAGATTATTTCATTGGTTATCCTCTTTGTGC
TCTTTCTTTCTAATTATTCCCACCCACAACCCTTGCTTTAAGACACTATGGTGAAACTGGTAAGATAC

CATGACACTATGGTTTAATACGGCACGGAGGACTATTGGTGTTTTTATGCGCTTAAAGACTGAACTAC
GTACAGGGTGCTTTGATTGACTGGAATAGAGACGACGAAAGGACACTTTGACTCATTGGTTATCTAAT
AAACCTCTCTTTGAGCCCGTTTCATAAACACCCTACGCATAAGTAGAGCCAAGGACAAAGGCCCGGGT
GGATCTGCTGTTGGGACTGGCGTTGGTGCGCGCTTCAATGGATTTCCTTCACAACTTGAATTGATTTC
TTTCATTGGATCCCCTAGAAGCTATAGCAATTCAGTGGACTAGGGAGAGGAACAGACCCGGTTAGCTT
CTTTCGTTTACTTTTCCTGATGCCCCATGGGCAGAGGTCAAGGTAAAGGTAGCTAGAACAGTTCCATA
CCCAGAAGCAGGTGCATAAGCAAGGGCAGTAGCATATCCAGTTTTTGACTCAGTAGCACTAGCAGCAG
ATCCAGACGCATATCCAGATCCTCTAGCAAAGCCAGGTGTTCCATACCCAACATCTATACTACTAGTA
GATTCTGATGTTGCTTATGAGTTTGATCCAGAAATCCAGTAGCATCAGTACGAGAGTCAGTAGTTCCA
GTTCCTGATTCTTATGTTCCAGATCGAGATACAGTAGCAGAGGAAGCAGATGGTGAGTCAGTAGAGCG
AGATGGAGATTCTGTCTTTCTAGATCGATACCCTTTTGAACTTGTTTCAAAATAGGGGGCCAGCAAGA
GCAGTTCCATACGCGGCAGAGCTTCCAATAGTGGACTTAACTTCCCCACTAGTCGCGGCTAGATCGAA
ACCCAGACCCTTTCAAAATAGGGGGCCAGCTTCAGTCGCAGGAGAAGCGGGGGTACCTAGAGCAGTAG
ATCCAGTCGAAGAACCAGCAGGTGTAGCAGCTCAATTAATAAGGGGGGCTGGAGATGCATAGAAAGAT
CCAGGACCAGCATAACCTATTGATTCAGGTGGTTCAATAGCAGATTCTGTCGAGGTAGTTGATTCAGT
CGTTATAGGCGCGAAAGCGGGAGAGAATTTCCTCCTCACGAAGCCGGTTTAGATCAGGCCTTTATATA
CGTCATTTCGGGCAGGCTTTATATCTACCTTTAGCCTATATGAGGCAAGTGCGAAAGCGTAATTACGT
ATATAATGAG
>Repeat_24
ATACAAGCTTCTGCTTAACCTGGACCTGGAACTGCTCGTGCGGCATCATCTGAACCTGTAACTGCTCT
TGCTGCTTCTTCTGTTACCTATGCCTTTTCCTATGGTGGTGATTATTACTTAGGGTTTTAGGGTGCTT
CCACTGCTGCTTTTGGTAGTGCTGCTTCCGCTTCTAGCTTTCGAGATGGTGGAAGGTACGCTCGCTCT
CGCTCTGCAGAATATACTTAAACAACATAGTCGTAAACCAACCGAATCAACCTCCGCCTCGGATGCCT
TTGCTTCTGGGTTACGGGTAACTGGTTCAAGGTAAACAACTTAACCTAGTACTTTTGCTTCTAATGCC
TCCGCCTTTATTTCTGCGACCTTTGCTTCCATCCGTGGTGCTTCATCTGGATCATCAGGAACTGCATC
ACGAACTTCATAATATCGATCACGAACACGAACATCAGACTCTGGCTATCGATCTGGATTATCTGAAT
CTGGATATGGATCCTAGCGAAGGGAAGCTGGTTTTGAAACTGGATCGACGGGCAACTGGAACTGGAAC
TACTCAAAGGGATGTGGGCTCTGTCCCTGCTTACGCCTTTGTCTACGGTACCTTTGCTATTAGTCGTT
ATGGTGCCTAAACCACTGCTTCTTATGCTTGGTAAGCTGCTTAAACTCTTCAGAATTACCGCAGCTAC
TGGTGCATCTATCTTAGCTATTGGTGCTCCTACCCTTGCTTCTGCGCATGATGTCTCTATATATTATG
CTACCTTCGCTCCTGCTGCCCTAGATCTTTGCTGCATCTGTCTTTCCTAGAACAGCGTAAAGTAAAGC
TACCTTTGTATCCGCTGGATCTCCTGCTGCTTACTCAGCCACTGGATCGCCTCTGTTACCTTCTCCAT
TGGTGGTGCCTATGCCTTTGTAGGCTATGCGTATGGATATGTTCGAGGACCCAGATGTAGCGTTGGTC
GGTCAGCTGAATCATAAGAATCAACAGACTCTACATCGACAGCATAATCATCAAAGCCAGGTAGTGAA
AGGAATGATGAAATTCCATAGCCTGGTGCGCGGAAAGAGAGATGCCTTGGCCTTTTGCCAAGTACACT
AAGTCCCGCAAATGCCAGCCTTGAACCTGTTGAAAGAAGCCTTTCACCTTCTGCGCTTTGCTTATGAC
CACGCTTGTTGACCATGCATGTGGAAGCCTCGACCAGACCCCTATCCCTTGTCTGAGGTAACCGAAAG
CGAGCTGAGCTGCCGGCATATATCTCTTGGCCCGGCTACTCGTTTTTGACTCCCACCCACAACTACTT
GGTGATGCCCTTGCTTTTACTGGGCCTGGAACTGGAAATGGATATGCTGGGCGAGATGCTGCGGGTGC
TTCTGTGACTGGGTCTTGCTTTCTATAGGATATGGATGGATCTCGCTCACGAGGGCTTGGATCACGCT
GTAGATCATCGGTAGGAACTAGGTCTCGATCACGATCTGGATCTTCATCTGGAGCGGAACTGGACCAA
CTCAACTGCGGAATCGAAAGAGTCCAACTGGCTATGCCCTCTCCTTTGTGTGGGTGCTTCCCTTGCTT
TTGCTTATGGTACTACCTTAGCTACCGGTGGTGGTACCTCGGGTTATGCCTTTGTATCTGCAGCCATC
CGCACCTATGCTTCCGCAACTGATGGTAGATCCACTGACTCTGCTGTCCCTCTAGATGCCTTTGCCCT
AGCATCTTTCACCGTAGCCTCTGCCCCAACCTTTGCCTCTGCTTACTCTGTAACCTACGCTACTTACT
ATGCAGCTGCGGGTGAATCTGCTCGCTATGAATCTATAACTGGAGGGTGTCAAACGGAAACATAAACA
ACTGGATCAATTAGCGTGGGATCCGAATCTTTATTATCTGTGCCCGGAGCTCTTTCTCTGCTGGTTTT
GGGACTGGCTCTCGAACGGTAAGGAATGCTCGCTCTGTTATTGGTAATGCTTATTACTCTGGATCTTG
ATATGGAATCGGAAAAATGGACAGTGGGGAAGCTAGCACCGGGTAAACTCCAACCGGATCCGCTATGG
AACCTGCTGGATATAGATCTGGATCTATTGGTGTTGGCTCTTAGTCAACTGGTTCCAGGTCAACAACT
GCTGAGGGGTCACCTGGATGAGAAACTGCCTTATTAGCTGCCTTTGTATCCCCTGCCTTTGCCTTAAA
TACGTAATATTCAAAGATCTTAGGTAAGGTTTGAATTTGTTCCCCGGATCGATTTCCAGATGGTCGCT
AGGTAGGGGCAAAAAGTGCGAGGTGGGGCGCGGCGAAAGATATCGGTTAAGGTATACTTGGTGAACGT
AGGGAGCTACCCGTGTGGATCCGCTCGAACTCTAACTGGGTCTGCCGGAACTACTACCGTTACCACCT
AGAATAGGTGCTGATCAATTGTGCTTCTCTTATGGATATGTGCCTCCGCCTCTGATGCTGCTTGGTTA
TGTCGGGTCGGTCGGTGTTAGTGAGCTGCGCTTACCCTATGTGTGGTTCGATCGGTTATTGGAAATCA
ATTTGTTCGTGTCGTTTCTTCGGCGAGCAGGTGCCTGCGATCGATCGGTGTTGCTTCTATTCGTGCTT

GGTGCTTTCCGGCCGATGTGTTCTTCCCTTTCCTTGTCGATGCTTTGTATTTCCCCCCGATGCTCTGT
CCCTCCCTCCCCTTCCTTTCTCGCTGCGGGCCTTCGATCTCGATCTGCAGGGTCTGGATCTCGGTCTA
AAAATGTATCTGCGTTGATAGCTGTGGGCTGATGGTGGTGCCCCTTCCTTCCCTGCGGCCTTCACCTT
TGTGGGTGGTGCTTTCACCGCTCTACTGAAACTAGTGGGGGTGCTACCTTTCCTCCTGGTGCCTTCGC
TTATGGCTTTGACTTTGCAACCGAAGAAGCCGCCTATACGGAATCAACTTCACCAGGTCCTAATACCA
TCTATGCCATTATTTACGCCTTTGCTCCTAGAGATGATGCAGAGACAAGAGAGGTAGAGCCATCACCA
CCTTGCCCCAGAATACCTTCCGGTTCCATAGGCATTTAACCAAGTGAAAGCGCCAGTTGATCTTGATC
TATAAGCATAAACGACAGCAGTCATTGACCCAGATACAGATCCATAGCGCGCGGCATCCCTTCCAGTT
ACATATCCATTTCGAAAGCCAGAGCCCGTAGTTCCACCAGCAGATGACATGGATCCAGCGCCATATGA
CCGAGGAGCGAATTCACCAGTGGCAGAGCGAAAGGCATAGCTATCAAAGAGCAAAGGGGCCAGTTAGT
ATTCATAGGCCAGTATTTTACATATAAACATCTATAAAGCAGTACGGGAAACACTATAGATAGATAGA
AAGCCGCCGCTTTTGAATCCTTCCAACTATTCACACCTATATTCGAAGACACCTGGTAGGGAAGGAGT
AGGGAAGGGAGCAGAGACAGGAAAAGCAAAGGTAGTGCGGGTGGTAAAGCTTATTACATACCTACAAT
AGTTTATCACCCGTCCAAGGAGCATCACTAGCACGCATAGGAGGAAAAATACATCCCACGTAGACACC
TTATTTCGGTCGAAAACGTACTGATTGCCCATTTCACAGGGAGAAGAACGCATGTTACCCAGTGGATG
GATGCGAGTAAAAGGTACGTTTCATGGCCCAATATACCCTGATCCATAGGAGGTAATCTCGCCGGGTC
ATTTCTTTGAGTCGCTAAGTGGTTCAAGCTAAGTGGGGAAAGAGAGCCAGACGGAGAATCTCTTGCTT
GTTAAGCCGGGTGGAAAATAGATTATTTCATTGGTTATCCTCTTTGTGCTCTTTCTTTCTAATTATTC
CCACCCACAACCCTTGCTTTAAGACACTATGGTGAAACTGGTAAGATACCATGACACTATGGTTTAAT
ACGGCACGGAGGACTATTGGTGTTTTTATGCGCTTAAAGACTGAACTACGTACAGGGTGCTTTGATTG
ACTGGAATAGAGACGACGAAAGGACACTTTGACTCATTGGTTATCTAATAAACCTCTCTTTGAGCCCG
TTTCATAAACACCCTACGCATAAGTAGAGCCAAGGACAAAGGCCCGGGTGGATCTGCTGTTGGGACTG
GCGTTGGTGCGCGCTTCAATGGATTTCCTTCACAACTTGAATTGATTTCTTTCATTGGATCCCCTAGA
AGCTATAGCAATTCAGTGGACTAGGGAGAGGAACAGACCCGGTTAGCTTCTTTCGTTTACTTTTCCTG
ATGCCCCATGGGCAGAGGTCAAGGTAAAGGTAGCTAGAACAGTTCCATACCCAGAAGCAGGTGCATAA
GCAAGGGCAGTAGCATATCCAGTTTTTGACTCAGTAGCACTAGCAGCAGATCCAGACGCATATCCAGA
TCCTCTAGCAAAGCCAGGTGTTCCATACCCAACATCTATACTACTAGTAGATTCTGATGTTGCTTATG
AGTTTGATCCAGAAATCCAGTAGCATCAGTACGAGAGTCAGTAGTTCCAGTTCCTGATTCTTATGTTC
CAGATCGAGATACAGTAGCAGAGGAAGCAGATGGTGAGTCAGTAGAGCGAGATGGAGATTCTGTCTTT
CTAGATCGATACCCTTTTGAACTTGTTTCAAAATAGGGGGCCAGCAAGAGCAGTTCCATACGCGGCAG
AGCTTCCAATAGTGGACTTAACTTCCCCACTAGTCGCGGCTAGATCGAAACCCAGACCCTTTCAAAAT
AGGGGGCCAGCTTCAGTCGCAGGAGAAGCGGGGGTACCTAGAGCAGTAGATCCAGTCGAAGAACCAGC
AGGTGTAGCAGCTCAATTAATAAGGGGGGCTGGAGATGCATAGAAAGATCCAGGACCAGCATAACCTA
TTGATTCAGGTGGTTCAATAGCAGATTCTGTCGAGGTAGTTGATTCAGTCGTTATAGGCGCGAAAGCG
GGAGAGAATTTCCTCCTCACGAAGCCGGTTTAGATCAGGCCTTTATATACGTCATTTCGGGCAGGCTT
TATATCTACCTTTAGCCTATATGAGGCAAGTGCGAAAGCGTAATTACGTATATAATGAG
>Repeat_25
GACGCAGTTGACCGAGCACAACCACTAGCAAAGGCAGAACCAAGGGTAGCAATAGATTATGTGGTTTA
TTATGCCCTTGATTAAAGATCCGGCAGAGCCAGTAGATCATCAGTTGGTTCAGTAGATTGAGTAGATT
AAGTAGAAGATTCAGTAGAAGAAGCAACTATTAAATTATGGCGGCGGCGGGAGCAAAGACTAGAGAAG
CAGCAGTCGATTAGACAGTTGACTCAGTCGATCCAGTACCTGCAACCTCACTTCCGTAGAGCAGGCCG
TACCTTTAAATAAGAGGGCAAGAGCTTCTAGGAATGACTGTTTCTAGCCCCTCGGTAGCCAAGCGTAG
TTCCCAAAGCAAGCTATGATCGGCTGACAATGATTGAACACTGATTATATATTCTGGCGCTACAATGA
CGGGCTGGCAATGACCTTCTTCTGACTATGTGATCGACTATGATCGGCTATCGGCTATGATCAGCTGG
CTATTACTGGGTACTAAGACTAGGTTCTATGACTATTGGCTGCAGATCTATACGCAGTTGTTCTATCT
AGAGCCAGTCCAGTAGCGACACCGAGACCGAGCTTCACCAATAGCAAGGCTAGTTGATCCAGTCGAGT
CTGTTGGTTCAGCACCCCTAGCAGATTCAGTAATTCAGAAGTTCAGTGGATTATGCATAGACTCAGCA
GCAGGGAGAAAAATCTGTCTTTATTAGATGCCAATACCACTTAAGGTTCTGGGCCTCTCAATGGGGAA
CTTATAGGTGGACTAAGGCCCAGTAAAAGCAAGGGCATC
>Repeat_26
ATGGATAATCTTTCCCGTGGCAACTTAACTCAATTGATATCTCATTATCGTAGCCACTTGGATAACAT
CATATCTCGCGTCTGTTGGTTCGACGCAACCAATGGTATGGGTTCCATTCTCGGGTTGTTAGGTCTAG
CCACACTCTAGCCACTACCTCAAGAGCATAAGGTTGGCGATAGAACATTTCCCCAACCAGCAGCATGG
CATGTCGCCTTACTTATTGCCTAATCGCCTCACTTTAGCCTTCACAGCCTCACCAAGGACAACAGATG
ACTCACCAGGTAGGTACAACAGAGAGATGCCTTGGAGCCTTTTTGCCGAGAGCCTCGAGCTTTTACCT
ATAACCAAGGGCCTTGAGCCTTTAGACAGTCACCTTCCAGAAGCCATAAGCCTAAAGAATTCCACCAG
AAGTCATCCATTAGACTAGCCCAACTTCTGCCGGAAGCCAATTAGACAAGTCCAACAGCCCACCATAC

AAGCCGTAGACCCGTCCTACCGGCCAGACATAGACCCATCCTACCAGCCGAAGTCACCTTCAACACAC
GCTACATCCACCCAACAAGGAAAGAAGCTACGGCACGAGAAAAGCTACGGCACAGAAGGAAGTTACGG
GACAGGAAGGGGCCGGCATACTCGCCTCATATAAGTCACCCAACGTAGCCCACATAGCCAATCATTGC
CAGAAGGTCATTGTCGATAGCCAATATCATTCACCTACTAATCTCAGTAGTCTTGCTTCCATAGA
>Repeat_27
TACTTAGAAGGAAGGGGAAGAAAGGTATAACCTTGTCGTATCTCGGTGGAATACTTTAAAGAAAGTAA
GGAAGAGGGGAAGAAATAGAAGGTGATCCCAACCCTTCGCGTAGCTCTTTGAGGGGAATAAATATAGG
GATAACCTTTAGAGAGAATTTGCTAGTTCAAAGGTGGCGTTGCGAATGCTTAATTGATTGTTGAACCG
AGCAAACGACTGGTAAATTCAGTACTTAAAGGTGGCCTTTCTCTTTGAGGGACGAGCATGAGCCTAGT
CTTGTTGGTGGTGCCTACCCGCTTGCCCCATGAACCATAAGGATGAAATAGATCTCTCATGCCATGCA
ACACATGCATCGATTTTGCCATCTCTCATGCAACATATATAATGCATCCACAGACAGATCTCAATCCA
ACTGGGAGAAATAGATGCATCCACATTTATCTCTCCATCATGCGCATTTCCTATCCACTCAGCATTCT
CCAAACGCACCGCAATAGAGCGATGACAAAATATGGGGTATTGGTCCGGATCCGATAGCGAGTATATA
CGTGTCTAAGGGCGAGTATAACCCAA
>Repeat_28
AAGGAGCGCGGACTAGCCACGGAGCTTGGATACGGTTTCCCGATCGGAGATCCATGGATCACTAAAGG
TCTCTCCCCATGGCCTTTCGCCTTTGGAAGCGTCCTTCCCTCTCAATGCCCGGGCATCCATCCGATGC
ATTCTTTCCGATACAGTAGGCATTGAACCTGCTTCACACCGCTTTTATTCCCCTCAATCGATCAATCA
CTCAGGACTGACTTAAAGTCCATTTATTCCAGTCATTCTTTATATATGTAATAACACTAGATTCGGTG
TGAGTCGAAGGTTTAAACTCCGTACCCCAGTAGTCTACTTGCACCGGTGACCCACCCGCTTACGGGGT
GCTTTGGGCGGCGCTTTCGCTAACTACTCGTTCTCCTTCGACTTTCGCCGTATCTGTTGGTCATTCGT
TATATACGTAATTACGCTATTGAAGAATTTATAAAAGAATTGATCGGTGCGCACGCAAGCAGGCGAGA
CCAA
>Repeat_29
GTAGCCCAGTCGAGCCTGGTATCCAAGCTGATCCAATTAGCGCAGCAGCCATCCAGGCTGAATAGCCC
AGCTCTCTAATTCATCCCACCCAGAGCTAGGCCAGCAAGCTAATAAGCTCCCCAGCTCCAGCTCCCGA
TCTTAAAAATATCCATCTGGCTCTGGAGCTCCCGATCATCAATCCAACTCACCCACCTGAAGACCTAG
CACCTGTTCTCCCTATACCACTTGTTGAATTAGTCGCTTAGGGTGTTGGTTGTGGTGTCTCTCTCTTC
CTTCTGCCTTCCGTTGTTCCCTGGCTATATTATTAGCTGGTGCTATAGTAGCCTGTAGGATGGAAGTG
GGATTCTTAGGATAAGCCGCCGCCTATATGGTTGTAGCTGTGGGATGTAAAGTTTTAGGTCTCAGGTA
ATAAGTAGTAACCTTCACCGTTTTCTCTGTTCCCTTCTTCGCCTT
>Repeat_30
ATTAGCCGAGTCGTGAAGCTCAGCATCCTATATATAGTTTTCAGTGAGGCGGGAGGGCCAGCGCCTTT
ATTTATGTGCATGCTATCAACCTTTTGTGTTTACCAACTTTGCATATACTGTCTCAATTACCCTACTT
ACTGAGTGAGGCCCCAACAACCTATTGCTTCAGCTAGGAAGGAATGTGAGTACAGCACTTAGTTAGAA
CCTTTACTATGAACCTTGACTCACCGGCCTCCCTTTATCCTTACCAACTACCCACCCGATTCACCAAC
CTTAATCTGTACCTCACCTTGCCCCCAACTTACTGCATTGAATGAACGGCGGAACGGGGCTTCCAATA
AAAGAGATGTTGGACGGGAGGGATACAGACCGCTGCGTTCACCGGCTTAGAGCTGTTGCACCGCCTTC
CAGA
>Repeat_31
AGGGCTCGACCCCATGGCAGCGCTTTACGAACAATGAAAACGAAGCTGCTCGAACATGGTGATCGCCG
AGAAGTAATGAGGTAGCCACCCATAGACAGTATCATTACCGGGAGGTGTCATTGATACACGGTCGGCC
CAGAGACAACTCAGTGCTGATAGCTAGTCGCAGGTGTCGATGCCTTCTTGTCTGTTTTGGAACCGACC
AATGGGTGGACGATGCTAAATACTATCACCTGCTTTTGGGGGCAATGCACCTGTCGAGGATAATGCGC
CAGACACAGAAAGACATTCAGATATGGGAGAGTTGCATTCTCATGCTGCTTAGGTAGTTAGCGTCATT
CACGCAAATAGGGTAAGTCATGCTGGTTCTAAACAATAACAAAAAGAAGATCCAAAAGGGT
>Repeat_32
CTGATTATATATTCTGGCGCTACAATGACGGGCTGGCAATGACCTTCTTCTGACTATGTGATCGACTA
TGATCGGCTATCGGCTATGATCAGCTGGCTATTACTGGGTACTAAGACTAGGTTCTATGACTATTGGC
TGCAGATCTATACGCAGTTGTTCTATCTAGAGCCAGTCCAGTAGCGACACCGAGACCGAGCTTCACCA
ATAGCAAGGCTAGTTGATCCAGTCGAGTCTGTTGGTTCAGCACCCCTAGCAGATTCAGTAATTCAGAA
GTTCAGTGGATTATGCATAGACTCAGCAGCAGGGAGAAAAATCTGTCTTTATTAGATGCCAATACCAC
TTAAGGTTCTGGGCCTCTCAATGGGGAACTTATAGGTGGACTAAGGCCCAGTAAAAGCAA
>Repeat_33
GCAGACGCAGTTGACCGAGCACAACCACTAGCAAAGGCAGAACCAAGGGTAGCAATAGATTATGTGGT
TTATTATGCCCTTGATTAAAGATCCGGCAGAGCCAGTAGATCATCAGTTGGTTCAGTAGATTGAGTAG
ATTAAGTAGAAGATTCAGTAGAAGAAGCAACTATTAAATTATGGCGGCGGCGGGAGCAAAGACTAGAG

AAGCAGCAGTCGATTAGACAGTTGACTCAGTCGATCCAGTACCTGCAACCTCACTTCCGTAGAGCAGG
CCGTACCTTTAAATAAGAGGGCAAGAGCTTCTAGGAATGACTGTTTCTAGCCCCTCGGTAGCCAAGCG
TAGTTCCCAAAGCAAGCTATGATCGGCTGACAATGATTGAAC
>Repeat_34
GACGCAGTTGACCGAGCACAACCACTAGCAAAGGCAGAACCAAGGGTAGCAATAGATTATGTGGTTTA
TTATGCCCTTGATTAAAGATCCGGCAGAGCCAGTAGATCATCAGTTGGTTCAGTAGATTGAGTAGATT
AAGTAGAAGATTCAGTAGAAGAAGCAACTATTAAATTATGGCGGCGGCGGGAGCAAAGACTAGAGAAG
CAGCAGTCGATTAGACAGTTGACTCAGTCGATCCAGTACCTGCAACCTCACTTCCGTAGAGCAGGCCG
TACCTTTAAATAAGAGGGCAAGAGCTTCTAGGAATGACTGTTTCTAGCCCCTCGGTAGCCAAGCGTAG
TTCCCAAAGCAAGCTATGATCGGCTGACAATGATTGAAC
>Repeat_35
CAACACAAGGAGGAACAGGCCTAGCAACAGAACGACCAGCAGAATTCCCGTCAGCACCAATTGAACCA
GGAACAGTTACAACAGCAGTTTCATGAGCAGTTAGAGCAGCACTACGACCAGCAGGACCAGTTTAAGC
ATACGTTGAAGCATATCCAATAGCATAAGTGGATCTATCAGTTTCAGCACCACCATTAGTTTATCTAG
TAACATACCCGAAACTAAAAACACATAACTTTGGCCATATCCATACCTAGTAGCAGCATCAGCAGCAC
GAGTAGCTTACTTATAAAATTCCAATTTTTCCTCATTTTAAATATTTAAAA
>Repeat_36
AAGGCACCCCAGAATAGTCAGTCGTTACCAGGAATTTATTGAAACACTTAAATGCTTCGCTCCTCGAT
CGGGAAATAACATACTATGATGTCGGTATCCCAATCAGGCATAGGGACGCTACCTACCTACTAGCCGC
CAGGCATACGAGGCTAGGCACTACTGCCATGAAGCATATTACTTACGTGCATGCCCACATCATCTGGC
CCAACAACTACAGATACAGGCCCAACAACTCCTTATTCGAGCGGCACCGGGGATCTAAGCTCCCATTG
TGATGCTTCTTGCTCCCATTGCTTAAAAGCCCCCTATTGTCCAC
>Repeat_37
CCACCTGTTCTCTTTCCTTTCTATGATATTATGCTTGTATCGAAGCCTTCGCTTCCCTTCGGCCCCTC
ACCAAATGAGTAACTGATGCCTTGGGCACCTATTCAGTCTCCTTATGAGACAGACGCTCAGTCTCCTT
ATGAGACTGACGCCTGGATCAGAAGCAATATAGCGGCTTCCGGACACTCATTCTAGGAAGTCAGAGGA
GATCGCCCTCTCTTATAAACCTGGTCCTCATTCTCTCTCTCAAGGATCTCAGAGGTCTCTTTCGAAGC
ATGTATATTATATATGTGATTATGAGAGCAATCAGAGGTCC
>Repeat_38
GCTCCCCATGATGCGATTGCTGGATGGCATTGCTGGAATGGATGGGTGGGTGGCATGGCTGGAAGAAT
GAATGGGGATGGCTGCTTGGCTACTCGCTGGGGCTACTCGACTATATACAGTCAGACCTCTAATGATA
CCGACGAACCATAGACCTATATAAGTCGTCCCAACAGAGCCAGTCGAAGAAGCAGCCAAGCACACGTA
TAGGAAGCAATTGATCCATACGTAGCATATCCTATTGAAGCAGCGGTTGTTTATGGAGCTGTTGATTT
TACAGA
>Repeat_39
CGTCTAATAGTTGTGGGTGAGAGACGAGTAGCCGGGCCAAGAGATATATGGCTAGCAGAATGCTGAGG
TACCGCTGATGCAGGGCTTTATCGGATACTTATTGGGTGGGGGGGAAGCCCTGACCCACGTTGAGGTA
GTCAATATGTTGAGGCTCTCATAAGGTATCTAGGTCGCCTCAACTCTCTCTAGTTTAAGTACTGAGCT
CTATACACTTGAGGGATTTCCCTTGGGGTGTAATAGAAATGGTCAGGGCTCCTCAGA
>Repeat_40
TCGGTAAATATGCACTTGTATATCATTATACGATGCATAGTGGCAAAGGAAGCACTGAAGGGTGCTAC
TACGGACGTGCCTAAGGAAGGCTTCTAAGGAGGCTACTAAGGTCTTACCCTCTTAGGTGGTGGATCAC
AATCAGAACGGAACTGGATCTCGTTCACTAGGTTCTGCTTCAATGGCTACTGCTGGTTCTGCTGCTCT
TGGAGCTGGATCGACTGTATCAATGGCTACTGGTA
>Repeat_41
ACCATGTCTCCCGATCTCAGTACATATGGCGTAAGACTCACATATCGAGGTCGTTCTGGGATCGGGTG
TGTTTCCCGTCTTACAATGTTGCCCGCCCCAGGAGGTTCCGTGCCCTACTACTATAGCCTCTGACCTC
ATAGGAACCTCCTGACACTTAGCAGCCTTGGGTCCGCACACTAACTAGAATTTAGTACGATATCGCTT
CACACCTCGCGGTGCTTACACCT
>Repeat_42
TTAGTACGATATCGCTTCACACCTCGCGGTGCTTACACCTATCGCCTATCGAAGTGATGTTCTTTCAC
CTCGTACGAGAACTTGTCTAGAGAAGGATTTCCCGCTTAGATGCTTTCAGCAGTTCTTCCATACCGAC
TTGGCTGCCCGGCGCTGCTAGGCGTGACAACCGGTACACCATAGGTTGGCCCAACCCAGTCCTCTCG
>Repeat_43
AACCAGAAGTTTGGTTACACCGCGTTCAGTTCGAGCTGGGCGGTGGAGAGTTTGATAAGCAATAGCAC
GAGCTCTCGTTCTTTCACCATCGATCGTGCAAAAGTTGACTGACTTATCGATCAATCGTTTCTGTTCA
CCATCCAACCCCCCGGTATAGCTAAAAATTTCCGAGCAATTGTATGTGCCCGCCCTA

>Repeat_44
TGCATAGGTTCGTGCACAGATTTACGTTCAATAATACCTGGGGCTTTCGCTTCGACACGTCTGAGTAA
GTGCTCGCTTAAAGCCCCTTTACCATCAATAGGTACTCCCAACGCATCGACCACACGACCTAACATGG
CCTTTCCCACAGGAACAGACACAATAGATCCAGTGCGCTTGACGAGAGATC
>Repeat_45
CTTTTCTACGCGGTATCAATCGAGATCGCCTATCCTATCCCCGTGATCTCCCTACCACCCCACCAACT
GATACCGCAAGGAGAATTAAGATGAACCACAGCCTGTCCAGGCAAGGAAAAAGGGGATTCGATTCCTT
AAATAGTTGGCCGCAGATTACGGCTGACTTTTGTGAGCGGGCTTTTAC
>Repeat_46
TACGTATATAATGAGGGCTAGCTGGACCGACCAGAGCTTCGTTCATGCACCGACTAGAGCTTCCATGA
CTAGAGCTTCTTTCTGTTCTGGCACTGAGAAGAGCTTCATGCACTGACCGGAGCTTCTAGGCAATGAG
TATCCCAGCCAAGCCCAGCTATGACTGGCATCGACACCTG
>Repeat_47
AGGAGCAGGATCCGAAGCGGTGGCGGCTACGGCAATGGAATATTCCAAAGCATCTGCTTCCGAGATAA
TTTGAACCAATTGTGCCACGGTCGAGCGTTTCTGTCCAATCGCTACATAGACGCAATACAATTTATCA
CTATCAGCCTTTGACTTTGAGTTCATTCGCTTCTGGTTC
>Repeat_48
CTCTTTCGGTAAGCATCCGGTATCTCAGCAGATAAACATTTCTCTGAGCTTATCCTGTTGCTTATACG
TCGTTTGAAAGCTGCTCCAAGGATCCAACGAATAGCTAAGGTTTGTTGACGATCCCTGGCTACAATCT
CAGGTACATTATGAGTGGTACCTGCTACTCGTACTTT
>Repeat_49
TATAGCGCGTTTTTGATTAGCAAAACGCTTCAAGACCTAACCTAGCAGTCTCACCATTCTCCCTAGTC
TCACCACTATGTGGAGATATACAGTAAGACTGAGTCTCTATCAGTCTTTAAAGCTCAGAAGCTCTGTT
ATAAGAAATAGTCTCAAAATCACGATCCTT
>Repeat_50
TGCCAGAGAGTATTGCCAGCCGATCATAGCCAATAGTCCTAGACAGGAAGGAGTCCTTCTGTAATGAA
TCTGTATCCGGAAATATTGAATCTGCGAATCTATTTCTTTTTCTTTAAGGCGGGCTTGACCATGTCTC
CCGATCTCAGTACATATGGCG
>Repeat_51
TTTGAGGGAAAATAATAAGGCTTTCCCGAACTATTTAGGGTAAAGCCTGTGAACACTTCCTGAATTCA
CGTTTAGTGAGTGAATAAAGCAAGCAAGCGCTGTACTACTTTAAGAGTATAGCGATAAATGAAGCAAG
TGAGGTCTATCTAA
>Repeat_52
CCAGCAGCCTCATCGGAAGATTTTGTTTTAAATCCCATCCGTTGAACTCAATATGCTCGTAAGTAAAA
AAGCATGGTTCAATTAATCTGTTGGTAGGGGCTGCACAAACATTCATCAAGTAGGGTGAACCAGGTCC
TCCCTCA
>Repeat_53
GCTTAGATGCTTTCAGCAGTTCTTCCATACCGACTTGGCTGCCCGGCGCTGCTAGGCGTGACAACCGG
TACACCATAGGTTGGCCCAACCCAGTCCTCTCGGACTAGGGTTGGCTCCTCGCAGTTCTCCCTTCAAC
ACCCCAG
>Repeat_54
TTCTTTTCTACATAAAAACGCTAAATATGTCTTCTGATGAAGCCACCCTTGGGTTGGTATAAAAGGCC
ACTTAAACTGGATTTTTTTCTATATGAACTAATACTCAATTTAACCTACTCAATTCAAACCGTTCAAT
TCAAT
>Repeat_55
GCCGGGCCAAGAGATATATGGCTAGCAGAATGCTGAGGTACCGCTGATGCAGGGCTTTATCGGATACT
TATTGGGTGGGGGGGAAGCCCTGACCCACGTTGAGGTAGTCAATATGTTGAGGCTCTCATAAGGTATC
TAGGT
>Repeat_56
ACCCAATCCGTCCTATTACCAAAATCCTTACAGGCAGCCATCTATGCCTCAAGGGTATCCTAGTGCCC
CCTATGACCCAAATTGCTCGTCTTCTCATTTAGGAGCTAGTACTTCAGGATCTAGTGTACACCCTCAT
CACC
>Repeat_57
ATAGTTGGTAATTCTCCTTTCTAATAGAGTAGTGAGTTCCGCAGCTCCAGGATCTAATTTCATACTTC
AGGAACGAATGGATAGGGAGAAATCTACTTTACAAGATGATAAATAAAAGAGAATGGGGCCTGGAAAA
G
>Repeat_58

CTGGGTATCAACAAGCACTTATATTCAACGACTAACAACTATAAGCAGCACTTCCTAGAGCTTCTTCA
TTAGAGATGGTAGCTAAAGATCGAGATAGAGATCCAATGAGTATCAGAGCATCATGAACTTCCAA
>Repeat_59
AATAGTCCGTGCCAATTAGTCAATCATAGTCGAGAGCCAGTAATCCAGGTGTAGATGCCGGTACTCAA
TACTGGTGTTGATCCACCAGATGACGGTACTCTCATGTCAAGTTTCAATGCCAGGTTTCAAT
>Repeat_60
AAATAGACTATTGAGTATCAATCTGGTTCAAGAAGCGCATCACCGAATCCCCTAAAAGCCTGAGCTCC
GCGTAAGTAAGGGATTTGTTCTGACTTTCAGCAAACGTAATTATATACGTAATTA
>Repeat_61
GAGCGGGCTTTTACCATGTCTCCCGATCAATCTCAGTACATATGGCGTAAGACGATTTCACATATCGA
GGTCGTTCTGGGATCGGGTGTGTTTCCCGTCTTACAATGATGCCCGCC
>Repeat_62
CCGGATACCGGGCATTATCCTGTTTGGTTCTACCAACCAGCAAACACGGGCTCGAACCCCTACCTATG
AGGGAAGAAGATTTCGACGTTAACTTAGAGATTACCCTTCCCAACC
>Repeat_63
GGTTGGTTGGGCGGGGACTGATTCTACTCTAAATAGATGTGCGTCTGGTAGCTCATCGTCGATACCTA
TGGCTTCTTCTCCTGTTTCCACCCTAGAGAGGTTATCTGGGCC
>Repeat_64
TTAGATGCCAATACCACTTAAGGTTCTGGGCCTCTCAATGGGGAACTTATAGGTGGACTAAGGCCCAG
TAAAAGCAAGGGCATCGTCTAATAGTTGTGGGTGAG
>Repeat_65
CTGGATAAGCTGCAGGATCAACGACTGGATCGCTGATAAAACTGGGGCCTACTTTCAACGCTTCTCCT
GCGACTGCCTTAGCTGCAGGGCAAGGAGTTGTATGT
>Repeat_66
AGCCCACCAGTAAAAGAAAAGAACCATGGGCCGGCCCACCAAGATGCTGATAACACATCGCTAAATAT
ACGTCGATTCGCAATTTGAGGAACATAGTCCTTTA
>Repeat_67
AGCCTTAACCTGTGTCTGGGGAATAGAACTGAGCTCTATACAAGGAATTTATCTTTGGGTGGAATAGA
GATAGGGCACATGAAGAGGGCAAAGGCAAGGTTT
>Repeat_68
GCTTAGATGCTTTCAGCAGTTCTTCCATACCGACTTGGCTGCCCGGCGCTGCTAGGCGTGACAACCGG
TACACCATAGGTTGGCCCAACCCAGTCCTCTCG
>Repeat_69
ATATGATATCAATAGCTATAGCGGTTTTTCCAGTTTGTCGGTCCCCGATTATCAGTTCTCGTTGACCA
CGGCCTATAGGGACCAGGCTATCCACCGCTTT
>Repeat_70
TAGTTGGAGAATAAAATAGTATCCTGCCCTTCTTTAGAAGGGAAAGGTCTTGAGCTCAGGTAAGGTCC
TCGCCGGTCAACCCAGCCAGTAAGGTCCTG
>Repeat_71
AGAAAGCCTTATTTGTTAAACAAATCCTTCGTATATCGCTCGTTTAGCCCTCGGCTTACACAACCACC
CTTGCCGTAAGCCTTAAGAAGCCATATAG
>Repeat_72
ATGGATCTCGCTCACGAGGGCTTGGATCACGCTGTAGATCATCGGTAGGAACTAGGTCTCGATCACGA
TCTGGATCTTCATCTGGAGCGGAACTG
>Repeat_73
TGGGAAAGGCAGGAGCACAGCCAGTAGCAGCATCATGGACAAAGGAAGCATCTAACATAGAGGAATTT
ACCGTGAACTAGGTTCATGCAGAGG
>Repeat_74
TTTATTAGATGCCAATACCACTTAAGGTTCTGGGCCTCTCAATGGGGAACTTATAGGTGGACTAAGGC
CCAGTAAAAGCAAGGGCATCACCC
>Repeat_75
ATAGCGTTAGCGTCCGGGTCGTTGTGCCCCCCACCCAATAAGTAGGGACAGGGGGGAAGTAGGCTAGA
TGCGACCGATAGGGAGCCCTCCC
>Repeat_76
AGGAATCAGCCTTCAGCCTTGAGCCTCACCTTAAGAAGCCCTCATCACCAGATTAACCAAGTCAACTA
GTCCAACAAGTGAAATAGAA
>Repeat_77

TTTATTAGATGCCAATACCACTTAAGGTTCTGGGCCTCTCAATGGGGAACTTATAGGTGGACTAAGGC
CCAGTAAAAGCAAGGGCATC
>Repeat_78
ACCATTTCCCCGGCTTGAATCTCGTTCAATCCATAGACACGTGCAATACCATCTCCAACTGAGACCAC
TCGACCGATCTCATCAACTT
>Repeat_79
TTGTCCAATATTCCCCACTGCTGCCCCCCGAGGGAGTCTGGGCCGTGTCTCAGTCCCAGTGTGGCTGA
TCATCCGAAAAGACCAG
>Repeat_80
ACGAGCGATATACGAAAGATTTGTTTAACAACTCCAAAAACCAAGACCTAAGCCTGTAGGTTCATACG
CCAGTCTTACATAAGC
>Repeat_81
TTAGATGCCAATACCACTTAAGGTTCTGGGCCTCTCAATGGGGAACTTATAGGTGGACTAAGGCCCAG
TAAAAGCAAGGGCATC
>Repeat_82
GTTGGGAAGGCTAGATAAAGAGACTTACTATCTGTCTCTCTAAAGCTATTACCCACGAGGTAGGAGGT
CTGCTTGGGAGGGC
>Repeat_83
GATCGATGGGTATACGACTGAAGGTACCACGTCCATGGAGCCTAGCTCTATGGGCGGGGGTGGAGGAA
CCAGGAAGAGAAG
>Repeat_84
TTTATTAGATGCCAATACCACTTAAGGTTCTGGGCCTCTCAATGGGGAACTTATAGGTGGACTAAGGC
CCAGTAAAAGCAA
>Repeat_85
ATAAGATAAGTGGTTATCTTAAATAAGCGTTCTATGCTACATAAGAGCATTCCAGGAAGAGCTACTTT
ATCAGCAGTCTAT
>Repeat_86
CCCACCTAGGGGGGACAAGAGACGGCCATCTCGAAGCACATTGCGACCTACAGGCAGCACCGGCGAGA
CCCAACACAAGGG
>Repeat_87
TTTACTAGCCAGTCTATAGCGTTCGGTCACACAAGCTAGCAGAATGCTGAGCGCGAGGGCCGGATACT
TATTGGGTGGGG
>Repeat_88
CGTATGAACCTACAGGCTTAGGTCTTGGTTTTTGGAGTTGTTAAACAAATCTTTCGTATATCGCTCGT
TTACGCAATAGA
>Repeat_89
TCTGACTTGCCTTGAACAGATGAGCGTGTTTCTGACCCTTGACCCCCAAAGGGAGAGGGGAGCCTAAA
AGGGTTACGGC
>Repeat_90
TTTTATTCCCCCCTCTACGAGGTTCCTTATTGTGCCAGTTCCCAATCCCCCCGTTCTTGGTACCGCAA
CCCGCCCGGTA
>Repeat_91
CCAGTTTACCCATAAGCGAATGACACCCGTCTCTTGGCAGTGAGACCTTCTATTTAAGACAACTGGCT
ATCGGCCGGC
>Repeat_92
TCAACCCGGTCAAGTCCTGACCCCCTTCCTTATATAACAGATATTCTCTCAGTTTCTTGTGTGAAGAG
TTGGCTTTT
>Repeat_93
TTAGATGCCAATACCACTTAAGGTTCTGGGCCTCTCAATGGGGAACTTATAGGTGGACTAAGGCCCAG
TAAAAGCAA
>Repeat_94
TATGAATGGCAGGCATCCCTGCCAGAGAGTATTGCCAGCCGATCATAGCCAATAGTCCTAGACAGGAA
GGAGTCCTT
>Repeat_95
CCGAGCCAATATCTCCGATCCTGGGAGGAGCCGTATGAGGCGGAAGCTTCACGTACGGTTCTGAAGCC
GAGCCTTT
>Repeat_96

AGCGGGAAGCTGGTACTCTCAGGTTCATTACTTCGATAACAAGCAAGCGGGGAACAGTCCGGTATGCA
TAATAAAT
>Repeat_97
AAAGGTTTATTATATTCTACTGTTCGTTTCGGTATCTCTGTGGACAAGGCTGCTTTGGCAATTTATAG
TCTTAAT
>Repeat_98
GTTGTTGATACCGGCAAGACCCAGCCAGATGATATATCGTCTGCTGGTTGGTAGTGGGAGGACTCTTA
GTACCCG
>Repeat_99
CCATTCAGTCCATTCCTTAAATAGTTGTTCGTCAGGGTTCCACGCTCACTTACTAGTGCTTTCCTCCT
GCTTTC
>Repeat_100
CTGGAAATGGATATGCTGGGCGAGATGCTGCGGGTGCTTCTGTGACTGGGTCTTGCTTTCTATAGGAT
ATGGAT
>Repeat_101
AATGCGCATGATGGAGAGATAAATGTGGATGCATCTATTTCTCCCAGTTGGATTGAGATCTGTCTGTG
GATGC
>Repeat_102
AGAGAATAGTGCGCCTTTCCCCACCTTAGAAAGAGACTGAACCAGACCTTCCCTAAAGGACTAGATAA
GGTG
>Repeat_103
TAGTTGTGGGTGAGGCCGGGCCAAGAGATATATGGCTAGCAGAATGCTGAGGTACCGCTGATGCAGGG
CTT
>Repeat_104
TAATGGAGATCACATTGGTAGGGATATAGGCCGACACGTCTCCAGCTTGTGTTTCAATGACGGGTAAC
GCG
>Repeat_105
ACCTATTACGCAAAACGATCAACGCAAGCTAACTTAGCTTAGCCCTACGAATATGGGCAGGTGCAGAA
TGA
>Repeat_106
TCAGATTTGAGTGGATCCGCTGCTATGGAACAACACACGACCAAGGAACAACAGGGAGGGAGGAGCAG
GGC
>Repeat_107
CTATCTATACCTCCTGGGGGCCAACAGATACAGATGAAGCAGAAGTTTCCGTCTACGTCACTCCATCT
TT
>Repeat_108
GGATTCGCTCCGCCTTACGGCCTTGCTTCCCATTGTAATTGCCATTGTAGCACGTGTGTGGCCCAGCC
C
>Repeat_109
GATTTTGAAATAGAATCCCTGGGAACTAAGTAATACCCTTACTCTACCAGCTGAGTAAGCCCTTTCTT
T
>Repeat_110
GGGGAGAGGGCCTGGTCGACAATGGGGTGCGGCGAATAACGGGATTCGAACCCGTGTTTTCGAATTCA
C
>Repeat_111
TTGCATTCTCCTAGAGCTGGCGTCAACGAGCTCATGCTAATGCTTGCTAGGACCTGTCGCAAATGTTT
>Repeat_112
GGTCTATCCACAGGTTCTAGCATCTCACGGGTCTAGCCACACGTTCGTTCTAGCGTAACGTTCTAGCC
>Repeat_113
CCTCAACTCTCTCTAGTTTAAGTACTGAGCTCTATACACTTGAGGAATTTCCCTTGGGGTGTAATAGA
>Repeat_114
CGTATGAACCTACAGGCTTAGGTCTTGGTTTTTGGAGTTGTTAAACAAATCTTTCGTATATCGCTCGT
>Repeat_115
TTTACCACCATCTGAAATGCACGAAACTTCGATTGGTGGGAGCCGGTTCATGAGGATCATCCCTTTT
>Repeat_116
GTAGATAGGAACCGAACTGTCTCACGACGTTCTAAACCCAACTCACGTACCACTTTCATCGGCGAAC
>Repeat_117

AGGAAGGGAGGAAGATGGGGCAACTTGAGGAGCGATAGTAAGGGTCCAGGAGGGCAGTGGAGTGGAG
>Repeat_118
AATGAAAGCATGCTCCGAGTATCGGTCTGACAGCAAACGAAGAGTCATTATATACGTAATTACACCT
>Repeat_119
AGCCGACGTTAATAGCAGGTCTAATTCCGCGATAGAATAGCTCTGTTTCCAGACAGATCTGTCCATC
>Repeat_120
TTCTTCCAGTCATTCTTTATATATGTAATAACACTAGATTCGTGATTTTATGCGATCCGAAAGCGT
>Repeat_121
ACCTACCTTTCCTTTCAGGACCTTTCCCGTGGAAAAGGGAAGGAGATCACTTAGAGCTAGAAGGT
>Repeat_122
CACCCTTGCCGTAAGCCTTAAGAAGCCATATAGCGCATTTTTGATTAGCAAAACGACAGAAAGCT
>Repeat_123
GCCCTTTTTCCCTAGACCTCGGTTTCCTATTATTATTTCTGTTACCTATCCCGTTACCTATCCCG
>Repeat_124
ACCGAACCCTTGGGACCTTCTTCAACCCCAGGATGTGATGAGTCGACATCGAGGTGCCAAACGAC
>Repeat_125
CGGGGCGTAGCGTCGCATGTTCGCATGATGATGGATAGCAGGATAGCTATTGATATTCCGACATA
>Repeat_126
CGATTGGGAGTCAGAGGATCCATAGCACCTGCCTACCTCGATTTGGAAAGGACCTCGTCTTTCTT
>Repeat_127
GGCCAACTCCCTATCATGCATGCAACTGTTTATGCTCATCCCGCCAATCTACATCATGCAATAT
>Repeat_128
AGACGCCCCATTGTTCGGGGTGTTGCTATGAATCCGGTGGATCATCCCCATGGAGGAGGTGAAG
>Repeat_129
GGTGGGGGGCAAGGGACGCTAACGCTAACAGAAAGGACGCTAACGCGATATGCTCCTTAAAGAG
>Repeat_130
ATGATTGCGGAAGATCCCCATATAGAGTATGATGTATCCCCAGAAGATTGGCCCTCTATAAGA
>Repeat_131
CCCGGCTAAATGCCTTAGTCCTAATAGATATAGGAACAGACTTCCTACTCGGCGTACGCTATT
>Repeat_132
AAAGAAAGAAGGCAAGTATAGGAGTTCAATAAATTCTTAAATAGCGTAATTACATATATAAT
>Repeat_133
TATCTATCCATCTAATTAATTTCATTCTATCTCTGAGCATAATAGCTCTTCAGAGCATACCT
>Repeat_134
TAAGGTCCTGAAAGAAAGGTGTAATTACGTATATAATGACTCTTCGTTTGCTGTCAGACCGA
>Repeat_135
ACAGGAACAAGTGTAGTTGAATGGGGCAGCAGGCGGGTTACACGTTCCACTGTGCCGAGATG
>Repeat_136
ACCCTCCTCCACCCACCATAGTGTGAGAAACAGAGCCAGGCTTACTACAACCTGCCTGACTG
>Repeat_137
ATAATATCGTATACAAAGATATATCTCATTATATATGTAATTATGCTTGCCTTATGACTTGA
>Repeat_138
ACGCTTTAGGCTCTCATAATCGATCGTACCCTAATAGAATAATATCGTATACAAAGATATAT
>Repeat_139
ATGGCCTTAGAATCGGTTTTGACCTGCCATTGCCCCGAGCTCCCCATGATGCGATTGCTGG
>Repeat_140
AGCCTCAGTTCCCGGATCACCATTTGAACCAGTCTTATTCCCAGCGTCAACAGTTGAACCA
>Repeat_141
CCTTTTGCCAAGTACACTAAGTCCCGCAAATGCCAGCCTTGAACCTGTTGAAAGAAGCCTT
>Repeat_142
AAGGAGTTGTATGTGCCCAATAGTCCGTGCCAATTAGTCAATCATAGTCGAGAGCCAGTA
>Repeat_143
CCCGACCATCAGGAGGGCGAGGGCCTTTAATTCAATATCTTCCCGAACTCTAACTCAGGG
>Repeat_144
GACGAAAGATAGCACGAGTAATAGACTTATACAGACCATCCCTGCTGTGCGTCCCTCCTC
>Repeat_145
GCAGAAAGCCTTATTTGTTAAACAAATCCTTCGTATATCGCTCGTTTAGCCCTCGGCTTA

>Repeat_146
TTTTTTCCCTCTCAGGCTGGATGGGTGGAGGCGCACCATGGGACGGGGTTGGTGTTGGA
>Repeat_147
AACTAGAAGACCTATCGTAAAGGGCTACCCTTTCCGGGGCATCCCTTTACTAGCCAGTC
>Repeat_148
GTCATTCTTTATATATGTAATTACGCTTTAGGCTCGAATCATCGATCGTACCCTAATAG
>Repeat_149
ATATGGGTGAGGGAGAGGATGCGCAAGCAGGCTCACCCGTCGTGGGTGAGGAAATAAGT
>Repeat_150
AAAGCGTAATTACGTATATAATGAGGGAAGGTGCTGGTTAGAGTGATGCTGCTTACTTG
>Repeat_151
AGAAAGCCTTATTTGTTAAACAAATCCTTCGTATATCGCTCGTTTAGCCCTCGGCTTA
>Repeat_152
TCTCTTCTCTATAAACAGCCCGGATGCTGAGAGCGTTCACTCCCTAATATGAAAGCGT
>Repeat_153
TGCCAGAGAGTATTGCCAGCCGATCATAGCCAATAGTCCTAGACAGGAAGGAGTCCTT
>Repeat_154
GGGATGACATTTCAGATCTTCGTGCCGGGGGCTATTCCTTCGCGCTTCGATGGGAACA
>Repeat_155
AAGCCATATAGTGCGTTTTTGATTAGCAAAACGACAGAAAGAAAGCTATAGTCATCTA
>Repeat_156
CCTAAGTAGAATAGAAAACTTGTTTGTAAAAAGCGATAGCCTTTCGCGGCATGGCCA
>Repeat_157
TACCTTATTCCACCCCAAGAGGAAATTCCTCAAGTATATAGAGCTCAGTTCTACTAT
>Repeat_158
CTTGAAACGAAGTCTTTCACGAACGTTTAGTTAGGGTTCTTCACAACGCGTACCTCG
>Repeat_159
AAGGAAAGATAAGATCGAGTAAGTCGAGTAAGCCGCCCAGAACCAAAAAGTGGGTGG
>Repeat_160
GCCGGGCCAAGAGATATATGGCTAGCAGAATGCTGAGGTACCGCTGATGCAGGGCTT
>Repeat_161
CTTCTTCCCAATGAATGCGATAAGTGTTCGCTTCTTCCCAATGAATGCGATAAGTG
>Repeat_162
CGATCTTCCAGTCATTATATATGTAATTACGCTTTAGGCTCTCATAATCGATCGTA
>Repeat_163
AGTGAGACCGCGATCCACAAACGAAGCATGGGACCCACCCTTACTCGAGAACGGA
>Repeat_164
TTTTCTAAATTGGGGGGCGGCAACAGCTAGGAGTAGAGGGAGTAGGGATGGCTAG
>Repeat_165
TTAGGACTAAGGCATTTAGCCGGGTCAATTAGCGAGGGAAATACCTCTATTTGCC
>Repeat_166
ATAGGTACAAGGGATCTCTGCGTTGGGCTAGCGTTTGTCCAATGCAGCAATTTT
>Repeat_167
GCTTGCCTTCTGAGTATCAATCTGGCCGAATGAAAGCATGCTCCGAGTATCGGT
>Repeat_168
AGTTGAAGGAGCAAAATTCCCAGTATAAGCAGAACCAGTCCGAGCATCATAATT
>Repeat_169
AATACCTTCCTTCGCTATATATCTTCCCGAACTCGAAGGATCACTCTTTCCGTT
>Repeat_170
AAGGGCCCGCCTCCACTCCCTTCCCCCCCCGGAGGGATAGGCAGGCCTATCTAT
>Repeat_171
TGCCGAGAGAGATACAGAAGTGCTTGTTTATAGCCGCTAGTACTTGCCGGGAGT
>Repeat_172
GCCTCGCGAGCCTCCTGATGCTTTTAGAGATGAGGCCTTTCGGTCGGCGAAGC
>Repeat_173
CAGTCAGGACCGTCTGCCCTCTAGAGCCTTATCTTCGAAGTACTTATTTCCTC
>Repeat_174

GTGACTCTTTCTTCAAAAGTCCATTTCTTCCAGTCATTCTTTATATATGTAAT
>Repeat_175
TATCGGATTGCATAATATCGTAATATGCTTTCTCGTAACCCGCCCGAAAACC
>Repeat_176
CGAAATAGGCCATGGACTTGGATCTACCAAGTGATAAGAATGCCTCTGAGAT
>Repeat_177
TCCAGTCATTCTTTATATATGTAATAACACTAGATTCGGTGTGAGTCGAAGG
>Repeat_178
TGCTACCCCAAAGGAACCATTATCGGGAATCTGAAGGCGTTCTGGAGCCCTT
>Repeat_179
CCTTCCCCGGCTTGCAAGACGTTACGCTTTCTATTTCTTCTTTTTTTCTATT
>Repeat_180
GACCTAAACTGATCGACCTATTCTACCAGTTCAGGAAAGGTATAACCTTGC
>Repeat_181
GAGGTACGTTAAGTGACTCACCCGTTATGGGTGAGGTACGTTAAGTGACTC
>Repeat_182
CCTTCGACTCACACCGAAAAGCGTGTTATTACATATATAAAGAATGACTGG
>Repeat_183
GCACCAAGATCGAAACGAAAACCTCCGCTGCTTAGTATTTGCCCAATCCGC
>Repeat_184
AAACACAGTGGAATCACGCTTAGCGAACGCTCGCTGCTGGCTGGTGTGCCT
>Repeat_185
AGGCGTAGCATATTGAAAGCTAGGCGTAACATAGGTATATTGAAAGCTAGG
>Repeat_186
ATCTGGTTCAAGAAGCGCATCACCGAATCCCCTAAAAGCCTGAGCTCCGCG
>Repeat_187
GCTCTACTCATTGGAAGCTATGCTCAGGGCATGAAGCTCTACTCATTGGA
>Repeat_188
AGGATGCGCTAGCAGGCTCGACCAAAGGGAGAGGATGCGCTAGCAGGCTC
>Repeat_189
TGACTCGCTTACACTCAAACATGTATCTCATTATATATGTAATTACGCTA
>Repeat_190
TCCCTCCACACCTTTACTTACCTGCCTGGAAGGGCCTTACCTGAGCGAGT
>Repeat_191
TTTTGGAACGAAGTCGCTCACTTGAAGCATGAAGGTGAGGGGCCCCTACC
>Repeat_192
TCACATATCGAGGTCGTTCTGGGATCGGGTGTGTTTCCCGTCTTACAATG
>Repeat_193
TTCCTCTCCCCTTGGTCGAGCCATTCTTCCTCGATTTTCTATGCTCTCC
>Repeat_194
AGGCCCCTCACCAAATAAGTAACTTAGGAACGAAGTTGATTTATTGTGG
>Repeat_195
AGTGCTGTACCACGCTAACTCAGCCACTAACGACTGCCCTAGTTCGCGC
>Repeat_196
GAGAGCCGTGTGATGGGTTACTATCCAGCACGGTTCGGAGAGCACTTGT
>Repeat_197
GAAATTCCTCAAGTATATAGAGCTCAGTTCTACTATTTATGATTACCTT
>Repeat_198
CGTTCAATTCAATTTGAGACGTGCCTTGATAAACTCCCTAAAGTCAAGC
>Repeat_199
TTCGAAGAGCTAAAACCACGGAACAGTTAAGGAGCCTTGTTAGAAGAGG
>Repeat_200
GCCTGGTGGTCGGCGTAGCAATAATGACATTTGTCGATATGCCACCGCC
>Repeat_201
TTATTTGTTAAACAAATCCTTCGTATATCGCTCGTTTAGCCCTCGGCTT
>Repeat_202
GGAATTTGCATTCATATTATATATGTGATTATGCAAGGCTCCTGCCCC

>Repeat_203
TACGATATTATGGTACGATCGATTATGAGAGCCTAAAGCGTAATTACG
>Repeat_204
AAAGTCAATAGAACAGTTAGCTAAGTGCCGTACCCACTAATATTCCTT
>Repeat_205
GGCTTCCTCCTTACACTAACTAGCGCCTCCCGTTCCAGCACCACTGCT
>Repeat_206
CCTTCCTCGCCCATAACGGGCGAGTCCGCTTAATGCTTCCTCACCCTC
>Repeat_207
TAAGGCTCTAGAGGGCAGACGGTCCTGACTGGGCCTTAACCTAAAGTC
>Repeat_208
ATGTAATTACGCTTTAGGCTCTCATAATCGATCGTACCCTAATAGAAT
>Repeat_209
AAACTGGATTTTCTTCTCTATGAACCCTTTCATCAATGTTCTTTCTCT
>Repeat_210
CTTATTTGTTAAACAAATCCTTCGTATATCGCTCGTTTAGCCCTCGGC
>Repeat_211
TTGATGGAGCATGTTCAAAATATGGATCCGGAGCAGGAGTTGTCTTT
>Repeat_212
AGATAGAAGCGATAGCTCCGTGGAGCTTTCTCTGCGGTCCTGTTTGT
>Repeat_213
GGGATCTTCCGCAATCATCCTTCGAAGAACACGATCCCAGCCTGCAA
>Repeat_214
TTATTTGTTAAACAAATCCTTCGTATATCGCTCGTTTAGCCCTCGGC
>Repeat_215
AGCAATCCTAGCGAGTCCAGCCGGACATCTCGCCTATCTGTGTGAAA
>Repeat_216
TGGCCCAACAAGGAAGGTGGGTGTTAGTGGTTCATCATGGTCAATGA
>Repeat_217
AGGTTCAGATTCGAAATGATTACTGCTTGCAGCATGGGAGCTGATTA
>Repeat_218
TGACACATAGAATATGGCGACGACAACAGCATGTCGTAGAAGGAGAT
>Repeat_219
AGCATATAGCTAGCGTTCGTTCTGAGCCAGGATCAAACTCTTTTTTT
>Repeat_220
TTACCTGTTTTACACCTGATTCCTTGGGTGCCAGGCCTTTCTTCTCT
>Repeat_221
TTGCTGGTTAGCTATCGTTTGCTTGCTGACTAGACGTTTGGATAGAC
>Repeat_222
ATTTCGGGCCACCAAACCCTGCAACCGGAAAAGCGAACGTACGTTGT
>Repeat_223
AGAAGCTTGCCAGAGATCAGCTTAGAAGCTTGCCAGAGATCAGCTT
>Repeat_224
AACTTAAATGGGTTCATCGGCCAACTATTTAAGGAATCGAATCCCC
>Repeat_225
GAATTTGTGTAGGTTCTGTACTTACCTTCGTTATAAGGTAAGGGAT
>Repeat_226
GGCGGTCCAACGGACGAATGAGACAGCCCACATCTGTAGAGATAGG
>Repeat_227
ACATATTCACCGCGGCATGCTGATCCGCGATTACTAGCGATTCCAA
>Repeat_228
TCGGTCTGACAGCAAACGAAGAGTCATTATATACGTAATTACACCT
>Repeat_229
ACATCTCTCATCTGGTGGGAACCCAGTTTACCCATAAGCGAATGAC
>Repeat_230
ATCGATCGTACCCTAATAGAATAATATCGTATACAAAGATATATCT
>Repeat_231

ACCTTTTCTCTATATGCTTATGGGTAAACTGGCCAAAGAGAAATTC
>Repeat_232
TTTAGCCTATATGAGGCAAGTGCGAAAGCGTAATTACGTATATAAT
>Repeat_233
AAAAACGCGCTATACTATATGGCTTCTTAAGGCTTACGGCTTAGGT
>Repeat_234
TTAAGGAGCATATCGCGTTAGCGTCCTTTCTGTTAGCGTTAGCGTC
>Repeat_235
AAAGCGTAATTACATATATAATAGCAGCATTGTCGAAGGATTGAC
>Repeat_236
TATTTTGGAACGAAGTCGCTCACTTGAAGCATGAAGGTGAGGGGC
>Repeat_237
GAGGAACGAACGCGAGACCAAAGGGAGAGGCCCTTGAGGGACCTT
>Repeat_238
TAGTATAGCACAGAATTTGTGAATTACGGCTCACCTCACTATAGC
>Repeat_239
TAAGGCGTAGCATATTGAAAGCTAGGCGTAACATAGGTATATTGA
>Repeat_240
CCTCAACTCTCTCTAGTTTAAGTACTGAGCTCTATACACTTGAGG
>Repeat_241
ACTTACTAAGGGTAAGGGATCTATTCTCTGCAACCGTGCAGATTT
>Repeat_242
GGTTTTCTCTCAGGTTTTCGGGCGGGTTACGAGAAAGCATATTA
>Repeat_243
GAAGCCCTTCGAACCAATAGCTGGCTGGTCTTGACTTCTATCCC
>Repeat_244
GCAGCCCCAGCAGCGAAAGGAGATAATTCTTCCCGGCAGCAGCA
>Repeat_245
ATCGATCGTACCCTAATAGAATAATATCGTATACAAAGATATAT
>Repeat_246
TAGGCGTGACAACCGGTACACCATAGGTTGGCCCAACCCAGTCC
>Repeat_247
AGGCGTAGCATATTGAAAGCTAGGCGTAACGTATTGAAAGCCA
>Repeat_248
TCTAACGCGAAGTCAACCCTACAGATATGATCCTTAGAAACCC
>Repeat_249
GGGTTGGTGTTGGACCTTATTATTATTAAAGGCGGGGGTGCGA
>Repeat_250
CCCACGAACGAAAATGAGAGTTCTTGAAGTTAAGATGGATGAA
>Repeat_251
TCGACTAAGACTCCTGGTAGCAGCTATTGGTGACCGGTTGGGA
>Repeat_252
GGCACTATGGATCGATCTACGTTGCCGTAACAAATTCACAAAT
>Repeat_253
AAAGTCCATTTATTCCAGTCATTCTTTATATATGTAATAACAC
>Repeat_254
TCTGCGCGTTGTATCGAATTAAACCACATGCTCCACCGCTTGT
>Repeat_255
GCAGCAGCAAAAGTGGTCCCAGCAGCATCTCCATAAGCAGTTT
>Repeat_256
CGTAACATAGGTATATTGAAAGCTAGGAGTAACTAACATATTA
>Repeat_257
AGGCGTAGCATATTGAAAGCTAGGCGTAACATAGGTATATTGA
>Repeat_258
TTTTGGAACGAAGTCGCTCACTTGAAGCATGAAGGTGAGGGGC
>Repeat_259
TTACCCCCTTTTCTCCCTTTCCTAGCTTGATTGCTTACGCTCG

>Repeat_260
AATAGTAGAACTGAGCTCTATATACTTGAGGAATTTCTCTTTG
>Repeat_261
AAGGCTTATAGAGGAGTATAAGGCTTATAGTTCATAAACGCTA
>Repeat_262
GTGCCCCCCACCCAATAAGTAGGGACAGGGGGGGAAGTGATGA
>Repeat_263
TAGAGCTTCTTTCTGTTCTGGCACTGAGAAGAGCTTCATGCAC
>Repeat_264
AATGGCAGACTGGCGGCGAATGGCAGCCGACGTGGCAGCCGA
>Repeat_265
AACATTAACGCATTTAGTTATATACGATCCACCCTTATGACA
>Repeat_266
ATAGTCCCTCCCCTTCCTACCTATCTAGATTGAGTTCAAGGC
>Repeat_267
GTAAGGTAGGTCCTGCCCACCCAGTAAGGTCCTGATCAACCC
>Repeat_268
GCTATAGGATAACCTTCTAGGCCTTCCTTAGCACATGAGGCT
>Repeat_269
CAGGCCCCCGTCAATTCCTTTGAGTTTCAGTCTTGCGACCGT
>Repeat_270
AATAGTCCGTGCCAATTAGTCAATCATAGTCGAGAGCCAGTA
>Repeat_271
TATGACTCGCACTCAAACATCTCATTATATATGTGATTATGC
>Repeat_272
TCGGCTGGCAATACTCTCTGGCAGGGATGCCTGCCATTCATA
>Repeat_273
GGTTGATCAGGACCTTACTGGGTTGATCAGGACCTTACTGG
>Repeat_274
TAAACTAACCTAACCTATCTATCTCTAAATCACCTATATTG
>Repeat_275
CCTCGCCCTTCTTAGGTCGGGCGAGTCACTTAACGTACCTC
>Repeat_276
ACATAGGCCGACTGCTGAGCCCACTCTGGCTCAGCCGCACC
>Repeat_277
CGTTCAATTCAAACCGTTCAATTCAAACCGTTCAATTCAAA
>Repeat_278
CTGGCGGCTACCTATGCCCCAAAAAACGGCTAGGGACTTAA
>Repeat_279
GAGGTACGTTAAGTGACTCGCCCGACCTAAGAAGGGGGAGG
>Repeat_280
TACTTGCCTACAAGGAAGGTATTCGGTCAGCTAAGGTCTTC
>Repeat_281
GGGTCGTTGTGCCCCCCACCCAATAAGTAGGGACAGGGGGG
>Repeat_282
TTTTTGATTAGCAAAACGACAGAAAGAAAGCTATAGTCATC
>Repeat_283
AAGACTAGAGAAGCAGCAGTCGATTAGACAGTTGACTCAGT
>Repeat_284
GGCGGCTGCTCGCTCTTTGATCGGTGATTCACAAGCCACT
>Repeat_285
CATTAACTCTGAACCGGCCACCCTTTTTATTCCCATCCAT
>Repeat_286
GAGGGATAACCAACCCTCCCTTCTAGCTCTTTGAGGGGAA
>Repeat_287
ACCTTACCTTTCCTTGTTCGCACAGCGAATGAAGGCCGGG
>Repeat_288

TCATTATATATGTAATTATGCTTGCCTTATGACTTGACAC
>Repeat_289
CCATCAACACAGTTGGCTTTACCCATGGTTTAGATATTAA
>Repeat_290
GGAGATTGATTGATTGCCAACATCATTTTAGGGGAAGGGA
>Repeat_291
GTGTTCAGAATTTGTTACGGCAACGTAGATCGATCTATAC
>Repeat_292
AGAAGGTGATCCCAACCCTTCGCGTAGCTCTTTGAGGGGA
>Repeat_293
AGAATAATATCGTATACAAAGATATATCTCATTATATATG
>Repeat_294
TTAGTACGATATCGCTTCACACCTCGCGGTGCTTACACCT
>Repeat_295
TCGATCCCAAGAGTTCAAGATGTCGATGTCAGGAGTCAAT
>Repeat_296
ACGCTTTAGGCTCTCATAATCGATCGTACCCTAATAGAAT
>Repeat_297
ACGGCTTGAACGGCTGGCTTTACTTGAACGGCTTGAACGG
>Repeat_298
GGCCCCCCCTAGGTTTTGATGGCAAAGGCCCTCCCCCTT
>Repeat_299
TCCTCTTATGCACCTGTATCCTTCCTGCTACCTATTCTC
>Repeat_300
GTTGTTGAGGAGGTGGTCAATAGGTATCTATTGCGCCTG
>Repeat_301
TGAGATTCTTCGCGTTACTTATGAGATTATGCTATCCCA
>Repeat_302
TCAGGACCGTCTGCCCTCTAGAGCCTTATCTTCGAAGTA
>Repeat_303
CTGTCTCACGACGTTCTAAACCCAACTCACGTACCACTT
>Repeat_304
CCGGTACTCTCTCTCAACTGCTCAGATGAAAGCTGTAGC
>Repeat_305
ATGGTTTGAAATTAGCTATGAAAGAACCTATTTCACCAA
>Repeat_306
CCTGACTGGGTCTTACCTGAGCTCGAAGAGTTGGTCCTG
>Repeat_307
TTCCAGTCATTCTTTATATATGTAATAACACTAGATTCG
>Repeat_308
TTATATATGTTGCATGAGAGATGGCAAAATCGATGCATG
>Repeat_309
TTTCACCTCGTACGAGAACTTGTCTAGAGAAGGATTTCC
>Repeat_310
TTATTCCAGTCATTCTTTATATATGTAATAACACGCTTT
>Repeat_311
CCTTCGTTACATAAGGCATGGCAAGGCCCTTACACGTTA
>Repeat_312
TCAATTCAAACCGTTCAATTCAATTTGAGACGTGCCTT
>Repeat_313
AATGACTCGGATAATACTACATGGAGCTAGTGAAATTG
>Repeat_314
TCGATTCCATCCTGAGCTTATCGTTGCCCTTTCGGGTC
>Repeat_315
TTTATTCCCCTCAATCGATCAATCACTCAGGACTGACT
>Repeat_316
AGTGGAGTGGAGCTGGACACTTCAACTGAGATGGCTGG

>Repeat_317
AGTAGCGGGACAGAGCGCTTTGCTAGTGAAATAAATGG
>Repeat_318
AAACTCCGTACCCCAGTAGTCTACTTGCACCGGTGACC
>Repeat_319
CGAGGTAAGATGAAAGAGAAGATAGGTGGTTCGCAGAG
>Repeat_320
GATTGATTGCCAACATCATTTTAGGGGAAGGGAACACT
>Repeat_321
AACGTTAAGTGACTCGCCCCTCCCCCTCACTGTCTGTC
>Repeat_322
GCGTAATTACATATATAAAGAATGACTGGAAGAACAAA
>Repeat_323
TCCAGTCATTCTTTATATATGTAATAACACTAGATTCG
>Repeat_324
CTTACCTGAGCGAGTTAGTTCGGGAAGATAAAACCCTT
>Repeat_325
GACCAGTAGTGAGCGAACCCAACAGAGCCATTGAAGAA
>Repeat_326
CTTTCAGCAGTTCTTCCATACCGACTTGGCTGCCCGGC
>Repeat_327
CCTTGGTTCGGGACCTTGGAGTACTTGCCTACATTGGA
>Repeat_328
CCGGTTTAGATCAGGCCTTTATATACGTCATTTCGGGC
>Repeat_329
GAAATACCTCTATTTGCCGAAATAGACTATTGAGTATC
>Repeat_330
ATTTCCCTTGGGGTGTAATAGAAATGGTCAGGGCTCCT
>Repeat_331
ACCCTTTATTTCTTCCCCTCAAAGAGCTCTGCGAAGG
>Repeat_332
TATGTGTTCTGCAACCTTAGCCAGCCAATCTTCTTTT
>Repeat_333
ATAATATCGTATACAAAGATATATCTCATTATATATG
>Repeat_334
GACTTATGTCACGAATTCGCCTAACGCCTGGCTAAAA
>Repeat_335
GATTATTTGTTAAACAAATCCTTCGTATATCGCTCGT
>Repeat_336
CTAAGTATTTATTGAGTGGGGGGCTAAGTATTTATTG
>Repeat_337
ATAGATAGATAGATAGATAGATAGATAGATAGATAGA
>Repeat_338
ATAGTAGCGAGGGGTAGGTATCGGTCCGATAGGGATA
>Repeat_339
CAAGGAAGGCCAAGGATAAGTAGTTAGTATGAGCCCC
>Repeat_340
TGCCTCCTGCCCGTCGACGAGGAAAGAGCGACAAGGT
>Repeat_341
CTGGTTTCACGAATTTGGACTGGTTTCACGAATTTGG
>Repeat_342
ACAGGAGACAGACTACGAACAGGAGACAGACTACGAA
>Repeat_343
AGGTTTTGGAGACCTACGTTCTACCGAACTGAACTAA
>Repeat_344
AAAGCACCCCGCCAGTGCTTTGGCGACTTTCACTTTC
>Repeat_345

ACAGGCACACTTGTTCATATGTCAAGGGCTGGTAAGG
>Repeat_346
CTCGACCTACCTTAAGGAAGGGAGTAGTTTTTGGAGG
>Repeat_347
AAGGCGTAATTACATATATAAAGAATGACTGGAAGAA
>Repeat_348
TTGGTTCTACCAACCAGCAAACACGGGCTCGAACCCC
>Repeat_349
TCATTATATATGTAATTATGCTTGCCTTATGACTTGA
>Repeat_350
CTTAAGATGCCATAAGCAGCGGATCCACTCGAATAAG
>Repeat_351
CGAGGTACGTTAAGTGACTCGCCCGACCTAAGAAGGG
>Repeat_352
CCCAGCTATTTATCAGGGGCTTCACTCGAAGAAATGG
>Repeat_353
ACTAGGGTTGGCTCCTCGCAGTTCTCCCTTCAACACC
>Repeat_354
AATAGTAGAACTGAGCTCTATATACTTGAGGAATTTC
>Repeat_355
GCTTCGAGAGATCGGTCTGGTCCTACCTTGCTTATTG
>Repeat_356
TTTCCTCCTCCCGCTAGGGAGTACTCATGGTTCAGTT
>Repeat_357
AAACTCCCCACACAAGCACTGCCTGCCATATTGCTT
>Repeat_358
CTAAACCGATCATCATATCGGTCCCTAAGCCCCATT
>Repeat_359
GAGGGAACACTCAATTTCACCTACTCAATTTGAGGG
>Repeat_360
ACTCCACTAGAGATTGAAAACCTATTCTATTTAGTA
>Repeat_361
ATGTCCCAAACTTACTGCATTGGATCGGACAGATGG
>Repeat_362
CTCGTATCATTTCGGTAACTAGAGATAGAATAAAGA
>Repeat_363
TTCGGTGATGCGCTTCTTGAACCAGATGGATACTCA
>Repeat_364
AATCTATTGCGCGCTGCCTACAGGAACAGGGAAGGA
>Repeat_365
AGTGCAGATATAGCGCCTCATGCTGCAATCCATTCG
>Repeat_366
AAGGATATGATAAGGAGAGGACACAACACCTTAGAA
>Repeat_367
GTTCGCGCTAGAAGGATATGATAAGGAGAGGACACA
>Repeat_368
CCCTTCTTAGGTCGGGCGAGTCACTTAACGTACCTC
>Repeat_369
ATGTAATTACGCTTTAGGCTCTCATAATCGATCGTA
>Repeat_370
GGCCAAGGTTGCTACCTTATTGTTTGAAAGTCATTT
>Repeat_371
AACTAGAGATAGAATAAAGAATATAGAGCGCGCTCA
>Repeat_372
CCCTCGCCCTCCTACCTCGGGCGAGTCACTTAACGT
>Repeat_373
AGGTCCTGAAAGGTCTTACCTGAGCTAGCTAGAAGG

>Repeat_374
TTGGTCAGCCTTTACCTAACCAACTACCTGATACTA
>Repeat_375
CAGGCTAGGCGTAGCATATTGAAAGCTAGGCGTAAC
>Repeat_376
GCCAAGAGCCATAGTGAGTTGCTGTTCCCGTTTCCA
>Repeat_377
GTAATTACGCTTTAGGCTCTCATAATCGATCGTACC
>Repeat_378
GAGGTACGTTAAGTGACTCGCCCGACCTAAGAAGGG
>Repeat_379
GCGTAAGCATCTGGATAATCTGGAATGCGACGTGGC
>Repeat_380
GCTGCTGGTCATTCTTTATATATGTAATTACGCTTT
>Repeat_381
TACGTAATTACGCAATCGAAGAATTGATCAAATAAT
>Repeat_382
CGGTATCACTACCAAATACCACGATACCTACATTCT
>Repeat_383
ATAGTAGAACTGAGCTCTATATACTTGAGGAATTTC
>Repeat_384
CTTCCCTGCGGCCTTCACCTTTGTGGGTGGTGCTTT
>Repeat_385
CTGGGTGTGCCTCAAACAGATAGAATAGCTACCCA
>Repeat_386
AGCCAGCAATGGGGCTTAGGGACCGATATGATGAT
>Repeat_387
AGCCGACGTGGCAGCCGATGTGGTAGCCGACGTGG
>Repeat_388
TCCCACTCTTTCCTTTCAAGACGTTATGCCGCTTT
>Repeat_389
GTGGGGTGCAGGTGGTGATAATCACCCCGGGGTAT
>Repeat_390
CAATGCTGCTATTATATATGTAATTACGCTTTTCT
>Repeat_391
ATTCTTACTCACTTATTTCTTTGGCTTTGGCTGGC
>Repeat_392
ATTTCATAATTCCCATAGCCCCATCGTGAAAGGGG
>Repeat_393
TGGTAAAACAGTGAAATAAAGCAAGTAGGCTTCAA
>Repeat_394
ATGAATGAATGAGGATCGCTACTTGCTCACATGAA
>Repeat_395
TCGTTAGGTCAATAGTTCGGGAAAGCCTATACTAT
>Repeat_396
AACGGAGTACTCGCCCGACCTAAGAAGGGCGAGGA
>Repeat_397
ATGCCTTAGTCCTAATAGATATAGGAACAGACTTC
>Repeat_398
CCAAGGATAAGTAGTTAGTATGAGCCCCAGCTTGC
>Repeat_399
ATAGGTATATTGAAAGCTAGGAGTAACTAACATAT
>Repeat_400
CTATTGGGGACAAGGGACGGAACGACCTCTCGATC
>Repeat_401
TTATTCCAGTCATTCTTTATATATGTAATAACACT
>Repeat_402

GAATAATATCGTATACAAAGATATATCTCATTATA
>Repeat_403
AGGGCCTATTTGCTACAGTTAAAGCCCGGGACTTA
>Repeat_404
CCCTTCCTTATTTCCTTGCCCCCTCAAAGCCTGAG
>Repeat_405
GACCAATTACTCTACCCCGCTATCGTTCGTTATTC
>Repeat_406
CATTCTTTATATATGTAATTACGCTTTTCCTTCTT
>Repeat_407
ACCTTGCCCGATGCGGACAAGCGGCTTTGCTCCTT
>Repeat_408
ACTTATTGGGTGGGGGGCAACAGAACTCCTGTTAG
>Repeat_409
CTTCCCCCCTGTCCCTACTTATTGGGTGGGGGGCA
>Repeat_410
TTATTTGTTAAACAAATCCTTCGTATATCGCTCGT
>Repeat_411
TCCTCGAATCATTGTGCAATGTCCTAATCGAATG
>Repeat_412
TTCTTCCAGTCATTCTTTATATATGTAATTACGC
>Repeat_413
GGATAGATGGCTTGAGCGAGGGATAGATGGCTTG
>Repeat_414
TCGTGATGCTCCTGGTTATGCCGCTTCAACTGGT
>Repeat_415
TCGGTAACTAGCCAGGTATCTAAGTGCATAAAGA
>Repeat_416
CAGAATTTGTGTAGGCAATTACGGCAACTACGAT
>Repeat_417
TTTATATACGTAATAATACGCTTTTTATTCTTTT
>Repeat_418
ATGCTCAGCCCTGCCGAAAGAGAAAGAAAGAGAT
>Repeat_419
ACTCTCTGCCTTCAATCGTGTCTAGCCTAGCCCT
>Repeat_420
GCCTCATTAACTGCCTCTTTCCCACTCTTTCCTT
>Repeat_421
TACGATCGATTATGAGAGCCTAAAGCGTAATTAC
>Repeat_422
TGCCAGCCATCTCAGTTGAAGTGTCCAGCTCCAC
>Repeat_423
AAGCCCTTCGAACCAATAGCTGGCTGGTCTTGAC
>Repeat_424
AACAGGGAATTACTGAGAATCTAGGCCAGACCTA
>Repeat_425
AAAGCGTAATTACATATATAATAGCAGCATTGTC
>Repeat_426
CCTCAAATTTGATACAGTGAAGGTCTTCATTCGT
>Repeat_427
CGACGTCGAGAGACTCGAGGGACCGTAGCATGCC
>Repeat_428
CTAAAGCGTAATTACATATATAAAGAATGACTGG
>Repeat_429
ATTGTATACTTTCTATATGTACCTTTCCTACCTT
>Repeat_430
GAGTGGTTGTACCAGCCAAAACTCATCTACGTAT

>Repeat_431
CAAGCAATTGGTTGGGTCTTGGAGCGAACTACTC
>Repeat_432
AGGTGCCTCCCAGCCGGCGGATCGAATCGGAGTT
>Repeat_433
GCATAGCGAAGGCCTTGAGCTCTTTCTTAAGTGC
>Repeat_434
GGGGGAGGTACGTTAAGTGACTCGCCCGACCTAA
>Repeat_435
CCCATATTTTGTCATCGCTCTATTGCGGTGCGTT
>Repeat_436
ACTAGACTAGACTATTACCATGACCCCTTAATAC
>Repeat_437
CCAGTCATTCTTTATATATGTAATAACACGCTTT
>Repeat_438
TTATTCCAGTCATTCTTTATATATGTAATAACAC
>Repeat_439
GACGGGAATGTAAATAGAAAACATCCCCTGGGAA
>Repeat_440
TATGCTTATGGGTAAACTGGCCAAAGAGAAATTC
>Repeat_441
ATAAGGGCGGGCGAAGCGTGGAATGCGAGTTGGC
>Repeat_442
ATGTCGGTAACTAGAGCCAGGGAGACCAATAAGA
>Repeat_443
AGTCGAAAGGCGGTTCTGCGTGCGGGCTGCTGGG
>Repeat_444
GTCAATATGTTGAGGCTCTCATAAGGTATCTAGG
>Repeat_445
GATACATCCAATACCAATTAGTTAGTGAATACGG
>Repeat_446
GATGTCGTGAACGCCAGACCAAATTGAGTGGTTC
>Repeat_447
GATTGATTGCCAACATCATTTTAGGGGAAGGGA
>Repeat_448
AAAGTGGGTAAATTCACTAAGAAAAGGTGGCGT
>Repeat_449
GTAAGTAAAACTAATTATGTTGTACAGGTAACT
>Repeat_450
ATATCTTCCCGAACTCTAACTCAGGGTTTACCT
>Repeat_451
GAGAAGGGGCCGGCCGGGGGACTTCGTTTAAAT
>Repeat_452
ATTATTCCTAACAGCGTTCCCAAAAGGAACAGT
>Repeat_453
ATAGATAGATAGATAGATAGATAGATAGATAGA
>Repeat_454
GAGGGTGAGAAACGGTTTTAACGCTCGACCGAC
>Repeat_455
TATCCTTATCGCGCGAACTAAGGCAGTCGTTAG
>Repeat_456
TCAACTATGATCGGCAGCTATGTTCAACTATGA
>Repeat_457
GATCTATCTCTTTCTTCATCTCTTCCTGGACCT
>Repeat_458
ATTCATTCAGCCAGGAATCACTGAATATCACGG
>Repeat_459

TTATATACGTAATTACGCAATCGAAGAATTGAT
>Repeat_460
TTCCCCTCAATCGTTATAGTTCGGGAAGATATG
>Repeat_461
GGAAGATATTGAGTTTATGGCCTTAGAATGCTT
>Repeat_462
GCATCACCAGTCTAATCAGCTTCATTCCCAGCA
>Repeat_463
TATAATGAGATATATCTTTGTATACGATATTAT
>Repeat_464
CTCTTTGAGGGGAAGAAATCAAGGGCCAGGACC
>Repeat_465
ATTCGCTACTCTCTCTGCTAATAATTTGAACTT
>Repeat_466
CCCCCCTGTCCCTACTTATTGGGTGGGGGGCAC
>Repeat_467
AGATGTCGTGAACGCCAGACCAAATTGAGTGGT
>Repeat_468
CACCCTTGCCGTAAGCCTTAAGAAGCCATATAG
>Repeat_469
TGAGTCTTCAGGGTAAGGCCTTAAGTGGTCACT
>Repeat_470
AGCTGGCTATTACTGGGTACTAAGACTAGGTTC
>Repeat_471
GGTTTTCGGGCGGGTTACGAGAAAGCATATTA
>Repeat_472
TAGGCGTAACGTATTGAAAGCCAGTAAGTAAG
>Repeat_473
TAGTGGTACGCTCGCTCTCTTTACTCACGGAG
>Repeat_474
ATATCACGTATGGTAACAGCTTGTCTCAATCT
>Repeat_475
CTCTCCTTATCATATCCTTATCGCGCGAACTA
>Repeat_476
GTAGCGGGACAGAGAGAGGGGCTAGGTGAGGC
>Repeat_477
GATATTACGCAATCGATTCATATGCATGGGTT
>Repeat_478
TGTGTCTGGGGAATAGAACTGAGCTCTATACA
>Repeat_479
GTTACCTATCCCGTTACCTATCCCGTTACCTA
>Repeat_480
GGCAATGCTGCTATTATATATGTAATTACGCT
>Repeat_481
AGGCTCTCTTAATGGAAGGCTCTCTTAATGGA
>Repeat_482
TCTCAATGCCCGGGCATCCATCCGATGCATTC
>Repeat_483
GTGGAGCTGGACACTTCAACTGAGATGGCTGG
>Repeat_484
AAAGCGTAATTACATATATAATAGCAGCATTG
>Repeat_485
AACAGAGGTAACTAAACCATGCCTAAAGAGGA
>Repeat_486
CCTCAAAGAGCTAGAAGGTAAGGTCCTGCGCC
>Repeat_487
GTGGGAGAAGCCTATAGTATGAGGTCCGAGTT

```
>Repeat_488
CTTAATCTACTGCTGCTAGTGCTGGCTCTGCT
>Repeat_489
CGTAGTGATTCATATGACTCGCACTCAAACAT
>Repeat_490
GCTACATAAGAGCTTTGGAGATTGATTGATTG
>Repeat_491
AACTCCTATACTTGCCTTCTTTCTTTCTATCC
>Repeat_492
TTCCAGTCATTCTTTATATATGTAATAACACT
>Repeat_493
TGTAGTGGTGGAAACCCGGGTAATGGGGCAAA
>Repeat_494
CTTGGATTATGGTCCATCAGAGCAACCTCGTT
>Repeat_495
TTGCCAGCCCCCCTTATTAATTGAAGAGAGCT
>Repeat_496
CCCCCCTGTCCCTACTTATTGGGTGGGGGGCA
>Repeat_497
GATGTCGTGAACGCCAGACCAAATTGAGTGGT
>Repeat_498
GACGCAGCAGAGCGACCGGGAGCGGATTACCC
>Repeat_499
AAGGGAAACATAGCATGTCGCAAGAGCGAGG
>Repeat_500
CTTAAGCTCCCGATTTCTTGAGGGAGCGGAG
>Repeat_501
TACGATATTATGCAATCCGATACTTGTTGGT
>Repeat_502
CATATGCCTGCAACGATCCAGCAGCACAAAT
>Repeat_503
TGCTTAGAATGCTGCTGCCGGGAAGAATTAT
>Repeat_504
AGATATCTGTCTTGTGCTTTGACACGCTAAC
>Repeat_505
ATAGGGCTCTAGATACAGACTATCTAGCTTC
>Repeat_506
CAGAGCTCTTTGAGGGGAAGAAATATAGGGT
>Repeat_507
TTAGGCATGGTTTAGTTACCTCTGTTCCCTT
>Repeat_508
CCCGTTACCTACCCGGAAACCTACCTATTGA
>Repeat_509
AAATTTGAACATTCAATTTATATCTCTTTGT
>Repeat_510
TAAGCCTCCCTAAGGGTTAGAGGCATTAGGC
>Repeat_511
TTCTGTCAAATATCGAGATATAGCGGTAAAA
>Repeat_512
CTATCTCTAGTTACCGAAAGCATACGAGTCA
>Repeat_513
AACTACACTTGCTTCTGGATAGATGTGTGGG
>Repeat_514
CCCTCCTTCTTAGGTCGTCGGGCAAGCGGCT
>Repeat_515
CCGCCTACGTGCCCTTTACGCCCAGTCATTC
>Repeat_516
```

AATCAAGCTTTTGTTGCGACATGCTATGTTT
>Repeat_517
TAAGGCTCTAGAGGGCAGACGGTCCTGACTG
>Repeat_518
AAGGCGTAGCATATTGAAAGCTAGGCGTAAC
>Repeat_519
CCTCCGAAAGACATGTTCCCTCGAAAGACCT
>Repeat_520
TCTCTATCTCTCCGCCAAGGAAATAAGTAAC
>Repeat_521
CCTCAGGTAGCCACCGACCTACAGTTATCCC
>Repeat_522
TCCAGTCATTCTTTATATATGTAATAACACT
>Repeat_523
TCTTCCAGTCATTCTTTATATATGTAATAAC
>Repeat_524
TGGGTTGGTCCTTCCTGAAAGCCCTTGATTT
>Repeat_525
AAGGTCATCACTTACCTTCCTTCTAGCGGGA
>Repeat_526
TATTTATTCCCCTCAAAGAGCATAGCGTAAG
>Repeat_527
CCCTACCTATGAGGGAAGAAGATTTCGACGT
>Repeat_528
GTCATTCTTTATATATGTAATTACGCTTTAG
>Repeat_529
TCAATATATACAGCACCCGGCACAACACCCG
>Repeat_530
ACCATGTCTCCCGATCTCAGTACATATGGCG
>Repeat_531
ACGGATACATAAGGGCCGAAACTGCTAAGGG
>Repeat_532
CTTTACCCATACTTTCTTACTGCCTTCACCC
>Repeat_533
TTCCAGTCATTCTTTATATATGTAATAACAC
>Repeat_534
ATTATCTACGTAATTACGCAATCGAAGAATT
>Repeat_535
ATTGCTTGCTCTTAGGTAGGAGGGCAAGGAA
>Repeat_536
GCCTGCCTTATTTGGTTGGTTGGGAAGGCTA
>Repeat_537
ATTCCAAATGTGCTGTGCCAGGTCTTGCTTG
>Repeat_538
ACACAAATTCTGGGCGAGCCATACGAGTGGT
>Repeat_539
CCCACCTGTCATCGAGTGCATCGATCGCTAC
>Repeat_540
GGCTTTCCTTTCCCTTGGCTTTCCTTTCCC
>Repeat_541
CGCACGACGCATAAGAGCGAAATGTTCCGT
>Repeat_542
AGAAACAGAACGAGAAACAGAACGAGAAAC
>Repeat_543
TAAAGTGGAAAATCTCATTAGTAACGAGAA
>Repeat_544
CGGCTGCTCGCTCTTTGATCGGTGATTCAC

>Repeat_545
GGACTGTGTGGGTGCCTTACTGGGACTGTG
>Repeat_546
CGGCCAACTATTTAAGGAATCGAATCCCCT
>Repeat_547
TGGTCTTGCTTAGTGAGCAACTGGTAAATT
>Repeat_548
CCTCTGCTCTGTGGACGGATCGGGCTCTGT
>Repeat_549
AACTGTAGCAAATAGGCCCTTACACGTTAC
>Repeat_550
CTCGTATGATTTCGGTAACTAGAGATAGAA
>Repeat_551
CCCTCAAAGAGCATAGCGAAGGGTTGGGAT
>Repeat_552
CCTCACCCCTTGATTTCTTCCCCTCAAAGA
>Repeat_553
CACTACTTTAGCCCATCAAGAAAAGTCGTT
>Repeat_554
AGCGTAATTACATATATAATAGCAGCATTG
>Repeat_555
GCTAGGCGTAACGTATTGAAAGCCAGTAAG
>Repeat_556
ATTAAAGGACCTCCCTGGACTTGTCTGGAG
>Repeat_557
AGGTAAATAGTCAGAATGCCTTGCCTTGCC
>Repeat_558
GAATGACTGGAAGAATTGCTCGATTTTCTA
>Repeat_559
ATTCAGGCAGGTCAGGTTGTGCCAGTGTGA
>Repeat_560
AAAGCGTAATTACATATATAAAGAATGACT
>Repeat_561
CCCTTTCATTCTTTGAAAGAAGTAAGTGCC
>Repeat_562
CGTTCCTCGCCCTTCTTAGGTCGGGCGAGT
>Repeat_563
AGAAGTACGAGTTCCTAGTGTGTGCGCCCG
>Repeat_564
TTGATGGGCTAAAGTAGTGGTGGAATAGAA
>Repeat_565
GGTCAATCACGTTTATTAGAAGTGGACAAT
>Repeat_566
TATACTTGCCTTCTTTCTTTCTATCCGGAA
>Repeat_567
GTAATTACGCTTTAGCATCGTATGGGATCT
>Repeat_568
CGTTACGCACCCGTTCGCCACTTTGCTTGC
>Repeat_569
AGAGTCATTATATACGTAATTACACCTAAT
>Repeat_570
GCTTGGCTTTATACTTATCTCCCTGGCTCT
>Repeat_571
ACAGGCTAGGCGTAACGTATTGAAAGCCAG
>Repeat_572
AGGCGTAGCATATTGAAAGCTAGGCGTAAC
>Repeat_573

AGGCGTAGCATATTGAAAGCTAGGCGTAAC
>Repeat_574
GGTAGATTTTCCCCGACGCAATCCACAAAC
>Repeat_575
AAGTACCTAGCCCATCATGGTGAACCTCTC
>Repeat_576
TTCGCGTAGCTCTTTGAGGGGAATAAATAT
>Repeat_577
TAAAGTGACTCTTTCTTCAAAAGTCCATTT
>Repeat_578
CTTCCCAACCCACCTGTTCTCTTTCCTTTC
>Repeat_579
TCATTATATATGTAATTATGCTTGCCTTAT
>Repeat_580
GCAGGAAGGATACAGGTGCATAAGAGGAAC
>Repeat_581
GAGGTACGTTAAGTGACTCGCCCGACCTAA
>Repeat_582
CCCCTTCCTTATTTCCTTGCCCCCTCAAAG
>Repeat_583
GAGGATGCGCAAGCAGGCTCGACCAAAGGG
>Repeat_584
ACGCTTTAGGCTCTCATAATCGATCGTACC
>Repeat_585
TCCAGTCATTCTTTATATATGTAATAACAC
>Repeat_586
ATTATATATGTGATTATGCGAGCAATCAGA
>Repeat_587
TTTGCTTATGGTACTACCTTAGCTACCGGT
>Repeat_588
CCCCCCTGTCCCTACTTATTGGGTGGGGGG
>Repeat_589
GTTAGCGTCCCTTTTGCCATCTCGGGCCTC
>Repeat_590
GGCAACGTAGATCGATCTATAGTGACAGA
>Repeat_591
CCCGACCCGATGGTAGGAGCCTGCCCCAG
>Repeat_592
CATATTCATAGGATGCATATTCATAGGAT
>Repeat_593
GCTAGCTTTCAGTCAATCAATCAATCTCC
>Repeat_594
AAGCTCCCGATTTCTTGAGGGAGCGGAGG
>Repeat_595
AGGCTATAGCTAACGCTCTTAAGCCAGTT
>Repeat_596
CGAGCTCGTGTTTACAGGTGCGGCTGTAT
>Repeat_597
CTCACTCACTCACTCACTCACTCACTCAC
>Repeat_598
GTAACGTATTGAAAGCCAGTAAGTAAGTC
>Repeat_599
GGCTTCACACCCCACGCCTTTAATAATAA
>Repeat_600
CATGTCGGTAACTAGAGATAGCATAAAGA
>Repeat_601
GGGGCCATTAAAGGACCTCCCTGGACTTG

>Repeat_602
GGGGATTCGATTCCTTAAATAGTTGGCCG
>Repeat_603
TAGGAAGCACTTACTAGCAATAGGAAGCA
>Repeat_604
CCGGCGCAGGCCCCTATATTTATTCCCCT
>Repeat_605
AAAATGAAAAGATTTTGGAATCGCGTAAT
>Repeat_606
GATGGCTTGACTCACTAGGTAGGCATGGG
>Repeat_607
ATAGATAGATAGATAGATAGATAGATAGA
>Repeat_608
ATGAAATCGCTTGTCCGCATCGGGCAAGG
>Repeat_609
TGGGTGCCGCAAGGTTATACCCCCTATCT
>Repeat_610
CTTACTCATATTTATTCCCCTCAAAGAGC
>Repeat_611
GTCATTCGTTATATACGTAATTACGCTAT
>Repeat_612
TACTTACCTTCGTTATAAGGTAAGGGATC
>Repeat_613
CCCTCGCTTTCTGATAGACATAACGCAAA
>Repeat_614
TAACTAGCCAGGTATCTAAGTGCATAAAG
>Repeat_615
GCGTAATTACGTAGATAATGAATGACCGG
>Repeat_616
GTATTTCACGAACGTAAAGTTAGGGTGAT
>Repeat_617
GTATTAAGTAAGTGTTATGTAAGGCTGGT
>Repeat_618
TGGAAGTCGGGGCTACCCCGTCCCCATGG
>Repeat_619
AATAGCGTAGAAAATACAATAGCTAAAGA
>Repeat_620
AATAAAAGCATTTGCTGCAAAAGGGTTAG
>Repeat_621
GGATTGCATAATATCGTATAATATGCTTT
>Repeat_622
ACTCAGCAGCAGGGAGAAAATCTGTATTT
>Repeat_623
AAGGCTTGGTGGTATCAAGATGGTATCAA
>Repeat_624
ACCTACTCAATTCAAACCGTTCAATTCAA
>Repeat_625
AAAGCTGAGTTAGCGTGTAAAAGCACAAG
>Repeat_626
CTGTCTGTCCGTTCGGGAAAGGTTGTTCC
>Repeat_627
GCGTAACATAGGTATATTGAAAGCTAGGC
>Repeat_628
AGGCGTAACATAGGTATATTGAAAGCTAG
>Repeat_629
CTTTGAGGGGGCAAGGAAATAAGGAAGGG
>Repeat_630

GAGCTAGCTAGAAGGTAAGGTCAGTCATC
>Repeat_631
TTATATATGTAATTACGCTTTAGGCTCTC
>Repeat_632
GCGTAATTACATATATAAAGAATGACTGG
>Repeat_633
GCCACCTTAGCAGGCTGGACCTGACCCTT
>Repeat_634
TTGAGGGGAATAAATATAGGGATAACCTT
>Repeat_635
CCAGTCATTCTTTATATATGTAATAACAC
>Repeat_636
TTCTTCCAGTCATTCTTTATATATGTAAT
>Repeat_637
AGGTTTATAAGAGAGGCCGGAATGATGGC
>Repeat_638
CCCGTTACACTTAGCTGTAGAGAGATAAG
>Repeat_639
TCCTTATTTCCTTGCCCCCTCAAAGCCTG
>Repeat_640
CACCACTAGAAGCATAAGAAGCAAAGGTA
>Repeat_641
GTCATTCTTTATATATGTAATTACGCTTT
>Repeat_642
AGAATAATATCGTATACAAAGATATATCT
>Repeat_643
TTCCAGTCATTCTTTATATATGTAATAAC
>Repeat_644
TTCCTTGGTTCTGTCTCCCTGTCTCCCAG
>Repeat_645
TTCCCCCTTATTATTTAAGCGGTGACTCG
>Repeat_646
TATTTACTCGTATCATTTCGGTAACTAGA
>Repeat_647
TAAGAGAGGGCTCATTTATAGATAGGAAA
>Repeat_648
AAGCTCCCGATTTCTTGAGGGAGCGGAG
>Repeat_649
ATCATCATATCGGTCCCTAAGCCCCATT
>Repeat_650
GGGATAACCTTGTTCGAGCCGGCCGAAG
>Repeat_651
GTTCGCGCGACAAGGATACAATAAAGAG
>Repeat_652
TGTAACGTGTAAGGGCCTATTTGCTACA
>Repeat_653
AATTAGAAGGGTTGGGATAACCTACCAG
>Repeat_654
ATCCCTTACCTTATAACGAAGGTAAGTA
>Repeat_655
TATTGACTCATATGATTTCGGTAACTAG
>Repeat_656
CTGGCATGGTCAAGGCTCGCTTGCTGGC
>Repeat_657
TGCCGTTAGTTGCCGTTAGTCTTCAACC
>Repeat_658
GCTACTACTCTTGCTACTGCTGACTATT

>Repeat_659
CGGTACTCGCTGGTAGGCCATGCCAGGT
>Repeat_660
CTTAACTTAATTAGATTATACGGGCGGG
>Repeat_661
TATATATATATATATATATATATATATA
>Repeat_662
ATATATATATATATATATATATATATAT
>Repeat_663
GCCCCCCCACCCAATAAGTAACTTAGGA
>Repeat_664
ATTATTTATATATGTAATTACGCTTTAG
>Repeat_665
GCAGCCTTTCTTTCAATGTCCCCACCCA
>Repeat_666
CAGTTCAAAGTGCCTAACTAAGCAAGCC
>Repeat_667
ATAGCGTTCCCGATAGGAAATGACGACC
>Repeat_668
TAGGCGTAACGTATTGAAAGCCAGTAAG
>Repeat_669
CTTACCTTCTAACTCAGGTAAGTAATAA
>Repeat_670
GTCGTTCTGATGCTTCGACTGCTGCCGG
>Repeat_671
CGATGCTAAAGCGTAATTACATATATAA
>Repeat_672
GCAAGCTCATAAGCAAGCCTTAACCATA
>Repeat_673
CCTCAGGTGTCAAATGTGATGCTGTACC
>Repeat_674
AAATAAATGGATAGCCTCGCGGGACACA
>Repeat_675
TCAATACGTTAAAGCAATACTTCGTCAA
>Repeat_676
AATAGCGTAGAAAATACAATAGCTAAAG
>Repeat_677
CAAGCAACGCGCCACACAGTACACGTCT
>Repeat_678
AGTAAGGTCCTGCCCAGTAAGGTCCTGC
>Repeat_679
AGGGATTTATTGCTCACCTGACAGAGGA
>Repeat_680
ACCCGCTTACGGGGTGCTTTGGGCGGCG
>Repeat_681
TCTCTATTCCACCCAAAGATAAATTCCT
>Repeat_682
GATCGTACCCTAATAGCATAATATCGTA
>Repeat_683
TACCTGATACTACGCGGGCTCATCGAAC
>Repeat_684
TCCAGTCATTCTTTATATATGTAATAAC
>Repeat_685
CCAAGGATAAGTAGTTAGTATGAGCCCC
>Repeat_686
TAAGGCTCTAGAGGGCAGACGGTCCTGA
>Repeat_687

ACCGTTCAATTCAAACCGTTCAATTCAA
>Repeat_688
TCAATTCAAACCGTTCAATTCAAACCGT
>Repeat_689
CGTTACGCCTAGCTTTCAATATGCTACG
>Repeat_690
GCGTAACATAGGTATATTGAAAGCTAGG
>Repeat_691
GAATTTGTTACGGCAACGTAGATCGATC
>Repeat_692
ATTACATATATAAAGAATGACTGGAAGA
>Repeat_693
GCTATCGTTGTGCTGAAGGATCTGTAGG
>Repeat_694
TTTTAGGGGAATAAATATAGGGTCCTGG
>Repeat_695
CATGGCAAGGCCCTTACACGTTATAGGC
>Repeat_696
TTTATATATGTAATTACGCTTTAGGCTC
>Repeat_697
GCAGGAAGGATACAGGTGCATAAGAGGA
>Repeat_698
GGATTCTTCAGGCCTACCTGATACAGAG
>Repeat_699
TAAAGGCGTAGCATATTGAAAGCTAGGC
>Repeat_700
TCACCCAGGATCGCCCAGTACGCGTAAG
>Repeat_701
TCTAGCCCAGGATTTGCCAGGACTATTC
>Repeat_702
AGGGGAAGAAATCAAGGGCCAGGACCTT
>Repeat_703
TCTTTGAGGGGAAGAAATCAAGGGCCAG
>Repeat_704
CCAAGAGGCGATCACTCGATGACAGGTG
>Repeat_705
ACATGAAAAGACGATGTCCTAACCACTA
>Repeat_706
GAATAATATCGTATACAAAGATATATCT
>Repeat_707
ACGCTTTAGGCTCTCATAATCGATCGTA
>Repeat_708
TACGTAATTACGCAATCGAAGAATTGAT
>Repeat_709
GAATGATCCCCTAAATGCCCACCTCCTT
>Repeat_710
CGAGTCACCGCTTAAATAATAAGGGGGA
>Repeat_711
GACCCTGAAGCCCTGACCCACGTTGAGG
>Repeat_712
TCACACCCCACGCCTTTAATAATAATAA
>Repeat_713
TTTACTTATTGGGTGGGGGGAAGCCCT
>Repeat_714
ACTCCTTGATTTCTTAGGTATATTTTC
>Repeat_715
AGGTTCATAAAGAAGTTCAAGTGTAAT

>Repeat_716
AACAACTGGATATTAAACAACTGGATA
>Repeat_717
ATAAGCATCAGTTGAACCAGCTGAATT
>Repeat_718
ATATCTTCCCGAACCTAATTTTCACCC
>Repeat_719
GTAACGTATTGAAAGCCAGTAAGTAAG
>Repeat_720
TACCCTCTTCAACCCTTCGTCAGTAAA
>Repeat_721
CCGCTCCCTCAAGAAATCGGGAGCTTT
>Repeat_722
TATCCAACCAACCCATTCAAATAAATA
>Repeat_723
GATATACAGTAAGACTGAGTCTCTATC
>Repeat_724
AGATATACAGTAAGACTGAGTCTCTAT
>Repeat_725
TTAGGAGAGGACACAACACCTTAGAAC
>Repeat_726
CTCTCCCTCACCCATATGATGAGTTTC
>Repeat_727
GCCAGATAGCTACTACTACCTAGTGAG
>Repeat_728
TGCTCAGGCTTTGAGGGGAAGAAATAT
>Repeat_729
CGCTTGTCCGCATCGGGCAAGGTAGTT
>Repeat_730
CGCAGAGCTCTTTGAGGGGAAGAAATA
>Repeat_731
GATGACACTTATGTGTGCCGAACAAAC
>Repeat_732
CCCCTATTATTATAAAGGCTTTCCCAT
>Repeat_733
GAGCCAATGAGCAGGCAGGTTGAGCCA
>Repeat_734
CCGGGGAATACCGTACTAACCGGGAAA
>Repeat_735
TACTTATTGGGTGGGGGGAAGCCCTTA
>Repeat_736
GCTGTCCCTCTCCCACGAATGATATCC
>Repeat_737
GCCCTTGCTTTCTCTGCACCTGCCTTT
>Repeat_738
ATATATATATATATATATATATATATA
>Repeat_739
GCCCTTACACGTTATAGGCGGACCTAT
>Repeat_740
TTTCGGTAACTAGAGATAGCATAAAGA
>Repeat_741
AGGGAGAGGTACGTTAAGTGACTCACC
>Repeat_742
CTATTTTTCGGAAACCCAGACAGGTCC
>Repeat_743
TCGCTTGTCCGCATCGGGCAAGGGTGA
>Repeat_744

GAGCATAGCGTAAGGGATAACCTTACC
>Repeat_745
TGTAGCAAATAGGCCCTTACACGTTAC
>Repeat_746
CATACTTATTCATTTTAGGACCTGCTA
>Repeat_747
CGACAAGGAATTTCGCTACCTTAGGAC
>Repeat_748
GAGCATAGCGAAGGGTTGGGATAACCT
>Repeat_749
AGAGTCATTATATACGTAATTACACCT
>Repeat_750
TGAGGGTAATGAGGGTAATGAGGGTAA
>Repeat_751
TTATTGATTCTATCTCTAGTTACCGAA
>Repeat_752
GCGTAACATAGGTATATTGAAAGCTAG
>Repeat_753
AAATGATACGAGTCAATACGTTAAAGC
>Repeat_754
TAGGCAGTCGCAGGCAGGCAGAGCAGG
>Repeat_755
GCGAGTCACTTAACGTCCCTCACCCTC
>Repeat_756
CTTATATGATCTATTCCCACCCAACCG
>Repeat_757
GATGCTAAAGCGTAATTACATATATAA
>Repeat_758
GGGCTTGAAGAACACGGACACGGATGC
>Repeat_759
TTGAAAGAAAGCCAAATTTTATTCTTT
>Repeat_760
ACTCGCCCGACCTAAGAAGGGCGAGGA
>Repeat_761
ATCTTGGAAGGAAGCGCTTGCTTGGGT
>Repeat_762
AGCTTGACCCATTAATTCCTGCTGCGA
>Repeat_763
AGAATAATATCGTATACAAAGATATAT
>Repeat_764
GGTTATACCTTTCCCGTAGCGATAAGG
>Repeat_765
ATAAGCACCTGACATAGCCGATCATAG
>Repeat_766
CCCTTGGCTCGACGTATCTACAACTAC
>Repeat_767
ATCTCATTATATATGTAATTACGCTAT
>Repeat_768
AAAGCGTAATTACGTATATAATGAGGG
>Repeat_769
TCTTTGAAAGAAGTAAGTGCCGGTCAT
>Repeat_770
AGGAGAGAGCTACCCGCATATATACGT
>Repeat_771
CCAGTCATTCTTTATATATGTAATAAC
>Repeat_772
TGATAGACATAACGCAAAGGAAGACCT

>Repeat_773
TCGGGCGAGTCACTTAACGTCCCTCGC
>Repeat_774
ATCCAGGTGTAGATGCCGGTACTCAAT
>Repeat_775
CGTTCAATTCAATTTGAGACGTGCCTT
>Repeat_776
CGTTCAATTCAAACCGTTCAATTCAAA
>Repeat_777
TATAGAGCCAGTTCAAGCCCTAAGGCC
>Repeat_778
GCTAGGCGTAGCATATTGAAAGCTAGG
>Repeat_779
CGTAACATAGGTATATTGAAAGCTAGG
>Repeat_780
CGTAGCATATTGAAAGCTAGGCGTAAC
>Repeat_781
GTTTGAACTAAACAAGACACTGAATTC
>Repeat_782
GCGTAATTACATATATAAAGAATGACT
>Repeat_783
ATTTCTTCCCCTCAAAGGCTTTCAATA
>Repeat_784
AAAGTCCATTTCTTCCAGTCATTCTTT
>Repeat_785
TTATATATGTAATTACGCTTTAGGCTC
>Repeat_786
CATTCTTTATATATGTAATTACGCTTT
>Repeat_787
TTTATTCTCTCTCTAGTTACCGAAATG
>Repeat_788
GAAGGGGGAGGTACGTTAAGTGACTCG
>Repeat_789
ATCTGACACTCTGACCTATTGGGTTAT
>Repeat_790
TCAAAGAGCTACGCGAAGGGTTGGGAT
>Repeat_791
TTAATCAGGACAAGTTGGAAGGCCCCC
>Repeat_792
GCTCTTTGAGGGGAAGAAATCAAGGGC
>Repeat_793
TGATGCTCGCCCGGAGAGATCTTGATT
>Repeat_794
GGCTTATCGGAGTTACGAAGTTAGTGG
>Repeat_795
ACTTGCTGACAGAACAGATCTTGGTAT
>Repeat_796
AATATGGGGCCAGAAATTGCAGAGGAG
>Repeat_797
GATAGATACTCTTTTCCTACCTTATAG
>Repeat_798
AGTTTCCCTGCATGTGGCTCGTCCGTG
>Repeat_799
CCTTCCCCTTTACATACGCTTTCTGTT
>Repeat_800
ATCTGGTTCAAGAAGCGCATCACCGAA
>Repeat_801

GCCGTAAGCCTTAAGAAGCCATATAGT
>Repeat_802
ACGAGTAGCCGGGCCAAGAGATATATG
>Repeat_803
AGGCCCAGTAAAAGCAAGGGCATCACC
>Repeat_804
GAACTTCCAATCCGTAGAGAAAGAAC
>Repeat_805
AACCTGTAGGGATACCCGTAGTGAGA
>Repeat_806
CCTCGTACTTCTATTGGATAAGCCCT
>Repeat_807
CACAAGCCACCCCTACTAATCTAAGC
>Repeat_808
CTTTTTATCTTGCCTAAGAGCGTTAG
>Repeat_809
ATATATGTCTGAATGAAAACGAATGA
>Repeat_810
TCATGCGGGGATGTGGCTTTCATGGG
>Repeat_811
TTGATGACCGTGTACTTGGTGGACTT
>Repeat_812
GATATACAGTAAGACTGAGTCTCTAT
>Repeat_813
GGTCCTGGCCCTTGATTTCTTCCCCT
>Repeat_814
GTAAGTCGAGTAAGTCGAGTAAGTCG
>Repeat_815
AGTAGCGTAGCTACGATTGGAAGTAT
>Repeat_816
GCCCAACGACAACAAGCGTAGGAGGA
>Repeat_817
CTGAGCTCTGCGAAGGTAAGGTTATC
>Repeat_818
ATATCTTCCCGAACTCTAACTCAGGG
>Repeat_819
CCCTTGTTGAGGTCATACAAATATGA
>Repeat_820
ACTGCCTCACTGTAGAAAGTCACGCT
>Repeat_821
GTTCATTGCCTCTATCATTGGTATGG
>Repeat_822
CCGCTCCCTCAAGAAATCGGGAGCTT
>Repeat_823
ATAGTGTTATGGAGTTATGTCTTCTC
>Repeat_824
AAAACATAACTCCGGAGTTATGTTTT
>Repeat_825
TCGGTAACTAGAGATAGCATAAAGAG
>Repeat_826
CCATTGACCGGGGGCTTGTAAGGTCT
>Repeat_827
TGGTGGGCTGGCTTGCTGGTGGGCTG
>Repeat_828
TAGATCGTAGTTGCTTAACAAATTGC
>Repeat_829
TTTCGGTAACTAGAGAGAGAATAAAA

>Repeat_830
TACGATATTTCGCTACGATATTTCGC
>Repeat_831
AAAGCTGGAAGCTTAAAGCTGGAAGC
>Repeat_832
TTCTTCCTTACTATCTGTCTTTCAGT
>Repeat_833
CTTCTTCCCAATGAATGCGATAAGTG
>Repeat_834
GCAAATGAGAGGAAAGTTCGCACTTA
>Repeat_835
GCCGATGAACCCATTTAAGTTGATGA
>Repeat_836
TATATCGAAAAGCTTCTCATTTGCCA
>Repeat_837
TTACTTATGAGATTATGCTATCCCAT
>Repeat_838
AATTGCAGGCTGGGATCGTGTTCTTC
>Repeat_839
TATCACCTGAATGAAAGGTCAATCCT
>Repeat_840
GCGCCGTATCACCTGAATGAAAGGTC
>Repeat_841
TATATATATATATATATATATATATA
>Repeat_842
ATATATATATATATATATATATATAT
>Repeat_843
CTATAGATAGATAGATAGATAGATAG
>Repeat_844
TGCACTCGACTTACTAATTAATAAGA
>Repeat_845
AGGCTAAGGCAGAAGTTTACAGGTGT
>Repeat_846
GAGGTACGTTAAGTGACTCACCCGTT
>Repeat_847
AAATGGACTTTTGAAGAAAGAGTCAC
>Repeat_848
AGATATGAGGAAGGAGTTTCAGGAGC
>Repeat_849
TGAGGAGCTGGAGGATTTGGATTATG
>Repeat_850
CACGTTACGGCATGGCAAGGTATTCT
>Repeat_851
GCACCCCTTCTCCCGAAGTTACGGGG
>Repeat_852
AGTGTCTTGTTTAGTTCAAACGCGCC
>Repeat_853
AAACCCAACTCACGTACCACTTTCAT
>Repeat_854
CATTACTTTATAGATACGGCGTCAGT
>Repeat_855
TTTCATATCTTCCCGAACTCTAACTC
>Repeat_856
TAAGAAACTCCGATGGGAGCAGCAAC
>Repeat_857
CGTAACATAGGTATATTGAAAGCTAG
>Repeat_858

GAAAAGGTGGCGTATCGGGACCTTCC
>Repeat_859
AGGAGAGGACACAACACCTTAGAACG
>Repeat_860
GCAGGTAGGCAGTCAGTCCAGTACGT
>Repeat_861
TAGGCAGTCCCCAATCAAGCAGCCCT
>Repeat_862
GCAGGCAGGCAGAGCAGGTAGGCAGG
>Repeat_863
CCTGCACGCCAGCAGTCCCAGTACGT
>Repeat_864
ACTCATATTTATTCCCCTCAAAGAGC
>Repeat_865
TGCTAAAGCGTAATTACATATATAAA
>Repeat_866
ACACTTGGGCACCTCGGACGTACCAG
>Repeat_867
AACAGAGGTAACTAAACCATGCCTAA
>Repeat_868
TGGCACACGAACCAATAGCTAATCAC
>Repeat_869
CTGTATAAGGCCAGGTTCTTGAATGT
>Repeat_870
ACTCGCCCGACCTAAGAAGGGCGAGG
>Repeat_871
ACATTATATATGAGCATATGCATTTT
>Repeat_872
TGTAGCTTAGTAAAAAGCGTATCAAC
>Repeat_873
TCCTGGCTGGCTCTGTCTGGGCATCA
>Repeat_874
TTGTAATGCCCTTTCCTTTGGTGGTT
>Repeat_875
CACCCCTAAGTATAGTCTCGCAAGCC
>Repeat_876
GAGAGAAAACCATTTCAAGGGCTTCC
>Repeat_877
GAGTGAACTGTTCGGCAGATTCCCAC
>Repeat_878
AAGGGAGATAGTAACCCTAAAGAAAG
>Repeat_879
TTCATGTTCTCGAGTTGCAGAGAACA
>Repeat_880
AATGAAAGCATGCTCCGAGTATCGGT
>Repeat_881
ATATCTGTATATCGATATACAGATAT
>Repeat_882
TGAGCTCAAGACCTTTCCCTTCTAAA
>Repeat_883
GTAGAGGCAGGACTTATGCTTAGGCA
>Repeat_884
CGTTCAATTCAAACCGTTCAATTCAA
>Repeat_885
GCTAGGCGTAACGTATTGAAAGCCAG
>Repeat_886
TGTTATCCCAGAAGTAGGCAACTACG

>Repeat_887
AACCTTACTTACGAGCATGCCAGGCT
>Repeat_888
AATTTGTTACGGCAACGTAGATCGAT
>Repeat_889
CAGCAGTGCGGAATGGAGTTTCTCGT
>Repeat_890
CAATCATTTTGTTCCTTGCTCTCTCT
>Repeat_891
GCCTAGCTTTCAATACGTTACGCCTT
>Repeat_892
CCTTTGGTGGTTGCATCGGCATCGTA
>Repeat_893
ATATATCTTTGTATACGATATTATTC
>Repeat_894
TATACTTGCCTTCTTTCTTTCTATCC
>Repeat_895
AGGGGATGACCTTACCTGAGCTATGC
>Repeat_896
AACTCCTATACTTGCCTTCTTTCTTT
>Repeat_897
TGCTCAGGCTTTTAGGGGAATAAATA
>Repeat_898
AAGTCTTATTACGTATGTAATAACGA
>Repeat_899
ATCTCATTATATATGTAATTACGCTA
>Repeat_900
GCCCTTGATTTCTTCCCCTCAAAGAG
>Repeat_901
CTCAGGCTTTGAGGGGAAGAAATATA
>Repeat_902
GCTTGCCTTATGACTTGAAACGAAGT
>Repeat_903
TCTCTAGTTACCGAAATGATACGAGT
>Repeat_904
CAGGGTTGGGAAAGCGGCTTCCTTCG
>Repeat_905
AAGGCGTAGCATATTGAAAGCTAGGC
>Repeat_906
CCTCGATCGATCAATCACGAAGGACT
>Repeat_907
ACTTACTCTCTAGTGAAAGACTTCGT
>Repeat_908
CCTTATTCGTTCCTTCTGAACTTACT
>Repeat_909
GCTTTCAAATCTTTGCGCCTTGCCTT
>Repeat_910
GCTCTTTGAGGGGAAGAAATCAAGGG
>Repeat_911
GTCATTCTTTATATATGTAATTACGC
>Repeat_912
ATAATATCGTATACAAAGATATATCT
>Repeat_913
TTCCAGTCATTCTTTATATATGTAAT
>Repeat_914
GGTCGAGCGGCTCTACAACTCGCCCT
>Repeat_915

AAGGAAAGATAAGATCGAGTAAGTCG
>Repeat_916
CTATGTGAGGTGCTCTATGTGCTGTT
>Repeat_917
AGGAGGTCGAGGAACACGAAGATTTT
>Repeat_918
AAGGGTGAGGAGCGTAGGGACGGTCG
>Repeat_919
CCCACGGACTCAAGCAGTAAGTGAAT
>Repeat_920
CTACCAGCCTAAGAATAATTTCCGGG
>Repeat_921
GGGGGCACTTATTGGGTGGGGGGCAA
>Repeat_922
TGTGATTATGCGAGCAATCAGAGGCC
>Repeat_923
CTATATGGCTTCTTAAGGCTTACGGC
>Repeat_924
AGGTAAGGGATAACCTACACAAATTA
>Repeat_925
GGCCCTTTATTTATTCCCCTCAAAGA
>Repeat_926
TTTGGGTAGGGGCCCCAAAATAGAGG
>Repeat_927
TCCCTAAATGATGTTGGCAATCAATC
>Repeat_928
TTTTTGATTAGCAAAACGACAGAAAG
>Repeat_929
TCCAGCACAGATTGGATGCGTTGCTA
>Repeat_930
GCAGGCCGTACCTTTAAATAAGAGGG
>Repeat_931
CTGCTTAACCTGGACCTGGAACTGCT
>Repeat_932
AGGAGTGCGTTTTGCTTTACTAGGG
>Repeat_933
TTTAGGGCATGTAAAAAGGTAAGTC
>Repeat_934
GGTTTGATCTGGTCCGGGCAGGGAT
>Repeat_935
ACTCAATTTGAGGGAACACTCAATT
>Repeat_936
CTTCTTCCCTCATAGGTAGGGAAGG
>Repeat_937
GGTCTCCGGTAGCAAGATCTCGATC
>Repeat_938
CTCACTCACTCACTCACTCACTCAC
>Repeat_939
CAGAGCTCTTTGAGGGGAAGAAATA
>Repeat_940
AGTAAGTCGAGTAAGTCGCGTAAGT
>Repeat_941
TAAAGCAAGAGAAGGGGTAAGGTCT
>Repeat_942
CCCTATAGTCGAGGGGAAGGTAGGT
>Repeat_943
CTTTGAAGGGAATAAATATAAGTAG

>Repeat_944
CCCAGACCCCTATAGCAATAAAGGA
>Repeat_945
CTCCTTTCGCTGCTGGGGCTGCTGG
>Repeat_946
GCACTAGCAGGAAGGATATTATCTG
>Repeat_947
GGGAGAAAGTACGTCTACTTACCGG
>Repeat_948
GTGGCACACCCCCCCCACAAGCACC
>Repeat_949
TGTGTCCTCTCCTTATCATATCCTT
>Repeat_950
CGAACTCCTGGGGACTAAAAGCTCG
>Repeat_951
CCTTGCCCGATGCGGACAAGCGACT
>Repeat_952
GTCCGGCATTCGTAGATTAAGAGAC
>Repeat_953
CCCTGAGCAGAGCTCAGGTAAGACC
>Repeat_954
CTTTCAGTCAATCAATCAATCTCCT
>Repeat_955
AAGGAATGAAATCGCTTGTCCGCAT
>Repeat_956
TAGGCGTAACGTATTGAAAGCCAAG
>Repeat_957
AGAAAGCCCTTTAGGTTAGGATTCA
>Repeat_958
TCTCTAGTTACCGACATGATACGAG
>Repeat_959
ATATCCGATATCCGACATCGGAACT
>Repeat_960
CATAAGGAGAAGACATAACTCCATA
>Repeat_961
TAGGGTATAGGGTATAGGGTATAGG
>Repeat_962
ATTTCTTCCCCTCAAAGACTGAGCT
>Repeat_963
CTTTGAGGGGGCAAGGAAATAAGGA
>Repeat_964
CCTCACCTATTAATTAGTGGGAAGG
>Repeat_965
GTTTCAATGCCAGGTTTCAATCCAG
>Repeat_966
TGTCCTGGGCGTTGACGGAATGCAT
>Repeat_967
TACTTATTGGGTGGGGGGAAGCCCT
>Repeat_968
TTACTTATGAGATTATGCTATCCCA
>Repeat_969
TCCGTGAAACATGGAAGAAATGGAC
>Repeat_970
GTTACCTATCCCGTTACCTATCCCG
>Repeat_971
AAAGGTGCATCGTAAGTAATGCATA
>Repeat_972

CTAAGTATTTATTGGGTGGGGGGCT
>Repeat_973
ATATATATATATATATATATATATA
>Repeat_974
ATAGATAGATAGATAGATAGATAGA
>Repeat_975
TCGGTAACTAGAGATAGCATAAAGA
>Repeat_976
CCCTCACCAAATAAGTAACTTAGGA
>Repeat_977
GAGGTACGGAGTCGCTCTCCCGACG
>Repeat_978
GCTTGTTTACTTACGAGCATATCGA
>Repeat_979
GTCCTTCCCTCTCAATGCCCGGGCA
>Repeat_980
AGGACCTTACCTGAGCGGATGTAAC
>Repeat_981
CCCTTGATTTCTTCCCCTCAAAGAG
>Repeat_982
AGTGACATTATAGAACTCTCGGTCC
>Repeat_983
CTCTTTGAGGGGAAGAAAGAAAGGG
>Repeat_984
ACACGTTATAGGCGGACCTATTTGA
>Repeat_985
TGCTAAAGCGTAATTACATATATAA
>Repeat_986
AGGAGAGGACACAACACCTTAGAAC
>Repeat_987
CTAGTTACCGAAATGATACGAGTCA
>Repeat_988
AATCTATCCCGCTTCCAGCTTGCAG
>Repeat_989
AGGTCAATAGTTCGGGAAAGCCTAT
>Repeat_990
TTCGAAGCGGCTTCGCACCTCGTCC
>Repeat_991
TGTGTTCGTGCGCGACCAGTTGCAG
>Repeat_992
AGGAGAATGAAGTGTCTTGTTTAGT
>Repeat_993
TCCTGGAATACCACTCTTTCGTCTA
>Repeat_994
GATGATCCAGTCGATGAAGCAGTCG
>Repeat_995
ATTTATTCCCCTCAAAGAGCTCTGC
>Repeat_996
CCTTTATTTATTCCCCTCAAAGAGC
>Repeat_997
TACGTAATTACGCAATCGAAGAATT
>Repeat_998
CTGCCTACACGTACTGGACTGACTG
>Repeat_999
CTTTCCTTATCGAGGAGAACAACTC
>Repeat_1000
ACCATGCCAGCAAGCGAGCCTTGAC

>Repeat_1001
TGGCTGGCTATTCTCAGATACTTAC
>Repeat_1002
CTGTCTGTATCCTTCCCATCCCTGC
>Repeat_1003
TGAAGATACAGATCAAGACCCATAT
>Repeat_1004
CTTCGAAGACTTGTCCTGATTAACC
>Repeat_1005
GCTCAGGTCAGGTTATCCTTACCTT
>Repeat_1006
AATGGTTCCTTCGAAGCGGCTTCGC
>Repeat_1007
TCTTCTGGGGAGGCAAGTTACAACT
>Repeat_1008
CATCCCCGCAGGAGCATGCTACTAA
>Repeat_1009
GCAACAGTGTCCTGAGGGAGGAGTT
>Repeat_1010
TGCTTCTAAGAGAAGGGTTCATAGC
>Repeat_1011
CCTTATTCTATCTCTAGTTACCGAA
>Repeat_1012
CGCCAGACGAACGAAAGACAGGTTC
>Repeat_1013
TCTCAAGGTCAGATTTCAATTTCAC
>Repeat_1014
AAGGTCGAGGAGGGAGAGAGAGAAG
>Repeat_1015
AGGCTCTCAAGGGCGAGGCCCAAGC
>Repeat_1016
GGTCCTGAAAGGTCTTACCTGAGCT
>Repeat_1017
CAGGTTCCCCTACGGCTACCTTGTT
>Repeat_1018
ACAGACCCGAGAGAGAACCATATCC
>Repeat_1019
TTCCTTGGTATTGCACTTCCTTGGT
>Repeat_1020
TATTTGGGTTGACTAAGTTTCGCAT
>Repeat_1021
CACTCCATAGGTTGTTGTTGGGTGA
>Repeat_1022
GCTAGGCGTAACGTATTGAAAGCCA
>Repeat_1023
TTACGGCAACGTAGATCGATCTATA
>Repeat_1024
TTCGATAGCGTAATTACGTATATAA
>Repeat_1025
ATTACATATATAAAGAATGACTGGA
>Repeat_1026
TTGCCATTTTATTACCCCGATCTAC
>Repeat_1027
TTCCCCTCAAAGGCTTTCAATATGC
>Repeat_1028
GATAGGATAGCGACCTAAGGAGCGA
>Repeat_1029

CACTCAAACATCTCATTATATATGT
>Repeat_1030
GGTCCTGGTCCTGAGCTCTGCTCAG
>Repeat_1031
GCCCTTGATTTCTTCCCCTCAAAGA
>Repeat_1032
CTTGCTTCGTTCACTAAGGGTCAGG
>Repeat_1033
CTCAGGCTTTGAGGGGAAGAAATAT
>Repeat_1034
ACGAAGTATTTCACGAACGTAAAGT
>Repeat_1035
ATTATGCTTGCCTTATGACTTGAAA
>Repeat_1036
TCTCTAGTTACCGAAATGATACGAG
>Repeat_1037
TTTATTCTCTCTCTAGTTACCGAAA
>Repeat_1038
TACAGTTAAAGCCCGGGACTTATGT
>Repeat_1039
CATGATGAAGCAAACATGCATGATG
>Repeat_1040
TATACTCGCTATCGGATCCGGACCA
>Repeat_1041
ATATAAAGTGGTGAACGATTGAAGG
>Repeat_1042
AGGCGTAGCATATTGAAAGCTAGGC
>Repeat_1043
ACCTTACTAACCGGTGAAGACCTTA
>Repeat_1044
CGGGGCAAAATAAAGTGCCTTACAA
>Repeat_1045
CGAGGATGCGCAAGCAGGCTCGACC
>Repeat_1046
ATATCCGTATACAAATAGAGATATC
>Repeat_1047
TACGGGAATCGAACCCGTGTCTTCG
>Repeat_1048
TACTTACCCACCTACTTACCCACCT
>Repeat_1049
AGCTACCCGCACCTGTCTGGTCCGA
>Repeat_1050
CCTTGCCCGATGCGGACAAGCGGCT
>Repeat_1051
ACACTTGCCTTTAGGAATCTTAGGA
>Repeat_1052
ATTCATCCAATTCACTGAATCGCAC
>Repeat_1053
AAAGCGTAATTACGTATATAATGAG
>Repeat_1054
GCCTAGCTTTATTCCCTTTGAACGA
>Repeat_1055
ACAGCATTCACAGGCATCACACTTG
>Repeat_1056
AGGAAAGATAAGATCGAGTAAGTCG
>Repeat_1057
TAAGGTGTAATTACGTATATAATGA

>Repeat_1058
AAGCTTGGCAGCAATGTATTCCCCA
>Repeat_1059
TCACACCCCACGCCTTTAATAATAA
>Repeat_1060
TCTTTATTAGATGCCAATACCACTT
>Repeat_1061
TGCGATACCGCTTCAAAGTCACACG
>Repeat_1062
AGGGCCTTTCGGTAACTAGAGAGA
>Repeat_1063
TAAAGATGAAGAGAAAGGCCCGGC
>Repeat_1064
TTGCATTATATATTATAATATCCA
>Repeat_1065
GATAACCTACACACACAAATTCTG
>Repeat_1066
ATGCTGCTTATGCTGCTTATGCTG
>Repeat_1067
AGTGATCCCGGTCTTCGTATTGAC
>Repeat_1068
GTAAGGAAAGATAAGATCGAGTAA
>Repeat_1069
GGAAACAGACACACGGGAGACAGA
>Repeat_1070
CCCCTCACCTTCCCGAGGTCAGGT
>Repeat_1071
TTCTAAGGTGTTGTGTCCTCTCCT
>Repeat_1072
TTTATTTATTCCCCTCAAAGAGCG
>Repeat_1073
CCCCCCCTTTCTTTTCAATTAGTA
>Repeat_1074
CTCTCCCTCACCCATATGATGAGT
>Repeat_1075
AGCGATTCGCTCGTACCTCGCTCA
>Repeat_1076
GCTCTATATATATATATATATATG
>Repeat_1077
CTTTCAGTCAATCAATCAATCTCC
>Repeat_1078
GCCCTCCCTTTCAGTCAATCAATC
>Repeat_1079
TCACTATAGATCGATCTACGTTGC
>Repeat_1080
TCGCTTGTCCGCATCGGGCAAGGT
>Repeat_1081
CTCCTACGTCGAGAAAGCGGCTTC
>Repeat_1082
TTAGGCGTAACGTATTGAAAGCCA
>Repeat_1083
ATAATATCTAGAGATCCTCTCCCT
>Repeat_1084
AAAAGCATATTACTTACGAGCATG
>Repeat_1085
TAGTGACAGAATTACGGCACTATA
>Repeat_1086

CATTCCCTCAATTTCGGGGCCCCT
>Repeat_1087
ACGTAATAATACGCTTTTTATTCT
>Repeat_1088
CTTTGGGCGGCGCGTTTGAACTAA
>Repeat_1089
AGTCATTGCTTGAAGGTCATTGCC
>Repeat_1090
TCAATTCAAACCGTTCAATTCAAT
>Repeat_1091
GCTAATTCCATCTAATCCATTCAT
>Repeat_1092
ATTCTTATCCGCCGAGTGATATTA
>Repeat_1093
TAAGTATTTCCACATTATAATTGG
>Repeat_1094
TTGCAGGCTGGGATCGTGTTCTTC
>Repeat_1095
TGATCCCAAGGTAGGTGAGTTAGG
>Repeat_1096
ATATATATATATATATATATATAA
>Repeat_1097
TATATATATATATATATATATATA
>Repeat_1098
ATATATATATATATATATATATAT
>Repeat_1099
ATAGATAGATAGATAGATAGATAG
>Repeat_1100
GTAATTACGCCTTGATTTCGGCAT
>Repeat_1101
GCTGTGGCCTTATAATAAGCGGGG
>Repeat_1102
CTCACCCTAAGTGGGTGAGGTACG
>Repeat_1103
AATATATTTCAGTTTGAGGGGAAG
>Repeat_1104
TACTGCCTGGCCTGCCTTCTCTCT
>Repeat_1105
ATATATATATATATAATATATATA
>Repeat_1106
GGGGGAGGTACGTTAAGTGACTCG
>Repeat_1107
AAATTGAGTTAGATGGGTGGTAGC
>Repeat_1108
GCCTTGGCCTTATTGATCGAACTA
>Repeat_1109
AAGTCCTGACCCCCTTCCTTATAT
>Repeat_1110
CTGAGTTCTGCTTTGGTCGATCTG
>Repeat_1111
CTTACTCATATTTATTCCCCTCAA
>Repeat_1112
ATCACACTTGCTTAAATATGCTTG
>Repeat_1113
TGCATTGACCGGTGGTTATGGGCT
>Repeat_1114
GATCTAAGCTCAAGTGCTTTATGA

>Repeat_1115
ACCCTTCTAACGCGAAGTCAACCC
>Repeat_1116
ACCCAGTAAGGTCCTGATCAACCC
>Repeat_1117
TGAGTCACTTAACGTACCTCACCC
>Repeat_1118
TAAAGCGTAATTACATATATAATA
>Repeat_1119
ATAGGTCCGCCTTTCCCGAATGGG
>Repeat_1120
CTGAGTTAGAGTTCGGGAAGATAT
>Repeat_1121
TAGTCATTGCCTTAAGGTCATTGC
>Repeat_1122
TCCCCTCGCTACTTACTATAGGGG
>Repeat_1123
CGGTGGAACCGGTGAACCACGCAT
>Repeat_1124
CTACGTTGCCGTAACAAATTATGA
>Repeat_1125
CGCAGTGACAACCTCTTACGAATG
>Repeat_1126
CTAAAGCGTAATTACATATATAAA
>Repeat_1127
TTTATTCCCCTCAAAGAGCTCTGC
>Repeat_1128
GCTTGCTGATCCATCAGCAGATCC
>Repeat_1129
TATTCCTTCGCGCTTCGATGGGAA
>Repeat_1130
GGGCCATTAAAGGACCTCCCTGGA
>Repeat_1131
CTGTGCCCACGCACCCAAGCAAGC
>Repeat_1132
ATAATATCGTATACAAAGATATAT
>Repeat_1133
TTGGTCGAGTCACTTAACGTACCT
>Repeat_1134
CCTAACGCCTTAAATGAGTGGGCT
>Repeat_1135
TTATTCTATCTCTAGTTACCGAAA
>Repeat_1136
ATTTTACTTATTGGGTGGGGGGGC
>Repeat_1137
TGATGTATAATTACGTATATAATT
>Repeat_1138
TGAGGGGAATAAATATAGGGTGAG
>Repeat_1139
AACAGAGAGGCAAGCTAGAACAGA
>Repeat_1140
TTGGATACGCCTTCACTTGGCCGC
>Repeat_1141
GGTCGAGCCTGCTTGCGCATCCTC
>Repeat_1142
GGGCGAGTCACTTAACGTCCCTCG
>Repeat_1143

AACCAGATATGAACTGGTACCATA
>Repeat_1144
GAAAAATCCCTCGCATAATATCAT
>Repeat_1145
TCAATTCAAACCGTTCAATTCAAA
>Repeat_1146
TTAGTTACGCCTAGCTTTCAATAT
>Repeat_1147
TACTGTGCCATCCTGGGTGACGTG
>Repeat_1148
CATTCTGTCTGTCACTATAGATCG
>Repeat_1149
GTAGGCAACTACGATCTATAGTGC
>Repeat_1150
CCCAGCGTGAGGTGCACCCCCAAA
>Repeat_1151
AGGCGTAACGTATTGAAAGCCAGA
>Repeat_1152
TAGGCGTAACGTATTGAAAGCCAG
>Repeat_1153
ATATTGAAAGCTAGGAGTAACTAA
>Repeat_1154
GGCAACTACGTACGATCTATAGTG
>Repeat_1155
ATTATATATGTAATTACGCTTTAG
>Repeat_1156
TCATTATATATGTAATTACGCTTT
>Repeat_1157
ATTACATATATAAAGAATGACTGG
>Repeat_1158
GCGTAATTACATATATAAAGAATG
>Repeat_1159
GTCGACCAGGCCCTCTCCCCTATC
>Repeat_1160
AGGGCGAGTATAACCCAATAGGTC
>Repeat_1161
TTGAACGGGTATCCATTGAAGGGG
>Repeat_1162
ACGAGCATGAGCCTAGTCTTGTTG
>Repeat_1163
AAAGCGTAATTACATATATAATAA
>Repeat_1164
AAAAGCGTAATTACATATATAATA
>Repeat_1165
AAAAAGCGTAATTACATATATAAT
>Repeat_1166
AGAGAGAGAATCAGAGGTCAGGCA
>Repeat_1167
TATGACTCGCTTACACTCAAACAT
>Repeat_1168
AAGAGCTAAACGCACCTCTATTTC
>Repeat_1169
GGCTTTGAGGGGAAGAAATATAAG
>Repeat_1170
ATCGTATACAAAGATATATCTCAT
>Repeat_1171
TTTATATATGTAATTACGCTTTAG

>Repeat_1172
AAGTTACCAAACCTATACCTTAAG
>Repeat_1173
CCAGTTTACCCATAAGCGAATGAC
>Repeat_1174
AATGCTAGTTCAACTGGTGATGCT
>Repeat_1175
AAGGTCCTTCAAGGTCAACCCAGT
>Repeat_1176
GGTAAGGTCCTTCAAGGTCAACCC
>Repeat_1177
AGGGGAATAAATATGAAGCGTAAC
>Repeat_1178
AGGCGTAGCATATTGAAAGCTAGG
>Repeat_1179
ATTGGAGAAGCGTGAGCCGTTATT
>Repeat_1180
ATCTCAGTACATATGGCGTAAGAC
>Repeat_1181
ATACGATATGACTAATGATACCGA
>Repeat_1182
ACCCAACTACTCGATCTATCTAAC
>Repeat_1183
TCTTTGAGGGGAAGAAATCAAGGG
>Repeat_1184
GCCTTAATTAATCGTTCCTAGGCA
>Repeat_1185
ATATAAGTAATTACGCTTTAGCAT
>Repeat_1186
TTCTTTATATATGTAATAACACGC
>Repeat_1187
CCAGTCATTCTTTATATATGTAAT
>Repeat_1188
GAGCGTAGCAGGCAGAGCAATGAA
>Repeat_1189
CTATATTCTTTTCACTGGCCTATT
>Repeat_1190
CACCTTGCCCGATGCGGACAAGCG
>Repeat_1191
CGCCTCCACCCATCCAGCCTGAGA
>Repeat_1192
AAAATAGAGGGTTTTAACACGAAA
>Repeat_1193
GAGGAACACGAAGATTTTGGGGCC
>Repeat_1194
AACTCTTCAGTGGGGCACGAACTA
>Repeat_1195
ACACAATTCTTCCGGTTATGCCTG
>Repeat_1196
ATTGAATGAGACTGAAAGGCTTAT
>Repeat_1197
TAGGTTAATACCAATAGGTGCCTA
>Repeat_1198
TACAAGACCTGGCATTGGAATGAC
>Repeat_1199
TTAGGACTAAGGCATTTAGCCGGG
>Repeat_1200

GCCTGGCATGCTCGTAAGTAAGGT
>Repeat_1201
TATGCCTGGCATGCTCGTAAGTAA
>Repeat_1202
AGGATGAGCGAGGTACGAGCGAAT
>Repeat_1203
ATTAAGTAGTTCGTTAGGTCAATA
>Repeat_1204
GAGGAAAGATAAGATCGAGTAAGT
>Repeat_1205
AAGGTGTAATTACGTATATAATGA
>Repeat_1206
CTTGATTGAATAGGGCGGAAGCCC
>Repeat_1207
ACTCGTATCATTTCGGTAACTAGA
>Repeat_1208
GATGCCCTTGCTTTTACTGGGCCT
>Repeat_1209
CAGTGACACATAGACACCGCTGCC
>Repeat_1210
CCATCGAAGCAAGTCCCCAAAATA
