# Supplementary material for: The mitochondrial genome sequence of Abies alba Mill. reveals a high structural and combinatorial variation
Source: BMC Genomics. 2022 Nov 28;23:776. doi: 10.1186/s12864-022-08993-9 (PMC9703787; doi:10.1186/s12864-022-08993-9)

Additional file for "The mitochondrial genome sequence of *Abies alba* Mill. reveals a high structural and combinatorial variation" by Birgit Kersten, Christian Rellstab, Hilke Schroeder, Sabine Brodbeck, Matthias Fladung, Konstantin V. Krutovsky, Felix Gugerli

Additional file 5: Size distribution of repeats ≥ 50 bp identified in the Abies alba mitogenome (repeats according to Additional file 1: Table S4)

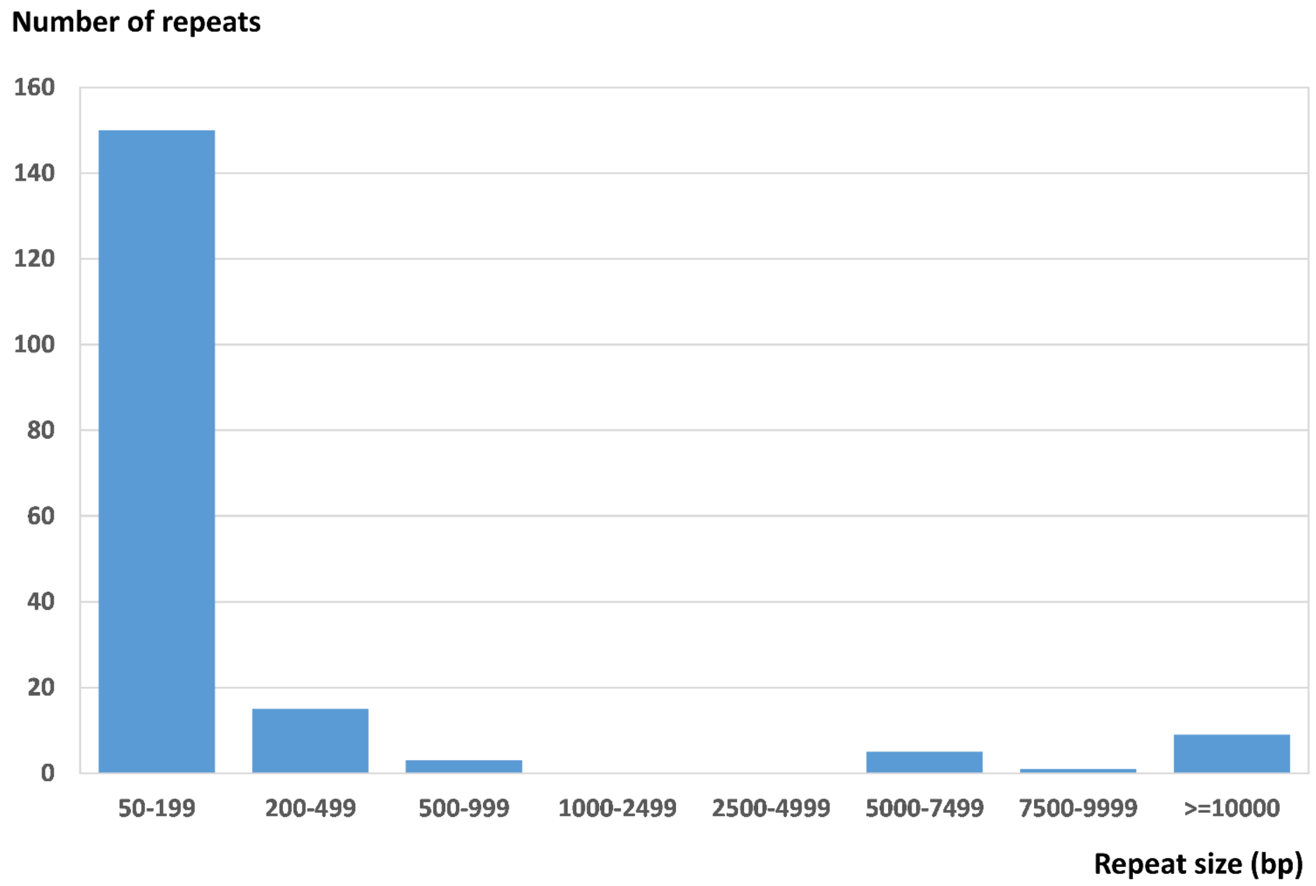

Supplement: Supplementary file 5 — Additional file 5. Size distribution of repeats ≥ 50 bp identified in the Abies alba mitogenome (repeats according to Additional file 1: Table S4). [file 12864_2022_8993_MOESM5_ESM.pdf]
